# Tracking Marsupial Evolution Using Archaic Genomic Retroposon Insertions

**DOI:** 10.1371/journal.pbio.1000436

**Published:** 2010-07-27

**Authors:** Maria A. Nilsson, Gennady Churakov, Mirjam Sommer, Ngoc Van Tran, Anja Zemann, Jürgen Brosius, Jürgen Schmitz

**Affiliations:** Institute of Experimental Pathology (ZMBE), University of Münster, Münster, Germany; Massey University, New Zealand

## Abstract

The Australasian and South American marsupial mammals, such as kangaroos and opossums, are the closest living relatives to placental mammals, having shared a common ancestor around 130 million years ago. The evolutionary relationships among the seven marsupial orders have, however, so far eluded resolution. In particular, the relationships between the four Australasian and three South American marsupial orders have been intensively debated since the South American order Microbiotheria was taxonomically moved into the group Australidelphia. Australidelphia is significantly supported by both molecular and morphological data and comprises the four Australasian marsupial orders and the South American order Microbiotheria, indicating a complex, ancient, biogeographic history of marsupials. However, the exact phylogenetic position of Microbiotheria within Australidelphia has yet to be resolved using either sequence or morphological data analysis. Here, we provide evidence from newly established and virtually homoplasy-free retroposon insertion markers for the basal relationships among marsupial orders. Fifty-three phylogenetically informative markers were retrieved after in silico and experimental screening of ∼217,000 retroposon-containing loci from opossum and kangaroo. The four Australasian orders share a single origin with Microbiotheria as their closest sister group, supporting a clear divergence between South American and Australasian marsupials. In addition, the new data place the South American opossums (Didelphimorphia) as the first branch of the marsupial tree. The exhaustive computational and experimental evidence provides important insight into the evolution of retroposable elements in the marsupial genome. Placing the retroposon insertion pattern in a paleobiogeographic context indicates a single marsupial migration from South America to Australia. The now firmly established phylogeny can be used to determine the direction of genomic changes and morphological transitions within marsupials.

## Abstract 2

Genome-wide comparisons of shared retroposon insertion patterns resolve the phylogeny of marsupials, clearly distinguishing South American and Australian species and lending support to Didelphimorphia as the basal split.

## Author Summary

Ever since the first Europeans reached the Australian shores and were fascinated by the curious marsupials they found, the evolutionary relationships between the living Australian and South American marsupial orders have been intensively investigated. However, neither the morphological nor the more recent molecular methods produced an evolutionary consensus. Most problematic of the seven marsupial groups is the South American species *Dromiciops gliroides*, the only survivor of the order Microbiotheria. Several studies suggest that *Dromiciops*, although living in South America, is more closely related to Australian than to South American marsupials. This relationship would have required a complex migration scenario whereby several groups of ancestral South American marsupials migrated across Antarctica to Australia. We screened the genomes of the South American opossum and the Australian tammar wallaby for retroposons, unambiguous phylogenetic markers that occupy more than half of the marsupial genome. From analyses of nearly 217,000 retroposon-containing loci, we identified 53 retroposons that resolve most branches of the marsupial evolutionary tree. *Dromiciops* is clearly only distantly related to Australian marsupials, supporting a single Gondwanan migration of marsupials from South America to Australia. The new phylogeny offers a novel perspective in understanding the morphological and molecular transitions between the South American and Australian marsupials.

## Introduction

The phylogenetic relationships among the four Australasian and three South American marsupial orders have been intensively debated ever since the small species *Dromiciops* was taxonomically moved from Didelphimorphia into the new order Microbiotheria and the cohort Australidelphia was erected based on ankle joint morphology [1]. Australidelphia comprises the four Australasian marsupial orders and the South American order Microbiotheria, a close relationship suggesting a complex ancient biogeographic history of marsupials. However, the exact phylogenetic position of Microbiotheria within Australidelphia has so far eluded resolution. Moreover, sequence-based attempts to resolve the positions of the South American opossums (Didelphimorphia) and the shrew opossums (Paucituberculata), which appear some few million years apart in the South American fossil layers close after the Cretaceous-Tertiary boundary [2], relative to Australidelphia have so far been futile (e.g., [3],[4]).

The two recently sequenced marsupial genomes of the South American opossum (*Monodelphis domestica*) [5] and a kangaroo, the Australian tammar wallaby (*Macropus eugenii*), provide a unique opportunity to apply a completely new approach to resolve marsupial relationships. The insertion patterns of retroposed elements, pieces of DNA that are copied via RNA intermediates and pasted randomly elsewhere in the genome, have successfully resolved the more than 130 million-year-old branch of therian mammals [6] and early placental mammalian divergences [7] as well as relationships within other mammalian orders [8]. Because the insertion sites are effectively random and parallel insertions or exact excisions are very rare [9], the shared presence of retroposed elements at identical orthologous genomic locations of different species, families, or orders is a virtually homoplasy-free indication of their relatedness. Thus, the interpretation of retroposon markers is simple and straightforward: the presence of one of these elements in the orthologous genomic loci of two species signals a common ancestry, while its absence in another species signals a prior divergence [10]. No other sequenced mammalian genome has shown as high a percentage of discernible retroposed elements as marsupials (52%) [5], an extremely large number of possible informative markers.

In addition, because young retroposed elements can insert into older elements, but older, inactive elements are not capable of inserting into younger ones, nested retroposon insertion patterns provide invaluable information about the relative times during which given retroposon families integrated into genomes. We used the transposition in transposition (TinT) application [11] to screen for such nested transpositions and to provide a complete picture of the succession of ancient retroposon activities so as to aid in the proper selection of element groups for resolving different parts of the marsupial tree.

## Results/Discussion

**Figure 1:**
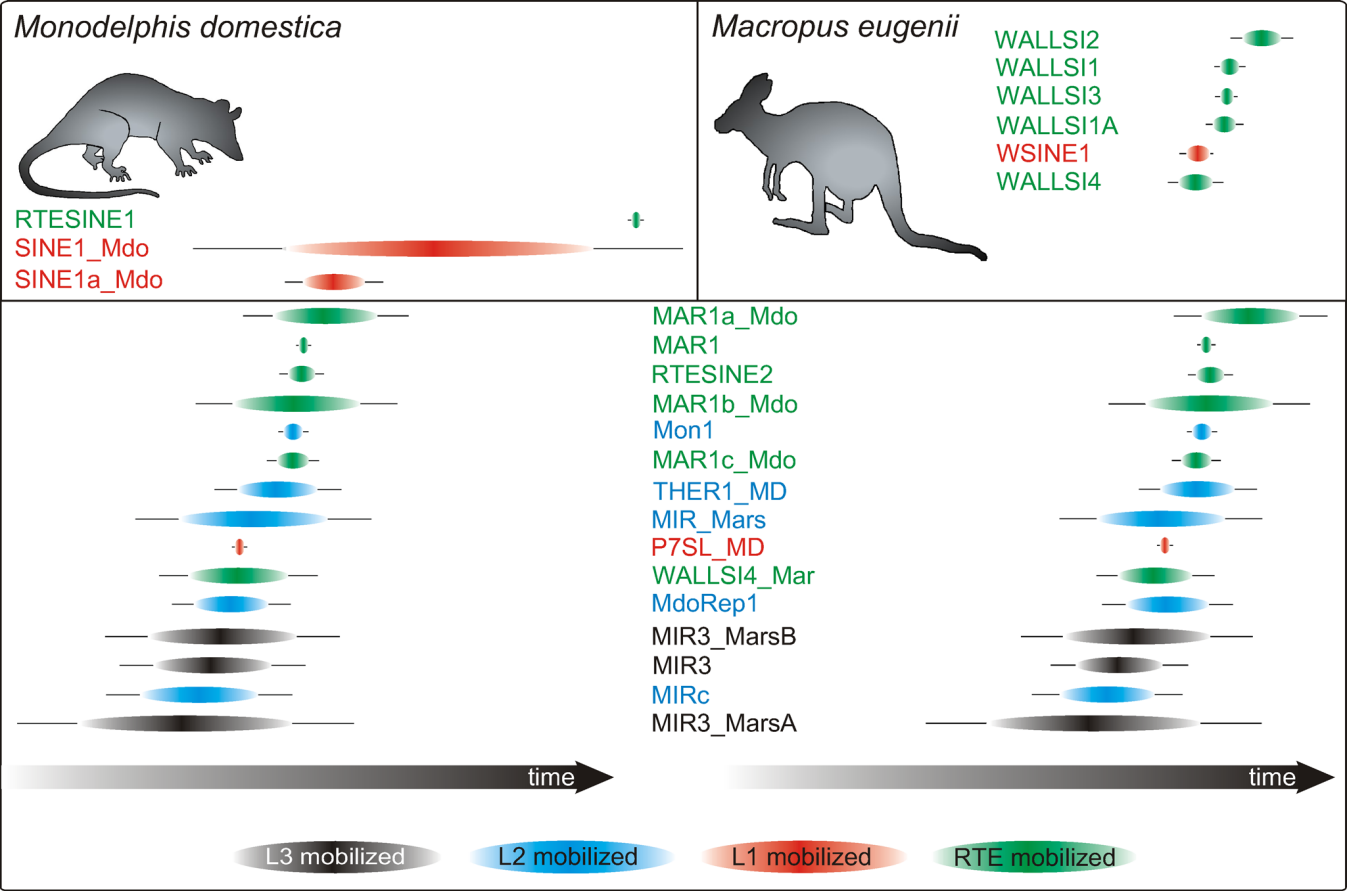
Transposition in transposition (TinT) pattern of selected marsupial SINEs. In genomes there is an intricate association between SINE elements and the much longer long interspersed elements (LINEs), as the replication of SINEs ultimately depends on the enzymatic machinery of LINEs. Using the TinT method in marsupial genomes, we detected independent SINE-LINE associations that overlapped in time. The L1 system dominates SINE retropositions in Didelphimorphia (SINE1_Mdo, SINE1a_Mdo). The retroposon-like transposable element (RTE) system predominates in the lineage leading to the kangaroo (WALLSI1-4), and LINE2- (MIR, MdoRep1, THER1_MD) and LINE3- (MIR3) mobilized SINE systems are present in both lineages and were active over long periods of marsupial evolution. Experimental screening revealed the activities of two, sometimes three, SINE-LINE associations at some deep nodes. For instance, at least three SINE-LINE associations were active in the common ancestor of the four Australasian orders (RTE-WALLSI1a/L1-WSINE1/L2-MIR_Mars) (Figure S1). In most other mammalian genomes, only one SINE-LINE group was active at a time; thus, the discovery of multiple groups may indicate a long branch and/or overlapping activity. As several different SINE-LINE systems were also active at the Australidelphia node (RTE-Mar1a,b,c_Mdo/L1-WSINE1/RTE-WALLSI3), we favor overlapping, extended activity of retroposition systems in marsupials. The extended presence of diverse SINE transposition systems found in marsupial genomes is unique in mammals. Twenty-four SINE subfamilies were extracted from genomic data of *M. domestica* and *M. eugenii* to screen for nested insertions, revealing information about their relative activity periods. Elements shown in black denote L3-, those in blue L2-, those in green RTE-, and those in red L1-mobilized SINEs. Ovals represent the 50% probability of the activity distribution and horizontal lines indicate the 90% probability of the activity distribution. Relative time axes are given at the bottom.

After a complete screening of the opossum and kangaroo genomic sequences using the TinT algorithm, we recovered 8,245 and 4,499 nested retroposon insertions, respectively (Table S1). We then calculated the frequencies and time scales of short interspersed element (SINE) insertions using the likelihood approach implemented in TinT. The resulting pattern (Figure 1) revealed three different groups of retroposed SINEs: (1) elements specific for the lineage leading to opossum (RTESINE1, SINE1_Mdo, SINE1a_Mdo), (2) elements specific for the lineage leading to kangaroo (WALLSI1-4, WSINE1), and (3) a compiled group of elements active in both marsupial lineages. These three groups of elements were then used as a basis to screen for phylogenetically informative markers present in (1) the opossum lineage, (2) the branches leading to kangaroo, and (3) to find marsupial monophyly markers.

**Figure 2:**
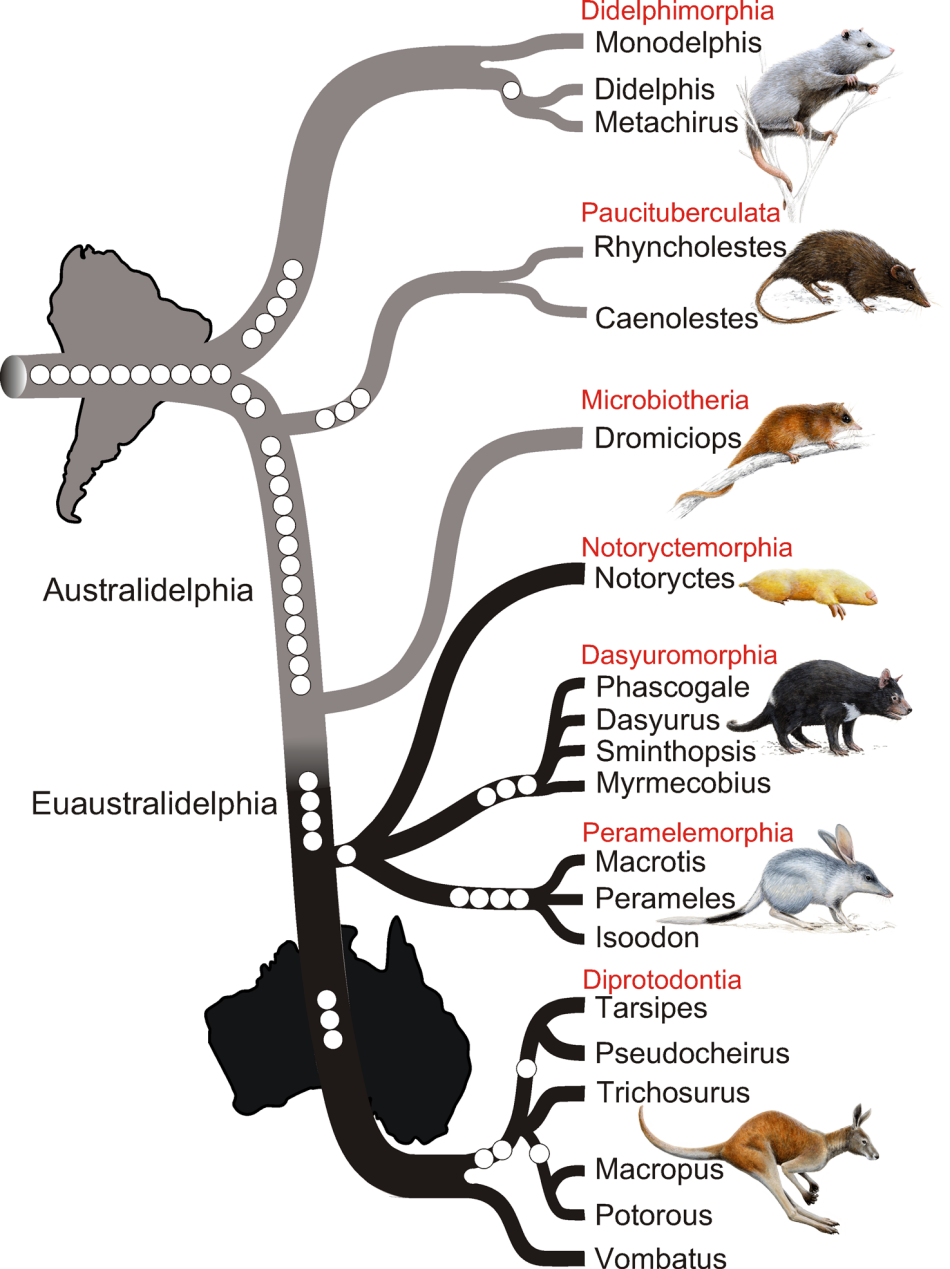
Phylogenetic tree of marsupials derived from retroposon data. The tree topology is based on a presence/absence retroposon matrix (Table 1) implemented in a heuristic parsimony analysis (Figure S3). The names of the seven marsupial orders are shown in red, and the icons are representative of each of the orders: Didelphimorphia, Virginia opossum; Paucituberculata, shrew opossum; Microbiotheria, monito del monte; Notoryctemorphia, marsupial mole; Dasyuromorphia, Tasmanian devil; Peramelemorphia, bilby; Diprotodontia, kangaroo. Phylogenetically informative retroposon insertions are shown as circles. Gray lines denote South American species distribution, and black lines Australasian marsupials. The cohort Australidelphia is indicated as well as the new name proposed for the four “true” Australasian orders (Euaustralidelphia).

Three different search strategies (see Materials and Methods) revealed ∼217,000 retroposon-containing genomic loci. Highly conserved exonic primers were generated for 228 loci and experimentally tested on a small set of species. After carefully screening the sequences, we selected 32 loci based on criteria outlined in the Materials and Methods section for amplification in 20 marsupial species (Table S2). We carefully aligned and analyzed approximately 440 marsupial sequences to reveal 53 informative markers (Figure 2, Table 1).

**Table 1:** Presence-absence table of the marsupial markers.

Marker	Species																				
	Md	Dv	Mn	Rr	Cf	Dg	Nt	Pt	De	Sc	Mf	Ml	Pg	Io	Tr	Pp	Tv	Mr	Pt	Vu	Hs
08a	+	+	+	?	+	+	+	?	+	?	+	+	?	?	+	+	+	+	+	+	−
14a	−	−	−	−	?	?	−	+	+	+	+	−	−	−	?	−	?	−	−	?	−
14b	+	+	+	+	?	?	+	+	+	+	+	+	+	+	?	+	?	+	+	?	−
20a	+	+	+	?	−	−	−	?	−	−	−	−	−	−	−	−	−	−	−	−	−
20b	−	−	−	?	−	−	−	?	+	+	+	−	−	−	−	−	−	−	−	−	−
26a	+	+	+	?	+	+	?	+	?	?	?	?	?	+	+	+	+	+	+	+	−
26b	+	+	+	?	−	−	?	−	?	?	?	?	?	−	−	−	−	−	−	−	−
38a	+	?	+	−	−	−	−	−	−	−	−	−	−	−	−	−	?	−	−	−	−
38b	−	?	−	−	−	−	−	+	+	+	+	−	−	−	−	−	?	−	−	−	−
57a	+	+	+	+	+	+	+	+	+	+	+	+	+	+	+	?	?	+	+	?	−
57b	−	−	−	+	+	−	−	−	−	−	−	−	−	−	−	?	?	−	−	?	−
85a	−	?	?	?	−	+	?	+	+	+	+	+	+	+	+	?	+	+	+	+	−
89a	−	−	−	−	−	+	?	?	?	?	+	+	+	+	+	+	+	+	+	+	−
89b	−	−	−	−	−	−	?	?	?	?	−	+	+	+	−	−	−	−	−	−	−
90a	−	−	?	?	?	−	+	+	+	+	?	+	?	?	+	+	+	+	?	?	−
93a	+	+	+	+	+	+	?	+	+	+	+	?	?	?	+	?	+	+	+	+	−
93b	−	−	−	−	−	−	?	−	−	−	−	?	?	?	+	?	+	+	+	+	−
94a	−	−	−	−	−	?	−	?	−	−	?	−	−	−	+	+	+	+	?	+	−
95a	−	?	−	?	−	+	+	+	+	?	?	?	?	?	+	?	?	+	?	?	−
95b	+	?	+	?	−	−	−	−	−	?	?	?	?	?	−	?	?	−	?	?	−
95c	+	?	+	?	+	+	+	+	+	?	?	?	?	?	+	?	?	+	?	?	−
96a	−	−	−	−	?	+	+	+	?	+	+	+	+	?	+	+	+	+	+	+	−
107a	−	−	−	−	−	+	?	+	+	+	+	+	+	+	+	+	?	+	?	+	−
108a	−	−	?	−	−	−	+	+	+	+	+	+	+	+	+	+	+	+	+	+	−
108b	−	−	?	−	−	−	+	+	+	+	+	+	+	+	−	−	−	−	−	−	−
122a	−	?	?	−	−	−	−	−	−	−	−	−	−	−	+	+	+	+	+	−	−
125a	−	−	−	−	−	+	?	+	+	+	+	+	+	+	+	+	?	+	+	+	−
126a	−	?	?	?	−	+	+	+	+	+	?	?	+	+	+	+	?	+	?	+	−
129a	−	−	−	−	−	+	+	+	?	+	+	+	+	+	+	+	+	+	?	+	−
129b	−	−	−	−	−	−	−	−	?	−	−	−	−	−	+	+	−	−	?	−	−
129c	−	−	−	−	−	−	−	−	?	−	−	+	+	+	−	−	−	−	?	−	−
129d	−	−	−	−	−	−	−	−	?	−	−	+	+	+	−	−	−	−	?	−	−
135a	−	−	−	?	−	+	+	+	+	+	?	+	?	?	+	+	+	+	+	+	−
139a	−	−	?	+	+	−	−	−	−	−	?	−	−	−	?	?	−	−	−	−	−
139b	−	−	?	−	−	+	+	+	+	+	?	+	+	+	?	?	+	+	+	+	−
142a	−	+	+	?	?	?	−	?	?	−	?	−	−	−	?	−	?	−	?	−	−
142b	−	−	−	?	?	?	−	?	?	−	?	+	+	+	?	−	?	−	?	−	−
144a	+	+	+	+	+	+	?	+	+	?	+	?	+	+	+	+	+	+	+	?	−
144b	−	−	−	−	−	+	?	+	+	?	+	?	+	+	+	+	+	+	+	?	−
155a	−	−	−	−	−	+	+	+	+	+	+	+	+	+	+	+	?	+	?	+	−
162a	−	−	−	?	−	−	+	?	+	+	+	+	+	+	+	+	?	+	?	?	−
168a	−	−	−	−	−	−	d	+	?	+	+	?	+	?	+	+	+	+	+	+	−
169a	−	−	−	+	+	+	+	+	+	+	?	+	+	+	+	+	+	+	?	+	−
172a	−	−	−	+	+	−	−	−	−	?	?	−	−	−	−	−	?	−	?	?	−
172b	+	+	+	−	−	−	−	−	−	?	?	−	−	−	−	−	?	−	?	?	−
172c	−	−	−	−	−	+	+	+	+	?	?	+	+	+	+	+	?	+	?	?	−
172d	+	+	+	+	+	+	+	+	+	?	?	+	+	+	+	+	?	+	?	?	−
172e	+	+	+	+	+	+	+	+	+	?	?	+	+	+	+	+	?	+	?	?	−
182a	−	−	−	?	?	?	?	?	−	−	?	?	−	−	−	−	−	+	+	−	−
182b	+	+	+	?	?	?	?	?	+	+	?	?	+	+	+	+	+	+	+	+	−
194a	−	−	?	?	?	?	?	?	−	−	?	−	?	?	?	+	+	+	?	−	−
205a	−	−	?	+	+	+	+	?	+	?	?	?	+	?	+	?	?	+	?	+	−
206a	−	−	−	?	?	?	?	?	?	?	?	−	−	?	+	+	?	+	?	+	−

+, presence; −, absence; d, deletion; ?, missing data. Md, *Monodelphis domestica*; Dv, *Didelphis virginiana*; Mn, *Metachirus nudicaudatus*; Rr, *Rhyncholestes raphanurus*; Cf, *Caenolestes fuliginosus*; Dg, *Dromiciops gliroides*; Nt, *Notoryctes typhlops*; Pt, *Phascogale tapoatafa*; De, *Dasyurus geoffroii*; Sc, *Sminthopsis crassicaudata*; Mf, *Myrmecobius fasciatus*; Ml, *Macrotis lagotis*; Pg, *Perameles gunnii*; Io, *Isoodon obesulus*; Tr, *Tarsipes rostratus*; Pp, *Pseudocheirus peregrinus*; Tv, *Trichosurus vulpecula*; Mr, *Macropus robustus*; Pt, *Potorous tridactylus*; Vu, *Vombatus ursinus*; Hs, *Homo sapiens*.

Ten of the phylogenetically informative markers accumulated in the metatherian genome since their split from placental mammals, approximately 130 million years ago (MYA) [12],[13], and before the earliest divergence of the modern marsupial mammals, 70–80 MYA [3],[14]. All ten are absent in other mammals, significantly confirming the monophyly of marsupials (*p* = 2.0×10^−5^; [10 0 0] [15]).

The other 43 phylogenetically informative retroposon markers provide significant support for most of the basal splits within marsupials. The earliest marsupial divergence was previously impossible to resolve based on sequence data, which could not distinguish between Paucituberculata and Didelphimorphia as the sister group to Australidelphia [14],[16]–[19]. We identified two markers (MIR3_MarsA) in the South American shrew opossums (Paucituberculata) that were also present in all Australidelphia but absent in Didelphimorphia (Figure 2). Albeit not significant (*p* = 0.1111; [2 0 0]), this is the first molecular support for the earliest branching of Didelphimorphia, establishing it as the sister group to the remaining six marsupial orders. However, as significant support for this important marsupial branch requires three or more conflict-free markers [15], we attempted to find additional retroposons for the marsupial root. To find the third marker for the supported topology (Figure 2), a MIR3_Mars element present in kangaroo plus Paucituberculata but absent in opossum, we recovered ten additional loci from in silico screening; two contained the previously detected markers and eight contained new retroposons. Unfortunately, experimental verification showed that the absences of MIR3_Mars in opossum were due to non-specific deletions. On the other hand, we also did not find any loci with MIR3_Mars elements present in opossum plus Paucituberculata but absent in kangaroo, which would have supported the alternative of a close relationship between Didelphimorphia and Paucituberculata. We then screened for markers that would support the alternative hypothesis of Paucituberculata being the sister to all marsupials by performing an exhaustive in silico pre-screening for orthologous MIR3_Mars elements present in short introns of opossum and kangaroo. Starting from ∼6,000 potentially informative loci, we selected 39 highly conserved MIR3_Mars-containing introns. However, experimental verification showed that all of the elements were also present in the order Paucituberculata (*Rhyncholestes*), thus supporting the monophyly of marsupials (data not shown), but not the basal divergence.

Assuming that, in the entire genomes, there are more than just the two detected diagnostic insertions for the root, an expanded search including larger introns and conserved intergenic regions is required to find significant support for this branch. Such relaxed search conditions are expected to provide a huge number of additional markers spread over the entire marsupial tree, but will require extensive additional computational and experimental work.

Molecular estimates have placed the earliest divergences of Marsupialia in the Late Cretaceous, 65–85 MYA [3],[4],[14]. To resolve placental mammalian Cretaceous divergences [20], large amounts of sequence data were crucial to gain sufficient phylogenetic signal, which is a plausible explanation for the difficulties encountered in trying to resolve this branch in previous marsupial investigations [3],[4],[14]. However, morphological data have revealed several characters from the skull and postcranium, supporting Didelphimorphia as the sister to all marsupials [21], consistent with our two molecular markers.

Leaving the base of the tree for the time being, 13 of the original 53 markers were present in the South American Microbiotheria and the four Australasian orders but not in either Didelphimorphia or Paucituberculata, significantly supporting the monophyly of Australidelphia [1] (*p* = 6.3×10^−7^; [13 0 0]; Figure 2). The large number of phylogenetically informative markers indicates a long phylogenetic branch and/or a high degree of retroposon activity and fixation in the ancestral Australidelphia lineage. The branch separating Australidelphia from Didelphimorphia and Paucituberculata is one of the strongest supported and evolutionarily longest inter-ordinal branches in the marsupial tree [3],[4]. The fossil Australian marsupial *Djarthia murgonensis* is the oldest, well-accepted member of Australidelphia. Thus, combined with the lack of old Australidelphian fossils from South America, the most parsimonious explanation of the biogeography of Australidelphia is of an Australian origin [22]. However, the poor fossil record from South America, Antarctica, and Australia does not exclude that *Djarthia*, like *Dromiciops*, could be of South American origin and had a pan-Gondwanan distribution. Additional fossils from Australia or South America will shed more light on the early Australidelphian relationships and their biogeography.

Four markers significantly support the monophyletic grouping of the four Australasian orders to the exclusion of Microbiotheria (*p* = 0.0123; [4 0 0]; Figure 2). Several studies have presented evidence for the monophyly of the Australasian orders; these have typically been based solely on nuclear protein-coding genes such as *ApoB*, *BRCA1*, *IRBP*, *RAG1*, and *vWF* [4],[17],[19], albeit with relatively low support values. By contrast, other sequence-based studies, relying completely or partially on mitochondrial data, find the South American order Microbiotheria nested within the Australasian orders [3],[16],[23]. Thus, the two competing hypotheses, Microbiotheria nested within or outside Australasian orders, have confounded the search for a reliable marsupial phylogeny.

Two studies tried to combine the nuclear and mitochondrial data using different approaches to achieve a larger dataset with higher probability of resolving the marsupial phylogeny [14],[18]. Only R/Y-coding, removing of sites [18], or partitioning [14] reduced possible artefacts from the mitochondrial data enough to reach a topology consistent with the retroposon markers. However, both studies gave low support for the position of Microbiotheria, illustrating the difficulties in resolving a short branch using sequence data under difficult conditions, such as possible nucleotide composition bias problems and randomization of fast evolving sites. The support from two independent sources of phylogenetic information, our retroposon markers and nuclear genes [4],[17],[19], invalidates the mitochondrial results [3],[16],[23]. Complete mitochondrial genomes can give misleading signals, as was demonstrated for the incorrect position of Monotremata among mammals [24], and can even mislead phylogenetic reconstruction when mixed with nuclear data.

The position of Microbiotheria has been intensely debated since the cohort Australidelphia was first suggested based on tarsal evidence [1]. After decades of uncertainty derived from molecular and morphological data, we have uncovered four independent diagnostic retroposon insertions that finally place the South American order Microbiotheria at its correct place in the marsupial tree (Figure 2). Therefore, we propose the new name Euaustralidelphia (“true Australidelphia”) for the monophyletic grouping of the four Australasian orders Notoryctemorphia, Dasyuromorphia, Peramelemorphia, and Diprotodontia.

The relationship among the four Australasian orders is not resolved, and of special interest is the phylogenetic position of the marsupial mole, *Notoryctes typhlops*, which has been debated for a long time [3],[4],[14],[17]–[19],[21],[23]. The marsupial mole is the only burrowing marsupial and is found in the deserts of Australia. The eyes of the marsupial mole are vestigial and the fore- and hind limbs are morphologically derived due to the burrowing lifestyle. The derived morphology and the fact that the marsupial mole is the single species in the order Notoryctemorphia have complicated attempts to resolve its phylogenetic position relative to the other three Australian orders. Most analyses of molecular sequence data find the marsupial mole closely related to the orders Dasyuromorphia and Peramelemorphia, but the support values are generally weak [3],[4],[14],[17]–[19],[23], and the exact phylogenetic position relative to the other two orders is yet to be determined. During the retroposon screening one marker was found supporting a grouping of *Notoryctes*, Dasyuromorphia, and Peramelemorphia (*p* = 0.3333 [1 0 0]). The single retroposon marker is in agreement with the results from the sequence data. Extended screening of retroposons can provide additional evidence for the position of the marsupial mole among marsupials and which of the orders, Dasyuromorphia or Peramelemorphia, is the sister group.

Of the original 53 markers, 18 of them provide significant support for the monophyly of each of the five multi-species marsupial orders: five for Didelphimorphia (*p* = 0.0041; [5 0 0]), three each for Paucituberculata, Dasyuromorphia, and Diprotodontia (*p* = 0.037; [3 0 0]), and four for Peramelemorphia (*p* = 0.0123; [4 0 0]). Four of the remaining markers provide non-significant support for various intra-ordinal relationships of Diprotodontia (Figure 2). Two of them support the division between Vombatiformes (wombats and koala) and Phalangerida (kangaroos, possums) (*p* = 0.1111; [2 0 0]), challenging the results from mitochondrial sequence-based studies ([3], but see [25]), and one marker each supports the grouping of the possums *Tarsipes* and *Pseudocheirus* and that of the kangaroos *Macropus* and *Potorous* (*p* = 0.3333 [1 0 0]). One final marker supports the grouping of the *Didelphis* and *Metachirus*.

The outstanding advantage of using retroposon presence/absence data for phylogenetic reconstructions is the low probability of insertion homoplasy. Independent parallel insertions of identical elements or exact deletions are extremely rare [9], but nevertheless not completely negligible, especially after genome-wide in silico screening of rare informative loci. LINE1-mobilized elements, in particular, show a slight preference for a TTAAAA consensus insertion motif [26], but on the other hand, such elements are rare in the deep phylogenetic branches of marsupials (Figure 1; Figure S1). Excluding the more frequent *near* identical insertions or *unspecific* deletions requires careful aligning and interpretation of orthologous informative markers (see Materials and Methods and Dataset S1).

Another possible source of errors is incomplete lineage sorting (polymorphism during speciation) or ancestral hybridization that can affect any marker system. Particularly short internal branches of a tree (rapid speciation) and biased in silico pre-screening for potential phylogenetically informative loci are exposed to such effects [27].

The available genomes of the opossum and the kangaroo placed us in the advantageous situation of independently pre-screening two distant branches of the marsupial tree. All 53 experimentally verified markers confine a phylogenetic tree free of any marker conflicts. Fourteen of them were randomly inserted as a second marker in specific loci. For most internal branches we found significant support for the underlying prior hypothesis by three or more markers with a clear rejection of alternative hypotheses.

Given the limitations just mentioned, the retroposon marker system identified a clear separation between the South American and Australasian marsupials. Thus, the current findings support a simple paleobiogeographic hypothesis, indicating only a single effective migration from South America to Australia, which is remarkable given that South America, Antarctica, and Australia were connected in the South Gondwanan continent for a considerable time.

The search for diagnostic South American or Australidelphian marsupial morphological characters has been so far confounded by the lack of a resolved marsupial phylogeny [21],[22],[28]. The newly established marsupial tree can now be applied not only to morphological and paleontological studies but also to clearly distinguish genomic changes.

## Materials and Methods

### Taxon Sampling

The marsupial classification of Aplin and Archer [29] has been followed throughout the text. Representatives of all seven marsupial orders were included for retroposon screening. Except for the two single-species orders, at least two species per order were investigated. For all orders except Didelphimorphia, representative species were chosen to cover the deepest splits within each order. **Didelphimorphia:** *Monodelphis domestica* (gray short-tailed opossum), *Didelphis virginiana* (Virginia opossum), *Metachirus nudicaudatus* (brown four-eyed opossum). **Paucituberculata:** *Rhyncholestes raphanurus* (Chilean shrew opossum), *Caenolestes fuliginosus* (silky shrew opossum). **Microbiotheria:** *Dromiciops gliroides* (monito del monte). **Notoryctemorphia:** *Notoryctes typhlops* (marsupial mole). **Dasyuromorphia:** *Phascogale tapoatafa* (brush-tailed phascogale), *Dasyurus geoffroii* (western quoll), *Sminthopsis crassicaudata* (fat-tailed dunnart), *Myrmecobius fasciatus* (numbat). **Peramelemorphia:** *Macrotis lagotis* (bilby), *Perameles gunnii* (eastern barred bandicoot), *Isoodon obesulus* (southern brown bandicoot). **Diprotodontia:** *Tarsipes rostratus* (honey possum), *Pseudocheirus peregrinus* (common ringtail possum), *Trichosurus vulpecula* (common brushtail possum), *Macropus robustus* (wallaroo), *Potorous tridactylus* (long-nosed potoroo), *Vombatus ursinus* (common wombat).

### Transpositions in Transpositions (TinT)

The marsupial genome harbors about 500 different families of interspersed repeats [30]. Several retroposon families were active around and after the split of Australasian [31] and South American marsupials and potentially encrypt information about their phylogeny. For successful and focused experimental retroposon screening it is invaluable to have, a priori, a map of the ancestral retroposon activities. The previously developed TinT method [11] relies on a numeral compilation (Table S1) of nested transpositions (TinT) extracted from RepeatMasker coordinates and visualized after calculating their maximal activity probabilities. For experimental application, 24 subtypes of small SINE elements, active over the range of marsupial evolution, were pre-selected for the TinT analysis (Figure 1). The complete statistics of SINE elements in *M. domestica* and *M. eugenii* are given in Figure S2.

### In Silico Strategies

The assembled genome of *M. domestica* (MonDom5) and the draft genome of *M. eugenii* were used to pre-select potential phylogenetically informative intronic retroposon loci. Three different in silico high-throughput strategies, implemented in specially developed C-scripts, were applied to extract the genomic information.

- Short introns (∼1 kb) containing potential phylogenetically informative retroposed elements inserted in the lineage leading to opossum were extracted together with their conserved flanking exons. A total of 12,416 loci were computationally detected, and 113 were selected for their highly conserved (conserved in opossum, mouse, and human) exonic flanks suitable for generating applicable PCR primers for Zoo PCRs; six loci were phylogenetically informative in a reduced species sampling (*Monodelphis*, *Didelphis*, *Dasyurus*, *Perameles*, *Tarsipes*, *Pseudocheirus*, *Macropus*, *Vombatus*) and used for screening in the full taxon sampling.
- *M. domestica*–annotated unique exonic sequences (203,152) were blasted against *M. eugenii* trace sequences to derive exon-flanked intronic regions. Such introns were screened for lineage-specific retroposed elements (absent in *M. domestica*). We reconstructed 2,738 introns and selected 80 loci for their highly conserved flanking exons suitable for primer generation (conserved in opossum, kangaroo, and human); 22 were phylogenetically informative in a reduced species sampling (see strategy A) and chosen for full screening.
- *M. eugenii* sequences were screened for *M. domestica* orthologous introns from strategy A (containing opossum retroposed elements); 1,027 loci with additional kangaroo retroposed elements (not present in *M. domestica*) were selected for generating 35 highly conserved exonic primers (conserved in opossum, kangaroo, and human); four of these were phylogenetically informative in a reduced species sampling (see strategy A) and experimentally screened for in all selected marsupials.

The 228 loci extracted by these three strategies were experimentally analyzed in a small subset of eight representative marsupial species (see strategy A). The sequences from the experimental screening were aligned and carefully inspected for (1) identical genomic insertion points of retroposed elements, (2) identical element orientation, (3) identical element subtypes, (4) as far as available, concurrent element flanking repeats, (5) shared diagnostic indels, and (6) the consistency of insertion in representative species. The 32 selected loci mentioned above (in A–C) were determined to be phylogenetically informative (elements present at orthologous genomic locations in two or more species) and were screened in a larger taxon sampling comprised of 20 marsupials covering all seven orders (see taxon sampling). After sequencing, 53 phylogenetically informative retroposon markers were identified from the 32 introns. More than one informative marker was recovered in each of 15 of the introns, due to independent retroposon insertions (Table 1), and an additional 18 autapomorphic insertions were found.

### Experimental Work

Total DNA was extracted from tissues using the standard phenol-chloroform protocol [32]. Approximately 10–50 ng DNA was used in each 25 µl PCR amplification using ThermoPrime Taq (ABgene, Hamburg) with 1.5 mM MgCl_2_. All PCR reactions were prepared for high throughput in 96-well plates and the DNA was amplified using the touchdown PCR strategy, decreasing the annealing temperature stepwise by 1°C for the initial ten cycles, followed by 25 cycles at 45°C annealing temperature (for primers see Table S3). The initial screening was performed using eight representative marsupial species (see above) and PCR products were visualized on 1% agarose gels to detect presence/absence patterns via the size shifts of fragments. The PCR products indicating such size shifts were purified and ligated into the TA cloning vector pDrive (Qiagen, Hilden). Ligations were left overnight at 7°C and transformed into XL1-Blue competent cells. Colonies were PCR screened using standard M13 primers. For each positive PCR product, at least two colonies were sequenced. All sequence alignments were conducted using Se-Al [33]. Sequences were screened for retroposons using the RepeatMasker program (http://www.repeatmasker.org/RMDownload.html) and a specific retroposon library (available upon request).

### Cladistic Analysis of Retroposon Markers

From the markers in Table 1 we built a presence/absence (1/0) data matrix of retroposons (Figure S3). The strict consensus, most parsimonious tree was reconstructed using the irrev.up option of character transformation implemented in PAUP*4.0b10 [34] in a heuristic search performed using 1,000 random sequence addition and tree-bisection and reconnection (TBR) branch swapping. Because strictly marsupial-specific retroposons were investigated, the hypothetical human outgroup was coded 0. The resulting tree had a length of 53 and a consistency index of 1. The tree topology shown in Figure 2 refers to the derived parsimony tree. Due to the complexity and randomness of retroposon insertions, there are an extremely large number of possible unique character states (insertion sites), and maximum parsimony analyses converge to maximum likelihood estimators [35]. Evidence from retroposon markers is considered to be statistically significant when three or more markers are found supporting one node (i.e., when *p*<0.05) [15].

## Supporting Information

Dataset S1 Fasta alignments of all investigated phylogenetic informative loci.
(0.69 MB PDF)

Figure S1 Marker location and SINE retroposon subtypes. Marker location and SINE retroposon subtypes (red = L1-, green = RTE-, blue = L2-, black = L3-mobilized SINEs; see also Figure 1). The numbering of the elements corresponds to Table 1.
(3.18 MB JPG)

Figure S2 Compilation of genomic copies of SINE elements in opossum and kangaroo. Compilation of genomic copies of SINE elements in opossum and kangaroo (red = L1-, green = RTE-, blue = L2-, black = L3-mobilized SINEs; see also Figure 1).
(0.41 MB JPG)

Figure S3 Presence/absence data matrix and phylogenetic reconstruction. Presence/absence data matrix and phylogenetic reconstruction. (A) presence (1) and absence (0) matrix of orthologous SINE elements. Question marks denote missing data. (B) Strict consensus parsimonious tree from six equally parsimonious trees, using the irrev.up option of character transformation (PAUP* 4.0b10), heuristic search (1,000 random sequence addition), and TBR branch swapping. Human was used as outgroup. Treelength: 53; Consistency Index = 1.
(0.83 MB DOC)

Table S1 TinT matrices. (a) Transpositions in transpositions in opossum. (b) Transpositions in transpositions in kangaroo.
(0.29 MB DOC)

Table S2 The accession numbers for the established marsupial sequences.
(0.06 MB DOC)

Table S3 The primers used for amplification of single copy marsupial introns containing retroposed elements. The primers used for amplification of single copy marsupial introns containing retroposed elements. The location of each marker on the chromosomes (Chr.) in the *Monodelphis* genome is listed.
(0.07 MB DOC)
