# Supplementary material for: Tracking Marsupial Evolution Using Archaic Genomic Retroposon Insertions
Source: PLoS Biol. 2010 Jul 27;8(7):e1000436. doi: 10.1371/journal.pbio.1000436 (PMC2910653; doi:10.1371/journal.pbio.1000436)
Supplement: Dataset S1 — Fasta alignments of all investigated phylogenetic informative loci. (0.69 MB PDF) [file pbio.1000436.s001.pdf]

Supplementary Information

Data Set S1. Fasta alignments of all investigated phylogenetic informative loci.

Tracking marsupial evolution using archaic genomic retroposon insertions.

Maria A. Nilsson, Gennady Churakov, Mirjam Sommer, Ngoc Van Tran, Anja Zemann, Juergen Brosius, and Juergen Schmitz

Data set S1.
Fasta alignments of all investigated phylogenetic informative loci.

Abbreviation	Scientific name
Mdo	*Monodelphis domestica*
Dvi	*Didelphis virginiana*
Mnu	*Metachirus nudicaudatus*
Rra	*Rhyncholestes raphanurus*
Cfu	*Caenolestes fuliginosus*
Dgl	*Dromiciops gliroides*
Nty	*Notoryctes typhlops*
Pta	*Phascogale tapatoafa*
Dgo	*Dasyurus geoffroii*
Scr	*Sminthopsis crassicaudata*
Mfa	*Myrmecobious fasciatus*
Bil	*Macrotis lagotis*
Pgu	*Perameles gunnii*
Iso	*Isoodon obesulus*
Tro	*Tarsipes rostratus*
Ppe	*Pseudocheirus peregrinus*
Tvu	*Trichosurus vulpecula*
Mro	*Macropus robustus*
Ptr	*Potorous tridactylus*
Vur	*Vombatus ursinus*

MARKER 08

```
>MdoRep1
------------------------------------------------------------
------------------------------------------------------------
------AATGAATGAATGAATAAAGCATTTATTAAGCACTTACTATGTGCAAAGCACTGT
GCTAAGCGCTGGGGATACAAATAGAAAAGTAAGACAGTCCCTGCTCTCAAGGAGCTCACA
TTCTAATGGGGGAGACAACACATATGGAAGGTTTCAG--CTGCAAGTCAGA-TGGAAAGG
TCCCATG-GTCCTTA-GGGTGCAGCGGCAAAGC-AGATGGTAATGCCTCTTCTTTAATGT
CATTTCCACTGATAAAATCA-----TATCAGTTTCT-GATG-TTGAACCATTTGACAGTG
CC---AAGGACTTTGGTGGCAAGAACTTTCTTTTCTGGGTCTTCAGTAGCTGTGGCTG--
------------------------------------------------------------
------------------------------------------------------------
------------------------------------------------------------
------------------------------------------------------------
--------------------
>Mdo
TGGCCCAGCAGCTCCTCCTCC---TTCAGCATTAGGCTATG--GTGAGTGACT-GGGGTT
GCAAAATCT-------TTTTTTCCTTACCTCCTTTCCGTGTCCCTC--------------
------AAGGAATATATGAAAAA-GCATTTATTAATCTCTTACTGTGTCCAAAATATAAA
TCTGGGTGGGGGGAGG----ATAGAAAGT--------------------AGGAGTTCC--
-TCTAGTGG-CAATACAATATATGTAGGGGGTTTCAA-TTTTCAAATTAGA-TGAGAAGA
T-GTAGC-CTCTTTA-AGATGCAGTGGTAAAAC-AGGTGATCATGCATC---TTTAGTGA
CATTTCCATTAATTAAACCA-----TACCAGCTTTA-TATA-TGGAACTAGTGGACACTG
CC---AAGTACTTTGCCATTGAGAACTTTGTTTTCTAGGTCCTTGGTAGATATGACTG--
-ATATAAACTATAAGGTTCTATGATGGGTTCCTTAACACTGA------------------
TTCCCTGTGTGATGGGATAATTATAGTC-----------------------ACCTCTGCT
G-TGTGGATAGAGAAACAACATGTTAAATAAGCAGG-AACAGGGATTAGGGTAGTAACTT
ATCCCTTTCTGTTTCTCAG--GGCCACC---TACATCAATGGCTTCAGCTTCAGGAAGTT
TTCCAGGCTCCAATCTATAT
>Dvi
TGGCCCAGCGGCTCCTCCTCC---TTCAGCATTAGGCTATG--GTGAGTGGCT-GGGGTT
ACAAAATCT-------TTTTTTCCTTCCCTT-----------------------------
------AAGGAATAGATGAAAAA-GCATTTATTAACCTCTTACTGAGTCCAAAGTATAAA
TCTGGGTGGGGGGAGG----ATAGAAAAG--------------------AGGAGTTCC--
-TCTAGTGGGCAATACAGCACATATAGGGGGTTTCAA-TTTTCAAGTTAGA-TTAGGT--
--GTAGC-CTCTTTA-CGATGCAGTGGTAAAAC-AGGTGATCATGTATC---TTTTGTGA
TGTTTCCATTAATTAAACCA-----TACCAGCTTTA-TTTA-TGAAACCTTTGGATACTG
TC---AAGTACATTGCCATTGAGAACTTAGTTTTCCAGGTCCTTGGTAGATATGACTG--
-ATATAAGATATAAGGTTTTATGATGGGTTTCTTAACTCTAC------------------
TTCCCTGTGTGATGTGATAATTACAATC-----------------------ACCTCTGAT
G-TGTGAATAGAGAGTCAACTTGTTAAATAAGCAGG-AACAGGGACTAGGGTAGTAATTT
ATCCCTTTCTGTTTCTCAG--GGCCACC---TACATCAATGGCTTCAGCTTCAGGAAGTT
TTCCAGGCTCCAATCTATAT
>Mnu
TGGCCCAGCGGCTCCTCCTCC---TTCAGCATTAGGCTATG--GTGAGTGGCT-GGGGTT
ACAAAATCT-------TTTTTTCCTTCCCTT-----------------------------
------AAGGAATAGATGAAAAA-GCATTTATTAACCTCTTACTGAGTCCAAAGTATAAA
CCTGGGTGGGGGGAGG----ATATAAAAG--------------------AGGAGTTCC--
-TCTAGTGGGCAATACAGCACATATAGGGGGTTTCAA-TTTTCAAGTTAGA-TTAGGT--
--GTAGC-CTCTTTA-CGATGCAGTGGTAAAAC-AGGTGATCATGTATC---TTTTGTGA
TGTTTCCATTAATTAAACCA-----TACCAGCTTTA-TGTA-TGAAACCATTGGATACTG
TC---AAGTACTTTGCCATTGAGAACTTAGTTTTCCAGGTCCTTGGTAGATATGACTG--
```

-ATATAAGATATAAGGTTTTATGATGGGTTTCTTAACTCTAC------------------
TTCCCTGTGTGATGTGATAATTACAATC-----------------------ACCTCTGCT
G-TGTGAATAGAGAGTCAACTTGTTAAATAAGCAGG-AACAGGGACTAGGGTAGTAATTT
ATCCCTTTCTGTTTCTCAG--GGCCACC---TACATCAATGGCTTCAGCTTCAGGAAGTT
TTCCAGGCTCCAATCTATAT
>Cfu
CGGC---GCAGCCCCTCCTCC---TTCAGGGTTAGGCTATG--GTGAGTGGCC-AGAGTT
GCAATGGCT-------TTGTTTCCTTCTCTTCTCTCCCTGTCACCT--------------
-------AGAAAT-GAGAAAAGT-------------------------------------
---AGTAAATCC--
-TCTACTGGGTGATATA-TATGGGGAGGGTGTTTCAG-TTTTC-AGTTTAGATGAAAAGT
C-ATAGA-ATCTTTA-GTGTGCAGTGGAAAAGC-AAGTGGTCATTCATC---TTTAGTTT
CATTTCCATTAATTAAACTG-----TACCAGTTTTTTATA-TGGAACCATTGGATACTT
CCAACAAGTACTTTGCTATTGAGAACTTTGTTTTACGGGTCCTTGATAGGTATAGCTG--
-CTAAAAAATATAAGGTTCTTTGATAGGTTCCCTAACTCGGC------------------
TTCCCTATGCCTCTGGATATCAGAAGAT----GGGGTAGATAATTACAGTCACCTCTGCT
G-TGGAGATAGAGAAGTGGTTTGTTAAATCAGCAGG-AATAGTGTAGTAACTTATATCTT
A------TCTGTTCTTCAG--GGCCACC---TACATCAATGGCTTCAGCCTCAGGAAGTT
TTCCAGGCTCCAATCTATAT
>Dgl
TGGCCCGGCTGCCCCTCCACC---TTCAGGGTTAGGCTATG--GTGAGTGGCT-AGGGGT
ACAAAGGCT-------TTGTTTCCTTCCCTTCTTTCCCTGTCCCCT--------------
------AAGGAAGGGAGGAAAGA--CATTTACTAAGC---------------------AA
AACCTTGGGACTAGAA----ATAGAAAAGT-------------------AGGACCTCC--
-TCTAAAGGGTGGTCCAACACATGGAGGGGGTTTCTATTTTTCAAGTTGGACGGGGAAGG
C-ATAGC-ATCTTTG-GTATGCAATGCCAAAGCCAGTCGATCATGCATC---TTTAGTGA
CATTGCCATGAATTAAATCA-----TGCGGGCTTTT-TAAA-TGGAACCATTGGACATTG
CT---GAGCACTTTGCCATTGAGAGTTTTGTTTTCCAGGTCTTTGGTTGCTGTGGTTG--
-ATTTAAGCTATAAGGTTCTGTGATGGGTTCCCTAACTTTGC------------------
TTCCCTGTTTCCCTTGGTATCAAATGAT--------GGGATGACAGTCACCTCTCCTG-T
G-TGG--ATGGAGAAGCAGCTTGTTAAATAAGCAGGAATAGGGA-CCAGGGTAGTAACTT
TTTTTCTTTTGTTCCTTAG--GGCCACCACCTACATCAGTGGCTTCAGCCTCAGGAAATT
T-CCAGGCTCC-AGTCATAT
>Nty
TGCCCCTCCTCCCCCTCCTCC---TTCAGGATTAGGCTATG--GTGAGTTACT-GGGGGT
GCAAAGATT-------TTGTTTCTTTCCCTTCTCTCCCTGTCCCTC--------------
------AAGGAATGGAGGAAA---
--CCTGGGGAGTATGG--
------------------TAC--ATAGTGGGTTTCAGTTTTCC-AGTTAGA-TCAGAAGG
C-ACACC-ATCTTTA-GGGTGCAATGGCAAAGC-AGGTGATCATGCATC---TTTACTGA
CATTTCCATTCATTAAACCA-----TACCAGCTTAT-TCTC-TGGAACCATTGGACATTG
CC---------------ATTGAGAGTTTTGTTTTCCAGGCCTTTGGTAGCT-AGGATG--
-ATAGAAACTTTTAGGTTCTGTGATGGGTTCCCT-ACTCTATGCC---------------
TTCCCTGTGCCTCTGGGAATCAAAAAAT--------GGGATGATTACAGTCACCTTTGCT
G-TGTGGATAGAGAAGCAACTTGTTAAATAAGCAGCAATA-GTGACTAGGGTAATAACTC
GTGCTCTTTTGTTTTTTAG--GGCCTCCACCTACATCAATGGCTTCAGCCTCAGGAAATT
TTCCAGGCTCCAATCTGTAT
>Dgo
TGGCCCGGCTGCTGCTCCTCCTCCTTCAGGGTTAGGCTATG--GTGAGTGGC--------
-------------------------------------CCATCCTCC--------------
------AAGGAATGGAGAAAAGA-ACATTTATTAAGTTTTTACTCTGTCTAAAGCATATA
AACCTTGGGGGTATAA----GTAGAAAAGT-------------------AGGAGTTCC--
-TCTAATGGATGAGACACTATATGTAGAGGTTTTCAG-TTTGCAAGTTGAA-TGATAAGG

C-ACAAC-ATCTTTAAGGGTGCCGTGGCAAAGC-AGGTAATCTTGTGTC---TTTAGTAA
CAATTGCATTA-TTAAACCA-----CACCAGCTTTT-TATG-TGGCACTACTGGGCACCG
CC---AAATACTTTTCTGTTGAGAGTTTTGTTTTCAGGGTCTTTGGTAGTTATGACTG--
-ATAAAAACTATAAGGTTCTGTGATGATATCC-TAACTCTGC------------------
TTCCCTGTACTTCTTGGTATCAAAAAAT--------GGGAGAATTATAGTCACCTCTGTT
G-GGTGGATAGATAAACAACTTGTAAAAT------------------ATGGTAATAACTT
A-TCCTTTCTGTTCTTCAG--GCCCACCACCTACATCAATGGCTTCAGCCTCAGGAAATT
TTCCAGGCTCCAGTCTCTAT
>Mfa
WGGCCCGGCTGCTCCTCCTCC---TTCAGGGTTAGGTTATG--GTGAGTGGCTGGGGGAG
GGGGGAGGTGCCAAGACTATTTCCTTCCATTCTTTCCCCATCCTCC--------------
------AAGGAATGGAGAAAAGA-ACATTTATTAAGTTCTTACTATGTCTAAAGCATATA
AACCTGGGGAGGTATA---AATAGAAAAGG-------------------AGGAGTTCC--
-TCTAATGGATGATACATTATATGTAGAGGG-TTCAG-TTTTCAAGTTGGA-TGGTAAGG
C-ACAGGCATCTTTAAGGGTGCA-GGGCAAAGC-AGGTAATCACGTGTC---TTTAGTGA
CAATTCCATTA-TCAAACCA-----TACCAGCTTTT-TATG-TGAAATTACTGGGCATTG
CC---AAATACTTTTCTGTTGAAAGTTTTGTTTTCAGGGTCTTTGGTAGCTATGACTG--
-ATAAAAGCTATAAGGTTCTGTGATGGTTCTCTTACGCCCCCCCCCCCCCCCAACTCTGC
TTCCCTGTACCTCTTGGTATCAAAAAAT--------GGGAGAATTATAGTTACCTCTGCT
TGGGTGGATAGAGAAACAACTTGTAAAAT------------------ATGGTAATATCAT
ATTCTCTTCTGTTCTTTAG--GCCCACCACCTACATCAATGGCTTCAGCCTCAGGAAATT
TTCCAGGCTCCAGTCTCTAT
>Bil
TGGCCCACCTGCCCCTCCTGC---TTCAGGGTTGACCTATG--GTGAGTGC---------
---------------TTTTTCTCCTT-CTCTCTCTCCCTGTCCCCC--------------
------AGGGACAC-AG----------------------TT-TTGTGTCCAAAACATATT
----TTGAGAATATAA----ATAGAAAAGT-------------------CGAAGCTTC--
-TCTAATGGGAG-------------------TTTGC--TTTTCAAATTGGA-TGGGAAGG
CAAAAGC-ATCTTGA-GGGTGCAATGGTAAAGC-AGGTAATCATACGTC---TTTAATGG
CATTCCCATCAGTTAAATGA-----TACTAGCTTTTTGAAAGTGGAACCATTGGGCACAC
TC---AAGCATTTTGTCATTGAGAGTTTTGTTTTCC----------------AAACTG--
-ATAAAAGCTATAAGATGCTCAGATGGGCTCCTTAACTCTGC------------------
-TTCCTCTGCCTCTTACTACTCAAAAAT--------GAAATAATTGCAGGCTCCTCTGCT
G-TGCAGATAGAGAATCAAATTATTAAATAAGCAGA-AGTAGAGACTGGTGTAGTAACTT
ATCTTCTTCTATTCTTCAG--GGCCACCACCTACATCAATGGCTTCAGCCTCAGGAAATT
TTCCAGGCTCCAATCTATAT
>Tro
WGGCTCGGCTGCCCCTCCTCC---TTCAGGATTAGGCTACG--GTAAGTACTTGGGGGGG
ACAAAGAT-------TTTGTGTCCTTGT----TCTC-YCCTGGCCCC-------------
------AAAGGGAGGAGGAAAGA-ACATTTATGAAGTTCTT---GTGTCTAAGGC---TT
AACCTTGGGGATTTCA----GTAGAAGAAT-------------------AGGCGCTCT--
-TCTAATGGGTGATACAGCTAATGTAGGAGCTTTCAG---------TTGTA-GGGGAAGG
C-ACAGC-ATCTTTAAGG-TTCAGTGGGAGAGT-AGGTGGTCATGGATC---TCTAGTGC
CATTTCCATTAATTARACTA-----TGCC---------------------------ACTG
CC---AGGCCCTTTGCCCTTGAGCACTTTGTTTTCTGGCTCTTTGGTAGATTTAACCA--
-TTATAAGCTAGAAGGTTCTTTTGATGGATCCCTAAC--AAC------------------
TTTCCTGTGCCTCTTGGTTTTAAAAAAAAAAAAAAAGGCAGGAATAAAGTCACCTTTGCT
G-TGTGGAYAGAGAAGTAGCTTGT--AATAAGCATGAATTAGGGGCTAGGGTAATCACTT
GTCCTCTTCTCTTCTTTAG--GGCCACCACCTACATCAATGGCTTCAGCCTCCGGAGGTT
TTCAAGGCTCCAATCTATAT
>Ppe
MGGCTCAGGTGCCCCTCCTCC---TTCAGGGCTAGGCTATG--GTAAGTGCCT-GGGGGT
ACAAAGAT-------TTTGTTTCCTTCCTTTCTCTCCCTGTCCCCA--------------

```
------AAGGAATGGAGGAAAGA-ACATTTATTAAGTTCTTACTGTGTCCAAAGC---TT
AACCTTGGGGGCATCA----ATAGAAAAGT-------------------AGGAGCTCC--
-TCTAATGGGTGATACAACACATGTAGGAGGTTTGAGTTTTTG-AGTCAGA-TGGAAAGG
C-ATAGC-ATCTTTA-GGCTTCAGTGACAAAGC-AGGTGATCATGGATC---TTTAGTGA
CATTTCCATTAATTTAATTAAACTATGCCAACTTTT-TATA-TAGAACCACTGGATGCTG
CC---AAGCACTTTGCCATTGAGATTTTTGTTTTCCTGGTCTTTGGTAGCTATA-CTG--
-ATATAAGCTATAAGGTTCTTTGATGGGTTCCCTAACTCTAC------------------
TTTCCTTTGCTTCTTGATATTAAAAAAAA----GGGGGAGTAATTAAAGTCACTTTTGCG
G-TATGGATAGAGAAGCACCTTGT--AATAAGCAGGAATTAGGGACTAGGGTAATAACTT
ATCCTCTTCTGTTCTTTAG--GGCCACCACCTACATCAATGGCTTCAGCCTCAGGAAGTT
TTCAAGGCTCCAGTCTATAT
>Tvu
AGGCCCGGCTGCCCCTCTTCC---TTCAGGGTTAGGCTACG--GTGAGTGCCT-GGGGGT
GCAAAGAC-------TTTGTTTGCTTTCTTTCTTTGCCTGTCCCCC--------------
------AAGGAATGGAGGAAAGA-ACATTTATTAAGCCTTTGCTGTGTCCAAAGCATAAA
--CCTTGGGAGTGTAA----ATGGGAAAGT-------------------ATGTGCTCC--
-TCTAATGGGTGATACAGCACATATAGGGGGTTTCAGTTTTTG-AGTCAGA-TGGGAAGG
C-ACAGC-ATCTTTA-GG-TGCAACGGCAAAGC-AGATGATCATGCATT---TTTAGTGA
CATTTCCATTAATTATACCA--------GTTTTTTTTAATA-TGGAACCACTGGACACTG
CC---AAATGCTTTGCCATTGAGAGTTTTGTTTTCCAGGTCTTTGGTAGCTATAGCTG--
-ATATAAGCTAGAAGGTTCTGTGATGGGTTCCTTAACTCTAC------------------
TTTTCTGTGCCTCTTAATACCAAAAAAT--------GGGATAATTACAGTCACCTCTGCT
T-TGTGGATAGAGAAGCAGCTTATTAAATAAGCAGGAATTAGGGACTGGGGCAATAACTT
ATCTGCTTCTGTTCTTCAG--GGCCACCACCTACATCAGTGGCTTCAGCTTCAGGAAGTT
TTCAAGGCTCCAGTCTATAT
>Mro
TGGCCCAGCTGCCCCTCCTCC---TTCAGGGTTAGGCTATG--GTGAGTGCCT-GGGGGT
GCAAAGAC-------TGTATTTCCTTCTTTTTTCTCCCTGTCCCCC--------------
------AAGAGATGGAGGAAAGA-ACATTTATTAAGCTCTTACTGTGTCCAAAGTGTAAA
--CCTTGATCCTATAA----ATGGAAAAGT-------------------AGGAGCTCC--
-TCTAATGGATGATAAAGCACAAGTAAG---TTTCAGTTTTAC-AGTCAGA-TGAGAAGG
C-ACAGC-ATCTTTA-GGGTGCAATGGCAAAGC-AGGTG-TCATGCACC---TTTAACGA
CATTTCCATTAATTAAACTG-----TACTAGCTTTT-TATA-TGGAACCACTGGACACTG
CC---AAGCACTTTGCCATTGAGAGTTTTGTTTTCCAGGTCTTTGGTAGCTATGGTTG--
-ATATAAGCTAGAAGGTTCTGTGATGGGTTCCTTAACTCTAC------------------
TTTCCTTTGCCTCTTGATATCAAAAAAAAT------AGGATAATTATAGTCATCTGTGCT
G-TGTGGCTAGAAAAGCAGCTTGTTAAATAAGCAGGAATTAGG-------GTAGTAACTT
ACCCTCTTTTGTTTTTCAG--GGCCTCCACCTACATCAATGGCTTCAGCTTCAGGAAGTT
TTCAAGGCTCCAATCTATAT
>Ptr
TGGCCCAGCTGCCCCTTCTCC---TTCAGGGTTAGGCTATG--GTGAGTGCCT-GGGCGT
GCAAAGAC------TTTTATTTCCTTTTCT---CTCCCTGTCCCCC--------------
------AAGGGATGGAGGAAAGA-ACATTTATTAAGCTCTTACTGTGTCCGAAGTGTAAA
--CCTTGATCCTATAA----ATAGAAAAGT-------------------AGGAGCTCC--
-TCTAATGGATGATAAAGCACAAGTAGG---TTTCAGTTTTGC-AGTCAGA-TAAGAAGG
C-ACAGC-ATCTCTA-GGGTGCAATGGCAAAGT-AGGTA-TCATGCACC---TTTAATGA
CATTTCCATTAATTAAACTG-----TACCGGCTTTT-TATA-TGGAACCACTGGACATTG
CC---AAGCACTTTGCCATTAAGAGTTTTGTTTTCCAGGTCTTTGGTAGCTGTGACTG--
-ATATAAGCTAGAAGGTTCTGTGATGGGTTCCTTAACTCTAC------------------
TTTCCTGTGCCTCTTGATATCAAAAAAY--------GGGATAATTATAGTCACCTGTGCT
G-TGTGGCTAGAAAAGCAGCTTGTTAAATAAGCAGGAATTAGGAATTAGGGTAGTAACTT
AYCCTCTTTTGTTTTTCAG--GGCCTCCACCTACATCAATGGCTTCAGCCTCAGGAAGTT
TTCAAGGCTCCAATCTATAT
```

```
>Vur
TGGCCCGGCTGGCCCTCCTCC---TTCAGGATTAGGCTATG--GTGAGTGCCT-AGGGGT
GCAAAGAC------TTTTGTTTCCTTCCCTTCTCTTCCTGTCCCCC--------------
------AAGGAGTGGAGGAAAGA-ACATTTATTAAGCTCTTACTGTGTCCAAAGCATAAA
--CCTTGGAGGTATAA----ATAGAAAAGT-------------------AGGAGTTCC--
-TCTAATGGGTG---------------------------TTTTTG-AGTTAGA-TGGGAAGG
C-ACAGC-ATCTTTG-GGGTGCAATGGCGAAGC-AGGTGATCATGTGTC---TTTAGTGA
CATTTCCATTAATTAAGCTA-----TACAGACTTTT-TATA-TGGAACCACTGGATGCTA
CC---AAGCACTTTGCCATGGAG---TTTTTTTTCTAGGTCTTTGGTAGCTATGGCTG--
-ATACAAGCTGTAAGGTTCTGTAATGGATTCCTTAACTCTAC------------------
TTTCCTGTGCCTCTTGGTATCAAAAAAT--------GGGATAATTACAGCCACCTCTGCT
A-TGTGGATAGAGAAACAGCTTGTTAAATAAGCAGGAATTAGGAACTAGGGTAGTAACTT
ATCCTCTTCTGTTCTTCAG--GGCTACCACCTACATCAATGGCTTCAGCCTCAGGAAGTT
TTCAAGGCTCCAATCTATAT
>Human
-CGGTGCTGTGGCCCCAGCCCC--TCCTTCTTCAGGGCTGG--GCTTTGGTGA-GTGGCT
GTGAACAC----------AGGAATGTTACT--GTCCCCTGTTCAGAGGCATCAGACTCTG
GGG---------------------------------------------------------
------------------------------------------------------------
------------------------------------------------------------
------------------------------------------------------------
------------------------------------------------------------
------------------------------------------------------------
-TTATA--CCTGAA---TCTCTTTTATTTCCCTTGTA-----------------------
TTTCCTGTGCCTCTGAGCCTCAGAAGA--------TGGGACAGTCATATTTGCCCCTCTT
G-TGTGGAAAGGGAAGTGATATACCGAACAAGGAGGCAGAATG-ACTGGGCTTTTGACTC
TACCTTTATTCTACTTCTC--AGGC-CCACCAACATCGCTGGCTTCAGCCTCAGGAAGTT
TCCCTAACTCTGGTCTGTAT
```

MARKER 14

```
>MIRc
------------------------------------------------------------
------------------------------------------------------------
------------------------------------------------------------
------------------------------------------------------------
------------------------------------------------------------
------------------------------------------------------------
------------------------------------------------------------
------------------------------------------------------------
------------------------------------------------------------
------------------------------------------------------------
------------------------------------------------------------
------------------------------------------------------------
------------------------------------------------------------
------------------------------------------------------------
------------------------------------------------------------
------------------------------------------------------------
------------------------------------------------------------
------------------------------------------------------------
-------------------------------------GGGGATAATAATA---CCTGCCC
TGCCTACCTCACAGGGTTGTTGTGAGGA--------------------------------
------------------------------------------------------------
------------------------------------------------------------
--------------------TCAA---ATGAGAT--------------------------
------------------------------------------------------------
------------------------------------------------------------
---------------------AATGTAT--------------------------------
----GTG-AAAGCGCTTTG---TAAACTGTAAA---------------------------
-----------------------------------------------
>WSINE1
------------------------------------------------------------
------------------------------------------------------------
------------------------------------------------------------
------------------------------------------------------------
------------------------------------------------------------
------------------------------------------------------------
------------------------------------------------------------
------------------------------------------------------------
------------------------------------------------------------
------------------------------------------------------------
------------------------------------------------------------
------------------------------------------------------------
------------------------------------------------------------
------------------------------------------------------------
------------------------------------------------------------
------------------------------------------------------------
------------------------------------------------------------
------------------------------------------------------------
------------------------------------------------------------
------------------------------------------------------------
------------------------------------------------------------
```

```
------------------------------------------------------------
-------------------------------------GAGGGGCAGCTAGGTGGTGCAGT
GGAT-AGAGCACCAGCCCTGGAGTCAGGAGGACCTGAGTTCAAATCCAGCCTCAGACACT
TA---------CTAGCTGTGTGACCCTGGGCAAGTCACTTAACCCCAATTGCCTC-----
----AAAAAAAAAAAAAA------------------------------------------
------------------------------------------------------------
-----------------------------------------------
>WSINE1
------------------------------------------------------------
------------------------------------------------------------
------------------------------------------------------------
------------------------------------------------------------
------------------------------------------------------------
------------------------------------------------------------
------------------------------------------------------------
------------------------------------------------------------
------------------------------------------------------------
------------------------------------------------------------
------------------------------------------------------------
------------------------------------------------------------
------------------------------------------------------------
------------------------------------------------------------
------------------------------------------------------------
------------------------------------------------------------
------------------------------------------------------------
------------------------------------------------------------
------------------------------------------------------------
--------------------------------------------------GAGGGGCAGC
TAGGTGGTGCAGTGGAT-AGAGCACCAGCCCTGGAGTCAGGAGGACCTGAGTTCAAATCC
AGCCTCAGACACTTA----CTAGCTGTGTGACCCTGGGCAAGTCACTTAACCCCAATTGC
CTCAAAAAAAAAA---AAAA----------------------------------------
------------------------------------------------------------
------------------------------------------------------------
------------------------------------------------------------
------------------------------------------------------------
-----------------------------------------------
>retropseuodgene
------------------------------------------------------------
------------------------------------------------------------
------------------------------------------------------------
------------------------------------------------------------
------------------------------------------------------------
------------------------------------------------------------
------------------------------------------------------------
------------------------------------------------------------
------------------------------------------------------------
------------------------------------------------------------
------------------------------------------------------------
------------------------------------------------------------
------------------------------------------------------------
------------------------------------------------------------
------------------------------------------------------------
-------------------CCTTCTTTACCCTGGAAACTACCTGACCCCATCTTTATGCA
```

```
TTTAAACCATCTTCCTCTTTGAAAGTTCTTGACCTTTGTCAAGTTTCTCAAAATAGAAAC
CT----------------------------------------------------------
------------------------------------------------------------
------------------------------------------------------------
------------------------------------------------------------
------------------------------------------------------------
------------------------------------------------------------
------------------------------------------------------------
------------------------------------------------------------
------------------------------------------------------------
------------------------------------------------------------
-----------------------------------------------
>L1_Mdo3a_3end
------------------------------------------------------------
------------------------------------------------------------
------------------------------------------------------------
------------------------------------------------------------
------------------------------------------------------------
------------------------------------------------------------
------------------------------------------------------------
---------------------------------------------TTTTTTTTTTTTTTA
A---AATTTTATTTAATTAATTAATTTAGAATATTTTTCCATGGTTACAAGAATCATGTT
CTTTCCCTCCCCTCCCCCCACCCCCCTCCCATAGCCGACGCGCAATTCCACTGGGTTTTA
CATGTGTCATTGATCAAGACCTATTTCCATATTATTGATATTTGCACTAGGGTGATCATT
TAGAGTCTACATCCCCAATCATATCCCCATCGACCCATGTGATCAAGCAGTTGTTTTTCT
TCTGT-------------------------------------------------------
------------------------------------------------------------
------------------------------------------------------------
------------------------------------------------------------
------------------------------------------------------------
------------------------------------------------------------
------------------------------------------------------------
------------------------------------------------------------
------------------------------------------------------------
------------------------------------------------------------
------------------------------------------------------------
------------------------------------------------------------
------------------------------------------------------------
------------------------------------------------------------
------------------------------------------------------------
-----------------------------------------------
>WSINE1
------------------------------------------------------------
--------------------------------------------TTTTTTTTTTTTTT--
------------GAGGCAATTGGGGTTAAGTGACTTGCCCAGGGTCACACAGCTAG----
----TAAGTGTCTGAGGCTGGATTTGAACTCAGGTCCTCCTGACTCCAGGGCTGGTGCTC
TATCCACTGCACCACCTAGCTGCCCCTC--------------------------------
------------------------------------------------------------
------------------------------------------------------------
------------------------------------------------------------
------------------------------------------------------------
------------------------------------------------------------
------------------------------------------------------------
```


>Mdo
TCTCTTATACCAGTGTCCGCAGTAACAGCTCTTATCTGGGCAGTGATGAGATGGGATCTG
--GTAAGTAGGTTGGGAC--

-------------------------------------CA--TTTTC---AAGATGA----
-------------------CTTGGGAGACACTAAGGAACGATCTGGTGTTCG-TCCAGAT
ATGTGTAACATTGTCAGGTGGAA--TGGTGGCTTTGG---GGTGGAAGATGAGTAGTGTT
CCAGGTAAAGACAATAGAATGA--ACAGAGATCCAGAGAGTGAACTCATCTTGAATATTT
----AATTCTATTTAATCAATTAATTTAGAATATTTTTCCATGGTTACATGA-TTATGTT
CTTTCCCTCC--TTCCCCCACCCCCCTCCCATAGCCGATGCACAATTCCACTGTGTTTTA
CATGTGTCATTAATCAATACCTATTTCCATATTATTGATATTTGCACAAGGGTGATCGTT
TAAAGTCTACCTCCCCAATCATATCCCCATAAAACCATGTGATGAAGCAGTTGGTTTTCT
TCTGTTGAATTCATCTTCAGAGTACAGTCTATAGGATAGA--------------------
----TTGATGACAGAGTATAAAAGGGAAATTAATGGGAGATGGGTTTGGGAATGTAGGGG
TTAGTCAAATTGTGGTGAGCTTT-GGCAAGACAAACAAAGTAAATAAGACTTTATTCTG-
CAAA--

-----TAGTAGGGA---ACCATCAAAGG---TTTTGTAGCCGGG-AATACCATGATCAAA
TCCATATTT---------TAAAGAGATGATGA--------GATGATGTTTGT-CCTGCCC
-----ACTTCATAGTGTTGTTTTTTAAAAAAA----------------------------

-------------------GTCAA---ATAAGAT--------------------------

---------------------ATTGGAT--------------------------------
----GGG-AAAGGACCATG-----------------AGAGATAGAAAGTACTACACAGAT
GTGAACTTCCTCACTTGTTCCCTCTCCCAG--GAGATGAGTTACCTT
>Dvi
TGTCTTATACCAGTGTCCGAAGTAACAGCTCTTATCTGGGCAGTGATGAGATGGGATCTG
--GTAAGTAGGATTGGAC--

-------------------------------------TA--TTTTC---AAGATGA----
-------------------CTGGGGAGACCCTAAGGAATGATCTGGTGTCCA-TCAATAC

```
ATGTGTAACATTGTCAGGTGGAA--TGGTGGCTTTGG---TGTGGAAGATGAGTAGTGTT
CCAGGTAAAGACAAGAGAATGA--ACAGAGGTCCAGAGAGTGAACTCATCTTCA------
------------------------------------------------------------
------------------------------------------------------------
------------------------------------------------------------
------------------------------------------------------------
-------------------GAGAATGCTCTATAGGATAGA--------------------
----CTGATGACAGAGTATAAAAGGGAAAATAATGGGAGATGAGTTTGGGAATGTAGGGG
TTAGTCAAATTGTGGTGAGCTTT-GGTAAGACGAGCCAAGTAGCTAAGACTTTATTCTG-
CAAA--------------------------------------------------------
------------------------------------------------------------
-----TAGTAAGGA---ATCGGTGAAGG---TTTTGCAGCTGGG-AATACCATGATCAAA
TCCATATTT---------TAAAGACATGATGA--------GATGATGAGA---CTTGCCC
TGTCTACTTCCTAGGTTTTTTTTTTTTTTTTTTTTA------------------------
------------------------------------------------------------
------------------------------------------------------------
-------------------GTCAA---ATGAGAT--------------------------
------------------------------------------------------------
------------------------------------------------------------
---------------------TTTGGAT--------------------------------
----GGGAAAAGG-----------------------AGAGATAGAAAGTACTACACAGAT
GTGAATTTCCTCACTTTTTCTCTCCCTTAG--GAGATGAGTTACCTT
>Mnu
TGTCTTATACCAGTGTCCGAAGTAACAGCTCTTATCTGGGCAGTGATGAGNTGGGATCTG
--GTAAGTAGGATTGGAC------------------------------------------
------------------------------------------------------------
------------------------------------------------------------
-------------------------------------TA--TTTTC---AAGATGA----
-------------------CTGGGGAGACCCTAAGGAATGATCTGGTGTCCA-TCAATAC
ATGTGTAACATTGTCAGGTGGAA--TGGTGGCTTTGG---TGTGGAAGATGAGTAGTGTT
CCAGGTAAAGACAAGAGAATGA--ACAGAGGTCCAGAGAGTGAACTCATCTTCA------
------------------------------------------------------------
------------------------------------------------------------
------------------------------------------------------------
------------------------------------------------------------
-------------------GAGAATGCTCTATAGGATAGA--------------------
----CTGATGACAGAGTATAAAAGGGAAAATAATGGGAGATGAGTTTGGGAATGTAGGGG
TTAGTCAAATTGTGGTGAGCTTT-GGTAAGACGAGCCAAGTAGCTAAGACTTTATTCTG-
CAAA--------------------------------------------------------
------------------------------------------------------------
-----TAGTAAGGA---ATCGGTGAAGG---TTTTGCAGCTGGG-AATACCATGATCAAA
TCCATATTT---------TAAAGGCATGATGA--------GATGATGAGG---CTTGCCC
TGTCTGCTTTCTAGTTTTTTTTTAA-----------------------------------
------------------------------------------------------------
------------------------------------------------------------
-------------------GTCAA---ATGAGAT--------------------------
------------------------------------------------------------
------------------------------------------------------------
---------------------TTTGGAT--------------------------------
----GGGAAAAGGACCATG-----------------AGAGATAGAAAGTACTACACAGAT
GTGAATTTCCTCACTTTTTCTCTCCCCTAG--GAGATGAGTTACCTT
>Rra
TCTCCTACACCAGTGTCCGAAGTAACAGCTCTTACCTGGGCAGTGATGAGATGGGGTCTG
```

--GTAAGTAGGAGAAGGT--

-------------------------------------CTCTTTTTC---AAGATGA----
------AAAGGAGAGGATACTAGGGAGACATTTAGGAAACATCTTATATATG-TCCAACT
TTATGTAGTATTACTGGACGGAA--TGGGTCCTTTGA---CTGGCAAGGCAAGTAGCATT
CTAGGTGAGGACAGTAGTATGAATAGAGAAGCCCAGAGGGAGAACTCATTTGCA------

-------------------GGGAAAAGTTTATAGTGTAGT--------------------
----TTGATGAGAGAGT------------------GGAGGTATGGCTGGAAAGGTAAGGG
ATGGTCTATTTGTGGAGAATTTT-GACAAGACAAGCTAAGGAACT--AACTTTGTTCAT-
GAAG--

-----TAGTAGGGG---GTCTTTAAAAG---TTTTTAAACGCAG-CGTAACTTGGTCA--
-CCTTATTT---------TAAGGAGACAATA---------GGGAATGATAATACTTGCTC
TGTCTACTTCATAGGGTTGTTGTAAAAACAAA----------------------------

--------------------TCAG---ATGAGAT--------------------------

---------------------ACTGGTT--------------------------------
----GGGAAA-GGACTATG-----------------AGAGCTAGAAAGCACTCTACAAAT
GTGAGATTATACTCTTTTTCTCTCTCCCAG--GAGATGAATTGCCCT
>Nty
TTTCCTACACCAGTGTCCGTAGTAACAGCTCTTACCTGGGCAGTGATGAAATGGGATCTG
--GTAAGTAGAATAGGGC--

-------------------------------------CA--TTTTC---AAAATGA----
------AAACAAGAGGGCCTTAGGGAGAC----AGGAAAGATATGGTGCACA-TCCATAA
ATGTGTAGCATTGTCAAGTGGAA--GGGGGTCTTTGG---TGGGAAGGATGAATAGCATT
CCAGGCAGAAACAATAGTATGA--ACAGAGGCCCAGAGGGTAGACTGTTTTTCA------

-------------------GGAAACAGTTAA---TGTAGA--------------------
----TTGATGAGAGAATATGCAAGAGAAATTAA-GGGAGATAAAGTTGCGAAGATAGGAC
ACAGTCAGATGGTGAAGAACCTT-GGCAAGACAAGCTAAGGAGCTAAGGCTTTATTCAG-
TTAA--

-----TAGTAGGGG---GCCATTGAAGG--TTTTCTATTATTTTC-----CATAATCAGA
TTCATATTT---------TAAAGATATGACAG--------GAGA-TGATAATTCTTACCC
TGCCTATGTCATAGGATTCTTGTTAAAAAAAA-------AAAGTCAG----GGGGGCAGC
TAGATAGTGCAGTGGAT-AGAGCACTGACCTTGGACTTAGGAGGACCTGAGTTCAAATCT
AGCCTCAGACATTTGATACTTAGCTGTGTGGCCTTAGGCAAGTCACTTAACCCCAATTGC
CTAGGGGAAAAAA---AAAGTCAG---ATGAGAT--------------------------

---------------------ATTGGCT--------------------------------
----GAGAAAGGACCAGAA---------------------GTAGAGAGTACTACCTGGAA
GTGAACTTTCTCATTCGTTCTTTCTCCCAG--GAGATGAGTTGCCTT
>Pta
TTTCCTACACCAGTGTCCGTAGTAACAGCTCTTACCTGGGTAGTGATGAGATGGGATCTG
--GTAAGTAGGATAGGGC--

-------------------------------------CA--TTTTC---AGGATGA---A
AAAAAAAAAAAAGAGGATGCTAGCAAGACAGTGAGGAAAGATGTGGTATACA-TCCATAT
ATGTGTAGTATTGTCATATGGAA--GGAGGTCTTTGG---TGAAGAG-------AATGTT
CCAGGCAGAGACAATAGTGTGA--AC---TGCCCAGAGGGTGGACTGATTATCA------

-------------------GGGAACAGTT-------------------------------
---GGG
GGGT-------GTGGT-AAGAAA-ATATTAGCAAGCTAAGGAGC-----------TCAGA
CTTA---TAGTAGGGA---CTTTCTTTATCCTGGAAACTACCTGATCCCATCTTTATGTA
TTTAAACCATCTTCCTCTTTGAAAGTCCTTGACCTTTGTCAAGCTTCTCAAAATAGAAAT
CT---TAGTAGGGC---GCTATTGAAGG--TTTTGAAAGTGGGGA--TA---TCATCAGA
TCTTTATTT---------TAAAGAGATGACA---------GAGGATGATAATCCTTGCTC
TGCCAA----CAAGGATTCTTGTTTAAAAAAAA---------------------------

-------------------ATCGG---ATGAGAT--------------------------

---------------------GTTGACT--------------------------------
----GGGAAA-AGACTACC-----------------------------GAAAGTGTGAAC
TTTCATGTACTTCTTTGTTCTCTCTCCCAG--GAGATGAGTTACCTT
>Dgo
TTTCCTACACCAGTGTCCGTAGTAACAGCTCTTACCTGGGCAGTGATGAGATGGGATCTG
--GTAAGTAGGACAGGGC--

-------------------------------------CA--TTTTC---AGGATGA----
----AAAAAAAAGAGGATGCTAGCAAGACAGTGAGGAAAGATCTGGTATACT-TCCATAT
ATGTGTAGCATTGTCAGATGGAA--GGAGGTCTTTGG---TGCAGAGAATGAGTAATGTT
CCAGGCAGAGACAATAGTGTAA--AC---TGCCCAGAGGGTGCACTGATTTTCA------

-------------------GGGAACAGTTAAAGGTATAGA--------------------
----CTG---AGAGAGTTTGCAAGGAAA-TTAATGGGGGATAAAGTT-------------
GGGTGGTGGTGGTGGT-AAGAAA-ATATTGGCAAGCTAAGGAGC-----------TCAGA
CTCC---TAGTAGGGA---CCGTCTTTACTCTGGAAATTACCTGATCCCATCTTTATGTA
TTTAAACCATCTTCCTCTTTGAAAGTCCTTGACCTTTGTCAAGCTTCTCAAAATAGAAAC
TT---TAGTAGGGA---G-----------------------------TA---TCATCAGA
TCTTTATTT---------TAAAGAAATGACA---------GAGGATGATAATCCTTGCTC
TGCCAA----CAAGGATTCTTGTTTAAAAAAA----------------------------

```
------------------------------------------------------------
------------------------------------------------------------
-------------------ATCAG---ATGAGAT--------------------------
------------------------------------------------------------
------------------------------------------------------------
---------------------GCTGACT--------------------------------
----GGGAAA-AGACTACG------------------------------GAAAGTGTGAAC
TTTCATGTATTTCTTTGTTCTCTCCCAG--GAGATGAGTTACCTT
>Scr
TTTCCTACACCAGTGTCCGCAGTAACAGCTCTTACCTGGGCAGTGATGAGATGGGATCTG
--GTAAGTAGGATAGAGC------------------------------------------
------------------------------------------------------------
------------------------------------------------------------
-------------------------------------CA--TTTTC---AGGATGAAAAA
AAAAAAAAAAAAGAGGATGCTAGCAAGACAGTGAAGAAAGATCTGGTATACA-TCCATAT
ATGTGCAGCATTGTCAGATGGAA--GGGGGTCTTTGG---TGCGGAGAATGAGTAATGTT
CCAGGTAGAGACACTAGTGTGA--AC---TGCCCAGAGGGTGGACTGATTTTCA------
------------------------------------------------------------
------------------------------------------------------------
------------------------------------------------------------
------------------------------------------------------------
-------------------TGGGACAGTTAATGGTATAGA--------------------
----TTG---AAAAAGTATGCAAGGGAAATTAATTGAGA-TAAAATT-------GGCGGG
GGGTGGTGGTGGTGGT-AAGAAA-ATGTTGGCAAACTAAGGAGC-----------TCAGA
CTCC---TAGTAGGGA---CCTTCTTTACCCTGGAAACTACCTGACCCCATCTTTATGCA
TTTAAACCATCTTCCTCTTTGAAAGTTCTTGACCTTTGTCAAGTTTCTCAAAATAGAAAC
CT---TACTAGGGA---GCTATTGAGGG---TTTGTAAGTGGGGAGTAA----CACCAGA
TCTATATTT---------TAAAAAGATGACA---------GAGGATGATAATCCTTGCTC
TGCCAA----CAAGGATTCTTGTTTTTTAAAAAAAA------------------------
------------------------------------------------------------
------------------------------------------------------------
-------------------ATCAG---ATGAGAT--------------------------
------------------------------------------------------------
------------------------------------------------------------
---------------------TTTGACT--------------------------------
----GGGAAA-AGACCACG------------------------------GAAAGGGTGAAC
TTTCATGTACTTCTTTGTTCTCTCCCAG--GAGATGAGTTACCTT
>Mfa
TTTCCTACACCAGTGTCCGTAGTAACAGTTCTTACCTGGGCAGTGATGAGATGGGATCTG
--GTAAGTAGGATAGGGC------------------------------------------
------------------------------------------------------------
------------------------------------------------------------
-------------------------------------CA--TTTTC---AGGATGA----
---AAAAAAAAAGAGGATGCTAGCAAGACAGTGAGGAAAGATCTGGTATATG-TCTGTAT
ATGTGCAGCATTGTCAGGTGGAA--GGGGATCTTTAG---TACGAAGAATGAGTGATGTT
CCAGGCAGGGACAATACGATGA--AC---CTCCCAGAGGGTGGACTAATTTTTA------
------------------------------------------------------------
------------------------------------------------------------
------------------------------------------------------------
------------------------------------------------------------
-------------------GGGAACAGTTAATGGTGTANA--------------------
----TTG---ATAGAGTATGCAAGGGAAATTAATGGAAAATAAAGTT-------------
------GGAGGGTGGT-AAGAAA-ATGTTGGCAAGCTAAGGAGC-----------TCANA
```

CTCC---TAGTAGGGA---CCTTCTTTACCCTGGAAATCACCTGACCCCATCTTTATGCA
TTTAAATCATCTTCCTCTTTGAAAGTCCTTAACCTTTGTCAAGCTTCTCAAAATAGAAAC
CT---TAGTAGGGA---GCTATTTATTGAAGTTATTAGGTGGAAAGTAA-TATGATCAGA
TCCATATTT---------TAAAGAGATGACA---------GAGGATGATAATCCTTGCTC
TGCCTA----CAAGGATTCTTGTTAAAGAATAAATA------------------------

-------------------ACCAA---AAAAAAC--------------------------

---------------------CAGGANT---TCTNGTTTTTTTTTTTTTTTTTTTTTTTA
AAGTCAGATG-AGATGCTG------------GCTTGGAAAAGACCATGGAAAGTGTGAAC
TTTCATGTA---CTTTGTTCTCTCTCCCAG--GAGATGAGTTACCTT
>Bil
TTTCCTACACCAGTGTCCGTAGTAATAGCTCTTACCTGGGCAGTGATGAGATGGGATCTG
--GTAAGTAGAATAAGGC--

-------------------------------------CA--TTTTC---AAGATGA----
------AAAGGAGAAGATGCTAGGGAGACATTTAGGAAATATTAGGTGGGTA-TTCATAT
ATGGGTAGCATTGCCTGGTAGAA--GGGGCTCTTCTTAGATGGAGAGGACAAGTGGCATT
CTAGGCAGAGAAAATTGTAAGA--ACAGA--GGCAGAGGATAAACTTATTTTCA------

-------------------GAGAACAGTTAGTAGTATAGA--------------------
----TTAATGAGAGCATGTGCAAGAGAAATTAATGGGAGATAAAATTGGGAAGGTAGAAG
ACATTTAAACAGTGAATATTCTT-GAAAAGACAAGGTAAAGAGCTAAGACCTTATTCAG-
AAAA--

-----TAGTAGGGA---GTCATTGAAGA--TTTTGCAGGTGGGGAATGA-CATAATCAGA
TCCATATTT---------TAAAGAGATGAAAA--------GA-GTTGACA------GTGC
TGCCCACTTCATAGGATTCTTGTTAAA---------------------------------

-------------------GTCAA---TTGAGAT--------------------------

---------------------ACTGGCT--------------------------------
----GGTTAA-GGACAATG-----------AGAAGTAAAAAGCACTACACCAGTGTGGCT
T-------ACTCACTTGTTCTCCCTGGCAG--GGGATGAATTGCCTT
>Pgu
CTTCCTACACCAGTGTTCGTAGTAACAGCTCTTACCTGGGCAGTGATGAGATGGGATCTG
--GTAAGTAGGATAGGGC--

-------------------------------------CA--TTTTC---AAGATGA----
------AAAGGAGAAGATGCCAGGGAGACATTTAGGAAATATTAGGTGTACA-TTCATAT
ATAGGTAGCATTGTCATGTAGAAGGGGGGGC---CTTAGATGGGGAGGATAAGTGGTATT
CTAGGCAGAGAAAATAGTATCA--ATAGA--GGCAGAGAATAGACTCATTTT--------


```
------------------------------------------------------------
------------------------------------------------------------
------------------------CAGCTAATAGTATAGA--------------------
----TTAATGAAAGAATATGCAAGGGAAATTAATGAGAGATAAAATT-------GGGAAG
GTACAAGACATTTAAAGATTCTT-GGCAAGATAAGCTTAAGAGCTAAGACTTTATTCAG-
AAAA--------------------------------------------------------
------------------------------------------------------------
-----TAATAGGGA---GTCATTGAAGA--TTTTGCAGATGGGGAATGA-CATCATCAGA
TCCATGTCT---------TAAGGAGATGAAAA--------GAGA-TGACA------ATGC
TGCCTACTTCATAGGATTCTTGTTAAAAA-------------------------------
------------------------------------------------------------
------------------------------------------------------------
-------------------GTTAG---ATGAGATA-------------------------
------------------------------------------------------------
------------------------------------------------------------
---------------------ACTAGCT--------------------------------
----GGTTAA-GGACAATG-----------AGAAATAGAAAGCAGTACACCAATGTGGTT
T-------ACTCATTTGTTCTCCCTGGCAG--GGGATGAATTGCCTT
>Iso
TTTCCTACACCAGTGTTCGTAGTAACAGCTCTTACCTGGGCAGTGATGAGATGGGATCTG
--GTAAGTAGGATAGAGC------------------------------------------
------------------------------------------------------------
------------------------------------------------------------
-------------------------------------CA--TTTTC---ATGATGA----
------AAAGGAGAAGATGCCAGGGAGACATTTAGGAAATATTAGGTGTACA-TTCATGT
ATAGATAGCATTGTCATGTAGAA--GGGGCC---CTTAGATGGGGAGGATAAGTGGTATT
CTAGGCAGAGAAAATAGTATCA--ATAGA--GGCAGAGACTAGACTCGTTTTCA------
------------------------------------------------------------
------------------------------------------------------------
------------------------------------------------------------
------------------------------------------------------------
-------------------GAGAACAGCTAATAGTATAGA--------------------
----TTAATGAAAGAATATGCAAGGGAAATTAATGAGAGATAAAATTGGGAAGGTACGAG
ACA-------TTTAAAGATTCTT-GGCAAGATAAGCTTAAGAGCTAAGATTTTATTCAG-
AAAA--------------------------------------------------------
------------------------------------------------------------
-----TAATAGGGA---GTCATTGAAGA--TTTTGCAGATGGGGAATGA-CATCATCAGA
TCCATATCT---------TAAGGAGATGAAAA--------GAGA-TGACA------ATGC
TGCCTACTTCATAGGATTCTTGTTAAAAA-------------------------------
------------------------------------------------------------
------------------------------------------------------------
-------------------GTTAG---ATGAG---------GGCGGCTAGGTGGCGTAGT
GGATAAGAGCACCGGCCCTGGAGTCAGGACTTCCTGAGTTCAAATCCGGCCTCAGATACT
TACTAAT-TACCTAACTGTGTGGCCTTGGGCAAGCCACTTAACCCCA-TTGCCTTGCAAA
ACCTTAAAAAAAAAAAAA---GTTAGCT--------------------------------
----GGTTAA-GGACAATG-----------AGAAATAGAAAACAGTACACCAATGTGGTT
T-------ACTCATTTGTTCTCCCTGGCAG--GGGATGAATTGCCTT
>Ppe
TTTCCTACACCAGTGTCCGTAGTAACAGCTCTTACCTGGGCAGTGATGAGATGGGNTCTG
--GTAAGTAGGACAAAAC---CATTTTC--TTTTTTCTTCTTTTTCTTTTTTCTTTTTGG
AGAGGGGAAGGTAGGGCAGTTGTGGTTAAGTGACTTGCCCAAGGTCACAGGGGTAGTTTT
GTGTTAAGTGTG--AGGCTGGATTTGAACTCAGGTCCTCCTGACTCCAGGGCTGGTGCTC
TATTCAGTGAGCCACCTAGCCACCCCAACAGGAC---CA--TTTTC---AAGATGA----
```

------AAAGGGGAGGACACTAGGGAGACTTTTAGGAAAGATCTGGTGTACATTACATAT
ATATGTAACATTGTCAGGTGGAA--GGGGGTCTTCAG---TGGGGAGGATGAGCAGGATT
CCAGGCAGAGACAGTAGTGTGA--ACAGAGGCCCAGAACGTGGACTAGTTTTCA------

-------------------GGAAACAGTTAATAGTATAGATTGATGAGAGAGTATGCAAG
CAAATTAATGGGAGATAAAGCT-------------------------GGCAAGATAGGAG
GCAGTCAAATAGTGGAGAACCTT-GGCAAGACAAGCTAAGGAGCTAAGACTTTATTCGG-
TAAA--

-----TAGTAGGGA---GCCACTGAAGA--TTTTGCAGATGGTGAGTAA-CATGATCAAA
TTCATATTT---------TGAAGAGATGACAA--------GAAA-TGATGATGTTTG---
------TTTCATAGAATTCTTGTTTTAAAAAAAAT-------------------------

-------------------GTCAG---ATGAGAT--------------------------

---------------------ACTGGCT--------------------------------
---TGTGAAA
T------TACTCACTTGTTCTCTCTTCTAG--GAGATGAGTTGCCTT
>Mro
TTTCCTACACCAGTGTCCGTAGTAACAGCTCTTACTTGGGCAGTGATGAGATGGGATCTG
--GTAAGTAGGATAGGGC--

-------------------------------------CA--TTTTC---AAGATGA----
------AAGGGAGAGGATGCTAGGCAGACATTTAGGGAAGATCTGGTGTACA-TCCTTTT
CTGTGTAGCATTGTCAGGTGGAA--GAGGGTCTTTGG---TGGGGAGGATGAGCATCATT
TCAGGCAGAGACAGTAGTATGA--GCAGAGGCCCAGAAGGTGGACTAATTTTCA------

-------------------GGGAACAGTTAATAGTATAGA--------------------
----TTGATGAGAGAGTAAGCAAGGAAA-TTAATAGGAGATAAAATTGGGAAGGTAGAAG
AAAGTCAAATGGTGGAGAACCTT-GGCAAGACAAGCTAAGGAGCTAAG-CT--ATTCAG-
TAAA--

-----TAGTAGGGT---GCTGCTAAAGA--TTTTGTAGGTGGGGAGTAA-CATGATCAGA
TCCATATTT---------TAAAGAGATGACTG--------GAGG-TGACAATGCTTGGCC
TGCCTACTTCTCAGGATTCTTGTT-AAAAAAAAAT-------------------------

-------------------GCCAG---ATGAGAT--------------------------

---------------------ACTGGCT--------------------------------
----GGGAAA-GGACTGTG-----------AGAAGTAGAAGGCACTACACAGATGTGCAC
T------GACTCACTTGTTCTCTCTCCCAG--GAGATGAGTTACCTT
>Ptr

TTTCCTACACCAGTGTCCGTAGTAACAGCTCTTACTTGGGCAGTGATGAGATGGGATCTG
--GTAAGTAGGATAGGGC--

-------------------------------------CA--TTTTC---AAGATGA----
------AAGGGAGAGGATGCTAGGCAGACATTTAGGAAAGATCTGGTGTACA-TCCTTTT
ATGTGTAGCATTGTCAGGTGGAA--GGAGGTCTTTGG---TGGGGAGGATGAGCGGCATT
TCAGGCAGAGACAGTAGTGTGA--GCAGAGGCCCAGAAGGTGGACTGATTTTCA------

-------------------GGGAACAGTTAATAGTATAGA--------------------
----TTGATGAGAGAGTATGCAAGGAAA-TTGATAGGAGATAAAGTTGGGAAGGTAGAAG
ACAGTCAAATGGTGGAGAACCTT-GGCAAGACAAGCTAAGGAGCCAAG-CT--ATTCAA-
TAAA--

-----TTGTAGGGT---GCTGCTAAAGA--TTTTGCAGGTGGGGAGTAA-CATGATCAGA
TCCATATTT---------TAAAGAGATGACTG--------GAGG-TGACAATGCTTGCCC
TGCCTACTTCACAAGATTCTTGT--TAAAAAAAAT-------------------------

-------------------GCCAG---ATGAGAT--------------------------

---------------------ACTGGCT--------------------------------
----GGGAAG-GGAC--TG-----------AGAAGCAGAAGGCACTAC-CAGATGTGAAC
T----------TACTTGTTCTCTCTCCCAG--GAGATGAGTTGCCTT
>Human

-----------------CAGACTCCACATCCCTTGTGGGTTTGGAGTT--CTGTAGCTGT
ATCCCCCT---------CTTAGGAAAGTGCTGGGGATGG---------------------

------------------------------TGTGAGGTGGGGCAGGAATGACTGATGG-C
T--------GCTGCTTCTACCTCCTGCCAG-GAGATGAGCTGCCCT

MARKER 20

>Mar1c_Mdo

--GTTCAG-T---CGTTCAGTC
GTGTCCGACTCTTCGTGACCCCGTGGACCATAGCACGCCAATACTGTCCATGGGGTTTTC
TTGGCAAAGATACTGGAGTGGTTTGCCATTTCCTTCTCCAG---------TGGAT-----
--TAAGGCAAACAGAGGTTAAGTGACTTGCCCAGGGTCACACAGCTAGTAAGTGTCTGAG
GCCGGATTTGAACTCAGGTCTTCCTGACTCCAGGCCCGGCGCTCTATCCACTGAGCCACC
TAGCTGCCTCC---

>Mdo
ATGACAAAGGATACAGCAATAGA--GTAATACGTTGCTT---AATTCAGGGAGTGTA-CC
-ATAGAATATCTAATGTAGAAACTTCATTTCAG---AAAATTGCAGAT---TGTTTAGTT
GTGTTTGATTCTTTGTAACTCCATGGACCATAGC----------TATCCATGGGGTAGTC
TTGGCAGAAATACTGGAATGATTTGCCATTTTCTTCTCCAGCAGATTCACTGGATCCTTT
TATCAGCCAATTAAAGGATAAGTGCTTTGCCCAGAGTTGTGTAGCTAGGTTGTATCTGAG
GGCAGATCTAAAGTCAGATCTTCCTGACTTTGGTCCTAGCACTCTACCCAATGAACCA--
TAACTGTCTCT---AAAATTGCAGAA------ACTTT-AACTTT--AAGATAAAATAAAT
GCTTAATTGTATGACCATAAGTCATTAAATATAAGCACTTAAAA-------TATTTTTAC
TATGATTTTACTCCATGTCTTTTGGAGCTAATTTATATATTAAAGTTATCTCCATAGGGC
AAGAAGGT-GGGAGAGAA-GATTC-TGTGCTGTGTGTTTCATTTATAA-TGATTC-----

----------------------TTTTGTTTTCCTTTTTTAATTG--GTTTTACTATTTTT
-AAAGTTTTCTAT-----TGATAAGCATAGAAA----TGTACTTATATTACTGTTTGTTA
TTTTAATTGCAGATCCAATATAATGTATGTTGAATTGTACCAATAGTGTTCTCCTTTTTT
-AAAAG
>Dvi
ATGACNAAGGATACAGCAATCGA--GTAATACAT--TTT----ATTCAAGAAGTGCA-CC
-ATANAATATCTAATGTAGAAACTTCATTTCAT---AAAATTGCAAAT---TGTTCAGTT
GTGTCTGACTC--TGTGACTCCATGGACCATGGC----------TATCCATGGGATAAAC
-TGGCAAAAATACTAGAATGATTTGCCATTTCCTTTTCCAGCAGATTCACTGGATTCTTT
TGTCAACCAGTTAAAGGTTAAGTGCCTTGCCAAGAGCTTTGTATCTAGGTTGTATCTGAG
GGCAAATCTGAAGTCTGGTCTTCCTGACTNTAGTCNTAGTTCTCTACCCACTCAACCA--
TAGCTGTCTNT---AAAATTGCAGAAAAA---ACTTT-AATGNT--AAGATAAAATAAAT
GCTTAAGGGTATGACCATAANTTATTAAAGATAAGCATTTAAAA-------TATTTTTAC
TATGATTTTACTCCATGTCTTTTGGAGCTAATATATNTATTGAAGTTATCTCCATAGGGC
AAGATGGT-GGGAGAGAA-GATTC--GTGCTGTATGTTTAATTTATAAATTATTC-----

----------------------TTTTGTTTAATTTTT--AATTA--GTTTTACTATTTTT
-AAAGTTTTCTAT-----TGATAAGTGTTGAAA----TGTAATTATATTACTGTTTGTTA
TTTTGATTGTAGATCCTATATAATGTATGTTGAATTGTACCAATAGTGTTNNCANNNNNT
T-AAAT
>Mnu
ATGACAAAGGATACAGCAATCGA--GTAATACAT--CTT----ATTCAGGAAGTGCA-CC
-ATAGAATATCTAATGTAGAAACTTCATTTCAT---AAAATTGCAAAT---TGTTCAGTT

GTGTCTGACTC--TGTGACTCCATGGACCATGGC----------TATCCATGGGATAAAC
-TGGCAAAAATACTAGAATGATTTGCCATTTCCTTTTCCAGCAGATTCACTGGATTCTTT
TGTCAACCAGTTAAAGGTTAAGTGCCTTGCCAAGAGCTTTGTAGCTAGGTTGTATCTGAG
GGCAAATCTGAAGTCTGGTCTTCTTGACTCTAGTCCTAGTACTCTACCCACTCAACCA--
TAGCTGTCTCT---AAAATTGCAGAAAAA---ACTTT-AATGTT--AAGATAAAATAAAT
GCTTAAGGGTATGACCGTAAGTTATTAAAGATAAGCATTTAAAA-------TATTTTTAC
TATGATTTTACTCCATGTCTTTTGGAGCTAATATATGTATTAAAGTTATCTCCATAGGGC
AAGATGGT-GGGAGAGAA-GATTC--GTGCTGTATGTTTAATTTATAAATTATTC-----

----------------------TTTTGTTTCATTTTT--AATTA--GTTTTACTATTTTT
-AAAGTTTTCTAT-----TGATAAGTGTTGAAT----TGTAATTATATTACTGTTTGTTA
TTTTGATTGTAGATCCTATATAATGTATGTTGAATTGTACCAATAGTGTTCTCCTTTTTT
TAAAAG
>Cfu
ATGACAAAGGATACAGCAATCGA--GTAATGCATTGCTT---AATTCAAGAAGTTCGTCG
-AAGGAATATCTAATATAAAAACTTCATCTCAT---AATATTGCAAAC------------

--------------------------------ACTTT-AAACTTT-AAGATAAAATAGAT
GCTTAACTCTATGGCTGTATGTCATAAAAGATAAGCATTTTACT----------------
--CTGTTTTACTCTGTGTCTTTGGGGGCTTATCTATATCCTAAAATTGTCCCCATAGGAC
AAGAGTAT-GGGG-AAAA-TATTC-TGTGCCACATATTTCATTTATGA-TTATTA-----

----------------------CTTTGTTTCCTTTTA--AATTA--GTTTTACTATTTTT
-AAAGTTTTCTATGTTGTTGAAATGGGTAAAA-----TATGACTATATTACGATTTGCTA
TTTTAATTGCAGAGCCTATATAATGTATATTGACTTGTACCAATAGTTTTCTCCTTTTTT
-AAAAG
>Dgl
ATGACAAAGGATACAGCAATAGA--GTAATACATTGCTT---AATTCAGGAAGTATA-CT
ACTGGAGTATCTAATGTAGAAACATCATCTCAT---AAAATTGCAGAT------------

--------------------------------ACTTT-AAACTTT-AAGATAAAATAAAT
GTTTAACTCTGTGACT--AAGTCATCAAAGATAAACATTTAAAA----TAC-TCTTTTAC
TATCATTTTACTCCATGTCTTTTGG----------------AAAATTATCTCCATATGAC
TAGAACTT-GGGAGAAAA-GATTC-TGTGCTATGTATTTCATTTATAA-TGATTA-----

----------------------TTTTGTTTCCTTTTT--AATTG--GTTTTACTATTTTT
---AGTTTTCTATTTTATTGAAAAGGGTAGAAA----TGTAATTGTATTACCATTTGCTA
TTTTGATGGCAGATTCTATATAATGTATATTGAATTGTACCAATAGTGTTCTCCTTTTTT
-AAAAG
>Nty
ATGACAAAGGATACAGCAATCGA--GTAATGCATTGCTT---AATTCAGGAAGTATA-CT
AATGGAATATCTAATGTAGAAACTTCATTTCAT---AAAATTGCAGAT------------

--------------------------------ATATT-AAACTTT-AAGATAAAATAAAT

GCTTAACTATGTGACTATGAGTCATTTAAGATAGACATTTAAAT----TAC-TCTTTTAT
TATGATTT--
----------------AA-GGATCCTGTGTCACATATTTCACTTATGA-TGACAA-----

----------------------TTTTGTTTCCTTTTA--AATTT--GTTTCACTATTTTT
-AAAGTTTTCCATTTTATTGAAAAATGTAGAAA----TGTAACTGTATTACCATTTGCTA
TTTTGGTTTTAGTTACTATATAATGTATATTGAATTATACCAATAGTGTTCTCCTTTCTT
-AAAAG
>Dgo
ATGACAAAGGATATAGCAACCGA--GTAATGCATTGCTT---AATTCAGGAAGTATA--C
-ATGGAATATAAAATGTAGAAACTTCATCTCAT---AAAATTGCAGAT------------

--------------------------------ACTTT-AAACT-------TAAAATAAAT
GCTTAACTATGTGACTATGGGTCCATAAAGATAAGCATTTAAAG----TAT-TCTTTTAC
TATGATTTTACTCCCATATAATTAG-----------------------------------
---AACAT-GGGGGGAAA-GATTCT-ATGCCATGAGTTTTATTT--------TTA---TT
TTTACTTATTTTTATTCATTAAATTATTGATGTTTTATTTATGATGGTTTATTATG---C
CATTAATTTATGATGACTA---TTTTGTTTCTTTTT---AATTAAAGTTTTATTATTTTT
-AAGGTTTTCTATTTTATTGAAAAGGGTAGAAA----TGTAATTGTATTACCATTTGCTA
TTTTAATTGCAGATCCTATATGATGTATATTGAATTATAGCAATAGTGTTTTTCTTTTTT
-AAAAG
>Scr
ATGACAAAGGATATAGCAACCGA--GTAATGCATTGCTT---AATTCAGGAAGTATA--C
-ATGGAATATAAAATGTAGAAACTTCATCTCAT---AAAATTGCAGAT------------

--------------------------------ACTTT-AAACT-------TAAAATAAAT
GCTTAACTATGTGACTATGGGTCCATAAAGATAAGCATTTAAAG----TAT-TCTTTTAC
TATGATTTTACTCCCATATAATTAG-----------------------------------
---AACAT-GGGGGGAAA-GATTCT-ATGCCATGAGTCTTATTT--------TTA---TT
TTTACTTATTTTTATTCATTAAATTATTGATGTTTTATTTATGATGGTTTATTATG---C
CATTAATTTATGATGACTA---TTTTGTTCCTTTTT---AATTAAAGTTTTATTATTTTT
-AAAGTTTTCTATTTTATTGAAAAGGGTAGAAA----TGTAATTGTATTACCATTTGCTA
TTTTAATTGCAGATCCTATATGATGTATATTGAATTATAGCAATAGTGTTTTTCTTTTTT
-AAAAG
>Mfa
ATGACAAAGGACACAGCAATCGA--GTAATGCATTGCTT---AATTCAGGAAGTATA--C
-ATGGAATATCAAATGTAGAAACTTCATCTCCC---AAAATTGCAGAT------------

--------------------------------ACTTT-AAACTTT-AAGATAAAATAAAT
GCT-----ATGTGACTATGAGTCCATAAAGATAAGCATTTAAAA----TAC-TCTTTTAG
TATGATTTTACTCCCATTTGATTAG-----------------------------------
---AACAT-GGGGGGAAAAGATTTT-ATGCTACGATTTTCATTT--------TTA---TT
----------------CATTAATTTATTGATGATTTATTGATGATGGTTTATTATGATAC
CATTAATTTATGATGATTA---TTTTGTTTCCTTTT---AATTA--GTTTTATTATTTTT

-TAAGTTTTCTATTTTATTGAAAAGGGTAGAAAGAAGTATAATTGTATTACCATTTGATA
TTTTGATTACAGATCCTATATGGTGTGTATTGAATTACAAAAGTAGTGTTTTCCTTTTTT
-TATAG
>Bil
ATGACAAAGGATACAGTAATCGA--GTAATACATTGCTT---AATTCAAGAAGCATA-CC
AATGGATTATCTAATGTAGAA-CTTCAACTCAT---AAAATTGTAGAT------------

--------------------------------ACTTT-AAACTTTTAAGATAAAGTAAAT
GCTTAACTGTGTGACTATGAGTCTTTAAAGATAGGCATTTAAAAGGCATACCCTTTTCCT
TATGATTTTATTCCAGGTCTTTTGG----------------AAAGTTATCTCCACAGGAC
AAGAACAT-GGAGCAAAA-GATTCTTGTGCCACATGTTTCATTT-----TGATTA-----

----------------------TTTTGTTTCCTCTTT--AATTA--GTTTTACTATTTTT
-AAAGTTTTCTATTTT---GAAAAGGGTAGAAA----TG---TAACATTAACATTTGTTA
TTTTGATTGCAGATCTTTTATAATGTATATTGAATTATACCAATAGTGTTCTCCTTTTTT
-AAAAG
>Pgu
ATGACAAAGGATACAGCAATCGA--GTAATACATTGCTT---AATTCAAGAAGTGTA-CC
AATGGATTATCTAATGTAGAA-CTCCATCTCAT---AAAATTGCAGAT------------

--------------------------------ACTTT-AAACTTT-AAGATAAAATAAAT
GCTTAACTGTGTGACTATGAGTCCTTAAAGATAGGGATTTAAGAGGCATAC-TTTTTTCT
TATAATTTTATTCCATGTCTTTTGG----------------AATATTATCTTCACAGGAC
AAGAACAT-GGGGGAAAA-GATTCTTGTGCTACATGTTTCATTT-----TGATTA-----

----------------------TTTTGTTTCCTCTTT--AATTA--GTTTTACTATTTTT
TAAAGTTTTCTATTTTATTGAAAATGGTAGAA------GTA-TAACATTAACATTTGTTA
TTTTGATCGCAGGTCTTTTATAGTGTATATTGAATTATACCAATAGTGTTCTCCTTTTTT
-AAAAG
>Iso
ATGACAAAGGATACAGCAATCGA--GTAATACATTGCTTCTTAATTCAAGAAGTGTA-CC
AATGGATTATCTAATGTAGAA-CTTCATCTCAT---AAAATTGCAGAT------------

--------------------------------ACTTT-AAACTTT-AAGATAAAATAAAT
GCTTAACTGTGTGACTATGAGTCCTTAAAGATAGGGATTTAAAAGGCATAC-TTTTTTCT
TATGATTTTATTCCATGTCTTTTGG----------------AATATTATCTTCACAGGAC
AAGAACAT-GGGGGAAAA-GATTCTTGTGCCATATGTTCCATTT-----TGATTA-----

----------------------TTTTGTTTCCTCTTT--AATTA--GTTTTACTATTTTT
TAAAGTTTTCTATTTTATTGAAAATGGTAGAA------GTA-TAACATTAACATTTGTTA
TTTTGATCACAGGTCTTTTATAGTGTATATTGAATTATACCAATAGTGTTC---------

>Tro
ATGACAAAGGATACAGCAATCGA--GTAATGCATTGCTT---AATTTAAGAAATGTA--C

```
AATGGAATATCTAATGGRGARACTTCATCTCAT---AAAATTGGAGAT------------
------------------------------------------------------------
------------------------------------------------------------
------------------------------------------------------------
------------------------------------------------------------
--------------------------------ACTTT-CAACTTT-AAGATAAAATAAAT
GCTTAACTGTGTTACTATGATTCATTAAAAATGAGCATTTAAAA----TAT-TCTTTTAC
TATGATTTTACTCAACGTTTTTTGG----------------AAAGTTATCTCCATAGGAC
AAGAACAT-GGGGAAAA--GATACT---GCCACATTTTTCATTTATGA-TAATTA-----
------------------------------------------------------------
----------------------TTTTGTTTCCTT------------GYCTTACTATTTTT
-AAAGTTTTCTATTTTATTGAAAA-------------TGTAACTATATTACCTTTTGCTA
TTTTGGTTGTAGATCCTATAAAATTTATATTGAATTGTACCAATAGTGTTCT--------
------
>Ppe
ATGACAAAGGATACAGCAATCGA--GTAATACATTGCTT---AATTTAAGAAATGTG-CC
AGTGGAATATCTAATGGAGAAACTTCATCTCAT---AAAATTGCAGAT------------
------------------------------------------------------------
------------------------------------------------------------
------------------------------------------------------------
------------------------------------------------------------
--------------------------------ACTTT-AAACTTT-AAGATAAAATAAAT
GCTTAACTGTGTCACTATGATTCATTAAAAATAAGCATTTAAAA----TAT-TCTTTTAC
TATGATTTTACTGAACATCTTTTGG----------------AAGTTAATCTCCATAGGAC
AAGAACAT-GGGGGAAAA-GATTCT-GTGCCACATGTTTCATTTATGC-TGATTA-----
------------------------------------------------------------
----------------------TTTTGTTTCCTTTT---AATTA--GTCTTACTATTTTT
-AAAGTTTTCTATTTTATTGAAGA-------------TGTAACTGTATTACCATTTGATA
TTTTGGTTGCAGATCCTATAGAATGTATATTGAATTGTACCAATAGTGTTC---------
------
>Tvu
ATGACAAAGGATACAGCAATCGA--GTAATGCGTTGCTT---ATTTCAGGAAGTGTAACC
AATGGAATATCTAATGTAGAAACTTCATCTCGT---AAAATTGCAAAT------------
------------------------------------------------------------
------------------------------------------------------------
------------------------------------------------------------
------------------------------------------------------------
--------------------------------ACTTT-AAACTTT-AAGATAAAATAAAT
GCTTAACTGTGTAGCTATGATTCTTTAAAAATAAGCATTTAAAA----TAT-TCTTTTAC
TGTGATTTTACTCAATGCCTTTTGG----------------AAAGTTATCTCCATTGGAC
AAGAACGTTAGGGGAAAA-GATTCT-CTGCCACATGTTTCATTTATGA-TGATTA-----
------------------------------------------------------------
----------------------TTTTGTTTCCTTTT---AATTA--GTCTTACTATTTTT
-AAAGTTTTCTATTTTATTGAAAAGGGTAGAAA----TGTAACTGTATTACCATTTGCTC
TTTTGATTGCAGATCCTATATAATGTATATTGAATTGTACCAATAGTGTTC---------
------
>Mro
ATGACAAAGGATACAGCAATCGA--GTAATGCATTGCTT---AATTCAGGAAGTGTA-CC
AATGCAATATATAATGTTGAAACTTCATCTCAT---AAAATTGCAGAT------------
------------------------------------------------------------
------------------------------------------------------------
------------------------------------------------------------
------------------------------------------------------------
```

--------------------------------ATTTT-AAACTTT-AAGATGAAATAAAT
GCTTAACTGTGTAACTATGGTTCATTGAAAATAAGCACTTAAAA----TAT-TCTTTTAC
TAGGATTTTACTCAATGTCTTTTGG----------------AAAGTTATCTCCATAGGAC
AA--GGGT-GGAGGAAAA-GATTCT-ATGCCATGTGTTTCATTTATGA-TCATTA-----

----------------------TTTTGCTTCTTTTT---AATTA--GTCTTAGTATTTTT
--AAGTTTTCTCTTTTATTGAAAAGGGTAGCAA----TGTAACTGTATCACCATTTGCTA
TTTTAATTGCAAATCCTATGTAGTATATATTCAATTGTACCAATAGTGTTCTCCTTTTTT
-AAAAG
>Ptr
ATGACAAAGGATACAGCAATCGA--GTAATGCGTTGCTT---AATTCAGGAAGTGTA-CC
AATGGAATATATAATATTGAAACTTCATCTCAT---AAAATTGTAGAT------------

--------------------------------ATTTTT-AACTTT-AAGATAAAATAAAT
GCTTAACTATATAACTATGGTTCATTAAAAATAAGCACTAAAAA----TAT-TCTTTTAC
TAGGATTTTACTCAGTGTCTTTTGG----------------AAAGTTATCTCCATAGGAC
AA--GGAT-GGAAGAAAA-GATTCT-ATGTCATGTGTTTCATTTATAA-TCGTTA-----

----------------------TTTTGTTTCTTTTT---AATTA--GTCTTAGTATTTTT
--AAGTTTTCTGTTTTATTGAAAAGGGTATCAA----TGTAACTGTATTACCATTTGCTA
TTTTGATTGCAAATCCTAAATAGTGTATATTCAATTGTACCAATAGTGTTCTCCTTTTTT
-AAAAG
>Vur
ATGACAAAGGATACAGCAATCGA--GTAATGCGTTGCTT---AATTCAGGAAGTGTA-CC
AATGGAATATCTAATGTAGAAACTTCATCTTAT---AAAATTGAAGAT------------

--------------------------------ACTTTTAAACTTT-AAAATAAAATAAAT
GCTTAACTGTGTAACTATGATTCATTAAAGATAAGCATTTAAAA----TAT-TCTTTTAC
TATGATTTTACTCTATGTCTTTTGG----------------AAAGTTACCTCCACAAGAC
AAGAACAT-GGGGGAAAA-GATTCT-GTTTCATATATTTCATTTATGA-TGATTA-----

----------------------TTTTGTTTCCTTT----AATTA--GTCTTACTATTTTT
-AAAGTTTTCTGTTTTACTGAAAAGTATAGAAA----TGTAACT--ATTACCATTTGCTA
TTTTGATTGCAGATGATATATAATGTATATTGAATTGTACCAATGGTGTTCTTCTTTTTT
-AAAAG

MARKER 26

>L3_Mars

----------TCATATGAGGATCGGTTGAAGGAACTGGGNATGTTTAGCCTGGAGAAGAG
AAGACTCAGGGG-GGA--------CATGATAGCTGTCTTCAA-GTATTTGAAGGGCTGTC
ATGTGGAAGAGGGA-TTAGACTTGTTCTGTTTGGCCCCAGAGGGCAGAACCAGGAGCAAT
GGG---

>MAR1

----------------------------------GTTGTTCNAGTCGTTTCAGTTGTGTC
TGACTCTTTGTGACCCCATTTGGG----GTTTTCTT--GGCAAAGATACTGGAGTGGTTT
GCCATTTCCTTCTCCAGCTCATTATTTACAGATGAGGAAACTGAGGCAAACAGGGTTAAG
TGACTTGCCCAGGGTCACACAGCTAGTAAGTGTCTGAGGCCAGATTTGAACTCAGGAAGA
TGAGTCTTCCTGACTCCAGGCCCAGCACTCTATCTACTGTACCACCTAGC----------

>Mdo
CCATGCGTCCTTGTGTACAGTGGAC--CTTCAAAAGAAGATAGGATTAAAATTATGGTGG
GGGCCAAAGTATCCCCTGA-TTATCTAGACTGGTTTTTT--CCCTTGGTCAAATTAACCT
TAAAA--------CCTTGTAAGTTCTATTTCATTTGAGGG------------------CA
TCTGTAGCCTTAGTCAGC--AGTGTAAAATGGGCTCAAGTTTTGGAATGAAATTTTAAAA
GAG-------TATAAAGAATCTTAGCTGAACAAACTAGATATATGAAGCCTGAAGAAGAG
AAGACTAGGAGGAGGAGGAGGAGGCATGACAATCATCTTCAA-GTATGAGAAAGACCTAA

ATAATGAATTGAAA-TTTGATGTGTTCAAATGGTCCCCAGTAGATAG-------------
----------TGTTTTGAAGAGGCAGACCATAGCTAAGAAAATA---CCCCCTGCACCCA
CCTGGCTCCAACTGCAACAGAGAAAGAGTATTTCTTTCCTGGGTCTCAAAGCTTCCTTCC
CTCAAATTTCAGGTCTCGATGCTTCCTTCATCCTCTATTTGGAGATCTCCATGTTCTTAC
CC-TTTGAAGCAGCTCTCTCACCTCAATGCT---------------------------TT
CTACTCTC---AACTTTCATTCTTTAGTTCTGATCCTACATCTTCACCAGCATTACCACC
-ACCACTCACTTTGTTCC---TACAAGCTAT------TGGTTAGTTGTTTCAGTTGTGTC
TGATTCTCTCTGACCCCATTTGGGTTGAGGGTTCTTTTGGCAAGGATACTAAACTAATTT
GCCATTTCCTTCTTCAGCTAAT--TTTACAGCTAAGGAAACTGAGGCAAACAGGGTGATG
TGGCTTTCCCAGGATCACACAGATAGTTAGTG-CTGAGACTGGATTTAAACTTATGAAGA
AGAGTCTTCCTGATTCCA-----------------------------------TACAAGA
CAT-------------------AGGACGAGGT-ATTTAAAGCCACTCAGAGAA-CTCTTC
TC--ATTTCCCAACTAT--------------------------GGATACAAGCATAAAAG
AAATACAGAAGACCAAAAGCATAAGAAACTTATGTAACCCAAGGACAGAAAGGTTTGCAT
GCTTTCCAAGACTTAC--TTTCCTTT--GTTAAATACGGA
>Dvi
CCATGCATCCTTGTGTACAATCGAC--CTTCRAAAAAAGATAGGATTATAATTACAGTGG
GGGCCAAACTATCCCTTGA-TTATCTAGACTGTTCCCCCCGCCCTTGGTCAAATTAACCT
TAAAGAAAGGAATTTTTTTAAGTTCTATTTCATTTGAGGG------------------CA
TTTGTAGCCTTAGTCAGG--AGTGAAAGATGAACTCAAGTTTTGGAATGAAATTTTAA--
GAG-------TATAAAGAATCTTAGCTGAACAAACTAGGTATATGRAACCTGAAGAAGAG
AAGACTTGGAGGAGGAGG------CATGACAATCATCTTCAA-GTATGAGAAGGACCTAA
AGAATGACTAGAAA-TTTGATGTGTCCAAATGGACCCCGATAGATAGAACCAGAGGCAAT
GGG---TGGGTGTTTTGAAGAGGCAAACCATAGCTAAGAAAAT----CCCCCTAGACCTA
CCTGGCCCCAACTGCATCAGAGAAAGAGTCTWTTTTATCTGGATCTCAAAGCTTCCTTCC
CTCAAATTTTACATCTCATTGATTCCTTCACCTTCTATTTGGAGATCTCCATGTTCTTAC
CCATTTGAAGCAATTCTCTCCCCTCAATCTTTTTTTTCTATTGTCAACTTGAAGTAAATT
CTACTTGCTCACGAAGTCATTCTTTAGTTCTAACCCTGCATCTTCTCCAGCATCAGCACC
-ACCACCCACTTTGTTCC---TACAAGCTAT------TGTTTAGTCGTTTCAGTTGTGTC
TGATTCTTTATGACTCCCTTT------AGGTTTGTTTTGGCAAGGATACTAAACTGATTT
CCCATTTCCTTCCTCATCTAAT--TTTACAGATAAGGAAACTGAGGCAAACAGGGTGACA
TGACTTGCCCAGGATCACACAGATAGTAAGTTTATGAGACTGGATTTAAACTCATGAAGA
TGAGTCTTCCTGATTCCA-----------------------------------TACAAGA
CAT-------------------AGGACAGGGG-ATTTAAAACCACTCAGAGCA-CTCTTC
TC--ATTTCCCAACTGT--------------------------GGATACAAGCATAAAAA
AA-TACAGAAGACCAAAAGTACAAGCAACTTATGTAACCCAATGACAGAAAAGTTTTCAT
GTTTCCCAAGACTTAC--TTTCCTTT--GTTAAATATGGA
>Mnu
CCATGCATCCTTGTGTACAGTCGAC--CTTCAAAAAAAGATAGGATTATAATTACAGTGG
GGGCCAAACTATCCCTTGA-TTATCTAGACTGTTCCCCCCACCCTTGGTCAAATTAACCT
TAATGAAAGGAATTTTTT-AAGTTCTATTTCATTTGAGGG------------------CA
TTTGTAGCCTTAGTCAGC--AGTGAAAGATGAACTCAAGTTTTGGAATGAAATTTTAA--
GAG-------TATAAAGAATCTTAGCTGAACAAACTAGGTATATGAAACCTGAAGAACAG
AAGACTTGGAGGAGGAGG------CATGACAATCATCTTCAA-GTATGAGAAGGACCTAA
CGAATGACTAGGAA-TTTGATGTGTTCAAATGGACCCCAATAGATAGAACCAGAGGCAAT
GGG---TGGGTGTTTTGAAGAGGCAAACCATAGCTAAGAAAAT----CCCCCTAGACCTA
CCTGGCCCCAACTGCATCAGAGAAAGAGTCTTTTTTATCTGGATCTCAAAGCTTCCTTCC
CTCAAATTTTACATCTCATTGATTCCTTCACCTTCTATTTGGAGATCTCCATGTTCTTAC
CCATTTGAAGCAATTCTCTCCCCTCAATCCTTTCTTTCTATTGTCAACTTGAAGTGAATT
CTACTTGCTCACAAAGTCATTCTTTAGTTCTAACCCTGCATCTTCACCAGCATCAGCACC
-ACCACCCACTTTGTTCC---TATAAGCTAT------TGTTTAGTTGTTTCAGTTGTGTC
TGATTCTTTATGACTCCCTTT------AGGTTTGTTTTGGCAAGGATACTAAACTGATTT
CCCATTTCCTTCCTCAGCTAAT--TTTACAGATAAGGAAACTGAGGCAAACAGGGTGACA

TGACTTGCCCAGGATCACACAGATAGTAAGTTTATGAGACTGGATTTAAACTCATGAAGA
TGAGTCTTCCTGATTCCA-----------------------------------TACAAGA
CAT-------------------AGGACAGGGG-ATTTAAAACCACTCAGAGCA-CTCTTC
TC--ATTTCCCAACTGT--------------------------GGATACAAGCATAAAAA
AA-TACAGAAGACCAAAAGTACAAGCAACTTATGTAACCCAATGACAGAAAGGTTTTCAT
GTTTCCCAAGACTTAC--TTTCCTTT--GTTAAATATGGA
>Cfu
CCATGCATCCTTGTATACAGTTGAC--CTTCAAAAGAAGACAGGCTTTTTATTAAGATAG
GGGCCAAAGCCTTCACTGGATTATCTAGATTGCTATTTT---CCCTGGTCAAATAAACTT
TTTA-AAAGAA---CTTAAAAACTTCACTTCATTTTAATG------------------CA
TTTACAG---TAGTCAGTAGAGTAAAA--TGGATTCATATTTTGGAATGAAATTTTAA--
--GCATT---TCTTTAACATCCTAGTT-AAAGAACTTAGTATGTGGAC--TTAAGAAGGG
AAGACTTGGAGGAGCAGG------CATGACAATCATCTTCAA-GTAGGTAAAAGACTTAT
AAAGCAAATTGGAAATTATATTTGCTCTAATAGACCCA-GAAGGCAAAATGAGAATCAAT
GGA---TGGATGTTTT-----ACTAGTCCTT-------------------CTCAGATACA
TCTGACTCCAAGTGGAGCATAGAAAGACTCT--CTTAGCTCCATCTCAAATCTCCCTTCC
CTGAAATTTCAAGCCTTATTGCTTCCTT-------TATTTGGAGATCTTCATGCTCTGAC
CC-CTTGAAGCAATGCTTTTGTCTCAATCCTTCCCGTCTACT-ATAGCTTGAAATAAATT
CTACTTGCTCACTAACTCATTCTTTAGTTCTGACCTTAAAGAATTATCATGGCCACCACC
-ATCCCCCACTTTGTTCC--

---TGCAAAT
T--------------------------GGAGT-AACTAAAGAAGTTCAAAGTAGATCTTC
TGTAATCTCCCATTTATGATCATAAGTATATGGACATGGGTACACATAAAAGTAGAAAAA
AAATACAAAACACCAAAAGCATAAGCAACTTAAGTAACCCAACGATAGAAAGACTTGCAT
GTTTCCCAAGACTTAC--TTTCCTTT--GTTAAATATGGA
>Dgl
CCATGCATCCTTATGTACAATTGAC--CTTCAAAAGAAGATAGGGTTATAATTAGTTCAG
GGGTCAGAACATTCATTGAATTATCTAGATTACTCTTTT---CCTTGATCAAAAA-----
---------------------TCTCCACTTCTTTATGGA-------------------CA
TTTATAGCCTTAAACAACAAAGTAAAA--TGGACTTGAATTTGGGGATGAAAGTTTAAAA
GAGAATT---TATATGGCCCTCTAGTTGAAAGAACTAGTTATGTGAAGCCTGGAGAAGGA
AAGACTTGGGGGAGAAGG------CATGACAGTCTTCGTTAA-GTACCTGAAGGACCTGT
ACAGGGAATAGGGA-TTAGATTTGTTCTAGTGGACCCCAGCAGGCAGAACCAGATGAAAT
GGG---TGGAAGTTTTGGAGAAACAGACCATAGGTAAGAAAG----CCCCCCTAGA----
------CCCAACTGCAACAGAGAAAGACTTTCTCTTATCTGGGCCTCAAAGCTCTCTTCC
CTGAAATTTTAGGTCTTACTGCTTCGTTCACCCTTTATTTGGAGATCTTCATGCTCTGTC
AC-CTTGAAGCACTTCTCTTGCCTCAACTCTTCCTTTCTATTGTCAGCTTGAAGT-----
-----------------TATTCTTTAGTTTTGACCCTGCTTCATCA------------CC
CA--CCCCACCTTACTCC--

--CATTTTGTTCCTACAT
TGTAT-----------------AGAATGGGGT-ATCTAAAGTAATTCAGAAT-GTTCATC
TA-AATCTCCCAATTAT--------------------------GGACATGAATATAAAAG
AAACACAGAAGACCAAAAGCATAAGCAACCCACATAACCCAAACACAGAAAGGTTTGCAG
GCTTCCCAAAACTTAC--TTTCCTTT--GTTAAATATGGA
>Pta
CCATGCATCCTTGTGTACAGTTGAC--CTTCAAAAAAAAATATGTTTATAGTTTTATTTA
GGGCCAAAGCATCCACTGAATTATCTAGATTCTGTTTT----CCTTGCTCCAAAAA----

----------------------CTCCATTTCATTTGAGGT------------------CA
TTTACAGCCTCAGTCAGCAAAGTAAAA--TGAACTCAAGTTTTGGAATAAAATTTTAAAA
GAGCATT---TATATAGCATCCTAGTTGAAAGAACTAGTAATGTCAAGCCTGGAAAAAAG
AATACTAGGGGGAGGAAG------TATGACAGTCTTCAAAAA----TGTGAAGGACTTAT
TCAGGGCTTAGAGA-TTGGATTTGTTCTAATGGACCTCAGAATGCAGAATCAGAGGCAAT
GGG---TGAATGTTTTCCAGAGGCTGACCATAAGTAACATGTT----CCCTCCAGTCACA
CCTGGGCCCAACTGCAACAGAGA----CTCTTTCTTATCTGGGTCTCAATGCTTCCTTCC
CTGAAACTTCAGGCCTTATTGGTTCCTTCACCCTTTATTTGTAGATTGCCATCCATTC--
---CTTGATGCGTCGCTCTTGCCTCAATGCCTTCTTTTTGTTGTCAGCTTAAA-----TT
CTACTTGCTCATTAATTCATTCTTTAGTTCTGATCCTGCATCATCATCCAC-------CT
CA-CCCCCATTTTGTTAC--

---TGTGAAG
TAT-------------------AGGAT------ATTTAAAGTAAATTGGAGT-TCTCTTC
TA-AATCTCCCAGTTAT--------------------------GGATACAAGTGTAAAAG
AAATACAAA---TCAAAAGCATAAGTCATTCATGTAACCCAAATACAGAAAGCTTTTAAT
GCTTCCCAAGACTTAC--CTTCCCTTTGGTTAAATATGGA
>Tro
CCATGCATCTTTGTGTACAGTTGAC--CTTCAAAAGAAGWTAGGGTTATAGTCAGTGCAG
AGACAACAGCATCCATTGAATTATCTAGATTGCTATTTT---CCTKGATTAAAAAA----
----------------------CTCCACTTCATTTGAGRG------------------CA
TTTCCAACCTTAGTAAGCAAAGTAAAR--TGAACTTAAGTTTTGGAATKAAATTTCARCA
GAGCCTT---TATATAY---CCTAGTTGAAAGAACTACTTATGTGAAGCCTGGAGAAAGG
RAGATTTAAGGAAGGAAG------CACGACAGTCATCTTCCAARTATGTGAAGGACTTAC
ACARGGAATAGGGA-TTAGATTTGTTCCAATGGATCCCAGAAGACAG-------------
--------------------------TTCATAYACATWTRARAAAGCYCCCCTGAACCCA
CCTGGACCCAACTGCAACAGAGAARGACTCTTTCTTAGCTKGGTCTCAAAG----CTTCC
CTGAAATTTCAGATCTTATTGCTTCTTTCACCCTTTATTTATAAATGTTCATGCTTTAAC
CC-CTTGAAGAGTATCTCCTRCTTCAATCCTTCATTTCTATTGTCAGGCTRAAGTAAATT
CTACTTGCKTACTAATTCATTTTTTATTTCATACCCTACATCATTA------------CC
YG-CCCCCATTTTGTTCC--

---TGCAAGA
TAG-------------------AAGA-TGGGAGATCTAAGGTAATTGAGAGT-GCTCTTC
TA-AATCTCCCARTTAT--------------------------GGACAACAATGTAAAAG
AAATACAGAGGAAAAAAAGCATAAGCAACTCATGCAACCCAACTACAGTAAGATTTGCAT
GCCACACAAGACTTAC--TTCCCTTT--GTTAAATATGGA
>Ppe
CCATGCACCTTTGTGTACACTTGAC--CTTCAAATGAAGATAGGGTTATAGTTAGTGCAA
AGACCAAGAAATCCATTGAATTATCTAGATTGATATCTT---CCTTGATCAAAAAA----
----------------------CTCCACTTCATTTGGGAG-------------------A
GTTCTAACCTTACTAAGCAAAGTAAAA--CAAACTTGAGTTTTGGAATGGAATTTTAACA
GAATATT---TATATAGCACACTAGTTCAAAGAACTACATATGTGAAGCCTGGAGAAGGG
AAGACTTAGGAAAGGAAG------CAAGACAGTCATCTTCCAAGTATGTGAAGGACCAAC
AC--GGAGTAAGGA-TTACATTTGTTCTAATGGACGCCAGAAGGCAAAATCAGAGGCAAT
GGG---TGGATGTTTTGGAGAGGCAGATGATACATATGTAAGAAAGCCCAACTGGACCCA
CCCGGACCCAACTGCAACAAAGAAAGACTCTATCTTATCTGGGTCTCAAAG----TTTCC
CTGAAATTTCAGGTCTTATTGCTTCTTTCACCCTTTATTTGGACATTTTCAGGCTCTGAC
CC-CTTGAAACATCTGTCTTACTTTAATCCTTCCTTTCTATTGTCAGCTTGAAGTAAATT

CCACTTGGTCACTAATTCATTCTTTAGTTCTGACCCTACATCATCA------------TC
CA-GCCCCATTTTGTTCC--

---TGCAAGA
TAT-------------------AGGA-TGGGGTATCTAAAGTAATTCAGAGT-GCTCCTC
TAAAACCTCCCAATTAT--------------------------GGATGAAAGTGTARAAG
AAATACAGAGGACCAAAAGCATAAGGARCTCATGGAACCCAAACACAGAAAGATTTGCAT
GCTGCACAAGACTTAC--TTCCCTTT--GTTAAATACAGA
>Tvu
CCATGCATTCTTGTGTACAGTTGAC--CTTCAAAAGAAGATAGGGTTATAGGTAGTGCAA
GGGTGAAAGCATCCATTGAATTATCTAGATTGCTGTTTT---CCTTAATCGAAAAA----
---------------------TTCCCACTTCATTTGGGGAGGAAGGAAGGTAGAAAGG--
--------------AAGCAAAGTAAAA--TAAACTCAAGTTTTGGGATGAAATTTTAAAA
GAGCATT---CATAGAGCACCCTAGTTGAAAGAACCAGTTATGTGAAGCCTGGAGAAGGG
AAGACTTGGGG-AGGAGG------CATGACTGTCATCTTCAA-GTATGTGAACGACCTAC
ACAGGGAATAGGGA-TTAGATTTGTTCTAATGGACCTCAGAAGGCGGAACCAGAGGCAAT
GGG---TGGATGTTTTGGAGAGGTAGATCATACACATATAAGAAAGGCCCCCTGGACCCA
CCTGGACCCAACTGCAACAGAGAAAGACTCTTTCTTATCTGGTTCTCACAC----CTTCC
CTGAAATTTCAGGCCTTATTGCTTCCTTCGCCCTTGATTTGGAGATTTTCATGCTCTGCC
CC-TTTGAAGAGCCTCTCTTGCTTCAATCCTTCCTTTCCATTGTCAGCTTGAAGTAAATT
CTACTTGGTCACTAATTCATTCTTTAGTTCTGACCCTATGTCATCACCCAC-------CC
CA-CCCCCATTTTGTTCC--

---TGCAAGG
GAA---------------CAAAAGGAATGGAGTATCTAAAGTAATTCAGAGC-GTTCTTC
TA-AATCTCCCAATTAT--------------------------GGGCACAAGTGTAAAAG
AAATACAGAGGACCAAAAGCGTAAGCAGCTCGTGTAACCCAAACACACAAAGATTTGCAT
GCTTCCCAAGACTTAC--TTTCCTTT--GTT---------
>Mro
NCATGCATCTTTGTGTATAGTTGAC--CTTCAAAAGAAGATAGGGTTATAGTTAGCACAG
AGATCAAAGCATCCATTGAAATATCTAAACAGCCATTTT---CCTTGATCAAAAA-----
---------------------TTTCCTCTTCATTTGGAAG------------------CA
TTTCTAGCCTTACTAAGCAAAGTGAAA--TGAACTTGAGTTTTGGAATGAAATTTTAAAA
GAGCATT---TATATAGAATCCTAGTTGAAAGAACTAGTTATGAGAAGCCGGGAGAAGGG
AAGACTTGGGGGAGCAAG------CATGACAGTCATCTTCAA-GTATGTGAAGGATCTAT
AGAAAGAAGAGGGA-TTAAATTTTTTCTAATGGACCGTAGAAGGCAGAACCAGAGGTGAT
GGG---TGGATGTTTTGGAGAGGCAGATCATACATACATAATAAAATTCCCCTGGACCCA
CCTGGACCCAACTGCAACAGAAAAAGACTCTTTCTTATCTGTGTCTAAAAG----CTTCC
CTGACATTTCAGGTCTTATTGCTTCCTTCACCGTTTATTTGGAGATTTTCATGCTTTGAC
CC-CTTGAATCACCTCTCTTGATCCAATCCTTCCTTTCTATTGTCAGTTTGAAGTAAATT
CTACTTGCTCACTTATTCATTCTTTAGTTTTG-ACTTACATCATCA------------CC
CA--CCCCATTTTGTTCC--

---TACAAGG
TAT-------------------AGGA-TGGGGCATCTAAAGTAATTCATAGT-GCTCTTC
TA-AGTCTCCCCATTAT--------------------------GGACACAAGTGTAAAAG
AAATACAGAGAACCAAAAGTGTAAGCAGCTCGTGGAACCCAAACACAGAAATGTTTGCAT

```
GCTTCCCAAGACTTAC--TTTCCTTT--GTTAAATATGGA
>Ptr
CCACGCATCTTTATGTATAGTTGAC--CTTCAAAAGAAGATAGGGTTATAGTTAGCACAG
AGGTCAAAGCATCCACTGAAATATCTAAATGGCCATTTT---CTTTGATCAAAAAA----
----------------------TTCCTCTTCATTTGGGAG------------------CA
TTTCTAGCCTTAGTAAGCAAAGTAAAA--TGAACTTGAGATTTGGAATAAAATTTTAAAA
GAACATT---TATATAGAATTCTAGTTGAAAGAACTAGTTACATGAAGCCTGGAGAAGGG
AAGACTTGGGGGAGCAAG------CATGACAGTCATCTTCAA-GTATGTGAAGGACCTAT
AGAAAGAAGGGGGA-TTAAATTTGTTCTAATGGACCCTAGAAGGCAGAACCAGAGGTGAT
GGG---TGGATGTTTTGGAGAGGCAGATCATACATATGTAAGAAAATCCCCCTGGACCCA
CCTGGACCCAACTGCAACAGAAAAAGACTCTT----ATCTGTTTCTCAAAG----CTTCC
CTGGCATTTCAGGTCTTATTGCTTCCTTCACCCTTTATTGGGAGATTTTCATGCTCTGAC
CC-CGTGAATTGCCTCTCCTGATCCAATCCTTCCTTTCTATTGTCAGTTTGAAGTAAATT
CTACTTGCTTACTCATTTATTCTTTAGTTTTGACCCTACATCATCA------------CC
CA--CCCCATTTTGTTCC------------------------------------------
------------------------------------------------------------
------------------------------------------------------------
------------------------------------------------------------
-----------------------------------------------------TACAAGG
TAT-------------------AGGA-TGGGGTATCTAAAGTAATTCAGAGT-GCTCTTC
TA-AGTCTCCCCATTAT--------------------------GGACACAAGTGTAAAAG
AAATAATGAGAAACA------------GCTCATGAAACCCAAACACACAGAAA-------
CATTTGCAAGACTTAC--TTTCCTTT--GTTAAATATGGA
>Vur
CCATGCATCTTTGTGTACAGTTGAC--CTTCAAAAGAAGATAGTG---TAGTTAGCAGAG
GGACCAAAGCTTCCATTGAATTATACAGATTGCTCTTTT---CCTTGATCAAAAAAA---
----------------------CTCTACTTCATTTGGGGG------------------CA
TTTCCAGTCTTAGTAAGGAAAGTAAAA--TGAACTCGAGTTTTGAAATGAAATTTTAAAA
GAGCATT---TATATAGCACCCTAGCTGAAAGAACTTGTTATGTGAAGCCTGGAGAAGGG
AAGACTTGAGGGAGGAGG------CATGATAGTCATCTTCAA-GTATGTGAAGGATCTAT
ACAAGGAATAGGGA-CTAGATTTGTTCTAATGGACCCCAGAAGGCAGAACCAGAGGCAAT
GGG---TGGATGTTTTGGAGAGGCAGATCATATATAGATAAGAAAGCCCCCCTGGACCCA
TCTGGCCCCAACTGCAACAGAGAAAGATTATTTCTTATCTGGGTCTCAAAG----CTTCC
TTGAAATTTCAGATCTTACTGCTTCCTTCACCCTTTATTTGGAGATTGCCATGTTCTGAC
TC-CTTGAAGCAACTCTCTTGCCTCCATCCTTCCTTTCTATTGTCAGCTTGAAGTAAATT
CTACTTGCTCACTAACTCATTCTTCAGTTCTGACCATACATCATCAGCCACCTCATCCCT
CA-GCCCCATTTTGTCCC------------------------------------------
------------------------------------------------------------
------------------------------------------------------------
------------------------------------------------------------
-----------------------------------------------------TGCAACG
AAT-------------------AGGA-TGGTGTCTCTAAAGTAATTCTGAAT-GCTCTTC
TA-AATCTCCCAATTAT--------------------------GGACACAAGTGTAAAAG
AAATATAGAAAACCAAAAACATAAGCAACTCATGTAACCCAAACACAGAAAGGTTTGCAT
GCTTCCCAAGACTTAC--TTTCCTTT--ATTAAATATGGA
>human
CCATGCCTTCTTGTGGACCATTGAC--CTTCGAAAA------------------------
------------------------------------------------------------
------------------------------------------------------------
------------------------------------------------------------
------------------------------------------------------------
------------------------------------------------------------
------------------------------------------------------------
```


---------------------------------------AAGACAAATTTGCCTATTTCT
ATACCCAGTTACTTAC--TTTCCTTT--GTTAAATACTGA

MARKER 38

```
>Hal1_Opos1_3end
------------------------------------------------------------
------------------------------------------------------------
---------------------------------------------------TTTTTATT-
---ATATACTTAGTA--ACCACCACCACCAACAAAAAA---CATTTAAATAAACAAATGC
ATAAAAAAATTATGTGCAAAACCACAAACTCCACATTGTTTACAATTTGTTTAAAAATAT
GTAAATTATATATGTGTGCTAATATAAACATCCTCTGCTTTTGAGTTCCCTTTGGATTTG
TTTTACTTNGATACTTTTTT-CTCTTGATTTTGTGGC-----TATTTTTNAAATGTAATC
ATAGTATGTATTATTCTTATTCTCTTTTCTTCTCTTTTCATCTCTTCATATATTTCCCTT
ATTTTCTTTATATTTCCAATATTTGTCATTTCTAATAACATAATACAATCCATTACATTC
AAATATCACAATCTATTCAGCCATTTCT--------------------------------
------------------------------------------------------------
------------------------------------------------------------
------------------------------------------------------------
------------------------------------------------------------
----------------------------------------------------
>L1_Opos2_3end
------------------------------------------------------------
------------------------------------------------------------
------------------------------------------------------------
------------------------------------------------------------
------------------------------------------------------------
------------------------------------------------------------
------------------------------------------------------------
------------------------------------------------------------
------------------------------------------------------------
------------------------------------------------------------
------------------------------------------------------------
---------------------------------TTTTTTTTTTCCCCAATTACATGTAAA
AACAATTTTTAACATT--TTTTTAAAAATTTTGAGTTCCAAATTCT--------------
------------------------------------------------------------
----------------------------------------------------
>Mdo
AAAAT--CTAATGAGAAATAACTGAAGCTATAA------------------------GCA
TTGT----GGGAAGTCACAAAAA----GTGTCTACATGTGTCCCGAAAACAAAG--ATTC
TCGTTTGGGGAAAC-GAGCCATTT------CGTGGT---AGGCTAAAA---CTATTA-TC
TTTTTAAAAATTGTATATTAATAACCACAAACAAAAAATAACATTTAGCCAAACAA----
---------GTATGTGAAAAATCACAAGTTTAACATTGTTTCAAATTTGTTTAAAAGTAT
ATAAAGTATATATGTGTTCTAATGCAACTATTCTCTGCTTTTATGTTTCCTTTAGGTTTT
TT-AATTTGGATACTTTTTTCCTCTTGATTTTGTGGCTGC--TATTTTT--AATGTAAGC
ATAGTATGTATCATT----------CCCATCCTCTTTT-A-TTCTCCATATATTTACCTT
ATTTTCTTTATATTTCCGATATTTGTCATTTCTATTAACACACTA-----CTTTACCTTC
AAATAAT----------------------------------------------GATTGAA
CTCTATAAAATTCCACTAGTGTCCACTTGGTGGTGCACAAGACA---TAC--CTTAT---
------------------------------------------------------------
------------------------------------------------------------
GCTCTTGAATGATCTTTTCACTGCCTTCCTGTAGCACCAACTTGTTCTCTCGACCAAGCT
CAGTTCTGCATTCTTCTACTAAAGCTAGGAACCTGTTTTTTTCCACTTCTAT
>Mnu
AAAAT--CTAATGAGAAATAACCGAAGCTATAA------------------------GTA
TTGT----GGTAAGTCACAAAAA----GGGTCTACATGTGTGCCCAAAACAAAAAGATTC
```

```
TCACATGGGGAAAT-GTGCCATTT------CATGGT---AGGCTAAA----CTGTTATGC
TTTTTAAAAATTGTATATTAATAACCACCAACAAAAAA---CATTTAACTAAACAA----
---------GTATGTGCAAAATCACCAGCTGCGCATTGTTAAAATTTGTTTCAAATG-T
ATAAATTTTATACATGTGCTAATA------TCTTCTGCTTTTATGTTTCCTTTAGGTTTG
TTTTATTTGGATACTTTTTT-CTCTTGATTTTGTGGCTGCCATATTTT---AATGTAAGT
ATAGTATGTATCATT----------CTTATTCTCTTTTCTCCTCTTTAAATATTTACCTT
ATTTTCTTTATATTTCCGATATTTGTCATTTCTAATCACACAGTAGAATGCTTTACATTC
AAATAAC-----------------------------CAAG-TTTATTACCTTTGAATGAA
CTCTATAAAATTCCACTAGTGTCCACTTGGTGGTGCATAAGACA---TAC--CTTAT---
------------------------------------------------------------
------------------------------------------------------------
GCTCTTGAATGATCTTTTCACTGCCTTCCTGTAGCACCAACTTGTTCTCTCGACCGAGCT
CAGATCTGCATTCTTCTACTGAAGCTAGAAACCTGTTTTTTTCCACTTCTAT
>Rra
AAAAC--CTATTGAGAAAGACCTGAAACTGTAA------------------------GTA
TTAT----GGGAAACCATAAGAA----GTCTCTACATTATTATAGGGGGGAAAAGGATTC
ACACTTGGG-AAATTGAGTTATTTTGTGATTTTGGTCTCAGGCCAAA-------------
------------------------------------------------------------
------------------------------------------------------------
------------------------------------------------------------
------------------------------------------------------------
------------------------------------------------------------
------------------------------------------------------------
-------------------------------GTATCACAA---------------TCAAA
CTCCATAAAATTCCACCAATGTCCATTTGGTGCAACACGAAACA---TAC--CTTAT---
------------------------------------------------------------
------------------------------------------------------------
GCTCTTGAATGATTTTTTCACTGCCTTCCNGTAGCACTAACTTGTTCTCTCGACCAAGCT
CTGATCTGCATTCTTCTATTGAAGCTAGGAATCTATTTTTCTCTACTTCTAT
>Cfu
AAAAC--CTATTGAGAAAGACCTGAAACTATAA------------------------GTA
TTAT----GGGAAACCATAAGAA----GTCTCTAGATTACTATAGAAAAAAAAGG-ATTC
ACACTTGGG-AAATTGAGTTATTTTGTGATTTTGGTCTCAGGCCAAA-------------
------------------------------------------------------------
------------------------------------------------------------
------------------------------------------------------------
------------------------------------------------------------
------------------------------------------------------------
------------------------------------------------------------
-------------------------------GTATAACAA---------------TCAAA
CTCTATAAAATTCCACCAATGTCCATTTGGTGCAACATGAAACA---TAC--CTTAT---
------------------------------------------------------------
------------------------------------------------------------
GCTCTTGAATGATTTTTTCACTGCCTTCCTGTAGCACTAACTTGTTCTCTCGACTAAGCT
CTGATCTGCATTCTTCTATTGAAGCTAGGAATCTATTTTTCTCTACTTCTAT
>Dgl
AAAAC--CTAATGAGAAACAAGTGAAGTTATAA------------------------GTA
TGGT----GGGAAGCCATTAAAAC-----------------------AACAAA--TATTC
TCCCTTGGG-AAATTGAGACATTTTGTGATTTTGGTCTCAGGCCAAA-------------
------------------------------------------------------------
------------------------------------------------------------
------------------------------------------------------------
------------------------------------------------------------
```


-------------------------------CTATCCCAG--TTG---------------
-------AAATTCCACTAGTGTCCGCTTGGTGGTGTATGAGACA---TAC--CTTAT---

GCTCTTGAATGATCTTTTCACTGCCTTCCTGTAACACCAATTTATTCTCTCGGCCAAGCT
CAGATTCACACTCTTCTACTGAAGCTAGAAACCTGTTTTTCTCCACTTCTAT
>Nty
AAAAT--CTAATGAGAAACATCTGAAGCTGTAA------------------------GTA
TTGTATGCAAGAAACTTCGAAAAG---GTGCTTACATTTGTACAGAAAACAAAAGTATTT
TCCCTTGGG-AAATTGAGTTGTTTTGTGATTTGGGTCTCAGGCCAGA-------------

-------------------------------GTATCACAG-TTCATTACCACTGCACTAA
CTTCATGAAATTCTGCTAGTGTCCACTTGGTGGTGCATGAGACA---TAC--CTTAT---

GCTCTTGAATGATCTTTTCACTGCCTTCTTGTAGCACCAACTTGTTCTCTCTACTAAGCT
CAGATTTGCACTCTTCTACTGAAGCTAGAAATCTGTTTTTTTCCACTTCTAT
>Pta
AAAAT--CTAATGAGAAACAGAAGAAGCTATCA------------------------GTA
TAGTATTTAAGAATCCACAAAAAAA--GTGTCTACATTTGTATAGAAAACAAAAATATTC
TCCCTACAG-AAATTGGGCCATTTTGTCATTCTGGTCTCATGCCAGA-------------

-------------------------------CTATCACAG-GTTGTTACCTTTGG-TGAA
CTTCATGAAATTCCACTAATTTCCACTTGGTGGTGCCTAAGACA---TAC--CTTAT---
ATTTTTTAATTAAATTTTTAAAAATAGGTTTTTGGGTTTTTTTTCCCAGTTACATGCAAT
GAAAATTTTTAGCATTCATTTTTACAAG-------ATCCAAATTCT---TACCTTAT---
GCTCTTGAATGATCTTTTCACTGCCTTCCNGTAGTACCAATTTGTTCTCTCGACCCAGTT
CAGATTTGCACTCTTCTACAGAATCTAGAAATCTATTTTTCTCCACTTCTAT
>Dgo
AAAAT--CTAATGAGAAACAGATGAAGCTATCA-----------------------AGTA
TTGTATTTAAGAATCCACAAAAAA---GTGTCTACATTTATATGGAAAACAAAAATATTC
TCCTTTGGG-AAATTGGGCCAYTTTGTCATTCCGGTCTCATGCCAGA-------------

-------------------------------CTATGACAG-TTTGTTACCTTTGG-TGAA
CTTAATGAAATTCCACTAATGTCCACTTGGTGGTGCATAAGACA---TAC--CTTAT---
ATTTTTTAATTAAATTTTTAAAAA--GTTTTTTTGGCGTTTTTTCCCAGTTACATGTCAT

```
GAAAATTTTTAGCATTCATTTTTATAAG-------ATCCAAATTCT---TACCTTAT---
GCTCTTGAATGATCTTTTCACTGCCTTCCTGCAGCACCAATTTGTTCTCTCGATCCAGTT
CAGATTTGCACTCTTCTACTGAAGCTAGAAATCTATTTTTCTCCACTTCTAT
>Scr
AAAAC--CTAATAAGAAACAGATGAAGCTAKCA------------------------GTA
TTGTATTTAAGAATCCACAAAAAAAATGTGTCTACAGTTATATAGAAAACAAAAATATTC
TCCCTTGGG-AAATTGGGCCATTTTGACATTCTGGTCTCATGCC----------------
------------------------------------------------------------
------------------------------------------------------------
------------------------------------------------------------
------------------------------------------------------------
------------------------------------------------------------
------------------------------------------------------------
--------------------------------AGACATAG-TTTGTTACCTTTAG-TGAA
CTTCATGAAATTCCACTAATGTCCACTTGGTGGTGCATAAGACA---TAC--CTTAT---
ATTTTTAAATTAAATTTTTAAAAA----------------------TAGT--CACGTCAT
GAAAATTTTTAGCATTCATTTTTACAAG-------ATCCAAATTCT---TACCTTAT---
GCTCTTGAATGATCTTTTCACTGCCTTCCTGTAGCACCAATTTGTTCTCTCGACTCAGTT
CAGATTTGCATTCTTCTACTGAAGCCAGAAATCTATTTTTCTCCACTTCTAT
>Mfa
AAAAT--CTAATGAGAAACAGATGAAGTTATCA------------------------GTA
TTGTATTAAAAAATCCACAAAAAG---GTGTCTACATTTGTATAGAAAACAAAAATATTC
TCCCTTGAG-AAATTGGGCCATTTTGTCATTCTGGTCTCATGCCAGG-------------
------------------------------------------------------------
------------------------------------------------------------
------------------------------------------------------------
------------------------------------------------------------
------------------------------------------------------------
------------------------------------------------------------
-------------------------------CTATC--AG-TTTGTTACCTTTGG-CGAA
CTTCATGAAATTCCACTAATGTCCACTTGGTGGTGCATAAGACA---TAA--CTTTT---
----TTAAATTAAATTTTTAAAAA--TAATTTTTTGAGTTTTTTTCCAGTTACATGTCAT
GAAAATTTTTAGCATTCATTTTTACAAG-------ATCCAAATTCT---TACCTTAT---
GCTCTTGAATGATCTTTTCACTGCCTTCCTGTAGCACCAATCTGTTCTCTCGACCCAGTT
CAGATTTGCACTCTTCTACTGAAGCTAGAAATCTATTTTTCTCCACATCTAT
>Bil
AAAAT--CTAATGAGAAACAGCTGAAGCTATAA------------------------ATA
TTGAATAAAGGAAGTCATAAAAAG---GAGTCTACATTTGTAGAGAAAACAAAAACATTC
TCCTTTTCG-AAATTG---CA-----CCTTTTYGGTCTGTCAGACYA-------------
------------------------------------------------------------
------------------------------------------------------------
------------------------------------------------------------
------------------------------------------------------------
------------------------------------------------------------
------------------------------------------------------------
----------------------------------TCATAG-TTTGTTACCTTTTCATGAA
TGCCACAAAATTTCATTACTGTCCACTTGGTGGTGCATGAGACA---TAC--CTTAT---
------------------------------------------------------------
------------------------------------------------------------
GCTCTTGAATGATCTTTTCACTGCCTTCCTGTAGTACCAACTTGTTCTCTCGACCCAGTT
CAGATTTGCATTCTTCAACTAAAGCTAGAAATCTATTTTTCTCCACTTCTAT
>Pgu
AAAAT--CTAACGAGAAACAGCTGAAGTTATAA------------------------ATA
```

```
TTGAATACTGGAAATCACAAAAAAG--ACTTCTATGTTTGTAGAAAAAAGAAAAACATTC
TCCCTTGGG-AAATTG---CA-----TCATTTTGGTCTCAGTCCAA--------------
------------------------------------------------------------
------------------------------------------------------------
------------------------------------------------------------
------------------------------------------------------------
------------------------------------------------------------
------------------------------------------------------------
-------------------------------CTGTCACAG-TTTGTTACCTTTTCATGAA
TGCCACAAAATTTCATCACTGTCCACTTGGTGGGGCACAAGACA---TAC--CTTAT---
------------------------------------------------------------
------------------------------------------------------------
GCTCTTGAATGATCTTTTCACTGCCTTCCTGTAGTACCAACTTGTTCTCTCGACTCAGTT
CAGATTTGCATTCTTCAACTAAAGCTAGAAATCTATTTTTCTCCACTTCTAT
>Iso
AAAAT--CTAAAGAGAAACAGCTGAAGTTATAA------------------------ATA
TTGAATACTGGAAATCACAWAAAG---ACTTCTATGTTTGTAGAGAAAACAAAAACATTC
TCCCTTGGG-AAATTG---CA-----TCATTTTGGTCTCAGTCCAA--------------
------------------------------------------------------------
------------------------------------------------------------
------------------------------------------------------------
------------------------------------------------------------
------------------------------------------------------------
------------------------------------------------------------
-------------------------------CTGTCATAG-TTTGTTACCTTTTCATGAA
TGCCACAAAATTTCATCACTGTCCACTTGGTGGGGCACGAGACA---TAC--CTTAT---
------------------------------------------------------------
------------------------------------------------------------
GCTCTTGAATGATCTTTTCACTGCCTTCCTGTAGTACCAACTTGTTCTCTCGACTCAGTT
CAGATTTGCATTCTTCAACTAAAACTAGAAATCTATTTTTCTCCACTTCTAT
>Tro
AAAAT--CTGATGAGAAACAGCTGAAGCTGTAA------------------------TTA
CTGTATACAGGAAGCCGCAAAAAG---GTGTCTACATTTGTGCTGAAAACAAAAATATTC
TCCCTTGGG-AAATTGAGCCATTTTCTGTTTTTGGTCTCAGGTCAGA-------------
------------------------------------------------------------
------------------------------------------------------------
------------------------------------------------------------
------------------------------------------------------------
------------------------------------------------------------
------------------------------------------------------------
-------------------------------CCATCCCA--TTCATTACCTCTGCAGGAA
CTCCATGAAATTCCACTAATGTCCACTTGATGGTGCATGAGACA---TAC--CTTAT---
------------------------------------------------------------
------------------------------------------------------------
GTTCTTGGATGATCTTTTCACTGCCTTCCAGCTGCACCAACTTGTTCTCTCGTCCAAGCT
CTGATTTGCACTCCTCTACCAAAGCTAAAAATCTGTTTTTCTCCACTTCTAT
>Ppe
AAAAT--CTAACGAGAAACAGCTGAAGCTATAA------------------------GTA
TTGTATACAGGAAGCCAC-AAAAC---GTGTCTACATGTGTACAAAAAACAAAAATATTT
TCCCATGGG-AAACTGAGCCATTTTGCGATTTTGGTCTCAAGCCAGA-------------
------------------------------------------------------------
------------------------------------------------------------
------------------------------------------------------------
```

```
------------------------------------------------------------
------------------------------------------------------------
------------------------------------------------------------
-------------------------------CAATCATCGATTTGTTACCTTTACATGAA
CTCCATGAAATTCCACTAATGTCCACTTGGTGGTGCATGAGACA---TAC--CTTAT---
------------------------------------------------------------
------------------------------------------------------------
GTTCTTGAATGATCTTTTCACTGCCTTCCAGCAGCACCAACTTGTTCTCTCGACCAAGCT
CTGATTTGCACTCTTCTACTGAAGCTAAAAATCGGTTTCTCTCCACTTCTAT
>Mro
AAAAT--CTAGAGGGAAACAGCTGAAGCTATTA------------------------GTA
ATGTACACAGGAAGTCGCAAAAAA---GTGTCTACATTTGCACAGGAAACAAAAATATTC
TCCTTTGGG-AAACTGAGCCATTTTGTGATTTTGGTCTCAGACCAGA-------------
------------------------------------------------------------
------------------------------------------------------------
------------------------------------------------------------
------------------------------------------------------------
------------------------------------------------------------
------------------------------------------------------------
-------------------------------CTATCACAG-ATCTTTATCTTTGTACAAA
CTCGACAAAATTCCACTAACGTCCACTTGGTGGGGCATGAGACA---TAC--CTTAT---
------------------------------------------------------------
------------------------------------------------------------
GCTCTTGAATGATCTTTTCACTGCCCTCCTGTGGCACCAACTTGTTCTCTCGACCAAGCT
CTGATTTGCACTCTTCTATTGAAGATAAAAATCTGTTTTTCTCCACTTCTAT
>Ptr
AAAAT--CTAGAGAGAAACAGCTGAAGCTATAAGTAACATAATAAGTAAATAAAGTAATA
AGTAATATAAGTAAAGTCAAAAAA---GTATCTACATTTGTACAGAAAACAAAAATATTC
TCCTTTGGG-AAACTGAGCCATTTTGTGATTTTGGTCTCAGGCCAGA-------------
------------------------------------------------------------
------------------------------------------------------------
------------------------------------------------------------
------------------------------------------------------------
------------------------------------------------------------
------------------------------------------------------------
-------------------------------CTATCACAG-TTCTATATCTTTGTACAAA
CTCGCCAAAATTCCACTTATATCCACTTGGTGGG-CATGAGACA---TAC--CTTAT---
------------------------------------------------------------
------------------------------------------------------------
GCTCTTGAATGATCTTTTCACTGCCTTCCTGTGGCACCAACTTGTTCTCTCGACCAAGTT
CTGATTTGCACTCTTCTATTGAAGATAAAAATCTGTTTTTCTCCACTTCTAT
>Vur
AAAAT--CTAATGAGAAATAGCTGACAGTATAA------------------------GTA
CTGCATACAGGAAGTTGCAAAAAG---GTGTCTACATTTGTACAGAAAACAAAAATATTC
TCCCTTGGG-AAATGGAGCCATTCTGTGGTTTTGGTCTCAGGCCAGA-------------
------------------------------------------------------------
------------------------------------------------------------
------------------------------------------------------------
------------------------------------------------------------
------------------------------------------------------------
------------------------------------------------------------
-------------------------------CTATCATAG-TTCATTACYTTTGCATGAA
TTCCACAAAATTCCACTAAGGTCCACTTGGTGGTGCATGAGACA---TAC--CTTAT---
```

```
------------------------------------------------------------
------------------------------------------------------------
GCTCTTGAATGATCTTTTCACTGCCTTCCTGTAGCACCAACTTGTTCTCTCGACCAAGTT
CTGATTTGCACTCTTCTACTGAAGCTAGAAATCTGTTTTTCTCCACGTCTAT
```

MARKER 57

>WSINE1

--TTTTTTTTTTTTTT
---GAGGCAATTGGGGTTAAGTGACTTGCCCAGGGTCACACAGCTAGTAAGTGTCTGAGG
CTGGATTTGAACTCAGGTCCTCCTGACTCCAGGGCTGGTGCTCT----------------
-------------------------------------ATCCAC-----------------
--------TGCACCACCTAGCTGCCCCTC-------------------------------

>Mar1b_Mdo

-------------------------------AGGGCAGCTAGGTGGCACAGTGG-ATAGA
GCGCCGGGCCTGGAGTCAGGAAGACCTGAGTTCAAATCTGGCCTCAGACACTTACTAGCT
GTGTGACCCTGGGCAAGTCACTTAACCCTGTTTGCCTCAGTTTCCTCATCTGTAAAATGA
GCTGGAGAAGGAAATGGC--AAACCACTCCAGTATCTTTGCCAA-GAAAACCCCAAATGG
GGTCACGAAGAGTCAGACATGACTGAAAACGACTGAACAACAACAACAA-----------

>WSINE1

---GAGGGGC
AGCTAGGTGGTGCAGTGGATAGAGCACCAGCCCTGGAGTCAGGAGGACCTGAGTTCAAAT
CCAGCCTCAGACACT-----------TACTAGCTGTGTGACCCTGGGCAAGTCACTTAAC
CCCAA-TTGCCTC-AAAAAAAAAAAAAA--------------------------------

>RTE1_Mars

---------------------------------TTGGGTGGACCCTCTGTGG-------A
GGATTTATGGGAGGACATGGACAAGAGT--------------------------------

---CACACAGGATGAGNAGGCATGGATGGGTTGCGATCTGCATCACTGGAGGGAGT-ACC

--------------------------------CACATCGATGAGATCACAGATCCATTGA
AGTATTGAAGTA---TTGAA--

>Mdo
TTCACTTTCTCCACATCTTTTCCTTGTCTCTGAAA--CTTAATTTAAAGAGCAAAGTCAA
TGTTAGCAAGAAGAG-CACTCCAACAGCA---TTTTTGAAGACTA--------GGAACAT

```
GG-TTTG-GGGAGGGCTTGAACAAGAGT--------------------------------
------------------------------------------------------------
------------------------------------------------------------
------------------------------------------------------------
------------------------------------------------------------
---CACTTGGGACGATGAGGAGTGGAGATGTTACACTTTATGCCACTGAAGGGAGG-ACT
------------------------------------------------------------
------------------------------------------------------------
------------------------------------------------------------
------------------------------------------------------------
------------------------------------------------------------
------------------------------------------------------------
--------------------------------CAAAACACAGGT-CCA-TG----AAGGA
AGAGATTCCCTA---CTACTTCTTTA---GAATTAAATTATTCACAGAATGCGATGAGAA
CTGAGCAAGCATCAGCTATCAACTCCCAGTTCATTTTT-CTTAAATAACTGGATCCAATT
GGA-GATGATG-CCCCCGAGGAAACCTTTCTGCTGTCCTTGGTGCTACTGTATTAAAAGA
TTATGTGAT--GGGTTTGCTGCTGAAACTTCAAAGCTGTCCAAAGCAAG-----------
------------------------------------------------------------
------------------------------------------------------------
------------------------------------------------------------
-------------------------------CATGTGCCCCCT---AATCCAATTTGGCC
ACGAACGTAC--CTCCCGATTGTCGGCCACGATCACAA
>Dvi
TTAACTTTCTCCACATCTTTTCCTTGTCTCTGAAA--CTAAATTTAAAGAGCAAAGTCAA
TGTTAGCAAGAAGAA-CACTCAAACGGCA---TTTTCGAGGACTA--------GGAACAT
GGGTTTGAGGGAGGGCTCAAACAAGAAT--------------------------------
------------------------------------------------------------
------------------------------------------------------------
------------------------------------------------------------
------------------------------------------------------------
---CACTTAGGAAGGTGAGGCGTGGAGACGTTGCACTTTATGCCACTGAAGGGAGA-ACC
------------------------------------------------------------
------------------------------------------------------------
------------------------------------------------------------
------------------------------------------------------------
------------------------------------------------------------
------------------------------------------------------------
--------------------------------CATAACAAGGGT-CCACTG----AAGGA
AAAGACTCACAA---CTACTCCTTTA---GAATTAAATTATTCACAGAGTACAAGGAGAA
CTGAGCTAGCATCAGCTATCAACTCCCAGTTCATTTTT-CTTAAATCACTGGACCCAGTT
GGA-GATTCTGGYCCCTGAGGAAACCTTTCTGCTGTCCTTGGTGCTACTGTAGTAGTAGA
TTCTTCGAT-GTGGTTTGCTGCTGAAACTTTGAAGCTGTCCAAAGCAAG-----------
------------------------------------------------------------
------------------------------------------------------------
------------------------------------------------------------
-------------------------------CATGTGCCTCCT---AATCCAATTTGGCC
ACAAACTTAC--CTCCCGATTGTCGGCCACGATCACAA
>Mnu
TTAACTTTCTCCACATCTTTTCCTTGTCTCTGAAA--CTAAATTTAAAGAGCAAAGTCAA
TGTTAGCAAGAAGAA-CACTCAAATGGCA---TTTTCGAGGACTA--------GGAACAT
GGGTTTGAGGGAGGGCTCAAACAAGAAT--------------------------------
------------------------------------------------------------
------------------------------------------------------------
```

```
------------------------------------------------------------
------------------------------------------------------------
---CACTTAGGAAGGTGAGGCGTGGAGATGTTGCACTTTATGCCACTGAAGGGAGA-ACC
------------------------------------------------------------
------------------------------------------------------------
------------------------------------------------------------
------------------------------------------------------------
------------------------------------------------------------
------------------------------------------------------------
--------------------------------CATAACAAGGGT-CCACTG----AAGGA
AAAGACTCCCAA---CTACTCCTTTA---GAATTAAATTATTCACAGAGTACAAGGAGAA
CTGAGCTAGCATCAGCTATCAACTCCCAGTTCATTTTT-CTTAAATCGCTGGACCCAGTT
GGA-GATTCTGGTCCCTGAGGAAACCTTTCTGCTGTCCTTGGTGCTACTGTAGTAGTAGA
TTATTTGAT-GTGGTTTGCTGCTGAAACTTTGAAGCTGTCCAAAGCAAG-----------
------------------------------------------------------------
------------------------------------------------------------
------------------------------------------------------------
-------------------------------CATGTGCCTCCT---AATCCAATTTGGCC
ACAAACTTAC--CTCCCGATTGTCGGCCACGATCACAA
>Rra
TTAACCTTCTCCACATCTTTTCCTTGTCTCTGAAA--CTTAATTCAA-----AAAGTCAA
TGTTAGCAATAAGAA-CAATCTAACAGCA---TTTTTGCGGACTGTGGAGTCATAGACAT
GGGTTTGGGGGAAGGAATGGATAAGAAT---GGGGCAGCAGGATGGCACAGGTGAAAAGA
GCCACTGCT---------------------------------------------------
------------ACCACTTGGGCAGTACCATGGATCATCGTAAGGTGATTCACTA-----
---ATGAAAGCAACTACA-AAAACCACTCCAGTATCCTTGTCAAAGAAAACTTG--AAGG
GGGCACCAAGATTTGGACAGCACTAAAAAGT---ACACAACAACCCAAATGGACAGAAAT
---CACTTAGGAAGATGAAAGGTGGAGAGGATACTCT----GCTACTGAAGGGAGAATTC
------------------------------------------------------------
------------------------------------------------------------
------------------------------------------------------------
------------------------------------------------------------
------------------------------------------------------------
------------------------------------------------------------
--------------------------------ACACCAAGGACT-CCACTACTTCTTCC-
---------------CTACTTCTTAA---TAAT-----------------GGATCAAGAA
TTATTCTAGCATCAGCTATCAAAATCTAACTAATTTTT-CTTAAATAATTAAACCTAACT
TGAAGAA-----TCATTGAGAA-CCCTTTCTGCTGTCCTTAGTGCTACTGTCTACAAAAT
TTGCTTGGT-TTGGCTTATTGCTCCAGTTTCTAAATTGTCCTGAGAAAATGAAGTAACCC
CAAAGTAAGCATGGAGCCCAAATCAAAAAGCAAAAGCT----------------------
------------------------------------------------------------
------------------------------------------------------------
-------------------------------CTAATGGAAGC----ATGAGCATTTGGTC
TGGGACCTAC--CTCCCGATTATCTGCCACGATGACAA
>Cfu
TTAACCTTCTCCACATCTTTTCCTTGTCTCTGAAA--CTTAATTCAA-----AAAGTCAA
TGTGAGCAATAAGAA-CAATCTAACAGCA---TTTTTGAGGACTCTGGAGTCATGAACAT
GGGTTTTGGGGAAGGAATGGACAAGAAT---GGGGCAGCAGGATGGCATAGGTGAAAAGA
GCCACTGCT---------------------------------------------------
------------ACCACTTGGTCAGTTCCATGGGGCATCGTAAGGTGATTCACTA-----
---ATGAAAGAAACTGCA-AAAACCACTCCAGTATACTTGCCAAAGAAAACCTG--AAGG
GGGCACCAAGATATGGACATAACTAAAAAGT---ACACAACAACCCAAATGGACAGAAAT
---CACTTAGGAAGATGAAAGGTGGAGAGGATACTCT----GCTGCTGAAGGGAGAATTC
```


--------------------------------ACACCAAGGACT-CCACTACTTTTTCC-
---------------CTACTCCTTTA---TAAT-----------------GGATCAAGAA
TTATTCTAGCATCAGCTATCAAAATYGAACTAATTTTT-CTTAAATAATTAAACCTAACT
TGAAGAA-----TCATTGAGAA-CCCTTTCTGCTGTCCTTAGTGCTACTGTCTCCAAAAA
TTGCTTGGT-TTGGCKTGTTGCTCCAGTTTCTAAGATGTCCTGAGAGAATGAAGTAACCC
CAAAGTAAGCATGGAGCCCGAATCTTTTG-------CT----------------------

-------------------------------CCAATGGAAGC----ATGAGAGTTTGGTC
TGGGACCTAC--CTCCCGATTATCTGCCACGATGACAA
>Dgl
TTAACTTTCTCCACATCTTTTCCTTGTCTCTGAAA--CTTAAA-----AAGTAAAGTCAA
TGTTAGCAATAAGAT-TAGTCAAACAGTG---TTTCTGAGGACTCTGGAGTGATGAGCAT
GGGTTTTGGGGAGGGCAGGTACAGGAAT--------------------------------

---CACATAGGAAGATGAGGAATGGAGAGATCATACTCTGTGTCACTGAAGGGAGAACGC

--------------------------------ACACCAAGGAATTCATGGGTCCACTGCA
GGAAGATTTTC----CTACTTCTGTA---TAATTAAGTTATTCACGGAATGCAATGAGAG
TTAAGCTAGCATCAGCCATCAACTTCCATTTCATTTTT-CTTAAATAAATGAACCCAAGT
AGA-GAGTACAGACACTGAGGAAACCTTTCTGCTGTCCGTGGTGCTACTGTCT-CAGATG
ATTATTTGATGTGGTTTTTTGCTAATGCTCTTCAACTGTTGTGAAAGAATTAAATAACCC
ATGTGTTTACATG-CCTCCAAATCCCTTT-------CT----------------------

-------------------------------CCAATTTCCTC----ATGGGAGTTTGGCC
ATGAACTTAC--CTCACGATTGTCTGCCACAATCACAA
>Nty
GATTCTTTCTCCACATCTTTTCCTTGTCTCTGAAA--CTTAAT-----GAGTAAAGTCAG
TATTAGCAACAAGAA-TAATCAAACA-TT---TTGGGGGG--CCCTGGGGTCATGAACAT
GAGACTGGAGGAGGGTATGGCTGGGAAT--------------------------------

---CACTTGGGAAGATGAGGAGTGGGGAGGTC--ATTCTCTGTCACTGAAGGGAGAACCC

--------------------------------ACGCCAAGGGGTTCATGGGTC-CGCTGA
AAGAAGACTTTCC--CTACTTCTCTA---GAATCAAATTGTTCACAGAATGCAATGAGAA
GTAAGCTAGCACCAGCTATCAACGTCCACTTCATTTTT-TTTAAGTAACTGAACCCAACT
AGA-GATTATAGTCACCAGGAAAACCTTTCTGCTGTCCTTGGTGCTACTGTCT-CAGATG
ATTACTAGATGTGGTTTGTTGCTGATGCTTTTAAACTCTTGTGAGAGAATGAAATAT-CC
CAAAGCAAACACATGCTCCAAATTCCTTT-------CT---TCAATTTCCTC---GGGGC
AGCTAGATGGCGCAGTGGATAGAGCACTGGCCTTGGACTTAGGAGGACCTGAGTTCAAAT
CCAGCCTCAGACAGACACTTGATATGTTCTATCTGTGTGACCTTGGGCAAGTCACTTAAT
CCCAAATTGCCTCTCCAAAAAAAAGAAG---CCAATTTCCTC----GTGGGAGTTTAGCC
ATGAGCTTAC--CTCGCGATTGTCCGCCACGATCACAA
>Pta
TTAACTTTCTCCACATCTTTTCCCTGCCTCTGAAA--CTGAATTTAAAGAGCAAAGTCAA
TGTTAGCAATCCGAA-TAATCAG----CG---TTCTTTCGGATTTTGGAGTCGTGAACAT
GGATTCAGAGGACGGCACGGACAGGAAT--------------------------------

---CACTTAAGAAGTTGAGGAGTGGGGAGGTC----ACTATGTCACTGTAGGAA--GCGC
---CTTTCCCTACTTCTTTATTTTTT-CTCTACTTTTTTTT-------------------
---------------------------------------TTAATTTTTAAAATGTTTTTT
TCTAAGGCAATTAGGATTAAGTGACTTGCCCAGGGTCACAAAACTAG-----------GC
TCATCTTTGAACTCAGGTCCTTCTGATTTCAGAGCTGGTGCTCT----------------
-------------------------------------ATCCAC-----------------
--------TGCGCCATCTAGTTACCCTTCCCC----------------------------

---------------CTACTTCTTTA---GAATTAACTTGTTCATAGAATGCAATGAGAA
TTAAACTTGCATCAGCTATCAACTTCCAAGTCATTTT-CTTAAATAATGGAACCCAAAG
AGA-GATTATAGTCACTGAAGAAATTTCCCTGCTGTCCTTGGTGCTACTGTCTTT-----
---------GATGTTTTGCTGTTAATGCCTTTCAACTGTTGGGAGAGAATGAAATAA-CC
T-AAGCAGGCACATGCCCCAGATCCCCTT-------CT----------------------

-------------------------------CTAACGTCCCC----ATGGAAGTTTGGCC
ATGAACTTAC--CTCGCGATTGTCCGCCACGATCACAA
>Dgo
TTAACTTTCTCCACATCTTTTCCTTGCCTCTGAAA--CTGAATTTAAAGAGCAAAGTCAA
TGTTAGCAATCTGAA-TAATCAAACAGTG---TTCTTTCGGATTCTGGAGTCATGAACAT
GGATTCAGAGGACGGCACAGACAGGAAT--------------------------------

---CACTTAGGAAATTGAGGAGTGGGGAGGTC----TCTATGTCACTGTAGGAAGT--GC
---CTTTCCCTACTTCTTTATTTTTTCCTCTACTTTTTT---------------------
---AATTTTTTAATTTTTTTTT
CGAG------------TTAAGTGACTTGCCCAGGGTCACACTGCTAG-----------AT
CCAGATTTGAACTCAGGTCCTCCTGACTTCAGGGCTAGTGCTCT----------------
-------------------------------------ACCCAC-----------------
--------TGTACCATCTAGCTACCCTTGCCCC---------------------------

---------------CTACTTCTTTA---GAATTAACTTGTTCATAGAATGCAATGAGAA
TTAAATTTGCATCAGCTATCAATTTCCAAGTCATTTTT-CTTAAATAATGGAACCCAAAG
AGA-GATTACACTCACTGAAGAAATTTTCCTG-TGTCCTTGGTGCTACTGTCTAGATGAA
TGTTTG----TTGTGTTGTTATTAATGCCTTTCAACTGTTGGGAGAAAATGAAATAA-CC
TAAAGCAAACACGGGCCCCAGA---TCTT-------CT----------------------

-------------------------------CTAACGTCCCC----ATGGAACTTTGGCC
ATGAACTTAC--CTCGCGATTGTCCGCCACAATCACAA
>Scr
TTCACTTTCTCCACATCTTTTCCTTGCCTCTGAAA--CTGAATTTAAAGAGCAGAGTCAA
TGTTRGCAATACGAA-TAATTAAA--GTG---TCCTGTCAGATTCCGGAGTCATGAACAT
GGATTCAGAGGACGGCATGGACAGGAAT--------------------------------

---CACTTAGGAAGTTGAGGAGAGAGGTCATT-------ATGCCACTGAAGGAA----CC
---CTTTCCCTACTTCTTTATTTTTTCCTCTACTTCTTTTT-------------------
---AAATTTTTTTT
CCAGAAGCAATTRGGGTTAAGTGACTTGCCCAGGGTCACACAGCTAG-----------GC
CGAGATTTGAACTCAGGTCCTTCTGACTTCAGGGTCGGTGCTCT----------------
-------------------------------------ATCTAC-----------------
--------TGCACCATCTAGCTGCCCTCC-------------------------------

---------------CTACTTCTTTA---GAATTAACTTCTTCATAGAATGCAATGAGAA
TTAAGCTTGCATCAGCTATCAACTTCCAAGTCATTTTT-CTTAAATAATGGAACCAAA-G
AGA-GATTACARTCACTGAAGAAATTTTCCTGCTGTCCTTGGTGCTA-TGTCTAGACGAG
TGTTTG----ATGTGTTGTTGTTAATGCCTTTCAACTGTTGGGAGAGAATGAAATAA-CC
TAAAGCAACCATGTGCCCCAGATCCCCTT-------CT----------------------

-------------------------------CTAATGTCCCC----ATGGAACTTTGGCT
ATGAACTTAC--CTCGCGATTGTCCGCTACAATCACAA
>Mfa
----CTTTCTCCACATCTTTTCCTTGCCTCTGAAA--CTGAATTTCAAGAGCAAAGTCAA
TGTTAGCAAGAAGAA-TAATCAAACAGTG---TTCTTTCGGACTCTGGAGTCACGAACGT
GGATCCGGAGGACGGCATGGATAGGAAT--------------------------------

---CACTTAGGAAGTTGAGGAGTGGAGAGGTCCCTCTCTATGTGACTGAAGGAA----GC
---CTTTCCCTACTTCTTTATTTTTTCCCTTACTTCTTTTTTTTCTTTTTTATCCTTATT
TTTTATTTTGTGAAAATTCTTAAAAAAAAGAAAATGAGAAGAATTTTAAAATTTCATTTT
C-AGTATAACATCATCAATATAGTGTTGC-TTCTCTCAATAATACAG-------------
-GAAAATTATAGAACCATGCTTTGATTTAAAGCTACCCTCTCTTTTTTTCTTTTTTTTAA
TTAAAAGCTTTTTATTTACAAAACATATGAGTGATTTTTCTACATTGACCTTTGCAAAAC
TTTTTTTTTGTTCCATGCTTTTCCCCTCCTTCCCCCCACCCCCACCCCTAGTACATGCTA
AATATATTAAATTATATGACAAATTTCCC-------------------------------
---------------TTACTTCTTTA---GAATTAACTTGTTCATAGAATGCAATGAGAA
TTAAGTTTGCCTCAGCTAGCAACTTCCAAGTCATTTTT-CTTAAATAATGGAACCCAAAG

AGA-GATTACAGTCACTGAAGAAACTTTCCTGCTGTCCTTGGTGCTGCTGTCT-CAAATG
AGTGTTTGATGTG--TTGTGGTTAATGCCTTTCAACTGTTGTGAGAGAATGAAATAA-TC
TAAAGCAAGCACGTGCCCCAAATCCCCTT-------CT----------------------

-------------------------------CTAATGGCCCC----ATGGAACTTTGGCC
ACGAACTTAC--CTCGCGATTGTC--------------
>Bil
TTAACCTTCTCCACATCTTTTCCTTGTCTCTGAAA--CTTAAA-----AGGTAAAGTCAA
TGTTAGCA-TAAAAAATA-TCAAACA-GT---TTTTTGAGGACTCTAGAGTCATGAACTG
GGGATTGGGGGAGTGCATGGACAGGAAT--------------------------------

---CACTTAGGAATATGAAGAATGGAAAGGTC--GTAGTGTGCCATTAAA-------CCC

--------------------------------ACACTGAGGAGTTCATGGGTTCCCAGAA
GGAAGACTTTCT---CTATTTCTTTA---GAATTRAGTTGTTCACAGAATGTAATGAGAA
TAAAGCTAGTATCAACTTCCACTT-------CATTGTT-CTT-AATAGCTGAACCCAACT
AGA-GATTACAGTCATCAAAAAAGCCTTTATGCTGATCTTGGTGATACTGTAA-TAGGTG
GTCATTTAATATGAGTTGCTGCTAATATTTTTAAACTGTTGTGGGAGAATGAAATAA-TC
CAAAGCGAGCACATTCCCCAAA--TCTTT-------CT----------------------

-------------------------------CCAATTTCCTC----ATGGGAGTTTGACC
ATGAACTTAC--CTCACGGTTGTCTGCCACAATCACAA
>Pgu
TTAACCTTCTCCACGTCTTTTCCTTGTCTCTGAAA--CTTAAA-----GGATAAAGTCAA
TGTTAGCAATAAGAA-----CAAACA-GT---TTTTTGAGGACTCTAGAGTCATGAACCA
GGGATTGGGGGAGCGCATGGACAGGAAT--------------------------------

---CAATTAGGAATATGAAGAAGGGAAAGGTC--ATATTGTGTCATTAAA-------CCC

--------------------------------ACATAGAGGAGTTCATGGGTTCACCGAA
GGAAGACTTTCT---CTATTTCTTTA---GAATTAAATTGTTCACAGATTGTAATGAGGA
TTAAGCTAACATCAACTCCCAATT-------CATTTTTTCTTAAATAACTGAACCCGACT
AGA-GATTACAGTCATCAAGAAAACCTTTATGCTGATCTTGGTG-ATTTGTCA-TAGATA
AGTATTTAATGTGATTTATTGCTAATATTTTTAAACTGTTGTGGGAGAATGAAATAA--C
CAAAGCAAGCACATTCCCCAAATCTCTTT-------CT----------------------

-------------------------------CCAATTTGCTC----ATGGGAGTTTGACC
ACGAACTTAC--CTCACGGTTGTCTGCTACAATCACAA
>Iso
TTAACCTTCTCCACGTCTTTTCCTTGTCTCTGAAA--CTTAAA-----GGATAAAGTCAA
TGTTAGCAATAAGAA-----CAAACA-GT---TTTTCGAGGRCTCTAGAGTCATGAACCA
GGGATTGRGGGAGWGCATGGACAGGAAT--------------------------------

---CAATTAGGAATATGAAGAAGGGAAAGGTC--ATATTGTGTCATTAAA-------CCC

--------------------------------ACACAGAGGAGTTCATGGGTTTACCGAA
GRAAGACTTTCT---CTATTTCTTTA---GAATTAAATTGTTCACAGATTGTAATGAGAA
TTAAGCTAACATCAACTCCCAATT-------CATTTTT-CTTAAATAACTWSACCCRACT
AGA-GATTRCAGTCATCAAGAAAACCTTTATGCTGATCTTGGTG-ATTTGTCA-TAGATA
AGTATTTAATGTGATTTGTTGCTAATATTTTTAAACTGTTGTGGGAGAATGAAATAA--C
CAAAGCAAGCACATTCCCCAAATCTCTTT-------CT----------------------

-------------------------------CCAATTTGCTC----ATGGGAGTTTGACC
ACGAACTTAC--CTCACGGTTGTCTGCTACAATCACAA
>Tro
TTAACTTTCTCCACGTCTTTTCCTTGTCTCTGAAA--CTTAATTTCAAGA----------
---------TAAGCA-CAATCAAGCAGTG---TTTTTGAGGACCCTGGAATCATGAATGT
GGGTTTGGGGGGGTGCAKGGAYAGAAAT--------------------------------

---GCCTTGGGAAGATG------------------------------------AGAACCT

--------------------------------TCMCCAAGAAGGTCATGGGTCCACTGAA
AGAACACTTTGT---CTACTTC---------ATTAAATTGTTTACAGAATGGAATGAGAA
TTAAGTGGGCACCAGCTATCAATTCCYGCTTCATTTTT-CTTAAATAACAGAACCCAACT
AGA-G-------TCACCTAGGGAAYCTTTCTGCTGTCCATGGTGCTAYSGACT-CAGATG
ATCATATGAT----------------------AAGCTGTTGGGAGAGAAYGAAATAACCC
CAAAGCAAACACATGCTCCAAATCTCTTY-------CT----------------------

-------------------------------CTAATTTCCTC----AGAGGAGT-GGATA

```
GAGAGCTTAC--CTCCCGATTGTCTGCTACRATCACAA
>Mro
TTAACTTTCTCCACGTCTTTTCCTTGTCTCTGAAA--CTTAATTTAAAGAGTAAAGTCAA
TGTTAGCAATAAGAA-TAATTAAACAGTG---TTTCTGAGGACTCTGGAGCCATGAACAT
GAGTTTGGGGGAGGGCAGGGATAGATAT--------------------------------
------------------------------------------------------------
------------------------------------------------------------
------------------------------------------------------------
------------------------------------------------------------
---CACTTGGGAAGATAAGGAGAGGAGAGGTCACACTATAT--CACTGAAGGGAGAACCC
------------------------------------------------------------
------------------------------------------------------------
------------------------------------------------------------
------------------------------------------------------------
------------------------------------------------------------
------------------------------------------------------------
--------------------------------CATCAAGGAGCTTGATGGGTCCACTGAA
GGAAGACTTCCT---CTACTTCTTTA---GAATTAAATTGTTTGTAGAATGGAATGAGAA
TTAAGTTAGCATCAGCTCTCAATTCCCAATTCATTTTT-CTTAAATAACTGAACCCAACT
AGA-GAGTATAGTCCT-GAGGAAACCTTTCTGCTGTCCTTGGTGCTACTTCCT-CAGATG
ACTATTTGATGTG----ATTGCTAATGCTTTTAAACTGTTGTGAGAGAATGAAATA-CCC
CCAAGCAAACACATGCCCCAAATCTCTTT-------CT----------------------
------------------------------------------------------------
------------------------------------------------------------
-------------------------------CCAATTTCCCC----TTGGGAGTTTGGCT
GTGAACTTAC--CTCACGATTGTCTGCCACTATCACGA
>Ptr
TTAACTTTCTCCACGTCTTTTCCTTGTCTCTGAAA--CTTAATTTAAAGAGTAAAGTCAA
TGTTAGCAATGAGAA-TAATTAAACAGTG---TTTCTGAGGACTCTGGAGCCATGAACAT
GGGTTTGGGGGAGGACATGGACAGAAAT--------------------------------
------------------------------------------------------------
------------------------------------------------------------
------------------------------------------------------------
------------------------------------------------------------
---CACTTGGGAAGATAAGGAGATGAGAGGTCATACTGTAT--CACTGAAGGGAGAACCC
------------------------------------------------------------
------------------------------------------------------------
------------------------------------------------------------
------------------------------------------------------------
------------------------------------------------------------
------------------------------------------------------------
--------------------------------CATCAAGGAGCTTGATGGATCCACTGAA
GGAAGACTTCCT---CTTCTTCTTTA---GAGTTAAATTGTTTGCAGAATGGAATGAGAA
TCAAGTTAGCATCAGCTCTCAATTCCCAATTCATTTTT-CTTAAATAACTGAACCCAACT
AGA-GACTATAGTCTT-GAGGAAACCTTTCTGCTGTCCTTGGTGCTACTTCCT-TAGATG
AATGTTTGATGTG----ATTGCTAATGCTTTTAAACTGTTGTGAGAGAATGAAATA-CCC
CCAAGCAAACCCATGCCCCAAATCTCTTT-------CT----------------------
------------------------------------------------------------
------------------------------------------------------------
-------------------------------CCAATTTCCTT----TTGGGAGTTTGGCT
GTGAACTTAC--CTCACGATTGTCTGCCACTATCACGA
>human
TTAACTTTTTCCAGATCTTTTCCTTGCCTCTGAAA--CTTAACAA--TTTTAAATGGCAG
```

CATTA---

---AATTTTAAA
AAGTTCTTAC--CTCTCGGTTGTCTGCCACGATCACCA

MARKER 85

```
>RTESINE2
------------------------------------------------------------
------------------------------------------------------------
------------------------------------------------------------
-------------------------------------------CCTGACTTTTGTC----
------------------------------------------------------------
------------------------------------------------------------
------------------------------------------------------------
---TTGCCACTGGA-CTTCGATGACTCTGGAAGAGAGAGTGAGGCTGA--------TGAC
TTTGTGCAACTCTGCCTCACTTAAATCCAATTCATGCGCAAGTCAAGACA-TCACCCCAT
GA---TGTCATTGGTCCTCTTC---GAAAACG----------------------------
------------------------------------------------------------
------------------------------------------------------------
----AAGGACGAACAACAACAACAA-----------------------------------
-----------------------------
>WSINE1
------------------------------------------------------------
------------------------------------------------------------
------------------------------------------------------------
------------------------------------------------------------
------------------------------------------------------------
------------------------------------------------------------
------------------------------------------------------------
------------------------------------------------------------
------------------------------------------------------------
-------------------------GAGGGGCAGCTAGGTGGTGCAGTGGATAGAGCACC
AGCCCTGGAGTCAGGAGGACCTGAGTTCAAATCCAGCCTCA-------GACACTTACTAG
CTGTGTGACCCTGGGCAAGTCACTTAACCCCAATTGCCTC---AAAAAAAAAAAAAA---
------------------------------------------------------------
-----------------------------
>WSINE1
------------------------------------------------------------
------------------------------------------------------------
------------------------------------------------------------
-----------------------------------------------------------T
TTTTTTTTTTTTTTG----------------AGGCAATTGGGGTTAAGTGACTTGCCCAG
GGTCACACAGCTA------GTAA-GTGTCTGAGGCTGGATTTGAACTCAGGTCCTCCTGA
CTCCAGGGCTGGTGCTCTATCCACTGCACCACCTAGCTGCCCCTC---------------
------------------------------------------------------------
------------------------------------------------------------
------------------------------------------------------------
------------------------------------------------------------
------------------------------------------------------------
------------------------------------------------------------
-----------------------------
>Mdo
TTGGAAGCAGAGAAGATGGTCAGTAC--CTAAAACCAAAACAGGAGAGAAA--GG-TGGG
ACCCTTCGC-----CGAGC-------TTCCCCATCTCTTCCCGCCTTGGACCTGGGGGGG
GGTTGAGGGTGGGCCACAGGACTGCTGTTTGTTGTGGAGGGGC---AGTGGTCC--GCTC
T---CTCCTTTCC-----------------------------------------------
------------------------------------------------------------
```


----------------------------GAGGAGGCACCCAC--CTTGCGATTGTGGCAG
AACTCGTAACCTTCCGGCTTACACTCGTG
>Cfu
TTAGAAGCAGAGAATATGGTCAGCAC--CT-ACACAAAAATAGGAGAGAAA--GGATAGG
TA-----------TGGAGC--TTCATTTTCCCATTCATTCT-------------------
-------------CTCGGTGATCATTGTTTGCTGCTGAGGGTA---AGCAATCGAATTCT
TCTCCTCCCTTGCTTTGCTCTACAAGAGTCTTGGGCAA----------------------

-------------------------------CCGGCACCTAC--CTTGCGATTGTGGCAG
AACTCATAGCCTTCTGGCTTGCATTCATG
>Dgl
TTAGAAGCAGAGAAGATGGTTAGCAC--CT-GAACAAAAAGAGGAGAGAAA--GGACGAA
CG-----------TTTAGC--TTCATTTTCCC------TGTTGGCTTCCCACCTTGGACC
C--------TGGTGCTTGTAATTGCTGTTTGTTGTTGAGGGGA---AGCTACTAAACTCC
-CTTCCCCTTTGCTCTGCTCTACAAGACC---TACCTC----------CTAAGTGC----

---GTACCTAC---------AGAACTCTGGAGGAGAGAGTGAGGCTGA--------TGAC
TTTTCAAAGCTCTGTCTCACTTAAATCCAATTCATTCTCGAGTCAAGACATCCCCCTCCT
GA---CATCACTGGTCTTCTTT---AAGAAGGAAGGACGA--------GGATAGAGCACT
GGCCCTGGAGTCAGGAGTACCTGAGTTCAAATCCAGCCTCAGACACTTAACACTCACTAG
CTGTGTGACCCTGGGCAAGTCACTTAACCCCAATTGCCTCACTAAAAAAAAAAAAAAAAA
A---AAGAAGGAAGGACGAACAAT---AACAAAGGTACCTAC--CTTGCGGTCGTGACAG
AACTCATAACCTTCCGGCTTGCACTCATG
>Pta
TTAGAAGCAGAGAAGATGGTTAGCAC--CT-AAACAAAAACAGGTTAGAGA--GGATGGA
CAC-CTGGATTTTCTTTGCCGTTCACTCTCCCACTCCCTGTTGGGTTCCCACCTTCAAAG
C--------TGGTTTCTCTTGCTGTTATTTGTTGGTGAGGGAA---AGCTACCAAACTCT
-CCTCCCCTTTGCTCTGCTCTACAA-------GAGCTC-----TGGTACCCAAGTC----

---GTAGGGATGGA-TCTTAAGGACTCTGGAGGAGAGACTAAGGCTGA------------
-------------------CTCAAATCCGATTCACACACAACCCAAGACAT-CCCTCCCT
GA---TGTCACTGGTCCTTCTC--------------------------------------

----AAGAGCAAAGAATGCAC------AACAGAGGGACCTAC--CTTGCGGTTATGGCAG

```
AATTCATAGCCTTCCTGCTTGCACTCGTG
>Dgo
TTAGAAGCAGAGAAGATGGTTAGCAC--CT-AAACAAAAACAGGATAGAGA--GAATGGA
CAC-CTGGATTTTCTTTGCTATTCACCCTCCCACTCCCTGTTGGGTTCCCACCTTCAAAG
C--------TGGTTTCTCTTGCTGTTATTTGTTGGTGAGGGAA---AGCTACCAAACTCT
-CCTCCCCTTTGCTCTGCTG----AGAATCTAGAGCTC-----TGGTACCCAACTC----
------------------------------------------------------------
------------------------------------------------------------
------------------------------------------------------------
---GTAGGGATGGA-TCTTAAGGACTCTGGAGGAAAGACTAAGGCTGA--------CAA-
-----------CTATCTCACTCAAATCCGATTCACACACAACCCAAGACATCCCCTCCCT
GA---TGTCACTGGTCCTTCTC--------------------------------------
------------------------------------------------------------
------------------------------------------------------------
----AAGAGCAAAGAATGCAC------AACAAAGGTACCTAC--CTTGCGATTATGGCAG
AATTCATAGCCTTCCTGCTTGCACTCGTG
>Scr
TTAGAAGCAGAGAAGATGGTTGGCAC--CT-AAACAAAAACAGGATAGAGA--AGTTGGA
CAC-CTGGATTTTCTTTGCTATTCACCCTCCCACTCCCTGTTGGGTTCCCACCTTCAAAA
C--------TGGTTTCTCTTGCTGTTATTTGTTGTTGAGGGAATCCAGCTACCAAACTCT
-CTTCCCCTTTGCTCTGCTCTACAAGAGTCTAGGGCTC-----TGGTACCCAAGGT----
------------------------------------------------------------
------------------------------------------------------------
------------------------------------------------------------
---GTAGGGRTGGA-TCTTAAGGACTCTGGAGGAGAGACTGAGGCTGA--------CAAT
A---------GTACAACTATCAAATCTGATTCACACGCAACCCAAGACATCCCCTTCCT
GA---TGTCACTGGTCCTTCTC--------------------------------------
------------------------------------------------------------
------------------------------------------------------------
----AAGAGCAAAGAATGCAC------AACAAAGGTACCCAC--CTTGCGATTATGGCAG
AATTCATAGCCTTCCGGCTTGCACTCATK
>Mfa
TTAGAAGCAGAGAAGATGGTTAGCAC--CT-AGACAAAAACAGGATGGAAA--GGATAGA
CAC-TGAGATTTTCTTTGCTATTCACCCTTCCACTCACTGTTGGGTTCCCATCTTCAAAG
C--------TAGTTTCTTTTGCTGTTGTTTGTTGTTGAGGGAA---AGCTACCAAACTCT
-CCTCCCCTTTGCTCTGCTCTACAAGAGTCTTGGGCTC-----TGGTATCCAAC------
------------------------------------------------------------
------------------------------------------------------------
------------------------------------------------------------
----TCGGGGT--------GGGGACTCTGGAGGAGAGAGTAAGGCTGA--------CAAC
T--GTAT-ACTCTATCTCACTCAAATCTGCTTCATTTGCAACCTAAGACATTTCCTTCCT
GT---TGTCATTGGTCTTTTTC--------------------------------------
------------------------------------------------------------
------------------------------------------------------------
----AAGGTAT----------------AACAAAGGCACCTAC--CTTACGATTATGGCAG
AATTCATAGCCTTCCTGCTTGCACTCATG
>Bil
TTAGAAGCAGAGAAAATGGTTAGCAC--CT-AAACAAAAACAGAAGAGAAAGAGGATGGA
CACTCAAGCTTTACTTTCCCTTTCACCCTCCCATTCACAGTTAGGTT-CTACCTTGGATG
C---------TGATAGTTCTTGTATTATTTGTTGTTGAGGGAA---AGCTACCACACTCT
-CCTCTCCTTTGCTGTGCTCTACAAGAGTGTTGGGCTC-----TTGCACCCAACAT----
------------------------------------------------------------
------------------------------------------------------------
```

---GTAGGTATATACCCTACAGGACTCTGGAGGAGAGAGTAAGGCTGA--------AGA-
-----------CTGCCTCACTTAAATTCAATTCACACATAAGCCAAGAC-TCACCCTCAT
GG---TGTCATCATTCCTCTTC--------------------------------------

----AAGAATGAAGGATGAACAAT---AGCAAAAGTCCTTAC--CTTGCGATTGTGGCAG
AACTCATAACCTTCCTGTTTGCACTCATG
>Pgu
TTAGAAGCAGAGAAAATGGTTAGCAC--CT-AAACAAAAACAGAAGAGAAAGAGGATGAA
CACTTAAGCTTTACTTTCCCTTTCACCCTCCCATTCACAATTAGGTT-CTACCTTGGACC
C-----------------------TGATTGTTCTTCAGGGAA---TGCTACCATACCCT
-CCTCTCTTTTGCTATGCTCTACAAGAGTCTTGGGCTC-----TTGCACCCACCTT----

---GTAGGTACATACCCTACAGGACTCTGGAGGAGAGAGTAAGGCTGA--------AGAC
T-AGTGCAACTCTGCCTCACTTAAATGTAGTTCACACCTAAGCCAAGAC-TCACCCTCAG
GG---TGTCATCGTTCCTCTTC--------------------------------------

----AAGAAGGAAGGATAAACAAC---AGCAAAGGTACTTAC--CTTGCGATTGTGGCAG
AACTCGTAACCTTCCTGCTTGCACTCATG
>Iso
TTAGAAGCAGAGAAAATGGTTAGCAC--CT-AAACAAAAACAGAAGAGAAAGAGGATGAA
CACTTAAGCTTTACTTTCCCTTTCACCCTCCCATTCACAGTTAGGTT-CTACCTTGGACC
C-----------------------TGATTGTTCTTCAGGGAA---CGCTACCATACCCT
-CCTCTCCTTTGCTGTGCTCTACAAGAGTCTTGGGCTC-----TTGCACCCACCTT----

---GTAGGTCCATACCCTACAGGACTCTGGAGGAGAGAGTAAGGCTGA--------AGAC
T-AGTGCAACTCTGCCTCACTTAAATCTAGTTCACACCTAAGCCAAGAC-TCACCCTCAG
GG---TGTCATCGTTCCTCTTC--------------------------------------

----AAGAAGGAAGGATGAACAAT---AGCAAAGGTACTTAC--CTTGCGATTGTGGCAG
AACTCATAACCTTCCTGCTTGCACTCATK
>Tro
TTAGAAGCAGAGAAGATGGTTAGCAC--CT-AAACAGAAATAGGAGAGAAG--GGATGGG
AGACATGACTTTACTTACCCTTTCTCCCTCCCACTCACTGGTGGGTTCCCACCTTGGACC
C--------------TGGTTATTCTTGTTTGT-GTTGAGGGGA---AGCTTAA-------
----------------------------------GCTC-----TTATCCCTACCTT---T
TTTTCTTTTGCTTTCTGGAGGGGGGAAGGTAGGGCAAGTAGGGTTAAGTGACTTGCCCAA
GATCACACAGCTACTAAATGTTAGGTGTCTGAGGCTGGCTTTGAACTCAGGTCCTCCTGA
CTCCAGGGCTGGTGCTGTATTCACTGTACCTCCTAGCCGCCCCC---TTATCCTCACTTT
---ATAGGTACGTG-CCTACAGGACTC--AAGAAGAGAGTGAGGCTGA-----------
------------GGTCTCACTTCAATCCAAATGTCAGGCAAGTGAAGACCTCACTCTCAC
GA---TGTCACTGTTCCTCTTC--------------------------------------

----CAGCACAAAGGATGAAC------ACCAAGGGGACCTAC--CTTGCGATTGTGGCAG
AACTCGTAACCTTCCGGCTTACACTCGTG

>Tvu
TTGGAAGCAGAGAAGATGGTTAGCAC--CT-AAACGAAAAT-GGAGAGAAA--GGCTGGA
CA--TTGGCTGAACTTTCCCATTCTCCTTCCCTCTCACTGTTGGGTTCCCACCTTGGACC
C-----------TGCTTGTTGTTGCTGTTTGC-GTTGAGGGGA---AGCTACCGTACTTT
-CGG-CCCCTTTGCTTGCTCTAT--GAGTCTTGGGCTC-----CTATCCCTACCTC----

---GTAGGTACGTT-CCTACAGGACCCTGGACGAGAGAGTGAGGCTGA--------CGAC
T--GCGCAAACCTCTCTCACTGCCATCCAGTTCATGCGCAAGTCAAGACCTCACCCTCAT
GA---TGTCATCGGTCCTCTTC--------------------------------------

----CAGAACGAAAGGTGAACAAC---AGCAAGGGTACCTAC--CTTGCGATTGTGGCAG
AACTCGTAACCTTCCGGCTTGCACTCATG
>Mro
TTAGAAGCAGAGAAGATGGTTAGCAC--CT-AAAGAAAAACAG----GAAA--GGATGGA
CC-CTTAGCTTTACTTTCCCATTCACTCTCCCACTCTTCTGTTGGACTCCGGA-------
------------TGC-TTTTCTTGTTGTTTGT-GCTGAGGGGA---AGCTACTGAACTCT
-CCT-CCCATTGGCTTGCTCTACAA---GCTTGGGCTC-----TTATCCCTGCCTC----

---GTAGGGACGTG-CCTACAGGACGCTGGAGGAGAGAATGAGGCTGAAGGAAGGACGAC
T--GCGCAACCCTGTCTCATTTCAATCCATTTCACACTCAAGTCAAGACCTTACCCTCAT
GA---TGACATCGGTCCTCCTC--------------------------------------

----CAGAGCGAAGGAGAAGC------AACAAAGACACCCAC--CTTGCGATTGTGGCAG
AACTCATAACCTTCCGGCTTGCACTCATG
>Ptr
CTAGAAGCAGAAAAGATGGTTAGCAC--CT-AAAGAAAAACAGGAGAGAAA--GGATGGA
CC-CTTAGTTTTACTTTCCCATTCACCCTCCCACTC-TCTGTTGGGTTCCCACC------
------------TGC-TTTTCTTGTTGTTTGT-GCTGAGGGGA---AGCTACTGGACTCC
-CCT-CCCATTGGCTTGCTCTACAA---GCTTGGGCTC-----TTACCCCTGCCTC----

---GTAGGGACGTG-CCTACAGGACGCTGGAGGAGAGAATGAGGTTGAAGGAAGGACAAC
T--GGGCAACCCTGTCTCACTTCAATCCAGTTCACACTCAACTCAAGACCTTACCCTCAT
GAGGACATCATCGGTCCTCCTC--------------------------------------

----C-GGAGGAAGGAGGAGC------ATCAAAGGTACCCAC--CTTGCGATTGTGGCAG
AACTCATAACCTTCCGGCTTGCACTCATG
>Vur
TTAGAAGCAGAGAAGATGGTTAGCAC--CT-AAAGAAAAACAGGAGAGAAA--GGATGGA
CAC-TTAGCTCAACTTTCCCATTCACCCTCCCACCCACTATTGGG------------ACC
C--------------TGGTTGCTC---TTTGTTGTTGAGGGGA---AGCTACCGAACTCT
-CCTCCCCCTTGCTCTGCTCTACAAGAGTCTTGGGCTC-----TTGTACCTACCTC----


```
---TTAGGTTTATG-CCTACAGGACCCTGGAGG--AGAGTCAGGCTGA--------GGAC
T--GTGCAACCCTG-------------CAATTCA-CCGCAAGTCAAGACCTCACCCTCAT
GA---CATCATTGGTCCTCTTC--------------------------------------
------------------------------------------------------------
------------------------------------------------------------
----CAGAACAAAGGACAAACAAC---ATCCAAGGTACCTAC--CTTGCGATTGTGGCAG
AACTCGTAACCTTCTGGCTTGCACTCATG
```

MARKER 89

```
>RTESINE2
------------------------------------------------------------
------------------------------------------------------------
--------------------------GATGCCAAGGTCATCC--ACTGCATCCC------
------------------------GGGCCATCACCAGTCATCCTGACTTTTGTCTTGCCA
CTGGACTTCGATGACTCTGGAAGAGAGAGTGAGGCTGATGACTTTGTGCAACTCTGCCTC
ACTTAAATCCAATTCATGCGCAAGTCAAGACATCACCCCATGATGTCATTGGTCCTCTTC
GAAAACGAAGG-------------------------------------------------
------------------------------------------------------------
------------------------------------------------------------
----------------------------------------------------ACGAACAA
CAACAACAA---------------------------------------------------
------------------------------------------------------------
------------------------------------------------------------
------------------------------------------------------------
-----------------------------------------
>WSINE1
------------------------------------------------------------
------------------------------------------------------------
------------------------------------------------------------
------------------------------------------------------------
------------------------------------------------------------
------------------------------------------------------------
-----------------GAGGGGCAGCTAGGTGGTGCAGTGGATAGAGCACCAGCCCTGG
AGTCA-GGAGGACCTGAGTTCAAATCCAGCCTCAGACACTTACTA--------GCTGTGT
GACCCTGGGCAAGTCACTTAACCCCAATTGCCTCAAAAAAAAAAAAAA------------
------------------------------------------------------------
------------------------------------------------------------
------------------------------------------------------------
------------------------------------------------------------
------------------------------------------------------------
-----------------------------------------
>Mdo
ATCCCCAGCAGAGTTCTACATATTGCTGC-CACTCTGTGATGGTACTTGAGGTTCAGGGC
TAGGAGAGAATGGG--TA--GCAGGCTT-GGTGAGGGTGGT--GGCTGAGG---------
--------------------GGA-------------------------------------
------------------------------------------------------------
------------------------------------------------------------
------------------------------------------------------------
------------------------------------------------------------
------------------------------------------------------------
------------------------------------------------------------
------------------------------------------------------------
----------------CCCAGGACTCCTGCCTTTCTGGTAACC-AGGGGCTTCGCCC--A
CTTCTGCCTTGTAGTAGTAGCTTGTCTCAGATTCTGGGCC--TTAGTTTCCCCTTTGTCC
TCAGCTTCTCAACTAGGTTGGGAGAAGAAT-----AAAGGGAAGGGACCCTTCTTAGGCA
GTGTCCTGGGAGAGGTAGATGCTGTCCAACTTTCTTTTGTCCCAGATTGTGTACATGAAG
GCCAGACATATGGACCGGGGGAGAGTTTCCAGCCAGGCAGC
>Dvi
---------------------ATTGCTGCCCACTCTGTGATGGTACCTGAGGTTCAGGGC
TAGGAGAGAATGGG--TA--GCAGACTT-GGTGAGGGTAGT--GGCT-AGG---------
```

```
----CCAGCAGGATGTCCTGAGA-------------------------------------
------------------------------------------------------------
------------------------------------------------------------
------------------------------------------------------------
------------------------------------------------------------
------------------------------------------------------------
------------------------------------------------------------
------------------------------------------------------------
----------------CCCAGGACCTCTGCCTTTTTGGTAACC-AGGGGTTTCACCC--A
TTTCTGCCTTGTAGTA---GCTTGTCTCTGATTCTGGGCC--TTAGTTTCCCCTTTGTCC
CCAGC-TCTCTACTGAGTTGGGAGAAGAAC-----AAAGGGTAGAGGTCCTTCTCAGGCA
GTGTCCTGGGAGAGGTAGATGCTGTCGAACTTTCCTTCATCCCAGATTGTGTACATGAAG
GTCAGACATATAGGCCGGGGGAGAGTTTCCAGCCAGGCA--
>Mnu
--------------AATTCAGATTGCTGCCCACTCTGTGATGGTACCTGAGGTTCAGGGC
TAGGAGAGAATGGG--TA--GCAGACTT-GGTGAGGGTGGT--GGCT-AGG---------
----CCAGCAGGATGTCCTGAGA-------------------------------------
------------------------------------------------------------
------------------------------------------------------------
------------------------------------------------------------
------------------------------------------------------------
------------------------------------------------------------
------------------------------------------------------------
------------------------------------------------------------
----------------CCCAGGACCTCTGCCTTTTTGGTAACC-AGTGGTTTCACCC--A
TTTCTGCCTTGTAGTA---GCTTGTCTCTGATTCTGGGCC--TTAGTTTCCCCTTTGTCC
CCAGC-TCTCTACTGAGTTGGGAGAAGAAC-----AAAGGGTAGAAGTCCTTCTCAGGCA
ATGTCCTGGGAGAGGTAGATGCTGTCGAACTTTCCTTCATCCCAGATTGTGTACATGAAG
GTCAGACATATAGGCCGGGGGAGAGTTTCCAGCCAGGC---
>Rra
------------------CAGATTGCTGCCCACTCTGTGATGGTACCTGAAGTTCAGAGC
TAGGAGAGAATTGTA-TA--GCAGTGCTGGGTGAGGGTGGT--GGCC-AGGGAGGTTGGC
TGGTCTGGGAGGACTTGCTGAGA-------------------------------------
------------------------------------------------------------
------------------------------------------------------------
------------------------------------------------------------
------------------------------------------------------------
------------------------------------------------------------
------------------------------------------------------------
------------------------------------------------------------
----------------TCTAGGATTCCTGCCTTTGTTGTAACC-AAGGATGTTACCCAAA
TCTTATCTTGTGAGTA---GCTTGTCTCGGTTTCTGTGCC--TCAGTTTCCCCTTCATCC
CCAATTTCTCAATAGGGTTGGGGGAAGAAT-----AAAGG-AAGGGGCCCTGCTCAGGCA
GTGCCCTGGAAGAGGTAGATACTGTAGATCTTTCTTTCATCCCAGATTGTGTTCATGAAG
GCCAGACGTATAGGCCGGGGGAGAGTTTCCAGCCAGGCA--
>Cfu
------------------GTGATTGCTGCCCACTCTGTGATGGTACCTGAAGTTCAGAGC
TAGGAGAGAATTGTA-TA--GCAGTGCCGGGTGAGGGTGGT--GGCC-AGGGAGGTTGGC
TGGTCTGGGAGGACTTGCTGAGA-------------------------------------
------------------------------------------------------------
------------------------------------------------------------
------------------------------------------------------------
------------------------------------------------------------
```


----------------TCTAAGATTCCTGCCTTTGTTGTAACC-AAGGATGTTACCCAAT
TCCTATCTTGTGAGTA---GTTTGTCTCAGTTTCGGTGCC--TCGGTTTCCCCTTCATCC
CCAATTTCTCAATAGGGTTGGGTGAAGAAT-----AAAGG-AAGGG-CCCTGCTCAGGGA
GTGCCCTGGAAGAGGTAGATACTGTAGATCTTTCTTTCATCCCAGATTGTGTTCATGAAG
GCCAGACATATAGGCCTGGGGAGAGTTTCCAGCCAGGCA--
>Dgl
ATCCCCAGCAGGGGTCTACAGACTGCTGCCCATTCTGTGACGGTACCTGAGGGTCAGGGC
TAGGAGGGAACA-------------GTTTGGCGAGGGTGGT--GGCTGGGGGGAG-----
------GC-TGGGC-TGGT--GA---T-----------------------TCCT------
-----------------------GGGGCCACCACCAGGTGTTTTGACTCTTGTCTTACCA
CTGGACTCTGATGACTCTGGAGGAGAATGGGTTCAATGACCTTGTGCAGCTCTGCCTC
ACTTAAACCCAATTCACTCTCAAGTCAAGCCATCACCCT-------CATTGGTCC---TT
GAGAATGAAGG---

--ATGAACAA
CAAACAATGATAG---TCCAGGACTCCTCCCTGTGCTGTAACC-AGGGACCTCACCCCAA
TTTCTACCTTGCAGTA---GTTTGTCTCGGCTTTTGTGCC--TCAGTTTCCCCTTCACCC
CCAACTTTTCAAGTGGATTGGGAGAAGAAT-----AAAGG-AAGGAGTCCTTCTCAGGCA
GTCCCCTGGG-------ATTCTGGTTGATCTTTCCTTCATCCCAGGTTGCATCCATGAAG
GCCAGACATATAGGCCTGGGGAGAGTTTCCAGCCAGGCAGT
>Mfa
ATCC-------AGAATTCGTGATTGCTGCCCACTCTGTGATGGTACCTG-----------
-AGGAGAAAATGGTG-TA--GCCAAGTTTGGTGAGGGTGGCAGGAGCAAGGAG-------
------GC-TGGAT-TGGCAAGA---CTGCTG-AGATCTGGG------ACTCCCACCTTC
AAGGGCTGAGATCCGGAAGTCCTGGGACTGGCCCCAGTCCTCCTGCACCATGTCTTGCCA
CTGGGCTCTGGTACCTCTAGAATAGTGATTAAGGCTGATGACTTTGTACAGTTCTGTCTC
ACTTAAATCCAATTCACTCAAAAGTCAAGAGATCACCCT-------CCTTGGTTCTCTTT
GAGAATGAAGG---

--ATGAACAG
TAAACATCAACAA---TGCAGGACTCCTGCCTTGGTT-TAACC-AGGGCCTTTATAG-AA
TTTCTACT-GGTAGCA---GCTTGTCTCAGATTCTGTGCC--TCAGTTTCCCTGCTTTTT
G----------GCTGGATTAGGGGAAGAAT-----AAAGG-AAGGGGTCCTTCTTGGGCA
GTGCCCCGGGAAAGGTAGAACCTGCTGATCCTTCCTTCATCACAGATTGTATACATGAAG
GCCAGAAGTATAAGCCGGGGGAGAGTTTCCAGCCAGGCA--
>Bil
------------------GTGATTGCTGCCCACTCTGTGATGGTACCAGGGGGTCAGGGC
CAGGAGGAAATGGTG-TA--GCCAGGTTTGGCGAGGGTGGCAGCAGGGAAGAG-------
------GC-TGGGC-TGGCTAGA---CCCACTGAGGTCTAGG------ACTCCT------
-----------------------AAGGACAT-------TATTTTGACTCTTGTCTGGCCA
TTGGACCCCAATGACTATGGAGGAGAATGGGAGGCTGATGACTTTGCCAGGCTCTGTCTT
ATTTAAACCCAGTTGTCTCAAAATTCAAGATATCAACCA-------CTTTGGTCCTCTTT
AAGAATGGAGG---TCAAGGGG-CAGCTAGGTGG--ATGTAGATAGAACATCAGCCTTGG
AGAAACGGAGGACATGAGTTCAAATTTGACCTCAGACACTTAATAATTCCCTAGCTATGT
GATCTTGGGCAAGTCATTTAACTCTG-TTGCCTTGCAAAAA-CCGAAAGAAAGAAAGAAA
GAAAGAAAGAAAGAAAGAAAGAAAGAAAGAAAGAAAA---GAATGGAAG---TCAAATGA
ATAACAG---------TCTAGGATTCTTGCCTTTGTTGTAACTCAGGGGCTTCACCC-AA
TTTTTACCTTATAGTA---GCTTGCCTTAGATTCTGTGCCTCTCAG--------------

----CTTCTCAGCTGCGTTGGGGGATGAATGGAGGAAGGGGAAGGGACCCTTCTCAGGCA
GTGCTCTGGGA------GAAGCTGTTGATCTTTCCTTCATCCCAGATTGCACACATGAAG
GCCAGACATATAAGCCCGGGGAGAGTTTCCAG---------
>Pgu
ATCCTCACCAGGGGTCTACAGATTGCTGCCCGCTCTGTGATGGTAACAGGGGGTTAGGGC
CAGGAGAAAAT-GTG-TA--GCCGCATTTGGTGAGGGAAGCAGCAGGGAGGAG-------
------GC-TGGAC-TGTCTAGA---TTCACCAAGGTCTAGG------ACTCCT------
-----------------------GGGGACATCACCAGTCATCTTGACT-------GGCCG
CTGGACCCCACTGACTCTGGAGGAGAGAGGGAGGCTGATGACTTTGCCCTGTTCTGCCTT
ACTTAAACGCAATTGTCTCAAAATTCAAGATATCACCCA-------CCTTGGTCCCCTTT
AAGAATGGAGG---CCAAGGGGGCAGCTAGGTGGCACAGTGAATAGAGCACCAGCCCTGG
AGTCA-GGAGGTGCTGAATTCAAATCCAGACTCAGACACTTACTAATTACCTAGCTGTGT
GACCTTGGGCAAATCCCTTAACTCCA-TTGCCTTGCAAAAAAACAAAACAAAACAAA---
ACAAAAA---------------------------------GAATGCAAG---TCAAACAA
ATAATAG---------TCCAGGGTTCCTGCCTTTGTTGTAACGCAGGGGCTTCACCC-AG
TTTTTATCTTGGAGTA---GCTTGCCTCAGATTCTGTGCCCCTCAG--------------
----CTTCTCAGCTGTGTTGGGGGATGAGTAGAGGATGGGGAAGGGGCCCTTCTCAGGCA
ATGCCCTT--------------------CTTTCCTTCATCCCAGGTTGTATACATGAAG
GCCAGATGTATAAGCCTGGGGAGAGTTTCCAGCCAGGCAGT
>Iso
ATCCTCACCAGGGGTCTACAGATTGCTGCCCGCTCTGTGATGGTAAC-GGGGGTTAGGGC
CAGGATAAAATGGTG-TA--GCCGCATTTGGCGAGGGAAGCAGCAGGGAGGAG-------
------GC-CGGAC-TGGCTAGA---TTCACCAAGGTCTAGG------ACTCCT------
-----------------------GGGGACATCACCAGTCATCTTGACT-------GGCTG
CTGGACCCCACTGACTCTGGAGGAGAGAGGGAGGCTGATGACTTTGCCCTGTTCTGCCTT
ACTTAAACGCAATTGTCTCAAAATTCAAGATATCACCCA-------CCTGGGTCCCCTTT
AAGAATGGAGG---CCAAGGGGGCAGCTAGGTGGCACGGTGAATAGAGCACCAGCCCTGG
AGTCA-GGAGGTGCTGAATTCGAATCCGGACTCAGACACTTAGTAATTACCTAGCTGTGT
GACCTTGGGCAAATCCCTTAACTCCA-TTGCCTTGCAAAAAA-CAAAACAAAACAACAAA
ACAAAAA---------------------------------GAATGCAAG---TCAAACAA
ATAATAG---------TCCAGGGTTCCTGCCTTTGTTGTAACGCAGGGGCTTCACCC-AG
TTTTTATCTTG------------------GATTCTGTGCCCCTCAG--------------
----CTTCTCAGCTGTGTTGGGGGATGAATAGAGGATGGGGAAGGGGCCCTTCTCGGGCA
ATGCCCTT--------------------CTTTCCTTCATCCCAGGTTGTATACATGAAG
GCCAGACGTATAAGCCTGGGGAGAGTTTCCAGCCAGGCAGT
>Tro
---------------------ATTGCTGCCCACTCTGTGATGGTACCTGAGGGTCAAGGC
CAGGAGAACATGGTG-TC--TCA------GGGTGGCGAGG--TTGGCGAGGGG-------
------GG-GGAAG-CAGGGAGG---GGT-CTGAGGTCTAGG------ACTCCT------
-----------------------GGGGCCATGGCCAGTCCTCTCGACCCTTGTCCTGCCA
CTGGACTCTGATGACTCTGGAGAAACAAGTGAGGCTGATGACTGTGCACAGTTCTGGGG-
CAATAAACCCAATTCACTCCAAAGTC----------------------------CTCTTC
GAGAATGGAGG---

--ATGAACAG
ACAACAA---------GCCAGGACTCCTGCCTTTGTTGTAAC--TGGGGCGTCACCC-AA
TTTCTCGCCTC-AGATT--CTGTGCCTCAG---TTTCCCC--TCTGCCTCCC--------
----CTTCTCAGCT----------------------GGGGGAAGGGGTCCTTCTCAGGTA
GTGCCCTGGGAG-AGTAGATGCTGTTGAACTTTCCGTCATTCCAGATTGTGTACATGAAG
GCCAGACGTACGGGCCGGGAGAGAGTTTCCAGCCAGGC---
>Ppe
------------------TCAGATGCTGCCCACTCTGTGATGGTACCTGAGGGTCGGGGC

CAGTAGAAAATGGTGGTATCGCAGGGTTT-GGTGGGGTGGCAGCGGGGAGGGG-------
------GG---------------------CTGGGGTCCAGG------ACTCCT------
-----------------------GGGGCCATTGCCAGTCCTCTTGACTCTTGTCATGCCA
CTGGACTCCGATGACTCCGGAGAAGCAAGTGAAGCTGATGACTCTGCACAGCTCTGCCAC
GCTTAAACCCAAGTCCCTCCAAAGTCCAGATATCACTCT-------CATTGGTGCTCTTT
GAGAATGAAGG---

---ACAAACAA
CAAACAATAAGAA---ACCAGGACTCCTGCCTTGGTTGTAAC--AGGGGCTTCACCC-AA
TTTCTACTTCATAGTA---GCTTGTCTCAGATTCTGTGCC--TCAGTTTCCCCTTTATCC
CCAGCTTCTGAGCTGGGTTGGGGGAAGAAT------AAGGGAAGGAATCCTTCTCAGGCA
GTGCCCTGGGAGAGGTAGATGCTGTTGACCTTTCCTTCATCCCAGATTGTGTACATGAAG
GCCAGACATATAGGCCTGGGGAGAGTTTCCAGCCAGGC---
>Tvu
--------------------GATTGCTGCCCACTCTGTGATGGTACTTGAGAGTCAGGGC
TAGGAGAAAATGGTG-TA--GCAGGGTTTTGTGAGGGTAGTGGCAGGGACGGG-------
------GC-TAGGC-CGGCAAGA---TTT-CAAAGGCTGAGGTCCAGAACTCCT------
-----------------------AGGGTCAGCGCTCGTCCTCTTGACTCCTGTCATGCCA
CTGGACTCTGATGACTCTGGAGGAGTGAGTGAGGCTGATGACTTTGCACAGCTCTGCCTC
ACTTAAACCCAATTCACTCAGAAGTCCAAATACCACCCT-------CATCGGTCCTCCTT
GAGAATGAATG---

---ACAAACAA
CAAACACCAATAA---TTCAGGACTCCTGCCTTTGTTGTAACC-AGGAGCTTCACCC-AA
TTTCTACTCTGTAGTA---GCTTGTCTCAGATTCTGTGCC--TCAGTTTCCCCTTTGTCC
CTGGCTTCTCAGCTGTGTTGGGAGAAGAAT------AAAGAAGGGAGTCCTTCTCAGGCA
GTGCCCTGGGAGAGGTAGATGCTGTTGACCTTTCCTCTATCCCAGATTGTGTACATGAAG
GCCAGACACATAGACCCGGGGAGAGTTTCCAGC--------
>Mro
ATCCCCAACAAGGGTCTACAGACTGCTGCCCACTCTGTGATGGTACCTGAGGGTCAGGGC
TAGGAGAAAATGGTG-TA--GCAGGGTTT-GGGAGGGTAGCAGCAGGAAGGGG-------
------GC-TGGGC-TGGCAAGA---CTG-CTGGGGTCTAGG------ACTCCT------
-----------------------GGGGCCATCACCAGTCCTCTTGACTCTTGTCATGCCC
ATGGACTCCAAAGACCCTGGAGGACCATGTGAGGTTCATGCCTTTGCACAGCTCTGCCTC
ACTTGAACCCATTTCACTCCAAAGTC-----------CT-------CACTGGACCTCTTT
GAGAATGAAGG---

---ATGAACA-
CAAAAAC-AAGAA---TCCAGAACTCCTGCCTTTGTTGTAACC-ACGGGCTTTACCC-AA
TTTCTACTTTGTAGTT---GCTCGTCCCAGATTCTGTGCT--TCTGTTTCCCCTTTGTCC
CCAGCTTCTCAGCTGGGTCGGGTGAAGAAT------AAAGGAAGGGGTCCTTCTCAGGCA
GTGCCCTAGGAGAGGAAGATGTTGTTGATCTTTCCTTCATCCCAGATTGTGTACATGAAG
GCCAGACATATAAGCCTGGAGAGAGTTTCCAGCCAGGCAGT
>Ptr
---------------------ATTGCTGCCCACTCTGTGATGGTACCTGAGGGTCAGGGT
TAGGAGAAAATGGTG-TA--GTGGGATTTGGTGAGGGTAGCAGCAGGAAAGGG-------
------GCTTGGAA-TGATAAGA---CTTGCTGGGGTCTAGG------ACTTCT------
-----------------------GGGGCCATCACCAGTCCTCTTGACTCTTGTCATGCCC
ATGGACTTCAATGACCCTGGAGGACCATGTGAGGCTCATGACTTTGCACAGCTCTGCCTC
ACTTAAACCCATTTCACTTAAAAGTC-----------CT-------CACTGGACCTCTTT

GAGAATGAAGG---

--ATGAACA-
CAAAAAC-AAGAA---TCCAGAACTCCTGCCTTTGTTGTAACC-TCGGGCTTCACCC-AA
TTTCTACTTTGTAGTT---GCTCATCTCAGATTCTATGCC--TCGGTTTCCCCTTTGTCT
CCAGCTTCTCAGCTGGACTGGGTGAAGAAT------AAAGAAAGGGGTCCTTCTCAGGCA
GTGCCCTAGGAGAGGTAGATGTTGCTGATCTTTCCTTCATCACAGATTGTGTACATGAAG
GCCAGACATACAGGCCTGGAGAGAGTTTCCAGCCAGGCA--
>Vur
--------------------GATTGCTGCCCACTCTGTGATGGTACCTGAGGGTCAGGGT
TAGGAAAAAAATGGAG-TA--GCAGGGCTCGGTGAGGGTGGCGGAAGGGAGGAG-------
------GC-TGGGC-TGGCAAGG---TTTACTGAGGTCTAGG------ACTCCTGCCTTC
AA---------------------AGGCTGAGATCCAGTTGTCTTGAC--TTGTCTTGCCA
CTGGACTCTGATGACTCTGGAGGAGTGAGTGAGGCTGA---CTTTGCACAACTCTGCCTC
ACTTGAACCCAATTCACGTGAAAGTCAAGATATCACTCT-------CATTGGTCCTCTTT
GAGAATGAAGG---

--ACAATCC-
--GGGGGCTTCACCC-AA
TTTCTACCTTGTAGTA---GCCTGTCTCAGATTCCGTGCT--TCACTCTCCCCTTTGTCC
CCAGCTTCTCAGCTGGGTTGGGGGATGAAT------AAAGGAAGGGGTGCTTCTCAGGCA
GTGCCCTGAGAGAGGGAGACACTGTTGATCTTTCATTCATCCCAGACTGTGTACATGAAG
GCCAGACATATAGGCCTGGGGAGAGTTTCCAGCCAGGCA--

MARKER 90

>WALLSI1A

--TT--GT-TGTTTGTCCTT
TGTTCTCAAAGAGGACCATGA-CATCAGGGAGGTGATGMCATGACTTGCAAGTGAATTGG
ATTTAAGTGAGGGAGGGCTGTGCAAAGTCACCAG-CCTCACTTTCTCCTCCAGAGCCATC
TGGGTCCAGTGGC-AAGATATAGATCAGGATGA-CTGGAGATGGCCCAGGATGCAATGGG
AGACCTTGGCCTTTTTAAGCTAAGGTCTTTCNCAGGTCTCAGTTTGACTGAGGCAACACC
-----CATTC-AGTGATTAAGGCTAGGTAAGAAATGAGGC-AAAGAATGGCCTCT-TTTA
CCTAGTCAAAAAAAAAAAAAAAATCAATCTGGGAGGGGAAGA--CCCTCAGGGTTTCT--
-GGCCAAAACAGAAAC--

>Mdo
GTCACCATCATACAAACCACGTTCCAGGACCGTCAGTTCCGTCAAGCACGGTTTGGCATT
CTCACATC--CTGGAGGGG----------TGAGAATGGGAGAAGAGAGATTATTGTTGGA
TGCCCCAAAGTCAGCCCAGCTACCCTT-CCCTTGCAACAAATTCCAGCTTTTAAAACTGC
CCTCTGCAGGAAAATCTTCCTAGATTTG--------TGTCCCATGTCCTTCTCTCCCCAG
GCCCAGGTAGATCTC-CAAATATCCCTTTCCCTTCAGCAGGTAAAA--------------
TGTCTATCTGTTGTACCCTTCAACCATGGG----------TTCCGTGAAGGTTCATTC--
------------------TTGTCTCAGAAGCGCACAGCTCTTTCCC-TAGC----------
----

--------------------------------GATAGAGTATGACTCTGAAATGAAATTC
ATTCATACAAAATGAAGCCTTTC-CTACTCAGATCCAGGCCCTACCCTCTCTAGGAGTTT
ACATTCCAAGTCTATGCAG-----------CAAAAGAGT---------------------

--------------------CAAGACCACACTGGAGT---------AGCCACGGGAGAGG
GGGC-TGAGTCCGCCCAGGCTCCCTAC--CTTCTGTCCCTT
>Dvi
GTCGCCATCATACAAACCACGTTCCAGGACCGTCAGTTCCGTCAAGCACGGTTTGGCATT
CTCACATC--NTGGAAGAT----------CAAGAATGGGAAAAGAGGAATTATTGTGGGA
TGGCCAAAAGTCAGCCCAGCTATCCTT-CCCTTGCAGCAAATCTTAGCTTTTAAAACTGT
CCTCAGCAGGAAAACCTTCCTGGATTTG--------TCCCCCATGTCCTTCTCTCCCCAG

GCTCAGGTAAATCCC-CAAATATCCCTTTCCCTTCAGCAGATAAAA--------------
TGTCTATCTGTTGTACCCTTCAAGTATGGG----------TTCCCTGAAGGTTCCTTC--
------------------TTGTCTCAGAAGCACACAGCTCTTTCCC-TAGT---------

--------------------------------GATAGAGTATGGCTCTAAAATGAAAGTC
ATTCATATAGAAGGAAACCTTTC-CTACTCAGATCCAGGTCCTATC--CTCTAGGAGTTT
ACA-TCCAAGGCTATGCAG-----------CAAAAGAGT---------------------

--------------------TAAGACCGCACTGGTGT---------AGCCATGGGAGAGG
GGTC-TGAGCCCACCCAGGCTCCCTAC--CTTCTGTCCCTT
>Dgl
ATCACTATCATACATCCCACGCTCCAGGACCGTCAGTTCCGTCAGGCACGGCTTGGCATT
CTCACAGC--CTGGGGGGT-----------GGCAATGGGAGAAGGGAAA---CTGTGGAA
TGCCCATTAGTCAGCCCAGCTGCCCTCCCCCTTC-AACAAATCCTGGCTTGAAGAAA--T
CCTCTAAAGGA-------------TTTG---------CACCCATGTCCTTCTCTCCACTG
GCCCAA----------GGGTTGTCCCTTTCCTTTCAGCAGCAACATTTCCTTAGACAACA
CCTCTATTTGTTGTACCCTTAGACCTTGGG----------TTCCCTGAGGGTTTATTCTT
GCCTCAGAAGCACAAAG-TTGTTGCACCTCA-TTCAATACT-GGCC-GAGT---------

--------------------------------GATAGAGTATGACCCTAAAATGAAATTC
ACTCATATGGAATGAA----------------TGCCA-GTCCTGCC--CTCTGTCAGTTT
ACATTCCAAGGCTATGTAACAAAAGAGCAGCCTGAGAGT---------------------

--------------------TAAGACCACACCCACTTGGGCAGTCCAGTACAATGAGAGG
AGGC-TGAGCAGGCCCAGACTCCCTAC--CTTCTGTCCCTT
>Nty
ATCACCATCATACAATCCACGCTCTAGGACCACCAGTTCTGTCAGGCACGGTTTAGCGTT
CTCACAGC--CTGGGAGCAGAGGGAAGG--GGGAATAGGAGATGGAAGA---TTGTGGGA
TAACCACAAGTCAGCCCAGCTGCCCTCCCCCTGC-AGCAAATCCTGGCTTGAAGAACTGC
CATCTGCAGGAAAACTTTCCTGGATTTG---------CCCCCATGTTCTTCCCTCCACAG
ACCCAGGTAAATCCCTGAGCTGTCCTTT-CCTTTCAGCAGCAACATT-CCTTTGACCACA
TGTCTATCTGTTGTACCCTTAGGCCATGGG----------TTCCCTGAGAGTTTATTCCT
GCCTCAGAAGCACAAAGC-TGCTCCTGGGCA-TTCAATAT-TGGCCAATGTT------GG
GGCAGCTAGGTGGTACAGTGGATAAAGCACCAGGCCTGGAGTCAGGAGAATCTGAGTTCA
AATCTGGCCTCAGACACTTGACATTTAACTAGCTTGTGTGACCCTGGGCAANTNNNGAAN
GCCCNGGNGCCGCNNNGNGNNTNNGAGGNNGGANNAAA---NNNNNNNN--CNNGNTCNC
ACAGNTAGTACATATCAAGTGTNTGTNTGAGGNTGGATTGGAANTAGGTCCTCNTAAATC

```
CAAGGACTCTCTCCANTGTGCCA---------TNTAGC---TGCC--CCCAATGGAATCC
ATTCATACAGAATGAAAGCCTGCCCTGCACAGAACCTGGTCCTACT--CTCTGGAAGTTT
ATATTCCAAGGCTATGCAA--------CAA--AAAGCA----TTTTGT-CC---GCCCTT
TGTTCCAGAAGAGGACCAGGGGCATCAAGGAGGTGATGTCTTCACTTACAAGTGAATTAG
ATTTAAGT--GGCAGAGCTGTGC--AGTCATCAGCCTCAC-CCTCTCCTCCAGAGCCATT
AGAATCTAGTGAC-AAGATATAGGGCAAGATAA-CTGGTGGATGGCCCCGATGCTGTGAA
AGG-CAAAGCCTGTTTAAGCCAAAGTCTTTTCTAAGTCTCAGTTTCTCTGAGGCAAAAGC
-----CATTCAATGATTCAAGGATAGGTGAGAACTGAGGTAAAAGAATGGCTTAG-TTTA
CCTTCA---------TAAAAGACTTAATCAGGGAGGAAAAGA--CCCTCAAGGTTTCT--
-C--TTAAGCTGAGAG----TAAGACCACACT-GGGG--------CTGTTT---------
--------------TGGGCTCCAGTAC--CTCCTGTCCC--
>Pta
ATCACCATCATACACCCCACGCTCTAGGACTGTTAGTTCTGTTAGGCATGGTTTGGCATT
CTCACAGC--CTGAGGAGT-----------AAGAATAGGAAAAGAGAAA---TTGTGGGA
T------------GCCCAGCTG---------------TAAATCCTGACTTAAACAAGT-G
GACCTGCAGAAAGACGCTCCTGGATTTC---------CTCCCATGTTCTTCTCTCCACAG
GCCCACATAAAGTCTTGGACTGTCCCTTTCCTTTCAGTAGGCACATTTCCTTAGACAACA
TGTCTATCTGTTGTGCCCTTAGAACATGGA----------TTTCTTGAGAGTGTATTCTT
GCCTCAGAAGC--------TGTTCCTGGTTG-TTCA-----TTGGCTTAAT---------
------------------------------------------------------------
------------------------------------------------------------
------------------------------------------------------------
------------------------------------------------------------
--------------------------------GATAAAGTATGGCCTCCAAATTAAATTC
ATTCATATAGAAT-AAAACCTGACCTACTCTACTAA---TCCTGCT--CTGTGGAAGTTT
ACATTCCAAGGTTATACAA--------CA---AAAACAT---TGTTGC-TGTTCATCCTT
TGATCTCAAAAGGGACCAATGACCCCAGGAAGGTGATATATTGACTTTC-----------
----AAATGAGGGAGGGCTGTGCAAGGTCATCTGCCTCAC-TTTCCCCTCCAGAGTTATC
TAGGTCCAGTAGC-AAGACACAGATCAGGACGACCTGGTGATGGCTCTAGATGCAATGGG
AGACCTTGGCCATTTAAAGCGCAAGCCA-----------AAGTCTGTCTGAGGTAACACC
-----TATTC-CATTGTCAAGGCTAGGTGAGAAATGAGAT-AAAAGATGGCCTAGTTTTG
CCTTCA---------AAGAAGAATCCATCAGGGAGGGAAAGA--------------CT--
-C--TTAGCCTGAGAGT---TAAGAGCACACTTAGGG--------CAGTTTGGTGAAAGG
AGGC-TAAGAAGGTCCAGGCTCCCTAC--CTTCTGTCCCTT
>Dgo
ATCACCATCATACACCCCACGCTCTAGGACTGTTAGTTCTGTCAGGCACGGTTTGGCATT
CTCACAGC--CTGAGGAGT-----------AAGAATAGGAAAAGAGAGA---TTGTAGGA
T------------ACCTAGCTA---------------TAAATCCTGGCTTAAACAAGT-G
GATCTGCAGAAAGACCCGCCTGGATTTC---------CCTCCATGTTCTTCTCTCCACAG
GCCCACATAAAACCTTGGACTGTCCCTTTCCTTTCAGTAGGCACATTTCCTTAGACAAGA
TGTCTATCTGTTGTGCCCTTAGACCATGGA----------TTCCTTGAGAGTGTGTTCTT
GCCTCA-------GAAGCTGGTTCCTGGTTG-TTCAATAT-TGACT-TAAT---------
------------------------------------------------------------
------------------------------------------------------------
------------------------------------------------------------
------------------------------------------------------------
--------------------------------GATAAAGTATGGCCNTAAAATTAAATTC
ATTCATATAGCAT-AAAACCTGACCTACTCAGACCCTCGTCCTGCT--CTGTGGAAGTTT
ACATTCCAAGGTTATGTAA--------CCA--AAAACAT---TGTTGT-TGTTCACCCGT
TGTTCTCAAAAGGGGCCAATGATCCCAGGAAGGTGATATCTTGACTTTC-----------
----AAGTGAGGGAGGTCTGTGCAAGGTCATCTGCCTCAC-TTTCCCCTCCAGAGTTATC
CAGGTCCAGTGGC-AAGAACCAGATCAAGACAA-CTGGTGATGGCTCTGGATGCAATGAG
AGACCTAGACCTTTTAAAGCCAAAGCCA-----------AAGTTTGTCTGAGGCAACACT
```

-----CATTC-CGTTGTCAAGGT--GGTGAAAACTGAGAT-AAAAGATGGCCTAGTTTTG
CCTTCA---------CAGAAGAATCCATCAGGGAAGGAAAGA-------------CT--
-C--TTAGCCTGAGAGT---TAAGAGCACACTTAGGG--------CAGTTTGGTGACAGG
AACC-TAAGAAAGTCCAAGCTCCCTAC--CTTCTGTTCCTT
>Scr
ATCACCATCATACACCCCACGCTCTAGGACTGTTAGTTCTGTCAGGCAAGGTTTGGCATT
CTCACAGC--CTGGGGAGT-----------AAGAATAGGAAAAGAGAGA---TTATGGGA
T------------GCCCAGCTGC---------------AAATCCTGGCTTAAACAAGA-G
GATCTGCAGAAAGGCCCTCCTGGATTTC------------CCATGTTCTTCTCT-CACAG
ACCCATATGAATCCTTGGACTGTCCCTTTCCTTTCAGTAGGCACATTTCCTTAGACAACA
TGTCTATCTGTTGTGCCCTTAGACCATGGA----------TTCCTTGAGAATGTATTATT
GCCTCA-------GAAGC-TGTTCCTGGTTG-TTCAATAT--TGGCTTAAT---------

--------------------------------GATAAAGTATGGCCCTAAAATGAAATTC
ATCCATATAGAAT-AAAACATAACCTACTCAGACCCTGGTCCTGCT--CTGTGGAAGTTT
ACATTTCAAGGTTATGCAA--------CAA--AAAAAAT---TGTTGT-TGTTCATCCTT
TGTTCTCAAAAGGGACCAATGACTCCAGGAAGGTAGTATCTTGACTTTC-----------
----AAGTGAGGGAGGGGTATGCAAGATCACCTGCCTCAT-TTTCCCCTCCAGAACTATC
TGAGTCCAGTGGC-AAGACACAGATCAAGACGA-CTGGTGATGGCTATGGATGCAATGGG
AGACCTAGGCCTTTTAAAGCCAAAGCTAAAGCCA-----AAGTTTGTCTGAGGCAACACC
-----CATTC-CATTGTCAAAGCTAGGTGAGAGTTGAGAT-AAAAGATGGCCTAG-TTTG
CTTTCA---------CAGAAGAATCCATCAGGGAGGGAAAGA-------------CT--
-CTTTTAGCCTGAGAGT---TAAGGGCACATTCAGGG--------CAGTTCGGTGAAAGG
AGGC-TAAGAAGGTCCAGGCTCCCTAC--CTTCTGTCCCTT
>Bil
ATCACCATCATACAACCCATGCTCTAGGACTGTCAGTTCTGTCAGACATGGTTTGGCATT
TTCACAGC--CT-GGGGGT------------------GAGAAAAGAGA---CTATGAGA
TGCCCACAGGTCAGCCCAGCTG-CCTTCCCC-AGCACCAAATCCTGGTTTGCAGAATTGT
CAACCAAAAGACAGCCCTCCTGAATCTGCCCTGCCCCCCCCCA---CCTCCTCTCCACAG
GTCCAGGTAAATTCCTGGGATGTCCTTTTCCTTTCAGCAACAACATTTCCTTTGACAACA
TATTTATCTGTTGGACCCTTAGTCTATGGA----------TTCCCTGAGAATTTATTCTT
GTTTCAGAAGTAGAAAAC-TGTTTCTGGTTA-TTCAGTAC-TGGCC-------------

-----------------------------------TAGTGATAAACTGAAACTGAAATCC
ATTCCTTCAGAATGAAAAGCTGCCCTATTCAGACCTTGGTCCTGAC--CTCTGGATGTGA
ATAGTCCAAGGCTGTGCAA--------CC---AAAGCAT---TACTGT-TGTTCATCCTT
CCTTCTAG----AGACCAATGACATCAGGA-GGTGATGTCTTGACTTGCAACTGAACTGG
ATTTCAGTGAGCCAGAGCTGTGCAAAGTCATCAGTCTCACTCCCT-CCTCCAGAGTGGTC
AGAGTCCAGTGT---AAACATAGGT-AGGATGATC-AGAGATGGCCCAGGATGGAGTGGG
AGACCTTGGTCTTTTTAAGCCAAGGTCTG--CCAGTTCTCAGTTTGTTTGAGGCAAAAAC
-----CATTCAATGATTCAAGATTAGATAAGAACTGAGGT-AAAAGATGGTCTCG-TTTG
CCTTCA---------CAAAAGAATCAAGCAGGGATGTGAAGA--CCCTCAAAGTTTCC--
-T--TTAGGCTGAAAGT---TAAGACCACACT-GGGG--------CAACTGAGGGAGAAA
AGGCTTGAGCAGGTCTGAAATCCCTAC--CTTCTGTCCCTT
>Tro
--CACGGTTTGGCATT
CTCACAGC--CT-GAGGGT----------GGG-AACAGGAAAAGAGAGA--TTTGTGGGA

```
T-----------------GCTGCCC-TCCCCCTGAAGTAAATCCTGGCTTAAAGAGCTGC
CATCTGCA-GAAAACTTTCCTGGATTTG---------CTCCCATGTCCTTCTCTTCACAG
GCACAGGTAAATCCCTGGGATGTCCCTTTCTTTTCAGCAGCAACATTTCCTTAGACAACA
CATCTATCTGTTGTACCCTTAGATCATGAG--ATTTTATTAAACCTGAGAGTTTATTCTT
GCCTAAGAAGCAAGAAGC-TATTCCTGGTTG-TTCAATAC-TGGCCTTGTG---------
------------------------------------------------------------
------------------------------------------------------------
------------------------------------------------------------
------------------------------------------------------------
--------------------------------ATAAAGTATAAC-CCTAAAGAGAAATTC
ATTTATACAGAATGAAAACCTGCCTTA-TCAGACCCTGGTCCTGCC--CTCTGGAAGTTT
ACCTTCCAAGGCTATGCAA--------CA---AAAGAAA---CATTGC-TGTTCATTCTT
AATTCTAGAAGAACGCCAATGACATC-----------GTCTGGACTTGCAAAAGAACTGG
ATTTAAGTGAGGCAGGGCTGTGCAAAATCATCAGCCTCACTCTCT-CCTTCACAGTCATC
AGAGTCCAGTGGC-AAAACACAGATCAGGACTA-CT------------GCATGCAGTGGG
AAACCTCGACCTTTATAAGCCAAGGTCTTTCCCAGGTCTCAGTTTGTCTGTGACAACACC
CATGACATTC-AGTAACCAAGGCTAGGTAAGGAATGAGGT-AAAAG-TGACCCAG-TTCG
CCTTTA---------CAAAAGAATCCATAGGGAAGGGGAAGACTTTCTCAGAGCTTCC--
-C--TTAACCAGAGTGT------------------GG--------CAGCTGGGTGAAAGG
AGGC-TGAAAAGGTCAGGGCTCCCTAC--CTTCTGTCCC--
>Ppe
ATCACCATCATACAACCCACGCTGCAGGACTGTCAGTTCTGTCAGGCACGGTTTGGCATT
CTCACAGC--CT-GGGGGT----------GTG-AAAAGGAGAAAAGGAA---TTGCGGAA
T-----------------GCTGCCC-TCCCCCTGAAGTAAATCCTGGCTGTAAGAACTGC
CATCTGCAGGAAAATCTTCCTGGATGTG---------GCCCCACGTCCTTCTCTCCACAG
GCCCAGGTAAATCCCTGGGATGTCCCTTTCCTTTCAGCAGCAACATTTCCTTAAACAACA
TGTCTGTCTGTTGTATCCTTAGACCATGGG----------CTCCCTGAGAGTTTATTCTT
GCCCAAGAAGGATAAAGC-TGTTCCTGGTTA-TTCAATAT-TGGCCTTGTG---------
------------------------------------------------------------
------------------------------------------------------------
------------------------------------------------------------
------------------------------------------------------------
--------------------------------ACAAAGTACAAC-CCTGAAGTGAAATTC
ATTCATACAGAATGAAAACCTGTCC-AATCAGACCCTGGTCCTGCC--CTCTGGAAGGTT
GCCTTCCAAGGCTATGCAA--------CA---AAAGCAA---TGTTGC-TGTTCATCCTT
TGTTCTAGAAGAAGACCAATGGCATCAGGAGGGTGATGGCTTGACTTGCAAGTGAGCTGG
ATTTAAGTGAGATAAGGCTGTGCAAAGTTACCAGCCTCACTCTCT-CCTCCACAGTAGTC
AGAGTTCAGTGGC-AAAACACAGGTCAGGGCTA-CTGGTGAGGGCCAACGGTGCAGTGGG
AGACCTTGACCTTTCTAAGCCAAGGACTTTCCCAGGTCTCATTTTGTCTGTGGAAACACC
-----CATTC-AGTAGTCAACGCTAGGTAAGAACTGAGGT-AAAAGATGACCTAG-TTCA
CCTTCA---------CAAAAGCATCAATCAGGAAGGGGAAGACTTTCTCGGAGGTTCT--
-C--TCAACCAGAGAGT---TAAGACTGCACT-GGGG--------CAGCTGGGTGAAAGG
AGGC-TGAGCAGGTCCAAGCTCCCTAC--CTTCTGTCCCCT
>Tvu
----------------------------------------------CACGGTTTGGCATT
CTCACAGC--CTAGGGGGT----------AGG-AATAGGAGAAGAGAGA---TTATGAGA
T-----------------GCTCCCC-TCCCCCTGCAGTAAATCCTGGCTTGAAGAACTGC
CATCTGTAGGAAAACCTTCCTGGATTGG---------CCCCTGTGTCCTTCTCTCCACAG
GCCCAGGTAAATCCCTGGGCTGTCTCTTTCCTTTCCGCAGCCACATTTCCTCAGACAAGA
TGTCTATCTGTTGTACCCTTAGACCATGGG----------TTCCCTGAGAGTTTATTCCT
GCCTCGGAAGCACAAAGC-TGTTCCTGGTGG-TTCAATAT-TGGCCTTGTG---------
------------------------------------------------------------
------------------------------------------------------------
```

```
------------------------------------------------------------
------------------------------------------------------------
--------------------------------ATAAAGTACGACTCCTAAAATGAAATTC
ATTCATATAGAACAAAAGCTTGCCCTACTCAGACCCTGGTCCCGCC--CCCTGGAAATTT
ACATGCCAAGGCTATGCAA--------CA---AAAGCAT---TGTTGT-TGTTCATCCTT
CGTTCT---AGAAGACCAATGACATCAGGAGGGTAATGTCTTGACTTGCAAGTGAACTGG
ATTTAAATGAGGCAGGGATGTGCAAAGTCATCAGCCTCCTTCTCT-GCTCCAGAGTCACT
GGAGTCCAGTGGCAAAAACACAGGTCAGGACTA-TTGGTGATAGCCATGGACGCAGTGGG
AGACCTTGGCCTTTTTGAGTCAAGGTCTTTCCCAGATT-CAGTTTGTCCGAGGCAACACC
-----CATTC-AGTAGTCAAGGCTAGGTAAAAACTGAGGTTAAAAGATGGCCTAG-TTTG
CCTTCA---------CAAAAGAATCAGTCCAGGAGGGGAAGACATTT--AGAGTTTCT--
-C--TTAACCAGAGAGT---TAAGACCACAC--CGGG--------CAGTTGGATGAGAGG
AAGG-TGAGCAGGTCCGGGCTCCCTAC--CTTCTGTCCC--
>Mro
ATCACCCTCATACAACCCACGCTCCAGGACTGTCAGCTCTGTCAGGCACGGTTTGGCGTT
CTCACAGC--CTGGGAGGT----------------GGACAGAAGACAGA---CTGTGGGA
T-----------------GCTGCCCTTCCCCTTGCAGTAAATCCTGGCTTGAAGAGCTCC
CAACTGCAGGAAAACCTTCCTAGATTTG---------CCCCCATGTCCTTTTCTCCATAG
GCCCAGGTAAATCCCTGGGCTGTGCCTTTCCTTTCAGCAGCAACATTTCCTTAGTCAACA
CATCTGTCTGTTGTACTCATAGACCATGAG----------TTCCCTGAGAGTTTTTTTTG
TN-TAAGAAGCACAAAGC-TGTCCCTGGATG-TTCAATAC-TGGCCTTTTT---------
------------------------------------------------------------
------------------------------------------------------------
------------------------------------------------------------
------------------------------------------------------------
--------------------------------GATACAGCATGACCCTAAAATGAAATTC
ATTCATATGGAATGAAAGCCTGCCCTCCTCAGACCCTGGTCCTGCC--CTCTGGAAGTTT
ATATTCCAAGGCTATGCAG--------CA---AAAGCAT---TTGTGGTTGTTCG-----
-------------------TGACATCAGGAGGGTGATGCCCTGACTTGCGAGTGAACTGG
ATTTAAGTGAGGCAGGGGTGGACAACATTATCAGTCTCACTCTCTCCCTCCAGAGTCATC
GGAGTCCAGTGGC--AAAACATGGTCTGGACGA-CTCATAATGGCAAAGGATGCAGTGGG
AGACCTTGACTTTTTTAAACCAAAGTCTTTCCCAGGTCTCAGTTTGTCTGAGGCAACACC
-----CATCC-AATAGTCAAGGCCAGGTAAGAACAGAGGT-AAAAGACAGCCTAG-TTTA
CCTCCA---------CAA-----TCAATCCA--------------------AGTTTCT--
-C--TTAACC--AGAGT---TAAAACCAC-------------------------------
--------------CCAGACTCCCTAC--CTTCTGTCCCTT
```

MARKER 93

```
>MIR3_MarsA
------------------------------------------------------------
----------------------------------------CCTCTCTGGGCC--------
--TCAGTTTCCTCA----TCTGTAAAATGAGGGGGTTGGACTAGATGACCTCTAAGG-TC
CCTTCCA---------GCTCTAAAT-----CTATGATCCTATGA---TCCTATGA-----
------------------------------------------------------------
------------------------------------------------------------
------------------------------------------------------------
------------------------------------------------------------
------------------------------------------------------------
------------------------------------------------------------
------------------------------------------------------------
------------------------------------------------------------
------------------------------------------------------------
------------------------------------------------------------
------------------------------------------------------------
------------------------------------------------------------
------------------------------------------------------------
------------------------------------------------------------
------------------------------------------------------------
------------------------------------------------------------
------------------------------------------------------------
------------------------------------------------------------
------------------------------------------------------------
------------------------------------------------------------
------------------------------------------------------
>WSINE1
------------------------------------------------------------
------------------------------------------------------------
------------------------------------------------------------
------------------------------------------------------------
------------------------------------------------------------
------------------------------------------------------------
------------------------------------------------------------
------------------------------------------------------------
------------------------------------------------------------
------------------------------------------------------------
---------------------------------------TTTTTTTTTTTTTTGAGGCAA
TTGGGGTTAAGTGACTTGCCCAGGGT-CACACAGCTAGTAAGTGT-------CTGAGGCT
GGATTTGAACTCAGGTCCTCCTGACTCCAGGGCTGGTGCTCTATCCACTGCACCACCTAG
CTGCCCCTC---------------------------------------------------
------------------------------------------------------------
------------------------------------------------------------
------------------------------------------------------------
------------------------------------------------------------
------------------------------------------------------------
------------------------------------------------------------
------------------------------------------------------------
------------------------------------------------------------
------------------------------------------------------------
------------------------------------------------------------
```

>WALLSI1

-TTGCTCATATGTTG-TGTTTGTCCTTCGTTCTCGAAGAGGACCATGACATCAGGGAGAT
GATGACATGACTTGCAGTTGACTTTGATTTGA-GTGAGGG---AGGGCTGT-GCAAGGTC
ACCAGCCTCACTTTCTTCTCC---------------------------------------

>Mdo
ACAGGCCACCTAATG--GTAATCCCTGCGTGCTGCC---------------------CCC
AGGGAGGCGCCGGGTG--------------CTGGG----------CCTGCCTCTGCT---
-ACAACCTTCCCTC-----CTCGAAGGCGAGG-CTGCGGCCTCAGC--CTTAGGGAGGCT
CCTTCC----------CCCTCGCAGG---------GCCCTGAT----CTTGC--ATGTCT
CTCTCCTCACTC---TCACTGGTTTGACTTGCACAGGCCCCGGAAGTTTAAGTGTTCCCA
GGTCAGCAGTGACTTTT-GGCCTACTTGAAAGAGC--TTTGGTTCCCTAGGAGAGAGGA-
--GCATTTCAGGTCCCTCAGCAGGATGGAACAGGTTGAATGTTTTTATC---TCCTCTCC
-CTGTCAAGTGTC--ACAGATA------TGTTGGCAAGGCCAAATGGGAGTTCTTG-GTA
TGAGA--AAGCAGGAAAAGCAA-GCAAAGGCCTGGTGAG---------------------
----------GCCAATTCCCAAGCCCGGCAGGGAT-CAGGGCCTCCGGCTTCAGTTCCGG
GGGGAGGGGGATTTAAAAGGGAAAGAACT-CCAGCCTT----------------------

---TTTTC
CCGGGCATTTTCCCAGGTTTACACA-CAGACAGCCCCCCCCCCCCCCGGGCACTCTAGCC
CTCAATCTGGCAAC--------------AGAGGCCCTAGAGAAGCCTTCCC-AGAGC-CG
GA--
---GTCAGGGAGCC
ACATCATTGGTGGCACCACTCTGACCCAGC----------------------------CC
TCCACTTTGGTAGAG--CCCT--GAGTTGGGAGATAATTTCTCATTAAACTCACCACAGT
CTCCTCTGATGCCATTGCAG--ATTGGAAATATAGCTCCGGATTCATTAATGCA

>Dvi
ACAGGCCTCCTAATG--GTAATCCCCGCAGGCTGCC------------------------

----TCCGTCCCCA-----CTAGCAGGCGAGGCTGC-GGACTCGG---CCTGCAGAGGCT
CCTTT-----------GGTCTCCTG--------GGGCCCTGGT----CTGGTGCGG----
CTCTCCCACTC---TCACTACTTTGATTTGCACAGGCCC-GGACGTGCATACATTCCCA
GCTCACCAGTGACTTTG-GGCCTACTTGGAAGGGC--TTTG-CTCCATAGCACAGGGA--
--GCATTTCGGGTCCCTCAGCGGGTAGTAGCAAGTCGAGGCTGTTTATC---TCCTCCCC
-CCATCAAGCCTCACAGAGATA------CCTTGGCAAGGCCAAATGGGAGTTCTTG-GTA
TGGGG--AAGCAGGAAGAGCAA-GCAGACGCCTGGCGAG---------------------
----------GCCAGCGCCTGAGCCTGCAGTGATCAGCACCTGCTGATCTCTTCCCTGGC
CCTCTCCCGGGCTCACAC--

----------------------GGGGCCGGGCCTCCCTCTGGGCACTCCCCAGGGGAGCC
CTGGTTGCTGTGATCTGGCGTC------AGAGGCCTCAGAGAAACCTTCCC-AAAGC-CT
AG--
---TCAGGGCCGGC
AGCTAGCAGAGCCGCCCTGACCCAGCCCTCCGCCCTCCGCCCTGGCCGTGGCCGA----
----------------GCCCT--GGG-TTGGAGATAATTCCTCATTAAACTCCCC--CGT
CTCCTCTGATGCCATTGCAG--ACTGGAAATACAGCTCGGGATTCATCAGTGCA
>Mnu
-ATTGCCTCCAAATG--GTAATCCCTGTGGGCTGCC------------------------

----TCTGTCCCCA-----CTAGCAGGCGAGGCTGC-AGACTCGG---CCTGCAGAGGCT
CCTTT-----------GGCCTCCCG--------GGCCCCTGGT----CTGGCGTGG----
CTCTCCCACTC---TCACTGCTTTGACTTGCACAGGCCC-GGACGTGCACGCGTTCCCA
GCTCACCAGTGACTTTG-GGCCTACTTGGAAGGGC--TTTG-CTCCATAGCACAGGGA--
--GCATTTCGGGTCCCTCAGCGGGTAGTAGCAAGTCGAGGCTGTTTATC---TCCTCCCC
-CCATCAATCCTCACAGAGATA------CCTTGGCAAGGCCAAATGGGAGTTCTTG-GTA
TGGGG--AAGCAGGAAGAGCAA-GCAGACGCCTGGCGAG---------------------
----------GCCAGCGCCTGAGCCTGCAGTGATCAGCGCCTGCTGATCTCTTCCCTGGC
CCTCTCCCGGGCTCACAC--

----------------------GGGGCTGGGCCTCCCTCCAGGCACTCCCCAGGGGAGCC
CTCGTTGCTATGATCTGGATCTGGCGTCAGAGGCCTCAGAGAAACCTTCCC-AGAGC-CT
AG--
---TCAGGGCCGGC
AGCTAGCAGAGCCGCCCTGACCCGGCCCTCCGCCCTCTGCCCTGGCCGTGGTGGGG---
---------------------------TTGGAGATAATTCCTCATTAAACTCCCC--CAT
CTCCTCTGATGCCATTTCAG--ATTGGAAGTATAGCAANCAGGAATTCTGGATC

```
>Rra
GATTGCCTCCAAATG--GTAATCCTTGATTGTTTCC-TTCCATTAGAAGTAGCATGATGG
TAACACTGCCCA----------CAGAAGACCTGGGTT---CCAGTCCTGGCTCC-ATA--
-ACTGCCTACCCTA----GCTATCCACTGAGGGGTGAGGACTTGATGACAA--AAGG-TC
CCC-CCACCCCCTACACCTCTCTATGCAGGCTGGGATCCTGTAA---TTCC------TCT
TTTACCTCATTTCCATCACTGTTTT-ACTCACTCAGGCCCCATG---TTAAGTCTTCCTA
GGTTAGAGCCGACTTTT-GGACTACTTTGAAGAGC--TTTGGCTCAGGAA--TAGAGAGT
TAACATTTCAGGTCACTTAGCAGGATGTAACAGGTTGAAACTCTAATTCATTGCCTCTTT
TCCCTGATT-----GTATCATAAAGAT------------------GAGAGCACCTAAGTA
-GGGG-AAAGGAGGAGAGTCAGTTCTAAAGACTGGATAGACAAGTTCAGAAGTACTTTTG
TGATCGGTGTCAGTCTGCTTCAGCCTAAGGAAGCAGTGGGAATCAAA--TGCAAAACAAC
CATTACCCCTTCTG-AGGGGCTGAGGGTT--------------------------------
------------------------------------------------------------
------------------------------------------------------------
---------------------------------------------------CCTTCCGGG
GCATTG--CTTAGGCTCA-GTGATGCACAAG-TACCTTCCAGCTATTT---GG---GT--
------------------------------------------------------------
-------------------------------------------AGCAAAAGCAGAATAGG
AGTCGATCCAGGCACTCATACCTTTGAACTCCACATTAAAGA------CTGC--------
------------------------------------------------------------
----------------------------AGAAACATGGGAGAAATCTCTTT-AGAGGAGT
AGTTAGTGATCTGAAATATTTGCAGGGG-TCATT---GGC-ACTGANAAGGTGCCA-ACC
AC-----------------------TGACCTAGCCCTCCTGGAACGGTT-----------
---------------------------------------------------------TGA
ACCACTTTACTAGGG---TCACAGGTTTTCTANATCCTTTCTGAATAAACTCNCC--AGT
GTCCTCTGATGCCATTTCAA--ATTGGAAGTATAGCAATCT-------------
>Cfu
NAAGGCCTCCAAATG--GTAATCCTTGATTGTTTCC-TTCCATTAGAAGTAGCATGATGG
TAACACTGCCCG----------AAGAAAACCTGGGTT---TCAGTCCTGGCTCC-ATA--
-ACTGCCTACCCCA----GCTATCCACTGAGGGGTGGGGACTTGATGAC---------CC
CCCTGCA---------CCTCTCTTTGCAGGCTGGGATCCTGTAA---TTTC------TCT
TTCTGCTCATTTC-ATCACTGTTTT-ACTCACTCAAGCCCCATG---TTAAGTCTTCCTA
GGTTATAGACGACTTTT-GGCATACTTTGAAGAGC--TTTGGCTCAGGAA--TAGAGAGT
TAACATTTCAGGTCACTTAGCAGGATGTAACAGGTCGAGACTCTAATTCATTGCCTCTTT
TCCCTGATT-----GTATCATAAAGAT------------------GAGAGCACATAAGTA
-GGGG-AAAGGAGGAGAGTCTATTCTAAAGACTGGATAGACAAGTTCAGAAGTACTTTTG
TGATTGGTGTCAATCTGCTGCAGCCTAAGAAAACAGTGGGAACCAAA--TGCAAAACAAC
CATTACCCCTTCTTAAATGGCCGAAGGTT-------------------------------
------------------------------------------------------------
------------------------------------------------------------
---------------------------------------------------CCTTCCTGG
GCATTG--CTTAGGCTCA-CTGAAGCACAAGACACCTTCCAGCTATTT---GG---GT--
------------------------------------------------------------
-------------------------------------------AGCAAAAGCAGAATAGG
AGTCGATCCAGGCACTCATACGTTTGAGCTCCACATTAAAGA------CTGC--------
------------------------------------------------------------
----------------------------AGAAACAT-GGAGAAATCTCTTT-AGAGGAGT
AGTTAGTGATCTGCAATATTTGCAGGGG-TCATT---GGC-ACTGAGAAGGTGCCA-ACC
AC-----------------------TGACCTAGCCCTCCCGGAACGGTT-----------
---------------------------------------------------------TGA
ACCACTTTAGTAGGG--TCCACAGGTTTTCTAGATCCTTTCTGATTAAACTCACT--AGT
GTCCTCTGATGCCATTTCAG--ATTGGAAGTATAGCACTGGATTCGTTAATGCA
```

>Dgl
GATTGCCTCCAAATG--GTAATCCCTGTTTGCTTCC-CTCAGCTAGAAATAGCAATAGTG
GCACCACTCCCAGATGTTGAGACAGAGGACCTGGGTT---CCAGTCCTGGCTCTGATA--
--CTACTATTCATA----TCTATAAAAGGA-----GAGG----TGTGATTACTAGGG-TC
CCTACCA---------CCTCTAAATA----CCATGATCCTTTGA---CTTC----CGTCT
CTCTTCTCACCAAAGTCACTGTTTTTACTCTTTCAGGCCTCATGGGTTTAAGTCTTCCCA
GATCAGAACTGGCTTTTTGACCTACTTTAAAGAGCTTTTTGGCTCAGTATCAGAGAATGT
-AACACTTCAGGCCATTTAGCAGGATGTAACAGGTTCAGTCCTTAATCCATTGCCTCCTC
T----CTGTGTCTAGT---ATAAAGATACCTTGGCAGGGTGAAATGGGAGTACTTC-ATA
-GGGG-AAGATAGGAAAAGCAATTCAAAAGACTGGAAAGGCTAGTTCACCAACACTTTTG
TGATCAGTGCCAATCTGCTGCAGCTCAAGGCAGGAACGTGATCCAAAA-TGCAAA-CAAC
CCTTACT-----------GGCCTAAGGTT---------CTTTTTTTTTTTTGTGGGGCAA
TGGGGATTAAGTGACTTGCCCAGGGT-CACACAGCTAGTAAGTGTCAAGTGTCTGAGGCC
GGATTTGAACTCAGGTCCTCCTGAATCCAGGGCTGGTGCCTTCTCCACTGCACCACCTAG
CTGCCCCCT-GGCCTAAGGTTC---TTAACTG------GCCTAAC------TGCTGCTGG
GCATTTTCCCA-GGCTTA-CTGATGGACA-GATGCCTTCCAGCCATTTT--GG----T--

---AGC-AAAGCAGTGTCAA
-CTAGGTCCAG---

-------------------------------ATGCTCACCTATAAAGAA-GTATCAAACT
GTTGGCCAGATGATGACTTCCTCTCTGATCCAGTCCTCCAGGAAGGGTT-----------
---TGA
AGGATTACTTTGATAGAACCTCAGGTTTTTAAGATACTTTCGAGTTAAACTTACCACTGT
TTCCTCTGATGCCATTTCAG--ACTGGAAGTATAGC------------------
>Pta
ACAGGCCTCCGAATG--GTAATCCTTGTTTGCTTCC-CTCAGCTAGACCAGGGATCA-TG
GCACTCTTCCCAGATGGTAGAGCTGAGGGCTTGGGTT-----TCTCGAGGCTTTGATA--
--CCACTTTCCTTA----TCTGTAAAATGAAGGTTGTGGATTCAGTGATTACTGAAG-TC
CTTCTCA---------CCTCTAAATG----CCATGATCCTTTAA---CTTCTCTCTGTCT
CTCTTCTCACCATAGTCACTGTTTGTACTTTCCCAGGGCCC------------------A
GGTCAAAACTGGCTTTT-GACCTATATTGTAGAGC--TTTGGCTCAGTGTCAGAGAGAAT
-AATACTTCAGGTCATTTAGCAGGATATAACAGGTTCAGTCTTTAATCCATTGCTTCTTC
T----CTATGTCTGGTGTCATAA----ACCTTGGCAGGGTGAAATGGGAGTACTTC-ATA
-GGGG-AAGGTAGGAAAAGTAGTTCAAA-GACTGGAAAGGCTAGTTCATCAACACTTTTA
TGATCAGTATCATTCTGCTGTAGCTCAAGGC-------TGATCCAAAA-TGCAAA-CTGC
CTTTACT-----------GGCCTAAGGTT-------------------------------

------------------------CTTAACTG--CTTGGTCCAAGGTT---ACTTCCTGA
GTATTTTTCCA-AGCCTA-CTGATGGACA-AATGTCTTCCATCTATTT---GGATGGT--

---AGT------AATGTAGG
-CTCAATCTAAGCTCTTGCCAATTA--GCTCCACATTAAAGATAGGTTTGGC--------

----------------------------AAAAGCTTTAGTGAAATCTCCTTTAGAAGAAT
AGTCAGTGATGTGCATTATTGGCAAGCAGTTATTAGTAGC-ACCAAG---GTCTCAAATT
ATTGGCTGGAGGGATACTTCCTCTCTGATCCAACCCTCCAGAAAGGATT-----------
---TGA
GCCCC---TTTGATAG--CTTCAGGTTTTTAAGATAATTTCTAGTTAAACTCACCACTGT
TTTCTCTAATGCCATTTCAG--ATTGGAAGTATAGCTCTGGATTTATTAATGCA

```
>Dgo
ACAGGCCTCCTAATG--GTAATCCTTGTTTGATTCC-CTCAGCTAGACTAGGGATCA-TG
GCACTCTTCCCAGATGGCAGATCTGAAGGCTTGGGTT-----TCTCGAGGCTCTGATA--
--TAATTTTCCTTA----TCTGTAAAATGAAGGTTGTGGATTCAGTGATTACTGAAG-TC
TCTCTCA---------CCTCTAAATG----CCATGATCCTTTAA---CTTTTCTCTGTCT
CTCTTCTCACTTCAGTCACTGTTTGTACTTTCCCAGGGCC---------AAATCTT----
-------ACTGGCTTTT-GACCTACATTGTAGAGC--TTTGGCTCAGTGTCAGAGAGAAT
-AATATTTCAGGTCATTTAGCAGGATATAACAGGTTCAGTCTTTAATCCATTGCCTCTTC
T----CTATGTCTGGTGTCATAA----ACCTTGGCAGGGTGAAATGGGAGTACTTC-ATA
-GGGG-AAGGTAGGAAAAGTAGTTCAAA-GACTGGAAAGGCTAGTTCATCAACACTTTTG
TGATCAGTGCCATTCTGCTGTAGCTCAAGGC-------TGATCCAAAAATGCAAA-CAGC
CCTTACT-----------GGCCTAAGATT--------------------------------
------------------------------------------------------------
------------------------------------------------------------
------------------------CTTAACTG--CTTGGTCCAAGGTT---ACTTCCTTA
GTATTTTTCCA-AGCCTA-CTGATGGACA-GATGTCTTCCAGCTATTT---GGATAGT--
------------------------------------------------------------
-------------------------------------------ATT------AATGTAGG
-CTCAATCTAAGCTCTTGCCAATTA--GCTCCACATTAAAGATAGATTTAGC--------
------------------------------------------------------------
----------------------------AACAGCTTTAGTGAAATCTCCTTTAGAAGAAT
AGTCAGTGATGTGCATTGTTAGCAAGCAGTTATTAGTAGC-AC---CAAGGTCTCAAATT
ATTGGCTGGAGGGATACTTCCTCTCTGATCCAACCCTCCAGAAAGGAGT-----------
---------------------------------------------------------TGA
GCCTC---TTTGATAG--CTTCAGATTTTAAAGATAATTTCTAGTTAAACTCACCACTGT
TTCCTCTAATGCCATTTCAG--ATTGGAAGTATAGCTCTGGATTTATTAATGCA
>Tro
ACAGACCTCCCASTG--GTRATTCTKATGTGCTACC-CTCCATTAGAAGTAGAGAG--TG
GCACTGCTWCTAGGTGTTGGATCGGAGGGCCTGGATT---CCAGTCCTGGCTCTGATC--
--TTACCTCACCTG----TCTAGAAAATGAGGGCTATGGATCCAGTGATTCCTAAGGTTC
CCTTCTA---------CCTCTAAATG----CCCTGATCCTTTCA---CTTCC--------
---MTCTCAT---AGAC------------------GGCCCAGTAGGTTTAAGTCTTCTCA
GGTCARAACTGGCCTTT-GACCCACTTTAAAGAGC--TTTGGCTCAGCATTACAGGGAGT
T----------AACACTTAGCAGGATGTAACAGGT--AGTCTTTAATCCATTGCCTCCTA
T----CTGTGTCTG----CATAAAGTTACCCTGGCAGGGTGAAATGGAAGTACTCC-ATA
TGGGGTAAAGTAGGAGAAGCAATTCAAA-GACTGGAAAGGCTAGTTCACCAACACCTTTG
TGATCAGTGCCAAACTGCTGCAGCTCAGGGCAGGAACGTGATCCAAAA-CGCAGA-CAAC
CCTCACT-----------GGCCTGAAGTT--------------------------------
------------------------------------------------------------
------------------------------------------------------------
------------------------CCTTCCC-----GGGCCC------------------
-----TTTCCCAGGCTTA-CTCACGGACA-CGGGCC-------------------------
----------------------------------AAAGAGGACCATGACACCAGGGAGGT
GATGCCATGACATGCAAGTGAATTGGATTTAA--CGAGGG---AGCAAA-GCAGTACAGG
-CTCAGTCCAAGCAGTCACTAATTA--GCTTCACAGTAAAGATAGATCTGACTTC-----
------------------------------------------------------------
----------------------------AGAAGCTTTA-GGAAGGCTCCAT-AGAGGGAT
AGTTAATAG--TGTGGTATTGGCAGGGGGTCACTGGTGGC-ACTGAGAA-GTCTCAAGCT
ATTGGCTGGA---------------TGATACTTCCCCCCAGGAAGGGTT-----------
---------------------------------------------------------TGA
ACCAC---TTTGATAGAACCCCAGGTTTTTAAGAGAATTTCTAATTAAACCTACCACTGT
TTCCTCTGATGCCATTTCAG--ATTGGAAGTACAGCTCTGGATTCATTAATGCA
```

```
>Mro
ACAGGCCTCCCAATG--GTAATCCTCATGTGCTTCCCGTGTGCTACAAGTAGAGAGAGTA
GCACTGGTCCCAGATGTT--------------GGGTT---CCAGTTCTG-CTCTGAYC--
--CTACCTTCGCTG----TCAAGAAAATAAGGGGTGTGGATTCACTGATTACTAAGG-TC
CCTCCCA---------CCTCTAAATG---------ATCCTTTCA---CTTCC--CTCTGT
CTCTTCYCACCATAGTTACTGTTTTTCCTCTCTCAGGCCCTATAGGTTTAAGTTTTCCCA
GGTCAGAACYGGCTTTT-GACCTACTTTACAGAAC--TTTGGCTCAGTATCAT-------
-------------CACTTAGCAGGATGTAACAGGTTTAGTCTTTAATCCATTGCCTCCTG
T----CTGTCTCTGATATTCTAAAGTTACCTTGGCAAGGTGAAATGGGAGTATTTC-TTA
TAGGGTAAAGTAGGAAAAGCACTTCAAA-GACTGGACAGGCTGGTTCACCGACACTTCTG
TGATCAGTGCCAGTCTGCTCCCACTGAGGGCAGGAATGTGATCCAAAA-TGCAAA-CAAC
ACTTACT-----------GGCTTAACGTT-------------------------------
------------------------------------------------------------
------------------------------------------------------------
------------------------GCTTCCT-----GGGCAT------------------
-----TTTCCCAGGCTTA-CTGATGGACT-GAATGG--CCATGT-------GG--GGT--
-----------G---GTGTTTGTCCTTTGTTCTCAAAGAGGACCATGATGTCAGGGAAGT
GATGCCATGACACACAAGGGAATCAGATTTAA-GTGAGAG---AGCAAA-GCAGTATAGG
-CTCAGTCCATAGTCTCAACAATTA--GCTTCACATTAAAGACAGATCTGGCTTC-----
------------------------------------------------------------
----------------------------AGAAGCTTTAAGGAAATCTTCT---GAGGAAT
AGTCATTAA--TGCGTTATTGGCAGGGGGTTATTGGTGGC-ACTAAGAA-GTCCCAGACT
GTTGGCTGGG---------------TGATACTT-CCTCCAGGAAGGGTT-----------
---------------------------------------------------------TGA
ATCAC---TTTGATAGAACCCTAGGTTTTTAACATAATGTCTAATTAAACTTACCACTGT
TTCTTCTGATGCCATTTCAG--ATTGGAAGTATAGCTCGGGATTCATTAATGCA
>Ptr
ACAGGCCTCCCAATG--GTAATCCTCATGTGCTTCCTGTGTGCTACAAGTAGAGAGAGTA
GCACTGGCCCCAGATGTT--------------GAGTT---CCAGTTCTG-CTCTGATCCT
TCCTACCTTCCCTG----TTGAGAAAATAAGGGGTGTGGATTCACTGATTACTAAGG-TC
CCTCCCA---------CCTCTAAATG---------ATCCTTTCA---CTTCCTTCTGT--
CTCTTCTCACCGTAGTTACTGTTTTT-CTCTCTCAGGCCCTATAGGTTTAAGTTTTCCCA
GGTCAGAACTGGCTTTT-GATCTACTTTATAGAGC--TTTGGCTCAGTATCGT-------
-------------CACTTAGCAGGATGTAACAGGTTTAGTCTTTAATCCATTGCCTCCTA
T----CTGTGTCTGATATTCTAAAGTTACCTTGGCAGGGTGAAATGGGAGTATTTC-TTA
TAGGGTAAAGTAGGAAAAGCACTTCAAA-GACTGGGCAGGCTGGTTCACCAACACTTCTG
TGATCAGTGCCAGACTGCTGCCACTGAGGGCAGGAATGTGATCCAGAA-TGCAAA-CAAC
ACTTACT-----------GGCCTAATGTT-------------------------------
------------------------------------------------------------
------------------------------------------------------------
------------------------GCTTCCT-----GGGCAT------------------
-----TTTCCCAGGC-TTCCTGATGGACT-GAATGG--CCTTGT-------GG--GGT--
-----------G---TTGTTTTTCCTTCCTTCTCAAAAAGGACGATGATATCAGGGAAGT
GATGCCATGATACGCAAGGGAATCAGATTTA--GTGAGAG---AGCAAA-GCAGTATCAG
------TCCACACTCTCAACAATTA--GCTTCACATTAAAGATAGATCTGGCTTC-----
------------------------------------------------------------
----------------------------AGAAGCTTTAAGGAAATCTCCC---GAGGAAT
AGTCAATAA--TCCATTATTGGCAGAGGGTTATTAGTGGC-ACTAAGAA-GTCTTAAACT
GTTGGCTGGG---------------TGATACTT-CCTCCAGGAAGGGTT-----------
---------------------------------------------------------TGA
ACCAC---TTTGATAGAACCTTAGGTGTTTAAGATAATGTCTAATTAAACTTACCACTGT
TTCTTCTGATGCCATTTCAG--ATTGGAAGTATAGCTCTGGATTCATTAATGCA
```

```
>Vur
ACAGGCCCCCTAATG--GTAATCCTTGTGTGCTTCC-CTCAACTAGAAGTGAC-------
CGAGTGCTCCCAAATGTTGAGTCAAAGGATCTGGGTT---CCA-TCTTGGCTCTGCTC--
--CTACCTTCCCTA----TCTAGAAAGTGAAGGGTGTGGATTTAGTGATTACTA-AGTTC
CCTTCCA---------CCTCTAAATG----CTGTGATCCTTTCA---TTTCTCT--CAGT
CTCTTCTCACCATAGTCCCCATCTTTACTCTCTTAGGCCCCATCCATTTAAGTTTTCCCA
GGTCAGAATTGGCTTTT-GACCTACTTTAACGAGC--TTTGGCTCAGTATCAGAGAGAA-
---CACTTCAGGTCGCCTAGCAGGATGTAACAGGTTCAGTC---AACCCATTGCCTCCTT
CCTCTCTGTGCCTAGTATCATAAAGATGCCTTGGCAGGGTAAAATGGGAGTACTTC---A
TAGGGTAAAGTAGGGAAAGCAATTCAAA-GACTGGCAAGGCAAGTTCACCAGCACTTCTG
TGATCAGTGCCAGTCTGCTGCAGCTCAGGGCAGGAAAGTGATCCAAAA-TGCAGA-CAAC
TCTTACT-----------GGCCTAAGATT-------------------------------
------------------------------------------------------------
------------------------------------------------------------
------------------------CCTTCCT-----GGGCAC------------------
-----TTTCCCAGACTTTGCTGATGGACT-GATGCCCTCTAGACATTTGGGGGGGGGT--
-----------GTTGTTGTTTGTCCTTTGTTCTTAAAGAGGACCATGACATCAAGGAGGT
GATGCCATGACATGCAAGTGAATTGGATTTAA-GTGAGGG---AGCAAA-GCAGTATAGG
-CTCAGTCCAAGCGCTCACCAATTA--GCTCCACTTCAAAGATAGATCTGGCTAC-----
------------------------------------------------------------
----------------------------AGAAGCTTTAGTGAAATCTCCAT-AGAGGAAT
AGTAAGTAA--AGCATTACTGGCAGGGGGTCATTGGTGGC-ACTGAGAA-GTCTCAAACT
GTTGGCTGGATGATACATCCTCTC-TGATCCAGCCCTCCAGGAACGGTT-----------
---------------------------------------------------------TGC
ACCAC---TTTGGTAGAGCCCTGGAGTTTTAAGATAATTTCTAGTTAAACTCATTACTGT
TTCCTCTGATGCCATTTCAG--ATTGGAAATATAGCTCTGGATTCATTAATGCA
```

MARKER 94

```
>WALLSI1A
------------------------------------------------------------
------------------------------------------------------------
------------------------------------------------------------
------------------------------------------------------------
------------------------------------------------------------
------------------------------GGCCAGAAACC---------CTGAGGGTCT
TCCCCTCCCAGATTGATTTTT---------------------------------------
------------------------------TTTTTTTTTTTGACTAGGTAAAAGAGGCCA
-TTCTTTGCCTCATTTCTTACCTAGCCTTAATCACTGAATGGGTG--TTGCCTCAGTCAA
ACTGAGACCTGNGAAAGACCTTAGCTTAAAAAGGCCAAGGTCTCCCATTGCATCCTGGGC
CATCTCCAGTCATCCTGATCTATATCTTGCCACTGGACCCAGATGGCTCTGGAGGAGAAA
GTGAGGCTGGTGACTTTGCACAGCCCTCCCTCACTTAAATCCAATTCACTTGCAAGTCAT
GKCATCACCTCCCTGATGTCATGGTCCTCTTTGAGAACAAAGGAC---------------
---AAACAACAA------------------------------------------------
------------------------------------------------------------
------------------------------------------------------------
----------------------
>Mdo
ATTCGTTAACGTCACCATTGAAAGGGGTGACCACATTGTCCCCTCTTCTCTGCTGCATTT
TTTCCAGCAATTTATCTAGATCATCAC--CTGTTAAAGAAATGATGAC-AGAGAGAAGAG
TGTT-ATAAGGAATGATGTATCCCCAAA----------CCAGGGCGACA-----------
-----------------------AGAGTTATGCTAGTTGGAGCTGGCACTCAGAATTGAC
T-GACAG-TATTCTGTCTTTGTTTGACAAGGAG-TCACG-ACTGGCTGCCTCTGTGGTAG
TTTCCTTGCAAGGCC---------------------------------------------
------------------------------------------------------------
------------------------------------------------------------
------------------------------------------------------------
------------------------------------------------------------
------------------------------------------------------------
------------------------------------------------------------
------------------------------------------------------------
----------------------ATGAAC---AAAATGCTAAGAAGTGGA-CG-CTATGAA
CCTATAGGATCAGTAAAGAAAGCCCAAGTCTC---ATGGATAGGTTATAACAGAACATTA
AGGAGTCATGCAGCATTTTACTAGGAGGGTTGCTGGCTTATGTATCCACTTAC--CTAAC
GATGTGTTTTTGAAATGGGATG
>Dvi
ATTCATTAACATCACCATTGAAAGGGGTAACCACATTGTCCCCTCTTCTCTGCTGCATTT
TCTCCAGCAATTTATCTAGATCATCAC--CTGTTAAAGAAATGATG---AGAGAAAAGAG
AATT-ATAAGAAAAGATGCATCCCCAAAACAGAAGGTCCCAGGGTGACA-----------
-----------------------AGAATTATGCTAGTTGGACCTGGTACTCAGAATTGAC
T-GCTGGGCTTTAAGTC-TTGTTTGATAAGGGG-TCACG-GCTGGCTGCCTCTGTGGTAG
TTTCCTTGCAAGACC---------------------------------------------
------------------------------------------------------------
------------------------------------------------------------
------------------------------------------------------------
------------------------------------------------------------
------------------------------------------------------------
------------------------------------------------------------
------------------------------------------------------------
----------------------ATG-ACAAAAAAATGCTAA---GTGGA-CA-TTCTGAA
```

```
CCCACAGAATCAGTAAAGAAAGCACAAATGAGACCATGGATAGGTTATAACAGAACACTA
AGGAGTAATGCAGAAGTTTTCTAGGAGGGTTGCTGGCTTATGTATCCACTTAC--CTAAA
GATGTATTTTTGAAATGGGATG
>Mnu
ATTCATTAACATCACCATTGAAAGGGGTAACCACATTGTCCCCTCTTCTCTGCTGCATTT
TCTCCAGCAATTTATCTAGATCATCAC--CTGTTAAAGAAATGATGAC-AGAGAAAAGAG
AATT-ATAAGAAAAGATGCATCCCCAAACCAGAAGGTCCCAGGGTGACA-----------
-----------------------AGAATTATGCTAGTTGGACCTGGTACTCAGAATTGAC
T-GCTGGGCTTTAATTC-TTGTTTGATAAGGGG-TCACG-GCTGGCTGCCTCTGTGGTAG
TTTCCTTGCAAGACC---------------------------------------------
------------------------------------------------------------
------------------------------------------------------------
------------------------------------------------------------
------------------------------------------------------------
------------------------------------------------------------
------------------------------------------------------------
------------------------------------------------------------
----------------------ATG-ACAAAAAAATGCTAA---GTGGA-CA-TTCTGAA
CCCACAGAATCAGTAAAGAAAGCACAAATGAGACCATGGATAGGTTATAACAGAACACTA
AGGAGTAATGCAGAAGTTTTCTAGGAGGGTTGCTGGCTTATGTATCCACTTAC--CTAAA
GATGTATTTTTGAAATGGGATG
>Rra
ATTCATTAACATCACCATTGAAAGGGGTCACCACATTATCCCCTCTTCTCTGTTGCATTT
TCTCTAGCAGTTTATCGAGATCATCAC--CTGTTCAAAAAATGATGATAAAATACAGGAA
GAAT-ATAAGGAATAAGACATTCATCAACCACCCGGCCCCGGGGTGACG-----------
-----------------------GTGGTGATGCCATTTAGGCTTGGCATTCAGAACTGAT
T-GCCAGGTATTGAGTCTTTTTTTGACAAGGAGTTCACA-GCTGACTGCCTCCATGGCTA
TTTCCTTGTG-GACC---------------------------------------------
------------------------------------------------------------
------------------------------------------------------------
------------------------------------------------------------
------------------------------------------------------------
------------------------------------------------------------
------------------------------------------------------------
------------------------------------------------------------
----------------------ATTAAC---AAAGTGCTAAGAAGTGGA-CA-TTATGAA
CCCATGGGATCAGCAAAGAAAGCACAAGTCTC---ATGGGCAGGCTATGACAGAACACTG
AGAAGTAATTCAGAAGTTTATTCGGAGGGCTGCATATTTCTGAATCCACTTAC--CTAAA
GATGTGTTTTTGAAATGGGATG
>Cfu
ATTCATTAACATCACCATTGAAAGGGGTCACCACATTATCCCCTCTTCTCTGCTGCATTT
TCTCTAGCAGTTTATCGAGATCATCAC--CTGTTCAAAAAATGATGATAAAAGACAGGAA
GAAT-ATAAGGAATAAGATATTCATCAACCACCCGGCCCCAGGGTGACT-----------
-----------------------GTGGTGATGCCATTTAGGCTTGGCATTCAGAATTGAT
T-GCCAGGTATTCAGTCTTTGTTTGACAAGGAGTTCACA--GTGACTGCCTCTATGGCTA
TTTCCTTGTG-GACC---------------------------------------------
------------------------------------------------------------
------------------------------------------------------------
------------------------------------------------------------
------------------------------------------------------------
------------------------------------------------------------
------------------------------------------------------------
------------------------------------------------------------
```

```
----------------------ATTAAC---AAAGTGCTAAGAAGTGGA-CA-TTATGAA
CACATGGGATCAGCAARGAAAGCACAAGTCTC---ATGGGCAGGCTATGACAGAACACTG
AGAAGTAATTCAGAAGTTTATTTGGAGGGCTGCATATTTCAGAATTCACTTAC--CTAAA
GATGTGTTTTTGAAATGGGATG
>Nty
ATTCATTAACATCACCATTGAAAGGGGTAACCACATTGTCCCCTCTTCTCTGCTGCATTT
TTTCTAGCAGTTTATCTAGATCATCAC--CTATGAAAGAAATGATGAC-AGAGA-AAGAG
GATT-ATAAGGAATGAAGTTTTCATAAACCAGAATGTCCCAGAGTAACC-----------
-----------------------AGGATCGTGCTAGTTAGACTTGGTACTTAGATCTGAT
T-CCCA-------GGCATTTGTTTGATAAGGAGTTCACA-GCTGGCTGCCTCC-TGGCTG
TTTCCCTGTGAATCC---------------------------------------------
------------------------------------------------------------
------------------------------------------------------------
------------------------------------------------------------
------------------------------------------------------------
------------------------------------------------------------
------------------------------------------------------------
------------------------------------------------------------
----------------------ATTAAC---AAAATACTAAGAAGTGAG-CATTATAAAT
CCCAGGGGTTAAGTGAAGGAAGCACAAACCTC---ATG----------GATAAAACCCTG
AGAAGTCATATAGAAGTTTAGTGGGAGAGCTGCTGGCTTTTGTATCCACTTAC--CTAAA
GATGTGTTTTTGAAGTGGGATG
>Dgo
ATTCATTAACGTCGCCATTGAAAGGGGTAACCACATTGTCCCCTCTTTTCTGCTGCATTT
TCTCTAATAATTTATCTAGGTCATCAC--CTATGAAAGAAACAATGAC-AGAGAGAAGAG
GA---ATAAGGAATGAGGCTTTCATAAACCATTA-GTCCCAGGGTGATT-----------
-----------------------GGGGGCTTGCCAGTTAGACCTGGCACGCAGAG-TAAT
T-GCCAAGCATTCGGCATTTGTTTGACAAGGAGTTCACA-GCTGGCTGCCTCCATGGCTG
TTTACTTGTCAG-GC---------------------------------------------
------------------------------------------------------------
------------------------------------------------------------
------------------------------------------------------------
------------------------------------------------------------
------------------------------------------------------------
------------------------------------------------------------
------------------------------------------------------------
---------------------TATTAAA---AAATTACTGAGAAGTGTA-CAC-TCTGAA
CCTCCAGGATCAATGAGAAAAGCACAAGTCTCACAA--GATAGGCTATGACAGAAC-CTG
AGAAGCAATCCAGCAC---------AGAGTTCCTAACTTCTGTATCCACTTAC--CTAAA
GACGTGTTTTTGAAATGGGATG
>Scr
ATTCATTAATGTCGCCATTGAAAGGGGTAACCACATTGTCCCCTCTTCTCTGCTGCATTT
TCTCTAGTAATTTATCTAGATCATCAC--CTGTGAAAGAAACGATGAC-AGAGAGAAGAG
GAATAATAAGGAATGAGACTTTCATAAACCAGAA-GTCCCAGAGTGATT-----------
-----------------------GGGGGCTTTCCAGTTAGACCTGGCACTCAGAG-TGAT
T-GCCAAGCATTCAGCATTTGTTTGACAAGGAGTTCACA-GTTGGCTGCCTCCACAGCTG
TTTACTTGTCAG-GC---------------------------------------------
------------------------------------------------------------
------------------------------------------------------------
------------------------------------------------------------
------------------------------------------------------------
------------------------------------------------------------
------------------------------------------------------------
```

```
------------------------------------------------------------
---------------------TATTAAA---AAATTACTGAGAAGTGTA-CAC-TCTGAA
CCTCCAGGATCAATGAGAAAAGCACAAGTCTC-CCA--GATAGGCTATGACAGAAC-CTG
AGAAGCAATCCAGCAC---------AGAGCTCCTAACTTCTGTATCTACTTAC--CTAAA
GACGTGTTTTTGAAATGGGATG
>Bil
ACTCATTAACATCACCATTGAAAGGGGTAACCACATTGTCTCCTCTTCTCTGCTGCATTT
TCTCTAGCAGTTTATCTAGATCATCAC--CTGTGAAAGAAGTGATGAC-AGAGAGAAGAG
GATT-ACAAGGAATGAAGCTTTCTTAAACCAGAAGGTCTCAGGGTA---TGGGAGGT---
GAGAAGTGTGGGTGGAGTGGGGAAGGGTCATGCCAGTTAGATGTGATACTACGAATTGAT
T-GCCAAGCATTTAGCTTTTGTTTGATAAGGAGTTCACA-GTTGGTTGCCTCTTTGGTTG
GTTCCCTATGAGTCC---------------------------------------------
------------------------------------------------------------
------------------------------------------------------------
------------------------------------------------------------
------------------------------------------------------------
------------------------------------------------------------
------------------------------------------------------------
------------------------------------------------------------
----------------------ATTAAC---AAAATACTAAGAAGTGGA-CTC-TCTGAA
CCCCTAGGAGCAGTGAGAAAAGCACAAGTCTC---AAGGATAGGCTATGATAAAACCCTG
AGAAATAATAAAGCAATTTACTGGGAGAATTGCTGGGTTTTGTATGCACTTAC--CTAAA
GATGTGTTTTTGAAGTGAGATG
>Pgu
ACTCATTAACGTCACCATTGAAAGGGGTAACCACATTGTCTCCTCTTCTCTGCTGCATTT
TCTCTAGCAGTTTATCTAGATCGTCAC--CTGTGAAAGAAGTGATGAC-AGAGAGAAGAG
GATT-ATAAGGAATAAGGCTTTAAAAGACCAGAAGGTCCCAGGGTG---TGGGAAGTGAG
GAGAAATGTGGGTGGAGTGGGGAAGGGTCATGACAGTTAGA-CTGATACTAAGAATTGAT
T-GC-AAGCATTTAGCTTTTGTTTGACAAGGAGTTCACA-GCTGGCTGCCTCCATGGTTG
GTTCCCCGTGAGACC---------------------------------------------
------------------------------------------------------------
------------------------------------------------------------
------------------------------------------------------------
------------------------------------------------------------
------------------------------------------------------------
------------------------------------------------------------
------------------------------------------------------------
----------------------ATTAAC---AAAATGCTAAGAAGTGGA---C-GCTGAA
CCCCTAGGAGCAGTGAGGAAAGCGCAAGTCTC---ATGGAGAGGCTATGATAAAACCCTG
AGAAGTAATAAAGCAATTTATTGGGAGAGTTGCTAGTTTTTGTATCCACTTAC--CTAAA
GATGTGTTTTTGAAATGAGATG
>Iso
ACTCATTAAYGTCACCATTGAAAGGGGTAACCACATTGTCTCCTCTTCTCTGCTGCATTT
TCTCTAGCAGTTTATCTAGATCGTCAC--CTGTGAAAGAAGTGATGAC-AGAGAGAAGAG
GATT-ATAAGGAATGAGGCTTTAAAAGACCAGAAGGTCCCAGGGTG---TGGGAAGTGAG
GAGAAATGTGGGTGGAGTGGGGAGGGGTCATGCCAGTTAGA-CTGATACTAAGAATTGAT
T-GC-AAGCATTTAGCTTTTGTTTGACAAGGAGTTCACA-GCTGGCTGCCTCCATGGTYG
GTTCCCTGTGAGACC---------------------------------------------
------------------------------------------------------------
------------------------------------------------------------
------------------------------------------------------------
------------------------------------------------------------
------------------------------------------------------------
```

```
------------------------------------------------------------
------------------------------------------------------------
----------------------AATAAC---AAAATGCTAAGAAATGGA---A-GCTGAR
CCCCTAGGAGCAGTGAGGAAAGCACAAGTCTC---ATGGATAGGCTATGATAAAACCCTG
CGAAGTAATAAAGCAATTTATTGGGAGAGTTGCTGGTTTTTGTATCCACTTAC--CTAAA
GATGTGTTTTTGAAATGAGATG
>Tro
ATNCATTANTATCACCATTGAAGGGAGTAACCACATTNNCCCNTNTTNTNTGNTGCATTT
TCTCTAGCAATTTGTCCAGATCATCAC--NTGAGAAAGAAATGATATC-AGTGAGAAAAG
GNTT-ATAAGGAATGAGGCGTTCATCAACGGGAAGGTTTCAGGGTGACC-----------
-----------------------AGGGTCATGCCAGTTAGACTTGGCTCTCAGAACGCAT
CAACCGGGCATCTTGTCTTTATTTAACAAGGAGCTCACA-GCTGGCTGCCTCCATAGTTG
TTTCCCTGTGAGACC---ATTAAC-AAAATACTAAGAAACC---------CCGA-GGTCT
TCCCCATCCAGACTGA--TTTAAAATT--TTTAAAATTTCTTTTTAAAAATGTAATAATA
ATAATTATTTTTAACATTATTTTA-TATTATTATTTTATGTTATTAGGT-AAAGAGGCCA
CTTTTTGGCCTTATTTCTCATTAAGCCTAAAGTACTGAATGGGCTTTTTGTCTCAGTCAG
ACTGAAACCTGTTAAAAACCTT----------CGCCAAGATCCCCCACTACATCCTGGGC
CATCTCCAGTCATTCTGATGTATATCTTGCCAATGGACCCAGATGGCTCTGGGGGAGAAA
GTGAGACTGGTGGCCTCACACAGATTTCATTCACTTCAATCCAATTCACTTGCACGTCAT
G-----------------------------------GAGAAGGAC---------------
--ACATG-ACAA---------TATTAAC---AAAATATTAATAAACAGA-CTC-TCTGAA
CCCTTGGGATCAGTGATGAAGGCACAAGTCTC---ATGGAGAAGCTATGACAGAACCCTG
AACAGTAACACTAAAGCTTATTGGCAGAACTGCCGTTTTTTGTGTCCACTTAC--CTAAA
GATGTGTTTTTGAAATGGGGTG
>Ppe
ATTCATTAACATCGCCATTGAAGGGAGTAACCACATTGTCCCCTCTTCTCTGCTGCATTT
TCTCTAGCAATTTATCTAGATCATCAC--CTGTGAATGAAATGATAAC-AGAGAGAAAAG
GATT-ATAAGGAATGAGGCTTTCATCAGCCAGAAGGTTCCAGGC-GACC-----------
-----------------------ACGGTCATGCCAGTTAGACTTGGCACTTAGAACTGCT
T-GCCAGGCATTCACTCTCCATTTGACAAGGCATTCACAAGCCGGCTGCCTCCATGGATG
TTTCCCTTTGAGACC---ATTAACAAAAATACTAAGAAACC---------CTAA-GGTCT
TCCCCTCCCAAACTGA--TTTAAAATTTTTTTTAAACTTCTT------------------
---------------TTTATTTTATTATTATTATTA--TTTGACTAGGT-AAAGAGGCCA
-TTTTTTGCCTCATTTCTTATTAAGCCTAAATCACTGAATGGAAG--TTGCCTCAGTCAA
ACTGAGACCTGTTAAAGACCTTAGCTTGAAAAGGCCAAGACCCTCCACTGCATCCTTGGC
CATCTCCAGTCATTC-----TATATCTTGCCACTGGACCCAGATGACTCTGGAGGAGAAA
GTGAGGCTGGTGACCTTGCACAGCCCTGCCCCACATAAATCCAATTCACCTGCAAGTCAT
GGCATCACCTCCCTGATTTTTTGGTCCTCTTCAAGAAGGAAGGAA---------------
AAACAACAACAA---------TATTAAC---AAAATACTAAGAAATGGA-TAC-TCTGAA
CCCTTGGAACCAGTGATAAAAGCATAAGTCTC---ATGGAAGGGCTACGACAGAA-CCTG
AAAAGTAATACAGAAGTTTACTGGGAGAGCTGCTGGCTTTTGTATCCACTTAC--CTAAA
GATGTGTTTTTGAAATGGGNNN
>Tvu
NTTCATTAACATCGCCATTGAAAGGGGTAACCACATTGTCCCCTCTTCTCTGCTGCATTT
TCTCTAGCAATTTATCTAGATCATCAC--CTGTGAAAGAAATGACGAC-ACAGAGAAAAG
GATT-ATAAGGAATGAGGCTTTCATAAACCAGAAGGTTCCAGGGCAACC-----------
-----------------------A-GGTCATACCAGTTAGACTTGGCACTTAGAACTGAT
T-GCCAGGCATTCAGTC----TTTGACAAGGAGTTCAC-----GGC--------TGGCTG
TTTCCTTGTGAGACC---ATTAAC-AACATACTACGAAACC---------CTGA-GGTCT
TCTCCTCCCAGATTGATTTTTAAAATTTTTT---TATTTCTTTCT---------------
-------------ATGTTA--TTATTATTATTATTGTTTTTGACTAGGT-AAAGAGGCCA
-TTCTCTGCCTCATTTTTTATTAAGCCTTAAACACTGAATGGGCA--TTGCCTCANTCAA
ACGGAGACCTGTTAAAGACCTTAGCTTGAAAAGGCCAAGATCCCCCACTTCATTCTGGGC
```

CATCTCCATTCATTATGATCTATATCTGGCCACTGGAC----------TTGGAGGAGAAA
GTAAGGCTGGTGACTCTACACAGCCC-TCCCCACTTAAATCCAATTCATTTGCAAGTCAT
GTTATCATCTCCCTGATGCTATGGTCCTCTTCCAGAATGAAGGAC---------------
---AAACAACAA---------TATTAA------TATACTAAAAAATGGA-CAC-TCTGAA
CCCTTGGGATCAGTGATGAAAGCACAAATCTC---ATGGAGAAGCTATGACAGAACTCTG
GAAAGTTATACAGCAGTTTACTGGGAGAGCTGCTGGCTTTTGTATCCACTTAC--CTAAA
GATGTGTTTTTGAAATGGGATG
>Mro
N-TCATTAACATCGCCGTTGAAAGGGGTAACCACATTGTCCCCTCTTCTCTGCTGCATTT
TCTCTAGCAATTCATCTAGATCATCAC--CTGCAAAAGAAATGATAAC-AGAGAGAACAG
GATT-GTAAGGAATGAGGTTTTCATAAACCAGAAGGTTCCAGGGTGACC-----------
-----------------------AGGGTCATTCCACTTAGACTTGGCATTTAGAACTGAT
T-GCCAGGCATTAAATC----TTTGACAAGGAGTTCACA-GCTGGCTGCCTCCATGTTTT
TTTCCCTGTGAGACC---ATTAAC-AAAATACTAGGAAACC---------CTGA-GGTCT
TCCCCCCCCAGACTGATTTTTAAAATTTCT----TATTTCTTTTT---------------
------------------ATTTAATTATTATTATTATCATTTACTAGGT-AAAGAGGCCA
-TTCTCTGCCTCAATTTTTAATAAGCCTTAATCATAGAATGGGTG--TTGCCTCAGCCAA
ACTGAGACCTGCTAAACATTTTAGCTTGAAAAGGCCAAGATCCCCCACTGCATCCTGGGC
CATCCCCAGTAATTCTGATCTATATCTTGCTACTGGACCCAGATGGCTTTGGAGGTGAAA
GTGAGGCTCATGACTTTGTACAACCCTCTCTCACTTAAATCCAATTCACTTGCAAGTCAT
GGTATCACCTCCCTGATGTCATGGTCCTCTTTGAGATCAAAAGACAAAGAATAACAACAG
CACTATGAGCAAAA---------TACTA---AGAATACTAAATACTAAAATAC-TCTGAA
CCCCTGGGATCGGTGATGAAAGCACAATTCTC---ACAGATAGGCCACAATAGAACCCTA
AATGGTAATACAGCAGTTTGCTGGGACAGCTGCTGGCTTTTGCATCCACTTAC--CTAAA
GAGGTGTTTTTGAAATGGGATG
>Vur
-TCATTAAGCATCGCCATTGAAAGGGGTAACCACATTGTCCCCTCTTCTCTGCTGCATTT
TCTCTAGTAATTTATCTAGATCATCAC--CTGTGAAAGAAATGATGAC-AGAGAGAAGAG
GATT-ATAAGGAATGAGGCTTTCATAAATCATAAGGTTCCAGGGTGACC-----------
-----------------------AGGGTCATGCCAGTTAGACTTGGCACTTAGAAGTGAC
T-GCCAGGCACTCAGCCTTTGTTTGAC--GGAGTTCACA--GTGATTGCCTCC-TGGGTG
TTTCCTTATGAGACC---ATT-AC-AAAATAGTAAGAAACAAAATAGTAACTAA-GGTCT
TCCCCTCCCGGATTGATTTTCAAAA-----------------------------------
--------------TATTTTTTNAATATTATTATTTN--TTCACTAGGT-AAAGAGACCA
-TTCTCTGCCTCATTTCTTATTAAGCCTTAATCACTGAATGGGCA--CTGCCTCAGTCAA
ACTCAGACTTGTTAAAGACCTTAGCTTGAAAAGGCCAAGGTCCCCCACTGCATCCTGGGT
CATCTCCAGTG-TCCTGATCTGCATCTGGCCACTGGACCCAGATGGCTCGGGAGGAAAGA
GTGAGGCTGGTGACTTTGCACAGCCCTCCCTCATTTAAATCCAATTCACCTACATGTCAT
GGCATCACCTCCCTGATGGGACCATGGGGTCCCTTTTGTGAACAACGG------------
----ATAAACAA------------CAAC---AGTA-ACAACAATATT---AAC-TCTGAA
CCCCTAGGATCAGTGGGGAAAGCACAAGTCTC---ATGGCTAGCCTATGATAGAACCCTG
AAAAGCAATACAGCAGTTTACTGGGAGAGCTGCTGGCTTTNGTATCCACTTAC--CTAAA
GAGGTGTTTTTGAAATGGGATG

MARKER 95

```
>RTESINE2
------------------------------------------------------------
--------------------------------CGGCTGAAGCAGGCGCTGTGGAGCGCTT
AGAGCTTGGTCAGACATCGAAGATGCCAAGGTCATCCACTGCATCCCGGGCCATCACCAG
TCATCCTGACTTTTGTCTTGCCACTGGACTTCGATGACTCT-GGAAGAGAGAGTG-AGGC
TGATGACTTTGTGCAACTCTGCCTCACTTAAATCCAATTCATGCGCAAGTCAAGACATCA
CCCCATGATGTCATTGGTCCTCTTCG----AAAACGAA--GGACGAACAACAACAACAA-
------------------------------------------------------------
------------------------------------------------------------
------------------------------------------------------------
------------------------------------------------------------
------------------------------------------------------------
------------------------------------------------------------
------------------------------------------------------------
------------------------------------------------------------
------------------------------------------------------------
------------------------------------------------------------
------------------------------------------------------------
------------------------------------------------------------
------------------------------------------------------------
------------------------------------------------------------
------------------------------------------------------------
------------------------------------------------------------
------------------------------------------------------------
------------------------------------------------------------
------------------------------------------------------------
------------------------------------------------------------
------------------------------------------------------------
------------------------------------------------------------
------------------------------------------------------------
--------------------------------
>SINE1a_Mdo
------------------------------------------------------------
------------------------------------------------------------
------------------------------------------------------------
------------------------------------------------------------
------------------------------------------------------------
------------------------------------------------------------
------------------------------------------------------------
------------------------------------------------------------
------------------------------------------------------------
------------------------------------------------------------
------------------------TTTTTTTTTTTTTTTTAAACCCTTAC---TTTCTGT
CTTAGTAACAA-------------------CTCTAAGACAGAAG-GGCAAGGGCTAGGCA
ATTGGGGTTAAGTGACTTGCCCAGGGTCACACAGCTAGGAAGTGTCTGAGGCCAGATTTG
AACCCAGGTCCTCCTGACTCCAGGCCTGGTG-CTCTATCCACTGTGCCACCTAG-----C
TGCCCC------------------------------------------------------
------------------------------------------------------------
------------------------------------------------------------
------------------------------------------------------------
------------------------------------------------------------
```


>Plat_RTE1

---AACACGACAACACAACA------AGGGACA-GCCTTTTTGTGTGCACAATG---TGG
CCGGGACTGTGGGTCCCGCATTGGCCTTTTCAGCCACA-CACGCACTCATAGGTGAACTT
CACCGTCAGTGGCGTCT-TCTTCGAA----TACGAAGGACAACTATATATATATA-----

>SINE1a_Mdo


```
------------------------------------------------------------
------------------------------------------------------------
------------------------------------------------------------
------------------------------------------------------------
------------------------------------------------------------
------------------------------------------------------------
------------------------------------------------------------
------------------------------------------------------------
------------------------------------------------------------
------------------------------------------------------------
------------------------------------------------------------
------------------------------------------------------------
--------------------------------------------------TTTTTTTTTT
TTTTTTAAACCCTTACTTTCTGTCTTAGTAAC-------------AACTCTAAGACAGAA
GGGC---AAGGGCTAGGCAATTGGGGTTAAGTGACTTGCCCAGGGTCACACAGCTAGGAA
GTGTCTGAGGCCAGATTTGAACCCAGGTCCTCCTGACTCCAGGCCTGGTGCTCTATCCAC
TGTGCCACCTAGCT--GCCCC---------------------------------------
------------------------------------------------------------
------------------------------------------------------------
--------------------------------
>Mdo
TATTGAGGCCTGCTGTCACTAATGGAATTTCGCAG--GTAAGCAAG----TAGGCTACTT
AG----AGACTGACTATAAAC---------------------------------------
------------------------------------------------------------
------------------------------------------------------------
------------------------------------------------------------
------------------------------------------------------------
--TATAA---GGGTTTTAAAACAACTATAGAATATAGCCCTT--GTTGAAATAACCCAGT
---------------------TTCATTTTTCTTATCCCAGGACCTACCATTGAGGTTAGA
AAAGGCTTATCTAGAAA-CAGACCTAACAGTTGTTCTAGCACTCCCATAAGGAGCCCTTG
AATGCTTCATAAATGTCCCAGTGATGACTTCCTGGTTTTCCTCTCTTCATTT----GCC-
--TGTTCTTGGTTTGGTTTAG------------TTTTATAAAACTCTTAC---CTTCTGT
CCTAGAATTGATACTTAATAATAATAGATTCTAAGACAGAAGAGTGGCAAGGGCTAGGTA
AGCAGGCTTAAGAGACTTGCCCAGAGTCATACAACTAGGAAGTATCTGAGGCAAGATTTG
AACCTAGGTCCTTTTGACTCCAGACCTGGGCCCTCTATCCACTGTGCTACCTAACTGCCC
TGCCCC---TCTGCTCAGTTTTGATTGAG---GAAGACTATTAGA--ATA--CTTGACTT
-----CGAGTCAAGATTGTTC-TGGCAGGGTCC-ACGTTTTTGTGTGTTCACAG--ATGG
AAAAGACTCCTGAGCCTCCATTGGTCTGTTCAGCTGCATCACACAGGCAAAACACAGTCA
T------GGTGTATGTAATCTTTAAGCTTAGTGATAGGAAAGTGATTCATTTGTT---TG
GGATTTGCCTTCTGCAAACTCCATTCCCAATAATCATGGTGTAACCAAAACAACAAAAAA
A-----------------------------------------------------------
------------------------------------------------------------
---------GGCATGAAGGTGTCCATAGACATGTATATACGTTTACTAAGGTT-GAAGTG
TCAGGCCA-AGCAATATGGGAAATG------------CTTTAATAA--------------
------------------------------------------------------------
------------------------------------------------------------
------------------------------------------------------------
--------------------------------------TTTAAAACAATTATGACACAAC
AGTAATACTTTTTT-AGGTATACTTCTGTGTTACTATTTGCAATTCAT--TTATTTTTCC
TTCCCAAGAACTTTA-TTTTAA-CTACAATTGTCAACTATA--TTTTTCAG--CTTCCTA
GTTTAAATTCCAGTGCTTCCTCTTCTGGAAGT
>Mnu
TATTGAGGCCTGCTGTCACTAATGGGATTTCACAG--GTAAGCAAGTCAGTAGGCTACTT
```

```
AG----AGATTGATTATAAAC---------------------------------------
------------------------------------------------------------
------------------------------------------------------------
------------------------------------------------------------
------------------------------------------------------------
--TATAT---ATGTTTTAAAACAACTATAGAATATAGCCTTT--GTTAAAATAACCCAGT
---------------------------------ATCCCAAGACCTACCATTGAAGTTAGA
AAAG-CTCATCTAGAAA-CAGACCTAACAGTTGTTCTAGCACTCCCACAAGGAGCCAACG
AATGCTTTATAAATGTCCCAGTG-------------TTTCCTCTCTTCATTT----GTC-
--TGTTCTTGGTTTGGT-----------------CTTATAAAACTCTTACTACCTTCTGT
CCTAGAATTGATATTTAATACTAATAGATTCTAAGGCAAAAGAGTGGCAAGAGTTAGGCA
AGCAGGCTTAAGAGACTTGCCCAGACTTATACAGCTAGGAAGCAGCTGAGGCAAGATTTG
AACCCAGGTCCTTTTGAATCCAGGCCTGGGCCCTCTATCCACTGTGCTACCTAGCTGCCC
--TCTC---TGTGATCAGTTTTGATTCAG---GAAGTGTATTAGA--ATA-CTTAGAGT-
----------CAAGATTGCTC-TGGCAGGGTCC-AAGCCTTTGTGTGTTCACAG-GATGG
AAAAGTCTCCTGAGCCTCTGTTGGTCTGTTCAGCTACATCTCACAGACAAAACA--GTCA
T------GGTGTGTGTAATCTTTAAGCT-AGTTATAGAAAGG-GA-TCATT-GTT---TA
GGATTGG-CTTTTGCAAACTCCATTCCCAACAATCNCGGNGTAACCAAAACAAAAAAAA-
------TCCTAAAGTTGATTAGTTTTATCCCCAAGATCCTTTTAGGATAAAGAATACAGT
CAGTAGTACAAACTCAGGAACTGAAAATGTAGGAGAAGATCAAATAGGTGGAAAGAAGAA
AG----TAAGGCATAAAAGTGTTCATAAAAATGTATATATGTTTACTAAGGTT-GAAGTG
GCAGGCCA-AGCAATATGGAAAATGCAAGACATT---TTAGAATTAA----------TTT
TTTTTTAAACCCTTACCTTCCGTCTTAGAGGCAATATTGTGTATAGGTTCCAAGGCAGAA
GAGTGATAAGGGTAGGGCAATGGGGGTCAAGTGACTTGCCCATCGTCACACGGCTGGGAA
GTGTCTAAGGCCAGGTTTTAACCTAGGACCTCCTGTCTGTAGGCCTGGCTCTCAATCCAC
TGAGCTACCCAGCTTTCCCCC---TTTAGAATTAA---TTTAAAACAATTATGGCACAAC
AGCAACACTTTTTA-AGGTATCCTTCTGTGTTTCTATGTGCAATTCAC--CTATTTTTCC
TTCCCAAGAACTTTA-TTTTAA-CTACAATTGACAACTATATATTT--CAG--CTTCCCA
GTATAAATCCCAGTGCTTCCTCTTCTGGAAGT
>Cfu
TATTGAGACCTGCTGTCACTAATGGGATGTCACAG--GTAAACAAGTCAATAGGTTACTT
ATATACAGATTAACTATGAAG---------------------------------------
------------------------------------------------------------
------------------------------------------------------------
------------------------------------------------------------
------------------------------------------------------------
--TATGT-----TTTCTAAAACAACAGTAGAATAGCTCATTGAGGTTAAAATGCTCATGT
-----CTG-AGATAATACAACTTGGTCCTTCTTGTCCCAGGACTTACCATTGAGGTTAT-
-------AAGAAACAAAACAGATCCCAGAGTTGTTCCAGCTCTCCCACTAGGAGCCAATG
AATGCTATAGAAATACCGCCAGAG--ATTTCCTGGTTT--CTTCCTTCATTTGTCTGTC-
------------------------------------------------------------
------------------------------------------------------------
------------------------------------------------------------
------------------------------------------------------------
---------TGTGCTCAGGTTTGATTCAG---GA---GTATTAAA--ACACCTTTGACTT
-----AGAGCCAGGATTGCTATTGTCAGGGTCC-AAGTCTTTTTGTGTTCACAG-TGTAG
AAAAGACTGCTGAGCC-GCATCGATCTGTTCAGCCCTACCAAACAAGCAAAGCACAATCA
CCATCTGAGTCATCATTCCCTTCAAGCTTAGTAATAG-AGACTGATTCATTTATT---TG
AGATTGTCCTTCCACAAACTCCTTTCCCAGCAATCCTGGTGTCACCAAAACACAAATAAA
AAAA--TCCTGAAGTTGATTAATTTTATCC-CAAGATCCTTTCAGGATGAAGAATGAAGT
CAGTAATAAAAGTATAGGAATTGAAAATGCAGGGGAAG---AGAAGGGAGGCAGAAAGAA
AG----TAAGGCTTAAAATA-TCCACAGGAATTTATCTGTTTTTACTAAGGTT-GAAGTG
TTAGGCCA-AGTGATATGAAAAGTGGAGGGCATT---CTAAAATTAG-------------
```


--------------------------------------TCCTAAAACAATGA-GTACCAC
AGCAACACTTTTTT-AGG---TCTCTTATATTGCTGTTTGTAATTCAT--CT-GTTTTCT
TCTCTAAGTACTTCAGTTTTAA-CTACAGTTGATGACTATATATGYTTCAG--CTTCCCA
GTATAAATCCTAGTGCTTCCTCTTCTGGAAGT
>Dgl
TATTGAGACCTGCTGTCACTAATGGGATTTCACAG--GTAAGCAAGTCAGGGAGCTACTT
AT----AGATTAACTACAAAC---TATGT---CAGCTGAAGCAGGTGCTGTGGCATGCTT
AGAGCTTGGGCAGGCATCAAAGACACCAAGGTCGTCCACTGCATCCTGGACCATCATCTG
TCGTCTTGACTTTTGTTCTACCACTAGACTTGGATGACTTTAAGAAGAGAGAGTG-AGTC
TGATAACTTTGTG----------------------AATCCACAAACAAGTCAGGACATCA
CTAT-CCATGTCATTGGTCCTCTTTTT-AAAAAACCAAACGGATGAACAA-----GTTA-
--TATGT-----ATTCTCAAGCAATGGTGGAATATA-CTGCTTGTTGAGATTAAGCTCCT
CATGTCTG-AGGTAACCCAACTTGATCCTTCTTGTGCCCAGTCCTACCACTGAGGTTATG
AAAGACTCAAAAAACA--GACACA--AAAGTTGATCTACCTCATCCACTAGGAACTGGTG
AATACTTTAGAAATATCCCAATG---ACTTCCTGGTTTCCTTTCCTTCGTTA-----TC-

---------TATGCTCAGTTTTGATTCAG---GG-GAATACTAAA--ACATCTTTGACTT
-----ANAGTCAGGATCACCA-TGTCAGGGTCC-ACATCT--GTGTGTTCACAG-TGTGG
AAAAGACT----AACCTCCATTAGTCTGTCCAGCCATANTGCACAAGCAAA-CATGATC-
-----TGGGGCACAGTTATCTTCAAGCCGGNTGGTATAGA-CTGATTCNTTTGTT---TG
GGATTGTCCCCCTGCAAACTTAATTCCCNAAAATCCTAGNGTAGTCAAAATTAAAAAAAA
AAAACACCCTGAAGTTGATTAATTTCATCC-CAAGATC-TTTCAGGATGAAGAATATTGC
CAGTAGTGAAAGCATAGGAATTGAAAATGCAGGGGGAG--GAGTAGGTAGGTAGAAAGAA
AG----TCAGGTTTGAAGGTGTCTACAGAAATGTTTCTATTTTTACTAAGGTT-GAAGTG
TTCAGGCTGGACGATAT-AAAAATGCAGGGCATT---TTAAAATTAG-------------

--------------------------------------TCTATAACAAGTATGGTACACA
AGCAAC-TTTTTTT-AGGTATTCTTCTGTATTAGTATGTATAGTTTAT--CTATTTTTCT
TTCTGCAGTACTTT-GTTTTAA-GTACAGTTGATGACTATATATGTTTCAG--CTTCCCA
GTATAAATCCCAGTGCTTCGTCTTCTGGAAGT
>Nty
TACTGAGACCTGCTGTCACTAATGGGATTTCACAG--GTAAGCAAGTCAGGAAGCTACTT
AT----AGATTAACTATAAAC---TATGT---TATTTGAAGCACGGGCTATGGAGTTCTG
AGAGCTTAGTTAGGCATCAAAGACACTGAGATCATCCACTGCATTCTGAGCCATTGCAAG
TTGCCTTGATGTTTGTCCTGCCACTAGACTTTGAGGACTTT-------------------
----------GTGCAGCTCTACCTCATTTAAATACAATTCACACAGCAGA-TGAACAG--
---CAA-----GTTA-
--TATGT-----GTTCTAAAGCAACTGTAGAATATAGCTGGTTGTTGAGGTTAAACTCCT
TGCATCTG-AGATAACCTAACTTGATCCTCCTCATCCCCAGACCTACCATTGAGCTTAGG
GGACACTCTAGAAA----CAGACCCAAAAGTTGTTCCAACTCATCTACTAGGAGCCAGTG
AATGCCTTAGACATTTCCTACTG---ACTTCCTGGTATCCCTTCCTGTATTT----GTC-

---------TGTGCTCAGTTTTGATTCAG---GGGATGTATTAATAAACACTTTTGACTT
-----AGAGTCAAGATTGTTA-TGCCAGGGTCC-AAATCTTTGTATGTTCACAG-TATAG
AAAAGACTGTTTAACCTCCATTAGTCTGTTCAACCACACTGCACAAGCAAAACAAAATTG
CCATCTGTGGCATCATTATCTTCATGCCTGGTGATAGAGC-CTGATTCATTTGTT---TG
GGATTGTCCTACTATAAACTCTATTCCCAGCAGTCCTGGTGTAATCAAAGTAAAAAAAAA
A-----TCCTAAAGTTGATTAATTTTATCC-CAAGATCCTGTCAGCATGAAGAATATAGC
TAGTAATGAAAGCATAGGAATTGAAAATTCCAGGGTTCA-GAAGAGTAAGTAAGC-AAAA
AGAAAGTAAAGTATGAAGATGTCCACAGAAATGTTTCTCTTTTTACTAACGTT-GAAGTA
TTGGTCCA-GACAATATGAAAAATGCAGGGCACT---TTAAAATTAG-------------

--------------------------------------TCTAAAACAATTATGGTATACT
AGCAATAGTTTTT--AAGGTATCTTTTGTGTTACTTTGTGAAATTCAT--CTATTTTTCT
TTTCCAAGAACTTT-----------ACACTTGATGACTATATATGTTTCAG--CTGCCCA
GTATAAATCCCAGTGCTTCCTCTTCTGGGAGT
>Pta
TATTGAGACCTGCTGTCACTAATGGGATTTCACAG--GTAAGCAAGTCAGGAACCTACTT
ATTTATAGATTAATTGTAGAC---T--GT---CAGCTGAAGTACACACCATGGAGTCCTT
AAAGCTTGTTCAGGTATCAAAGACGCCAAGGTCATCCCCTGCATTCTGGACTGTTGCCA-
TTGTCTCAACTTTTGTCCTGTCACTGAACTTTGATGACTTA-GAAAGACAGAGTG-AGGC
TGATGACTTTGTATAGCTCTGCCTTACTTAAATCCAGTTCATGAGCAAGTCATGACATCA
CCAT--GATATGATTGGTTCTCT---AAAAAAATCCAAGACAAACAGTAA-----GTTA-
--T--CT-----GTTCTAAAGCAATTTTGGAATATAGGTGTTTGTTGAAGTTCAGCTCCT
CATGTCTGGGGACAACTTAACTTGATTCTTCTTGTCCCCAA-CCTACTATTGAGGTTGTG
AAAGATTTATCTGGAAA-CAGAACC-AAAGTTGTTCTGGCTCATCTGTTAGGAGCCAATG
AATGCTTTAGAAATTTTCCAGTG---ACTTCCTAGTTTCTCTTCTTTCATTT--------

---------------------CAAAATCT---TT-CAGT-TTAAA--ACACCTTTGACCT
-----AGGGTCAAGATTGCAA-CGTCAGGGTCC-AAGTCTTTGTGTATTT----------
------------AACTTCCATTAGTCTGTTCAGCCACACTGCACAAGGCAAACACAATC-
-----TGTGGCATCATTATTTTCAAGCTTGGTGATAGGGG-CTGATTCATTTGTT---TG
GTATTGTCATACTATAAACTCTATTCCCAATAATCCTGGTGTGATCAAAGTAAAAAAAAA
AA----TCCTGAAGTTGAGTAATTTTATTCT-AAGATCTTT-CAGAATGAAGAACACAGC
CNATAATAAAAGCATAGAAATTGAAATTGCAGAGGGAA--GAGTAGATAGGTAAAAAGAA
AG----TAAAGCATGAAGTTGTCCACAGAAATAGTTATATTTTTAATAAGGTT-GAAGTA
TTAGAACA-GACAGTATGAAAAGTGAAATGCACT---TTTAAATTAG-------------

--------------------------------------CCTAAAACAATTAGGTTACACT
AGCACCACTTTT---AGGTATTCTCTGTTATTCT-----GGAGTTCAT--TTATTTTCCT
TTCACAAATACTTT------AA-GTACAATTGATGATTATTTATGTTTCAG--CTTCCCA
GTATAAATCCCAGTGCTTCCTCTTCTGGAAGT
>Dgo
TATTGAGGCCTGCTGTCACTAATGGGATTTCACAG--GTAAGCAAGTCAGGAACCTACTT
ACTTATAGATTAACTGTAAAC---TGTGT---CAGCTAAAGTACACACCATGGAGTCCTT
AAAACTTGTTCAAGCACCAAAGACGCCAAGGTCATCTCCTGCATCCTGGACCATTGCCA-
TTGTCTCAACCTTTGTCCTGTCACTGAACTTTGATGACTTA-GAAAGACAGAGTA-AGGC
TAATGACTTTTTATAGCCCTGCCTTACTTAAATCCAATTCATGAGCAAGTCATAATATCA

CCAT--AATATCATTGGTTCTCT---AAAAAAATCCAAGACAAACAAGAA-----GTTA-
--TATGT-----T--CTAAAGCAATTTTGGAATATAGGTGTTTGTTGAGGTTAAGCTCCT
CATGTCTGGGGACAACCTAACTTTATTCTTCTTGTCCCCAA-CCTACTATTGAGGTTATG
AAAGATTTATCTGGAAA-CAGACCCAAAAGTTGTTCTGGCTCATCTGCTAGGAGCCAATG
AATGCTTTAGAAATTTTCCAGTG---ACTTCCTAGTTTCTCTTCCTTCATTT--------

---------------------TAAAATCT---TT-CAGTATTAAA--ACACCTTTGACCT
-----AGAGTCAAGATTGCAA-TGTCAGGGTCC-AAGTCTTTGTGTATTT----------
------------AACTTTAATTAGTCTGTTCAGCCACACTGCACAAGCCAAGCACAATC-
CCATCTGTGGCATCATCATTTTCAAGCTTGGTGATGGGGG-TTGATTCATTTGTT---TG
GTATTGTCATACTATAAACTCTATTCCCNGTAATCCTCATGTAATCAAAGTTAAAAAAAA
AAAA--TCCTGAAGTTGATTAATTTTATTC-TAAGATGCTTTCAGAATGAAGAACACAGC
CAATAATAAAAGCATAGAAATTGAAATTGCAGAGGGAA--GAGTAGGTAGGTAAAAAGAA
AG----TAAAGCATGAAGTTGTCCACAGAAATATTTTTATTTTTAATAAGGTT-CAAGTA
TTAGAACA-AACAGTATGAAAAGTGAAGTGCATT---TTTAAATTAG-------------

--------------------------------------CCTAAAACAATTAGGGTACACT
AGCACCACTTTT---AGGTATTCTCTGTTATTCT-----GGAGTTCAT--CTATTTTCCT
TTCACAAGTACTTT------AA-GTACAATTGATGATTATATATGTTTCAG--CTTCCCA
GTATAAATCCCAGTGCTTCCTCTTCTGGAAGT
>Tro
TATTGAGACCTGCTGTCACTAATGGGATTTCACAG--GTGAGTGAGTCAGGAAGCTACTT
AT----AGATTAACTATAAAC---TATGT---CAGTGAA-GCAGTTGCTTA---------
GAGCTTTGGTCAGACATCAAAGACACCACGGTCATCTACTGCATCCTGGGCTA------G
TCATCTTGACTTTTGTACTACCACTAGACCTTGATGACTGT-GAAAGAGAGAGTTAAGGT
TGATGACTTTGGGCAACTCTACCTCATTTGAATACAATTTACAATAAAGTCAGGGTATC-
-----TGATGTTGCTGGTCCTCTTTTTT----------------AAAAAA-----GTTA-
--TATGT-----GTTCTAAAGAAACTGTGGAATATAGCTGCTTATTGAAGTTAAGCTCGT
TGTGTCTG-AAATACCCTAACTTGATC---CTTGTCCCCAGACCTCCCTTTGAGGTTATC
AGAGACTCTTCTAGAAAACAGACCCAAAATTTGTTCCAGCTCATCTCCTAAGAACCAGTG
AACACTGTAGAAATGTCCCAGTG---ACCTCCTGGTTTCCTTTCCTTCATTA----GTC-

---------TGTGCTCAGTTTTGATACAG---ATGGAATATTAAA--CTACCTTTGACTT
T----AGAGTCAGGTTTGCCC-TGTTAGAGCCCCAAGTCTTTGTATATTCACTG-TGTGG
AAAACACTATTTAAATTCCATTGGTCTGTTTAGCCGCACTGCACAAGTAAACCACAGTCG
TTGTCTGGGG--TCATTATCTTCAGGCCTGGTGATAGAGA-CTGATTGATTTGTT---TG
AAATTGTCCTTCTGCAGATTCTATTCCCAGCAATCCTGGTGTAATCAAAGTTTTAAAGGG
G-----CCTTAAAATTGATTAA-TTTATCC-CAAAAGCCCTTCTGGATAAAGAATACAGC
CAGTAATGAAAGCATAGGAATTGAAAATGCAGGGGGGAA-GAGTAGGTAGCTACTAAGAA
AG----TAAGGTATGAAGATGTCCACAGAAATGTTGATGTTTTTACCA-GGTTTGAAGTG
TTAGGCCA-GACAATATGAAAAACC--GTGCATT---TTAAAATTAC-------------

--GGTGCACT

ACCCATACGTTTTGTAGGTATCCTTCTGTGTTGC-----------------CTATTTTCC
TTCCCAAGTACTTT------AA-GTACAGTTAATGACTATATATTTTTCAG--CTTCCCA
GTATAAATCCCAGTGCTTCCTCTTCTGGAAGT
>Mro
TATTGAGACCTGCTGTCACTAATGGGATTTCACAG--GTAAGCAAGTCAGGAAGCTACTT
AT----AGATTAACTATAAAC---TCTGT---CAGCTGAAGCAGGTGCTGTGGGGCACTT
AGAGCTTGGTCAGGCATCAAAGACATCAAGGTCATCCACTGCATCCTGGGCCATCGCCAG
TCCTCTTGACTTTTGTCCTGCCACTAGACTTTGATGACTTAAGAAAGACAGAGTG-AGGC
TGATGACTTTATGCAGCTCTGCCTCACTTAAATCCAATTCATGAGCAAGTCAAAATATCA
CCTG-GGATGCCACTTGTCCTCTTTAAAAAAAAAAAGAGATGAAGAACAA-----GTTA-
--TATGT-----GTTCTAAAGCAACTATGGAATATAGCTGCTTATTGAGGTTAAACTCCT
TTTGTCTG-AGATAACCAGACCTCCCGTT-------------------------------
--------------------------GATGTTGTTCCAGTTCATCTAGTGGGAGCCAATG
AAGGCTTTAGAAATTTCCCAGTG---ACTTCCTGGTTTCCCTTCCTTCATTT----GTC-

---------TGAT--CAGTGTTGATAGAG---GTAGAATATTAAA--ACACCTTTGATTT
-----CGAGTCAAGATTGCTA-TGTCAGAATCCACAGA----------------------
----GACTATTGAAACTCCATTAGTCCTTTCAGTGATACTGCACAAGCAAAACACGGTCA
CCATCTGGGGCATCATTATCTTCAAGCCTGCTGGTAGAGA-GTGATTCATTTGTT---TG
AAATTGTCCTTCTGAGGA--CTATTCCTAGAAATCCTGGTGTAAT----TTTTTAAAAAA
------TCCTAAAGTTGATTAATTTTATCC-CAAAATCCTTTCAGGGTGAAGAATACAGC
CAGTAATGAAGGGATAGGAATTGAAAATGCAGGGGGAA--GAGTAGATAGGTAGAAAGAA
AG----TAAGACATGAAGGTGTCTACAGAAATGTTTATATTTTTACTA-GGATTGAAGTG
TTAGGCCA-GACA---TG--AAATGCAATGCATT---TTTAAATTAG-------------

--------------------------------------TCTAAAACAATTATGGTACACT
AGCTACACTTTTTT-AGGTATCCTTCTGTGTTACTATGTTCAATTCACCTGTA--TTTCT
TTCCCAAGTATTTT-GTTTTAAATTATAGTTGATGACTATATATGTTTCAG--CTTCCCA
GTATAAATCCCAGTGCTTCCTCTTCTGGAAGT
>human
TATTGAGACCTGCCGTCACCAATGGGATGTCACAG--GTAAA----CTGGTGTGTGTGGG
--------------TATAACT---------------------------------------

-----------------------TGTTGAATTAAATAATGTATGAAATGCCAGTTTACAT
GACAGATTATTCTTCCCAGACCTATTTAGTATGTGAAGCCATTACTAGTTGTATTTTTTT
CTACCCA---AGCACAGCACTCTAATTATGT--TGGCCTTCTGCATTTTAG--CTTCCTA
GCATAAACCCTAGTG---CCTCTTCTGGAAAC

MARKER 96

>WALLSI3

---TTGTTGTTGTTGTTC
GTCCTTCGTTTTC---GAAGAGGACCAATGACATCAT-GGGGTG---ATGTCTTGACTT-
GCGCATGAATTGGATTT-AAGTGAGGCAGAGTTGCACAAA--GTCATCAGCCTCACTCTC
TCTTCCAGAGTC-ATCGAAGTCCAGTGG--CAAGACAAAAGTCAGGATGACTGGTGATGG
CCCGGGATGCA---

>WALLSI1A

-----------TGTTTGGGCCCTGAT--TGGCTCAGAGTGAATGTAAATAGCAATTGTTT
-CTGTTT---TGGCCAGAAACCCTGAGGGTCTTCCCCTCCCAGATTGATTTTTTTTTTTT
TTTTGACTAGGTAAAAGAGGCCATTCTTTGCCTCATTTCTTACCTAGCCTTAATCACTGA
ATGGGTGTTGCCTCAG--

>WSINE1

---GAGGGGCAGCTAGGTGGTG
CAGTGGATAGAGCACCAGCCCTGGAGTCAGGAGGACCTGAGTTCAAATCCAGCCTCAGAC

```
ACTTACTAG--------CTGTGTGACCCTGGGCAAGTCACTTAACCCCAATTGCCTC---
------------AAAAAAAAAAAAAA----------------------------------
------------------------------------------------------------
------------------------------------------------------------
------------------------------------------------------------
---
>Mdo
TGGTTCACAGACAGAGCTGGCCATTGTTACTGCAGACTTTGTCAAGGTAC----TCTCTC
T-TTCAGTTATTGCTGGTACAGTGACC----TGGGATTGAGATCCTTTGG----------
---------------TTCTCAAT---GGGGGTG---------------------------
------------------------------------------------------------
------------------------------------------------------------
------------------------------------------------------------
------------------------------------------------------------
------------------------------------------------------------
------------------------------------------------------------
--------------------ACCCATT---AGGGACTAACACAGAGATATCAGTGGCTCA
TTTTGGTTTTGCCAAGCATAA---------------------------------------
------------------------------------------------------------
------------------------------------------------------------
-----------------------------GAAATCTGGTTTAAGCCAA-GCTAA---AAC
TCATCTTAATCCTTTGTCTACTAGTCTCCACCGCAGCCTGGCAGCTTTAGTTGGTTTAAC
CACAAATGAATCAAATGGGA-----TTCTTCCAGAGAGGACCAAGAGTTGGCTTCTTGTT
T--AGTCTTTTCTCTCTC----------TACAGATATATGACCTCTCAGTTGATGCATTG
AGC
>Dvi
TGGCTCACAGACAGAGCTGGCCATTGTTACTGCAGACTTTGTCAAGGTACTGACTGTCTC
C-TTCAGTTATTGCTGTTATAGTGACCCTTCTGGGATTGAGATCCTTTGG----------
---------------TTCTCAAT---GGGGATG---------------------------
------------------------------------------------------------
------------------------------------------------------------
------------------------------------------------------------
------------------------------------------------------------
------------------------------------------------------------
------------------------------------------------------------
--------------------ACCCATT---AGGGACTAACACAGAGATATCAGTGGCTCA
TTTTGGTTTTACCAAGCATAA---------------------------------------
------------------------------------------------------------
------------------------------------------------------------
-----------------------------GAAATCTGGTTTAAACCAA-ACTAA---AAC
TCATCTTACTCCTTTGTCTACTAGTCACCACTGAAGCCTGACATGTTTAATTGGTTTAAC
CACTAATGAATCAAATGGGA-----TTCTTCCAGAGAGGACCAAGAGTTGGCTTCTTGTT
T--AGTCTTTTCTCTCTC----------TACAGATATATGACCTCTCAGTTGATGCATTG
AGC
>Mnu
TGGCTCACAGACAGAGCTGGCCATTGTTACTGCAGACTTTGTCAAGGTACTGACTGTCTC
C-TTCAGTTATTGCTGTTACAGTGACCCTTCTGGGATTGAGATCCTTTGG----------
---------------TTCTCAAT---GGGGATG---------------------------
------------------------------------------------------------
------------------------------------------------------------
------------------------------------------------------------
------------------------------------------------------------
------------------------------------------------------------
```

--------------------ACCCATT---AGGGACTAACACAGAGATATCAGTGGCTCA
TTTTGGTTTTACCAAGAATAA---------------------------------------

-----------------------------GAAATCTGGTTTAAACCAA-ACTAA---AAC
TCATCTTACTCCCTTGTCTACTAGTCTCCACCGAAGCCTGACATGTTTAATTGGTTTAAC
CACTAATGAATCAAATGGGA-----TTCTTCCAGAGAGGACCAAGAGTTGGCTTCTTGTT
T--AGTCTTTTCTCTCTC----------TACAGATATATGACCTCTCAGTTGATGCATTG
AGC
>Rra
----------------CTGGCCATTGTTACTGCAGACTTTGTCAAGGTA----CTGTCTC
C----TCCTGGTGTTGCCCCACTGACCCTATGCTGAT-GGGACTCCTTGCTTGTGG-CAT
A---CTTCAGAGGGTCACTCAGCAAAGGCCCCC---------------------------

-TTCAGG---

-----------------------------GA---TTG------TCCCA-GATCT------
--------CTGGCCC-TCTCCTTGTCTCCACCTTGGTCTGGCATCCTGGGCTGGCTCATT
CTCTAAAGGAGTCGGGGAAG-----TTCTTCAAGGGAGGATCAAGAGCTGGCCTCTGGTT
TTTAGTCCTCTCTCTCTATTTCTCTCCCCACAGATATACGACCTCTCTGTGGATGCTTTG
AGC
>Dgl
TGGCTCACAGACAGAGCTGGCCATCGTTACTGCAGACTTTGTCAAGGTA----CCGTGTC
C----TCTTGGGGTTGGCACAATGACCCTGTGATGGTTGGGAGCCTGTACTAGCCCACAG
TGTGCTTCAGAAAGCTTCTCAGT---GGGGGTG---------------------------
---ATCTATTGTC---GAAGAGGACCAATGACATCACAAAAGTG---ACATCTTGACTT-
GTGTGTGAATTGCTTTT-AAGTGAGACAGAGCTGCTCAAA--GTTGGCAGACCGACTCTG
TCCTCT--AGTC-ATTGAAGTCCAGTGG--CAAGACA-TAGG--TTAAGACAAAGAACCT
CTGTGGCTCATTGTTTAGGTTTTGA----TGGCTTAGAATGAATGTAAATTGCAATTGTT
TACTGTTCTGTTGTCTCAGATCTGTTAGGG------------------------------

----------------------------------AATAAC--------------GGCTCC
TTTTCCTTTTGCCAAGCATAA---------------------------------------

-----------------------------GAAA----------TCCCAAGTTAT---AAC
TCATTCTAATCCTTTGTCTTCTAGTCTCCATCCCAGCCTGGTAGCCTTGGTTGGTTTAGC
CACTAATGAATCAGCCGGAG-----TTCTACCAGACAGGACCAAGAGTTGGCTTCTTATT
G--AGTTTTTTCTCTCTCTG-----CCCCACAGATATACGACCTCTCTGTTGATGCCTTG
AGC
>Nty
TGGCTCACAGACAGAGCTGGCCATCGTAACTGCAGACTTTGTCAAGGTA----CTGTCTT
C-TTCTCTTCATGCGGGTACAAAGACCCTATGGTGA-CTGGAGCCTCTGCTAGCCAACAG
TATGCTTCTGAAAGCTTCTAAAT------CAGG-----TTACGC---ATTGTTGTTGTTC
ATCCTTCATTCTT---GAAGAGGGCTGACTACATCACAAAGATG---ATGGTTTGACCT-

GTGTGTGAATTGTTTTTTTTTTAAGTGAGTCAGTTGCGCAAAGTCAT----CAGCTTTAC
TCCTCC--AGTC-ATTGAAGGCCAGTAG--CAAGACATAAGT-CATGATGCCAAGCCTCT
ATGGCTTATT--GTTTAAGTTTTAA----TGGCTTAGAATGAATGTAAATAGTAATTGTT
TTCTGTTC---TGGTCAGAAACTTTGAGGGTCTTCCTCTCCCACATTGATATTTTT----
------TCATGGTAAATTTGGCCATCTTTTGTCTCANATCTTAGTTATTGAAA-------
---GGCCATCACCTCAT---ATCCATT---AGGGACTAACA----GATTTCAGTGGCTCA
TTTTGCTTTTGCCAAGCATTA---------------------------------------

-----------------------------GAAA----------TCCTA-ATTAA---AAC
TCATTTTAATCCTTTGTCTACTAGTTTCCACCTCAGCCTGA-------------TTTAGT
CACTAATGAATCAGATGAGG-----TTCTGCCAGAAAGGACCAAGAGTTGGCTTCTTGTT
T--ATTCTTTTTTCTCTCTA-----TCCTACAGATATACGACCTCTCTATTGACGCCTTG
AGC
>Pta
TGGCTCACAGACAGAGCTGGCCATCGTCACTGCAGACTTTGTCAAGGTA----CTGTCTT
C----TCTTAGTGTTGGTA-----------TGGTGG----GAGTCTCTGCTT--------
-------CAGAAGGCTTCTCT------GGGAGC---AACCCACT---GGTGATGGCGGTC
ATCCCTCGTTCTC---GAAGACGACCAGTGCCATTGCGAAGGTG----TGATTTGACTT-
GGATGTG-----------AAGTGAGACAG--CTGCTCAGA--GTCAT-------------
--------------------------------ACATA-TGTT--AAATTGGAAAGCCTCT
GTGACTTAAC-------TATTGAGG----CTTATGTGAATGTAGAT-AAGAGGGATCGTT
TTCTGTTC---TGCTCAAAAA---------TCTTCCCCTCCTAGATGGATGTTTTT----
------TGAAGATACATTAGGTCATCTTTTGTCTTAATTCTTACTTATTGAAA-------
---AGGCATTGTCTCAG---ATCCATT---AAGGACTAACA----GATTTCAGTGCCTCA
TTTTGCTTTTGCCA------A---------------------------------------

-----------------------------GAAA----------TCCAA-GTTAA---AAC
TCATTCTGACACTTTGCC----AGTCTCTACCTTAGCCTGGCAGCTTTAGTTT-AGCAGT
AATAGGGTAGGT----GAAC-----TTCTGCCCGAGAGTATCAAGGGTTGGCTTCTTATT
---AGTGTTTTCTTTCTCTA-----CCCTACAGATATACGACCTCTCTGTTGATGCCTTG
AGC
>Scr
TGGCTCACAGACAGAGCTGGCCATCGTCACTGCAGACTTTGTCAAGGTA----CTGTCTC
C----TCTTAGTGTTGGTA-----------TGGTGG----GAGTCTCTGCTT--------
-------CAGAAGGCTTCTCT------GGGAGC---AACCCACT---GGTGGTGATGGTC
ATCCCTCCTTCTC---GAAGAGCACCAGTGCCAGTGCAAAGGTG----TGATTTGACTT-
GGAGGTGAATTGTTTTTTAAGTGAGACAGAGCTGCTCAGA--GTCAT-------------
--------------------------------ACATA-CATT--AAATTAACAAGCCTCT
GTGACTTAC--------TATTG-------AGGCTTAGTATGAGTGTAGATAGGGATCGTT
TTCTGTTC---TGCTGAAAAACTCTGAGGGTCTTCCCCTCCTAGGTGGATGTTTTT----
-----GT-AAAGTTGATTAGGCCATCTTTTGTCTTAATTCTTATTTATTGAAA-------
---GGAAGTTGTCTCAG---ATCCATT---AGGAACTAACA----GATTTCAGTGGCTTA
TTTTGCTTTTGCCA------G---------------------------------------

-----------------------------GAAA----------TCCAA-GTTAA---AAC
TCATTCTAACACTTTGTCTGCCAATCTCTACCTTAGCCTGGCAGCCTTGGTTT-AGCAGT
AATGGGTTATGT----GAG------CTCTGCCTGAAAGTGTCAAGAGTTGGCTATTTATT
---AGTCTTTTCTTTCTTTA-----CCCTGCAGATATACGACCTCTCTGTTGATGCCTTG
AGC
>Mfa

TGGCTCACAGACAGAGCTGGCCATAGTCACTGCAGACTTTGTCAAGGTA----CTGTCTC
CCTTCTCTTAGTATTGGGA-----------TGGTGG----GAGTCTCTGCTT--------
-------CAGAAGACTTCTCT------GGGAGT---GACCCACT---GGTGGTGGGGGTC
ATCCCTAATTCTC---GAAGAGGATCAGTGCCATTGTGAAGGTG----TGATTTGACTT-
GGATGTGAGTTCTTTTT-AAGTGAGACAG--CTGCTCAGA--GTCATCAGCCTCATTCTG
TCCTCTGGAGTC-TTCAAAGTCCATTGATAAGACATA-TATT--AAGTCAGCAAGCCTCT
ATGACTTAT--------TGTTG-------GGGTTTAGAAGGAGCGTAAATAGGGATGGTT
TTCTGTTC---TGCTCAGAAACTCTGAGGGTCTTCCCCTCCCAGATGGATGTTTTT----
-----GTGAAGGTAGATTAGGCCATCTTTCATCTCAATTCTTAGTTATTGAAA-------
---GGGTGTTATCTCAG---ATCCATT---AG-AGCTAACA----GA-------------
TTTTGCTTTTGCCA------A---------------------------------------

-----------------------------GAAA----------TCCAA-GTTAA---AAG
TCATTCTAACGCTTTGTCTGCCAATCTCCACTTTAACCTGGCAGCCTTGTTTTTAGTAGT
AATAGGTTAGTT----GAGC-----TTCTGCCCGAGAGGATCAAGAGTTGGCTTTTTATT
---AGTCTTTTCTTTCTCTA-----CCCTGCAGATATACGACCTCTCTGTTGATGCCTTG
AGC
>Bil
TGGCTCACAGACAGAGCTTGCTATCGTTACTGCAGACTTTGTCAAGGTA----CTGTCTC
C-TTCTCTTGGTGTTGGTACAATGACCCTGTGGTGATTAGGAGCCTCTTCTAGCCAATAG
TGTACTTCAGAAAGCTTCTCAA-----GGGGGT---GACTTACC---TTGTTGTTT----
------A-TTCTC---CAAGAGAACCA---AAGACATGAAGGTG---ATGCTTTGATTT-
GTATGTAAATTGTTTTT-AAGTGAGACTGAGCTGCTTAAAA-GTCCTCAGCCTCACTTTC
AGCTCCAGAGTCATT-GAAGTCC--AGTGACAAGATA-TAAATCAAGAAGACAAGCCTTA
ATGACTTACA------AGGTTTTGA----TGACGTATAATGAGAATAAAAAGCAATTGCT
TTCTGTTC---TGGTCAGAAACTTTGAGGGTCTTCCCTTCCCAAATTAATATTTTT----
-----GTGATGGTAAATTAGACTTTCTTTTGTCTCAATTTTTAGTTCTTGAAA-------
---GTGTGTTGCCTTAG---GTCCA--------------------GATTTTGGTGAC---
TTTTGCCTTTTCCAAGTATGA---------------------------------------

-----------------------------GAAA----------TTCAA-GTTAA---AAC
TCATTCT-ATCCTTCATCTATTAGTCCTCACTTCAGTCTGGCAGCCTTGGTTGTTTTAGC
CATTAAGA-----------------TTCTGCCAGAGAGGACAAAGAATTGACTTCTT---
----GTCTTTTC-TTTTCTA-----CCCTTCAGATATACGACCTCTCTGTCGATGCCTTG
AGC
>Pgu
AGGCTCGCAGACAGAACTTGCTATAGTTACTGCAGACTTTGTCAAGGTA----CTGTTTC
C-TTCTCTTGGTGTTGGTACAATGACCTTGTGGTGATTGAGAGCTTCTTCTAGCCAATAG
TGTACTTCAGAAAGCTTCTGGGGGGGGGGGTGT---GACTTACC---TTGTTGTCT----
------AATTCTC---C-------------AAGCTGTGAAGGTG---ATGGCTTGATTT-
GTATGTAAATTGTTTTT-AAATGAGACTGAACTGCTTAAA--GTCCTCAACCTCACTTTT
AGCCCCAGAGTCC------------AGTGACAAGATA-GAGGTCAAGAAAATAAGCCTTA
ATGACTTACA------AGATTTTGA----TGGCTTATAATGAGAATAAATAGCAATTGTT
TTCTGTTC---GGGTCGGAAACTTTGAGTGTCTTCCCTTCCCAGATTAATATTTTT----
-----GTGATGGTAAATTAGGCTATCCTTTGTCTCAATTTTTAGTTTTTGAAA-------
GAAGGGTATTGCCTTAG---ATCCA--------------------GATTT-CGTAGCTTA
TTTTGCCTTTGCCAAGTATAA---------------------------------------

-----------------------------GAAA----------TTCAA-GTTAA---AAC
TCATTCTAATCCTTCATTTATTAGTCCTCACTTTGGTCTGGCAACCTTGCTTGTTATAGC

TATTAAGA----------------TTCTGCCAGAGAGGACCAAGAGTTAACTTCTTATT
TATAGTCTCTTCTTTTTCTA-----CCCTACAGATATATGACCTCTCTGTTGATGCCTTG
AGC
>Tro
TGGCTCACAGACAGAGTTGGCCATCGTAACTGCAGACTTCGTCAAGGTA----CTTTCTG
C----TCTTAGTGTCGGCATAGTGACCTTGTGGTGATTGGGAGCCTCTGCTAGACAGTGG
TGTGCTTCAGGAAGCTTCTCAGT----GGGGGA---GACCCATG---------------
-----TTTTCTT----GAAGAGGACGAATGACATC-----GGT----ATGTCCTGACTT-
GTCAGCAAATTGTTTTTAAGTGGAGCAGAGCTGCTCAGA--GTTGTCAGCCTCAC----
TCCTTCGGAGTC-ATCCAGGTTA--ACTGATAAGAGA-GAAGTGATGATGAAAAGCCTCT
CTGACTCACT--GTTTAGATTCTGA----TGGCCTACAATGACTGTAAATAGGAGTTGAT
TTCTGTTC---TCATCAGAAGCTTTCAGGGTCTTCCCTTCCCA----------------
-------------------GGCCATCTTTTGTCTCAGTTCTTAGTTTTTGAAA-------
---GGGCATTGCTTCAG---ATACATT---AGGAACTAACA----GATTTAAAGGGGTCA
TTTTGCTTTTGCCAAGCATAA---GAAATACAACTTAA-----GGGGTAGCTAGGTGTCA
CAGTGAATAGAGCACTGGTCCTGGAGTCAAGAGGATCCGAGTTCATATTCAGGCTCAGAC
ACGTAACACTTAACTAGCCGTATGACTCTGGGCAAGTCATTTAACCTCAATTGCCCTGTC
CTCCCCACTCCAGAAAGCAAAAGAAA---GAAA----------TCCAA-GTTAA---AAC
TCAATCTAATCCTTTGTATACTGGTTTCCAACTCAGCTTGGTAGCCTTGGTTGGTTTAGC
TACTGATGAATCAGGT-GAGG----TTCTGCAAGAGAGGACCAAGAGTTGGCTTCTTGCT
T--AGCTTTTTTCCTCTCTA-----CCTTACAGATATACGACCTCTCTGTTGATGCCTTG
AGC
>Ppe
TGGCTCACAGACAGAGCTGGCCATCGTTACTGCAGACTTCGTCAAGGTA----TTCTCTA
C----TCTTAGTGTTGGTATAATGACTGTGTGGTGATTGGGATCCTCTGCTAGTCAATGG
TGTGCTTCAGAAAGCTTCTCAAT----GGGGCT---GACCCATT----GTTGTTGGTCAT
-CTTTTTTTCTT----GAAGAGATCCATTGACATCATGAAGGTG---ATGGCTTAACTT-
GTGAGCGAATTGTTTTT-AAGTGAGGCAGAGCTGCTCAGA--GTTGTCTGCCTCAC----
TCCTCCAGAGTC-ATCCATGTCT--AGTGACAAGATT-CAAGTCATGATGACAAGCCTCT
CTGGCTCACT--GTTTAGGTTTTGA----TGGCCTAGAATGAGTGTAAATAGCGATTGTT
TTCTGTTC---TGATCAGAAGTTTTCAGGGTTTTCCGTTCCCAGATTGATAATTT-----
-----GTGATGGTAAATTAGGCCACCTTTTGTCTCATTTCTTAGCTTTTGAAA-------
---GGGCATTGCTTCAG---ATCTATT---AGGAACTAACA----GA-------------
TTTTGCTTTTGCCAAGCATAA---------------------------------------

-----------------------------GAAA----------TCTAA-GTTAA---AAC
TCATTCTAATCCTTTGTTTACTAGTTTCCAGCTCAGCCTGGCAGCCTTGGTTGGTTTAGC
CACTAATGAATCAGGT-GAGGTTCTTTCTGCCAAAGAGGACCAAGAGTTGGCTTCCTGTT
T--AGTCCTTTTTCTCTCTA-----CCCTACAGATATATGACCTCTCTGTTGATGCCTTG
AGC
>Tvu
TGGCTCGCAGACAGAACTGGCTATCGTTACTGCAGACTTTGTCAAGGTA----CTGTTTC
C-TTCTCTTGGTGTGGGTACAATGACCTTGTGGTGGTTGAGAGCTTCTTCTAGCCAATAG
TGTACTTCAGAAAGCTTCTCAAGGG--GGGGGT---GACTTACC----------------
---TTG---TTGTCTTGATTT-
GTATGTAAATTGTTTTT-AAATGAGACTGAGCTGCTTAAA--GTCCTCAACCTCACTTTT
AGCCCCAGGGTCATTTGAAGTCC--AGTGACAAGATA-GAGGTCAAGAAGATAAGCCTTA
ATGGCTTACA------AGATTTTGA----TGGCTTATAATGAGAATAAATAGCAATTGTT
TTCTGTTC---GTGTCAGAAACTTTGAGTGTCTTCCCTTCCCAGATTAATATTTTT----
-----GTGTTGGTAAATTAGGCTATCCTTTGTCTCAAGTTTTAGTTTTTGAAA-------
GAAGGGTATTGCCTTAG---ATCCA-------------------GATTTC-GTAGCTCA
TTTTGCCTTTGCCAAGTCTAA---------------------------------------

-----------------------------GAAA----------TTCAA-GTTAA---AAC
TCATTCTAATCCTTCATTTATTAGTCCTCACTTCAGTCTGGCAGCCTTGCTTGTTATAGC
TATTAAGA-----------------TTCTGCCAGAGAGGACCAAGAGTTAACTTCTTATT
TATAGTCTCTTCTTTTTCTA-----CCCTACAGATATACGACCTCCCTGTTGATGCCTTG
AGC
>Mro
CAGCTCACAAACAGAACTGGCCATCGTTACTGCAGACTTTGTCAAGGTA----CTCTCTC
C----TCTTAGTGTTGGTACAATGACTGTGTGGTGATTGGGAGCCTCT-GCAAGCCTCAG
AGTGCTTCAGAAAGCTTCTCAAT----GGGGGT---GATCCATT----GTTGTT--CATC
-C-TTTATTCTT----GAAGAGGACCAATTTCATCATGAAGGTG---ATGACTTGACTT-
GTGAGTAAATTGTTTTT-AAGTGAGGCAGAACTGCTCAAA--GTTGTCAGCCTCAC----
TGCTCCAGAGTC-ATTGAAGTCC--AGTGGCAAGACA---AGTCAAGATGACAAGCCTTT
ATGACTCATT--ATTTAGATTTTGA----TGGCCTAAAGTGAGTGTAAATAGCAGTTGTT
TTCTGTTC---TGATGAGAAGCTTTGAGGGTCTTCCCTTCCCAGACTGATATTTTC----
-----CTGATGGTAAATTAGGCCATCTTTTGTCCCAGTTCTTAGTTTTCGAAA-------
---AGGTGTTGCCTCAG---ATCCATT---AGGGACTAACA----GATTTCATTGGCTCA
TTTTGCTTTTGCCAAGCATAA---------------------------------------

-----------------------------GAAA----------TCCAA-GTTAA---AAC
TCGTTCTAATTTTTTGTCCGCTAGTTTCCAGCTCAGCCTGGCATCTTTGGTGGGTGTAGT
CACTAATGAGTCAGG--GAGG------------------ACCAAGAGTTGACTTCTTGTT
T--AGTCCTTTCTCTCTCTA-----CCCTCCAGATATATGACCTCTCTGTTGATGCCTTG
AGT
>Ptr
TGGCTCACAGACGGAGCTGGCCATCGTTACTGCAGACTTTGTCAAGGTA----CTCTCTA
C----TCTTAGTGTTGGCACAATGACTGT--GGTGATTGGGAGCCTCT-GCTAGCCTCAG
AGTACTTCAGAAAGCTTCTCAAT----GGGGGT---AATCCATT----GTTGTT--CATC
-C-TTTATTCTT----GAAGAGGACCCATTTCATCATGAAGGTG---ATGGCTTGACTT-
GTGAGTGAATTGTTTTT-AAGTGAGGCAGAGCTGCTCCAA--GTTGTCAGTCTCAC----
TGCTCCAGAGTC-ATTGAAGTCC--AGTGGCAAGACATTAAGTCAAGATGACAAGCCTTT
ATGACTCATT--ATTGAGATTTTGA----TGGCCTAAAGTGAGTGTAAATAGCAGCTGTT
TTCTGTTC---TGATGAGAAGCTTTGAGGGTCTTCCCTTCCCAGATTGATATTTTC----
-----ATGATGGTAAATTAGGCCATCTTTTGTCTCAATTCTTAGTTTTTGAAA-------
---GGGTGTTGCCTCAG---ATCCATT---AGGGACTAACA----GATTTCATTGGCTCA
TTTTGCTTTTGCCAAGCATAA---------------------------------------

-----------------------------GAAA----------TCCAA-GTCAA---AAC
TCATTCTAATCTTTTGTCTACTAGTTTCCAGTTCANCCCAGCATCCTTGGTGGGTTTAGT
CACTAACGGGTTAGG--GAGG------------------ACCAAGAGTTGACTTCTTGTT
T--AGTCCTTTCTCTCTCTA-----CCCTCCAGATATATGACCTCTCTGTTGATGCCTTG
AGT
>Vur
TGGCTCACAGACAGAGCTGGCCATCGTTACTGCAGACTTTGTCAAGGTA----CTCTCTC
T-TTTTCTTAGTGTTGGTACAGTGACCCTGTGGTGTTTGGGACCCTCTGCTAGCCAACAG
TGTGCTTCAGAAAGCGTCCCAGT----GGGGGT---GACCTATT-GTTGTTGTCATTTAT
-CCTTTATTCTC----CAAGAGTACCAGTGACATCATGAAGGTG---ATGGCTTGACTTG
GCGTGTGAATTGTTTTT-GAGTGAGGCAGAGCTGCTCAAA--GTTGTCAGCCTCACTCTC
TCCTCCAGAGTC-ATCGAAGTCC--AGCGGCAAGACA-TAGGTCAAGATGACAAGCCTCT
GTGACTCATTG-ATT-AGGTTTTGA----TGGCCTGGAACGAGTGTAAATAGTAATTGTT

TTCTGTTC---TGATCAGAAGCTTTGAGGGTCTTCCCTTCCCAGATTGATATTTTT----
-----GTGATGGTAAATTAGGCCATCTTTTGTCTCAGTTCTTAGTTTTTGAAA-------
---GGGCGTTGCCTCAG---ATCCATT---AGGGACTAACA----GATTTCAGTGGCTCA
TTTTGCTTTTGCCAAGCATGA---------------------------------------

-----------------------------GAAA----------TCCAA-GTTAA---AAC
TCATTCTAATCCTTTGTCTACTAGTCTCCAACTCAGCCTGGCAGCCTTGGTTGGTTTAGC
CACTATTGAATCAGGT-GAGG----TTCTTCCAGAGAGGACCAAGAGTTGGCTTCTTGTT
T--AGTCTTTTCTCTCTCTG-----CCCTACAGATATACGACCTCTCTGTTGATGCCTTG
AGC

MARKER 107

>RTESINE2

-------------GAGAGA--GTGAGGCTGATGACTTTGTGCAACTCTGCCTC-ACTTAA
ATCCAATTCATGCGCAAGTCAAGA--CATCACCCC-ATGATGTCATTGGTCCTCTTCGAA
AACGAAGGACGAACAACAACAAC---AA--------------------------------

>Mdo
ACCCGGGTCGTCGATGACAATG--GTGGGCATAGCTTCCCTTCACTCTCCATCCTTCCTG
TGTCAAAATGTGGGAAGAGG----GCCTTCTCCCATATGCTGTA-GGAGAGAATTCCCAA
AGAAGTAGAAATAAGACCCCAGCC-TAACCTTAATAACTCAAAGTTGAAATGGGAGAGAG
TGGAGGGAGTGCCTGAC-ATTGACTGCCACCTGCCTCCC----TCTTTCTTCCTTCCTTT
TCTGGTCATTGTGAGAAATAGAGGGACTCAGAGGGACACTT-ACTTCCCTTTATATTCTT

--------------------------AGTCTTG-------AGGCTGTGGGAGAAGGGAGG
GAAGGAA-------------------GTAACTCCAACGATTCTGGCCA---AAGAAAGTC
ATTCCTCTTGTTGAAATGAGTGGTGATGGGCTTGGGGGCTCTTGTAGATTTTTGGCATAT
GAAAGAGCAAGATTAAAGGG-AAAGAAAGAGAA-----TATCTAGTGA------CCCTGG
AAAAGGACGG--AGATGGAGATGGAATTTGCTTTCTCCTCTGGACTCAAGTCCATCTTGC
CTTTCTCTGACCT-CCAG--GCTTCAACCCTATGTGGGAAGAGACC
>Dvi
ACCCGGGTCGTTGATGACAACG--GTAGGCAGAGCTTCCCTTCATTCTCCATCCTTCCTG
TGTCAGAATGTGGGAAGAGA----GCCTTCTACCATATGCTGTA-GGAGGGAATTCCCAA
AGGAGCAGAGAAAAGGAGACTCCC-TAACCTTAATAACTCAAAGTCAAATTGGGAGAGAA
TGGTGGGAGTATCTGAC-ATTGACTGCCACCTGCCTCCC----TCTTTCTTCCTTCCTTT
TCTGGTCATGGTGAGAAATAGAGGGACTCAGAGGGACACTT-ACTTCCCTTTATATTTTT

--------------------------AGTCTTG-------AGGCTGTGGGAAAAGGGAGG
GAAGGAA-------------------GTAACTCCAGTGATTCTTGCCAAA-GACAAAGTC
ATTCTTCTTGTTGAAATGAATGGTAATGGGCTTGGGGGCTCTTATAGATTTTTGGCATAT
GAAAGAACAAGATTAAAGGG-AAAGAAAGAA-------TATATAGTGA------CCCTGG
AAAAGGAGGG--AGGCAGAGATGGAACTTGCTTTCTCCTCTGGACTCAAGTCCATCTTGC
CTTTCTCTGACCT-CCAG--GCTTCAACCCCATGTGGGAGGAGACT
>Mnu
ACCCGGGTCGTTGATGACAACG--GTGGGCAGAGCTTCCCTTCATTCTCCATCCTTCCTG
TGTCAGAATGTGGGAAGAGA----GCCTTCTACCATATGCTGTA-GGAGGGAATTCCCAA
AGGAGCAGAGATAAGGAGACTCCC-TAACCTTAATAACTCAAAGTCAAATTGGGAGAGAA
TGGTGGGAGTATCTGAC-ATTGACTGCCACCTGCCTCCC----TCTTTCTTCCTTCCTTT
TCTGGTCATAGTGAGAAATAGAGGGACTCAGAGGGACACTT-ACTTCCCTTTATATTTTT

--------------------------AGTCTTG-------AGGCTGTGGGAAAAGGGAGG

GAAGGAA-------------------GTAACTCCAGTGATTCTTGCCAAA-GACAAAGTC
ATTCCTCTTGTTGAAATGAATGGTAATGGGCTTGGGGGCTCTTATAGATTTTTGGCACAT
GAAAGAACAAGATTAAAGGG-AAAGAAAGAGAA-----TATATAGTGA------CCCTGG
AAAAGGAGGG--AGGCAGAGATGGAACTTGCTTTCTCCTCTGGACTCAAGTCCATCTTGC
CTTTCTCTGACCT-CCAG--GCTTCAACCCCATGTGGGAGGAGACC
>Rra
ACTCGGGTTGTGGATGACAATG--GTGGGCTTCGCTTTCCTTCACTCT------GTCCTC
TCT---------------GA----GCCCTGCACTTCGAGCTGTA-GGGGAGATTTCCCAA
GGGAGCAGAGACAATAAA---CCC-TA-CA-TGAGAACTCACAGATGAATTGGGAGA---
-GGGAGGAGGGTCTGAA-TGGCAT------------------------------------
--TGGTCATGATGAGAACTGGAGCGATTCTGAAGG-------GCTTCCCTGTGTATTCTT

--------------------------ACTTAAT---CTTAGGGCTTTGG-AAAAGAGAAG
TGAATGGGGATAATTCCAAT------GACCCTGGGCAAAGGTGTAGTCA---------TC
CCTCTTGGTAAATAGATTGACTTTGGTAGGTCCGGTGACTCTTG-TGGCATCTGACATAT
GAAAGGATGAGATTAAAGGG-AAAGAAAGGGAAA----TCTCTGGTGG--------CTGG
AAAAGGGAGGTGAGTCAGAAATGGAACCTGCTTTCTCCTCTGGGCTCAAGTCCTCCTCAC
TTTGATCTGACCT-CTAG--GCTTCAACCCAATGTGGGAGGAGACC
>Cfu
ACTCGGGTTGTGGATGACAATG--GTGGGCTTCGCTTCCCTTCACTCT------GTCCTC
TCT---------------GA----GCCCTGCACTGCGAGCTGTA-GGGGAGATTTCCCAA
GGGAGCAGAGACAACAAA---CCC-TA-CA-TGAGRACTCACAGATGAATTGGGAGA---
-GGGAGGAGGGTCTGAA-TAGCAT------------------------------------
--TGGTCATGATGAGAACCGGAGCGATTCTGAAGG-------GCTTCCCTGTGTATTCTT

--------------------------ACTTAAT---CTTAGGGCTGTGG-AAAAGGGAGG
TGAATGGGGATAATTCCAAT------GACCCTGGGCAAAGGTGTAGTCA---------T-
------------------------------TCCGGTGACTCTTG-AGTCATCTGACATAT
GAAAGAATGAGATTAAAGGG-AAAGAAAGGGAA-----TCT--GGTGG--------CTGG
AAAAGGGAGGTGAGTCAGAAATGGAACCTGCTTTCTCCTCTGGACTCAAGTCCTCCTCAC
TTTGATCTGACCT-CTAG--GCTTCAACCCAATGTGGGAGGAGACC
>Dgl
ACCAGGGTGGTGGATGACAACG--GTGAGCATCGCTTCCCTTCACTCTCCATCCTCCTTG
TGTCATAACGTGAGGGCAAA----GCCCTCCACTATCTGCTGTA-GGAGAGAATTGCCAA
AGGAGGAGAGATAA---CCCTCCC-CAACCTTTAGAACTCACCTTTGAATTGGGAGAGAG
GGGAGGGCTCATGTGAT-AGTGACTCCCACCTGCTTCCC---------TCACCCTCCTTT
CCTTGA-----TGAGAAT-AGAGGGGAGAATAGGGGGTTCTGGGGGGCATTGATATTCAT
---GGTCTTA---G-GA----GTGGAGCTGATGACCTTTTA-GGCTCTGCCTC-ATGTG-
-CCTCAT-CA---------------------CCCTGGTGATGTCATTGGTTTTCTTCGAG
AACAAAGGATGA-----------------CTG---------------------AGGGAGG
TGAAGATAACCAGAGAGAAAGG---GGTAACTCCAATGGTTCTGGGCAAA-GACACAGTT
ATTCCTCATGCTGGCATGGATTTTGGTGGGCTTAGGGACTCTTACAGACTTTTGGCATGT
GAAAGAACAAGATTAAAGGGAAAGAA-GGGGG--ATAATGAGTGGTG------ATCTTGG
AAAAGGGAGGTGAATCAAAGATGAAACCTGGTTTCTCCTCTGGACTCAAGTCCACCTTGC
TTCTCTCTGGCCT-CCAG--GTTTCAACCCCATGTGGGAGGAGACC
>Pta
ACTCGGGTCGTAGATGACAATG--GTGGGATTCGCTTCCCTTCGCTCTCTATCATCCTTG
GGTCCAAATGTGGGGCCAGA----GCCCTACGGCATCTGCTGTA-GAAGAAAATTCCCAA
AGGAATAGGGAT-----CCCTCCC-TAACTTTTAGAGCTCATAGTTGGATTGGGAGAGTA
GTGCT--------AGAC-AATGATAGCCGT-TGCCTCCC----TTTCCCTTCTTTCCTTT
TCTGGTAATGGTGAGAACTAGATGGACTCTGAGGGGCATTT-GCTTCTCTTTGTATTCTT

```
---AGTCTTA---A-GA----GTGAGTCTGATAATTTTGCCCAGTTCTGCCTC-ATTTAC
ACCCAAATCA-----------AGACATTA-------------CCATTGGTCCTTTTTGAG
AACAAAGGACAAA---CAACCAC---AGTCTTA-------AAGCTGGGG-AA--------
-----------------AAGGG--AGGCGACTCCAGTGATTCTGAGCAAA-GACAAAGTC
ATTCCTTTTACTGGGGTGGATTTTGGGGGGCTAAGGTG--------GAATTTTGGCAGAT
GAAAGAACAAGATTAA-GGAGAAAGAAAAAGG--ATAATATATGGTG-------------
------------ATTCAGAGATGAAACCTGATTTCTCCTCTGGGCCTAAGTCCACTGTGC
CTTTCTCTGGCCT-CCAG--GATTTAACCCTATGTGGGAGGAGACC
>Dgo
ACCCGAGTCGTAGATGACAATG--GTGGGAATTGCTTCCCTGCGCTCTCCATCATCCCTG
GGTCCAA-TGT------GGA----GCCTTGTAGCATCTGCTGTA-GGACAAAATTCCCAA
AGGAAGAGA---T----CCCTCCC-TGACCTTTAAAGCTCACAGTTGGATTGGGAGA---
-----GTAGTGCCAGAC-AATGATAGCCAT-TGCCTCCC-TTTCCAAACTTCCTTCCTTT
TCTGGTAATGGTGAGAACTAGATGGACTCTGAGGGGCATTT-GCTTCTCTTTATATTTTT
---AGTCTTA---G-GA----GTGAGTCTGATAACTTTGCCCAGTTCTGCCTC-ATTTAC
ACCCAAATCA-----------AGACATCA-------------CCATTGATCCTTTTTGAG
AACAAAGGACAAA---CAACCAC---AGTCTTA-------AAGCTGGGAAAA--------
-----------------AAGGG--AGGTGACTCCAGTGATTCTGAGCAAA-AACCAAGTC
ATTCCTTTTACTGGGATGGATTTTGGGGGGCTAAGGTG--------GAATTTTGGCAGAT
GAAAGAACAAGATTAA-GGAGAAAGAAAAAGG--ATAATATATGGTGAGTCAGAGGTTTC
ATCTCTATGGTGAGTCAGAGATGAAACCTGTCTTCTCCTCTGGGACTAAGTCCATTGTGT
CTTTCTCTGGCCT-CCAG--GATTTAACCCTATGTGGGAGGAGACC
>Scr
ACCCGGGTCGTAGATGACAATG--GTGGGAATCGCTTCCCTTCGCTCTCCATCATCCCTG
GGTCCAAATGTGGGCCCAGA----GCC-TGTAGCATCTGCTGTA-GGAGAAAATTCCCAA
AGGAATAGAG-CT----CCCTCCC-TGACCTTTAGAGCTCCCAGTTGGATTGGG------
-------------AGAC-AATGATAGCCAT-TGCCTCCC-----TTTCCTTCCTTCCTTT
TCTGGTAATGGGGAGAACTAGAGGGGCTCTGAGGGGCATTT-GCTTCTCTTTATATTTTT
---AGTCTTA---G-GA----GTGAGTCTGATAACTTTGCCCAGTTCTGCCTC-ATTTAC
ACCCAAATCA-----------AGACATTA-------------CCATTGATCCTTTTGGAG
AACAAAGGACAAA---------A---AGTCTTA-------AAGCTCTGGG-A--------
-----------------AAGGG--AGGTAACTCCAATGATTTTGAGCAAA-GACAAAGCC
ACTCCTTTTACTGGGGTGGATTTTGGGGG-CTGAGGGAATCTTACAGAATTTTGATAGGT
GAAAGGACAAGATTAA-GGAGAAAGAAAAAGG--ATAATGTATGGTGA------------
-------------GTCAGAGATGAAACCTGGTTTCTCCTCTGGGCCTAAGTCCACCATGC
CTTTCTCTGGCCT-GCAG--GATTTAACCCTATGTGGGANGAGACC
>Mfa
ACCCGGGTCGTAGATGACAATG--GTGGGAGTCACTTCCCTTCCCTCTCCATCATCCCTG
GGTCCAAATGTGGGGCCAGA----GCCCTGTAGCATCTGCTGTA-GGAGAAAATTCCCAG
AGGAACAGAGAT-----CCCTCCC-TAACCTTTAGAGCTCATGGTTGGATTAGGAGAGTG
GGGGAGGAGTGCAAGAA-AATGATCCCCAT-CGCCTCCC----TTTCCCTTCCTTCATTT
TCTGGGAATGGTGAGGACTAGAAGGA----------------------------------
------CTTA---G-GA----GTGAGTCTGATAACTCTGCACAATTCTGCCCT-ATTTAC
ACCCAAATCA-----------AGACATTA-------------CCATTGATCCTTTTTAAG
AACAAAGGACAAA---CAACAAC---AGTCTTA-------AAGCTGTGGG-G--------
-----------------AAGGG--AGATGACTCCGATGATTCTGATCAAA-GACAAAGCC
AT-CCTTTTACTGGAGTGGGTTTTGGAGGGCTCAGGGACTCTCACAGAATTTTGGCAGAT
GAAAGAACAAGATTAA-GGAGAAAGAAAAAGG--ATCATATATGGTGA------------
-------------GTCAGAGATGAA-CCACTTTTCTCCTCTGGGCTTAAGTCCACCGTGC
CTTTCTCCGGCCT-CCAG--GATTTAACCCTATGTGGGAGGAGACC
>Bil
ACCCGGGTCGTGGATGACAATG--GTAGGTATTTCTTCCCTTCAT-----ATTGTTCCTC
TATCTAAATGTGGGACTAGA----GCCCTCTACTATCTGCTGTAAGGAGAAAATCTCCAA
```

AGGAGTAGAGACAA---TCCTCCC-TAACCTTTAGACCT------------GGTAGAGCA
GGGAGGGTTTGCCTGAT-GGTGATACCCACCTGCCTCCC----TCCTTCTTCCCTCCTCT
TCTGGT----CTTAGAACTAG---GATTTTGAGGGGCATTT-CCTTCCCTTTATATTCTT
---AGTCTCA---G-GAGTGAGTGAGGTTGATAACTTTATACAACCCTGCCTC-ACTTAA
ACCCAATTCAAATGCAAGACAA---------CCCC-------CTTGTGGTCCTCTTTGAG
AACAAAGTATGAA---AAACATC---AATCTTA-------GGGCTGAGG-AAAAGCAAGG
TGATGATAATCAGAGGCAAGGG--GGATAACTCCAATGATCCTGGAAAAAAGACAAAGCT
ATTCTTCATATCCGAGTGCATTTTGATGGTCTAGGGGACTCTTGCAAACTTTTGGCATAT
GAAAGAACAAAAGTAAAGGGGAGAAAAAAGGG-AACAATATATGG-GG------CCCTGG
GAAAGGGAGGTGAGTCAGAGAAGAAATCGGTTTTCTCCTCTGGGCTCAAGTCCACTTTGT
CTTTCTCTGACCTTCCAG--GCTTCAACCCCATGTGGGAGGAGAC-
>Pgu
ACCCGGGTCGTGGATGACAACG--GTGGGTATTTCTTCCCTTCAC-----ATTTTCCCTA
TATCTAAGTGTGGGACCAGA----GTCCTGTACTAGCTGTTGTA-GGAGAAAATCTCTGA
AGGAGTACAGACAA---CCCTCCC-TAACCTTTAGAACT------------GGTACAGTG
GGGAGGGGTTGCCTGAT-AGTGATATCCATCTGCCTCCC--------TCTTCCCTCCTTT
TCTGGTCATGCTTAGA-C------------AAGGGGGAGT--------CTTTATATTCTT
---AGTCTCA---G-GAGTAAGTGAGGTTGATAACTTTATACAACCCTGTCTC-ACTTAA
ATCCAATTCATATGCAAGTCAAGACAA----CCCC-------CTTGTGGTCCTCTTTGAG
AACAAAGTACGAA---CAACAAC---AATCTTA-------GGGCTGAGG-AAAAGTGAGG
TGATAATAACCAGAGGCAAGGGAGTAGTAACTCCAATGATTCTGGAAAAA-GAGGAAATT
ATTCCAGATATTTGAGTGGATTTTGATGGTCTAGGGGACTCTTGCAGACTTTTGGCATAT
GAAAGAATAAAAGTAAAGGGGAGAAAAAGGGG--ACAATATATGGTGG------CCCTGG
AAAAGAAAGGTGAGTCAGAGATGAAATCTGTTTTCTCCTCTGAGCTCAAGTCCACCTTGC
CTTTCTCTGGCCTTCCAG--GCTTCAACCCCATGTGGGAGGAGAC-
>Iso
ACCCGGNTCGTGGATGACAACG--G-GGGTATTTCTTCCCTTCAC-----ATTTTTCCTA
TATCTCAGTGTGGGACCAGA----GTCCTGTACTAGCTGTTGTA-GGAGAAAATCTCTGA
AGGAGTACAGACAA---CCCTCCC-TAACCTTTAGAACT-------------GTACAGTG
GGGAGGGGTTGCCTGATAAGTGATATCCATCTGCCTCCC--------TCTTCCCTCCTTT
TCTGGTCATGGTTAGA-C------------AAGGGGGAGT--------CTTTATATTCTT
---AGTCTCA---G-GAGTAAGTGAGGTTGATAACTTTATACAACCCTGTCTC-ACTTAA
ATCCAATTCATATACAAGTCAAGACAA----CCCC-------CTTGTGGTCCTCTTTGAG
AACAAAGTACGAA---CAACAAC---AATCTTA-------GGGCTGAGG-GAAAGCGAGG
TGATAATAACAAGAGGCAAGAG---AGTAACTCCAATGATTCTGGAAAAA-GAGGAAATT
ATTCCTCATATTTGAGTGGATTTTGGTGGTCT-GGGGACTCTTGCAGACTTTTGGTATAT
GAAAGAATAAAAATAAAGGGGAGAAAAAGGGG--ACAATATATGGTGG------CCCTAG
AAAAGAAAGGTGAGTCAGAGATGAAATCTGTTTTCTCCTCT--------GTCCACTTTGC
CTTTCTCT--------------------------------------
>Tro
ACCCGGGTTGTGGATGACAATG--GTGGGTATAACTTCCCTTCACTCTCCATCCTCCCTG
GGTCAAAATGTAGGGTCAGAGAGAGCCCTCTGCTGTCCACTACA-GGA------------
-GGAGAAAAGACAA---GCCTCCC-TAGCCCTTAGAACTCACAGCTGAGTTGGAAGAGTG
GGGCGGGCATGCGTGAC-ACTGATGCCCACCTGCCTCCCTCCCTCTTTCGTCCCTCCTCT
TCCAGTCCT-GTGAGAACCAGAGGGACTCTGAGGGGCAT-CCGCTTCCCTTTATATACTT
---TGTCTCA---G-GA----GTAAGATTAAGGACTTTGCACGCACCTGCCTC-ACTTAA
ACCCAATTCATGCACAAGACAA-------------------------------------AAG
AGCCAAGTACGAA---CAACTCC---GGTCTTA-------AGGCTGTGG-CAGAGGGCTG
TGATTATAACCGGAGACAAGTG---CCTCACTACAATGGTTCTGGGCAAA-GACAAAGCC
ATTGCTCGTGCTGGCATCAATTTTAGTGGGCTGGGAAACTT--GCAGAC--TTGGCATAA
GRAAGAACAAGATTAAAGGG-AAATAAAGAGC--ATATTCTATGGGGA------CCCTGG
AAAACAGAGTTAAATCAGAAAGGAAACCTGTTTTCTCCTTTGGGCTTACGTCCACCTTGC
CTTTCTCTGATGT-CCAG--GCTTCAATCCCATGTGGGAGGAGACC

```
>Ppe
ACCCGGGTCGTGGATGACAATG--GTGGGCA---------TTCACTCTCCATCCTTCCTG
TGTCAAAATGTGGGGCCAGA----GCCCTGTGCTGTCTGCTATA-GGAAAGAACTCCCCA
AGGAGAAGAGACAA--ACCCTCCCCCAACCCTTAGAACTCACAGTTGAATTGAGAGAGTG
AGAAGGGTGTGCATGAC-ACTGATGCCCACCTGCCTCCC----TCTCTCTTCCCTCCTTT
TCTGGTCCTGGCAAGAAC-AGAGTACTACTGAGGGG-ATTCTGTTTCCCTTTATATTCTT
---AGTCTCA---G-GA----GTAAGGTTGATGACTTTGTGCACATCTGCTTC-ACTTAA
ACCCAATTCATGCCCAAGTCAAGACAA------------------------------GAG
AACAAAGGATAAG---CAACCAC---AGTCTTA-------GGGCTGTGG-AAGAAGGAAG
TGATGATAACCAGAGGCAAGGG---CATAACTCCAATGATTCTGGGCAAA-GACAAAGCC
ATTCCTCTCACTGGAGTGGATTTTGGTGGACTGGGAAATTC-------------------
----------------AGGG-AAATAAAGGGG--ACAATATATGGGGA------CCCTGG
AAAAGGGAGTTCAGTCAGAGATGAAACCTGTTTTCTCCT-------------CAC-TTGC
CTTTCGCTGACCT-CTAG--GCTTCAACCCCATGTGGGAGGAGACC
>Mro
ACCAGAGTCGTGGATGACAATG--GTGGGCATAGCTTCCCTTGACTCTCCATCCTTCCTG
TGTCAAAATGTGTGACCAGA----GCCCTGTGCTGTCTGCTATA-GGAGACAACTCCCAA
AAGAT-AGAGACAA---CCCTCCC-TAACCTTTAGAACTCTTAGTTGAATTGGGAGAGCA
GGGAGAGAGTGCATGAC-ACTAATGCCTCCCTGCTTCCC----TCTCTCCTTCTTCCTTT
TCTGGTCCTAGGGAGAACCAGAGGGATTCTGAGGGGCAT-CTGCTTCCCTTTATATTCTT
---AGTCTCA---G-GA----GTGAAGTTGATGACTTTGTGCATCTCTGCCTCGACTTAA
ACCCAATTTACGCACGAGTCAAGA--CGTCACCATCGTGATGTCAATGGTCCTCTTCAAG
AACAAAGGATGAA---TAGCCAC---AGTCTTA-------GGGCTGTGG-AAAAGGGAGG
TGATGATAACCAGAGGCAAGG------TAGCTCCAATGATTCTTGACAAA-GACAAAGCC
ATTCCTTGGGCTGGAGTGGATTTTGGTGGACTAGGGAACTCTTGCAGAATTTTGGCATAA
GAAAGAATAAGATTAAAGAG-AAATAAAGGGG--ATAAAATATG-TGA------CCCTGG
AAAAGGCAGTGGAATCACAGATGAAACCTGTTTTCTCCTCTGGGTTCAAGTCCATCTTGC
CTTTCTCTG-GCTTCCAG--GCTTCAACCCCATGTGGGAGGAGACT
>Vur
ACCAGGGTCGTGGATGACAATG--GTGAGCATTGCTTCCCTTCACTCTTCACCCTCCCTG
TGTCAACGTATGGGGCCAGA----ACCCTGTACTGTCTGCTGTA-GGAGAGAACTCCCAA
AGAAGTAGAGATAA---CCCTCCC-TAACCTTTAGAACTCACAGTTGAATTAGGAGAGCA
GGGAGGGCGTGCATGAC-AGTGATGCCCACCTGCCTCCC----TCTCTCTTCCCTCCTTT
TCTGGTCATGGTGAGAATCAGATGGACTCTGAAGGGCAG-TTGCTTCCTTTTACGTTCTT
---AGTCTCA---G-GA----GTGAGGTTGA---CTGTGCACAGCTCTGCCTC-ACTTAA
ATCCAGTTCACGCACAAGTCAAGA-------CCCTCCTGATGTCATTGGTCCTCTTTGAG
AATGAAGGATGAA------CCAA---AGTCTTT-------GGGCTGTGG-AAAAGGGAGG
TGATGGTAACCAGTCACGAGAG-----TAACTCTAATGATTCTGGGCAAA-GACA--GTC
CTTCCTCATGCTGGAGTTGATTTTGGTGGGCTAGGGGACTCTTCATTACTTTTGGCATGC
AAAAGAAGAAGATTAAAGTG-AAATAAAGGGA--ATAATATATGGTGA------CCCTGG
AAAAGGGAGTTGAGTCAAAA-TGAAACCTGTTTTTTCCTCTGGGCTCAAGTC-AC-TTGC
CTT-CTCTG-GCT-CTAG---CTT--ACCCC-TGTGG----AGAAC
```

MARKER 108

>WALLSI1A

--CTAGGTAAAA-GAGGCCATTCTTTGCCTCATTTC
TTACCTAGCCTTAATCACTGAATGGGTGTTGCCTCAGTCAAACTGAGACCTGNGAAA-GACCTTAGCTTAAAAA---GGC
CAAGGTCTCCC-ATTGCATCCTGGGCCATCTCCAGTCATCCTGATCTATATCTTGCCACTGGACCCAGATGGCTCTGGAG
GAGAAAGTGAGGCTGGTGACTTTGCACAGCCCTCCCTCACTTAAATCCA-ATTCACTTGCAAGTCATGKCATCACCTCCC
TGATGTCATGGTCCTCTTTGAGAACAAAGGACAAA---CAACAA------------------------------------
----

>WALLSI1A

--AGAT
TTCTTTCTTAAGGACCATGCCTTAAACCCAAATCACTCTGGCTCTCATTGATTGG----CCAACAATAGGTCCCAGCCCA
AGCCCCACTTGGTCATTGTTTGG--GCCCTGATTGGCTCAGAGTGAATGTAAATAGCAA--TTGTTTCTGTTTTGGCCAG
AAACCCTGAG---GGTCTTCCCCTCCCAGATTGA-------------TTTTTTTTTTTTTTTTGACT-AGGTAAAAGAGG
CCATTCTTTGCCTCATTTCTTACCTAGCCTTAATCAC-TGAATGGGTGTTGCCTCAGTCAA-ACTGAGACCTGNGAAAGA
CCTTAGCTTAAAAAGGCCAAGGTCTCCCATTGCATCCTGGGCCATCTCCAGTCA------TCCTGATCTATATCTTGCCA
CTGGACCCAGATGGCTCTGGAGGAGAAAGTGAGGCTGGTGACTTTGCACAGCCCTCCCTCACTTAAATCCAATTCACTTG
CAAGTCATGKCATCACCTCCCTGATGTCATGGTCCTCTTTGAGAACAAAGGACAAACAACAA------------------
----

>Mdo
CTTATTCCTGACCCTAAAAATGAAAGTAAACAGAAAACCAAAACAATCCGCTCCACGCTAAACCCACAGTGGAATGAATC
ATTCACATT--GTAAG--

----------------------TAGAAGCCTCTTCTTATGT---------------------------------------
----

--------AATAATG--TACCCTT-CTGTATGTGATCTATTCCTGCCCTTAATGAGGTACTTTTATAACAATTAATA--T
TATATATTTAGATTATCCTTATGCCTTTCTGCTTGTGGCTCTCTTCT--CAGATTAATGTATAAACAGAATGTCACTGTT
ATTTTCTCTGTCCTCT----AGAGAGAAAAACT---ATTTCCTGAGAAGTTATGCAGTGAAAAAAAAAATAAAAAAGAAA
GAAAAGAAGTTATGCAGTGAG-ATTGGGGG-TATCATTGGGACC-ATTTTCTCCCTGA-------TTCATGCTGAGATGA
TGAATATGTGCTTTTTTT--CCTCTCTTTCATTCTTTCTAG--CAAATTAAAACCTACAGACAAAGATAGAAGGTTATCT
GTCGAGAT
>Dvi
CTTATCCCTGACCCTAAGAATGAAAGTAAACAGAAAACCAAAACAATCCGCTCCACGCTAAACCCACAGTGGAATGAATC
ATTCACATT--GTAAG--

----------------------TAGAAGCCTCTTCTTATGT---------------------------------------
----

--------AATAATA--TACCCTT-CTGTATGTGATCTATTCCAGCCCTTAATGAGGTGCTTTTATTGTAGTTAATA--T
TATATATTTAGATTATCATTATGCCTTTCTACTTGTGGCTCTCTTTT--CAGGTTAATGTACAAACAAAATGTCACTGTT
ATTTTCTCAGTACTCT----AGAGAGGAAAACT---ATATCCTAAGAAGTTAAGCAGTGAA-AAATATATAAGAAGAAAA
AAAAAGAAGTTATGCAGTGAA-ATTGGGGG-TATCATTGGAACC-ATTTTCTCCCTTA-------ATCATGCTGAGATAA
TGGATATGTGCTTTTTT------CCCTCTCATTGTTTCTAG--CAAATTAAAACCTACAGATAAAGATAGACG-------
----
>Rra
CTTATCCCTGATCCTAAGAATGAAAGTAAACAAAAAACAAAAACCATCCGCTCTACACTAAACCCTCAATGGAATGAATC
ATTCACATT--GTAAG--

----------------------TGGAAGCCTCATCTCTTCT---------------------------------------
----

---TCTCTAATAATA--TGCCCTTCTTATGTGTGCTCTATTGCTGTTTTTAATGAGTAGCATTTATTACAAAT-CAT--T
GTGATATTTAAAATATCCATATGTCTTTCTGC----------------------------------AGAATATCATTT--
ATCTTCTTTATTCTATGAATAAAGAAA----CT---ATTTCCTGAGAAGTTAGGCTCTGAA-------------------
----------------------GTTGGAGA-TATTATTGGGACC-ATTTTCTC---TA-------TTCATGCTGAGATAA
TGAACAGGTGCTTTTTTTT-----CCTCTCACTCTTTCTAG--CAAATTAAAACCTACAGACAAAGATAGAAGATTATCT
GTCGAGAT
>Cfu
CTTATCCCTGATCCTAAGAATGAAAGTAAACAAAAAACAAAAACCATCCGCTCCACACTAAACCCTCAGTGGAATGAATC
ATTCACATT--GTAAG--

----------------------TGGAAGCCTCTTCTCTTCT---------------------------------------
----

---TCTCTAATAATA--TGCCCTTCTTATGTGTGCTCTATTGCTGTTTTTAATGAGAAGCATTTATTACAAAT-CAT--T
GTGATATTTAGAATATCCCTATATCTTTCTGC----------------------------------AGAATATCATTTTT
ATCTTCTTTATTCTCTGAAGAAAGAAA----CT---ATTTCCTGAGAAGTTAAGCTCTGAA-------------------
----------------------GTTGGAGC-TATCATTGGGATC-ATTTCTC----TA-------TCCATGTTGAGATAA
TGAACAGGTGCTTTTTTTTTT----CTCTCACTCTTTCTAG--CAAATTAAAACCTACAGACAAAGATAGAAGATTATCT
GTCGAGAT
>Dgl
CTTATCCCTGACCCGAAGAATGAAAGTAAACAAAAAACCAAAACGATCCGCTCCACACTAAACCCACAGTGGAATGAGTC
ATTCACATT--GTAAG--

----------------------TAGAAACCTCTTCTCTTCT---------------------------------------
----

---CCTCTAATAATG--TGTCCTTCCTGTATATGCTGTATTCCTCTTCTTAATGAGGTGCATTTATTATAGAC-CAT--C
ATGATATTTAGAATATCCCTGTGCCTTTTGGCCTGTGGCTCTCTTCT--CAGGTTAATGGATAAACGGAATGTCACTATT
ATCTTCTCTGTCCTCTATCTAGAGAGAAAAGCT---ATTTCCCGAGAAGTTATGCAGTGAA-------------------
----------------------GTTGGGGA-TATCATTGGGACC-ATTTCTCCCTGC--------TTCATGCTGAGATGA
TGAGTGTGTGCTTTTTTCT------CTATCATTCTTTCTAG--CAAATTAAAACCTACAGATAAAGATAGACGGTTATCT
GTTGAGAT
>Nty
-------------CTAAGAATGAAAGTAAACAAAAAACCAAAACAATCCGCTCCACACTAAACCCACAGTGGAATGAGTC
ATCCACATT--GTAAG---TAGAAACA--TTTTCCCTTCTTCT---CTAATTTAAAAGAGGCTATTCTTTGGGTCATTTC
TCACCTAGCCTTAATCACTAAATGGGCATTGCCTCATTCAGACTGAGACCTGGGAAA-GACCTTACCTTTAGAA---GGT
CAAGGTCTCCC-ATTGCATCTAGGGCTATCTCCAATTATTCTGATCTATAGCTTGCCGCTGGACCCAGATGGCTCCAGAA
GAGAGAATGAGGCTGGTCACTTTGCACAGCCCTCCCTCACTTAAATTCA-ATTCACTTGCAAGTCTTGGCATCACCTTCC
TGATGTCATGGTCTTCTTTGAGAGTGAAAGAGACCCAAC---
---TGGCCATTTCCAGTTGGGCAATGCAGAGCTGCTTCCTTCTTTCCTTCTTTCTTTCCTTCCTTCCTTCCTTCCTCCCT
CCCTCCCTTCCCCTCCCTCTCCTCCCACTGTCCACATAGAACATCATACCCTTACTTG----------------------
----

--TG

GAGGCTCTGCCC--AAAGAAATGTAAATTTTGATCCAAGAT----------------ATTTAGGGTTTATGGGCTAGAT
TTCTTTCTTAAGAAGCATGCCTTTAATCCATATCACTTTGGGTTTCATTGTTTGG----CCAACATTAGGTCTCAACCCA
AGCCCCTTTTGATCACTGTTTGG--ATCCTGAT-GGCTCAAAGTAAGTGTAAATAGAAA--TTGTCTCTGTTTTGACTCA
AAAGCAAAAG---GACCATCCCCTTCCAGACTGA--------------------TTTTTTTTTCCACTAGGTAAAAGGGA
CGATACTTTCCTTCATTTCTCATGTAGCCTTAATCCCCTGTTCAGGCATTGCCTCAGTCAA-ACTGAGACCTGGGAAAGA
CCTTAATTAAAAAAGGCCAAGGTCTTCTAGTACGTCCAGGGATATCTCTGGTCA------TCCTGATCTGTATCTTGTCA
CTGGACCCAGATGGTTCTGGAAAAGACAATGGGATTCATGACTTTACACAGTCCTCCCTCATTTGAATCCAATTCCCTTG
CAAGTCATGGCATCACCTTCATAAGGTCATGGTCCTCCTCAAGAACAAAGAGCAAACAACCGCAGAAA-------CCTCA
TCTTCTCTCATAATG--TATCCTTCCTTTATGTGCATTATTTCTGCTCCTAATGAGGTGCATTTAT--TGCAAACCA-TT
GTGAAAGTTAGAATATTCCTACGCCTTTCTGCCTGTGGCTTTCTTCT--CAGGCTAATGAATAAAC--AATGTCAGTATT
ATCTTCTCTATCCACT----AAAGAGAACAATT---ATTTCCTGAGAAGTTATACA------------------------
----------------------GTTGGGG--TTTCATTGGGACC-ATTTCTCCCTGA-------TTCATGCTGAGATGA
TGAGCGTGTACTTTTTTTT----CCCTTTCATTCTTTCTAG--CAAATTAAAACCTACAGATAAAGATAGACGA------
----

>Pta
CTTATCCCTGACCCTAAGAATGAAAGTAAACAAAAAACCAAAACAATCCGCTCCACACTAAACCCACAGTGGAATGAGTC
ATTCACATT--GTAAG---TAGCAA--CCTCTTCTCTTCTTCT---CTAAGTAAAA-GAGACCATTCTTTACCTTATTTC
TTACCTAGCTTTAATCATTGATTGGGCTTTACTTCAGT-TGCCTGAGACCTAGGAAA-CACTTTAGCTTAAAAT---AAC
CAAGGTCTCCC-TCTGTATCCAGGGATGTCCTAATCTGCAA----------CTTGCTATTGGACCCACATGGCTTTGAAA
GAGAGAGAGTC---------------TGGTCCATTCTCACTTAAATCTAAGTTTACTTCCAAGTCATGGTATCCCCTTCC
TAATGTCATGGTCCTTTTCAAAAATGAAGGAAGTAGAA---------------------GTAGGTGTTAGTTGGGGAAAC
AATTGACTAGTTCCAATTGAGCAATGCAGTTCT--TTCCTTCCTTTCTTCCTTCATTCCTTCATTCCTTCGTTCTTTTCT
TCCTTCTTTCCTTCTTCCCTTATCCCTCCTTTCCTCTTTTCTTCCTTCCTCCCTCCCTCTCTCCCTCCTTCCTTCCNTCC
TCCCTCCTTCTCTCCTCCTTCTCTCCCTCCTTTCTTCCTTCCTCCCTCCTCTCTCATCCTTCATTCNTTCCTCTCTCTTT
CCTCCTTTCCTCCTCCCTCCCTCTTTCNTCCTCCTTCCTTCTTCCNTNATNATNCTCCTCCTTCTTCCTCTTTNGCCTAT
CTTC---TGAGGTTTTCCAAGATTATTCCTTCC
TTCATTTCCAACTGTATTGGTTTCCT-TTTTAAGTGTGGATTTGAGAGTGTTTTTGTACTTTTTGCCCCTGAGTTTATTT
TGTGGTGTTCAACTCAAATAGAACTCCTAGTTACAGCAATTTTCTCAGGGGAAAAAAAATACAGCTTGCTTCCTTCTTTC
CTTTCTTCCTTCTCTTCCTTTCCTTTTTCCCTCCCTTCTTTCCCTCTGTACACATAAGGAATCGTAGACTTACC----TG
CAAGTGCTCGGCACAAAGGAATATAAATTTTGATCATGAAACCTTGGGAGAT-----ATCTTGGAGTTTATGTGCTGGAT
TTCTTTCTTAAGGACCATGCCTTAAGCCCAAATCACTCTGGCTTTCACTAATTAA----CCAGCAATAGGTCCCAGCCCA
AACTTCACTTGGTCATTGTTTGA--GTCTTGAT-ATCTCAGAGTAAGTGTAAATAGTAATTTTTTTTCTATTTTGGACAG
AAACCCTGAG---TTTCTTCCCCTCCTAGATTGA----------------TTTAATTATTTTTTACT-AAGTAAAAGAGA
TG-CTCTTTGCCTCATTTTTTACCTAA---CAATCAC-TGAGTGGGCATTGCCTCAATTAAAACTGAGTCCTGGGAAAGA
CCTTAACTTAAAAAGGCTAAGGCC----ATTTCCAGTTG---------------------TCCTAA------TCTTGCCA
CTGGAACCAGAAGGCTCTGGAAGAAAAAGTGTATCAGGTGACTTTGCACAGCTCTTCCTCAATTAAATCCAATTCGTTTG
GAAGTCATGGCATCATCTTCCTGATGTCTTGGTCCTTTTCAAAAACAAAGGATAAACAACAATAACAGAAA---CCT-CT
TCTTCTCTAATAAGG--TGTACTTCCTCTTTGTGCTCTATTCCTGTTCTTTATGAGGTATATTTAT--TATTAAACCATC
ATGATATTTAGGATATCCTTCTCTTTTTCTGTCTGTGA--CTCTTAT--CAGGTTAATGGATAAATGGAATGTCAACATT
TTCTTCTCTATTCTCT----AAAGAGAAAA-CTACTATTTCCTGAGAAGTTATACAGTGAA-------------------
----------------------GTTGGGGGTATCATTGGGACC-ATTTCTCCCTAA-------TTCATGCTGAGACAA
TGAGAATGTGATTTTTTTT------CTCTCGTTCTTTCTAG--CAAATTAAAACCTACAGATAAAGATAGACG-------
----

>Dgo
CTTATCCCTGACCCTAAGAATGAAAGTAAACAAAAAACCAAAACAATCCGCTCCACACTAAACCCACAGTGGAATGAGTC
ATTCACATT--GTAAG---TAGCAA--CCTCTTCTCTTCTTCT---CTAAGTAAAA-GAGACCATTCTTTACTTTATTTC
TCACCTACTTTTAATCATTGATTGGGCTTTACTTCAGT-TGCCTGAGACCTAGGAAA-GACTTTAGCTTAAAAT---AAC
CAAGGTCTCCC-TCCGCATCCAGGGATGTCCTAATCTGTAA----------CTTGCTATTGGACCCACATGGCTTTGGAA
GAGAGTGAGTC---------------TGGTCCATTCTCTCTTAAATCCA-GTTCGCTTGCAAGTCATGGCATCCCCTTCC
TAATGTCATGGTCCTTTTCAAAAATGAAGGACAAATAATAGAA----------------GTAGGTGTCAGTTGGGGAAAC
AACTGACCAGTTCCAATTGAGCAGTTAAGCTCC--TTCCTTCCTTCCTTCCTTCCTTCCTTNCTTACTTCNTTCCTTNCN
TTCCTTCCTTCCTTCCTTCCTTCCTTCCTTCCTTCCTTCCTTCCTTCCTTCTCTTCCTTCCTTCCTTCTCTAACTT
CCTTCCTTCTCTTCCTTCTCTCCCTTCCTTCCTTCTCTCCCTTCCTTCCTATCTTCCTTCCTTCCTTCCTTCCTTCCTTC
CTTCCTTCCTTCCTTCCTTCCTTCCTTCCTTCCTTCTTGCTTGTTTTCTTTCCTTTCCTTTCCTTTTCTTTATTTCCTTC
CTTCCTTCCTTTCTC--------------------------------------TGAGGTTTTCCAAGATTATTCCTTCC

TTCATTTCCAACTGTATTTGTTTCCTTTTTTAAGTATTTATTTGAGAGTGTTTTTGTACTTTTTACCCCTGAGTTCATTT
TGTGGTGTTCAACTCAAATATAACTCCTAGTTACAGCAATCTTCTCAGGGGGA-AAAAATACAGTTTGCTTCCTTCTTTC
CTTTCTTCCTTCTCTTCCTTTCCTTTTTCGCTCCCTTCTTTCCCTCTGTACACATAAGGAATCATAGACTTACC----TG
CAAGTGCTCTGCCCAAAGGAATATAAATTTTGATCATGAAACCTTGAGAGAT-----ATCTTGGAGTTTATGGGCTAGAT
TTCTTTCTTAAGGACCATGCCTTAAGCCCAAATCACTCTGGCTTTCATTAATTAA----CCAGCAATAGGTCCCAGCCCA
AACTTCACTTAGTCATTGTTTGA--GTCTTGAT-AGCTCAAAGT--GTAAGTAGTAAATTTTTTTTTCTGTTTTGGACAG
AAACCCTGAG---TGTCTTCCCCTCCTAGATTGA------------TCTTTTTTTTTTTTTTTACT-AAGTAAAAGAGA
TCACTCTTTGCCTCATTTTTTACCTAACCTTAATCAC-TGAGTGAGCATTGCCTCAATTAA-ACCAAGTCCTGGGAAAGA
CCTTAACTTAAAAAGGATAAGGC----CATTTCCAGTTG---------------------TCCTAAT------CTTGTCA
CTGGAACCAGAAGGCTCTGGAAGAAAAAGTGTGACTGGTGACTTTACACAGCTCTTCCTCAATTAAATCCATTTCATTTG
GAAGTCATGGCATCATCTTCCTGATGTCTTGGTCCTTTTCAAAAACAAAGAATAAACAACAATAACAGAAA----CCTCT
TCTTCTCTAATAATG--TATACTTCCTCTGTGTGCTCTATTCCTGTTCTTTATAAGGTACATTTAT--TATTAAACCACC
ATGATATTTAGGATATCCCTATCTTTTTCTGTCTGTGA--CTCTTAT--CAGGTTAACAGATAAATGGAATGTCAATATT
TTCTTCTGTATTCTCT----AAAGAGAAAAACTACTATTTCCTGAGAAGTTATACAGTGAA------------------
----------------------GTTGGGGG-TATCATTGGGACC-ATTTTCTCCCTAA-------TTCATGCTGAGACAA
TGAGAATGTGATTTTTTTT------CTCTTGTTCTTTCTAG--CAAATTAAAACCTACAGATAAAGATAGACG-------
----
>Scr
CTTATCCCTGACCCTAAGAATGAAAGTAAACAAAAAACCAAAACAATCCGCTCCACACTAAACCCACAGTGGAATGAGTC
ATTCACATT--GTAAG---TAGCAA--CCTCTTCTCTTTTTTT---CTAAGTAAAA-GAGACCATTCTTTACCTTATTTC
TTACCTAGCTTTAATCATTGATTGGGCTTTACTTCAAT-TGCTTGAGACCTGGGGAA-GACTTTAGCTTAAAAT---AAC
CAAGGTCTCCC-TCTGCATCCAGGGATGTTCTAATCTGTAA----------CTTGCTATTGGACCCAGATGTCTTTGAAA
GAGAAAAAGTG-----------AGTCTGGTCCATTCTCACTTAAATCCAGGTTCACTTGCAAGTCATGGCATCTCCTTCC
TAAAGTCGTGGTCCTTTTTAAAAATAAAGGACAAACAAG-----------------------------------TAGAAGT
AGTTG-TCAGTTCCAGTTGAGCAATGCAGCTCT--TTCCTTCCTTTCTTCCTTCTTTTCTTTGTTCCTTCTTTCTTTTGT
CCCTCTCTCCTTCCCTCCCTCTCTCCCTTCTTTCTCTTCCTCCCTTCCTCTCTCCCTCCCTTTTTCTCTCCCTCCCTCCC
TCCCTCCCTTCCTTCCTTCCTTCCTTCCTTACTTACTTACTTACTTNCTTCCNTCCTTCCCCNTTCCTTNCCTTCCCTCC
CTTCTTCCCTCCTTCCTT-------------------------------CCTTCTTCCTTCTTTCTTCCTTCCTTCCTC
CTTCTTTCCTTTCTC-------------------------------------TGAGGTTTTTCCAAGATTATTCCTCCC
TTCATTTCCAACTGTATTTGTTTCCTTTTTTAAGTAT-----TGAGGGTGTTTTCTTACTTTTTGCCCC-GAGTTCATTT
TGTGGTGTTAAACTCAAATAGAACTCCTAGTTACAGCAATCTTCTCAGGGGCAAAAA-GTACAGCTTGCTTCCTTCCTTC
CTTTCTTCCTTCTCTTCCTT----TTTTCCCTCCCTCCTTTCCCTCTATTCACATAAGGAATCATAGACTTTCC----TG
CAAGTCTCTGCTC-AAAGGAATATAAATTTTGATCACAAAACCTTGGGAGAT-----ATCTTGGAGTTTATGGGCTAGAT
TTCTTTCTTAAGGACCATGCCTTAAGCCCAAATCACTCTGGCTTTCATTGACTAA----CCAGCAATAAGAACCAGCCCA
AACTTCACTTGGTCACTGTTTGA--GTCTTGAA-AGCTCAGGGTAAGTGTAAATAGTAA-TTTTGTTCTATTTTCAACAG
AAACCCTGAG----TTCTTCC-NTCCTAG----------------------------TTTTTTCACT-AAGA--------
-----TCTTGCCTCATTTTTTACCTAACCTTAATCAC-TGAGT-GGCATTGCCTCAATTAA-ACTGAGTCCTGGGAAAGA
CCTTAACTTAAAAAAGCCAAGTTT--------CCAGTTG---------------------TCCTAATCTATATCTTGCCA
CTGGAACCAGAAGGCTCTAAAAGAAAAAGTGTGGCTGGTGACTTTGCACAACCCTTCCTCAATTAAATCCAATTCATTTG
CAAGTCATGGCATCACCTTCCTGTTGTCCTGGTCCTTTTCAGAAACAAAGGATAAACAACAATAACAAA------CCTCT
TCTTCTCTAATAATG--TGTACTTTTTCTTTGTGCTCTATTCCTGTTCTTTATGAGGTACATTTAT--TATTAAACCACC
ATGATATTTAGGATATCCCTATTTTTT-CTGTCTGTGA--CTCTTAT--TGGGTTAATGGATATATGGAATGTCAATATT
TTCTTCTCTATTATCT----AAAGAGAAAAACTAGTATTTCCTGAGAAGTTAAACAGTGAA------------------
----------------------GTGGGGGG-TATCATTGGGACC-ATTTTCTCCCTAA-------TTCATGCTGAGACAA
TGAGAATGTGATTTTTTT------TCTTTCGTTCTTTCTAG--CAAATTAAAACCTACAGATAAAGATAGACG-------
----
>Mfa
CTTATCCCTGACCCTAAGAATGAAAGCAAACAAAAAACCAAAACAATCCGCTCCACACTAAACCCACAATGGAATGAGTC
ATTCACATT--GTAAG---TAGCAA--CCTCTTCTCTTCTTCT---TTAAGTAAAA-GAGACCATTCCTTACCTTATTTC
TTACTTAGCTTTAATCACTGATTGGGCTTTACCTCAAT-TGCCTGAGACCTAGGAAA-GACCTTAGCTTAAAAT---AGC
CAAGATCTCCC-ACTGTATCCAGCGCTCTCCTAATCTGTAA----------TTTGCTATTGGATACAGATGGCTTTGGAA
GAGAGAGTGAGCCTGGTGACTTTGCATAGTCCATCCTCACTTAAATCTA-GTTCACTTACAAGTCCTGGCATCACTTTCC
TAATGTCATGGTCCTCTTCAAAAATGAAGGACGAACAG-----------------------------------TAGAAGT
TGCTGACCAGTTCCAGTTGAGCAATGTGGCTCC---TCCTTCCTTCCTTCCTTCCTTCCTTCCTTCCTTCCTTCCTTCCT
TCCTTCCTTCCTTCCTTCCTTCCTTCCTTCCTTCCTTCCTTCCTTCCTTCCTTCCTTCCTTCCTTCCTTCCTTCCTTCCT

TCCTTCCTTCCTTCCTTCTTTCCTTCCTTCCTTTCTTCCTTCCTTCTTCCTTTCCTTCTTTTTCTTTCTTCCTTCTTTCC
TTCTTTTCTTTCTTCTTTTCT-----------------------------------ATTCTTTCTTTCTTTCCTTTCTTC
CTTCCTTCCTTTTTC--------------------------------------TGAAGTTTTCTGAGATTAT----TCC
CTCATTTCCAACTATATTTGTTTCTTTTTTAAAGTATTGATTTGAGAATGTTTTTGTACTTTTTTGCCCCGAGTTCATTT
TGTGATATTCAACTCAGATAGAACTGCTAGTTACAGCTCTCTTCTCAGGGGCAAAAA-GTACAGCTTGCTTCCTTCCTTC
TTTCCTTCCTTCCTTTTCTT-----TTTCCCTCCCTCCTTTCCCTCTGTACACATGGGGAATCATAGCCTTACC----TG
CAAGTGCTCTGCCCACAGGAATATAAATTTGTCAT------------GAGAT-----ATCTTGGAATTTATGGTCTTAT
TTCTTNNNTAAGGATCATGTCTTAAACCCAAATCACACTGGCTTTCATTCATTAATTGACCAGCAATAGGTCCCAGCCCA
AGCCTCACTTGGTCATTGTTTGA--GCCTTGAT-AGCTCAGAGTAAGTATAAATAGTAA-ATTTTTCTGCTTTGGATAG
AAACCCTGAGAGTCTTCCT---CTCCTAGATTGATTCTTTTTTTTTTTTTTTTTTTTTTTTTT-ACT-AAGTAAAAGCTA
TCACACTTTGCCTCATTTCTTATCTAATCTTAATCAA-TGAATGGGCATTGCCTCAATTAA-ATTGAGTCCTGGGAAATA
CCTTAACTTAAAA-GGCCAAGGC----CATTTCCAGTTG---------------------TCCTAA---------TGCCA
CTGGAACCAGAAGGCTCTGGAAGAGGAAGTGTGGCTGGTGACTTTGCACAGCCCTCCCTCAATTAAATCCAAATCATTTG
CAAGTCATGGCACCATCTTCCTGATGTCCTAATCCTTTTCAAAAACAAAGCATAAACAACAACAGTAACAG---AAA-CC
TTTTCTTCTCTAATA---------------------ATATTCCTATTCTTCATGAGATATATTTAT--TATTAAACCACC
ATGATATTTAGGATATCTCTATATTTTTCTGTCTGTGA--CTCTTAT--TGGGTTAATGGATAAATGGATTGTCAGTAGT
TTCTTCTCTATTCTCT----AAAAAGAAAAACTACTATTTCCTGAGGAGTTATACAGTGAA-------------------
----------------------GTTGGGG-TATCATTGGACC-ATTTTCTCCCTAA-------TTCATGCTGAGACAA
TGAGAATGTGCTTTTTTT---CCCCCTCTTGTTCCTTCTAG--CAAATTAAAACCTACAGATAAAGATAGACG-------
----
>Bil
CTTATCCCTGACCCTAAGAATGAAAGTAAACAAAAAACCAAAACTATCCGCTCCACCTTAAACCCACAGTGGAATGAGTC
ATTCACATT--GTAAG---TAGAAA--CCTCTTCTCTTCTTCT---CTAGGT-AAAAGAGATCATTCTTTCCCTCATTTC
TTACCTAGCCTTAATCACTGAATAGGCTTTGCCTCAGTCAAA--CAGACCTGGGGAAAGACCTTAACTTAAAAA--AAGT
C--AGTCTCCC-ACTGAATCGAAGGCCATCTCCAGTCATCCTGATTTATATCTTGCCACAGAACCCAGATGTCTCTGGAG
AAGAGAGTGAGGTTGATGAGTTTGCACAGCCCTCCCTCTCTTAAATCTA-ATTAACTTGTACATCATAGCATCACCTTCC
TCAGGCCATGGTCCCCTTTGAGAATGAAGGACAAACAACAA----CCTGTGACCAATATCAGCTGCCCCACTGGGGACCC
AACTATCCAGTTCA--TTGAGCAATACAGCTCC--TTCCTTCTTTCCTTCCTTCTCTCCTTCCTTTCTT----CCATCTT
TTCTTCCTTCCCCCACT-----CCCTTCTTTCCCTTCCTCTTCTCCCTCTGTCCAC------------------------
----

---ATAGGGAATCATACACTTGCC----TG
TAGGTGTTCTGCCCAAATGATTGTAAATTTTGCTCGTTGAAACTTTTT---------ATCTTGGGATTTATAGGTTAAGA
TTTCTT---AAGGACCATACCTTAAACCCAAAATACTCTT----TCATTAACTGG----TCAACAATAGGTCCCAACTCA
AGCCTCACTTGGTCATTCATTGATGGTCTTGAT-GGCTCAGAGTAAGTGTAAATAGCAG--TTCTTTCTGTTT-GGCAAG
AAACCCTGAGAATCTTACC---TTCCCAGATTGA----------------TTTCTTTA-TTTGGACT-AGGTAAAAGAAG
CCATTCTTTACCTCATTTCTTATCTAACCTTAATCAC-TGAATAAATGTTACCTCAGTCAA-ACTGAGATCTGGGAAAAA
CCACTATA-------TCCAGGC----CATCTCCAGTCA----------------------TCTGA-TCTATATCTTGCTA
TTGGACCCAGATGGCTCC-AGAGGAGA-GTGAAGCTGGTAACTTTGCACAGCCCTCCCTTACTTAAATTC-----ATTTG
CATATCATGACACCACCTCTGTGATGGCATGTCTCTT--CAAGAATGAAGGACAAATAATAAAAA-------------TC
TCTTCTCTAATAATG--CATCCCTCCTGTGTGTGCTCCATTCTTGTTTTTAATGAGCTATATTTAT--TGCAAATAA-TC
ATGATATTTAGATTATTTCCATGTCTTTATGACTATGATTCTCTTTTTTTAGGGTAACGAATAAACAGAATGTTACTATT
ATCTTCTCTATTCTCT----AAAGAGAACAATG---AATTCCTGAGAAGTAATACAGTGAA-------------------
----------------------GTTGGGA--TGTCATTGGAAC-AATTTCTCTCTGA-------TTCGTGCTGAGATGA
TGAGCATGTGCTTTTCTTT------CTCTCCTTCTTTCTAG--CAAATTAAAACCTACAGATAAAGATAGACG-------
----
>Pgu
CTTATCCCTGACCCTAAGAATGAAAGTAAACAAAAAACCAAAACTATCCGCTCCACCCTAAACCCACAGTGGAATGAGTC
ATTCACATT--GTAAG---TAGAAA--CCTCTTCTCTTCTTCT---CTAGGT-AAAAGAGACCATTCTTTCCCTCATTTC
TTACCTATCCATAACCACCAAACAGGCATTGCCTCAGTCAAGCTGAGACCTGGGGAAAGACCTTAACTTAAAAG----GT
CAAAGTCTCCC-ACTGCATCCAGGGCTATCTCCAGTCATCCTGATTTATATCTTGCCACTGAACGCAGATGGCTCTGAAG
AAGAGAGTGAGGTTGCTTACTTTGCATAGCCCTCCCTCTCTCAAATCTA-ATTAACTTGTACAACATGGCATCACTTTCC

TCATGCCATGGTCCTCTTTGAGAATGAAGAACAAATAACAA----CCTGTGACTAATATCAGCTACCCCACTGGTGACCC
AACTATCCAGTTCA-GTTGAGCAATATAGCTCC--TTCCTTTCCTTCCTTCTTCTCTCCTCCCTTCCTTTCTTCCATCTT
TTTTTTTCCTTCCCATACTCCCTCCTTCTTTCTCTTCCTCTTCTTCCCCTGACCAT------------------------
----

---ATAGGGAATCATACTCTTGCC----TG
TAGGTGTTCTGCCCAAAGGAATGTACATTTTGATTGTTGAAACTTTATAAGAT----ATCTTGGTATTTATAGGTTAAGA
TTTCTT-----------AAGGACAAACTCAAAATACTCTT----TCATTGATTGG----CAAACAGTAGGTCCCAACTCA
AACCTCACTTGGTCGCTGATA----GTCTGGAT-GGCTCAGAGTAAGCATAAATG-AAG--TTGTTTCTGTTTTGGCATG
-AACCCTGAGGATCTTCT----TTCCCAGATTGA----------------TTTC-TTA-TTTGGACT-AAGTAAANGAGG
TCATTCTTTGCCTCATTTTCTATCTTACCTTAATCA--TGAATGAATGTTACCTCATTCAA-ACTGAGACNTGAG-AAAA
CTTTAAA--------TGG--TCTCCCACTATATCCAGGAC
TCTAGTCATCTGATCTCTTAGGGGAGA-GTAAGGCTGGTAATGTTGCACAGCCCTCCCTCACTTAAATCCAATTCACTTG
TATATCATGACACCACCTCTGTGATAATCATGTTTTCTTCAAGAATGAAGAACAAATAACAAAAA----------TCTTT
TCTTCTCTAATAATGCATGTCCCTCCTGCGTGTGCTCCATTTCTGCTCTTAATGAGCTGCATTTAT--TGCAAATAA-TC
ATGATATTTAGATTATTCCCATGCCTTTATGTCTATGATTCTCTTTTT-TAGAGTAATGAATAAACAGAATGTTACAATT
ATCTTCTTTATTCTCT----AAAGAGAACAATG---AATTCCTGAGAACTAATACAGTGAA-------------------
----------------------GTTGGGG--TGTCATTGGGACC-AATTTCTCCCTGA-------TTCTTGCTGAGATG-
--ACCATATGCTTTTTTTT----CCCTCTCATTCTTTCTAG--CAAATTAAAACCTACAGATAAAGATAGACG-------
----
>Iso
CTTATCCCTGACCCTAAGAATGAAAGTAAACAAAAAACCAAAACTATCCGCTCCACCCTAAACCCACAGTGGAATGAGTC
ATTCACATT--GTAAG---TAGAAA--CCCCTTCTCTTCTTCT---CTAGGT-AAAAGAGACCATTCTTTCCCTCATTTC
TTACCTATCCATAACCACCAAACAGGCATTGCCTCAGTCAAGCTGAGACCTGGGGAAAGACCTTAACTTAAAAG----GT
CAAAGTCTCCC-ACTGCATCCAGGGCTATCTCCAGTCATCCTGATTTATATCTTGCCACTGAACCCAGATGGCTCTGGAG
AAGAGAGTGAGGTTGCTTACTTTGCATAGCCCTACCTCCCTCAAATCTA-ATTAACTTGTACAACATGGCATCACCTTCC
TCATGCCATGGTCCTCTTTGAGAATGAAGAACAAATAA-------CCTGTGACTAATATCAGCTACCCCACTGGTGACCC
AACTATCCAGTTCCAGCTGAGCAATATGGCTCC--TTCCTTTCCTTCCTTCTTCTCTCCTCCCTTCCTTTCCTCCATCTT
TTTTTT-CCTTCCCATACTCCCTCCTTCTTTCTCTTCCTCTTCTTCCCCTGACCAT------------------------
----

---ATAGGGAATCATACTCTTGCC----TG
TAGGTGTTCTGCCCAAAGGAATGCACATTTTGATTGTTGAAACTTTATAAGAT----ATCTTGGTATTTATAGGTTAAGA
TTTCTT-----------AAGGACGAACCCAAAATACTCTT----TCACTGATTGG----CGAACAGTAGGTCCCAACTCA
AACCTCACTTGGTCGCTGATG----GTCTGGAT-GGCTCAGAGTAAGCGTAAATGGAAG--TTGTTTCTGTTTTGGCATG
AAACCCTGAGGATCTTTCT---TTCCCAGATTGA----------------TTTCTTTA-TTTGGACT-AGGTAAAAGAGG
TCATTCTTTGCCTCATTTTCTATCTTACCTTAATCA--TGAATGAATGTTACCTCATTCAA-ACTGAGACCTGGGAAAAA
CCTTAAA--------TGG--TCTCCCACTATATCCAGGAC
TCTAGTCATCTGATCTCTTAGGGGAGA-GCGAAGCTGGTAATGTTGCACAGCCCTCCCTCACTTAAATCCAATTCACTTG
TATATCATGACACCACCTCT--GATAATCATTTTTTCTTCAAGAATGAAGAACAAATAACAAAAA----------TCTTT
TCTTCTCTAATAATGTGTGTCCCTCCTGCGTGTGCTCCATTTCTGCTCTTAATGAGCTGCATTTAT--TGCAAATAA---
-TGATATTTAGATTATTCCTATGCCTTTATGTCTATGATTCTCTTTTT-TAGGGTAATGAATAAACAGAATGTTACAATT
ATCTTCTTTATTCTCT----AAAGAGAACAATG---AATTCCTGAGAACTAATACAGTGAA-------------------
----------------------GTTGGGGG-TGTCATTGGGACC-AATTTCTCTCTGA-------TTCTTGCTGAGATG-
--ACCATATGCTTTTTTT--CCCCCCTCTCATTCTTTCTAG--CAAATTAAAACCTACAGATAAAGATAGACG-------
----
>Tro
CTTATCCCTGACCCTAAGAATGAAAGTAAACAAAAAACCAAAACCATCCGCTCCACACTAAACCCACAGTGGAATGAGTC
ATTCACATT--GTAAG---TAGAAA--CCTCTTCT---CTTCT---CTAGATAAAA-GAAGTTCTTCTTTGCCTCTTTTT

```
TTACCTAACCTTACTCACTAAATGG-CATTGCCTCAGTCAAAATGACACCTGGGAAA-GACAATAATTTAAAAG---GGC
CAAGGTCTCCCTGCTACATCCAGGGATATCTCCATGTGTCCTGATCTATCTCTTGCCACTGGACCCAGATGGCTCAGGAA
CAGAGCGTAAGGCTAGTGACTTCTCAGAGCCCTCCCTCACTTCAATCCA-ATTCATTTTCAAGTCATGGCATCACCTTCT
TGATGTTATGGCCCTCTTTGAGAATGAAGGACCAAA--------------------------------------------
----------------------AAAAAATCTCT--TCTCATCTTTT----------------------------------
--------------------------------------------------------------------------------
--------------------------------------------------------------------------------
--------------------------------------------------------------------------------
--------------------------------------------------------------------------------
--------------------------------------------------------------------------------
--------------------------------------------------------------------------------
--------------------------------------------------------------------------------
--------------------------------------------------------------------------------
--------------------------------------------------------------------------------
--------------------------------------------------------------------------------
--------------------------------------------------------------------------------
--------------------------------------------------------------------------------
--------------------------------------------------------------------------------
--------------------------------------------------------------------------------
--------------------------------------------------------------------------------
------CTAATAATG--TTTCCTTTCGATATTTGTTCCATTCCTCCTCTTAATGA-GTGCATTTTT--TGCCAAACCA--
-TTATATTTAGAATATCCCTATGCCTTTTTGCCTGTGACTCTCTTCT--CAGGTTAATGGATAAATAGGATGTCACTATT
ATCTTCTCTATCCTCT----AGAAAGAAAAACT---ATTTCCTGAGAACTTATGCAGTGAA-------------------
----------------------GTTGTAGG-TATCATTGGGATCCTTTTTCTTCCTGA-------TTCATTCTGAGATGA
TGAATGCATGCTTTTTTTT----CCCTCTCATTCTTTCTAG--CAAATTAAAACCTACAGATAAAGATAGACGGTTATCG
GTTGAGAT
>Ppe
CTTATCCCTGACCCTAAGAATGAAAGTAAACAAAAAACCAAAACCATCCGCTCCACACTAAACCCACAGTGGAATGAGTC
ATTCACGTT--GTAAG---TAGAAA--CCTCTTCT-----------CTAGGTAAAAAGAAGCCCTTCTTTGCCTCATTTC
TTACCTAACTTAACTCACTGAATGGGCATTGCCTCAATCAAACTGAGA------------------CTTAAAAA---GGC
CAACGACTCCC-ACTGCATCCTGGGACATCTCCACTTGTCCTGACCTATATCTGGCCACTGGACCCAGATAACTCTTGAG
GAGA--GTGAGGCTAGTGACTTTGCATAGCCCTCCCTCACTTAAATCCA-ATTCATTTGCAAGTCATGGCATCACCTTCT
TGATATCATGGTCCTCTTTGAGAATGAAGGACAAACAACAA---------------------------------------
----------------------TAGAAATCTCC--TCTCATCATCT----------------------------------
--------------------------------------------------------------------------------
--------------------------------------------------------------------------------
--------------------------------------------------------------------------------
--------------------------------------------------------------------------------
--------------------------------------------------------------------------------
--------------------------------------------------------------------------------
--------------------------------------------------------------------------------
--------------------------------------------------------------------------------
--------------------------------------------------------------------------------
--------------------------------------------------------------------------------
--------------------------------------------------------------------------------
--------------------------------------------------------------------------------
--------------------------------------------------------------------------------
--------------------------------------------------------------------------------
--------------------------------------------------------------------------------
------TTCATAATG--TGTCCTTCCTGTATGTGCTCTATTCCTGCTCTTAATGATGTGTATTTTT----ACAAGCTGTC
ATGATTTTTAGAATATCCCTATGCTTTTCTTCCTCTGACTCTCTTCT--CAGGTTAATGGATAAACAGAATGTCACCATT
ATCTTCTCTATCATCT----AGAAAGAAAAACT---ATGTCCTGAGAAGTTATGCAGTGAA-------------------
----------------------GTTGTGGG-TATCATTGGGACCTTTTTTCTCCTTGA-------TTCATGCTGAGATAT
TAAGTGTGTGCTTTTTTCTCTCTCTCTCTCATTCTTTCTAG--CAAATTAAAACCGACAGATAAAGATAGACGGTTATCT
GTTGAGAT
```

>Mro
CTTATCCCTGACCCTAAGAATGAAAGTAAACAAAAAACCAAAACGATCCGCTCCACATTAAACCCACAGTGGAATGAATC
ATTCACATT--GTAAG---TAGAA--CCCTCTTCT---CTTCT---CTAGGTAAAAAGAGGCCATTCTTTGCCTCATTCC
TTATCTAGTTTTAATCACGGAATGGACATTGCTTCAGTCAAACTGAGACCTGGAAAA-GACCATAACTTAATAAAGAGGC
CAAGGTCTCCC-ACTGCATCCTAGGACATCTCCAGTTGTCCTGATCTATATCTGGTTGCTGGACCTAGATGACTCTGGAG
GAGAGAGTGRAGCTGGTGATTTTGCAGAGCCCTCTATCACTTTAATCTA-ATTCACTTGCAGTTTATGCCTTCACCTTCC
TGATGTCATGGTTCTCTCCAAGAAAGAAGGACAACCAACAACAA------------------------------------
----------------------TAGAAATCTCT--TCTTGTCTTCT----------------------------------
----

------CTAATAAGG--TGTCCTTTCTGTACATGCTCTATTCCTGCTCTTAATGACATGTATTTTT----ACAAACCATC
ATGATATTTGGAATATCCCTATGCCTTTCTCCTTGTGGCTCTCTTCT--CAGGTTAATGGATAAACAGAATGTGACTATT
ATCTTCCCTATCCTCT----AGAAAGAAAAACT---ATTTCCTGAGAAGTTATACAGTAAA-------------------
----------------------GTTA-GGG-TATCATTGGGACC-ATTTTTTCCTTGA-------TTCATGCTGAGATGA
TGAGTGTGTGATTTTTTT-----CTCTCTCATTCTTTCTAG--CAAATTAAAACCTACAGATAAAGATAGACGGTTATCT
GTTGAGAT
>Ptr
CTTATCCCTGACCCTAAGAATGAAAGTAAACAAAAAACCAAAACGATCCGCTCCACATTAAACCCACAGTGGAATGAGTC
ATTCACATT--GTAAG---TAGAAA--CCTCTTCT---CTTCT---CTAGGTAAAAAGAGGCCATTCTTTGCCTCATTCC
TTATCTAGTTTTAATCATGGAATG------------------------CCTGGGAAA-GACCATAACTTAATAAAGAGGC
CAAGGTCTCCC-ACTGCATCTGAGGACATCTCCAGTAGTCCTGATCTATATCTGGTCACTGGACCCATATGGCTCTGGAG
AAGAGAGTGAGGCTGGTGATTTTGCAGAGCCCTTCATCACTTTAACCCA-ATTCACTTGCAATTCATGGCTTCACCTTCC
TGATGTCATGGTTCTCTC--------------AACCAGCAATAA------------------------------------
----------------------TAGAAATCTCT--TCTTGTCTTCT----------------------------------
----

------CTAATAATC--TGTCCTTTCTGTGTGTGCTCTATTCCTGGTCTTAATGAAGTGTATTTTT----ACAAACCACC
ATGATATTTAGAATATCCCTATGACTTTCTCCTTGTGGCTC--TTCT--CAGGTTAATGGATAAACTGAATATCACTATT
ATTTTTTCTATCCTCT----AGAAGAAAAAACT---ATTTTCTGAGAAGTAATACAGTAAA-------------------

----------------------GTTA-GGG-TATCATCGGCAC----TTTCTCCCTGAGAGGATCTCCCTCCTGAGATGA
TGAGTGTGTGACTGTTTT-----TTCTCTCGTTCTTTCTAG--CAAATTAAAACCTACAGATAAAGATAGACGGTTATCT
GTTGAGAT
>Vur
CTTATCCCAGACCCTAAGAATGAAAGTAAACAAAAAACCAAAACAATCCGCTCCACACTAAACCCACAGTGGAATGAGTC
ATTCACTTT--GTAAG---TAGAAA--CCTCTTATCTTCTTCT---CTAGGTAAAAAGAGGCCATTCTTTGCCTCATTTC
TTACCTAGCCCTAATCAATGAATGGGCATTGCCTCAGTCAAATTGACACCTGGGAAA-GACCTTAACTTAAAAT---GGC
CAAGGTCTCTC-ACTGCATCT-GAACCATCTCCAATTGTCCTGATCTATATCTGGCCACTGGACCCAGATGGCTCCAGAG
GAGGGAGTGAGGCTGGTGACTTTGCACAGCCCTCCCTAACTTAAATCCA-GTTCCCTTGCATGTCATGGCATCACCT-CC
TGATGTTATGGTCCTCTTTGAGAAAGAAGGACAAACAACAACAA------------------------------------
----------------------TAGAAACCTCT--TCTCTTCTTCT----------------------------------
----

------CTACTGATG--TGTCCTTCCTATATATGCTCTATTCCTGCTCTTAAAGAGGTGTATTTATT---ATAAACCATC
CTGATGTTTAGAATATCCCTATGCCTTTCCACCTGTGGCTCTCTTCT--TAGGCTAATGGATAAGCGGAATGTCACTATT
ATCTTCTCTATCCTCT----AGAAAGAAAAACT---ATTTCCTGAGAAGCTCTGCAGTGAA-------------------
----------------------GTTGGGG-TATCATTGGGACC-ATTTCTCCCTGA-------TTCATGCTGAGATGA
TTAGTGTGTGCTTTTTTTT---TCCCCCTCATTCTTTCTAG--CAAATTAAAACCTACAGATAAAGATAGACGGTTATCT
GTCGA---

MARKER 122

>WALLSI1A

--GGGGTTTACTGGCTAGATTTCTTTCTTAAGGACCAT
GCCTTAAACCCAAATCACTCTGGCTCTCATTGATTGGCCAACAATAGGTCCCAGCCCAAGCCCC----ACTTGGTCATTG
TTTGGGCCCTGATTGGCTCAGAGTGAATGTAAATAGCAATTGTTTCTGTTTTGGCCAGAAACCCTGAGGGTCTTCCCCTC
CCAGATTGATTTTTTTTTTTTTTTTGACTAGGTAAAAGAGGCCATTCTTTGCCTCATTTCTTACCTAGCCTTAATCACTG
AATGGGTGTTGCCTCAGTCAAACTGAGACCTGNGAAAGACCTTAGC-TTAAAAAGGCCAAGGTCTCCCATTGCATCCTGG
GCCATCTCCAGTCA-TCCTGATCTATATCTTGCCACTGGACCCAGATGGCTCTGGAGGAGAAAGTGAGGCTGGTGACTTT
GCACAGCCCTCCCTCACTTAAATCCAATTCACTTGCAAGTCATGKCATCACCTCCCTGATGTCATGGTCCTCTTTGAGAA
CAAAGGACAAACAACAA---

>Mdo
AAATCATCAATGGGCTAGTGAGCCTGTTGCTGCACCTGCAGGACCCCAAGCCCTCCGTCGTCCGT--GTGAGTGCCGGGG
CGAGGGGGGCTGCCGGGCCG---------GGGAGGCGTGGGCTTCTGGGCGCCC-----GCTCCCGCCGCCG--CTCACT
ACATGTCTAGTGGCTGC---

--TGGCTCAGGAGCCC--GG
GGAGCCCCCTCAGCCC-TGCTTGCTCT-GCTTCCAG--GCCTGTAAGTTTGCCCTGCGCATGTGCGGCCCTAATATGGAA
TGTGACGAGCTCACAGACTTGTTTGAGAGGCAGCTGCATGACGGGCGTGGGCTA
>Rra
AGATCATCAATGGGCTAGCAAGCCTCCTACTGCACCTACAGGACCCCAAGCCCCATGTCGTTTCT--GTGAGTGACCTGA
GCAGGGCATAAGGGTGG-----------CATGTAGTCCAGGCTAATGGAGATGC----TGGAGAAGGAACAC--CACCAG
ACATCCTCAGTGGATGG---

-----------------------GTGGTGGT---GTCAAGCAAGGGAGGTGGGAGCTTCCACAGTG----CCCTGCTCCC
CAGAGGTGCACTTTGGATTGGACAGCAACTGCAG-------ACTAAAGGCGAGGGGGTTTCCAGGGTGAGGAGCGT--GG
G-AGCCCTCTCAGGCCGTGC-TCGCTCTGCTTCTAG--GCCTGTAAGTTTGCCCTGCGAATGTGTGGCCCTAATGTGGAA
TGTGAAGAGCTAGCAGCCTTTTTTGAGAAGCAACTGCATGACGGGCGTGGGCTA
>Cfu
AGATCATCAATGGGCTGGCGAGCCTCCTACTGCACCTACAGGACCCCAAGCCCCATGTCGTTTCT--GTGAGTGACCTGA
GCAGGGCATAAGGGTGG-----------CATGTGGTCCAGGCTAATGGTGATGC----TGGAGAAGGAACAT--CACCAG
ACATCCAAAGTGGATGG---

-----------------------GTGGTGTT---GTCAAGCAAGGGAGGTGGGAGCTTCCACAGTG----CCCTGCTCCC

CAGAGGTGCACTGTGGATTGGACAGCAGCTGCAG-------ACTAAAGGTGAGGGGGTTTCCAGGCTGAGGAGCAT--GG
G-AGCCCTCTCAGGCTGTGC-TCGCTCTGCTTCTAG--GCCTGTAAGTTTGCCCTGCGCATGTGTGGCCCTAATGTGGAA
TGTGAAGAGCTAGCAGCCTTTTTTGAGAAGCAACTGCATGACGGGCGTGGGCTA
>Dgl
AGATCGTCAATGGGCTGGTGAGCCTGTTACTGCACCTGCAGGACCCCAAGCCCCCTGTTGTCAGT--GTGAGTAACAAGG
GGGAGGGGAGGGGC-----------------GGGGCAGAGGGTGATCTTTCTGC----TAGGGAGGGACTAG--TCCCAA
CTGTCTAC--

-----------------------GGGGTGTT------------------ACCAAGCTTCTAA-GTGCC-TGC-TGCCCAG
CCAGTGTGCTTTTTGAATTGGATGGCAGCTATAG-------ACTAAATGGGAGGGGATTAACAGGGTGAAGAACAT--GG
GGTACCTTCTGAGCCCTTTGCTTGCTCTGCTTCCAG--GCCTGTAAGTTTGCCCTTCGCATGTGTGGCCCTAATATGGAA
TGTGAAGAGCTAGCAGCCTTGTTTGAGAAGCAGCTGCATGATGGGCGTGGGCTA
>Nty
AGATCATCAGTGGGCTGGTGAGCCTGTTACTGCACCTACAGGACCCCAAGCCCCCTGTTGTCCAT--GTGAGTAACAAGA
ACAAGGGGACAAGA--------------AGTGGGGCATAAAGTAATCTGGGAGGGAGGGAGGGAGGCACTAG--TATCAA
CTATCAGTGTGGGTGTT---

-----------------------ACCAGGCTT----GGAGAGGTATG------AGCTTAC---CTGC--TGCTTCCCCAT
CCAACATGCTTTTTGGATTGGATGACAGCTATAG-------ACTAAATGTGAGGGGATTTCCAGGATGAAGAACAT--GG
GGCACCCTCTGAGCCCATGC-TTACTCTACTTTCAG--GCCTGTAAGTTTGCCCTGCGCATGTGTGGCCCTAATATGGAA
TGTGAAGAGCTAGCAGCCTTGTTTGAGAAGCAGCTGCATAATGGGCGTGGGCTA
>Pta
AGATCATCAACGGGCTGGTGAGCCTGTTACTGCATCTGCAGGACCCCAAGCCCCCCGTTGTCAGT--GTGAGTAACTGGG
CAAGGGCCA---------------------------GGGGGCCGGACGGCCTGT----TAGGGAGGGA------------
----

---GCAGTGCCGGCCG-CCCGGAGG-TGAGGGGAGCGCGG
GGACCCCTCTGACCCGGCGCGTGTTCC-GCTTGCAG--GCCTGTAAGTTTGCCCTGCGCATGTGTGGCCCTAATATGGAA
TGTGAAGAGCTAGCAGCCATGTTTGAGAAGCAGCTGCATGATGGGCGTGGGCTG
>Dgo
AGATCATCAACGGGCTGGTGAGCCTGTTACTGCATCTGCAGGACCCCAAGCCCCCCGTTGTCAGT--GTGAGTAACTGGG
CAAGGGCTG---------------------------GGGGGCCGGACGGCCTGT----TAGGGAGGGAG-----------
----


```
--------------------------------------------------------------------------------
--------------------------------------------TGGTGCCGGCCGCCCCGGAGG-TGAGGGGAGTGCGG
GGACCCCTCTGACCCAGCGC--GTTCC-GCTTGCAG--GCCTGTAAGTTTGCCCTGCGCATGTGTGGCCCTAATATGGAA
TGTGAAGAGCTAGCAGCCATGTTTGAGAAGCAGCTGCATGATGGGCGTGGGCTG
>Scr
AGATCATCAATGGGCTGGTGAGCCTGTTACTGCATCTGCAGGACCCCAAACCCCCCGTTGTCAGT--GTGAGTAACCAGG
CAAGGGCCA--------------------------AGGGGCCGGATGGGCCTGC----TAGGGAG---------------
--------------------------------------------------------------------------------
--------------------------------------------------------------------------------
--------------------------------------------------------------------------------
--------------------------------------------------------------------------------
--------------------------------------------------------------------------------
--------------------------------------------------------------------------------
--------------------------------------------------------------------------------
--------------------------------------------------------------------------------
--------------------------------------------TGGTGCCGGCCGCCCCGAAGG-CGAGGAGAGCGCAG
GGACCCCTCTAACCCGGCGTGTGTTTC-ACTTGCAG--GCCTGTAAGTTTGCCCTGCGCATGTGTGGCCCTAATATGGAA
TGTGAAGAGCTAGCAGCCATGTTTGAGAAGCAGCTGCATGATGGGCGTGGGCTG
>Mfa
AGATCATCAACGGGCTGGTGGGCCTGTTACTGCATCTGCAGGACCCCAAGCCCCCCGTCGTCAGT--GTGAGTAACCAGG
CGAGGAACG--------------------------GGAGGCCGGACGGGCCTGCTAGGAAGGGAGCAGTGC---------
--------------------------------------------------------------------------------
--------------------------------------------------------------------------------
--------------------------------------------------------------------------------
--------------------------------------------------------------------------------
--------------------------------------------------------------------------------
--------------------------------------------------------------------------------
--------------------------------------------------------------------------------
--------------------------------------------------------------------------------
-------------------------------------------TGGCCGGCGGCCGGCCCCGAGG-TGAGGGGGGCGCGG
GG-CCCCTCTGACCCGACGC--GCTCC-TCTTGCAG--GCCTGTAAGTTTGCCCTGCGCATGTGTGGCCCTAATATGGAA
TGCGAAGAGCTAGCAGCCATGTTTGAGAAGCAGCTGCATGATGGGCGTGGGCTG
>Bil
AGATCATCAGCGGGCTGGTGAGCCTGTTACTGCACCTGCAGGACCCCAAGCCCCCTGTTGTCAGT--GTGAGTAACCAGG
ATCATGGGACAGTG-------------------GGAATAGGGTCATCCTTGGGT----TAGGGAGGGACTAG--CACCAA
CTCTTCACGGGGGTGTG--GCCCAGCTG----------------------------------------------------
--------------------------------------------------------------------------------
--------------------------------------------------------------------------------
--------------------------------------------------------------------------------
--------------------------------------------------------------------------------
--------------------------------------------------------------------------------
--------------------------------------------------------------------------------
-----------------------------------------------------------------AGGAGCAGGGAGCCG
GTAAGAGCTTTTTGG--------------------------------ACAACTCTGAACTCAAGGGTGAAGAAAAT---G
GGAGTGCTCTGAGACCATGCTTGCTCT-GCTTCCAG--GCCTGTAAATTTGCCCTACGCATGTGTGGCCCCAACATGGAA
TGTGAAGAGTTGTCCATCTTGTTTGAGAAGCAGCTGCATGATGGACGTGGGCTT
>Pgu
AGATCATCAGCGGGCTGGTGAGCCTGTTGCTGCACCTGCAGGACCCCAAGCCCCCTGTTGTCAGT--GTGAGTAAC----
--------------------------------------------------------------------------------
--------------------------------------------------------------------------------
--------------------------------------------------------------------------------
--------------------------------------------------------------------------------
--------------------------------------------------------------------------------
--------------------------------------------------------------------------------
--------------------------------------------------------------------------------
```

```
--------------------------------------------------------------------------------
-------------------------------------------------------------------------CAGGGTG
GGCAGAGGGTCTTCATG----------GCGGGTGATCTGACAG----AGGAGCAGGGACTCAAGGGTGAAGCCCAT---G
GGAGTG--CTGACCC-GCGCTTGCTC--CCTTCCAG--GCCTGCAAGTTTGCCCTGCGCATGTGTGGCCCCAACATGGAA
TGTGAAGAGCTGTCCATCTTGTTTGAGAAGCAGCTGCATGATGGACGTGGGCTA
>Iso
AGATCATCAGCGGGCTGGTGAGCTTGTTGCTGCACCTGCAGGACCCCAAGCCCCCTGTTGTCAGT--GTGAGTAACCAGG
AATGGGG-------------------------------------------------------------------------
--------------------------------------------------------------------------------
--------------------------------------------------------------------------------
--------------------------------------------------------------------------------
--------------------------------------------------------------------------------
--------------------------------------------------------------------------------
--------------------------------------------------------------------------------
--------------------------------------------------------------------------------
-------------------------------------------------------------------------CAGGGTG
GGCAGAGAGTCTCCATG----------GCGGGTGATCTGGCCG----AGGAGCAGGGACTCAAGGGTGAAGCCCAT---G
GGAGCG--CTGAGCCTGCGCTTGCTC--CCTTCCAG--GCCTGCAAGTTTGCCCTGCGCATGTGTGGCCCCAACATGGAA
TGTGAAGAGCTGTCCATCCTGTTTGAGAAGCAGCTGCATGATGGACGTGGGCTA
>Tro
AGATCATCAATGGGCTGGTGAGCCTGTTACTGCACCTGCAGGACCCCAAGCCCCCTGTTGTCAGT--GTGAGTAACAAGG
GGAAGGGGAGGGGG--------------------GTGGAGGGGAGTCTTTTTTC----TTAGGAGGAACTCG--CACCAA
TTATCCCTGGGAGCATG---ACCAGGCT----------------GGGCTTTACTGGCTAGACTTCTTTCTCAAAGACCAT
GCCCTAAATCGTATTCACTCTGGGTTTCATAGACTGGACAGCATGTAGGTCCCTGCCCAAACACCGCTTAATGGTTTCTT
TTGGGGGGGGTCC-----------------------AGCTGTTTTCTCTTGTGGCCAGCAACCCTGAAGACTTTTCCCTC
CCAGTTTAATTTTTAAATTTTTA---------TTAAAGGGGACAAACCACA-----------------------------
--------------------------------------------------------------------------------
--------------------------------------------------------------------------------
--------------------------------------------------------------------------------
-------------------------CACGAG-------------TAAGTCTGGAGAGCCGTGAGCAC---CTCTCCTAGT
CCAGTTTGCTTTCTGGATTGGA---CAGCAGTTAGTTGTAGACTGAATGTGAGGGGATTTCCAGGTTGAAG---------
---ACC--CTGAGCCCACGCTTGCTCTTGTTTCCAG--GCCTGTAAGTTTGCCCTGCGCATGTGTGGCCCCAACATGGCA
TGTGAAGAGCTGACGACCTTGTTTGAGAAGCAGCTGCATGATGGGCGCGGGCTA
>Ppe
AGATCATCAATGGGCTAGTGAGCCTGTTACTGCACCTGCAGGACCCCAAGCCCCCTGTTGTCAGT--GTGAGTAACAAAG
GCAAGCAGATGGGGTGGGGCGGGGGGGAGGAGGGGAATAGGGGAATCTTTTTGC----TAGGGAGGGACTAG-TTATTGA
CTATCCGTGGGAGCGTT---ACCAGGTT----------------GGGGTTTACTGGATAGATGTCTTTCTCGAAGACCAT
GCCTTAAACCCTGTTCACTCGGGATCTCATTGACTGGACAACA-GTAGGGCCCTGCCCAAGC--------AGGGTTATTT
TTTGA-GTCCTCCTGGCTCAGAGTAAATGTAAATAGTAAC--TTTCTCTTTTGGCCAGCAACCGTGAAGATTTTCCCCTC
CCAATTTGATTTTGTAATATTTA---------TTCAAGGGGCCATCCCTTG-----------------ACTCACTTCTTA
AA--GGCCTT-----ACACAAAGTGAGAACTTGAACTTATC------TTAAAAGGGCCAAGG---CCCATTGCATCCTGG
GCCATCTCCAGTGATTCCTGATCTCTGTCTTGCCACTGGACCCCAGTGGCTCTGGAAGAGAAAGTAAAGCTGGTGACTTT
GCATAGCCCTTCCTTACTTAAATCCAATTCACTTGCATGTCGTGGCATCA------------------------------
----------------------------------------------------------------------CCATCCTCAT
CCAACGTGCTTTTTGGATTGGACGGCAGTTAATAAGCA-----------AGAGGGGATTTCCAGGGGGAAGAATGT--GG
GG-ACC--CTCAGCCCATGCTTGCTCT-GCTTCCAG--GCCTGTAAGTTTGCCCTGCGCATGTGTGGCCCTAATATGGAA
TGTGAAGAGCTAACAGCCTTGTTTGAGAAGCAGCTACATGATGGGCGTGGGCTA
>Tvu
AGATCATCAATGGGCTGGTGAGCCTGTTACTGCACCTGCAGGACCCCAAGCCTCCCGTTGTCAGT--GTGAGTAACAAAG
GCAAGGGGATGGGG--------------AGGGGAGCATAATGTCATCTTTTTGA----TAGGGAGGGACTAG--CACAAA
CTATCCATGGGGGCATT---ACTAGGCT----------------GGGGTTTACTGGCTACATTTCTTTCTCAAAGACCAT
GCCTTAAACCCAATTCACTCTGGAGCTCATTGATTGGACAACA-GTAGATCCCTGCCCAGGCACCACTTCATGTTTATTT
TTTGG-GCCCTCCTGGCTCAGAGTGAATATAAATAGTAACTGTCTCTCTTTTGGACAGCAACTCTGAGGGTCTTCCTCTC
CCAGTTTAATTTTTTTTT---TA---------TTAAAGGGGCCATCCCTTG-----------------ACTCACTTCTTA
AA-GAGGCCTCCCTCACTCAAAACGAGAACTTGAAAAGACCTTAGC-TTAAATGGGCCAAGATCTCCCACTGTTTCCTGG
```

```
GCCATCTCCAGTCG-TCCTCATCCACATCTGGCCACTGGACCCAGGTGGCTCTGGAAGAGAAAGTGAGGCTGGTGACTAT
GCACAGGCCTCCCTCACTTAAATCCAATTCACCTGCAAGTCACGTCATCATCTCCCTGATGTCATGGTCCTCTTCAAGAA
TGAAGGACAAACCACACACA---ACCAGGCT-----------AGGGAGGTGTGAGTTTCTAAGTGCC---TGTTTCCCTT
CCAGTGTGCTTTTTGGATTGGATGGCAGCTA-------TAGACTAAATGTAAGGGGATTTCCAGGGTGAAGAACAT--GG
TGAACCCTCTGAGCCCATGCTTGCTCT-GCTTCCAG--GCCTCTAAGTTTGCCCTGCGCATGTGTGGCCCTAATATGGAA
TGTGAAGAGCTAACATCCTTGTTTGAGAAGCAGCTGCATGATGGGCGTGGGCTA
>Mro
AGATCGTCAATGGGCTGGTGAGCCTGTTACTACACCTTCAGGACCCCAAGCCCCCAGTTGTCAGT--GTGAGTAACAAAG
GCAAGGGTACAGGG--------------AGAGGGGCATACAGTAATCTTTTTGA----TGGGAAGGGACTAG--TAC-AG
CTATCCATGGGGACATT---GCCAGGCT----------------GGGGTTTACTGGCTAGATTTCTTCCTCAAGGATTGT
ACCTTAAACCCAGTTCACTCTGGATTTCATTGAATGGACGA----TAGGTTCCTGCCCAAGGACC---AATTGGTTATTT
TTTGGGTCC-TCCTGGCTTAGGGTGAATGTAAATAGTAACCATTTCTCTTTTGGCCAGCAAACCTGAGGGTCTTCCCTTC
CCAGTCTGAATTTTTTTTA-------------TTAAATGGACTTCATTCACTTCTTAAAGAGGAG---GTCTGTTCACTG
AATGGATGTTGCCTCATTCAAAGTGAGAACTTGTAAAGACCTTAGC-TTAAAAGGGCCAAGGTCTCCCACTGCAACCTC-
-CCATCTCTAGTCA-TCCTGATCTATGTCTGGCCACTGGACCCAGACAGCCCTGGAAGAGAAAGTGAGGCTGGTGACTTT
GCCCAGCCCTCTCTCACTTAAATCCAATTCAGTTGCAAGTCATGACATCATCTCCCTGATGTCATGGTCCTCTTCAAGAA
GGAAGGACAAACCATATATA---GCCAGGCT-----------TGGGAGG---------------------TATCTCCCAT
CCAGCACGCCTTTTGGATCAGATGGCA-------GCTGTAGACTAAACGTGAGGGGGTTTCCAAGGTGAAGAACAT--GG
GGAACCCTCAGAGTCTATGCTCACTCT-GCTTCTAG--GCCTGTAAGTTTGCCCTGCGCATGTGTGGCCCTAATATGGAA
TGTGAAGAGCTAACAGCCTTGTTTGAGAAGCAGCTGCATGATGGGCGTGGGNTT
>Ptr
AGATTGTCAACGGGCTAGTGAGCCTGTTACTACACCTTCAGGACCCCAAGCCTCCAGTTGTCAGT--GTGAGTAACAAAG
ACAG--GTACAGGG--------------AGAGGGGCATACCATAATTTTTTTGA----TGGGAAGGGACTAG--TAC-AG
CTATCCATGGGGACATT---GCCAGGCT----------------GGGGTTTACTGGCTAGGTTTCTT---CAAGGATCAT
ACCTTAAACCCAGTTCACTCTGGATTTTATTGAATGTACTA----TAGGTTCCTGCCCAAGGACC---AGTTGGTCATTT
TTTGGGTCC-TCCTGGCTTAAGGTGAATGTAAATAGTAACTAT----CTTTTGGCCAGCAACCCTGAAGGTCTTCTCTTC
CCAGTTTGATTTCTTGTTA-------------TTAAATGGACTTCATTTACTTCTTAAAGAGGAG---GTCTGTTCACTG
AA------------CACTCAAAGTGAGAACC-ATAAAGACCTTAGC-TTAAAAGGGCCAAGGTCTCCCACTGCAACCTGG
GCCATCTCTAGTCA-TCCTGATCTGTGTCTAATCACTGGACCCAGACAGCCCTGGAAGAGAA------------------
-----GCCCTCTCTCACTTAAATCCAATTCAATTGCAAGTCATGATATCATCTCCCTGATGCCATGGTCCTCTTCAAGAA
GAAGTACAAAACAATATACA---GCCAGGCT-----------GGGGAGG---------------------TGTCTCCCAT
CCAGCACTCCTTTTGGATCAGATGGCA-------GCTGTAGACTAAACGTGAGGGGGTTTCCAAGGTGAAGAACAT--GG
GGAACCCTCTGAGCCTATGCTCGCTCT-GCTTCTAG--GCCTGTAAGTTTGCCCTGCGCATGTGTGGTCCTAATATGGAA
TGTGAAGAGCTAACAGCCTTGTTTGAGAAGCAGCTGCATGATGGGCGTGGGCTA
>Vur
AGATCATCAATGGGCTGGTGAGCCTGTTACTACACCTGCAGGACCTGAAGCCCCCGGTTGTCAGT--GTGAGTAACGAGG
GCAAGGCGGCAGGG--------------AGCGGGGCATAGGGTCATCTTTTTGC----TAGGGAGGGACTAG--TACCAA
CTATCCAT------------------------------------------------------------------------
--------------------------------------------------------------------------------
--------------------------------------------------------------------------------
--------------------------------------------------------------------------------
--------------------------------------------------------------------------------
--------------------------------------------------------------------------------
--------------------------------------------------------------------------------
---------------------------GGCC-----------AGGGAGGTGTGAGCTTCTAAGTGCC---TATTCCCCAT
CCAACATGCTTTTTGGATCAGATGGCA-------GCTATAGACTAAATGGGAGGGGATTTCCAGGGTGAAGAACAT--GG
GGAACCCTCTGAGCCCATGCTTGTTCT-ACTTCCAG--GCCTGTAAGTTTGCCCTGCGCATGTGTGGCCCTAATATGGGA
TGTGAAGAGCTAACAGCCTTATTTGGGAAGCAGCTGCATGATGGGCGTGGGCTA
```

MARKER 125

```
>MAR1a_Mdo
------------------------------------------------------------
------------------------------------------------------------
------------------------------------------------------------
--------------------------------------------TTGTTGTTGTTGTT--
--CA-GTCA----TTTTTCAGTC---GTGTCTGACTCTT---------------------
------------------------------------------------------------
------------------------------------------------------------
-----------------------------------------------------CGTGACC
CCATTTGGGGTTTTCTTGGCAAAGATACTGGAGTGGTTTGCCATTTCCTTCTCCAGCTCA
TTTTACAGATGAGGAAACTGAGGCAAACAGGGTTAAGTGACTTGCCCAGGGTCACACA-G
CTAGTAAGTGTCTGAGGCCAGA-TTTGAACTCAGGAAGAT--GAGTCTTCCTGACTCCAG
GCCCGGCGCTCTATCCACTGCGCCACCTAGCTGCCCC-----------------------
------------------------------------------------------------
------------------------------------------------------------
------------------------------------------------------------
------------------------------------------------------------
------------------------------------------------------------
----
>WSINE1
------------------------------------------------------------
------------------------------------------------------------
------------------------------------------------------------
------------------------------------------------------------
--------------------------------------------TTTTTTTTTTTTTT--
-------------GAGG-----CAATTGGGGTTAAGTGACTTGCCCAGGGTCACACAGCT
AGT--------AAGTGTCTGAGGCTGGATTTGAACTCAGGTCCTCCTGACTCCAGGGCTG
GTGCTCTATCCACTGCACCACCTAGCTGCCCCTC--------------------------
------------------------------------------------------------
------------------------------------------------------------
------------------------------------------------------------
------------------------------------------------------------
------------------------------------------------------------
------------------------------------------------------------
------------------------------------------------------------
------------------------------------------------------------
------------------------------------------------------------
----
>Mdo
ATGGTAATTCGGGAAATGACCACCTGGGAGATGCCAATGACTGCAGCTTT--CTAAGAGT
AGGGAGAATAGAGGAAAGGAAGAAG----CCATCAGAGACCCCAGATACCAGACCATCCC
ACACCCACGCAAGAA----TCAAG-AAACAGC-ATTATAGCTCTAACAGAGCCCATTCAA
AAGGCACAGGGAATAGCTCTGCAAAGAAGATCACAGCC-CA-------------------
------------------------------------------------------------
------------------------------------------------------------
------------------------------------------------------------
------------------------------------------------------------
------------------------------------------------------------
------------------------------------------------------------
------------------------------------------------------------
------------------------------------------------TTCTAACAAT-A
```

CCCAAAGGCCAGCTATA-CCTTCCATAA--GGCCCTACAGCATGGCCAAGCAT-GACAAC
AGTGAGAATTAAAGAGGAGAAGTAAGAAGT-AAAGAAAAAA--------------CATAA
GAAAAT--GAGTGTAAATTATTACCTAAATTCAGAGCTCAGAGTATGCCTAGTATTTGGA
AGGACTTCTGGCTTCCTCCCTTCCCTCCCCGACCTTCATTCCCAAA----CACACAGATG
CCTCCAGCTCAC--CCGTGAGTGACCCAACTCCCGGGAGTTCCTGTCAGTCACACTGATG
CCGT
>Dvi
ATGGTGATTCGGGAAATGACCACCTGGGAGATGCCAATGACTGCAGCTTT--CTAAGAGT
AGGGAGAATAGAGGGAAGGAAGAAG----CCATCAGAAACCCCAGATACCAGACCATCCC
ACACCCATGCAAGAA----TCAAG-AAACAGC-ATAATAGCTCTAACAGAGCCCATTCGA
AATGCACAAGGAATAGCTCTGCAAAGAAGACCACAGCC-CA-------------------

--TTCTAACACT-A
CCCAAAGGCCAGCTATA-CCTTCCATAA--GGTCCTAGGGCATGGCCAAATAT-GACAGC
AGTGAGAGCTAAATAAATGAAGTCAGAGGT-AAAGAAAAGA--------------CAGAA
GAAAACAAGAGTGTAAATTATGACCTAAATTCAGAACTTGGAGAATGCCTAATATTTGGA
AAGAATTCTGGCTTCCTCCCTTCCCTCCCCTACCTTCATTCCCAAA----CACACAGATG
CCTCCAGCTCAC--CCGTGAGTGACCCAACTCCCGGGAGTTCCTGTCAGTCACACTGATG
CCAT
>Mnu
ATGGTGATTCGGGAAATGACCACCTGGGAGATGCCAATGACTGCAGCTTT--CTAAGAGT
AGGGAGAATAGAGGGAAGGAAGAAG----CCATCAGAAACCCCAGATACCAGACCATCCC
ACATCCATGCAAGAA----TCAAG-AAACAGC-ATAATAGCTCTAACAGAGCCCATTCGA
AATGCACAAGGAATAGCTCTGCAAAGAAGACCACAGCC-CA-------------------

--TTCTAACACT-A
CCCAAAGGCCAGCTATA-CCTTCCATAA--GGTCCTAGGGCATGGCCAAATAT-GACAGC
AGTGAGAGCTAAAGAAATGAAGTCAGAGGT-AAAGAAAAGA--------------CAGAA
GAAAACAAGAGTGTAAATTATGACCTAAATTCAGAACTTGGAGAATGCCTAATATTTGGA
AAGAATTCTGGCTTCCTCCCTTCCCTCCCCTACCTTCATTCCCAAA----CACACAGATG
CCTCCAGCTCAC--CCGTGAGTGACCCAACTCCCGGGAGTTCCTGTCAGTCACACTGATG
CCGT
>Rra
ATGATGATTCGGGAAATGACGACCTGGGAGATCCCAATGACTGCAGCTTT--CTGAGAGC
AGGGAAGATAGAGGGAAGAAGGAAGGAA-TCATCAAAGACCCCAGATGCCAGCCCATCCC
ACACCCATTCATTAA-GAGTCAAGTACT-AGC-CCAATAACCCTGACAAAGCTAATTCCA
AAAATGCAGGGGAT---------AAAATTGCTAAAAAA-GC-------------------

--ACAGATAAATATAACACT-A
CTCCAAGGCCAGCTATA-TCTCCCATAA--GAACCTAT-GGATGT-CAAATAT-GAAAGC
AGAGAGAGTTAAAGAGGCTAAGTCATAAGCAAAAG--------------------CCGAA
GAAAACAAGGCTATAAGTTATTACCCAAATTCAGAGCTCAGTGCATGCCTAAGATGTAGA
AGGAATTCTGGC---CTCCC-----TCTCCTACCTTCGTACCCAAT----GACACAGATA
CCTCCAGCTCAC--CCGAGAGCAACCCAGTTCCTGGCAGTTTCGGTCAGTCACACTGATG
CCGT
>Cfu
ATGATGATTCGGGAAATGACGACCTGGGAGATGCCAATGACTGCAGCTTT--CTGAGAGC
AGGGAAGATAGAGGGAAGAAGGAAGGGA-CCATCAAAGACCCCAGATGCCAGCCCATTCC
ACACCCATTCATTAA-GAGTCAAGTACT-AGC-CCAATAACCCTGACAAT----------
-----------------------AAAATTGCTAAAAAA-CC-------------------

--ACAGATAAATATAACACC-A
CCCCAAGGCCAGCTATA-CCCCCCATAA--GAACCGAT-GGATGT-CAAATAT-AAAAGC
TGAGAGAGTTAAAGAGGCTAAGTCATAAGCAAAAG--------------------CCAAA
GAAAACAAGGCTATAAGTTATTACCCAAATTCAGAGCTCAGTGCATGCCTAAGATGTAGA
AGGAATTCTGGC---CTCCC-----TCTCCTACCTTCATCCCCAAT----GACACAGATA
CCTCCAGCTCAC--CCGAGAGCAACCCAGTTCCTGGCAGTTTCGGTCAGTCACACTGATG
CCGT
>Dgl
ATGGTGATTCGGGAAATGACCACCTGCGAGATGCCAATGACTGCAGCTTT--CTGAGATT
--GAAGAATAGAGGGAAGGAAGGAATGAACCATCAGAGACCCTAGATCCCAGCCCATTCC
ACATACATGCAGGGA----TCAAGC-ACCAGC-ACAATAGCCCTGACAGAGCCAATTTGA
AATG-------------CCTTGCTAGAAGACCACAGCTACA---TCTTTGTTGTTCTTCA
TTCAA-TCA---TTTTTTCAGTC---ATGTTCAATTCTT---------------------

---TGTGACC
CCATTTGGGATTTTCTTGGCAAAGACACTGGAGTGCTTTGCCATTTTCTTTTCCAGCTCA
TTTCATAGATGAGGAAACCGAAGCAAACAAGGTTAAGTGACTTGCCCAGGGTCACACA-A
CTAATAAGTGCCTG-GAGCTGGATTTGAACTTGAGAAGAT--GAGTCTTCCTGACTCCAG
GCCTAACATTCTATCTACTTCACCACCTAGCTGCCC----ACAGCCAAATCTAACACT-A
ACCAAAGCCCAGCTATA--GCCCCAGAAGAGACCCTAT-GGATGGCCAAGCAT-GAGAGC
AGAAAGAATTAAAGAGGCTAAATCATAAGC-AAAGAAAAAAACAGAAGAGAAGC--AGAA
GA-AAAGAGGGTGTAAATTATTACCCAAATTCAGGGTTCAGTGCATGCCTATGATGTGGA
AGGAATTCCAGCTACCTCCC-TCCTCG----ACCTTCCTCCCCAAA----CACAAAGATG
CCCCCAGCTCAC--CCGTGATCGACCCAACTCCTGAGAGTTCCTGTCAGTCACATTGATG
CCAT
>Pta
ATGGTAATTCGGGAAATGACCACCTGGGAGATGCCAATGGCTGCAGCTTT--CT-----A
AAAAAGAATAGAGGGAAGT--------AGTTGTCAGAGATCCTAGATCATTGCCCATTCC
ACATACACACATGGC----TCAAGA-ACCAGT-TCAGTAGCCCTGAGAT-----------
ATATATGTGTGGGATACTTTTACCAAAAGACCACCTCTACA---TATTTGTGC-TGGTTG

TTCAAGTCA------------------GATCCAATTCTT---------------------

---GGTGACC
CTGTTTGGGGTTTTATTGGTAAAGATATTTTAGTAATCTGCCATTTTCTTCTCCAGCTCA
CTTTACAGATGAAGAA-CTGAGGCAAACAACATTAAGCAACCTGCCCAAGATCACACA-G
CTAGTCAGTGTCTAAGACCAGACTTGAAACTCAGGAAGAT--GAGTCTTCCTGACTCCAC
ATTTGACATTCTATAT-------------ACTGCCC----CCAGCTAAATCTAATACT-A
CCCAAAGGCCAGCTATA-CCCCCCATAAAAGACCCTAT-CAATGGCCAAGCAT-GAGAGG
AGAGAG-TTTAAATGGGGTAAGTCATAAGC-AAAGAAAAAAACAGAAGAAAAGAAAAGCA
GA-AAATGGGGTATAAATTATTTCCCAAATTCAAGAATCAGCATATGCCCAGGACGTGGA
AGGAATTCCAACCTCTTCCC-TCCCTCC---CAATTCATTTCAAAC----CATACAGATG
CTTCCAGCTCAC--CCGTGATCGACCCAACTCTTGAGAATTCCTGTCAGTCACAGTGATG
CCAT
>Dgo
ATGGTAATTCGGGAAATGACCACCTGGGAGATGCCAATGGCTGCAGCTTT--CT----AA
AAAAAGAATAGAGGGGAGA--------AGTTGTCAGAGATCCTAGATCATTGCCCATTCC
ACATACACACATAGA----TCAAGT-ACCAGT-CCAATAGCCCTGA--------------
-----TATATGGGATAGTTTTTCTAAAAGATCACCTCTATG---TATTTGTGG-TGGTTG
TTCAAATCAATCTTTTTTCAGTC---ACATGAAACTCTT---------------------

---GGTGACC
CTGTTTGGGGTTTTATTGCCAAAGATATTTTAGTAGTTTGCCATTTTCTTCCCTAGCTCA
CTTTACAGATGAAGAA-TTGAGGCAAACAACATTAAGCAACCTGCCCAAGGTCACACA-G
CTAGTAAGTGTCTAAGACCAGACTTGAAACTTAGGAAGAT--GAGTCTTCCTGACTCCAC
AGCTGACATTCTATCT-------------ACTGCCC----ACAACTAAATCTAATACTA-
CCCAAAGGCCAGCTAT--CCCCCCATAAAAGACCTTAT-GGATGGCCAAGCAG-GAGAGG
AGAGAG-TTTAAATGGGTTAAGTCATAAGC-AAAGAAAAAAACAGAAGAAAAGAGAAGCA
GA-AAATGGGGTATAAATTATTTCCCAAATTCAAGAATCAGGATATGCCCAGGATGTGGA
AGGAATTCCAACCTCTTCCC-TCCCTCC---CAATTCATCTCAAAC----CATACAGATG
CTTCCAGCTCAC--CCGTGATCGACCCAACTCCTGAGAATTCCTGTCAGTCACAGTGATG
CCAT
>Scr
ATGGTAATTCGGGAAATGATCACCTGGGAGATGCCAATGGCTGCAGCTTT--CT-----A
AAAAAGAATAGAGGGGAGG--------AGTTGTCAGAGATCCTGGATCATTGCCCATTCC
ACATACACACATGGA----TCATAC-----------------------------------
--------AGGGGATAGTTTTACTAAAAGACCACCTCTACA---TATTTGTGG-TGGTTG
TTCAAGTCAACC-TTTTTCAGTC---ACATACAACTCCT---------------------

---GGTGACC
CTGTTTGGGGTTTCATTGGCAAAGATATTTTAGAAGCTTGCCATTTTCTTC------TCA
TTTTACAGATGAAGAA-CTGAGGCAAACAACATTAAGCAACCTGCCCAAGCAACCTTGGG
CTAGTAAGTGTCTCAGACCAGACTTAAAACTCAGGAAGAT--GAATCTTCCTGACTCCAC
ATCTAACATTCTATCT-------------ACTGCCC----ACAGCTAAATCTAATATTA-
CCCAATGGCCAGCTATA--CCCCCATAAAAGACCCTAT-GGATGGTAAAACTCTGAAAGG
AGATAG-TTTAAATGGGTTAAGTCATAAGC-AAAGAAAAAAACAGAAGAAAAGAAAAGCA
GC-AAATGGGGTATAAATTATTTCCCAAATTCAAGAATCAGCACATGCCCAGGATGTGGA
AGGAATTCCAACCTCTTCCC-TCCCTCC---CAATTCATCTCAAAC----CATACAGATG
CTTCCAGCTCAC--CTGTGATCGACCCAACTCCTGAGAATTCCTGTCAGTCACAGTGATG
CCAT
>Mfa

```
ATGGTAATTCGGGATATGACCACCTGGGAGATGCCAATGGCTGCAGCTTT--CT-----G
AAAAAGAAAAAAGGGGAGG--------AGTTGTCAGAGATCCTAGATCATTGCCCATTCC
ACATACACATATGGA----TCAAG--ACCAAC-CCAATAGCCCTGAC-------------
-ATA-TATAGGGGATAGTTTTACTAAAAGACCACCTCCAC----ATTTTGTGG-TGGTGG
TTCAAGTCAATCATTTTTCAGTC---ATATCCAACTCTT---------------------
------------------------------------------------------------
------------------------------------------------------------
-----------------------------------------------------GGTCACC
CTGTTTGGGGTCTTATTGGCAAAGATATTTTAGTAGTTTGCCATTTTCTTCTCTAGCTCA
TTTTACAGATGAAGAA-TTGAGGCAAACAAGATTAAGTGACCTGCCTAAGGTCACACA-G
CTGGTAAGTGTCTAAGGCCAGACTTGAAACTCAGGAAGAT--GAGTCTTCCTGACTCCAC
ATCTGACATTCTATCT-------------ACTTCCC----ACAACTAAATCTAACACTA-
CCCAAAGGCCAACTATAC-ACCCCATAAAAGGCCCTAG-GGATGACCACCCAT-GAGAGG
AGAGAG-TTTAAACGGGTTAAGTCATAAGC-AAAGAAAAAAACAGAAGAAAAGAGAAGCA
GG-AAATAGGATATAAATTATTTCCCAAATTCAAGAATCAGCACATGCCCAAGATGTGGA
AAGAATTCCAACTTCTTCCC-TTCCTCC---CAATTCATCTCAAAC----CATACAGATG
CTTCCAGCTCAC--CCGTGATCGACCCAACTCCCGAGAATTCCTGTCAGTCACAGTGATG
CCAT
>Bil
ATGATGATTCGGGAAATGATCACCTGGCAGATGCCAATGACTGCAGCTTT--CTGAGAGC
AGGAAGAATAACGGGAAAGAA--------CCATCAGAGACCCTAGATTCCAACCTATTCC
ACATGCATGCAGTGA----GCAGGC-ACCAGC-ACAATAGCCCTTTA--GACCAATTCAA
AATATGCA-GGAGAAAG-TTTACCAGAAGACCACAGCTACA---TCTTTATCGTT-----
--CAAGTTAGTTGTTTTTCAGTC---AAATCGGACTCTT---------------------
------------------------------------------------------------
------------------------------------------------------------
-----------------------------------------------------GGTGATT
CCACTTAGAA-TTTCTTCACAAAGTAATGGGAGTACTTTGCCATATTCTCCTCTAGCTTG
ATTTACAGATGAGGAAACTGAGGCAAAC-AGGTTAAGTGATTTGCCCAGGGC--------
-----------------------------------------------TTCTTGACTCCAG
GGC-AAACTACTACATCTACCAACACCTACCTGCCC----ACAGCTAAATCTAATACT-A
CCCAAAGGCTAGCTATTCACNCCCATAAGAGACCCTAT-GGATAGCCAAGCAT-GAGAGC
AAAGTGTATTAGAGAGGCTAACTCATAAGC-AAAGAAAAAA-------------GCAGAA
GGAAA-GGAGGTATAAATTATTACCCAAATTCAGGGATCAGGGCA---------CATGGA
AGGCATTTTGGCTTCCTCCC-CCACC-----ACCTTCATCCCCAAACACACAGACAGATG
CCTCCAACTCAC--CCGTGATTGACCCAACTCCTGACAGTTCTTGTCAGTCACACTGATG
CCAT
>Pgu
ATGATGATTCGGGAAATGATCACCTGGCAGATGCCAATGACTGCAGCTTT--CTGAGAAT
AGGAAGAATAAAAGTTAAGAA--------CCATCAGAGATCCTAGATTCTAGCCCATTCC
ACATGCATACAGGCAGGGAGCAAGCAACCAGC-ACAATAGTCCTGATA-AACCAATTCAA
AATATGTA-GGAGATAGCTTTACCAGAAGACCACAGCTACA---TCTTTATTGTT-----
--CAACTTAGTTGTTTTTCAGTC---AGGTCTGACTCTT---------------------
------------------------------------------------------------
------------------------------------------------------------
-----------------------------------------------------GGTGATT
CCACTTGGAA-TTTCTTCACAAAGTAATGGGAGTGCTTTGTCATATTCTCCTCTAGCTTA
TTTTACAGATGAGGAAACTGAGGCAAAC-AGGTTAAGTGATTTGCCCAGGGCTCTTAA-G
AT----------------------------------------GAGTCTTCTTGACTCCA-
------------------------------------------------------------
----AAGGCTAGCTATTTGCCCCCATAAAAGACCCTAT-GGATAGCCAAGCAT-GGGAGC
AAAGAGTGTTAGAGAGGCTAACTCATAAAC-AAAGAAAAA-----------------GAA
GGAAAAAAGGGTATAAATTATAATCCAAATTCAGGGATCAGGGTA---------TGTGGA
```

AGGACTTTTGGCTTCCTCCC-TCCCCCCC--ACCTTCATCCCCAAA----CACATAGATG
CCTCCAACTCAC--CCGTGACCGCCCCAACTCCTGACAGTTCTTGTCAGTCACACTGATG
CCAT
>Iso
ATGATGATTCGGGAAATGATCACCTGGCAGATGCCAACGACTGCAGCTTT--CTGAGAAC
AGGAAGAATAAAGGTTAAAAA--------CCATCAGAGATCCTAGATTCTAGCCCATTCC
ACATGCATACAGGGA----GCAAGCAACCAGC-ACAATAGCCCTGATA-AACCAATTCAA
AATATGTA---GGAGAGCTTTACCAGAAGACCGCAGCTAAA---TCTTTATTGTT-----
--CAACTTAGTTGTTTTTCAGTC---AGGTCTGACTCTT---------------------

---GGTGATT
CCACTTGGAA-TTTCTTCACAAAGTAATGGGAGTGCTTTGCCATATTCTCCTCTAGCTTG
TTTTACAGATGAGGAAACTGAGGCAAAC-AGGTTAAGTGATTTGCCCAGGGCTCTTAA-G
AT--GAGTCTTCTTGACTCCA-

----AAGGCTAGCTATTTGCCCCCATAAAAGACCCTAT-GGATAGCCAAGCAT-GGGAGC
AAAGAGTGTTAGAGAGGCTAACTCATGAAC-AAAGAAAAAAATCA------------GAA
AGAAAAAAGGGTATAAATTATAACCCAAATCCAGGGATCAGGGCA---------TGTGGA
AGGACTTTTGGCTTCCTCCC-TCCCCCC---ACCTTCATCCCCAAA----CACATAGATG
CCTCCAACTCAC--CCGTGACCGACCCAACTCCTGACAGTTCTTGTCAGTCACACTGATG
CCAT
>Tro
AAGACGATTCTGGAAATGACCACCTGGGATATGCCAGTGACTGCAGCTTT--CTGAGAAC
AGGAAGAAGAGAGAAAAGG--------AACCATCAGAGAGCTTAGATCCCAGTCCATTCC
ACATACATGCAGGGA----GCAAGC-ACCAGCCCAAACTGCCCTGACAGAACCAATCTGA
AATG-TGCAGAGGACCGTTTTA-GAGAAGACCAAAGCTACA---TCTCTGTTGTTGGTCA
GTCACA---------GCTTAATC---ACGTCCAACTCTC---------------------

---GGTGACT
CCAGCTGGTATTTGTTAGAAAAAGATACTGGAAAGGTTTGCCTTTTCCTTCTCCATCTCA
TTTTACAGAAGAAGAAACTGAGGCAAACAGGATGAAGTGACTTGCCCAGGGTCATACA-G
CTAGCAAGTGTCCAAGGCCAGATTTG-AACTCAGGAAAAT--GACTCCTCTTGAG-TCAG
GCCTGACATTCCATCTACTGTACCACCTAGCTGCCC----ACAGCTAAATCTAATACTA-
CCCAAAGGCCAGCTATAT-CCCCCATAAAAGGTCCTAT-TGATGGCCAAGCAC-GAAAGC
AGAAAGAATTAAAGAGAATAAATCATAAGC-AAAGGAAAAAGT--------------CGA
AGAAAA---GGTATAAATTCTTACCCAAATTCAGGGATCAACACCTGCCTAAAATGAGAA
AGGAATTCTGGCTTCTCCCC-TCCCCC----AC-TTCATTCCC-AA----CACACAAGTG
CCTCCAGCTCAC--CCGTGATCGGCCCAACTCCTGAGAGTTCCTGTCAGTCACACTGATG
CCAT
>Ppe
ATGGTGATTCGGGAAATGACCACCTGGGAGATGCCAATGACTGCAGCTTT--CTGAGACC
AGGAAGAATAGAGAGAAGG--------AACCATTAGAGT--------------CCATTCC
ACACACATGCAGGGA----TCAAGC-ACCAGC-ACAATAGCCCTGAC-----AGATCCAA
TCTGAAACT-TGCAGAGGATAGCTTTACTAGAAGACCACAG-TTACATCTTTGTTGCTGT
T-CAG-TCAGGCATATTTTAATC---ATATCCAACTCTT-----TTTTTGTTGTTGTTGC
TTTTTGTGGGGGGGAGGTAGGGCAATTGGGGTTAAGTGATTTGCCCAAGGTTACACAGCT
AGTTTTGTGTTAAGTGTCTGAGGCTGCATTTGAACTCAGGTCCTCCTGACTCCAGGGCTG
GTGCTCTATTCACTGCACCACCTAGCTGCCCCC----ATGTCCAACTCTT---GATGAGT
CTATTTGGGATTTTCTTGGCAAATGTATTGGAGTGGTTTGCCATTTCCTTCTCTAGCTCA
TTTTACAAAAGAGGAAACTGAGGCAAACAGGGTGAAGTGACTTG-------TCACACA-G
CTAGCAAGCATCCAAGGTCAGATCTG-AACTCAGGGAAAC--GAATTTTCTTGAGTTCAG

GCCTGACATTCTATCTACTGCACCTCCTACCTGACC----ACAGCTAAATCTAACACT--
CCCAAAAAACAGCTATACCCCTCCATAAAAGGTCCTAT-GGATGGCCAAGCAT-GAGAGC
AGAAAGAATTAAAGAGGATAAGTCATAAGC-AAAGAAAAAAGT--------------CAA
AGACAAGAGGGTATAAATTATTAACCAAATTTAGGGATCAGCACATGCCTAGAATGTGAA
AGGAAGTCTGGCTTCTCCCC-TCTCCC----ACCTTCATCCCC-AA----CACACAAATG
CCTCCAGCTCAC--CCGTGATCGACCCAACTCCTGAGAGTTCCTGTCAGTCACACTGATG
CCAT
>Mro
ATGGTGATTCGGGAAATGACCACCTGGGAGATGCCGATGACTGCAGCTTT--CTGAGAGC
AGGAAGAATGGAGAGGA-----------CCCATCAGAGAACCTAGATCCCAGCCCATTCC
ACGCACATGCAGGGG----TCAGAC-ACCAGC-ACAA------TGACACA----------
-----TGCAGAGGACAGCTCTCCTGGAAGGCCACAGCCACA---TCTTTGCTGTTGGTGT
TGCTGCTTAGTCGTTTTTCAGTC---GTGTCCATCTCTT---------------------

---GGTGACT
CCATTTGAGGTTTTCTTGGCGAAGATACTAAAGGGCTTTGCCT-TTTCCTCTCCAGTTCA
TTTTACAGAAAAGGAAACTGAGGCAAACAGGGTGCAGTAACTTGCCCAGGGTCACCCA-G
CTAGTAAGTGTCTGAAGCCATATTTA-AACTCAGGAAAAT--GAGTTGTCTTGAGGCCAG
GCCTGACATTCCATCTACTGTACCACCTAACTGCCC----TTAGTTAAATCTAACTATGG
CCCAAAGGCCAGCTATAC-TCCCCATTAAAGGCCCTAT-GGATGACCAAGCAT-GAGAGC
AGAAAGAATTAAAGAGGGTAAATCATAAGC-AAAGAAAATAGC--------------AGA
AGA-----GAGTATAAGTTATCACCCCAATTCAAGGATCAGCACTTGCCTAAAATATGAA
AAGAATTCTGACTTCCTCCC-TCCCCC----CTCTTCATCCCC-AA----CACACAGATG
CCTCCAGCTCAC--CCGTGATCGACCCAGCTCCTGAGAATTCCCGTCAGTCACGCTGATG
CCAT
>Ptr
ATGGTGATTCGGGAAATGACCACCTGGGAGATGCCGATGACTGCAGCTTT--CTGAGAGA
AGGAAGAGTGGAGAGAAGG--------ATCCATCAGAGATCCTAGATCCCAGCCCACTCC
ACACACATGCAGGGA----TCAGAC-ACCAGC-ACAA------TGACATA----------
-----TGCA-GGGACAGCTTTA-TGGAAGGCCACAG--------CCTTCCTTGCTGCTG-
--------------------GTC---ATGTCCATCTCTT---------------------

---GGTGACT
CCATTTGAGGTTTTCTTGGTGAAGATACTAACGTGCTTTGCCA-TTTCCTCTCCAGTTCA
TTTTACAGGAAAGGAAACTGAGGCAAACAGGATGCAGTAACTTGCCCAGGGTCACCCA-G
CTAATAAGTGTCTGAAGCCATATTTA-AACTCAGGAAAAT--GAGATGTCTTGAGTCCAG
GCCTGACATTCCATCTACTGTACCACCTAACTGCCC----TTAGTTAAATCTAACTGTGG
CCCAAAGGCCAGCTATAC-CCCCCATTAAAGGCCCTAT-GGATGACCAAGCAT-GAGAGC
AGAAAGAATTAAAGAGAGTAAATCATAAGC--------------------------CCGA
AGA-----GAGTATAAATTATTACCCCAATTCAAGGATCAGCACATGCCTAGAATATGAA
AAGAATTCTGGCTTCCTCCC-TCCCC-----ACCTTCATCCCC-AA----CACATAGATG
CCTCCAGCTCAC--CCGTGATCGACCCAGCTCCTGAGAATTCCCATCAGTCACACTGATG
CCAT
>Vur
ATGGTGATTCGGGAAATGACCACCTGGGAGATGCCAATGACTGCAGCTTT--CTGAGAGC
AGGGAGAAGAGAGGGGAG---------AACCATCAGAGACCNTAGATCCCAGCCCACTCC
ACACATGTGAAGGGA----TCAAGC-ACCAGC-ACAACAGCCCTGAGAGAGCCAATCTGA
AATG-TGCAGGGCATAGCTTTACTAGAAGACCACAGCTACA---TCTTCA---TTGGTGT
--TCTGTCA-----------GTC---ACGTCCAACTGTT---------------------

--GGTGACT
GCATGTGGGGTTTTCTTGGCAAAGATACAGAAGTGGTTTGCCA---TCTTCTCCAGCTCA
TTTTACAGATGAGGAAACTGAAGCAAACAGGGTGAAGTGACTTACCCAGGGCCACACA-G
CTATTAAGTATCTGAGGCCAGATTTG-AACTCAGGAAAAT--CAGTCTTCTTGAGTCCAG
GCCTGACATTTTATCTACTGTACCACCTAGCTGCCC----ACAGCTAAATCTAACTCTA-
CCCAAAGGCCAGCCATAA-CCCGCATAAAAGGCCCTAT-GGATGGCCAAGCAT-GAAAGC
AGAATGACTTAAAGAGGCTAAGTCATAAGC-AAAGAAAAAA--------------GCAGA
AGAAAAAAGGGTATAAATTA-CAC----ATTCAGTGATCAGCACATGCCTAGAATGTGGA
AGGAATTCTGGCTTCCTCCA-TCCCCC------CTTCATCCCCAAA----CACACAGATG
CCTCCAGCTTAC--CCGTGATCGACCCAGCTCCTGAGAGTTCCTGTCAGTCACGCTGATG
CCAT

MARKER 126

```
>Mar1b_Mdo
------------------------------------------------------------
------------------------------------------------------------
------------------------------------------------------------
-----------------------------------------------------------A
TGGCAAACCA------------CTCCAGT-------------------------------
------------------------------------------------------------
------------------------------------------------------------
------------------------------ATCTTTGCCAAGAAAACCCCAAATGGGGTC
ACGAAGAGTCAGACATGACTGAAAACGACTGAACAACAACAACAA---------------
------------------------------------------------------------
------------------------------------------------------------
------------------------------------------------------------
---------------------------------------------------
>WSINE1
------------------------------------------------------------
------------------------------------------------------------
------------------------------------------------------------
------------------------------------------------------------
---------------------------------------------TTTTTTTTTTTTTTG
AGGCAATTGGGGTTAAGTGACTTGCCCAGGGTCACACAGCTAGT-------AAGTGTCTG
AGGCTGGATTTGAACTCAGGTCCTCCTGACTCCAGGGCTGGTGCTCTATCCACTGCACCA
CCTAGCTGCCCCTC----------------------------------------------
------------------------------------------------------------
------------------------------------------------------------
------------------------------------------------------------
------------------------------------------------------------
---------------------------------------------------
>Mdo
TGACCTCCAGCTGAACTGCTGCAGCCACTCACAGGTCATGCCTCCTGCCAAGGTGCCCAG
GTGC--CTGAGGGGG-------AAAAAAAACTTGGGAGAAGCAGTTAAGCCAGACAACA-
--GCTTCACT------------------------CTCCAACTTGACACCTATATTGCTGT
CAAAAT------------------------------------------------------
------------------------------------------------------------
------------------------------------------------------------
------------------------------------------------------------
------------------------------------------------------------
---------------------------------------------------TATGTGTGT
GTATGATTGT-AGGGG-AAGTCA--TCTATC-AGTCT-TG--GATGATGGCTACAGGCCC
TAGAAACACCTTGATTAGACAGGGCA-CCAGAGG-TTCCCAAGCCTACAC-AAATGTCTC
TTCAGCATTTCAG-GGACCAGGGTGAAAATGTCCTTCATTGTACAAGGAGAACAAAAGGA
AGAAGGCTGCCTCAATAGGTGCAGAGGGAACACAAGAGAAACTTCCTGCTT
>Cfu
TGTCCTCCAGCTGAACTGCTGCAGCCACTCACAAGTCATGCCTCCTGCCAGGGTACCTAG
AGAC--CTGAGAAG----------AAAAAACTGGGGAGAAGCAGTTAAGCCAAACAACA-
--TCCCCCCCCACCCCA-----------------CTCCAACTTGACACCTATAATGCTAT
CAAAAT---CATCT----------------------------------------------
------------------------------------------------------------
------------------------------------------------------------
------------------------------------------------------------
------------------------------------------------------------
```

--GTGTATATGTAT
GTTTGCTTGT-AGAGTGAGGTCA--TCTATC-----------AGTCTTGGATAAT-----
----------TTTTATGAGATTATATACAAGAGG-TCCCCAAGCCTACAT--AATGTCTC
T--AGCACTTCAG-GGGCTAAGGTAGAAATGCCA-TTCTTGTACAAGGAGAACAAAAGAA
GGAAGA-TGCCTCAACAGGAGCAAAGGAACAGAAGAGAAATTTCCCATTT
>Dgl
AGCCCTCCAGCTGAACTGCTGCAGCCATTCACAGGTCATGCCTCCTGCCAGGGTGCCCAA
AGAC--CTGAGGG--------GAAAAAAAATTTGGGAGAAGCAATTAAGCCAGATAACA-
--GCCCACCCCCCTA-----------------CCCCCCAGCTTGATACCTGTAATGCTGT
CAAAAT---TGTGCATCACACAC--------------------ATATCTATATCTATATC
TATCTATCTATC-------TATGTCCAGT---ATTTTTTGGTTGTTTTTTGTTTTTTGTG
GGGCAATGGGGGTTAAGTGACTTGCCCAGGGTCACACAGCTAGTAAGTGTCAAGTGTCTG
AGGCTGGATTTGAACTCAGGTACTCCTGAATCCAGGGCTGGTGCTTTATCCACTGCGCCA
CCTAGCTGCCCCC----TATGTCCAGT---GTTTTTGCCAAGAAAAGACCAAATAGGGTT
GTGAAGAGTTGGAGATGACTA-AAACAACTGAAC------AATAT---AAATGTATGTGT
GTATTTTTGT-AGGGAGCTGTCA--TCTATC-AACCTTAAGTGGTCTTGTCTAGAAACCC
TAGAAACCTC-TTGACCAGGCCAGACATGAGAGA-TCCCCAAGCCTCTACAAAATGTCTC
TTTAGCACTGCAG-GGTCCAGGGTGGA---------------------AGAACAAACGGA
GGAAGGCTGCCTCAACGGGGGCAGAGGGAACAGAAGAGGCGCTTCTTGTTT
>Nty
TGTCCTCCAGCTGAACTGCTGCAACCACTCACAAGTCATGCCTCCTGCCAGGGTGCCCAG
GGAC--CTGAAGG-----------AAAAAACTTGGGAGAAGCAATTAAGCCAGACAACA-
--ACCTATGACGTTCCCCAAA--------------CCCATATTGACACCTATAATGCTGT
GAATAT---TGTATGTATGTTTA-------------------------------------
-----AATTAT--------GAT---TAGT-------------------------------

------------------------------ATCTTTGCCAAGAAAATCTCAATTAGGATC
AGGAAGAGTTGGGCAGGACTG-AAGCAATTCAAC------AAG-----AACAGAAAG---
GTAT--------GGGAGAGGTCA--TCTATC-AGTCTTAGATGATCTTGGCTAGAGTCCC
TA-AACCATC-TTGATAAGATGAGGCATGAAAGA--------GTCTATAT-AAATGTCTC
TTTAGCACTGCAG-AGGTCAGGG--AAATGGTCATTTATTG-TAC----------AAGAA
GGAAGGCTTCTTCCACAGTGGCAGAGGAAATGGAAGAGAAACCTTCTGCTT
>Pta
TGTCCTCCAGCTAAACTGCTGCAGCCACTCACAGGTCATGCCTCCTGCTAGAGTGCCCAG
AGAC--CTGAAGAAAAAAAAAAAAAAAAAACTTGAGAGGAGCAATTAAACCTGACAACC-
--TGCCACCAGCCCACT----------------CCCCCAAGCTTGAACCTAAAATGCTGT
CAAAAT---TGTGTGTGTGTGTG---------TGTGTGTGTGTGTGTGTGTGTGTGTGTG
TGTGTAAATTG--------TATATCCAGT-------------------------------

------------------------------ATCTTTGCCAAGAGAACCCTAAATAGGGTC
ATGAAGAGTTGGACGTGATTA-AAACAGCTCGAC------AACAG---AAATGTATGTAT
ATATGTTTGT-AGGGAGGGTTCA--TCTATC-AGTCTTAAATGATCGTGGCTTGAG-CCC
TA----------------GACCAGACAT--GAGA-TCCTCAAGCCTACAC-AAATGTCTC
TTCAGCACTGCAG-GGGCCAGGGTGGAGATATCATTTATTG-CACAGGAGAATAAAAGGA
GGAAGGCTGCCTCTGTGAGGGCAGAGGGAACAGAAAAGAGACTTCCAGCTT
>Dgo
TGTCCTCCAGCTGAACTGCTGCAGCCACTCGCAGGTCATGCCTCCTGCCAGAGTGCCCAG
AGAC--CTGAAGGGA------AAAAACAAACTTGAGAGGAGCAATTAAGCCCGACAGCC-
--TGCCACCTGCCCACT----------------CCCCCAAGCTTGAATCTAGAATGCTGT
CAAAAT---TGTGTGTGTGTGTG-----------------------------------TG
TGTATAAATTG--------TATGTACAGT-------------------------------

------------------------------ATCTTTGCCAAGAGAATCCTAAATAGGGTC
ATGAAGAGTTGGACAGGATTA-AAACAACTTGAC------AACAT---AAATGGATGTAT
TTATGTTTGT-AGGGAGGGTTTA--TCTATC-AGTCTTAAATGATCTTGGCTAGAG-CCC
TA----------------GACCAGACAT--GAGA-TCCTCAAGCCTATAC-AAATGTCTC
TTCAGCACAGCAG-GGGCCAGGGTGGGAATAGCATTTATTG-CACAGGAGAATAAAAGGA
GGCAGGCTGCCTCTGTGAGGGTAGAGGGAAC-----AGAAACTTCCTGCTT
>Scr
TGTCCTCCAGCTGAACTGCTGCAGCCACTCACAGGTCATGCCTCCTGCCAGAGTGCCCAG
AGAC--CTGAAGGAA-----AAAAAAACAACTTGAGAGGATCAATTAAGCGTGCCA-CC-
--TGCCCACTC----------------------CCTCAAGCTTGCCACCTAGAATGCTGT
CAAAAT---NGTGTGTGTGTGTG-------TGTGTGTGTGTGTGTGTGTGTGTGTGTGTG
TGTGTAAATTG--------TATGTCCAGT-------------------------------

------------------------------ATCTTTGCCAAGAGAACCCTAAACAGGGTC
ATGAAGAGTTGGATGTGATTA-AAACAACTCAAC------AACAT---AAATGTATGTAT
ATATGTTTGT-AGGGAGGGTTCATCCCTATC-AGTCTTAAATGATCTTGGCTTGAGC---
-----------------------------------TCCTCAAGCCTACAT-AAATGTCTC
TTTAGCACTGCAG-GGGCCAGGGTAGAAATGTCATTTATTG-CACAGGAGAATA---GAA
GAAAGGCTACCTCTCTGAGGCCAGAGGGAATAGAAAAGAAACTTCCTGCTT
>Pgu
TGTCCTCCAGCTGAACTGCTGCAACCATTCACAGGTCATGCCTCCTGCCAAGGTGCCGAG
AGAC--CTGGGGG----------AAAAAAACTTGGGAGAAGCAATTAAGCCAGACAGCAA
CTTGTCAACTTCTCCCACA--------------CTTCCAGGTTGACACCTGTAATGCTGT
CAAAAC---TGTATGTGTGATTA-------------------------------------
-------AATG--------TCTGTCTAGT-------------------------------

------------------------------ATTTTTGCCAAGAAAACCCCAA--------
-CAAAGAGCTGGATATGACTG-ACACAACTTCAC------AACA----ATATAAATGTTT
GTATGTTTGT-AGGGAGCAGTCA--TTTATC-AGTCTTAGATGACTTTGGCTAGAATACG
TAGAGATATCTT-GACTAGACCAGATATGAGAGATTCCCCAAGCCTATAT-AAATGTTTC
TTTAGCACTGCAG-GGGCCAGGGTGGAAATGTCATTTATTATATAAAGGGAACAAAAGGA
GGAAGATTGCCTCAACAGAGGCAGAAGGAACAGAAGAGAAACTTCCTGCTT
>Iso
TGTCCTCCAACTGAACTGCTGCAACCATTCACAGGTCATGCCTCCTGCCAAGGTGCCGAG
AGAC--CTGGGGG----------AAAAAAACTTGGGAGAAGCAATTAAGCCAGACAGCAA
CTTGTCAACTTCTCCCACA--------------CTCCCAGATTGACACCTGTAATGCTGT
CAAAAT---TGTATGTGTGATTA-------------------------------------
-------AATG--------TCTGTCTAGT-------------------------------

------------------------------ATTTTTGCCAAGAAAACCCCAA--------
-CAAAGAGCTGGATATGACTG-ACACAACTTCAC------AACA----ATATAAATGTTT
GTATGTTTGT-AGGGAGCGGTCA--CTTATC-AGTCTTAGATGACTTTGGCTAAAATA--
TAGAGATATGTT-GACTAGACCAGATATGAGAGATTCCCCAAGCCTATAT-AAATGTTTC
TTTAGCACTGCAG-GGGCCAGGGTGGAAATGTCATTTATTATATAAAGGGAACAAAAGGA
GGAAGATTGCCTCAACAGAGGCAGAAGGAACAGAAGAGAAACTTCCTGCTT
>Tro
TGTCTGCCAACTGAACTGCTGCAGCCATTCACAGGTCATGCCTCCTGCCAGGGTGCCCAG
AGAC--CTGGGGGG----------AAAATACTTGGGAGAAGCAATTAAGCCAGACAACAG

```
TTTTCCACCTGCCTCCCCTG-------------CCCCCAATTTGACACCTATAATGCTAT
CAAAAT---TGTGTGTGTGTCTGTATGTATGTGTGTGTGTGTGTGTGTGTGTGTGTGTGT
ATGTGAGTATGTATAA---TATGTCCA---------------------------------
------------------------------------------------------------
------------------------------------------------------------
------------------------------AAATCTGCCAAGAAAATCCCAAATGGGGTC
ATGAAGAGCTGGATCTGACTGAAAATAATTCAAC----ACAACAT---AAATATATGCAT
ATATGTTTGT-AAGTAGAGGTCA--TCTATC-ANTCTTAGATGATCTTGGCTAGAGTCCC
TAAAGACATCTTTGACTAGACCAGACATGAGAGA-TCCCT-------------ATCTACC
TCTAGCCCGGCAA-GGGTCAGAGTG------------------------GAACAAAAGGA
GG-------CTTCCCCAGGGGCAGAGGGAACAGAAGAGAAGCTTGCTGCTT
>Ppe
TGTCTGCCAACTGAACTGCTGCAGCCACTCACAGGTCATGCCTCCTGCCAGGGTGCCCAG
AAAC--CTGAGGGG----------AAAAAACTTGGGAGAAGCAATTAAGCCAGACAACAG
CTTGCCACCTGCTCCCTA---------------CCCCCAGCTTGAAACCTATAATGCTGT
CAAAAT---TGTGTGTGTGTGTG--------------------------TGTGTGTGTGT
ATGTGTATA-G--------TATGTCCAGT-------------------------------
------------------------------------------------------------
------------------------------------------------------------
------------------------------ATCTTTGCCAAGAAAATCCCAGATGGGGTC
ATGAAGAGTTGGATATGACTG-AAACAACTCAAC---AATGACAT---AAATATATGTAT
ATATGTTTGT-AAGGAGAGGTCA--TCTATCAAGTCTTAGATGATCTTGGCTTGAGTCCC
TAGAGATATCTT-GACTAGACCAGACATGAGAGA-TCCCT-------------ATCTCTC
TTTAGCACTTTGA-GGGTCAGGGTG------------------------GAACAAAAGGA
GGAAGGCTGCTTCAACAAGGGCAGAGGGAACAGAAGAGAAACTTCCTGCTT
>Mro
TGTTTGCCAGCTGAACTGCTGCAGCCACTCACAGGTCATGCCTCCTGCCAGGGTGCCCAG
AGAC--CTGAGGG-----------AAAAAACTTGGGAGAAGCAATTAAAACAGACAACAG
CCTGTCACCTGATCCCTCCCA--------CCCACCCCCAGCTTGACACCTATAATGCTGT
CAAAAT---TGTGTGTGTGTGTG------------------------TGCGCGCGCATAT
GTGTATAAATG--------TACATCCACT-------------------------------
------------------------------------------------------------
------------------------------------------------------------
------------------------------ATCTTTGCCAAGAAAATTCCAAATGAGGTC
ATGAAGG-------ACGACTG-AAAAAACTCAAC------AACAT---AAATGTGTAGAT
ATATGTTTGT-AAGGAGAAGTCA--TCTGTC-AGTCTTAGATGATCGTGGCTAGAGTCCC
TAGAGACATCTT-GAGTAGACCAGACATGAGAGA-TCCCT--ACC-----------TCTC
TTTAGCACTGCAA-GGGTCAGGGTG------------------------GAATAAAAGGA
GGAATGCTGCCTCAACAGGGGCAGAGGGAACACAAGAGAAACTTCCTGTTT
>Vur
TGTCCTCCAGCTGAACTGTTGCAGCCACTCACAGGTCATGCCTCCTGCCAGGGTGCCCAA
AGAC--CTAAGGG----------AAAAAAACTTGGGAGAGGCAATTAAGCCAGACAACAG
CCTGCCACCTACCCCCCCACACACATACCCCAACCCCAAGATTGACACCTATAATGCCGT
CAAAAT---TGTGTGTGTATGTG-------------------------------------
-TGTATAAATG--------TATGTCCAGT-------------------------------
------------------------------------------------------------
------------------------------------------------------------
------------------------------ATCTTCGCCAAGAAAACCCCAAATGGGGTC
ATGAAGGGGTGGATATGACTG-AAACAACTCAAC---AACAACAT---AAATGTATGTAT
ATATGTTTGT-AGAAAGAGGTCG--TCTATT-AGTCTTAGATGATTTTGGCTAGAGTCCC
TAGAGACATGTT-GACTAGATCATACAT--GAGA-TCCCCAAACCTATAT-AAATGTCTC
TTTAGTACTGCAAGGGGTTAGGGTGGAAATGTCATGCATTGTATAA---GAACAGAAGGA
GGAAGGCTGCCTCAACAGGGG---------CAGAAGAGAAACTCCCTGCTT
```

MARKER 129

>WSINE1

---TTCAAATCCAG
CCTCAGACAC---------TTACTAGCTGTGTGACCCTGGGCAAGTCACTTAACCCCAAT
TGCCT--------------CAAAAAAAAAAAAAA--------------------------

>L1_Opos2_3end

----------CATTTCTTATAGCACAATAGTATTCCATCACAAT-----CATATACCACA
ACTTGTTCAGCCATTCCCCAATTGATGGGCA-TCCCCTCAATTTCCAATTCTTTGCCACC
ACAAAAAGAGCT--

>WSINE1

-------------TTTTTTTTTTTTTTGAGGCAATTGGGGTTAAGTGACTTGCCCAGGGT
CACACAGCTAG---------TAAGTGTCTGAGGCTGGATTTGAACTCAGGTCCTCCTGAC
TCCAGGGCTGGTGCTCTATCCACTGCACCACCTAGCTGCCCCTC----------------

>WSINE1

---TTTTT
TTTTTTTTTG-AGGCAATTGGGGTTAAGTGACTTGCCC------AGGGTCACACAGCTAG
TAAGTGTC----TGAGG---CTGGATTT--------------------------------

--GAACTCAGGTCCTC
CTGACTCCAGGGCTGGTGCTCTATC-CACTGCACCACCTAGCTGCCCCTC----------

>WALLSI2

-------------------------------GGGGGCAGCTAGGTGGTGCAGTGGATAGA
GCACCAGTGCAGGAGTCAGGAGGACCTGAGTTCAAATCTCACCTC-AGACACTTGACACT
CACTAGCTGTGTGACCTTGGGCAAGTCACTTAACCCCAATTGCCT---------------

>Mdo
CAGCACATGGTGACGATGG--CTGGAACAGAGGAAGGGTTATTAACA-------------
------------------------------------CCAACTTCAT--------------

-----CCTCCACCCCCTC-AAATGACCCTGCACTGTGGCTAAACAAGCTACTAAG--TTA
GAAGCCATGACAGAGGAAGAGAG--------CA----TCTGTACAAACACAA---AGGAG
CACTGT--

---TCT
CAATAAA-----TGCAAAGAAATCC-----------------------------------

--ATGCAACCAC
TCTTTT-CT------AACCCATGTGACCAAG-TGGACA-GTGAACGATGTGCT-TGTGAG
AAAGGG--CTCATGTTCTC----ATTCCC---------ACCCCTTGAGGTAGGAACCCCA
AGGCTTTAC--CTGAAGGTCCCCCGTTCAACATCCTGGCCGCTGAGCCGAATGTGGATCC
CTTCTTTCAG
>Dvi
GAGCACATGGTGACGATGG--CTGGAACAGAAGATGGGTTATTAACA-------------
------------------------------------CCAACTTNAT--------------

-----CCCCCACCTCCTC-AAATGACCCTGCACTATGGCTAAACAAGTTACTAAGTTTTA
GAAGCCAGGACAGAGGAAGAAAG--------CA----TCTGTACAGACACAA---TGGAG
CACTAT--

---TCT
TAATAAA-----CGCAAAGAAATCC-----------------------------------

--ATGCGACCAC

```
TCTTTT-CTAACCAAGACCCTTGTGACCGAG-TGGACAGGGCAATGATATGCT-TGTGAG
AAAGGG--CCCCTGCTCTCTCTTATTACC---------ACCCCTTGAGGTAGGAAACCCG
AGGCTTTAC--CTGAAGGTCCCCCGTTCAACATCCTGGCCGCTGAGCCGAATGTGNATCC
CTTCTTTCAG
>Mnu
CAGCACATGGTGACGATGG--CTGGAACAGAAGACGGGTTATTAACA-------------
------------------------------------CCAACTTCAT--------------
------------------------------------------------------------
------------------------------------------------------------
-----CCCCCACCTCCTC-AAATGACCCTGCACTATGGCTAAACAAGTTACTAAGTTTTA
GAAGCCAGGACAGAGGAAGAAAG--------CA----TCTGTACAGACACAA---TGGAG
CACTAT------------------------------------------------------
------------------------------------------------------------
------------------------------------------------------------
------------------------------------------------------------
---------------------------------------------------------TCT
TAATAAA-----CGCAAAGAAATCC-----------------------------------
------------------------------------------------------------
------------------------------------------------------------
------------------------------------------------------------
------------------------------------------------------------
------------------------------------------------------------
--------------------------------------------------ATGCGACCAC
TCTTTT-CTAACCAAGACCCTTGTGACCGAG-TGGACAGGGCAATGATATGCT-TGTGAG
AAAGGG--CCCCTGCTCTCTCTTATTACC---------ACCCCTTGAGGTAGGAAACCCG
AGGCTTTAC--CTGAAGGTCCCCCGTTCAACATCCTGGCCGCTGAGCCGAATGTGGATCC
CTTCTTTCAG
>Rra
CAGCACATGGTGACGATGG--CTGGAACAGAAGAATGGTTATTAATA-------------
------------------------------------CCAACTTCAC--------------
------------------------------------------------------------
------------------------------------------------------------
-----CCCCTTCCCTCCC-----AATCCTGCGCTGTGGCTAAACAAGCTGCTACG--TAC
AAAGCCATGACAGAGGAAGTGAGCTCCCAAGCA----TCTGTTC-CACACAA---GGGAG
CGCTGT------------------------------------------------------
------------------------------------------------------------
------------------------------------------------------------
------------------------------------------------------------
---------------------------------------------------------TTT
CAATAAAAGCTGTCCAAAGAAATCA-----------------------------------
------------------------------------------------------------
------------------------------------------------------------
------------------------------------------------------------
------------------------------------------------------------
------------------------------------------------------------
--------------------------------------------------GTAGGACTGC
TATTTT-CAAACCAGGGCCTATTCCATTGAG-TGGACA-GTTAAGGATCTGCT---TATG
AAAGGC--CTCAGACAGTCTATAATTCCC-------AACCCCACCCAGGTGGGAACCCCT
AGGCTTTAC--CTGAAGGTTCCTCTTTCAACATCCTGACCGCTGAGTCGAATGTGGATCC
CCTCTTTCAG
>Cfu
CAGCACATGGTGACGATGG--CTGGAACAGAAGAATGGTTATTAATA-------------
------------------------------------CCAACTTCAC--------------
```


-----TCCCTCCCCTCCC-----GATCCTGTACTGTGGCTAAACAAGCTGCTACG--TAC
AAAGCCATGACAGAGGAAGCAAGTTCCCAAGCA----TCTGTTC-CACACAA---GGGAG
CACTGT--

---TTT
CAATAAAAGCTGTCCAAAGAAATCA-----------------------------------

--GTAGGACTGC
TCTTTT-CAAACCAGGGCCTATTCCACTGAA-TGGATA-GTTAAGGATCTGCT---TATG
AAAGGA--TTCAGACTATCTGTAATTCCC-------AACCCCACCCAGGTAGGAACCCCT
AGGCTTTAC--CTGAAGGTTCCTCGTTCAACATCCTGACCACTGAGTCGAATGTGGATCC
CCTCTTTCAG
>Dgl
TAGCACATGGTGACGATGG--CTGGAAGAGAAAAATGGTTATTAATATAAACTAAACAAA
ACAAAACAAAACCAAACAACAACAACAACAACAAAA-CAACTTCAC--------------

-----CCCCTCTTCCCCTAAAATGAACCTGCACTGACGCTAAACAAGCTATTTAGG-TGA
GAAGCCATTACAGAGGAAGATAGATCCCAAGCA----TCTGTACAAACACAA---CAGAG
TACTGT--

---TCT
TCATAAAAGATGTCCAAAGAAATCAGTGCAAGTGT-----TCTTTTCCTTCCTTCCTTCC
TTTCTTTTGGGAGGCAATCAGGGTTAAGTGACTTTCCC------AGGGTCACACAATAAG
TGGC--------TGAAG---CTGGCTTT--------------------------------

--GAACTCAGGTCCTC
TTGACACCAGGGCTGGTACACTGTG--------CTACCTAGCTGCCTCTT--CAA-TTGC
TCTTTT-TAAACATGTGACT--------GAG-TGGGCA-GTAACATATGCTTG--TTAT-
---GGG---------AATCTCCAATTCCC-------ACTCCCAATGAGGTGGGAATCACT
AGGTTTTAC--CTGAAGGTTCCACGTTCAACATCCTGGCCGCTGAGCCGAATGTGGATCC
CATCTTTCAG
>Nty
TAGCACATGGTGACGATGG--CTGCAACAGAAGAATGGTTATTAATA-------------
------------------------------------CCAACTTCAC--------------

-----TCCCTTCCCCCTC-AAATGATCCTGCACTATGGCTAAACAAGTTANTGAG--TCA
AAAGCCATTACAGAGGGAGAGAGCCCCCAAGCA----TCTGTACAAACACAAC--GGAAG
TACTGC--

---TCT
TAATAAAAGCTGTCCAAAGGGACCAT-----------------CCTTTTCTTTCTTTCTT
TTTTTTTGGGGAGGTAATCAGGGTTAAGCACCTGACCA-------GGGTCACACAGTAAA
TATCCAAG-----GCCG------AATTT--------------------------------

--GAACTCAGGTTCTC
CTGACACTAAGGCTGGTGCTCTATC-CACTATGCCACCTAGCTGCCCTTC------AAGC
TGCCCTAAGCTAAATGTGACAGCTATTTTCCAACCAA-GGCCCCTTCTGACTGAGTAGAC
A---GT-AATGGTA-CACTTGTAATTTCC-------ATCCCCAAAGAGGTTGGAACTACT
AGGCTTTAC--CTGAAGGTTCCTCGTTCAACATCCTGGCCACTGAGTCGAATGTGGATTC
CGTCTTTCAA
>Pta
TAGCACATGGTGACGATGG--CTGAAAAAGAAGAATAGTTATTAATA-------------
------------------------------------TCAACTTCCC--------------

-----CTC-TCCCCCCTC-AAATGACCCTGCACTATAGCTTCACAAGCTACACAG--TAA
GAAACTATGATTGAGGAAGAGAGTTCCCAAAAA----TCTGTACAAACATAAA--AGAGC
ATTGTT--

--TT
TAACCAAAGCTGCCCAAAGAAATCAGCATGACTG--------CTCTTTTCCTTTCTTTTC
TATGATTTGG-AGACAATTAGGGTTAAATGACTTGCCC------AGGTTCACACAATAAG
CGTCTGAG----ACCAC-------ATCT--------------------------------

--GAATTCA-GTTCTC
CTAAAACTAGGGCTAGTGCTCCCCATCATTACACACCTCAGCGGCCTCTG---TGATTAC
TCTTTT-CAAGCCAAGACCCC-ATTGACTAG--AGAG-CAGTAAGGATGTGCTTGTTAAG
A---GT-TCTGA---TCTCTCTAATTCCC------CTGCCCA-------CAGAAACTATT
AGGCTTTAC--CTGAAGGTTCCCCGTTCAACATCCTGGCCACTGAGCCGAATGTGGATTC
CATCTTTCAG
>Scr
TAGCACATGGTGACGATGG--CTGAAAAAGAAGAATGGTTATTAATA-------------
------------------------------------TCAACTTCCC--------------

-----CTCTCCCCCCCTC-AAATGACTCTGCACTGTAGCTTCACAAGCTACACAG--TAA
GAAACCATG--ATTGAGGAAGAGTTCCCAAAAA----TCTGTACAAACATAAA--AGAAC
ATAGTT--

--TT
TAACCAAAGTTGCTCAAAG-AATCAGTGTGACTG--------CTCTTTTCCTTTCTTTTC
TATGTTTTGG-AGACAATTAGGGTTAAGTGACTTGCCC------AGGTTCACAAAGTAAA
CGTCTGAG----ACCAC-------ATTT--------------------------------

--GAACTTA-GTTCTC
CTAAAACCAGGGCTAGTGCTCCATC--ATTACACACCTTAGCTGCTTCTG---TGATTTC
TCTTTT-CAAGCCAAGGCCCTACTGACTAAGATG--G-CAGTAAGGATGTGCTTATTAGA
G----C-TCTGA----CTCT--AATTCCC------CTGCCCA-----------AAACGCT
AGGCTTTAC--CTGAAGGTTCCCCGTTCAACATCCTGGCCACTGAGCCGAATGTGGATTC
CATCTTTCAG
>Mfa
TAGTACATGGTGACGATGG--CTGAAAGAGAAGAATGATTATTAATA-------------
------------------------------------TCAAC-TTTC--------------

-----CCTCTTCCCCCTC-AAATAACCCTGCACTGTGGCTTCACAAGCTTCACAG--TAA
GAAATCATG--ATAGAGAAAGAGTTCCCAAAAA----TCTGTACAAACATAAT--GGAAC
AATATT--

--TT
TAACTAAAGTTGCCCAAAGAAATCAGCATGACTG--------GTTTTCTCCTTTGTCTTC
TACGTTTTGG-AGACAATTAGAGTTAAGTGACTTGCCC------AGGTTCACAGAGTAAG
CATTTGAG----GCCAC-------ATTT--------------------------------

--GAACTCA-GTTCTC
CTAAAACCAGGGCTAGTGCTTTATC--ATTACATACCTTAGCTGCCCCCA---TGACTAC
TCTTTT-CAAGCCAAGGTCCTTCAGATTGAG--AAAG-CAGTAAGGATGTGTTTGTTACA
AAGAGG-TCTGA----CTCTCTAATTCCC------ATGTCC-AAGGTGGTGGAAAGTGCT
AAGCTTTAC--CTGAAGGTTCCCCGCTCAACATCCTGGCCACTGAGCCGAATGTGGATTC
CATCTTTCAG
>Bil
TAGCACATGGTGACGGTGG--CTGGAACATAAGAATGGTTATTAATA-------------
------------------------------------TGAACTTCAC--------------

-----CCCTTTCCCCCTC-AAGTGACTCTGCACTGTGACTAAACAAGCTACTAAG--TGA
GAAGCCATGATAGAGGAAGAGAGCTCCCAGGCA----TCTGTACAAACACAA---AGGCG
CACTAA----TTTTTTTTTT-ACTGGACTAATTTCCTTTATTATTTTTTCATATACCACA
ACTTGTTGAGTAATT-GTCAAATGATGGGCAATGGTTAAAGAGCACTATTCTTTAAAAAT
TT-------------TTTTATTTAATTAAGGCAA-TGGAGTTAAGTGACTTGCCCAAGGT
CACACAGCTAGGTAAATTATTAGGTGTTTGAAGCTGGATTTGAACTCAGGTCCTCCTGAC
TCCAGGGCTGGTGGTCTATCTACTGTGCCACCTAGTTGCCCCAA---GGAGCACTCTT--
-AATAGAAATTGTCCAAAGAAATCAGTCAGTCTGC--------TCTTTTCTTTCTCTTTC
ATTTTTTTGG-AGGCAATAAGGGTTAAGTGACTTATC-----------------------
------------TGAGG---CTGGATTT--------------------------------

--GAACTTAGGTCCTC
CTGACACCAGGGCTGGTGCTCTTTG--------TCATTTAGCTTCTCCTG---CAACTTG
GTTTC--CATACCAAGGTCCTCACAACTGAG-TGGACAGGAAGGGTTCACACT-------
-AAGG-------------TTCTAATTCTC------ATCCCC-----AGTGAGAAACTACC
AGGTTTTAC--CTGAAGGTTCCCCGTTCAACATCCTGGCCACTGAGTCGAATGTGGATCC
CGTCCTTCAG

```
>Pgu
TAGCACATGATGACGGTGG--CTGGAACATAACAAGGATCATTAATA-------------
------------------------------------CGAACTTCA---------------
------------------------------------------------------------
------------------------------------------------------------
-----CCTTTTCCCCTTC-AAATGACTCTGCACTGTGGCTAAACAAG-TTCTAAG--TGA
GAAGCCATGACAGAGGAAGAGAGCTCCCAGGCA----TCTGTACAAACATAA---AGGAG
AACTA----TTTTTTTTTTTAACTGAAATAATTTCCTTTATTA-TTTTTTATTTACCACA
ACTTATTTAATAATT-GTCAAATGATGGGCAATAGTTAAGGAGCACTATTCTTTTAAAAA
AA-------------TTTTATCTATTTAAGGCAA-TGGAGTTAAGTGACTTGCCCAAGGT
ATCACAGCTAGCT-AATTATTAAGTGTCTGAGGCTGGATTTGAATTCAGGTTCTCCTGAC
TCCAGGGCCGGCGCTCTATCCACTGTGCC-----------CCAG---GAAGCACTCTTTT
TAATAGAAATTGTCCAAAGAAATCAGTTCAGCTGC--------------TCTTTTCTTTT
TCTTTTTTGG-AGGCAATAAGATTTAAGCAACTTACC-----------------------
------------CGAAG---CTGGATTT--------------------------------
------------------------------------------------------------
------------------------------------------------------------
----------------------------------------------GTATTTACATCCTC
CTGACACCAGGGCTGGTGCTCAATG--------TCATCTAGCTTACTCTG---CAACTTG
GTCAT--CATACTAAGGCCCCTATGACT-TG-T---GAGGAAGGGTTTATGCT-------
-AAGG-------------TTCCA-TTCAC---------------------------TATC
AGGTTTTAC--CTGAAGGTTCCCCGTTCAACATCCTGGCCACTGAGTCGAATGTGGATTC
CGTCCTTCAG
>Iso
TAGCACATGGTGACGGTGG--CTGGAACATAACAAGGATCATTAATA-------------
------------------------------------CAAACTTCA---------------
------------------------------------------------------------
------------------------------------------------------------
-----CCTTTTCCCCTTC-AAATGACTCTGCACTGTGGCTAAACAAG-TTCTAAG--TGA
GAAGCCATGACAGAGGAAGAGAGCTCCCAGGCA----TCTGTACAAACATAG---AGGAG
CACTAA---TTTTTTTTTTTAACTGAAATAATTTCCTTTATTA-TTTTTTATTTACCACA
ACTTATTTAATAATT-ATCAAATGATGGGCAATAGTTAAGGAGCACTATTCTTTTTAAAA
AA-------------TTTTATCTATTTAAGGCAA-TGGAGTTAAGTGACTTGCCCAAGGT
ATCACAGCTAGCT-AATTATTAAGTGTCTGAAGCTGGATTTGAATTCAGGTTCTCCTGAC
TCCAGGGCCGGCGCTTTATCCACTGTGCC-----------CCAG---GAAGCACTCTTTT
TAATAGAAATTGTCCAAAGAAATCAGTTCAGCTGC--------------TCTTTTCTTTT
TCTTTTTTGG-AGGCAATAAGATTTAAGCAACTTACC-----------------------
------------TGAAG---CTGGATTT--------------------------------
------------------------------------------------------------
------------------------------------------------------------
----------------------------------------------GTATTTACATCCTC
CTGACACCAGGGCTGGTGCTCTATG--------TCATCTAGCTTACTCTG---CAACTTG
GTCAT--CATACTAAGGCCCCTATGACT-TG-T---GAGGAAGGGTTTATGCT-------
-AAGG-------------TTCCAATTCTC------ATCCCCACTGGGATGAGAAACTATC
AGGTTTTAC--CTGAAGGTTCCCCGTTCAACATCCTGGCCACTGAGTCGAATGTGGATCC
CGTCCTTCAG
>Tro
TAGAACATGATGACNATGG--CTGGAAGAGAAGAATGGTTATTAATA-------------
------------------------------------CCAACTTCAC--------------
------------------------------------------------------------
------------------------------------------------------------
-----ACCTTTCTCCCTC-AAATGACACTGCACTATAGGTAAACAAGCTCCTAAG--TGG
GAAGCCAGGGCAGAGGAAGAGAGCTCCTCAGCG----TCTGCACAAACACAAC--AGAGT
```

GCTGTT--

--CT
TAATAAAAGCTGTCCAAAGAAATCAGTGCAAATAC-----TCTTTTTTTTTTCCNTTTTT
TTTTTTTTTTGGGGCAGCTGGGGAAAAGTGACTTGCCT------ACGGTTACACAGTAAG
TT---AAGCGTCTAAGA---CTGGATTC---GGGGATGGTTAGGTGGTGCAGTGAATAGA
GCAC--------------------------------CAATCCTGGAGGT--AAAGGATCT
GAGTTACTAGCCATGTGACCTTGGGCAAGTCACTTAACCCCAATTGCCTGGCCTTAGCCC
CTCCAAAAAG--------TTTAAAAGAAAAGA---CTGGATTT---GAACTTGAGTCCTT
CTAACGCCAGGGTTGGTGCTTTATCC-ACTAAACCACCTAGCTGCACATG---CCATTGC
TCTTTATAAAACCAAGGCCCCCATGACTGAA-TAAACA-ATAATGATACAATT--GTCAG
AAAGGA--CTCAGGCTATCTCCAATTCCC------ATCCT----------------CAGT
AGGCTTTAC--CTGAAGGTTCCCCGTTCAACATCCTGGCCACTGAGCCGAATGTGGATCC
CATCTTTTAG
>Ppe
TAGCACATGATGACGATGG--CTGGAAGAGAAGAATGGTTATTAATA-------------
------------------------------------CCAATTTCAT--------------

-----CCCCTTCTCCCTC-AAATGACCCTGCACTGTGGCTAAACAAGCTGCTAAG--TAA
GAAGCCATGACAGAGGAAGAGAGCTCCCAAGCA----TTTGTACAAACACAAC--AGAAT
GCTGTT--

--CT
TAATATAAGTTGTCCAAGGAAATCAGTGCAAACAC-------------------TCTTTT
CTTTTTTTT----------------AAGTGACTTGCCT------AGGGTCACACAGTAAG
TAAGTAAGTGTGTTAGA---CTGGATTT---GGGGGTGGCAAGGTGGTGCAGTGAAGAGA
GCACCAGCCCTGGA----TTCAGGAGGACCTGAGTTCAAATCTGACCTCAGACACTTACT
AC-TTGCTAGCCATGTGACCTTGGGCA-AACACTTAACACCAACTGCCTGGACTC-CCCC
CTCCAAAAAGTTAAAAAAAAAAAAAATGAAAA---CTGG--TT---GAACTGAGGTCCTC
CTAACACCAGGGTTGGTGCTCTATCT-GCTATACCACCTGG-TGTCCATG---TGACTGC
TCTTTT-CAAACCAAGGACCCCATGACTGAATTGGACA-GTAATGATATGACTGTGTGAG
AAAGGG--TTCAGGCTATCTCCAATTCCC------ATCCTCAA-TGAGATGGTAACCAGT
AGGTCTTAC--CTGAACGTTCCCCGTTCAACATCCTGGCCACTGAGCCGAATGTGGATCC
CATCTTTCAG
>Tvu
TAGCACATGATGACGATGG--CTGGAACAGAAGAATGATTGTTAATA-------------
------------------------------------CCAACTTTAC---CTCAAATCCAG
CCTCAGACACTCGACACACTTACTAGCTCTGTGATCCTGGGCAAGTCACTTAACCCCAAT
TGCCCTGCCTTCCCCCCTCCAAAAAAAACCCCAACCAAAAAAAAATATA---CAAACTTC
AC---CCCCTTCCCCCTC-ATATGACCCTGCACTGTAGCTAAACA-TCTCCTAAG--TGA
GAAGCCATATCAGAGGAAGAGAGCTCCCAAGCA----TCTGTACAAACACAAG--GGAGA
GCTGTT--

--CT
TAATAAAAGCTGTCCAAAGAAATCAGTGTAATCACTCTTTTATTTCTTTTTTTGTTTGTT
TTTTCTTTTGAAGGCAGTCGGGGTTAAGTGACATGCCC------AGGGTCACACGGTAAA

```
TGT--------AAAACA---CTGGATTT--------------------------------
------------------------------------------------------------
------------------------------------------------------------
----------------------------------------------GAACTCAGGTCCTC
TTAACACCAGGGCTGGTGCTCTATCC-ACTCTGCCACCTGGCTGCCCATA---TGACTAC
TCTTTT-CAATCCAAGGTCCCCATGAC-TAAGTGGACA-GTAATGATACAATT---GTAA
GAAGGAG-CTCAGGCTATCTCCAATTCCC------ATTCTTGA-TGAGGTGCTAACCACT
ACGCTTTAC--CTGAAGGTTCCCCGTTCAACATCCTGGCCGCTGAGCCTAACGTGGATCC
CGTCTTTCAA
>Mro
TAGCACATGATGACGATGG--CTGGAACAGAAGAATGGTTATTAATA-------------
------------------------------------CCAACTTCAC--------------
------------------------------------------------------------
------------------------------------------------------------
-----TCCCTTCCCCCTC-AAATGACCCTGCACTGTGGCTAAACAAGTTACTAAG--TGA
GAAGCCATG--ATAGGAAGATATCTCCCAAGCATCTACCTATACAAACACAAC--AGAGT
GCTATT------------------------------------------------------
------------------------------------------------------------
------------------------------------------------------------
------------------------------------------------------------
----------------------------------------------------------CC
TAATAAAAGCTGTCCAAAGAAATCAGAGCAACCAAACATA-----------------CTT
TTTATTTTGG-AGGCAGTTGGAGTTAAGTGACTTGCC-------AGGGTAA----GTAAG
TAAGTATG----TAAGA---CTAGATTT--------------------------------
------------------------------------------------------------
------------------------------------------------------------
----------------------------------------------GAACTGAAGTCCTT
CTAACACCAGGGCTGGTGTTCTATCC-ACTGTGCGATCTGGCTGCCCATG---AGACTAC
TCTATT-TAAACCAAGGCCCCCATGACTGAG-TGAACA-GTAATGATACGACT--CTGAG
AAAGAG--CTCAGGCTATCTCCAATTCCAATTCCTATCCTCAA-TGAGGTGGTAAGCACT
AGGCTTTAC--CTGAAGGTTCCCCGTTCAACATCCTGGCCGCTGAGTCGAATGTGGATGC
CATCTTTCAG
>Vur
TAGCACATGATGACGATGG--CTGAAACAGAAGAATGGTTATGAACT-------------
------------------------------------CCAACTTCAT--------------
------------------------------------------------------------
------------------------------------------------------------
-----CCCCTTCCCCCTC-AAATGACCCTGCACTGTGGCTAAACAAGCTCCTAAG--TGA
GAAGCCATGACAGAGGAAGAGAGCTCCCAAGCA----TCTGTACAAAAACAAT--GAAGC
ACTGTT------------------------------------------------------
------------------------------------------------------------
------------------------------------------------------------
------------------------------------------------------------
----------------------------------------------------------CT
TAATAAAAACTGACCAAAGAAATCAGTGCAACTGC----TCTTTTCTTTCTTTCTTTCTT
TTTTTTCTGG-AGGCAGTCAAGGTCAAGTGACTTGCCC------AGGGTCACACAGTAAG
TAAGTGTC----TAAGG---CCGGATTT--------------------------------
------------------------------------------------------------
------------------------------------------------------------
----------------------------------------------GAACTCAGGTCCTC
CTGACACCAGGGC--------------------------TAGTGCCCCTT---TAACCCC
TCTTTT-TAAACCAAGGACCCCATGACTGAG-TGGACA-GTAATGATACACTT--GTGAG
AAAGGG--CTCAGGCAATCTCCTATTCCC------ATCCTCAT-TGAGGTGGTAACCACT
```

AGGCTTTAC--CTGAAGGTTCCCCTTTCAACATCCTGGCCGCTGAGCCGAATGTGGATCC
CATCTTTCAG

MARKER 135

>MAR1a_Mdo

-----------------------------------GGGGCAGCTAGGTGGCGCAGTGGAT
AGA-----GCGCCGGGCCTGGAGTCAGGAAGACTCATCTTC-CTGAGTTCAAATCTGGCC
TCAGACACTT--------ACTAGCTGTGTGACCCTGGGCAAGTCACTTAACCCTGTTTGC
CTCAGTTTCCTCATCTGTAAAA-TGAGCTGG-AGAAGGA---------------------
--AATGGCAAACCACTCCAGTATCTTTG-CCAAGAAAACCCCAAATGGGGTCACGAAGAG
TCAGACACGACTGAAAAATGACTGAACAAC----------------AACAACAA------

>SINE1_Mdo_partial

---------------------GGAACCAATACACAGTATTGATTCCAAGATGGAAGGTAA
GGGTTTAAAAAAAAAAAAA---

>Mdo
CTATGCTAAGTTCTTGCTTCAACAAACTTTTTTCCATGCTTCAAGTTCATCATGTTCAG-
-GTATGACCCTAAGA-----ACATATGAAATAATATGTTCAT------------------
-------------------TGAGGAATAGTAATTTTGTTTATGAGTTATAGTAGAAGATG
AAAT-AGTTTATAGAAAAA---

```
------------GAATTTTAACATGTGATTCATTGTATAAAATTTCATTCAATACATTCG
GC--TTTGTTTTTATGTAAT----------------------------------------
------------------------------------------------------------
------------------------------------------------------------
------------------------------------------------------------
------------------------------------------------------------
----------------------------------------------TACTAT---ACATG
TT--------TCATAAGTGG-GTATGTGTGTAGAGGTTTTTT---CTAGTATAGTTTTAC
AA-T--GATTATTCTTTTA-TGCTT-GTGCCACTTTATATCTTTTAAAAATTCATTTTTA
A-CATTTTCTTTTTAGTT------------------------------------------
------------------------------------------------------------
------------------------------------------------------------
------------------------------------------------------------
------------------------------------------------------------
------------------------------------------------------------
------------------------------------------------------------
----------------------------GGAGTCCCTTCTACAGTTATGGCTTACATTAA
GTCTAAATTCTAATTCCTCTGGAAGTAAAGAACATGGA
>Dvi
CTATGCTAAGTACTTGCTTCAACAAACTTTTTTCCATGCTTCAAGTTCATCATGTTCAG-
-GTATGGCCCTAAGA-----AAACATGAAGTTAT--GTTCTT------------------
-------------------TGAGGAATCATGATTATATTTACAAGTTACAGTATTAGATG
AAAA-GGTTTATGGAAAAA-----------------------------------------
------------TTATTTTGACACTTGATTCAATGTATAAAAATTCACTCAATACAATAG
AC--TTTATTTTTATGTAAT----------------------------------------
------------------------------------------------------------
------------------------------------------------------------
------------------------------------------------------------
------------------------------------------------------------
----------------------------------------------TACTAT---ACATG
TT--------TCATAAGTGGTGAGTATGTGTAGAAGTTTTT----CTAATACAGTTTTAG
GAAT--GATCATTCTTTTA-TACTT-GTGCCACTTCATACCTTTGGTTCCAGATTTGAAA
A-CAATTATTTATTTTTT-------------------AAGCAGAAATTGTATGTAATGGG
GGGTTGCAAAGGGTCTCAGTTGATTGAGAGTCAGGTCTAGAGGTGGTACATCTGGAAATC
AGTTCTGTGCCCTTCAATATTTCCTTGGTGTAGGCAAGA---------------------
-----------------------AACCAATACACAGTATTGATTTTAAGATGGAAAAAAA
--TGTTAAAAAAAA--TTTTTTTAACTGAAATAATAAGTTGTATGTAACTAATGGAGG--
AAAT-AAGATGGCAAGAGCAAATGTCTATCATGAATATAATTATTTATATAACCTTTAA-
ATGTGTTTCA-ATTTTTAAAAAATTATACTATTGACTGTACCAACCTTTTAAAATTTCAT
TTTTGTTATTTACTTTTT----AG--TTGGAGTCTCTTCTACAGTTATGGCTTACATTAA
GTCTAAATTCTAATTCCTCTGGAAGTAAAGAACATGGA
>Mnu
CTATGCTAAGTACTTGCTTCAACAAACTTTTTTCCATGCTTCAAGTTCATCATGNTCAG-
-GTATGGCCCTAAGA-----AAACATGAAGTTAT--GTTCTT------------------
-------------------TGAGGAATCATGATTATATTTACAAGTTACAGTATTAGATG
AAAA-GGTTTATGGAAAAA-----------------------------------------
------------TTATTTTGACACTTGATTCAATGTATAAAAATTCACTCAATACAATAG
AC--TTTATTTTTATGTAAT----------------------------------------
------------------------------------------------------------
------------------------------------------------------------
------------------------------------------------------------
------------------------------------------------------------
----------------------------------------------TACTAT---ACATG
```

```
TT--------CCATAAGTGGTGGGTATGTGTAGAAGTTTTT----CTAATACAGTTTTAG
GAAT--GATCATTCTTTTA-TACTT-GTGCCACTTCATACCTTTGGTTCCAGATTTGAAA
A-CAATAATTTATTTTTT-------------------AAGCAGAAATTGNATGTAATGGG
GGGTTGCAAAGGGTCTCAGTTGATTTAGAGTCAGGTCTAGAGGTGGTACATCTGGAAATC
AGTTCCGTGCCCTTCAATATTTCCTTGGTGTAGGCAAGA---------------------
-----------------------AACCAATACACAGTATTGATTTTAAGATGGAAAAAAA
--TGTAAAAAAAAT--TTTTTTTAACTTAAATAATAAGTTGTATGTAACTAATGGAGG--
AAAT-AAGATGGCAAGAGAAAATGTCTATCATGAATATAATTGTTTATATAACCTTTAA-
ATGTGTTTCA-ATTTTTAAAAAATTATACTATTGACTGTACCAACCTTTTAAAATTTCAT
TTTTGTTATTTACTTTTTTTTTAG--TTGGAGTCTCTTCTACAGTTATGGCTTACATTAA
GTCTAAATTCTAATTCCTCTGGAAGTAAAGAACATGGA
>Cfu
------------------------------------------------------------
-GTATGGCAGTGAAA-----ACACATGAAATTATCATTTTTT---CTTAGGAATTCACCT
AAGATA-GTGATTATGT--TTAGAA-----TTAAAGATTTGATG------TTGGAGTGGG
AGAT-GGTTTACAGAAAAA-----------------------------------------
------------GTGTTCTGACATTTGATTCAATGTATCAAAGCTAATTCAAGGCTGCAG
TC--CTTATTTTGATGTAGT----------------------------------------
------------------------------------------------------------
------------------------------------------------------------
------------------------------------------------------------
------------------------------------------------------------
----------------------------------------------TACTAT---ACATA
GA--------GTTACACAA-----TTAGTGTATATGTTTTTAT--ATAGTGCAGTTTTAT
AATTAACATTATACTTCTA-TATTT-GCGTTGCTATATAGTATTATGTCCAGATTTGAAA
A-CAGTTTTATATATTA-------------------------------------------
------------------------------------------------------------
------------------------------------------------------------
------------------------------------------------------------
----------------CCTAAATAGAACTTTTAATGAGTTGTATTTAATTT----AGG--
AAAT-GAGCTGGCAGGAG-------------------------TTTACATAATCTTTAA-
ATAGGTTTCT-GTTTTTAAAAC-TTAAACTGTTAGCTATACCAATATTTTATAAATTAAT
TTTTAATATTTACTTTTT----AG--TTGGAGTCACTACTACAGTTATGGCTTACATTAA
--------------------------------------
>Dgl
CTATGCTGAGTAATTGCTTCAACAAACTTTTTTCCATGCTTCAAGTTCATCATGTTCAG-
-GTATGACACTGAGA-----ACACATGAAATTATCATTTTTT-GCTTTATGTATTCATTG
AGGAAAACTGATTATGT--GTAGAA-----GTAATATTTTGGGGAGTAAAATGTTGGGGA
GGAA-GATTTATAGAAAAA-----------------------------------------
------------GTATTTTGATATTTGATTCAGTGTATCAAAATT----CAAGACACAGG
GC--TTTATTTTTATGTAAT---AACTATGTATATG--------AGGCAGTTCGGTGGAT
AAAGTGCTATATCT-----GGAGTC----------TTCTTC-CTGAGTTCAAATCCAGCC
TCAGATACTTGTCACTCAGCCACCTGTGTGACCCTGGACAAATCATATAGCCCTATTTGC
CTCAGTTTCCTCATCTGTAAAA-TGAGTTGG-AGAAGGA---------------------
--AACAACAAACCACTC-CAGTATCTTTGCCAAGAAAACCCCAAATGGGTTCATGAAGAG
TGTGATTCGAATGAAAA-CGCTTGAAAAAT----------------AACCTCT--GCATG
TG--------GTTACAATAAATGAGGATGTATATAGGTTTTTT--CTAGTACAGTTTTAG
TACCAAGATTATTCTTTT--GTGTTTGTGCTACTGTATAGCATTAGAATCAGATTTTAAA
A-CATTTATTTATTT---------------------------------------------
------------------------------------------------------------
------------------------------------------------------------
------------------------------------------------------------
--------------------AATGTGAATTTTAATAAATTGCAACGAACTA--GGAAATA
```

```
AGAT----------GGAGTAAATGCTAAGCTGAAATAA--TTATTTACGTAATCTTTAA-
TTGTGTTTCA-GTTTTTAAAAA-ATGTACTGTTGGCTATACAAACCTTCTAAAGCTTCAT
TTTAAATATTTACTTTTT----AG--TTGGAGTCCCTTCTACAATTATGGCTTACATTAA
GTCTAAACTCTAATTCTTCTGGGAATAAAGAACATGGA
>Nty
CTATGCTTAGTACTTGCTTCAACAAACTTTTTTCCATGCTTCAAGTTCATCATGTTCAG-
-GTATGGCAGTGAAA-----ATTCAGGAAACTATGATTTTTT-GCTTTATGCATTCATTG
TGGAAAATTGAGTCTGT--GTAGAA-----GTAACGATTTGATATTA--TTTATTGGGGA
GAAA-GGATTTTAGAAAAA------------------------------------------
------------GTATTTTGATGTTTGATTCAGTACATCAAAATT----CAAGACACCAG
TC--TTTATTTTTATGTAAT---AACTGTGCTTATG--------GGGCAGTTAGAATG-A
AAAGTACTGGGCCT-----GGAGTTAAGAAGACTCATCTTC-CTGAGTTCAGATACAGTC
TCAGAAACGT--------ATTACCTGTTTCACCCTGGACAAGTCATGTAACCCTGTTTGC
CTCGGTTTCTTCATTTATAAAAATTAGCTGG-AGAAGGA---------------------
--AATGGCAAGCC--TC-CAGTATCTTTGCCAAAATAACCCCAAATTGGGTCAAGAAGGG
TTGGACTTGACTGAATATCGACAATCTATT------------------CATATA-AT---
----------TACTTA---TGTGAGGATTTAAATAGTTTTTTTC-CTAATACAGTTTTAG
GA-CAAATAATATTTAA-----GGTTTTGTTACTGTATAGCATTAAGACCGGATTTTAAG
A-TATTTATGTAAATTTT------------------------------------------
------------------------------------------------------------
------------------------------------------------------------
------------------------------------------------------------
---------------------ACTGAAATTTTAATAAGTTTTATGCAACTAATCTGGG--
AAAT-AAGATGACAGGAACAAATGTTGAGCTGGAATAAAATTATTTACATCATATTTAA-
TTATGTTTCA-GTTTTTAAAAA-TTGTACTGTTGACTATAACAA-TTTTTTAAACTTCAT
TTTTAATATTTTT---------AG--TTGGAGTCTCTTCTACAATTATGGCTTACATTAA
GTCTAAATTCTAATTCCTCTGGTAATAAAGAGCATGGA
>Pta
CCATGCTAAGTGCTTGCTTCAACAAACTTTTTTCCATGCTTCAAGTTCATCATGTTCAG-
-GTATGACACTGAAA-----ACATATTAAATTGTCATTTTTTTACTTTAGACATTCATTG
AGGCAAAGTGTGTATAAAA-----------GTGAAGATGTTGTGTTAGTGTGTTGTGAAG
TTAG-ATTTGGTTTTTTTGTCTTTTTTTAA------------------------------
------------GTATTA-GACATTTGATTCAGTGAGTCAAAATT----CAAGACAGCCG
GC--TTTATTTTTATGTAAT---AATTATGCATATG------------AGACGTATGGAT
AAAGTGCTAGACCTAGAGTCAAGTCAGAAAGACTCATTTTC-CCAGGTTCAAACGTTATC
TCAAATACTT--------ACTAGCTTTGTGACCCTAGATGA------GAAACCTGTTTGC
CTCAGTTTCCTCATATGTAACA-TGAGTTGG-AGAAGGA---------------------
--AATGGCAAACCA--C-TAATATCTTTGTCAAGAAAAAACCCA-------TATAAGGAT
--ACATGTAACTGAATAGCAACAACCCATGCCTTTAGTT--------ACATAAG-TTACA
TA---TATAAGTAG-----TAACATGTTACATATAGGTTTTTT--CTAGTATAGCTTTAG
GA-TTAAAATTATTCCTTT-GTGTTTGTGCTACTGTAT-GCATTATGATCAGATTTTAAA
A-CATTTGTGAATTT---------------------------------------------
------------------------------------------------------------
------------------------------------------------------------
------------------------------------------------------------
--------------------AACAG-----TGAATAAAGTGTATATAATTAACCTAGA--
AAAT-AAGATGGTAGGAGCAAACCTTAATCTGGAATATTACTTTTTTCCTAATGATTAA-
GTATGTTTCA-GTTTTTAAAAA-TTGTCCTGTTGAATATATCAAGCTTTTTAAATTTTCT
TTTTAATGATTACTTTTT----AG--TTGGAGTCCCTTTTACAATTATGGCTTACATTAA
--------------------------------------
>Dgo
------------------------------------------------------------
-GTATGACACTGAAA-----ATACACTAAATTATCATTTTTTTACTTTAGACATTCATTG
```

```
AGGCAAAGTGTGTATAAAA-----------GTGAAAATGCTGTGTTAGTGTGTTGTGAAG
TTAG-ATTTGTTTTTTTTGTTTT-------------------------------------
------------GTTTTT-GACATTTGATTCAGTGAGTCAAAATT----CAAGACAGCAG
GC--TTTATTTTTATGTAAT---AATTATGCACGTG------------AGATGTATGGAT
AAAGTGCTAGACCTAGAGTCAAGTCAGAAAGACTCATTTTC-CCAGGTTCAAATGTTACC
TCAAATACTT--------ACTAGCTTTGTGACCCTAGATGA------GAAATCCACTTGC
CTCCGTTTCCGCATGTGTAACA-TGAGTTGG-AGAAGGA---------------------
--AATGGCAAACCA--C-TAGGATCTTTGTCAAGAAAAACCCCA-------TATAAGGAT
--ACACTTAACTGAACAGCAACAACCCGTGCCTTTAGTT--------ATATAAG-TTATA
TA---TATAAGTAG--TAACATAAGTTTACATATAGGTTTTTT--CTAGTATAGCTTTAG
AA-CTAAAATTATTCCCTT-GTGTTTGTGCTACTGTAT-GCATTATTATCAGATTTTAAA
A-CATTTATGAATTT---------------------------------------------
------------------------------------------------------------
------------------------------------------------------------
------------------------------------------------------------
--------------------AACAG-----TGAATAAAGTGTATACAACTGACCTAGA--
AAAT-AAGGTGGTAGGAGCAAACATTAATCTGGAATATTACTTTTTTCATAATGATTAA-
GTATGTTTCA-GTTTTTAAAAAACTGTCCTGTTGACTATACCAAGTTTTTTAAACTTTCT
TTTTAATGACTACCTTTT----AG--TTGGAGTCCCTTCTACAATTATGGCTTACATTAA
--------------------------------------
>Scr
------------------------------------------------------------
-GTAGGGCACTGAAAATTTCACACTCTAAGTTATCAATTTTTTACTTTAGGCATTCATTG
AGGCAATGTATGTATAAAA-------------GAAGATGTTGTTTTAGTGTGTTGGGAAG
TTAG-ATTTTTTTTTTTTA-----------------------------------------
------------ATATTA-GACATTTGATTCAGTGAGTCAAAATT----CAAGACAC---
-----TAATCTTTATGTAAT---AATTATGCATAGG------------GGACATATGGAT
AAAGTGCTAGACCTAAAGTCAAGTCAGAAGGCCTCAGTTTT-CCAGTTTCAAATGTCACC
TCAAATACTT--------ATTAGCTTTGTGACCCTAGATGA------AAAACCTGTTTGC
CTCAGTTTCCTCAGATGTAACA-CGGGTTGG-AGAAGGA---------------------
--ATTGGCAAACCAA-C-TAGGATTTTTGTCAAGAAAAATCCCA-------TATAAGGAT
--ACACTTAACTGAACAGCAACAACCCATGCCTATAGTT--------ACAAAAA-TTACA
TA---TAA--GTAG--TAATATAAGTT-ACATATAGGTTTT-----------AGCTTTAG
A--CTAAAATTATTCCTTT-GTGTTTGTGCTACTGTAT-GCATTATGATCAGGTTTTAAA
A-CATTTATGAATTT---------------------------------------------
------------------------------------------------------------
------------------------------------------------------------
------------------------------------------------------------
--------------------AACAG-----TGAATAAAGTGTATATAACTAATCTAGA--
AAAT-AAGGTGGTAGGAGCAAACATTAATCTGGAATACAACTTTTTTCATAATGATTAA-
GTATGTTTCA-GTTTTTAAAAA-TTGTCCTGTTGACTATACCAAGCTTTTTAAACTTTCT
TTTTAATGACTACTTTTT----AG--TTGGAGTCCCTTTTACAATTATGGCTTACATTAA
--------------------------------------
>Bil
------------------------------------------------------------
-GTATGTCAGTGAGA-----GCACATGTAATTGATTTTTTTTTTTTGCCNCATTTCATTC
ATTTGAAGTAATTATGT------ANAGCTTTAAAGGTTTAATATTAANAGGGTTGGGGAA
AAGG-TTTATCANAANA-------------------------------------------
------------ATATTTTGCCATTTGATCCATTGTGTCAAAATT----CAAGATCCCAT
GTGTTATACTTTTATGTTAT---AACTATGCACATG---GGGTAAT-----TAGGGGAAT
AAAGCGCTGGACCT-----GGAGTCAGGAAGACTCATTTTTTCTGAGTTAAAATCTTGCC
CCAGACACTT--------TTTCCCTGTGTG-ACCTGTACGTGTGATGTGATCCTGTTTGC
CTA--TTTTCTCATCTCTAAA-----GTTGG-----------------------------
```

--AATC----ACTA----TAATAGCTTTGCCATGAATACCCTGAATAGGGTCATGAAGAA
TTGTATTTGACTGAAAA-TAACTGAATAATGTTGTTACATAAG---ATATATAT-GCATG
TA--------GTTTTGTAA-ATGTGGGTGTAAATAAGTTTTTTC-CTGGTACAATTTTAG
GCCTTGAC-TTATTTT----GTGTTTGTGCTCCTATATAGCATTTGGACCAGATTTTTAA
A--ATTTAAGTATTTAT---

------------------ATAATAGAAATTTTAATAAATTGTTTGAAACTAATATATG--
AAAT-AAGATGGTAGGAGCAGATGTTATGCTTGAATGTAATT-----CACAATCT-----
TTATGTTTTA-CTTTTTAA----TCATATTGTTTGCTATTCTAACTTTTTAAAGCTTTGT
TTTCAATAA----TTTTT----AG--TTGGAGTCTCTTCTACAATTATGGCTTACATTAA

>Tro

-GTATGGCACTGAGA-----ACACATGA-----TCATTTTTTTCATTTATTCATTCACTT
AGGAAAAGTAATTTTGTT-------TAGAAGTAAGGATTT-------AAGTGTTGGGCAG
AAAG-GTTTATAGACAAA--
------------GTATTTTAACATTTGTTTAAGTATATGAAAATT----CAAGTCACCAG
TC--TTTATTTTTATGTAAT---AACTATGCATATG---GGTTTAT--CTTTAGTTGGAT
AAAGTGCTGAACCT-----GGAGTCAGGAAGACTTACCTTC---AAGTTCAAATCTGGCC
CTCAGGCATT-------TAATGTCTACGT-AACCTGGACAAGCCACATAT--CTCTTTGT
CTAAG-TTCCATATCTATAAAA-TGAGTTTT-AGAAGGAAGTAGTAGTATCTTTAGTATC
CACTTTAATATCCACTT-TAGTATCTTTGCCAAGAAAACCCCAAATGGAGCTATGAAGAT
TTGGGCTAGACTGAAAA-CGACTGATCAAC----------------AAT-TTAT-GCATG
TA--------GTTACATAA-ATGGGCATGTATATAGGTGTATTT-CTA-GTATAGTACTA
GGATTAACATTTTTTTGTGTGTGTTTATGCTACTTTATAGCATTAGAGCCAGATTTTTAA
GATACTTGTATTTTTAT---

------------------TTAACAGAAATTTTAATAAACTATAAGTAATG----TAGG--
AAAT-AAGATGACAGTATCAAATGTTGAGCTGCAATATAATTATTTAAATAATCCCTTAA
GTATGTTTCA-GTTTTTCAAAA-TTGTTCTCTTGACTATACCAACCTTTTCAAGCTTTAT
TTT-AATATTTACTTTTT----AG--TTGGAGTCCCTTCTGCAATTATGGCTTACATTAA

>Ppe
CCATGTTGAGTGCTTGTTTCAACAAACTTTTTTCCATGCTTCAAGTTCATCATGTTCAG-
-GTATGGCACTGAGA-----ACACATGAAATTATCATTTTTT-GCCTTTTACATTCATTT
AGAAACACTAAT-ATGT--AATATGTAGAAGTAAGGATTTGATGCTAGTGTGTTGGAAAG
AAAG-GTTTATAGAAAAAA---
------------GTATTCTGACATTTGATTAAGTGTATCAAAATT----CAAGTCATCAG
TC--T-------------AT-----CTAT-----------------------AGGCAGAT
AAAGTGCTGAATCT-----GATGTCAGGAAGACTCATTTTT-ATAAGTTCAAATCTGGCC
TCAGATGTTT--------ACTATCTATGTAATCCTGGACAAGTCACATAGCCCTATTTGT
CTCAG-TTCCTTATCCGTAAAA-TGAGTTGT-AGAAGGA---------------------
--AATAGTAAACTACTT-TAGTATCTTTGCCAAGAAAACCCCAAATAGGGCCATGAAGAT
TTGGACTAGACTGAAAA-CAACTGACCAAC----------------AACTTAAT-GCATG
TA--------GTTATATAA-GTGGGGGTGTATATAGGGGGTTTTTTTC-CTAACATTTTT
GGAAAAAGATTATTCTTTT-GTGTTTATGCTACTATATTGCATTAGAACCAGATTTTTAA
AACACTTGTGTTTTTAT---


```
------------------------------------------------------------
------------------TTAACAGAAATTCTAATAAATTTTATGTAACTAATGTAGG--
AAAT-AAGATAACAGGAGCGAATGTTAAGCTAGAATATATTTATTTGCATAATATCATAA
ATATGTTTCA-GTTTTTAAAAA-TTGTACTGTTGACTGTACCAACCTTTTTAAGCTTCAC
TTTTAATATTTACTTTTT----AG--TTGGAGTCTCTTTTGCAATTATGGCTTACATTAA
GTCTAAATTCTAATTCCTCTGGGAATAAGGAACATGGA
>Tvu
------------------------------------------------------------
-GTATGGCACTGAGA-----ACACATGAAATTACCATTTTTT-GCTTTATGCATTCTTTT
AGGAAAAGTAATTATGTG-------TAGAAGTAAAGATTTGATGTTAGAGTATTAGGGAG
AAAG-GTTCATATAAAAA------------------------------------------
-----------------CTGTCA------AGAAAGTGTCAAAATT----TGAGACATGGG
GC--TTTATTTTTATGTAGT---AACTATGCTTATA---GGACTAT--GCATCGTTGGAT
AAAGTGTTGGACCT-----ATTGTCAGGAAGACTCATCTTC-ATAAGTTCAAATCTGGCC
TCAGGCATTT--------ACTACCTGTGTGACCCTGGACAAGTCACAGAACTCTTTTTGC
TTCAG-TTCCTTATCTGTAAAA-TGAGTTGG-AGAAGGA---------------------
--AATAGCAAACAGCTC-CAGTATCTTTGCCAAGAAAACCCCAAATGGGGCCACAAAGAT
TTGGACTTGACTGACTG-ATAATGACTGACCAGC------------AACTTAAT-GCGTG
TA------------------GTGGGGATGTATATAGGTTTTTTT-CTA-GTATAGTTTTA
GGACTAAGATTATTCTTTT-ATGTTTATGCTGCTGTGTAGCATTTGTACCAGATTTTTAA
AACATTTATGTATCTA--------------------------------------------
------------------------------------------------------------
------------------------------------------------------------
------------------------------------------------------------
---------------------GCAGAAATTTTAAATAATCATATGCAACTAATATAGG--
AAAC-AAGATGGCAGGAGCAGATGTTAAGTTGGAATATAATTATTAACATAATCTCTAA-
GTATGTTT---GTTTTTAAAAA-TTGTACTGTTGACTATACCAACCTTTTTAAGCTTGAT
TTTTAAAAATTTTTCCTTTTT-AG--TTGGAGTCCCTCCTACAATTATGGCTTACATTAA
--------------------------------------
>Mro
CCATGCTGAGTGCTTGCTTCAACAAACTTTTTTCCATGCTTCAAGTTCATCATGTTCAG-
-GTATAGCACTGAGA-----ACACATGAAATTGTAATTTTTT-GCTTTATACATTCATAT
AGAAAAAGTAATTTTGTG-------TGAAAGTAGAGATTTGATGTTAGAGTGTTGGAGAG
AAAA-GTCTGTAGGAGAA------------------------------------------
------------GTATTT----------------GTATCAAAAGT----CAAGACACCAG
GC--ATTATTTTTATGTAAT---AACTATGCACATG---CGGCTATGGGGATAGGTGGAT
AAAATGCTGGCCGT-----GGAGTCAGGAACAGTCATCATC-ATAAGTTCAAATCTGGCC
TCAGACATTT--------ACTACCTGTGTTACCTTGGAAAGGTCACATAACCCTGTT-GC
CTCAG-TTCCTTATCTTTAAA--TGAATTGGGAGAAGGA---------------------
--AATAGCAAACCACTC-CAGTATCTCTGCCAAGAAAACCTCAAATGGGGCCGTGAACAT
TTGAACTCACCTGAAAA-CTACTTACCAAC----------------AACTTAAC-GCATG
TA--------GTTATATAA-GTGGGGATGTTTATAGGGTTTTTT-CTA-GTATAGTTTTA
GGACTAAGATTATTCTTTT-TTGTTTATGCTACTATATAGCATTAGGACCAGATTTTTAA
AACATTTCTGTACTTAT-------------------------------------------
------------------------------------------------------------
------------------------------------------------------------
------------------------------------------------------------
------------------TTAATGGAAATTTTAATAAATTATAGGTAATTAATACAGG--
AAAC-AAGATGACAGGAGCAAATGTTAAGCTGGAATATAATTATTTACATGATCTCTAA-
GTATGTTTCA-GTTTCAAAAAA-TTGTACTGTTGGCTGTACCAACCTTTTTAAGCTTCAT
TTTTAATATTTACTTTTT----AG--TTGGAGTCCCTTCTACAATTATGGCTTACATTAA
GTCTAAACTCTAATTCCTCTGGGAACAAGGAACATGGA
>Ptr
```

CCATGCTGAGTGCTTGCTTCAACAAACTTTTTTCCATGCTTCAAGTTCATCATGTTCAG-
-GTATAGCACTGAGA-----ACACATGAAATTGTAGTTTTTT-GCTTTATACATTCATAT
AGGAAATGTAATTATGTG-------TTAAAGTAGAGATTTGATGTTAGAGTGTTGGAGAG
AAAA-GTCTATAGAA---
------------GTATTTTGTCATTTGATTAACTGTATCAAAAGT----CAAGACACCAG
GC--ATTATTTTTATGTAAT---AACTATGCATATG---CGGCTATGGGGATAGGTGGAT
AAAATGCTGGATGT-----GGAGTCAGGAACACTCATCTTC-ATAAGTTCAAATCTGGCC
TCAGGCGTGT--------ACTACCTGTGTTACCCTGGAAAGGTCACATA-CCCTGTC-GC
CTCAA-TTTCTTATCTTTAAA--TGAGTTGA-AGAAGGA---------------------
--AATAGAAAACCACTC-CAGTATCTCTGCCAAGAAAACCTAAAATGGGGCCATGAACAT
TTGAACTCAGCTGAAAA-CTACTTACCAAC----------------AACTTA-C-GCATG
TA-----------ACATAC-GTGGGGATGTTTATAGGGTTTTTT-CTA-GAATAGTTTTA
GGACTAAGATTATTCTTTT-TTGTTTATGCTACTGTATAGCATTAGGACCAGATTTTCAA
ACCATTTCTCTACTTAC---

------------------TTAAAGGAAATTTTAATAAATTATATGTAGTTAATACAGG--
AAAC-AAGATGGCAGGAGCAAATGTTAAGCGGAAATATAATTACTTACATGATCTCTAA-
GTATGTTTCA-GTTTCAAAAAA-TTGTACTGTTGTCTGTACCATCCTTTTTTAGCTTCAT
TTGTAATATTTACTTTTT----AG--TTGGAGTCCCTTCTACAATTATGGCTTACATTAA
GTCTAAACTCTAATTCCTCTGGGAACAAGGAACATGGA
>Vur
CCATGCTGAGTACTTGCTTCAACAAACTTTTTTCCATGCTCCAAGTTCATCATGTTCAG-
-GTATGGCGCTGAGA-----ACACGTGAAATTATAATTTTTT-CCTTTATGCGTTCATTT
AGGAAAAGTGATTATTTG------------TAGAAGATTTGATGTTAGAGTGTTGGGGAG
AAAG-GTTTATATAAAAA--
------------GTATTTTGACATTTAATTAAATGTATCAAAATT----GAAGACA---A
GC--TTTATTTTTCTGTAAT---AACG--GCATATG---GGGCTATGCATAG--GTGGAT
AAAGTGTGGGACCT-----GGAGTCAGAAAGACTCATCTTC-ACAAGTTCAGATCTGGCC
TCTGACATTGT-------GTGACTTGTGTGATCCTCGACAAGTCACATAACCCTGTTTGC
CTCAG-TTCCTCGTCTGTAAAA-TGAGTTGG-AGAAGGA---------------------
--AATAGCAAACTACTC-CAGTATGTTTTCCAAGAAAACCCCAAATGGGGCCATGAAGAT
TTGGACTCAACTGAAAA-CAACTGAACAAC----------------AGCC-TGT-GCATG
TA--------GTTACATAA-GTGGGAATGTATATAGGTTTTTT--CTA-GTATAGATTTA
GGATTAAGATTATTCTTTT-GTGTTTATGCTATT----------AGGATGAGATTTTAAA
A-CATTTATGTTT---

-----------------------AGAAATTTTAATA---TGTGTGT-ATGTATGTAGC-T
AATC-TAGGAAATAAGAGTAAATGTTAAGCTGAAGTATAATTATTTACATA-AATTTAA-
GTATGTTTCATCTTTTTAAAAA-TTATACTGTTGACTGTACAAATTTTTTTAAGCTTCAT
TTTTAATATTTACTTTTT----AG--TTGGAGTCCCTTCTACAGTTATGGCTTACATTAA
GTCTAAATTCTAATTCCTCTGGGAATAAAGAACATGGA

MARKER 139

>MAR1a_Mdo

--TTGTTGTTGT
TGTTC-AGTCA---
-----------------------------TTTTTCAGTCGTGTCTGACTCTTCGTGACCC
CATTTGGGGTTTTCTTG-GCAAAGATACTGGAGTGGTTTGCCATTTCCTTCTCCAGCTCA
TTTTACAGATGAGGAAACTGA-GGCAAACAGGGTTAAGTGACTTGCCCAGGGTCACACAG
CTAGTAAGTGTCTGAGGCCAGATTTGAACTCAGGAAGATGAGTCTTCCTGACTCCAGGCC
CGGCGCTCTATCCACTGCGCCACCTAGCTGCCCC--------------------------

>P7SL_MD

--GGCATGGTGGCACATACCTGTAATCCCTGCTA----CTGGGGAGGCTGAGGCTGGTGG
ATCGCTTGAGTTCAGGAGTTCTGAGCTGCAGTAGGGCTAAAGACCAATTGGGTGTCTACA
CTAAGTCTGGCACCAATATGGTGAGCCCCTAGGA-GTGGGGG-------GCCACCAGGCT
GCCTAAGGAGGGGTAAACTGGCCCAGGTCAGAAATGGAGCAGGTCAAAGCTTCTGTGCTG
ATCAGTAGT-GGGATCAGGCCCATGAGTGGCCACTGTACTTCCAGCC-TGGGCAAGATAG
GGAGACCCAGTCTAAAAA--

>Mdo
CCCAAGTAACCAGCAACAACAAAGGGACAGGGTGTGAGTTTGAGCTATGGGATTGTGGAG
GAGATACAAA--GTAAGTGATCTCCTGTTTTCTTCTGAGAGATAGAGACAAAGACAGAA-

--TGTGTGTGTT------
----------------TAA-TGGATGATCAGAAAAATGGG-CTGAGATTCCCATCCTT--
----------AAA----TCTATATCGTGTCTTGGCCCTTATGGGCCAGGATCCAGCATCT
TCATCACCCCTACATTAATACAAGCAAGACACTCTAAGAAACACCCCTCCCCACAGGACC
GAGGGACCGGTTTC--

----TGATTCTGCTC--TTCCTTCTCT-GAAG--GTTTGAATCATGCTGGCCTGCCCTCA
TGAAGGACTCTCATGGGGTCGTGATCATCTTCAACGCCGATATCCCCA
>Dvi
CCCAAGTGACCAGCAACACCAAAGGCACAGGGTGTGAGTTTGAGCTGTGGGATTGTGGAG
GAGATACAAA--GTAAGTGTTCTC-TGGTTTG-----AGAGAGAGA--CAGAGAGAA---

--TGCGTGTGTT------
----------------CA--TGGGTGGTCCTAAAGATGGGCTTTTCTAACAGGTCTGT--
-----------------GAACGATCATGTCTTGGCCCTGGTGGGCCA-GACCGAGCATCT
TCATCACCCCTAGCTCAATACAAGCAAGAACTGG--------------------------
-------CCCTCTC--

```
------------------------------------------------------------
------------------------------------------------------------
------------------------------------------------------------
------------------------------------------------------------
------------------------------------------------------------
------------------------------------------------------------
----TGATTCTGCTC--TTCTT-CTGC-CAAG--GTTTGAATCATGCTGGCCTGCCCTCA
TGAAGGACTCTCACGGGGTCGTGATCATCTTCAACGCTGATATCCCCA
>Rra
CCCAAGTAACAAGCAACAACAAAGGGACTGGATGTGAGTTTGAGCTGTGGGATTGTGGAG
GAGATACAAA--GTAAGTGTTTTCCAGCTTTCTGTCAAAGGAGGGTATGTGTAGCT----
--GGCATGGTGGCTCATGCCTGTAATCCTAAC--CTTGGGAGGCTGAGGCTAGCAGATCC
TCTGAGCCCACAGTAAGTGGGCC------------ATGCTGATCAAGTGTTTGCACTTAA
GTTTGGCATTATAATGGTGAACCCCTGAGAGCTAGGGTGGGGGGG-ACACCAGGTTGCCT
AAGGAGGGACAGACCGGTTCAGGTC--AGACACAGCAGGTCAA-GCTCCTGTGCCAATCA
GTAGTAGGGATCATGCCTGTGAGTGGCTACAGCTAT--------GGGTAAGACA-GGAGA
CCTAGCCTCAAAAATAAAACAAAACAAA----------------------GGAGGATATG
------------------------------------------------------------
------------------------------------------------------------
------------------------------------------------------------
------------------------------------------------------------
------------------------------------------------------------
-------------------------------------TGTG-----TGTGTGTG------
-------------------TTAAATGTTTATAAAAATGACCT---CAGAAGGCAATTTGT
GCCTATAAGCCTG----TATCTCTCACCTT--GGCCTTCATGGGCCAATATCATACATCT
TCATCACTACTAGATTAATACAAATGAGA-------------------------------
------------------------------------------------------------
------------------------------------------------------------
------------------------------------------------------------
------------------------------------------------------------
------------------------------------------------------------
------------------------------------------------------------
------------------------------------------------------------
-------------------------------------------------------CTCCA
GGCATGATGTGGCTCTTTTCTTTCTCT-GGAG--GTTTGAATCATGCTGGCCGGCCCTCA
TGAAGGACTCTCATGGAGTGGTGATCATCTTCAATGCTGACATCCCCA
>Cfu
CCCAAGTAACAAGCAACAACAAAGGGACTGGATGTGAGTTTGAGCTGTGGGATTGTGGAG
GAGATACAAA--GTAAGTGCTTTCCGGTTTTCTGTCAAAGGAGGGTATAAGTAGCT----
--GGCGTGGTGGTGCATGCCTGTAATCCTAAT--GTTGGGTGGCTGAGGCTAGCAGATCC
TCTGAGCCTGCAGGTTCAGGGCCGAAGTGGG--CCATGCTGATCAAGTGTTAGCACTTAA
GTTTGGCATTATAATAGTGAGCCCTAGAGAACTGGGGGGAGGGGAGCCACCAGGTTGCCT
AAGGAGGGACAAGCCAGCTCAGGTTGGAAACGAAGCAGGTCAA-GCTCCTGTGCCAATCA
GTAGTAGGGATCATACCCGTGAGTGGCTACAGCCAT--------GGGCGAGACA-GGAGA
CCCAGCCTCAAAAACAAAACAAAACAAAACAAAAAAACAAAGGAGGG---TATATGTGTG
AGTGTGTGTGTATG----------------------------------------------
------------------------------------------------------------
------------------------------------------------------------
------------------------------------------------------------
------------------------------------------------------------
-------------------------------------TGTG-----TGTGTGTT------
-----------------AAATAGATGTTTATAAAAATGACCT---CTGAAGGCAATTTGT
GCCTATAAGCCTG----TATCTCTCACCTT--GGCCTTCATGGGCCAAGATCATACATCT
```

TCATCACCACTAGATTAATACAAATGAGA-------------------------------

---CTCCA
GGCATGATGTGGCTCTTTTCTTTCTCT-GGAG--GTTTGAATCATGCTGGCCGGCCCTCA
TGAAGGACTCTCACGGAGTGGTGATCATCTTCAATGCTGACATCCCCA
>Dgl
CCCAAGTAACTAGCAACAACAAAGGGACCGGGTGTGAGTTTGAGCTGTGGGATTGTGGAG
GAGATACAAA--GTAAGTGTTTGCCAGCTTTCTTGG------------------------

--GATGAAAGAG
GAGGAGGGACAGAGGAGAGAGAGAGAGAGAGAGAGAGAGAGAGAGAGAGAGAGAGAGAGA
GAGAGAGAGAGTGTGTNTGTG----------TTCCNNTCACNNCCANTCCTNTGNGACCC
-TTTTTGGGNTTNTCNTGGCAAANANACTGNACTATTTNNCCNTTTCCCTCTCTNGCTCT
TTTTACCANNGAGNCA---------GAGAGGNTTATNTNGCTNNCCCTGGNTCNCGCTGN
T-----------------------------TTTNATANATACACTTCCTGANTCCACGCC
CAGNGCTCNACTCTNTGTGCCNCCTANNTGCCCT-----TGTATATGGNGTAT-------
----------------TAAATAGATGCTCATATAAATGGCCTTCTCTAAATGCAGTTTGT
GCTCTTAAGCCAG----TAAATATCTCTTCTTGACCTTTGTGGGCCAAGATCAAATATAT
TCATCACTACTGGATTA---CCACTGTGAGAATCAGA-----------------------
-----------------GTGCTACCAACCTGCTGAGAAAGATATTGCAGGGAAGCTCCGA
CATGAGGAGAAAGCGTGAGGTTTCAAAGGTCAGATTGGCGTCAGGAAGTGATGTCTGCTG
CCTGTGGGTCCTGTCAATGAGTGCTATCAGACAATTAGCTTGGAGCTGTGTTTGTGGACA
GCCCTGTTTCCTGCTTCAGAGGGGGCTTCTGGGAGAAGCGAGGGTCTTTTGGTTTCCAGA
CTTTGGCTTGGCGGCAGGGGCAGGCAGAATAGGAAAAGCAGGCAGATCTCATTCTTTATC
CACATTTCTCTTTACTAACCCCTAATATACTTTAATAAATACTTAATGCCCAAAGATTGG
TGCTATACATTTCTAATTTAAGGCAACCTCTCATTAGATTTTAGACATTACAGCTAGAAT
TTTAGACCTTACAATACAAACAACACATTACAATACACACCCCTCCCCAGGAAGACCCCC
ATGCTGACAGGGCTCTTTTCCTTCTCT-GGAG--GTTTGAATCATGCTGGCCCGCCCTCA
TGAAGGACTCTCATGGAGTGGTGATCATCTTCAATGCCGATATCCCCA
>Nty
CCCAAGTAACTAGCAACAACAAAGGGACAGGGTGTGAGTTTGAGCTGTGGGATTGTGGAG
GAGATACAAA--GTAGGTACTTTCCAACTGTGT---------------------------

--GTGTGTGTGT
GTGTGTGTGTGTGTGTGTGTGTGTGTGTT-------------------------------
---------------------------------CCATTCATGTCTGACTCTTA---ACCC
-ATTTGGGGCTTTTCTTAGCAAAGATACTGGCATGGTTTGCCATTTCCTTCTCCAGCTCA
TTTTACAGATGAGGCAA----------GCAGAGTAAAGTGACTTGCCTAGGATCACACAG
CTAATAAGTGTCTGAGGCTAGATTTGAACTCATGAAGATGAGTCTTCCAGTTTCCAAGCC

CAGTGCTCTACCCATTGAGGCACCTAACTGCCCT---TGTT-TCTGTGTGTGTTT-----
-----------------AAATGGATACTCATAAAAATGACCTTCTCTAAATGCAATTTGT
GCCCTTAAACCTG----CAGATT----------GCTCTTATGGGTGAAGAGCAAGCATAG
CC---------------------ATGACA-------GTACCAACAAGACA-TTATAAG-A
ATCA--

--CCCC
ACCCTAACAAGGCTCTTTTCCT-CTCG---AG--GTTTGAATCCTGCTGGCCTGCCCTCA
TGAAGGACTCTCATGGAGTGGTGATTGTCTTCAATGCCAATATCCCCA
>Pta
CGCAAGTAACTAGCAACAACAAAGGGACAGGGTGTGAGTTTGAGCTGTGGGACTGCGGAG
GAGATAGAAA--GTAAGTGTTTTTCAGTTTTCTCAG------------------------

--GAAGAAGGGA
GGGGGAGAGAGATAAAGGGAGGAAATTGGGAGGGAGGGAGACAGAAACAAAAAGAAAGAG
TGTGTGTGTGTGTATGTGTGTTT---------TCCATTCATGTCTGACTCTTTGTGACCC
-ATTTTGGGTGTTCTC-AGCAAAGATACTGGAATGGTTTCCCATTTCCTTCTCTAGCTCA
TTTCACAGATGAGGAAACTGA-GGTAGGCGGGTTGAAGTAACTTGCCCAGAGTCACACAG
CTAGTGAGGGTCTGAGTCTGGATTTGAACTCAGGATGATGAGTCGTCCCAATTCCAAGCC
CAGCGCTCTGCCCACTGCACCAGCTAACTGCCCT---TGTG-TCTATGTGTGTTT-----
----------------AAATAGGG-CT-CATAAAAATGGTTTTCTCTAAAAGCAGTTTGT
GGCCTTAAGCCTG--------TCTCTTCTTAGAGCCTCT--TCTTAGCTTCAATAGACTG
AGATCA------AGCATGCTCACTGAAAA----------CAGACGAGA-CTCTCAAAG-A
CACA--GCCCTCCCTGG---

---GCAGACCCCGC
CC--TGACCAGGCTCTTATCTTCCTCG-GCAG--GTTTGAATCGTGCTGGCCCGCCCTCA
TGAAGGATTCTCAAGGGGTGGTGATCATCTTCAATGCTGACATCCCCA
>Dgo
CCCAAGTAACCAGCAACAACAAAGGGACAGGGTGTGAGTTTGAGCTGTGGGACTGCGGAG
GAGATAGAAA--GTAAGTGCTTTCCAGTTTTCT-CG------------------------

--GGGGAAGGGA
GAGAGAGTGTGTGTG---
-----------------------------TTTTCCATTCGTGTCTGACTCTTTGTGACCC

```
-ATTTTGGGTGTTCGT-GGCAAAGATACTGGAGTGGTTTCCCACTTCCTTCTCTAGCTCA
TTTCACAGATGAGGAAACTGA-GGTAGGCAGGATGAAGTGACTTGCCCAGAGTCACACAG
CTAATGAGGGTCTGAGGCTGGATGTGAACTCAGGATGATGAATCTTCCCAATTCCAAGCC
CAGGGCTGTGCCCGCTGCACCAGCTAACTGCCCT---TGTG-TCTGTGTGTGTTT-----
----------------ACATAGGGTCT-CGTAAAAATGGCTTTCTCTAAAAGCAGTTTGC
AGCCTTAAGCCTG----TAAATGTCTCTTCTTGGGCTTGATGGAGTGAGATCAATGAGCA
TGTTCAC-------TGCAAACAGACAAGA--------------------CCCTCGAAGAA
CACA--GCCCTCCCTGG-------------------------------------------
------------------------------------------------------------
------------------------------------------------------------
------------------------------------------------------------
------------------------------------------------------------
------------------------------------------------------------
------------------------------------------------------------
-------------------------------------------------GCA---GACCC
CC--TGACCAGGCTCTTGTCTTCCTCG-GCAG--GTTTGAATCGTGCTGGCCCGCCCTCA
TGAAGGATTCTCAAGGGGTGGTGATCATCTTCAATGCTGACATCCCCA
>Scr
CTCAAGTAACTAGCAACAACAAAGGGACAGGGTGTGAGTTTGAGCTGTGGGACTGTGGAG
GAGATAGAAA--GTAAGTGTTCTTCAGTTTTCTCAG------------------------
------------------------------------------------------------
------------------------------------------------------------
------------------------------------------------------------
------------------------------------------------------------
------------------------------------------------------------
--------------------------------------------------GGGGAAGGGA
GGGAGAGAGGGAAACAGAGGAGAGAGAGAGCGTGTGTGTGTGTGTGTGTGTGTGTGTGTG
TGTGTGTGTGTGTGTGTGTGTTT-------------TCCATTCATGTCTATTTGCGACCC
-ATTTTGGG---TCTT-GGCAAAGATACTGTTATGGTTTCCCATTTCCTCCTCTGGCTCA
TTTCACAGATGAGGAAACTGA-GGTAGGCAGGGTGAAGTGACTTGCCCAGAGTCACACAG
CCAGTGAGAGTCTGAGGCTGGATTTGAACTCAGGATCATGAGCCTTCCTGATTGCAAGCC
CAGTGCTCTGCCCACTGCACCAGCTAACTACCCT---TGTG-TCTGTGTGTGTTT-----
----------------AAATAGGGGCT-CATAAAAATGGCTTTCTCTAAAAGCAGTTTGT
GGCCTTAAGCCTG----TAAATGTCTTTTCTTGGCCTCGATGGACCAAGATCAAGCA---
----------------TGTTCACCAAAAA----------CAGGCA-AGGCCCTCAAAG-A
CACA--GCCCTTCCTGG-------------------------------------------
------------------------------------------------------------
------------------------------------------------------------
------------------------------------------------------------
------------------------------------------------------------
------------------------------------------------------------
------------------------------------------------------------
-------------------------------------------------GCAG-CGCCGC
CCC-TGACCAGGCTCTTGTCTT------CCAG--GTTTGAATCGTGCTGGCCCGCCCTCA
TGAAGGATTCTCAAGGGGTGGTGATCATCTTCAATGCTGACATCCCCA
>Bil
CCCAAGTAACTAGCAACAACAAAGGAACAGGGTGTGAGTTTGAGCTGTGGGATTGTGGAG
GTGATACAAA--GTAAGTACCATCTGGTCTTCTTCC------------------------
------------------------------------------------------------
------------------------------------------------------------
------------------------------------------------------------
------------------------------------------------------------
------------------------------------------------------------
```

```
--------------------------------------------------AAGCACAAGT
GAGGAGCAAGTGTGTGTGTTTGTG------------------------------------
--------------------------------------------------------TCCA
GTTTGGGGGTTTTCTT-GGCAAAGGTACTGATGAGG------------------------
----CAAAAAGACAGC--------------------------------------------
-TACTAAGTGTCTCAGGCTGAATTTGAACTCTTGACGATGAGTCTTCCTGATTCCAAACC
CAGCATTCTCCCCACTGTGCCACCTAACTGCCCC---TGTG-CTTGTGTGTGTTT-----
---------------------------------------AAATGGAAACATGTAGTTTGT
GCCTTTAAGCCTG----TAAATATCTCTTCTTGGCCTCTATGGGCCAAGATCAAGTATAT
TTATCACAGGATAAACAAGACATATAAGAAATAAT-------------------------
--------CCTCCCTGG-------------------------------------------
------------------------------------------------------------
------------------------------------------------------------
------------------------------------------------------------
------------------------------------------------------------
------------------------------------------------------------
------------------------------------------------------------
-------------------------------------------------TAAGATGTCCA
GAC-TGACAGGACTCTTTTTCTTCTCT-GAAG--ATTTGAATCGTGCTGGCCTGCCCTCA
TGAAGGACTCTCATGGAGTGGTGATCGTCTTCAATGCTGAGATCCCCA
>Pgu
CTCAAGTAACTAGCAACAACAAAGGAACAGGGTGTGAGTTTGAGCTGTGGGATTGTGGAG
GCGATACAAA--GTAAGTGCTGTCCAGTCTTCT-GA------------------------
------------------------------------------------------------
------------------------------------------------------------
------------------------------------------------------------
------------------------------------------------------------
------------------------------------------------------------
--------------------------------------------------GAGACAGACA
GACACACAGAGACAG-AGGGGGCAGGGGT-------------------------------
----------------------------------TTGCA------------TGTG-TCCA
ATTTGGGGGTTTTCTT-GGCAAAGGTACTAGAGTAGTTTACCATCTCCTTCTCTAGCACA
T------AATGAGGCAAA----------CA--GTGAAGTAGCTTGCCCAG----------
---------------------------------------------------------GCC
CAGTGCTCTCCCCACTGTGCCACCTAAC---------TGTG-ATTGTGTGTGTT------
----------------CAAATGGA--TCCA--------------------GGGAGTTTGA
GCTTTTAAGCCTC----TAAGTGTCTCCTCTTGGCCTTTATGGGCCACGATCAAGTCTAT
TTATCACAGGACAAACAAGACACAAGAAATA-----------------------------
------CCCCTCCCTGG-------------------------------------------
------------------------------------------------------------
------------------------------------------------------------
------------------------------------------------------------
------------------------------------------------------------
------------------------------------------------------------
------------------------------------------------------------
-------------------------------------------------TAAGATGCCCA
AAC-TGACA-GGATCTCTTCCCTCTCT-GAAG--ATTTGAATCGTGCTGGCCTGCCCTCA
TGAAGGACTCTCACGGCGTGGTGATCATCTTCAATGCTGACATTCCCA
>Iso
CTCAAGTAACTAGCAACAACAAAGGAACAGGGTGTGAGTTTGAGCTGTGGGATTGTGGAG
GCGATACAAA--GTAAGTGCTGTCCAGTCTTCT-GA------------------------
------------------------------------------------------------
------------------------------------------------------------
```


--GAGACAGACA
GACACACAGAGACAGA-GGGGGCGGGGGT-------------------------------
----------------------------------TTGCA------------TGTG-TCCA
ATTTGGGGGTTTTCTT-GGCAAAGGTACTAGAATGGTTTACCATCTCCTTCTCTAGCACA
T------AATGAGGCAAA----------CA--GTGAAGTAGCTTGCCCAG----------
---GCC
CAGTGCTCTCCCCACTGTGCCATCTTAAC--------TGTG-ATTGTGTGTGTT------
----------------CAAATGGA--TCCA--------------------TGGAGTTTGG
GCTTTTAAACCTC----TAAGTGTCTCCTCTTGGCCTTTATGGGCCACAATCAAGTATAT
TTATCACAGGACAAACAAGACACAAAAAAATA----------------------------
------CCCCTCCCTGG---

---TAAGATGCCCA
AAC-TGACA-GGATCTCTTCCCTCTCT-GAAG--ATTTGAATCGTGCTGGCCTGCCCTCA
TGAAGGACTCTCACGGGGTGGTGATCATCTTCAATGCTGACATCCCCA
>Tvu
CCCAAGTGACCAGCAACACCAAAGGAACGGGCTGTGAATTTGAGCTGTGGGATTGTGGAG
GAGACACCAA--GTAAGTGCTTCCA-----------------------------------

---GGGGAGAGA
GAGAGTGTGTGTGTT---
--------------------------------CCATTCACGTCCAACCCTTCATGACCCC
CATTTGGGGTTTTCTT-AGCAAAGCCACTGGAGCGGTTTACCGTTTCCTTCTCTAGCTCA
TCTTATAGATGAGGCAAA----------GAGGGTGAAGTGACTTGCCCAGGGTCACACAG
CTAGTAAGTGTCTCAGGCTACATTTGAACTGTGC-AGGTGAGTCTTCT-GATTCCAAACC
CAGTTCACAACCCCATGTGTCCCCAAACTGCCCC-----------GTGTGTGTT------
----------------TACATGGAGGCCTATAAAAATGGCCTTCTCTAAATGTGGTTTGT
GCCCTTAAGCCACATTTACAATCTCTCTTCTTGGCCTTTGTGGGCCAAGACCAAGCATAT
TCATCACCGTACAAATGAGACATTAAAAGAAGTAT-------------------------
------CCCCACCCT---

-----GACAGGGCTTCTTTCCTTCTCT-GCAG--GTTTGAATCATGCTGGCCCGCCCTCA
TGAAGGACTCCCATGGAGTGGTGATCATCTTCAATGCTGATATCCCCA
>Mro
CCCAAGTGACCAGCAACACCAAAGGCACAGGGTGTGAGTTTGAGCTGTGGGATTGTGGAG

GAGACACAAA--GTAAGTGATGAACAGCTTTCTTCT------------------------

--AA-TGGAAGG
GAGAGTGTGTGCGCCTGTGTGTGTGCGTGCCTGCCTT-----------------------
----------------------------CCTTCTATTCATGTCCCACTCTTTGGGACCCC
A-TTTGCAGTTTTCTT-GGCAAAGATACTGGAGTGGTTTACCATTTCCTTCTCTGGCTCA
TTTTATAGATGAGGCAAATGA-----------CTT-GGTCACTTG---AGCGTCACTCAG
CTGGTAAGTGTCTCAGGCTGAATTTGAACTCATGAAGATGAGTCTTCCTGATTCCAAGTC
AAGTATTCTACCCACTGTGCCACCTAAGTGCCCT---TGTGTA-TCTGTGTGTGTT----
----------------CACACGGATGCTCATAAAAATGGCCTTCTCTAAATGGG--TTGT
GCCCATTAGCCTG----TAAATATCGCTTCTTGACCTTTATGGGCCAAGATCAAGCGTAT
TCATCAGCATACAGAGGAGACATGATAAGAAGTAC-------------------------
------CCCCTCCC--

----TGACAGAGCTCTTTTCCTTCCCT-GCAG--GTTTGAATCATGCTGGCCCGCCCTCA
TGAAAGACTCTCATGGAGTGGTGATCATCTTTAATGCTGACATCCCCA
>Ptr
CCCAAGTAACCAGCAACACCAAAGGCACAGGGTGTGAGTTTGAGCTGTGGGATTGTGGAG
GAGACACAAA--GTAAGTGTTGAACAGCTTTCTTCC------------------------

--AATGGA----
-------------------------------AAGGA-GTGTGTGTGTGTGTGTGTGTGTG
TGTGTGTGTGTGTGTGCCTGTGTGC----CTTCTATTCATGTCCCACTCTTTGGGACCCC
A-TTTGCAGTTTTCTT-GGCAAAGATACTAGAGTGGTTTACCATTTCCTTCTCTGGCTCA
TTTTATAGATGAGGCAAATGA-----------CTT-GGTCACTCG---AGGGTCACTCAG
CTGGTAAGCATCTCAGGCTGAATTTGAACTCATGAAGATGAGTCTTCCTGATTCCAAGTC
AAGTATTCTACCCCCTGTGCCACCTAAGTGCCCT---TGTGTA-TGTGTGTGTT------
----------------CACACGGATGCTCATAAAAATGGCCTTCTCCAAATGGGGTGTGT
GCCCATTCGCCTG----TAAATATCACTTCTTGGCCTTTATGGGCCAAGACCAAGCATAT
CCATCAGCATACAGAGGAGCCATGATAAGAAGGAC-------------------------
------CTCCTCCC--

----TGACAGAGCTCGTTTCCTTCCTT-GCAG--GTTTGAATCATGCTGGCCCGCCCTCA

TGAAAGACTCTCATGGAGTGGTGATCATCTTTAATGCTGACATCCCCA
>Vur
CTCAAGTAGCTAGCAACAACAAAGGGATAGGGTGTGAGTTTGAGCTGTGGGATTGTGGAG
GAGACACAAA--GTAAGTGTTTTCCGGTCTTCTTCC------------------------

--AAGGGGGAGG
---GAGAGTGAGTGAGTGAGTG
TGTGTGTGTGTGTGTGTGTGTGTG------TTCCATTCATGTCTGACTTTCTGTGACGCG
T-TTTGAGGTTTTCTT-GGCAAAGATGCTGGAGTGGTTTACCGTTTCCTTCTCTAGCTCA
TTTTACAGATGAGGCA---GA-------CAGAGTTAAGTGACTTACCCAGGGTCATACCA
CTAGTAAGTGTCTCAGGCTGAATTTGAACTCATGAAGATGGGTNTTCCTGATTCCAAGCC
CAGTGCTCTAACTGCTGTACCACCTAGCTGCCCT---TGTGTA-TGTGTGTGTGTGTGTG
TGTGTGTGTGTGTGTTCACACGGATGCTCATAAAAATGGCCTTCTGTAAATGCAGTTTGT
GACCTTAAGTCTG----TAAATACCTCTTCTTGGCCTTTATGGGCCAAGATCAAGCATAT
TCATCACCATACACACAAGACATTATAAGAATCA--------------------------
------CCCCCACC--

----TGACAGGACTCTCTTCCTTCTCT-GGAG--GTTTGAGTCGTGCTGGCCCGCCCTCA
TGAAGGACTCTCATGGAGTGGTGATCATCTTCAATGCCGATATCCCCA

MARKER 142

>WSINE1

-------------------------------GAGGGGCAGCTAGGTGGTGCAGTGGATAG
AGCACCAGCCCTGGAGTCAGGAGGACCTGAGTTCAAATCCAGCCTCAGACAC-------T
TAC-TAGCTGTGTGACCCTGGGCAAGTCACTTAACCCCAATTGCCTCAAAAAAAAA---A
AAAA--

>SINE1_Mdo

--TTTTTTTTTTTTTAAA
CCCTTACCTTCCATCTTGGAATCAATACTGTGTATTGGTTCCAAGGCAGAAGAGTGGTAA
GGGCTAGGCAATGGGGGTCAAGTGACTTGCCCAGGGTCACACAGCTGGGAAGTGTCTGAG
GCCAGATTTGAACCTAGGACCTCCCATCTCTAGGCCTGGCTCTCAATCCACTGAGCTACC
CAGCTGCCCCC---

>Mdo
TGAAGGGGATGAGGTAGAG--GTAAGAACACTGAAGTTC---------------------
--------TTCTAGCCCAGAAGATATATA------CCCTCTCTC----------------
---------------------------------ATCACCACC-------TTTCTTTTTGT
CTCAG-TTACTTTTTAGCTAAGATCATGAATACCC-TTTTA----TTAGGTTTGTTCAAA
TTGT---TCAACTC-TTTTCCATTGCTAGTTAGGTGAAGAATGGTAGGGAAGAGTGTCTG
TCTTGTTTGAGAAAAAATCT-GCAAGGAAGAATCCTCACTCCCTCTGTGTTTTTAATTTC
CTATCTTAAGTACTCCTACATGACTGGGCTCTTAATA----TTTCTTGTAGTTTTAACCT
CTTCCTTTTTCTTGCTGTTGCAATTTGCCAAACTTCC-----------------------

--------------AGAGA----ATACCTC-TATTTCAGTAAAAAACTTCCAACTTCCTC
CCTTTAAGAT--

-------AATAGAG-ATAGGTA------TCAAAAATTTC-----------ATGACTTCTT
-GCTCATTCTGATTTAGCAGCAACCTTTTCC----------TTTTCTCTTGATTCAGAGG
GACTCTAC---------------------------AGGTTATCTGGATCATCTCTTA-TT
TCTTCCCTAAC-----TCTAG--GTTTATTCCCGAGCCAACGAACAAGAGCCTTGTGGTT
GGTGGCTGGCAAGGGTTC
>Dvi
AGAAGGGGATGAGGTAGAG--GTAAGAACACTGAAATTCTTCACCTTAAACTA-------
----CCCCTTCTAGCCCAACAGACATATA------CCCTTTCTTCTC-------------
------ATCCCCA--------------------GTAACCACCTTTTACTTGTTTCTTTGT
CTCAG-TTATTTTTTAGCTAAGATCATGGATACCC-TTTTATTTATTAGTTTTGTTCAAA
TTAT---TTCACTN-TTTTCCATNGCTAGTTAGGNGAAAA---ATAAGG-AGAGTGTCTG
TCTTGTTN--GAAAAAATAT-GCAAGGAAGGATCCTCACCCCCTGTGTTT---------C
CCATNTTATGTATTCTTACATACCTGGGCTCTTA--------------------------
---TTTATTTT--AAA
CCCTTACTTTCTGTCTTGAAATCAATACTGTGTATTGATTCCAAGGCAGAAGAGTACTAA
GGG-TAGACATTGGGGGTCAAGTGACTTGACCAGGGTCACACGGCTGGGAAGCGTCTGAG
GCCAATTTTGAACCTAGGATCTCCTGTCTCTAGACATAGCTCTCAATCCACTGAGCCACC
CATCTGTCCCC---AGAACACCTCTATTTC-AGTTTAAAAAACAAAAAAAAACCTCCTTC
CCTTTAAGAT--

-------AATAGAG-ATAGGTA------TCTAAAATTTC-----------AT--------
--------CTGATATAGCATCAACCTTTTCC----------TTTTCTCTTGGTTCAGAGG
GGCTCTAC--------TTT-GCAGGTT--ATTGGACAGTTTTCAGGATCCCCTCTTATTT
TCTTCTCTAAT-----TCTAG--GTTTATTCCCGAGCCAATGAGCAGGAGCCTTGTGGTT
GGTGGCTGGCAAGGGTTC
>Mnu
TGAAGGGGATGAGGTAGAG--GTAAGAACACTGAAATTCTTCACCTTAAACTA-------
----CCCCTTCTGGCCCAACAGACATATG------CCCTTTCTTCTC-------------
------ATCCCCA--------------------GTAACCACCTTTTAC----TTGTTTGT
CTCAG-TTATGTTTTAGCTAAGATCATGGATACCC-TTTTATTTATAAGTTTTGTTCAAA
TTAT---TTCACTC-TTTTCCATTGCTAGTTAGGTGAAAA---ATAAGG-AGAGTGTCTG
TCTTGTTT--GAAAAAATAT-GCAAGGAAGGAACCTCACCCCCTGTGTTT---------C
CCATCTTATGTATTCTTACATACCTGGGCTCTTA--------------------------
---TTTATTTT--AAA
CCCTTACTTTCTGTCTTGAAATCAATACTGTGTATTGATTCCAAGGCAGAAGAGTACTAA
GGG-TAGACATTGGGGGTCAA----------------GTGACAGCTGGGAAGCGTCTGAG
GCCAATTTTGAACCTAGGATCTCCTGTCTCTAGACATAGCTCTCAATCCACTGAGCCACC
CATCTGTCCCC---AGAACACCTCTATTTC-AGTTTAAAAAAAAAAAAAAAAACTCCTTC
CCTTTAAGAT--

-------AATAGAG-ATAGGTA------TCTAAAATTTC-----------AT--------
--------CTGATGTAGCATCAACCTTTTCC----------TTTTCTCTTGGTTCAGAGG
GGCTCTAC--------TTT-GCAGGTT--ATTGGACAGTTTTCAGGATCCCCTCTTATTT
TCTTCTCTAAC-----TCTAG--GTTTATTCTCGAGCCAACGAGCAGGAGCCTTGTGGTT

GGTGGCTGGCAAGGGTTC
>Nty
AGAAGGGGATGAGGTGGAG--GTAAGAGCACTGAAGTTCCCTTCCCCTCAGATACTCATT
TTCTTCTATTCTCAGAAGACAGATACAGATA----TCTTCTCTCCTCCTGTG-------C
TGCCTCCCCCCCA-TCC--------------AC-TTACTACCTTTTACTTGTTCCTTTGT
CTCAGTTAACCTTTCAGCTAAAATCATAGGTACCC-CTTTATTTATTAGGCTTGTTCAAA
CTGTTT-CCCTCTCTTTCACCATTGCTAGTTAGGTGAAGA---ATAATG-AGAGTGTCTG
TCTTGATT--GAAAGTATCT-GCAAAGAAGGAACCTCACCCCATAGGTATTTTTAATTTC
CTATCCTAAGCTCTCCTGCATGCCTGAGGTCTTGTATATTTCATGCTGTAGCTTCAACCT
GTCCCCTTTCCCTGCTGTTGTGATATGCCAGACTTCAAG---------------------

--------------ACAGAGCCCCCATTTC-AGT--------------AAAAGCTTTATC
CTTTTAAGAT--

-------AATAGTG-AGAGGTA------TCTAAAATTTC-----------ATGTCTTCTG
TACTCATTCTGATTAT-CA-GTACTCACCTC-TGGTTTCT-TTTACTCTTGGTTCAGAGG
GGCTCTAC----TTTGTCT-GCAGGTT--ATTGGACAGTTTCAGACACTACCTT-TATTT
TCTTCTCTAATTTCC-TCTAG--GTTTATTCCCGAGCCAACGAGCAAGAGCCTTGTGGTT
GGTGGCTAGCAAGAGTTC
>Scr
AGAAGGGGATGAGGTAGAG--GTAAGAGCTCTAAAGTTACTT------------------
-------------AAAGTAAAAT-ACAGATA----CTTTCTCTTCTCCTGTCAGCTTACC
TCTATCCTACCCCATCCC-------------ACTTTACTGTCTTTTACTTGTTTCTTTGT
CCCAG-TTATCATTCATCTAAGAGCCTGGATATCT-CTTTATTTGTTAGACTTGTTCAAA
GTGTTC-TCCTCTCCTTCACCACTGTTTGTTAAGTGAAGAATGATAAGG-AGAGTGCCTG
TCTTGACT--GAAAATATCTGTAAAAGAGGGAACCTTA-CCCCCAGGTGGTTTTAATTTC
TTATCTTAAGCTTTCCTGCATGCCTAAGGTCTTGTGAATTTCCTGCTGTTG---------
------TTTCCCTGCTGTTCTGATATCCCACATTTCAAG---------------------

--------------AGAGAG--TCCTTTCC-ATT--------------AAAAATTCCATA
GTTTTAA---

-------GATGATAGAGAGGTT---------------TC-----------ATGGCTTCTT
-ATCCATTCTGATTAG-CA---GCTGTTGTC-TGGTTTCT-TTTACTCTTGGTTTAGAGG
GGTTCTACTTTTTTTTTTTTGTAGTT---ACTGGGCAGTTTCCAAGACTACCTC-TGTTT
TCTTCTCTAATTTCCATCTAG--GTTTATTCCCGAGCCAATGAGCAGGAGCCTTGTGGTT
GGTGGCTGGCAAGGGTTC
>Bil
AGAAGGGGATGAGGTGGAG--GTAAGGACACTGAAGTTCCATTCCCTTAAATAGTCATTT
CTAATC----TCGGAAGACAGAA-ACAGTTA----CCTTCTTCTCTCCTC----------
-CCCATCTCCCCCACCTC------------ACAGTTTCTACCTTTCACTTGTTTCTTTGT
CTCAG-TTACCATTCATCGAA-------GATTCCC--TTTATTTATTAGACATGTTCAAA
CTGTTT-CCCTCTCTTTCACTATTGTTAATTAGGTATAGAATGATAAGG-AGAGTGTCTG
TCTTGATT--GAAAATATCT-GCAAAGAAGAAACTTCA-CCCCTAGGTGTTTTTAATTTC
CTATCCTAAGCTTTGCTGCATGCCTGAAGTCTTGTTTGTTTCATGCTAGAGTTTCAATTT
TTCTCCTTTACATATC---ATAATATGCCAAATTTTAAGAA-------------------

```
------------------------------------------------------------
------------------------------------------------------------
--------------AGAGCTC-CC-ATCTC-AGT--------------AAAAATTTCATG
CATTT--------AAGATAATAGAGAGA----AGGGGCAGCTAGATGG--------ATAG
AGCACTGACTCTGATGTCAGGAGGAACTGAGTTCAAATCCTGCCTTAGACAGGTAATATT
TACCTAGCTATGTGACCTTGGGCAAGTCAGTTAATCTCATTGCCTTGTAAAAAA------
-------AAATAGAGAAGGGTA------TCTAAAATCTCTTACCCATTCTATTAGTGGCT
-ACCCATTCT-ATTAG-TG-GCACTCATCTC-TGGTTATTTTTTATTCTTGATTCAAAGG
GGTTTTAC---TTTTATCT-GCAGGTT--ATTGGGCAGTTTCCAAGATCACTTC-TATTT
TCTTCTCTAATTTCC-TCTAG--GTATATTCCCGAGCCAATGAGCAGGAGCCTTGTGGTT
GGTGGCTGGCAAGGGTTC
>Pgu
NGAACGGGNTGANGTGGAG--GTAAGGACACTGAAGTTCCGTTCCCCTT-----------
------CCCGCTTGAAGAG------------------------------------TCATT
TNTAATCTGNCCCANTTC------------AGAGTTGCGACTNTTGAGTNGGTGNTTTGG
CGCAT-TTACCATTCGTCGGAAC-----GATTCCCTTGNATTTGTNAGACATGGGTCAAA
CTGTGTTCCCTCTCTTTCACTATTGTTAATTAGGTATAGAATGATAAGG-AGAGTGTCTG
TCTTGATT--GAAAATATCT-GCAAAGAAGAAACTTCA-CCCCTAGGTGTTTTTAATTTC
CTATCCTAAGCTTTGCTGCATGCCTGAAGTCTTGTATGTTTCATCTA-GAGTTTCAATTT
TCCCCCTTTACATAT----CATAATTGCCAAATTTTAAGGG-------------------
------------------------------------------------------------
------------------------------------------------------------
------------------------------------------------------------
--------------AGAACTC-CC-ATCTC-AGT--------------AAAAACTTCATG
TGTTT--------AAGATAATAGAGAGA----AGGGGCAGCTAGATGG--------ATAG
AGCACTGACTCTGATGTCACGAGGACCTG-------------CCTCAGACAAGTAATATT
TACATAGCTATGTGACCTTGGGCAAGTCAATTAATCTCATTGCCTTTAAAAACAAACAAA
-AAA---AAGATAATAGTGGTA---TTATCTAAAATTTC-----------ATGACTTCTT
-ACCCATTCT-ATTAG-TG-GCACTCATCTC-TGGTTACTTTTTATTCTTGTTTCA----
--------------------GAGTGTT--ATTGGGTAGTTTCCAAGATCACTTC-TATTT
TCTTCCCTAATTTCC-TCTAG--GTTTATTCCCGAGCCAATGAGCAGGAGCCTTGTGGTT
GGTGGCTGGCAAGGGTTC
>Iso
AGAAGGGGATGAGGTGGAG--GTAAGGACACTGAAGTTCCTTCCCCTTAAATAGTCATTT
CTAAT----CTCAGAAGACAGAAACAGA------TACCTTCTCTCTTC------------
-CCCATCTCCCCCACTTC------------ACAGTTTCTACTTTTCAGTTGTTTCTTTGT
CTCAG-TTACCATTCATCGAA-------GATTCCCTTT--ATTTGTTAGACATGTTCAAA
CTGTTT-CCCTCTCTTTCACTATTGTTAATTAGGTATAGAATGATAAGG-AGAGTGTCTG
TCTTGATT--GAAAATATCT-GCAAAGAAGAAACTTCA-CCCCTAGGTGTTTTTAATTTC
CTATCCTAAGCTTTGCTGCATGCCNGAAGTCTTGTATGTTTCATCTA-GAGTTTCAATTT
TCCCCCTTTACATAT----CATAATTGCCAAATTTTAAGGG-------------------
------------------------------------------------------------
------------------------------------------------------------
------------------------------------------------------------
--------------AGAACTC-CC-ATCTC-AGT--------------AAAAACTTCATG
CGTTT--------AAGATAATAGAGAGA----AGGGGCAGCTAGATGG--------ATAG
CGCACTGACTCTGATGTCAGGAGGAACTG-------------CCTCAGACAAGTAATATT
TACCTAGCTATGTGACCTTGGGCAAGTCAATTAATCTCATTGCCTTTAAAAACAAACAAA
AAAA---AAGATAATAGAGGTA---TTATCTAAAATTTC-----------ATGACTTCTT
-ACCCATTCT-ATTAG-TG-GCACTCATCTC-TGGTTACTTTTTATTCTTGATTCA----
--------------------GAGTGTT--ATTGGGTAGTTTCCAAGATCAATTC-TATTT
TCTTCTCTAATTTCC-TCTAG--GTTTATTCCCGAGCCAATGAGCAGGAGCCTTGTGGTT
GGTGGCTGGCAAGGGTTC
```

>Ppe
AGAAGGGGATGAAGTAGAG--GTAAGAGCACTGAAGTTCCTTCCCCTTAAATATGCACTT
CTT----CTAGTCCAAGAA-GACAAAGA-----TTCTCTTTTCCTTCCCTGTTCTCCCCC
TGCCATCTCCCTGTCCCC---CTGCCC--------CATTACCTTTCACTTCTTTCTTTGT
CTCAG-TTACCATTCAGCTAAGATCATGGATACCCCTTAGATTCGTTAGGCTTGTTCCAG
CTGTTC-CCCTCTCTTTCACTGTTGCAAGTTAGGTGAAGAATGATAAGG-AGAGTGTCTC
TCTTGATT--GAAAATATCT-GCAAAGACGGAAATTTATCCCATAGGTATTTTAAATTTC
CTATCCTAAGCTCTCCTGCATGCCTGAGGTCTTGTATACATCATGCCGTAGTTTCAACCT
GTCTCCTTTCTCTGCTGTTCTGATATG---------------------------------

--------------------CCCCTAGCTC-AGT--------------AAAAACTTCATC
CTTTTA--

--------------AGATAGTA-----ATCTAAAATTTC-----------ATGACTTCTT
-TCCATCCTG-ATTAG-CA-GCACTCATCTCTTGATTTCTTTT-ATTGTTGGTTTATAGG
GGCTCTAC--------TTT-GCATGTTTTCCAAGTCAGTTTCTAAAACCATTTT-TGTTT
TCTTCTGTAATTTTC-TCTAG--GTTTATTCTCGAGCCAACGAGCAGGAGCCTTGTGGTT
GGTGGCTGGCTAGGGGTC
>Vur
AGAAGGGGATGAGGTGGAG--GTAAGAGCACCGAAGTTCCTTCCCCTTAAATACTTATTT
CTTCTA-GTTCCATCAGACAGATACAGATAACTTTCTCTTC----------TCCTCCCC-
TGCCATCCCCCAGTCCCCTGCCTGCCCTTCCCATTTATTACCTTTCACTTGTTTCTTTGT
CTCAG-TTACC--------------------------------ATTCAGGCTTGTTCAAA
CTGTTC-CCCTCTTTTTCACCATTGCTAGTTAGGTGAAAA---ATAAG---AAGTGTCTG
TCTTGATT--GAAAATACCT-GCAAAG------------------------TTTAATTTC
CTATCCTAAGCTCCCCTGCATGCCTGACGTCTTGTATATTTCATGCTGTAGTTTCAATTG
TTCTCCTTTCCTTGCTGTTCTGATATGCCATACTTCACG---------------------

--------------AGAGAGCCCCCATCTCAAGT--------------AAAAACTTCATC
CTTTTAAGAT--

-------AATAGAAAAGAGGTA------TCTAAAATTTC-----------ATGGCTTCTT
-CCCATTCTG-ATTAG-CA-GCACTCATCTC-TGGTTTCTTTT-ACTCTTAGTT---AGG
GGTTCTAC----TTTGTCT-GCAAGTT--ATTGGTCAGTTTCTAAGACCATCTC-TGCTT
TCTTCCCTAATTCCC-TCTAG--GTTTATTCCAGAGCCAATGAGCAGGAGCCTTGTGGTT
GGTGGCTGGCAAGGGTTC

MARKER 144

>WSINE1

---TTTTTTT
TTTTTT-----TGAGG-CAATTGGGGTTAAGTGACTTGCCCAGGGTCACACAGCTAGTAA
GTGTC-------TGAGGCTGGATTTGAACTCAGGTCCTCCTGACTCCAGGGCTGGTGCTC
TATCCACTGCACCACCTAGCTGCCCCTC--------------------------------

>MIRc

------------------------GTT---------TCCTC-------ATCTGTAAAATG
GGGATAATAATA--CCTGCCCTGC--CTACCTCACAGGGTTGTTGTGAGGAT--CAAATG
A-GATAATGTATGTGAAAG-----CGCTTT-GTAAACTGTAAA----GTGCTATACAAAT
--GTAAGGGGTTATTATTATT---------------------------------------

>MAR1c_Mdo

-------------------------GGAGGCAGCTAGGTGGCTCAGTGGATAGAGCGCCG
GGCCTGGAGTCAGGAAGACCTGAGTTCAAATC-----CGGCCTCAGACACTTACTAGCTG
TGTGACCCTGGGCAAGTCACTTAACCT-CTGTTTGCCTTAATCCACTGGAGAAGGAAATG
GCAAACCACTCCAGTATCTTTGCCAAGAAAACCC--CATGGACAGTATTGGCGTGCTATG
GTCCACGGGGTCACGAAGAGTC------------GGACACGACTGAACGACTG-----AA
CAACAACAAA--

>Mdo
TACAATGTCACACAACATGAACAGACTTTCCGTTGGAATGCCTACAGTGGCATTCTGGG-
-GTGAGCTCACTGACTT--------------ACTTTTCTTTTTCAAAGAATCTCCACCAT
CATCAAAACTCTATAGATTCTT------------------------------------GT
TCAGCGTAAATAGTGGCCCCTTCAATGAGCCTACATGTTAACTTCTGGCAGGGAGGAGTT
CGTCCCCAC-----TTCCTACCCCGA-----------CCTCTGCCCCAA-----------

---TCTCAAAGTTGG---AAAA
CTGGA---AAGA-AAAAG------CTT---------TTCTT-------ATCT--------

---------------------------------------GT--AAAAGAATTTTAAAGTC
--AACTAGGGTA--

----------------ATCTCTC-----ACTTAGTAAGTCTTAGAAGAGAATTGACTTGC
CCAAGG----------------TATTGTCTTTCT-GGTATGAGTCTAGATATTGGGCTCT
TGGGATGT--ATTAAAAGG-----AATATAAGC--ATCTCCTTTTTCTGGGGAAATTTTT
AGCAATTATGTTTTGGCA-AGTCATTCATTGGTAGCTTGTCTTGGATA---TAGTTTAAT
TT-GGTGACTT--ACTTTT-CAGGGATTA-----------------------GTCT-AAA
TATT--GCTTCTTAAGAA-GTAATAGTTTT--TATTTACTCTTTCCCAG--GATTTGGGA
TGAATGGGAAATTTTCAACAACACATTTACTGGCATGTGGAT
>Dvi
TCCAATGTCACACAACATGAACAGACTTTCCGTTGGAATGCCTACAGTGGTATTCTGGG-
-GTGAGCTCACTGAATG------------CTACTTTTCTTTTTCAAACCATCTCCACCAC

CATCAAAACTCGGTAGATTCTC------------------------------------GT
TCAGGGTAAAGAGGGGCCCCTTCGGCTAGCCTACATGTTAACCTTTGGCAGGGAGGAGTT
GCTCCCCAC-----CC-CTAT----------------CCTCTGCCCCAA-----------

---TCTCAAAGATGG---AAAA
ATAGA---AAGATAAAAA------CTT---------TTCTT-------ATCTGTAAAACG
AGGATGGAGCTGAC---------------CTTGAGAAGGTTGTCATGAGCCTG-CTCAAG
T-GAGATAATGTATGTATATAAT-GCATTTTGCAAACTTGATA----CT---AAGCATAT
-GTCCCATTTTTATTGCAAGA---AACCGATGA-GA--GTT-AAAAAGAATTTTAA--TC
--AACTAGGGTA--

----------------ATCTCTC---------AGTAAGTCTTAGAAGAGAATTGACTTGC
CCAAGGTTAGAGTGTTCTACCATACTGTCTTTCT-GGTATGAGTCTAGGTATTGGGCTCT
TGGTATGT--ATTAAACAGACTATAAAATAAGCATATCTCCTTTT-CTGGGGAAATTTTT
AGCAATTATATTTTGGCA-AGTCATTCATTGGTAGCTTGTCTTANATGCCTTAGGTCTCT
TT-GGTGACTCTTACTTTT-CAGGGATTC-----------------------GTCT-AAA
TATT--GCTTCTTAAGAA-GTAAAAGATTT--TATTTATTCTTTACCAG--GATTTGGGA
TGAATGGGAAATTTTCAACAACACATTTACTGGCATGTGGAT
>Mnu
------------------------------------AATGCCTACAGTGGCATTCTGGG-
-GTGAGCTCACTGAATG------------CTACTTTTCTTTTTCAAACCATCTCCACCAC
CATCAAAACTCAGTAGATTCTC------------------------------------AT
TCAGGGTAAAGAGGGGCCCCTTCGGCTAGCCTACATGTTAACCTTTGGCAGGGAGGAGTT
GCTCCCCAC-----CC-CTAT----------------CCTCTGCCCCAA-----------

---TCTCAAAGATGG---AAAA
ATAGA---AAGAAAAAAA------CTT---------TTCTT-------ATCTGTAAAACG
AGGATGGAGCTGAC---------------CTTGAGAAGGTTGTCATGAACCTG-CTCAAG
T-GAGATAATGTATGTATATAAT-GCATTTTGCAAACTTGATA----CT---AAGCATAT
-GTCCCATTTTTATTGCAAGA---AACCGATGA-GA--GTT-AAAAAGAATTTTAA--TC
--AACTAGGGTA--

----------------AGT-CTT--------AGAAGAGAATT-----------GACTTGC
CCAAGGTTAGAGTGTTCTACCATACTGTCTTTCT-GGTATGAGTCTAGGTATTGGGCTCT
TGGTATGT--ATTAAACAGACTATAAAATAAGCATATCTCCTTTT-CTGGGGAAATTTTT
AGCAATTATATTTTGGCA-AGTCATTCATTGGTAACTTGTCTTAGATGCCTTAGGTCTCT
TT-GGTGACTCTTACTTTT-CAGGGATTC-----------------------GTCT-AAA
TATT--GCTTCTTAAAAA-GTAAAAGATTT---ATTTA-TTTTTACCAG--GATTT----

>Rra
------------------------------------AATGCCTACAGTGGAATTCTGGG-
-GTGAGCTCACTGGCTG------------TTAGCCTTCTTTATGAAAGAATCTCCC----
----CAAACTCTGTAGATTCTT------------------------------------GT
TCAGGGTAACTGGTTGGGCCTTTATCAAGCCTACA-TTTAACTCCCTACAGAAAGAAGCT
GC-CTCCAC-----CT-TTCCT---------------TCCCTCCCCCAAA----------

------------------------------------TTTTTCAT----ATTGGTAACAC-
AGATTG-TAATATTATATAGTA---TTTATTTAATAGGGCTGCCATCAGGCTG-CTCAAG
T-AAGATAATGTATGTAAA-----GCATTTTGTATATTTT-------CTATTATGCATTA
-TTTCCATTGTTATTGAGAGA---AACCAATGG-GA--GCT-AAAAAGGATTTTAAAGAC
--AATTAGGCTA--

----------------ATC-CTC---TCTTAAAAAGAGTCTT-CAAGGGAACTGAATTGC
CCGAA-TT---GTGTTCTACCATACTA-CTTT--------------AGATAGAGGGCTCT
TGGGGTAT--GATAAAAA-----TAATTTAAAT--ATCACCTCTT-CTTGG--AATTTTT
AGCTATTATATTTTGATCAACTCGTTCATTGGTAGCTCT--------------CAGACAG
TTTGAGGACTC--AGTTTT-CAGAAATTA-GTGTCAGTAGCGA---------GTCT----
----------------TAAATAA--TAGGTAATATTTATTCTTTCTCAG--GATTTGGGA
TGAATGGGAAATTTTCAATAACACATTTACTGGCATGTGGAT
>Cfu
------------------------------------AATGCCTACAGTGGAATTCTGGG-
-GTGAGCTCACTGGCTG------------TTAGCCTTCTTTATGAAAGAATCTCCC----
----AAAA-----TAGATTCTT------------------------------------GT
TCAGGGTAACTGGTTGGGCCTTTATCGAGCCTACA-TTTTACTTCCTACAGGAAGAAGCT
GC-CTCCAC-----CT-CTCCC---------------TCCCTCCCCCAAA----------

------------------------------------TTTTT-------------------
-------ATATTATATAGTA---------TTTAATAGGGCTGTCATCAGGCTG-CTCAAG
T-AAGATAATGTATGTAAA-----GCATTTTGTATATTTT-------CTATTATGCATTA
-TTTCCATTGTTATTGAGAGA---AACCAATGA-GA--GCC-AAAAAGGATTTTAAAGAC
--AATTAGGCTA--

----------------ATC-CTC---TCTTAGAAAGAGTCTT-TAAGGGAACTGAATTGC
CCAAA-TT---GTGTTTTACCATACTA-CTTT--------------AGATAGAGGGCTCT
TGGGATAT--GATAAAAA-----TAATTTAAGT--ATCACCTTTT-CTGGA--AATTTTT
AGCAATTATATTTTGATCAATTTGTTCATTGGTAGCTTT-----------ACTTAGACAA
TTTGAGGACTC--AGTTTC-CAGAAATTA-GTGTCAGTATCAA---------GTCT----
----------------TAAGTAA--TAGGTAATATTTATTGTTTCTCAG--GATTT----

>Dgl
------------------------------------AATGCTTACAGTGGAATTCTGGG-
-GTGAGCTCCTTGCTTT------------TAGCCTTTTTT---GAAAGAACATACACTAT
TCCCCAAACTCGGTTGTTTTTTTTT---------------------------------GT
TCAGGGAAAATAGTGTGGGG-------ATCC--------TATAGCAGGAAGGA-GCT-GC
CAGGTGCTGTCTTTCTCCCCTTTCCCCA---------TCTCCAAC-------------TT
TTTTTTTTGGCAGGGC----AATGAGG---TTGACTTGCCCAGGGTCACACAGCTAGNAA
GTGTCAAGNGTCTGAGGCTGGATTTGAACTCTGGNCCTCCTGAATCCAGGGCTGGNGCTT
TATCCACTGCGCCACCTAGCTGTCCCATTT-AAAT--TTTTTTTTTGCACNTCAACTCCN

```
TTTGGAG-ANGAAAA-CTAGA----ANGAAAAAAC-TTTTTTAAAAAAATCTGCAAAGTG
GAGCTAATTATAGCATAATGCTT---ATCACTCATAGGGCTGTCATGAGGTTG-CTCAGA
T-GACAGAGTACAAGTAAA-----GCATCTTGCAAACATTCT-------ATTATGCATA-
-TGTCCATTGTTATAGAGGGA---AACCAGTGA-GA--GCTAAAAAAGGATTTTAAAGTC
--AATTCTGCTA---TTTCTCT----TGGCGGC-TGGATCGCTCAGTGGATAGAACACTA
AGCCTGGAGTTGGGAAGACCAGAGTTCAAATC-TAGCCCCAAACATTTGCTAGCTGTGTG
ATCTTGGGCAAG----CCACTTAA-----------CCT-------CT-GAGAAGGCAAG-
ATA-----TTCCAGTATCTTTGCCAAGAAAACCCCTACACAGCAT--AGTACACAGGGTC
ACAAAGAGTTAGACACAACTCAAC------------AACAAGGCTAATCCCCTCACTTAA
AGAC-----AGTG---AGT-CTT--------CTAGAGAATCT-----------AATTTGT
CCAAGGCTTCAGTGTTCTATCAGCCTGTCTTTTT-GGTATGAGCCTGGATACTGAGCTCT
TGGGATGTGGAATTTAAA----GGAATTTAAGC--ATCATCTTTT-CTGGGGAAATTTTT
AGCTACTATGTTTTGACTGAGTCATTCATT--CATCTTT----TGTAAGATATAACTCAT
TTTGGTGACTCTCAGTTTT-CATGGATTA-----------------------GTGT-AAG
TAGTG--TTTCTTGAGCA-GTAA--TGGGTTATATTTATTCTTTCCCAG--GATTT----
------------------------------------------
>Pta
------------------------------------AATGCCTACAGTGGAATTCTGGG-
-GTGAGCTCACTGCTCTCTGCCTCCTTCTCCCCCTTCCCTTTTGAAAGAACCTCTACTGT
TCCCAAAACTGAGTCAATTCTT------------------------------------GT
TCAGGGTAAATGATGAGATA-------AACGTACAGACTAGTTTCTGAAAGGAAGAA-GC
TGTCTGCTG------TTCCCTCTCCCCA----------TTCCAACCCA------------
------------------------------------------------------------
------------------------------------------------------------
-----------------------------------------TTTTGGAGATGG---AAAA
C---A---AAGAAAAAGAG-----CC----------TTTTTTTA----AACTGTAAGATG
GGAATAAC----------------------------------------------------
---------------------------TTTTGCAAATTTTCT-------GTTACATATA-
-TGTCTATTTTTATTGAGGGA---AACCAATGA-GG--ACT-AGAAGGGATTTTAA--TC
--TGCCCAGCTA---ATTGTC-----TGGACAG--------------------AATACTA
GGCCTGAAGTCAGGAAGACCTGAATTCAAATT-----TGGCCTCAGACACTTACTATGTG
AACCTGGCCAAG----TTACTTAA-----------CCTAATCC-ACTAGAGAAGGAAATG
ATAAACCNCACCAGTATCTTTGCCANGAAAACCC--TATGGACAG---GTCCCCAGGGTA
GTGAACTGTTGGACAAGATTGAAC---------AGCAAAATAACTAGTCCCTTCACTTAA
AGAGAAGACAGTG---AGT-TTT--------AAAAGAGAATT-----------GATTTGC
TCAAGGCTGTAGTGTTCTACCATATTGTCATTTTTGGTATGAGCCTAGTTATAGGGCTCT
TGAAATGT---ATTAAAA----GGCTTTTAAGC--ATTACCTTTT-CTGGGTAAAATTTT
AGCACTGATGTTTTGAGGATATCATTCATTGATACCTTG----TATTAGAC---ATTCAT
TTTGGCGATTC--AGTTTTTCATGGATTATGTGTATA-TAATAAG-----TGATGC-AAG
TAA-----TTCTTGAGT-TGTAA--TGGGTTGTATTTATTTTTTCCCAG--AATTTGGGA
TGAATGGGAAATTTTCAACAACACATTTATTGGCATGTGGAT
>Dgo
CACAATGTCACACAGCATGAACAGACTTTCCGTTGGAATGCCTACAGTGGAATTTTGGG-
-GTGAGCTCACTGCTCTCTGTCTCCTTCCCCTCCTTCCCTTTTGAAAGAACCTCTACCGT
TACCAAAACTGAGTCAATTCTT------------------------------------GT
TCAGGGTAAATGATGGGATG-------AACCTACAGACTAGTTTCTGGAAGGAAGAA-GC
TGTCCGCTG-----TCACC-TCTCCCC-----------TTCCAACCCA------------
------------------------------------------------------------
------------------------------------------------------------
-----------------------------------------TTTTGGAGATGG---AAAA
CTAGA---AAGAAAAAGA------GCTATTTTTTTCTTTTTTTA----AACTGTAAGATG
GGAATAAC----------------------------------------------------
-----------------------------TTGCAAATTTTCT-------GTTACATATA-
```

-TGCCCATTGTTATTGAGGGA---AACCAATGA-GG--ACT-AGAAGAGATTTTAA--GC
--------------------------TGGGCAG--------------------AATACTA
GGCCTAAAGTCAGGAAAACCTGAATTCAAATC-----TGGCNTCAGACACTTACTATGTG
AATCTGGCCAAG----TTACTTAA-----------CCTAATCC-ACTAGAGAAGGAAATG
ATAAACCACACCAGTATCTTTGCCAAGAAAACCC--TATGGACAG---GTCCACNGGGTA
GTG---
--AA-----------GAGTTCG
ACAAGAT-----TGTTCTACCATACTGTCATTTTTGGTATGAGCTTAGTTATAGGGCTCT
TGAAATCT---ATTAAAA----GGCTTTTAAGC--ATTACCTTTT-CTGGGTAAAATTTT
AGCACTGATGTTTTGGGGATATCATT-----------------CATTAGAC---ATTCAT
TTTGGCGATTC--AGTTTTTCATGGATTATATGTATG-TAATAATAAA--TGATGC-AAG
TAA-----TTCTTGAGTA-GTAA--TGGGTTGCATTTATTTTTTCCCAG--AATTTGGGA
TGAATGGGAAATTTTCAACAACACATTTATTGGCATGTGGAT
>Mfa
CATAATGTCACACAGCATGAACAGACTTTCCGATGGAATGCCTACAGTGGAATTTTGGG-
-GTGAGCTCGCTGCTCTCGGCTTCCTTCCTCCCCT-CCCTTTTTAAAGAACCTCTACTGT
TCCCAAAACTCAGTCGATTCTT-----------------------------------GT
TCAGGGTAAATGATGGGGTG-------AACCTACAAACTGCCTTCTGGCAGAAAGAA-GC
TGTTTTGCC-----ATCCCCTCTCCCCA----------TTCCAACCCA------------

---TTTTGGAGATGG---AAAA
CTAGA---AAGAAAAGAAAAA---CC----------TTTTTTT-----AACTGTAAGATG
GGGTAA-TAATATCATAGTGCTT--ATTACTTTACAGGGTTGTCATGAGGCTGGCTCAGA
T-GAGATAATTTATGTATA-----GCAGTTTGCAAATTTTCT-------ATTACCTGTA-
-TGTCCATTATTATTGAGGGA---AACAAATGA-AG--ATT-AGAAAGGACTTTAA--TC
--TTCCCAGCTA---ATTCTC-----TGGGCAG--------------------AATACTA
GGCCTGCAGCCAGGAAGAGCTAACTGCAAATCAAATCTGGCCTCAGACACTTACTATGTG
ATCTGGGCCAAG--------TTA-----------CCTAATCC-ACTAGAGAAGGAAATG
ATAAACCACACCAGTATCTTTGCCAAGAAAACTC--TATGGAGAG--GGGCCCACAGAGT
AAT---------GAAGTTAGATGAGA--TTGAATGGCAAAGTAGCTAATCCCCTCACTTA
AAGATAAGAC---------------------AGAAGAGAATT-----------CATTTGC
TCAAGGCTGTAGTATTCTATCATACTGTCTTTTT-GGTATAAGCCTGGTTATAAGGCTTT
TGAGATGT---TTTAAAA-GGCTTTTTTTAAGC--GTTACCTTTT-CTGGGTAAAATTTT
AGCTCTGCTGTTTTGGGGATGTCATTCATTGATACCTTG----TATTAGACATAATTCAT
TTTGATGACTC--AGTTTTTCATGGATTATGTGTAGCTTAATAATTA-----ATGC-AAG
TAA-----TTCTTGA----GTAAA-----TTGTATTTATTTTTTCCCAG--GATTTGGGA
TGAATGGGAAATTTTCAGCAACACATTTACTGGCATGTGGAT
>Pgu
CACAATGTCACGCAGCACGAACAGACGTTCCGTTGGAATGCCTACAGTGGAATTCTGGG-
-GTATTGAGC----------------------CTTCTTTTTTTGAAAGAACCTTGATCAT
TCCCAAAACATGG-GATGTATT-----------------------------------CT
TCAGGGTAATAAATA-GGTG-------ATCCCAAAACCTAACAATTGGCCAGAAGAA-GC
TGCCTGCTG-----CCCTCTGTTGTCCCCTTATAATTCCCCCAACCCA------------

---TTCTAGAGATGGGG-AAAA
CTAGG---AAGAAAAACAA-----CTA---------TTTT--------ATCTGTAAAATG
GAGGTAATAATTTACCACT------GTTATTGAGGAACACCAGTGAGA-GCTAAAAAAGA
T-TTTTAAGAAAAAAATTAA------------GCTAATTCCCT---GGGCTGGACAGGCT-
---------------AAGCCA---GG--AATCA-GG----A-AGATTGGACTTCAGA-TC
CAACCTCAGGC----ATTTAC--------------------------------TATAACTA
TG--TGATCTTGGG------------CAAATC----------------------------

------------------ACTTAA--CTTTTGTTGCCTAATCCATTGCAAGAAGAAAATG
ACAACCCATTCCAGTATTTTTGCCAAGAAAGCCC--CATTGAATG---AACCCATGGTTC
ATTGGGTGGTGAAGAGTTGGAGAAGTTATTTAATAACAAA---TCTAATCCTTTCACTTA
GAGAAAGCTG-CG---AGT-CTT--------AGAAGAGAATT-----------GTTTTGC
CCAAGGCTGCAGTGTTCTACCATACTGCTTTTTG-GAATGAACC-TAGATGTAAGGCTCT
TGGAATAT--GTTAAATA-----GAATTTAAGT--ATCATCTTTT-CTGGGTAAATTTTT
AGCAATTA-----TGGCTGAGTTATTCATTGGTAGATTA----TGTCAGAC--AACTCAT
TTTGGTGACCCCTAGTTTT-CATGGATTA-----------------------GTCT-AAG
TGTT-GGTTTCTTAAGTA-GTAA--TGGT-TATTTTTATTCTTTCCTAG--GATTTGGGA
TGAATGGGAAATTTTCAACAACACATTTACTGGCATGTGGAT
>Iso
CACAATGTCACACAGCACGAACAGACGTTCCGTTGGAATGCCTACAGTGGAATTCTGGG-
-GTAAGCTCACTGGCTA---------TTGAGCCTTCTTTTTTTGAAAGAACCCTGATCAT
TCCCAAAACATGG-GATGTATT------------------------------------CT
TCGGGTTAATAAATA-GGTG-------ATCCCAAAACCTAACAATTGGCCAGAAGAA-GC
TGCTTGCTG-----CCCTCTGCTGTCCCCTTATAATTCCCCCAACCCA------------

---TTCTGGAGATGGGGGAAAA
CTAGG---AAGAAAAACAA-----CTA---------TTTT--------ATCTGTAAAATG
GGGGTAATAATTTACCACT------GTTATTGAGGAACACCAGTGAGA-GCTAAAAAAGA
T-TTTTAAGAAAAAATTAA------------GCTAATTCCCT---GGGCTGGACAGGCTC
AGTGGATAGAACACTAAGCCA---GG--AATCA-GG----A-AGATTGGACTTCAGA-TC
CAACCTCAGGC----ATTTAC-------------------------------TATAACTA
TG--TGATCTTGGG-------------CAAATT---------------------------
------------------ACTTAA--CTTTTGTTGCCTAATCCATTGCAAGAAGAAAATG
ACAACCCATTCCAGTATTTTTGCCAAGAAAGCCC--CATTGAATG---AACCCATGGTTC
ATTGGGTGGTGAAGAGTTGGAGAAGTTATTTAATAACAACAAATCTAATCCTTTCACTTA
GAGAAAGCTG-TG---AGT-CTT--------AGAAGAGAATT-----------GTTTTGC
CCAAGGCTGCAGTGTTCTACCATACTGCTTTTTG-GAATGAGCC-TAGATGTAAGGCTCT
TGGAATAT--GTTAAATA-----GAATTTAAGT--ATCATCTTTT-CTGGGAAAATTTTT
AGCAATTA-----TGGCTGAGTTATTCATTGGTAGATTA----TGTCAGAC--AACTCAT
TTTGGTGATCCCTACTTTT-CATGGATTA-----------------------GTCT-AAG
TGTT--GTTTTTTAAGTA-GTAA--TGGT-TATTTTTATTCTTTCCTAG--GATTTGGGA
TGAATGGGAAATTTTCAACAACACATTTACTGGCATGTGGAT
>Tro
------------------------------------AATGCTTACAGTGGAATTCTAGG-
-GTGAGCTTATTGGCTC------------TTAGT-CTTTTTTTGAAAGAACCTATACCAT
TCTCCAAATTCGGAAGATTCTT------------------------------------GT
TCAGGATAAATGGTGGGGTG-------ATTCTACAGACTAAC-TCTGAGAGTAAGAA-GT
GACCTCCCC-----TCC--------------------CCCCGAACTAA------------

---TTTTGTACAAGG---AAAA
CGAGA---AA--------------CTTTT-------TTTTTTT-----------GAAATG
GGAATAATAATATAGTG--------GTTACTTCATAGGGTTG-TATGAGGCTG-CTCAGA
T-GAGATAATGTATGTAAA-----GCATTT--CAAACCCTGT-----ATTATGAA-----
-TGTCCAATGTTACTGAAGGA---AACCAATGA-GA--GTG-AAAAAGAATTTTAAAGTC
--AATT-AGGTA---GTTTTC-----TGGGCAGCTAGACTGCTCAGTGGATAGAATACTA
GGCCTGGAGCTAGGAAGACCTGAGTTAA--------------------------------
-----------GCCGTTACCTTAAAGCTCTCTCTGCCTAATGT-GCTGGAGAAGAAAATA
GCAACCTAT-GGACAAGTATGG--------TCCC--CAGGAATCAT--------------
----------------------------------GACAAGCTGGACAATCCCCTCACTTA

GAGAAAGACAAGG---TAT-CTT--------AGAAGAG--------------------

---TAAATTTTT
AGCC-------------------GTTCACTGGGAGCTCA----TGTTAAACATAATTCAC
TTTGTGGACTTTCCGTTTT-CATGGACTG----------------------GTTT-AAG
TATT--GCTTCTTGAGTA-GTAA--TGGGTTAAATTTATTCTTTCCCAG--GATTTGGGA
TGAATGGGAAATTTTCAACAATACATTTACTGGCATGTGGAT
>Ppe
CACAACGTCACACAGCATGAACAGACTTTCCGTTGGAATGCTTACAGTGGAATTCTAGG-
-GTGAGCTCACTGGCTA---ATAGTCTTCTCTTTTGAAAGCACAAAAGAACATCCACCAT
TCCCCAAACTTGGTAGATTCTTGAAACCTCCACCATTTCCCAAACTCAGTAGATTCTTGT
TCAGGATAAATTGT-GGGTG-------ATCCTATAGACTAACTTCTGAGAGTAAGAA---
-GCTAA--------CTCCTTACTCCCCA---------TCCCCAACCCA-----------

--CTTTGTACATGG---AAAA
CTAGG---AAGAAGGAAA------CCTTT-------TTTTAA------ATCTGTAAAATG
GGAATAACAATACAATG--------TTTACTTCATAGGGCTGTTATGAGGCTG-CTCAGA
T-GAGATAATGTATATAAA-----GCATTTTGCAAACCTTGT-----ATTATGCA-----
-TGTCCAGAGTTATTGAGGGA---AACCAATGAAGA--GTT-AAAAAGGATTTTAAAGTC
--AATTCAGCTA---ATTCTC-----TGGGCAGCTAGATGGCTCAGTGAATAGAACAGTA
GGCCTGGAGTTAGGAAGACCTGAGTTAAAATC-----TGGTCGCGGACATTTACAAGCTA
TATTACCTTAAGCANGTTATTTAAC-CTCTCTTTGCCTAATCT-GCTGTAGAAGAAAATG
GCAACCCATTCCAGTATCTTTGCCAAGAAAACCCC-CACGGACAATATGGTCCACAGGGT
CATGACAGGCTGGACATGACTG------------AACAAGGCC---AATCCC-TCACTTA
GACTTAGAAG--------------------AGAATTG---------------ACTCGT
CCAGGGCTTCAGTGTTCTACCGTACTGTCTTGCT-GATGTGAGTCAAGATATAGGGCTCT
CGAGATAT--GTTAAAAAG----GAATTTAAGT--ATCATCTTTT-CTAAGTAAATGTTT
AGCGATTGTGTTTTGGCTGAGTTGTTCGTTGGCAGCTCA----CATTAGACATAACTCAT
TTTGCTGACTCTTAGTTTT-CATGGATTA----------------------GT------
------GCTTCTTGAGTA-GTAA--TGGGTAATATTTATTCTTTCCCAG--GATTTGGGA
TGAATGGGAAATTTTCAACAATACTTTTACTGGCATGTGGAT
>Tvu
CACAATGTCACACAGCATGAACAGACTTTCCGTTGGAATGCCTACAGTGGAATTCTGGG-
-GTGAGCTTAGTGGCTC--------------------TTAATCTAAGAACCTCCACCAT
GCCCCAAACTCGGTAGATTTTT-----------------------------------GT
TCAGGATGAATGGTGGGGTG-------ATCCTACAGACTAACTTCTGACAGTAAGAA-GG
CCTGCTGCC-----TCGTT-----------------CCCCCAACCCA-----------

--TTTTGTATATGG---AAAA
CTAGA---AA--------------C-----------TTTTT-------ATCTGTAAA-TG
GGGATGATAATATTGTAGCA-----CTTATTCCATAGGTCTGTCATAAGGCGG-CTCAGA
T-GAGATAACAGATGTAAA-----GCATTTTGCAAACCTTGT-----ATTATACAG----
-TGTCCATTGTTATTGAGGGA---AACCAATGA-GA--G---AAAAAGGATTTTAAAGTC
--AACCCAGCTA---ATTTCG-----TGGACAGCTAGATGGTTCAGTGAATAGAACACTA
GGCCCGGAATCAGGAAGAACGGAGTTAAAATC-----CGGTCTCAGACATTTACTAGCTG
TGTGACTTTGAGCAAGTCATTTAACCACCTCTTTGCCTAATCCAATTGGAGAAGAAAATG
GCAACCTGTTCCAGTATCTTTGCCAAGAAAATTC--CACAAACAGTACAG--CTCAGAGT
CATT-----------AAGAGTT----GGGCATGACTGAACAACTG--------TCGCTTA
GAGAAAGACAGTG---AGT-CTT--------AGAAGAGAATT-----------GATTTGT
CCAAGCCTGCAGTATTCTGTCATATTGTCTTTTT-GATATGAGCCTAGATAGAGA----T
TAAGATAG--GTTAAAA-G----GAATTTAAGC--ATCACCTTTT-CTGGGCAAATTTTT

```
AGCAATTATTTTTTGGCTGAGTCGGTCATTGGTAGCTCA----TGTTAGACATAACTCAT
TTTGATAACTCTTGGTTTT-CATGGATTA-----------------------GTGT-AAG
TATT--GTATCTTGAGTA-GTAA--TGGGTTATATTTATTCTTTCCCAG--GATTTGGGA
TGAATGGGAAATTTTCAACAATACATTTACTGGCATGTGGAT
>Mro
CACAACGTCACACAGCATGAACAGACTTTCCGTTGGAATGCCTACAGTGGAATTCTGGG-
-GTGAGCTCACTGGCTA-----------TTAGTCTTCTTTTTTGAAAGAACCCCCATCAT
TCCCCAAACTAGGTAGATTCTT------------------------------------GT
TCAAGATAAATGGTAGGGTG-------ATCATACAAAC----TTCTGACAGTAAGAA-GC
TGCCTTCTT-----CCCCACACCTCCAA---------CCCTCAACCCA------------
------------------------------------------------------------
------------------------------------------------------------
-----------------------------------------TTATGTACACAG---AAAA
CTAGC---AA--------------C-----------TTTTTT------ATTTGNAAAATA
GGGATAATAATAGCATAGTG-----TTTACTTCATAGGACTGNCATGAGGCTG-CTTANA
T-GGTGTAATGTATGTAAAA----NCATTTAGCAAACCTGGT-----ATTATACA-----
-TGTCCAGTGTTATTGAGGGA---AACCAATGA-AA--GCT-AAAAAGGATTTTAAAGTC
--AATTCAGCTA---ATTCTC-----AGGGCAGCTAGATGGCTCAGTG----------GA
GGCCTGGAGTCAGGAAGACCTGAGTTAAAATC-----TGGTCTCAGACATTTATTAGCTG
TGTCA--------------TTTAACC-TCTCTTTGCCTAATCT-ACTGGAGAAGGAAATG
GCAACCCATTCCACTATCTTTGCCAAGAAAACCC--TATGGAAGATATGGCCCACAGAGT
TGGACATCACTG----------------------AACAACAAGGCTAATCCCCTCACTTA
GAGAAAGACAGTA---AGT-CTT--------AGAAGAAAGAAAATC-------GATTTGT
CCAAGGCTGCAGTGTTCTGCCATACTGTGTTTTT-GGTATGAGCCTAGATATAGGGCCCT
TGAGATAT--GTTAAAAAG----GAATTTAAGC--ATCACCTTTTTTTGGGTAAATTTTT
AGCAATTATGTTTTTGCTGAGTTGTTCATTGGTAGTTCA----TGTTAGACGTAACTCAT
TTTTGTGACTC-----TTT-CATGGATTA-----------------------GTGT-AAG
TA---------------------------TTATATTTATTCTTTCCCAG--GATTTGGGA
TGAATGGGAAATTTTCAACAATACATTTACTGGCATGTGGAT
>Ptr
CACAATGTCACACAGCATGAACAGACTTTCCGTTGGAATGCCTACAGTGGAATTCTGGG-
-GTGAGCTCACTGGCTA-----------TTAGTCTTCTTTTTTGGAAGAACCCCTATCAT
TCCTCAAACTAGGTAGATTCTT------------------------------------GT
TCAAGTTAAATGGTAGGGTG-------ATCCTACAGACAAACTTCTGACAGCAAGAA-GC
TGCCTTCTT-----CTCCGCACCTCCAA---------CCTTCAACTCA------------
------------------------------------------------------------
------------------------------------------------------------
-----------------------------------------TTTTGTACACAG---GAAA
CTAGA---AA--------------C-----------TTTTTT-----AATCTGTAAAACA
GGGATAATGAGAGCATACTG-----CTTACTTCACAGGGCTGTCATGAGGCTG-CTTAGA
T-GATGTAATGTGTGTAAAAA----CATGTAGCAAACCTTGT-----ATGATACA-----
-TGTCCAGTGTTACCGAGGGA---AACCAATGA-GA--GCT-AAAAGGGATTTTAAAGTC
--AATTCAGCTA---ATTCTC-----TGGGCAGCTAGATGGCTCAGTG----------GA
GGCCTGGAGTCAGGAAGACCTGAGTTAAAATC-----TGGTCTCAGGCATTTATTAGCTG
TGTTACCTTCAGCAAGTCATTTAACC-TCTCTTTCCCTAATCT-GCTGGAGAAGGAAATG
GCAACCCATTCCACTGTCTTTGCCAAGAAAACCC--TATGGACGATATGGCCCACAGAGT
TGGGCATCACTG----------------------AACAACANGGCTAATCCCCTCNCTT-
ANANAAGACAGNG---AGT-CTA---------AAAAAAATTGATTG--------------
------------------------------------------------------------
------------------------------------------------------------
------------------------------------------------------------
------------------------------------------------------------
------------------------------------------------------------
```

MARKER 155

```
>Mar1b_Mdo
------------------------------------------------------------
---------------------------------------TTGTTGTTGTTGTTCAGTCGT
TTTCAGTCATGTCTGACTCTTCGTGACCCCA--TTTGGGGTT------------------
----------------TTCTTGGCAAAGATACTGG---------------------AGTG
GTTTGCCATTTCCTTCTCCAGCTCATTTTA------------CAGATGAGGAAA-CTGAG
GCAAACAG-GGTTAAGTGACTTGCCCA----GGGTCACACAGCTAGTAA-----------
---GTGTCTGAGGCCAGATTTGAACTCAG--------GTCTTCCTGACTCCAG--GCCCG
GC-----GCTCTATCCACTGTGCC-ACCTAGCTGCCCT----------------------
------------------------------------------------------------
------------------------------------------------------------
------------------------------------------------------------
------------------------------------------------------------
------------------------------------------------------------
------------------------------------------------------------
------------------------------------------------------------
-------------------------------------------------
>Mdo
CTCAGAGAATGCAGCCCTGGTACGGGAACAGAATGGCCATCAGTTACTAGTGGCCCTGGT
TGGGGATAGCCTGCTTGAG--GTGAGTATGAAGAAA------------------------
------------------------------------------------------------
------------------------------------------------------------
------------------------------------------------------------
------------------------------------------------------------
------------------------------------------------------------
---------------------------------------------------------AGA
GGGTGTCATTTT-AGAGGGGAGGGATGTATTAATGTTAGAGTATCTGCTA--------CC
AAAAGAGCCACAGCTAGACTTTGT-TTAAATATATCATTGTTATTT-ACTATAGCTAAAC
TCCA--------------------------------------------------------
------------------------------------------------------------
-----------------------------------------TAGAATGTCATGCTCTTTA
GAGTTCATTTTCCCCCTTTAACTTATTTTGCTCTTTTTCTCTTCTTCTTC--ACATCC--
-----------CCCA-CCCAAA------TTTTTATGTAG--CCTTTTTGGCCAATGGGAA
CTGGAATAGCCCGAGGTTTTCTGGCTGCTATGGACTCCGCCTGGATGGT
>Dvi
CTCAGAGAATGCAGCCATGGTACGGGAACAGAATGGCCATCGGTTACTGGTGGCCCTGGT
TGGGGATAGCCTGCTTGAG--GTGAGTATGAAGGGA------------------------
------------------------------------------------------------
------------------------------------------------------------
------------------------------------------------------------
------------------------------------------------------------
------------------------------------------------------------
---------------------------------------------------------AAA
GGGTATAATTTT-AGAGGAGGGGAGAAATGTAATAAT--GTTATCTGT-----------C
AAATGAGCCATAGCTGGACATTTT-TTAAATATGCCATT---------------------
------------------------------------------------------------
------------------------------------------------------------
-----------------------------------------GATAATGTCATGTTCTTTA
--GTTCATTTTCCCCCTTTAACTTAT------CTTTTTCTCTTCTTC-----ACTTCCT-
-----------CCCACCCCAAAAAA---CTATTATGTAG--CCTTTTTGGCCAATGGGAA
CTGGAATAGCCCGAGGCTTTCTAGCTGCTATGGATTCAGCCTGGATGGT
```

>Mnu
CTCAGAGAATGCAGCCATGGTACGGGAACAGAATGGCCATCGGTTACTGGTGGCCCTGGT
TGGGGATAGCCTGCTTGAG--GTGAGTATGAAGGGA------------------------

---AAA
GGGTATAATTTT-AGAGGAGGGGAGAAATGTAATAAT--GTTATCTGT-----------C
AAATGAGCCATAGCTGGACATTTT-TTAAATATGCCATT---------------------

---GATAATGTCATGTTCTTTA
--GTTCATTTT---------------------------CTCTTCTTCTTC--ACTTCCC-
-----------CCCACCCCAAAAAA---CTTTTATGTAG--CCTTTTTGGCCAATGGGAA
CTGGAATAGCCCGAGGCTTTCTAGCTGCTATGGATTCAGCCTGGATGGT
>Rra
ATCAGAGAATGCAGCCCTGGTTCGAGAGCAGAATGGACACCAGCTATTGGTGGCCTTGGT
TGGAGATAGCCTGCTTGAG--GTAAGTGTGAAGGAA------------------------

---AAG
GAGTGATATTTTTATAGGGGAGGAATTTCATAC------TATATCTGATATTAT-----G
AAATGAGGCAT-AGGAGAGTTTTTATATTTCATTGTTT----------------------

---------------------ATTATAACGAAACATCT-AGCAAAATGCCATTT------
--------CTTCCCTTTAAAACTTATTTACTTTTTTTTCTCTCTT-----GCACTCCCTC
CACACCCATACT---CTCCAAAAA----CTTTTATGTAG--CCTTTTTGGCCAATGGGAA
CTGGAATAGCCCGAGGATTTCTGGCTGCCATGGACTCAGCCTGGATGGT
>Cfu
ATCAGAGAATGCAGCCCTGGTTCGAGAACAGAATGGACACCAGCTGTTGGTGGCCTTGGT
TGGAGATAGCCTGCTTGAG--GTAAGTGTGAAGGAA------------------------

---AAG
GAGTGATATTTTTACAGGGGAGGAATTTCATAC------TTTATCTGATATTAT------
GTT--AGATATGAGGAGAGTTTTTATATTTCATTGTTT----------------------

---------------------ATTATAACTAAACATCT-AGCAT----------------
--------CTTTCCTTT--AACTTATTTACTTTTTTTTCTCTCTT---GTGC--TCCCTC
CACACCCACACT---CTCCAAAAA----CTTTTATGTAG--CCTTTTTGGCCAATGGGAA
CTGGAATAGCCCGAGGATTTCTGGCTGCCATGGACTCAGCCTGGATGGT
>Dgl
ATCAGAGAATGCTGCCCTGGTTCGGGAACAGAACGGTCACCAGTTATTGGTGGCTCTGGT

TGGGGATAGCCTGCTTGAG--GTGAGTATGAAGGAA-----GTTCTTGTT----------
--CAGTCGTGTCCAATACTTAA-----GTGG-----------------------------

--------TTTCCTTCTCCAGCTCATTTTA------------CAGATGAGAAAA-CTGAG
GCAAACAGAGGTTAAGTGACTTGCCCA----GGGTCACACAGCTAATAA-----------
--GTATCTG-AGGCTGCATTTGAACTCA-----------------TTTTCCTGACTCTAG
GCCTGGTGCTCTATCCACTACACC-ACCTAGCTGCCC-----AATATGAAGGAA---AGG
GAATGATATCTT-ATAGGGGAGTTATGTATCTGATACTACAGAATGAGCCATA-------
GTTAGAGATATGAGTGGAGTTTTATATATCATTGTTATTT--------------------

---------------------ACTACAGCTAAACTTCT-AGCAGAATGCCGTGGTTTTTT
--GTTCTTTTTCTCCCTTTAACTGATTTTAC----TTTCTCTCTT---CTGCACTTCCCC
--------TACTG-ACCCCAAAAAA--TCTTCTATGTAG--CCTTTTTGGNCGATGGGAA
CCGGAATAGCCCGAGGCTTTCTGGCTGCTATGGACTCAGCCTGGNTGGT
>Nty
ATCAGAAAATGCAGCCCTAGTTCGGGAACAAAATGGTCATCAGTTATTGGTGGCCCTGGT
TGGGGATAGCCTGCTTGAG--GTGAGTATGAAGGAA-----GTTGTTGTT----------
--CATTTCCGTCTCAACTCTTCATGACTCCA--TTTGGGGTTGGTTGTTTTTTTTGTTTT
TTGTTTTTTGTTTTTTTTTTTGGCAAAGATTTTGGCAAAGATTTGGCAAAAAATGGCAAA
GTTTGCCATTTCCTTTTCTAGCTCATTTTA------------CAGTTGAAGAAA-CTGAG
GCAAACAG-GGTTAAGTGACTTGCCCA----GGGTCACACAGCTAT--------------
--GTTTCTG-AGTCTGGATTTAAACTCATGAAGATGAATCTCTCTTATTCTTG--GCCTG
GC-----ACTCTATCCACTGTGCC-ATCTAGCTGCCC----GAGTCTGAAGGAA---GAG
GAATGATATTTT-ATAGGATGATAATGTATCTGATACTGTGAG-------ATGGGCCATA
GTTAGAGATATGAGTGGAGTTTTATATGTCA--TATATTT--------------------

---------------------ACTATAGCTAAACTTCC-AGCAGAATGTAATGCTTTTTT
------GTTTTCCCCTTTTAACTTATTTTAC----TTTCTCTCTTTTTCTCTATTTCCCC
TCACCCCATACTC-ACCCAAAAAA---CCTTCTATACAG--CCTTTTTGGCCAATGGGAA
CTGGAATAGCCCGAGGCTTTCTGGCTGCTATGGACTCAGCTTGGATGGT
>Pta
ATCAGAGAATGCAGCCCTGGTTCGGGAACAGAACGGCCATCAGTTATTGGTGGCCCTGGT
TGGGGATAGCCTACTTGAG--GTAAGCCTGAAGGAA-----ATTGATGTT----------
---TATTATGTCTAAT--------GACCTCA--TCTTTTTTC------------------
----------------TTTCTTTTTTTTTTATG---------------------------
----GCTATTTCCTTCTTCAGCTCATTTTA------------CAGATGA-GAAA-CTGAG
GCAAATAG-GCTTAAGTGATTTGCTCA----GAATTAAAT--------------------
----------------GATTTTACTTGAAG---ATGAATCTTTTTTATTCTAA--GCTTG
GCACTCTGTTCACTTAACTGCATC------------------ATTATGAAGGAA---GGG
GAATAACATTTT-ATAGGGCATACTATAGAATGA-GCCATA-------------------
------------------GTTATTCATAGATACGTATAGCTATATA-GTTAGATC-ATAG
TTAGAGATATGAAGTTTTATTTATCA----------------------------------

----------------------TAATAGCTAAATTTGCCAACAAAATGTCATGGGTTTTT
-GGTTCATTTTCCCTTTTTAACTTATTTTCC----TTTCTCTCTTTTTCTGTATTTT-GC
TCACCCTCTACT-CGCCCAAAAAA---CCTTTTATGCAG--CCTTTTTGGCCAATGGGAA
CTGGAATAGCCCGAGGCTTTCTAGCTGCTATGGACTCAGCCTGGATGGT
>Dgo
ATCAGAGAATGCAGCCCTGGTTCGGGAAAAGAACGGCCATCGGTTATTGGTGGCTCTGGT
TGGGGATAGCCTACTTGAG--GTAAGCCTGAAGGAA-----ATTGATGTT----------
---TATCAGGTCTAAT--------GACCTCA--TCTGTGATCTTTTTTT-----------

----------------TT--
-------------------------TTTTT------------GCTATGA-GAAA-CTGAG
CCAAATAG-ACTTAAGTGAATTGCTCA----GAGTCAAATAGCTATTAA-----------
--GTGTGTG-AGCATGGATTTTACTTGACG---ATGAATCTTTTTCATTTTAT-----AG
GG----TATACTATAGAATGAGCC-ATA--------------GTTATTCATAGA------
-------TATGT-ATAGCTATATAGTTAGA-----TCCATAGTTAGAGATAAGAA-----
------------------GTTTTTATTTATCATAATA-----------------------

---------------------------GCTAAATTTCTT---------------------
--GTCCATTTTCCCTTTTTAACTTATTTTCC----TTTCTCTCTTTTTCTGTACTTT-GC
TCACCCCCTACT-TGCCCAAAAAA---CCTTTTATACAG--CCTTTTTGGCCAATGGGAA
CTGGAATAGCCCGAGGATTTCTAGCTGCTATGGACTCAGCCTGGANGGT
>Scr
ATCAGAGAATGCAGCCCTGGTTCGGGAACAGAACGGCCATCAGTTATTGGTGGCCCTGGT
TGGGGATAGCCTACTTGAG--GTAAGCCTGAAGGAA-----ATTGATGTT----------
---TAGTGTGTCTAAT--------GACCCCA--TTTGTGATC------------------
----------------TTTCTTTTA-----------------------------------
TTTGGCTATTTCCTTCTTCAGCTCATTTTA------------CAGATGA-GAAA-CTAAG
GCAAATAA-GCTTAGGTGAATTGCTCA----GAGTCAAAAAGCTATTAAATGTCTTAATA
AAGTGTCTTAAGGCTGGATTTTAATTGAAA---ATGAATCTTTTTTATTCTAA--GCCTG
GA-------ACTCTTAACTGTCTC-------------------ACTATGAAGGAA---GGA
GAATTGCATTTT-ATAGCATATACTATAGAATGA--------------------------
----------TATATATAGTTTTTATTTATCATGTATAGCTATATA-GTTAGATCCATAG
TTGGAGATGTGAAGTTTTTACTTACTTACTT----ACTTACTTATTTATTTATCATAATA
GCTAAAATTTATTTTATTATAATATAATTATAATTATTATTTTAAAATTTATTTTATTGT
AATAGCTAAATTTATTTATTATAAATAGCTAAATTTG-AAGCAGAATGTCATGCTTTTTT
--GTTCATTTTCCTCTTTTAACTTATTTTAC----TTTCTCTCTTTTTCTGTACTTT-GC
TCACCCCCTACT-CACCCAAAAAAAA-CCTTTTATGCAG--CCTTTTTGGCCAATGGGAA
CTGGAATAGCCCGAGGCTTTCTAGCTGCTATGGACTCAGCCTGGATGGT
>Mfa
ATCAGAGAATGCAGCCCTGGTTCGGGAACAGAACGGCCATCAGTTATTGGTGGCCCTGGT
CGGGGATAGCCTACTTGAG--GTAAGTCTGAAGGAA-----ATTGATGTT----------
---CATCATGTTTTAT--------GACCACA--AATTTTATC------------------
----------------TTATGGCAAAGATAT-GAG--------------------AGCA-
GTTTGCCATTTCCCTCTCCAGCTTATTTTA------------CAGATGA-GAAA-CTGAG
----------------TGACTTGCTCA----GAGTCAAATAGCTATTAA-----------
--GTGTCTG-AGACTGGATTTGAACTGATGAAAATGAATCTTTT-GATTCTAA--GCCTG
GAACTCTCTTCATTTGCCACTTAGCTACCTC-----------ACTATGAAGGAA---AGG
GAATGACATTTT-ATAGGGTATACTATAGCATGA-GCCATAAGTAGAGATA---------
----------TGAATAAAGTTTTTATTTATCATGTATAGCTATAATAGTTAGATCCATAG
TTAGAGATATGAAATTTTTATTTATCACA-------------------------------

-----------------------TATAGCTAAACTTCC-AGCAGAATGTCATGCTTTTTT
-GGTTCATTTTCCCCTTTTAACTTATTTCAC----TTTCTCTCTTTTTCTGTACTTT-GC
TCACTCCCTATT-CACCAAAAAAA---CCTTTTATGCAG--CCTTTTTGGCCAATGGGAA
CTGGAATAGCCCGAGGCTTTCTAGCTGCTATGGACTCAGCCTGGATGGT
>Bil
ATCAGAAAATGCAGCCCTGGTTCGGGAACAGAATGGTCATCAGTTATTGGTGGCTTTGGT
TGGGGATAGCTTACTTGAG--GTGAGTATGAAGGAA-----TTTGTTGTT----CAGTTG
AACAACAAGTGTCCATCTCT--
----------------TTTGGACAAAGATATTGAG----------------------AG
GTCTCCCATTTCTTTTTTCAGCTCATTTCAGCTCATTCAGCTCAGATGAAGAAACTTGAG

```
TTAAACAG-TGTTAAGTGATTTATTTACCTACAGTCATAGAACTAGTGTAA---------
--GTTTCTT-AGGCCAGATTTT-ACCCTTGAAGATGAGACCTATCTACTGTAC-------
-------------------------CCCTACTGCCT-----GAGTGTGAAGTAA---GGG
GAATGATATTTT-ATAGGAGAGTAATTTATGTGATACTATAGAATGACATA---------
----------TGAGTAA-GTTTTTACTTGTCATGTGTC----------------------
------------------------------------------------------------
------------------------------------------------------------
--------------------------------ACTT---AACAGAATGTCATGCTTTTTC
--ATTCATTTTCCTCTTTTAACTTATTTTATC-TTTCTCTCTCTCTTTCTGCACTTC-CC
CCATCCGTTACT-TACTCCCCTAAAAA-CTTTTTTGCAG--CCTTTTTGGCCAATGGGAA
CTGGAATAGCCCGAGGCTTTCTGGCTGCTATGGATTCAGCCTGGATGGT
>Pgu
ATCAGAAAATGCAGCCCTTGTTCGGGAACAGCATGGTCATCAGTTANTGGTGGCTTTGGT
TGGGGATAGCTTGCTTGAG--GTGAGTATGAAGGAA-----TTTGTTGTT----------
---CAATTGTGTCCAACTCTTCTAGGCAAAG--ATATT----------------------
----------------------------------G---------------------GGAG
GGTTCCCATTTCCTTTTTCAGCTCATTTTA------------CAGATGA-GAAA-TTGAG
TTAAACAG-GGTTAAGTGT-----------------------------------------
--GTTTCTT-AGGCCAGATTTG-ACCCATGAAGATGAGACCTATCCACTATAC-------
-------------------------CCCCAACTGCCT----GAGTGTGAAGGA-------
---GAATATTTT-ATAGGAGAGTAATTTATCTGATACTATAGAATAAGATA---------
----------TGAGTAA-GTTTTTATATATCATGTATC----------------------
------------------------------------------------------------
------------------------------------------------------------
--------------------------------ACTG---AACAGAATGTCATGCTTTTTC
--ATTCATTTTTCTCCTTTAATTTATTTTATC---TTTCTC--TTTTTCTGCACTTC-CC
ACATTCCTTAC----------------CCTTCTTTGCAG--CCTTTTTGGCCAATGGGAA
CTGGAATAGCCCGAGGCTTTCTGGCTGCTATGGATTCAGCCTGGATGGT
>Iso
ATCAGAAAATGCAGCCCTTGTTCGGGAACAGCATGGTCATCAGTTATTGGTGGCTTTGGT
TGGGGATAGCTTGCTTGAG--GTGAGTATGAAGGAA-----TTTGTTGTT----------
---CAATTGTGTCCAACTCTTCTA------------------------------------
--------------------GGCAAAGATATTGCG-----------------------AG
GTTTCCCATTTCCTTTTTCAGCTCATTTTA------------CAGATGAAGAAA-TTGAG
TTAAACAG-GGTTAAGTGT-----------------------------------------
--GTTTCTT-AGGCCAGATTTG-ACCCATGAAGATGAGACCTATCCACTATAC-------
-------------------------CCCCAACTGCCT----GAGTGTGAAGGA-------
---GAATATTTT-ATAGGAGAGTAATTTATCTGATACTATAGAATGAGATA---------
----------TGAGTAA-GTTTTTATATATCATG-TATCAC-------------------
------------------------------------------------------------
------------------------------------------------------------
-------------------------------------TGAACAGAATGTCATGCTTTTTC
--ATTCATTTTTCTCCTTTAATTTATTTTATC---TTTCTC-TTTTTTCTGCACTTC-CC
ACATTCCTTAC----------------CCTTCTTTGCAG--CCTTTTTGGCCAATGGGAA
CTGGAATAGCCCGAGGCTTTCTGGCTGCTATGGATTCAGCCTGGATGGT
>Tro
ATCAGAGAATGCAGCCCTGGTCCGGGAACAGAATGGGCATCAGTTACTGGTGGCCCTGGT
TGGGGATAGCCTGCTTGAG--GTGAGTACGAAGGAA------------------------
---------------ACTACTCGTGACCCCGTTTTTGGGTTCT-----------------
----------------TCTTGGCAGAGGTATCAG----------------------GGTG
GTTTGCCATTTCCTTCTCCAGCTCATTTTA------------CAGATGAAGAAA-TTGAG
ACAAACAG-GGTTAAGTAATTTGCCCA----GCGTCACACAGCTATAAGTAA--------
--GTGTCTG-AGGCCAGATTTGAACTCAGGAAGCTGAACCTTCCTGATTCTAG-GCCAGG
```

CATTTTATCCACTGGGCC-------ACCTAGCTGCCC----AAGTATGAAGAAA---GGG
AATTGATGTTTT-ATAGGGGAGTAATGTGCCTGATACTACAGAATGAGCTATCGTTAG--
----AGATAGGAGTGAAATTTTTAATATATCA--TATTCAC-------------------

-----------------------TATAACTAAAACTTACAGCAGAACGTCATGCTTCTTC
--ATTCCTTTTACCTTTTTAACTTATTTTACT-TTTTTCTCTCATTTTCTGCACTTC-CC
C-ACCCTCACCTC-CCTCCCCCAAAA-CCTTTTATGCAG--CCTTTTTGGCCAATGGGAA
CTGGAATAGCCCGCGGTTTTCTGGCTGCTATGGACTCAGCCTGGATGGT
>Ppe
ATCGGAGAATGCAGCCCTGGTTCGGGAACAGAATGGTCACCAGTTATTGGTGGCCCTGGT
TGGGGACAGCCTGCTTGAG--GTGAGTGTGAAGGAA-----GCTGTTGTT----------
---CAGTCATACCCAATTCTTCGTGACCCCA--TTTG-----------------------
---G
GTTTGCCATTTCGTTCTCCAGCTCATTTTA------------CAGATGAAGAAA-CTGCC
CCAAACAG-GATTAAGTAACTTGCCCA----GAGTCACACAGCAATAA------------
--GTGTCTG-AGGCCAGATTTGAACTCAGGAAGATGAATCTTCCTGATTCTAG-GTTCAG
TATTCTATCCACTGCACC-------ACCTAGNTGACC----AAGTATGAAGGAA---AGG
GAATGATATTTT-ATAGGGGAGTAATGTATCTGATACTACAGAATGAACCATAGTTAA--
----AGTTATGAGTGGA-GTTTTATTATATCG--TATTTAC-------------------

-----------------------TATAGCTAAA-CTTCCAGCAGAACATCATGCTTTTTT
--GTTTATTTTACCCTCTTAACTTATTTTACTTTTTTTCTCTCTTTTTCTGCACTTC-CC
CCACCCCCTCCTT-ACCCACAAAAA--CCTTTTATGCAG--CCTTTTTGGCCGATGGGAA
CTGGAATAGCCCGAGGCTTTCTGGCTGCTATGGACTCAGCCTGGATGGT
>Mro
ATCAGAGAATGCAGCCCTGGTTCGAGAACAGAATGGTCACCAGTTATTGGTGGCCCTGGT
GGGGGATAGCCTGCTTGAG--GTGAGTATAAAGGAA-----ATTGTTCAGT---------
---CACG----TCCAACTCTTCGTGACCCCA--TTTGGGGTT------------------
----------------TTCT----TGGTAAAGAGG---------------------TATG
GATTGCCATTTCCTTCTCCAGCTCATTTTA------------CAGATGAAGAAA-CTGAG
GCAAACAG-GGTTGAGTGACTTGCCCA---------------------------------
--GTGTTTG-AGGCTAGATTTGAACTCATGAAGATGAATCTTCCTGAATCTAG-GCTCAG
CATTCTATCTACTGTGCC-------ACTTAGCTGCCC----GAGTATGAAGCAA---GGA
GATTGATATTTT-ATAGGGGAGTATTGTATCAG-----------------ATAGTTAG--
------ATATGAGTGGA-GTTTTGATATATCATATATTTAC-------------------

-----------------------TATAGCTAAA-CTTCTAGCAGAATGTCATGTTTTTC-
--ATTCATTTTTCCCTTTTAACTTATTTTACCTT--------CTTTTTCTACACTTC-GC
CCATCCCCATCTC-ACCCCCAAAAAAA-TTTTTATACAG--CCTTTTTGGCCGATGGGAA
CTGGAATAGCCCGAGGCTTTCTTGCTGCTATGGACTCAGCCTGGATGGT
>Vur
ATCAGAGAATGCAGCCCTGGTTCGGGAACAGAATGGTCATCAGTTATTGGTGGCCCTGGT
TGGGGATAGTCTGCTTGAG--GTGAGTATGAAAGAA-----GTCGTTGTT----------
---CAGTGGTGTCCAACTCTTTGTGACCACA--TTTGGGGTT------------------
----------------TTCC----TGGCGAAGAGG---------------------AGTG
GTTTGCCATTTCCTTCTCCAGCTCATTTTA------------CAGAGAAAGAAA-CCGAG
GCAAACAG-GGTTAAGCGACATGCCCA----GGCTCACACAACT---AGTAA--------
--GTGTCTG-AAGCCGGATTTGAACTCATGAAGATGAATCTTCCTGATTCCAG-GCCCAG
TGTTCTATCCACTGCACC-------ACT-GGCTGCCC----GAGTATGAAAGAA---GGG
GAATGATATTTT-ATAGGGGAGTAATGCATCTGATACTACAGAATGAGCCATAGTTAG--

```
----AGATATGAGCGGA-GTTTTTATTTATCATATATTTAC-------------------
------------------------------------------------------------
------------------------------------------------------------
-----------------------TCTAGCTAAA-CTTCCAGCAGAATGTCATGCTTTTTC
--ATTCATGTTTCCCTTTTAACTTATTTTACTTT----CTTTCTCTTTCTGCACTTC-CC
CCACCCCCTCTTC-ACCCTCAAAAAA--CTTTTACGCAG--CCCTTTTGGCCGATGGGAA
CTGGAATAGCCCGCGGCTTTCTGGCTGCTATGGACTCAGCCTGGATGGT
```

MARKER 102

>WSINE1

----------------------------TTTTTTTTTTTTTTGAGGCAATTGG-GGTTAA
-GTGACTTGCCCAGGGTCACACAGCTAGT-AAGTGTCTGAGGCTG---GATTTGAACTCA
G-GTCCTCCTGACTCCAGGGCTGGTGCTCTATCCACTGCACCACCTAGCTGCCCCTC---

>Mdo
ACCACATCAGTCACAGCCAGGCTGTGGAGGTGCTGAAGGGCCAGACGCACATCATGCTGA
CCATTAAG--GTAGACCATGGGCCACAGGGTTGT------------CCTGGACCTTGGGA
TGGAAA----GCAGAAATGGCAGCT-----ATTGGCGGGGAGAGGTAGACGACAGACGAC
AGGAGGGACGTCCTAAAAGTAGCCAGTGTAAGCACTGGA-AAAGGCAGCCTACTTCAGCA
GAGAGACTGAGTCCTGGTTTCTCAGTGGAAGGATGAACCCTGAAGCTCACAGTGGCAGGG
AGGAA-----CCTGATGACTTCTGAAGAGAGCTCGGCTTGGGGTCAGGGTGGGCAGAGAC
AGGGGA-GTACTCCCTGGGGCCATTATGTGGGCAATGAGGAGAAGA---GATTGTAGGCC
T---CCTAGACATGATTT--

--TAAA
GGTTCAACCAGG-----------------------GTGAGAGTGGAA--TCATTAACCAG
TTCCTGAGTCATATTCTCCCCATTACTTAGGATATGGGAGATGCTCATACCCTGTTCCCA
CC-CCCGTCCCCATTTCTCTTTTCCCCAATCCCCTGTCTCCCACCC-CCATTTCCCCACT
GATCTTCCAATTAC-TTCC-----------CTGTTCCTTATGGCATTCCTGTC-------
-----------ACTCTCCATTTCCCTCCCATTCCTC----CCCTCCTCTATCTGCCCC--
--------ACCTAG--GAGACAGGAAGATATCCAGCCTACAAGGAGATGGTGTCCGAATA
CTGCT
>Dvi
ACCACATCAGTCACAGCCAGGCTGTGGAAGTGCTGAAGGGGCAGACGCACATCATGCTGA
CCATTAAG--GTAAGCCAGGGACCACAGGGTCGTCG----GGAGCCCCAAGACCCAGGGA
TGGAAA----GCAGAAATGGAAGG------------------------AAGACAGACTAC
AGGAAAAGCTTCCTAAAAG-------------CACTATA-AAAGGCAGCCCACTTCATCA
GAGAGCCTGAGCCCTGGTCTCTCAGGGAAAGAATGAACCCTGAAGCTCATAGTGGCAGGG
AGGAA-----CAGGATGACTTCTGGAGGGAGCTAGGCTTGGGATCAAAGTGGGCAGAGAC
-CAGGA-TCACTCCCTGGGGCCATCATGTGGGCAATGAGAACAAGAAGAGACTGTAGGCC
T---CCTGGGCGTGATTT--

--CAAA
GGTTCAACGAGG-----------------------GAGAGAGTGGAA--TCATTAACCAG

TTCTTGAGTCAAATTCTCCCCATTACTTAGGCTATGGGAGATACTCATACCCTG------
-------------TTTCTCTTTTCCCCAATCCCCTGTCTCCCACCC-CCACTTCCACACT
GACCTTCCAATCACCTTCCCCACTACATCCCTGTTCCTTATTGCATTCCTGTC-------
----------CCCTCTCCACTTCCCTCCTGTTCCTCCTCTCTCTCCTCTCTGTGCCCC--
--------ACCTAG--GAGACGGGAAGGTATCCAGCCTACAAGGAGATGGTGTCTGAATA
CTGCT
>Mnu
ACCACATCAGTCACAGCCAGGCTGTGGAAGTGCTGAAGGGGCAGACGCACATCATGCTGA
CCATTAAG--GTAAGCCAGGGACCACAGGGTCGTCGT---GGAGCCCCAGGACCCAGGGA
TGGAAA----GCAGAAATGGAAGG------------------------AAGACAGACTAC
AGGGAAAGCTTCCTAAAAGT------AGCAAGCACTATA-AAAGGCAGCCCACTTCATCA
GAGAGCCTGAGCCCTGGTCTCTCAGGGAAAGGATGAACCCTGAAGCTCATAGTGGCAGGG
AGGAA-----CAGGATGACTTCTGGAGGGAGCTAGGCTTGGGATCAAGGTGGGCAGAGAC
-CAGGA-TCACTCCCTGGGGCCATCATGTGGGCAATGAGAACAAGAAGAGACTGTAGGCC
T---CCTGGGCGTGATTT--

--CAAA
GGTTCAACGAGG-----------------------GAGAGAGTGGAA--TCATTAATCAG
TTCTTGAGTCAAATTCTCCCCATTACTTAGGCTATGGGAGATACTCATACCCTG------
-------------TTTCTCTTTTCCCCAATTCCCTGTCTCCCACCC-CCACTTCCACACT
T---CCACACTGACCTTCCAATCACCTTCCCC-------ACTACATCCCTGTTCCTTATT
GCATTCCTGTCCCTCTCCACTTCCCTCCTGTTCCTTCTCTCTCTCCTCTCTGTGCCCC--
--------ACCTAG--GAGACGGGAAGGTATCCAGCCTACAAGGAGATGGTGTCTGAATA
CTGCT
>Cfu
ACCACATCAGTCATAGCCAGGCTGTGGAGGTGCTGAAGGGCCAGACCCACATCATGCTGA
CCATCAAG--GTAGATCCCTGAACACAGGCTTATTATCATAGACCCAGAGGACCCTGGGA
TGGAAG----GCAGAAATGGTAGCTA---CTATGTTGGGAGGGATAGAAGTCAGACTTGC
AGGAAAAACTTTCTGAAAG-------AGTAAGCACTAGA-AAAGGCAGCCTGTTTTATCA
GAGAGAATGAGCCCTGGTTTCTCAGTGGAAGAAGGGACCCTGTAGTGCTCAGAAAGATAA
GAATG------------ACTCCTGAGAAGAGCTAGGCTTGGGGTCAAGAAGAGCAGAGAC
-AGGGA-TAATTCTCTAGGGTCATTAAATA---------CAGAAGAAGCAA---TAGG--
----TCTTGATTTGATTT--

--AAAA
GAATCATCCAGAAGAAAAAAAAAAGAATCATCCAGGGATGGAGTAGGATTCATTAACCAG
CTCTTGGGCTATATGTTCCCCATTGCTTATGATAGAGGAGAG------ACCCTGTTTCCA
TT-CCTACCCCCATTTCTCAGTTCCCCAGTCACCCATTCCCCATCC-CCATTCCCACACT
GATTCTCCAATCTCCTTCCCATCTACTTCCCCATC---TAATGCATTCTATTC-------
------------------ATCCCCCCCTTTCCTTCTCGTCCCCTCCCCACC---------
--------A-CTAG--GAGACAGGAAGGTATCCGGCCTACAAGGAGATGGTGTCTGAGTA
TTGCT
>Dgl
ACCACATCAGTCACAGCCAGGCTGTGGAGGTGCTGAAGGGCCAGACGCACATCATGCTGA
CCATAAAG--GTAGACCACTCGCCACAGGCTTGT------AGACCTATAGGACCCTGGGG
TGGAAGGAAGGCAGAAATGGCAGCTGCGACTAGGTTGGGAGGGATAGAAGTCAAACT-GT
AGGAAGAACTTTCTAAAAGGAGCCAATGTAAGCACTAAA-CAAGGCAGCCAACTTTCTCA
AAGAG--CAAGTTCTAGTTTCTCAGAGCAAGGAGGGACTCTGTAGCTCAGCAGTGGGGAG
GAA-------GAGCATGATTTCTAGAGAGAGCCGGGTTTGGAATTAAGGAGGAGATTACT
CTTGGG------------GTCCATTATAAGGGCAACAAAAGGAAGAAGGGGCTGTTGGCC
T---TCTAGATTTGATTT--

```
------------------------------------------------------------
------------------------------------------------------------
--------------------------------------------------------TAAG
GGATCACCCAGGG--------------------AGGGAG---TGGGA--TCACTAACCAT
TTGTTGGGTCATATTCTCCCTGTTGCCTAAGATATGGGAGATATTCATACCCTCTTCCCA
CC-CCCATCCTCTTTTCTCAATTCCATAATCGATTGTCCTCCACCC-CCATTCCTACACT
TATCCTCCAGTCTCCTTCCCATCATCATCCCC------TAATGCAGCTCATTCTCCCTCT
CC----------------ATTCCCCTCCCCTTTCTCCTCTCCCTTCTCCCCTTCTCC---
--------ATCCAG--GAGACTGGAAGGTATCCAGCCTACAAGGAGATGGTGTCTGAGTA
TTGCT
>Nty
ACCACATCAGTCACAGCCAGGCTGTAGAGGTGCTGAAGGGCCAAACGCACATAATGCTGA
CCATAAAG--GTAGGCTGCTGACTGCAGAATTGTCAT---GGGCCCATAGAACCCTGGGA
TAGAAG---GCGAAATTGGTAGTTA----CTAGGTCAGGA-AGAATAAAAGTCAGACTGT
AGGAAGAACTTTCTAAAAGTAGCAAGGGTAAGCACTAAA-CAAGGCAGCTGACTTTATCA
GAGATAGCAAGCCCTAGCATCTCAGTGGAAAGACTCAGAGTG----------GTAGGGAG
GAA-------CAGGATAAATTCTAGACAGAGCTAGGCCTGGGGTCAAGGAG---ATTACT
GCGTGG-GGCCATCATATGGGCAATGAAA-----------GGAAGAAGAGACTGAAGGTC
T---TCTAGATTTGA------------------TTTGTGGGGGTGGGGAGCCA-------
-------------GGGTCACACAACTAGTTATATATCTTAGGCCA---GATTTGACCTTA
GGCATTCCTGACTTCCAGGCCCGGTGCTCTATCCATTGTGCCACCTACCTNCCCCT----
TCTACATTTGATTT----------------------------------------------
-----------------------------------------TANAAA--TCATTANCCAG
TTCGTGAGTCNCATN--CCCTGTTGCTTAANANATG-----AAACTCAACCCTTTTCCCC
ACTTCTTATCCCATTTCTCAATTTTCCAATTCCCTGTTCCCCATTC-CCATTCCCACACT
GATCTTCCTATCTCCTTCCCATCCCCAACCC-------AAATGTGTTCCTCTC-------
---------TCCCTCTCCATTCCTCTTCACTTCCTCCTCTC-------------------
--------CCACAG--GAGACAGGAAGGTATCCAGCCTACAAGGAGATGGTATCTGAGTA
CTGCT
>Dgo
ACCACATTAGTCACAGTCAAGCTGTGGAGGTGCTGAAGGGTCAGACACACATCATGCTGA
CCATCAAG--GTAGGCCATTGACTGTAGAGTTGTTAC---AGACCCATCAGACTCTGGGA
TGGAA----GGATAAA----------------AGTTAGA----------------CT-GC
AGAAAGAATTTTCTAAGAGTAGCAAGTGTAAGCATTAAA-CAAGGCAGCTGGCTTTATCA
G------CAAGCCCTGG---CTCTAT------------------AACTCAGAGTAATGGGG
GAACA-----CAGGTTGACTTCTGGAGAGAGCTAGGCCCAGGGTCAAGGAA----TTAGT
CTCTGTTGACCATTATATAGACAATGAAA-----------GGAAGAAGAGGCAGGAGGCC
T---TTTTTTTTTTTTTT---------------------TTTTGAGAGGAAGA-AGTTAA
-ATAACATGTCCTGCGTCACATAGCTAGT-AAATATTTGAAGGTG---GATTTGAACACA
G-GTCCTCCTGACTCCAAAACTGGTGTTCTGTCCACTGTGCTAACTAACTGCCTCT----
TCTAGATTTTATTT------------------------------------------TAAG
GGATCAACCAGG---------------------GAGAG--ATTGGGA--TCATTAATCAG
CTCTTGGGTCATATTTTTCCCATTGTTTAAACTATGTAAGATAATCATACCCTTACCTCC
------------ATGTCTCAATTCCCCAATTCTCTGTTCCC-ATCC-CAATCTCCATAAT
----TCCCATTCCCATTCC-------------------CACTGCATCCCTTTCTCCCTTT
CCATT----CCTCTCCCAATTCCTCTTCTCCTTTCTTCCTCCCTTCTCTCCTTCC-----
--------CCTCAG--GAAACAGGAAGATATCCAGCCTATAAGGAGATGGTGTCTGAGTA
TTGCT
>Scr
ATCACATTAGTCACAGCCAAGCAGTGGAGGTGCTGAAGGGTCAGACACACATCATGCTGA
CCATCAAG--GTAGGCCACTGACAATAGGGTTGTTAC---AGACCCATTGGACTCTGGGA
TGGAA----GGATAAA----------------AGTCAGA----------------CT-GC
AGAAAGAACTTTCTAAGAGTAGCAAATCTAAACACTAAAACAAGGCAGCTGGCTTTATCA
```

G------CAAGCCCTGG---CTCTAT-----------------AACTCAGAGTAATGGGG
GAACA-----CAAGGTGACTTCTGTAGAGAGCTAGGCCCAGGCTCAAGGAA----TTAGC
CTCTGT-GACTATTATATAGACAATGAAA-----------GGAAGACGCAGT---AGGCC
T---TCTTGATTTGATTT---------------TTTTTTTGTGGGGGGGAGGA-AGTTAA
-----CATCTCCTGGGTCACACAGCTAGT-AA-TATTTGAGGGTG---GATTTGAACACA
G-GTCCTCCTGATTCCAAAGC-TGTGTTCTATCCACTGTACTACCTAACTGCCTCT----
TCTAGATTTTATTT--TAAG
GGATCAACCAGG---------------------GAGAG--ATTGGGA--TAATTAACCAG
TTCTTGGGTGATATTTTTCATATTGTTTAAAATATGGAANATAATCATACCCTTACCTCC
------------ATGTCTCAATTCTTCAATTCTCTGTTCCC-ATCC-CAATCTCCATACT
----TCCCATTTCCTTTCC-------------------TACTGCATTTCTTTTTCCTTTT
CC----------------ATTCCTCTTCTCTTTTCTTCCTCCCTTCTCCCCCTTC-----
--------CTTCAG--GAAACGGGAAGGTATCCAGCCTATAAGGAAATGGTGTCTGAGTA
CTGCT
>Mfa
ACCACATTAGTCATAGCCAAGCTGTGGAGGTGCTGAAGGGTCAGACACACATCATGCTGA
CCATCAAG--GTAGGCCACTGACCATAGGGTTGTCAT---AGACTCATCCGACTCTGGGA
TGGAA----GGATAAA----------------AGTCAGAA-----------------TGC
AGAAAGAACTTTCTAAGAGTAGCAAGTGTAAGCACTAAA-CAAGGCAGCTGGCTTTGTCA
G------CAAGCCCTGGTTTCTCTGTGGAAGGAAGGGCTCTATAACTCAGA---------
-GACA-----CAGGGTGACTTCTGGAGAGAGCTAGGCCCAGGATCAAGGAA----TTAGC
CCCTGT-GACCATTATATAGTCAATGAAA-----------GGAAGAGGAGGCAGGAGGCC
T---TCTTGATTTGATT-----------------GGGTTTTTGGGGGGAGTGA-AGTTAA
-ATAATATGTCCCGGGTCACACAGCTAGT-AAATAGCTGGGTTTG---GATTTGAACACA
G-GTCCTCCTGACTCCAAAGCTGGTGCTCTATCCACTGTGCCACTTAACTGCCTCT----
TCTAGATTTTATTT--TAAA
GGATTAACCAGG---------------------GAGAG--ATTGGGA--TCATTAACCAG
TTCTTGGGTCATATTTTCCCCATTGTTTAAAATATGGGAGGTAATCATACCCTTACCTCC
------------ATGTCTCAGTTCCTCAATTCTCTGTTCCC-ATCC-TGATTTCCATACT
----TCCCATTCCCATTCC-------------------TACTGCATTCTTCTCTCCCTTT
CCATT----CCTCTCCCAATTCCTCTTCTTTCTTCCTCCTTTCTCCTCTTCCC-------
--------TCTCAG--GAAACAGGAAGGTATCCAGCCTATAAGGAGATGGTGTCTGANTA
CTGCT
>Bil
ACCACATCAGTCATAGCCAAGCCGTGGAGGTGCTAAAGGGCCAGACACATATCATGCTGA
CCATAAAG--GTAGGCCACTGCCCACAGGGTTGTCAT---AGACCC---------TGGGA
TGGAA-----AATAAA-----------------------AATGGTAGCTACTAGGTTGGG
ATGTATAAAAGTCAGACTGTAGGCAGAGTAAGCACTAAA-CAAGATAGCCTACTTTATCA
GAGAGAGCAAGCCCTGGTTTCCCAGTGGAAGGAGGAACTCTGTAGCCGAGTGGTGAGGAG
AAACA-------GGATGAGTTCTGGAGAGAGGTAGACCTGGGATCAAGGAGGAGATTACT
CTCTGG-GGCCTTTTTTTGGGCAATGAAA-----------GGAGGAAGTGGCTCTTGGCC
T---TCTAGGCTTTATGT---ATCTATCTATCTATTTATTTATTTTTGTTGAGGGGG---
------AAACCCCAAGTCACACACCTA---------------------------------
--GTCCTCCTGACTCCAAAGTTGGTGCTCTATCCACTTTGTTATCTAGTTGCCTCT----
TTTAGGTTGGATTC---TATTTTAATTTTTTAATTTTTTTCCAATTGGGTTTGATTTAAA
GGTTCAACCAGG---------------------GAGGGAATGTGGGA--TCATTAACCAG
TTCTTGGGTCATATTCTCCCCTTTGCTTAGGATATGAGAGGTACTCATACCCTGTTCCCA
C--CCTATTCCTCTTTCTCAATTCCCCAATGTCC-TTTCCCTCCCCTCTATCCCCACACT
-----------ATTTTTTA-------------------TAATGCATTCC-----------
---------TCCCTCTCTATTCCTCTCCTCTTCCTCCCCTCCC-----------------
--------ACTCAG--GAGACAGGAAGGTATCCAGCCTACAAGGAGATGGTGTCTGAGTA
TTGCT
>Pgu

```
ACCACATCAGTCATAGCCAGGCTGTGGAGGTGCTAAAGGGCCAGACACACATCATGCTGA
CCATAAAG--GTAGGTCACTGCCCATA-GGTTCTCAT---AGACCC-TAAGATCCTGGGA
A--------GTATAAA---------------------------------AGTCAGACTGT
GAGCAGAACTTTCTAAAAGTAGCAAGTGTAAGCCCTAAA-CAAGAAAGAT----------
-------CAAGCCTTGATTTCCCAGTGGAAAGAAGAACTCTGTAGCTCAGACTAGTGAGG
AAGAG-----CAGGAAGACTTCTGGAGAGAGGCAGACCTGGTATCAAGGAGGAGATTACT
CTGTGG-GACCATTTTATGGGCAATGAAA-----------GGAAGAAGAGGCTGTGTGCC
T---TCTAGCTTTTATTT-------------------ATTTATTTAGCT-----AGTTAA
TTTT----------AGTCACACACCAA---------------------------------
--GTCCTCCTGATTCCAGAATTGGTGTTCTATCCACTTTATTAGCTAGTTGCCTCT----
TTTAGGTTGGATTC---TATTTTAA--------TTTTTTTCCAGTTGGATTTGATTTAAA
GGATCAATCAGG---------------------GAGGGAGTGTGGGA--TCATTAACCAG
TTCTTGGGTCATATTCTCCCCTTTGCTTAGGATATGAGAGATACTCATACCCTGTTCCCA
C--CCTATTCCCCTTTCTTGATTCCCTAATTCCC-TTTCCCTCCCTTCTATTCCCATACT
ATTCCCACATCATTTTTTA-------------------TAATGCATTCCTCCC-------
---------TCCCTCTCCATTCCTCTCCTCTTCCTCCCCTTCC-----------------
--------ACTCAG--GAGACAGGAAGGTATCCAGCCTACAAGGAGATGGTGTCTGAATA
TTGCT
>Iso
ACCACATCAGTCATAGCCAGGCTGTGGAGGTGCTAAAGGGCCAGACACACATCATGCTCA
CCATAAAG--GTAAGTCACTGCCCACA-GGTTCTCAT---AGACCC-TGAGATCCTGGGA
A--------GTATAAA-------------------------------------AGACTGT
GAGCAGAACTTTCTAAAAGTAGCAAGTGTAAGCCCTAAA-CAAGAAAGAT----------
-------CAAGCCCTGGTTTCCCAGTGGAAAGAAGAACTCTGTAGCTCAGACTAGTGAGG
AAGAG-----CAGGAAGACTTCTGGAGAGAGGCAGACCTGGGATCAAGGAG---ATTAGT
CTGTGG-GGCCATTTTATGGGCCATGAAA--------------GGAAGAGGCTGTGGGCC
T---TCTAGCTTTTATTT-------------------ATTTATTTAGCT-----AGTTAA
TTTTAG-TC-------ACACACCAA-----------------------------------
--GTCCTCCTGATTCCAGAATTGGTGTTCTATACACTTTGTTAGCTAGTTACCTCT----
TTTAGGTTGGATTC---TATTTTAA--------TTTTTTTCCAGTTGGATTTGATTTAAA
GGATCAATCAGG---------------------GAGGGAGTGTGGGA--TCATTAACCAG
TTCTTGGGTCATATTCTCCCCTTTGCTTAGGATATGAGAGATACTCATACCCTGTTCCCA
C--CCTATTCCCCTTTCTTGATTCCCTAAT-CCC-TTTCCCTCCCTTCTATTCCCATACT
ATTCCCACATCATTTTTTA-------------------TAATGCATTCCTCTCCTCTTCC
TCCCCTTCC---------------------------------------------------
--------ACTCAG--GAGACAGGAAGGTATCCAGCCTACAAGGAGATGGTATCTGAATA
TTGTT
>Tro
ACCACATCAGTCACAGCCAGGCTGTGGAGGTACTGAAGGGCCAGACGCACATCATGCTGA
CCATAAAG--GTAGGCCCTGACCACAA-GGCTGTCCT---AGAACA--AGG-CCCTGGGA
TGGAAA---GCAGAAA-----------------------TGGCATCTATTAGATTAGAAA
GAATAGAACCTTCTGAAAGCAGCAAGTGTAAGAACCACA-AAAGGCAGCGGACTTTATCG
GAGAGAAGCAGCCCTGGTTTTTCGGTGGAAGGAGGGGCTCTGTATCTCAGAGTGGTGCCA
GGAAC-----CAT-ATGACTTCTGG--AGAACTAAGCCTGGGGTCAAGAAGGAGAGTGCT
CCCTAG-GGCCATTATATGGGCAGTGAAA-----------GGAGTAGGAGGCAGGAAGTC
T---TCTAGATTTGATTT-----GGGAGTGGGAGGGTAGGAAGAAGCAATTGG-AGTTAA
-GTGACCTACCCT-GGTCACAAAACTGGTACGTGTCTGACTGAGAACAGATTTGAA----
-----CTCCTGACTCCAGAGCCAGTGTTCTA-CCACAATG------------TCCT----
TCTAGACTTGATTT------------------------------------------TGAG
AGATCAGCCAGG----------------------------------A--TCATTAATCAG
TTCCTGGGTCATATTATCCCCATTGCTTAAGATA----CCCTGTTTCTACCCTTATC---
---------TCCATTTCTCAATTCCCCAATTCTCTGTCCTCCACTC-CCATTCCCACACT
GATCCTCCATCTTCC------CTCCCATCCC-------CAATGTATTCCTGTC-------
```

```
---------TCCTTCCCTACTGCTCTTCCATTTCCCCTCTCTCTTCTTCTCTCCTTTTCC
CCCTCTCCT-TCAG--GAGACAGGAAGATATCCAGCCTACAAGGAGATGGTGTCTGAGTA
TTGCT
>Ppe
ACCACATCAGTCACAGCCAGGCTGTGGAGGTGCTGAAGGGCCAGACGCACATCATGCTGA
CCATAAAG--GTAGGCCCTGACCACAG-GGTTGTCAT---AGACCCCTAGGGCCCTGGGA
TGGAAG---GCAGAAA-----------------------TGGTAGCTACTAGGTTAGGAA
GCATAGAACTTTCTAAAAGTAGCAAGTATAAGCACTAAA-TAAGGCAGCCAACTTTATCA
AAAAGAGCAAGCCCTAGTTTTTCAGTGGAAGGAGGGGCTCTGTATCTCAAAGTAATGGGG
GGGAA-----CAGGATGACTTCTGGAGAGAACCAGGCCTGGGGTCAAGGAGGAGATTGCT
CCCTGG-GGCCATTACATGGGCAGTGAAA-----------GGAAAAAGAGGCTGTAGCTC
T---TATAGATTTGATTT---GGGGGTGGGAGGGTGGGAAGGGAAGCAATTGG-AGTTTA
-ATGACTTACCCTGGGTCACATAATTAGTAAGTGTCTGAGACCA----GATTTGAA----
-----CTCCTGACTCTAGGGCCAGAGCGCTATCCATTTTGCCACCACAGTCCCCTT----
-CCAGATTTGATTT------------------------------------------TAAG
AGATAAACCAGG---------------------AAGGGAGTAGT-GA--TCATTAACAAG
TTCTTTGGTCATATTCTTCCCATTCCTTAAGATA----CCCTGTTCCTACCCTTATC---
---------TCTGTTTCTCAATTCCCTAATTCCCTGTCCTCCACCC-CTATTCCCACACT
GATCCTCCAATTTCCTTCCCATCTCCATCCC-------TAATGCATTCCTCTC-------
---------------------------CCATTTCTC---CTCTTCCTTCTCCCCTTTTCC
CCCCTCTCTTTCAG--GAGACAGGAAGGTATCCAGCCTATAAGGAGATGGTGTCTGAGTA
TTGCT
>Mro
ACCACATCAGTCACAGCCAGGCTGTGGAGGTGCTGAAGGGCCAGACGCATATCATGCTGA
CCATAAAG--GTAGGCCCTGACCACAA-GGTTGTCAT---AGACCCATAGGACCCTGGGA
TGGAA----GCTA----------------CTAGGTTAGGAAG-GATAAAAGTAAGTCTGT
AGGAAGAACTTTGTAAAAGTAGAAAGTGTATGCACTAAA-CAAGGCAGCAAATTTTATCA
GAGAGAGCAAGTCCTAGTCTCTCAGTGGAGGGAGGGGCTCTATATCTCAGAGCAGGGGGA
TGGAGGGGAACAGGATGACTTCTGGAGAGAACTAGGCCTGGGATCAAGGAGGAAATTGCT
CCCT-G-GGCTATTCTGTGAGCAGTGAAA-----------GGAAG---------------
T---TCTAGATTTGATTT---TTTTTGGGGGGGGGGGAGGGAAAGCAACTGG-AGTTAA
-GGGACTTGCCCCAAGTAACTTAGCTGGTAAGTGTCTGAGGCTA----GATTTGAA----
-----TTCCTGATTCCAAGGTCAGTGCTCAATCCACTGTGCCATCTGACTGCCCTT----
-GTAGATTTGATTT------------------------------------------TAAG
AGATCAACCAGG---------------------GAAGGAG------------TTAACCAG
TTCTTGGGTCACATTCTCCCCATTGCTTAAGATA----CCCTATTCCTACCCTTGTC---
---------CCC-------ATTT-CTCAATTTCATGTTCCCTGCCC-CCATTCCCACACT
GATCCTCCAGTTTCCCTTAAATCCCCATTAC-------TAATGCATTCCTCTC-------
------------TCCTCCATTCCTCTCCCTTTTTTCCTCTTCCTTCTTCTCCCCCTTTCC
CTTTCCCTC-TCAG--GAGACGGGGAGGTATCCAGCCTACAAAGAGATGGTGTCTGAGTA
TTGCT
```

MARKER 168

\>MIRc

---AATAATAAT
A---ACCCCTTACATTTGTATAGCACTTTACAGTTTACAAAGCGCTTTCACATACATTAT
CTCATTTGATCCTCACAACA---ACCCTGTGAGGTAG---GCAGGGCAGGT---ATTATT
ATC---

\>Mdo
TGACAAGCGCAGTCTCCG--GTGAGCCTGGGCGCTGCCCCCATTCCT-CACCCT-CGGGC
TCTGATGCCCTAGAACTGGGGGGGCCGGGGGGGCCACCCTGCTCACTGGGGTTCTGGGTG
GTGCAGGAGGCTCAGGAGGGAGA-------GCCGACGGATGGCCTGCCTC----------

-----------GGGTCCCC-GTTAGAGCGTGCGCAGGGGAGGGAAGGGATGG--------
--------CGAGTTAGCGTA----------------------------TG---TCCTGCG
TGGCCCTGGGGGCC--GCCTCCTTCCTCCCTGC--CAG--GGTCCCCAAACGCCCCGTCA
TCAACACGCCA
\>Dvi
CGACAAGCGCAGTCTCCG--GTGAGCTCAGGCG----CCC----CCTACATTCCTTCCCC
TCTGATGCCATGGAACAGG-GGGG---------CA-CCCTGCTCATTGGGGTTCTGGGTG
CGGGCGGGGG------T--TGG----------------GAGGAAGA--------------

------GTGAGCACTTACATGGGTTGTGGGGGGGACGGGGAGGAGGGATG-----GTGAG
TTAGT---
--------GCGTCCC-GCCTCCTCCCTCCCTGC--CAG--GGTCCCCAAACGCCCTGTCA
TCAACACACCG
\>Mnu
CGACAAGCGCAGTCTCCG--GTGAGCTCAGGCA----CCC----CCTACATTCCTTCCCC
TCTGATGCCATGGAACAGG-GGGG---------CA-CCCTGCTCATTGGGGTTCTGGGTG
CGGGCGGGGG------T--TGG----------------GAGGAAGA--------------

------GTGAGCACTTACATGGGTTGTGGGGAGGACGGGGAGGAGGGATG-----GTGAG
TTAGA---
--------GCGTCCC-GCCTCCTCCCTCCCTGC--CAG--GGTCCCCAAACGCCCTGTCA
TCAACACACCG
\>Rra
TGACAAGAGGAGTCTTCG--GTGAGCTTGGCTTGGCTCTCATTGCCCTTGTCCCTGCCCC
TTTGCTGCT---GAAGTGGGGTGGG--------CAGCCCCGTGGAGGGCTACGA---CCT
GACAAGGAGCTGGCGCTTATAT------GGTACCTGAAGGCTAACAAC------------

------AAACTGACTTCCTTTTATTACCTGCGGGTGTACAGGGAAGGCGTGG---GGTTG
TGCAT--GAGGGGAGCTAAGACTTTGGAGTCCTTGGGGGGGTGGGGTTTGACCACATAAG
CCATGGCTCCGGGGGGGGGCCATTTCTCCTCAC--CAG--GGTCCCCAAACGTCCTGTCA
TCAACACACCA

>Cfu
TGATAAGAGGAGTCTTCG--GTGAGCTTGGCTTGGCTCCCATTGCCCTTGTCCCTGCCCC
TTTGCTG--ATG-AAGTGGGGTGAG--------CAGCCCTGTGGAGGGCTATGA---CCT
GACAAGGAGCTAGCGCTTGTAT------GATACCTGAAGGCTAAGAAC------------

------ATACTGGCTTCCTTTTGTTACCTGTGGGTGTACAGGGAAGGCGTGG---GGTTG
TGCAT--GCACAGAGCTAAGACTTCGGGGCCCTT-GGGGGGTGGGGTTTGACCACATAGG
ACACGGCTCC--GAGGGAGCCATTTCTCCTCAC--CAG--GGTCCCCAAACGTCCTGTCA
TCAACACACCA
>Dgl
TGACAAGCGCAGTCTTCG--GTGAGTTCAGCGTGGCACCTATTGCCGTAT-TCCTTCATC
TCTTCTGACGCAAAGGAACTGGGCAGAGACCAGGAGCCC---------------CATCCT
GATCAGGGGGG-----TTGTGG------CTATAATGGGCTCAGTGAGA------------

------GCGGCTGACATTGAGATAGCGTGTGCATGTGC-----------------GTGTG
CACGC--GTGTGCACCTG------------------------------TG-A-CCCTGCA
TGGCCCTGGGGTGCC-ATCTCCTTCCTCCCCAC--CAG--GGTGCCCAAACGTCCTGTCA
TCAACACGCCA
>Nty
TGACAAGCGCAGCCTTCG--GTGAGTTTGGCCCAGCTCTCTCACCC--------------
-------ATGGCGGGAGGCACG-----------GAGCCT-AAGCAGGCAGGGC----CAT
CCAGAG---------CACGCCA------CT------GGC---ATCTAG------------
----ATAGCTCGTGAAGGCTGATAGGTTTT------------------------------

----------------GTCTCCTTCTTCCCCAC--CAG--AGTCCCCAAACGTCCTGTCA
TCAACACCCCG
>Pta
TGACAAGCGCAGCCTGCG--GTGAGCTCAGC-GTGCGCCCGCTGCCCGGCGTCCGTCCCT
G---CTGATGCAGAGGAGCTGC----GGGGGAGGGGCCG-GGTGGGCGGTGGC-----CC
TCATAGTCGGG-----TC-----------------------AGGAA--------------
------------------------GCTTCACAGCTGAGTGTGTG----------CCTCGT
GCCATCTGAGCCTC-CAGCA----------GAGA----GTGCCAGGGACAC---------
------GTG----GCCCCGGGTGATAGAGAGT----------------CCATGGGCAC--
---------ACACA----------------------------------TG-ACCCC----
----------GGGGCCCTCTCCTTCCTCCCCAC--TAG--GGTTCCCAAACGGCCCGTCA
TCAACACGCCC
>Scr
TGACAAGCGCAGCCTGCG--GTGAGCTCAGCAC-GTGCCCGCTGCCCTGTCCTCTTCTT-
----CTGATGGAGGGGAGCCGGGGA-GGGGGAGGAGCC--AGGCAGGGGGGCTC----CC
TCATAGTTGGG-----TC-----------------------AGGAAGT------------
----GTAGCTGGTGTTTAGACCACACTTTACAGCTGTGTTTGTA----------CCGCGT
GGTACCTGATCCTC-CAGCA----------GAGAGTGTGTGAGGGGCACGT---------
------GCAC-------TGGGATAGTGTGTGCAAAAGAGCGGAGCAGC--AGGGTGTGGT
GCAG-GTGCCCACGTGCACATCCA------------------------TG-ACCCTG---
----------GGGGCCGTCTCCTTCCTCCCCAC--TAG--AGTCCCCAAACGGCCTGTCA
TCAACACGCCA
>Mfa
TGACAAGCGCAGCCTGCG--GTGAGCTCAGCACGGTGCCCGCTGCCTGGGGTCCTTCCCT
T---CTGATGAAGAGGAGCTGGGA---GGGGAGGAGCCC-AGGC-GGGAGGGC-------

-----------------AGCC-------CTCAGATTGGCTCAGTAGGC------------
----ACAGCTGGCATTTAGATCACGCTTTACGACTGGC--AGTC-------GCGCCTTGT
GTTATCTGATGCACACAACA----------------GTGTGCGAGGGACAC---------
------GTG------GCACTGTGACAGTGTGTGCAAG---GAGGTGGAGCAGGTGC----
---------CCACG----------------------------------TGCACCCATGTG
A--CCCAGGGGGCC--GTCTCCTTCCTCCCCAC--CAG--GGTCCCCAAACGGCCTGTCA
TCAACACGCCA
>Pgu
AGACAAGCGCAGTCTTCG--GTGAGCTGGGCCAGGTGCCCAATACCT-------------
--------------------GTGGG--------GAGCGGGGAGGAT-----------CCT
GGGCGATGGGGG----TCT-GC-------TGTGAGAGGGGCTAACACC------------
------------------CACAGTGCTTTAGGGTTTACGCAGTC------TGTTCCTTGT
GTTACTTGATCTTTGCAACA---GCCCGATCAGGG-CTGTGTGGGGGATGT---------
------GTAGGATTATGTGTT-----------------------------------AGTA
TGTGC--ATGGGCTTCTA------------------------------TG-ACCCTTGCC
TGGTCCTGGGGGCCAT--------CTTCCCCAC--CAG--GGTCCCCAAGCGTCCANTCA
TCAACACACCA
>Tro
TGACAAGCGCAGCCTTCG--GTGAGCTCCGCCGGCCTCTCACTGCCCACGTCCCTGTCTT
TCTGTTGATGAAGAGGCAGTGGGGAGGAGGAAGGAGCCC-GTGCAGGCGAGGC-TGTCCT
GCTCACTGGGG-----TTATGG------CTGCAGCAGGGCCAGGCGG-------------
---TGCTTGGAG-
GTTATTTGGCCCTTGTGACA---ACCCTGTCCGAGTGTGTGCAGGGGATGT---------
------GTGGGGC-------TTGTTAGTGTGTGTAAGAAAATGGAGCAGCAGGGGATGT-
AGCAT----GTGTGCATA------------------------------TG-ACCCCT---
--------GGGGCCCTGTCTCCTTCCTCCACAC--TAG--GGTCCCCAAACGTCCCGTCA
TCAATACGCCA
>Ppe
TGACAAGCGCAGCCTTCG--GTGAGCTCAGTGTGGCTCTCACTGCCCACATTCCTTTCCT
TCTTCTGATTGAGAGGCACTGGGGAGGGGGAAGGAGCCC-AGGCAGGTGGGGC-TGTCCT
GATCACTGGAG---TATACTGG------CTGTAAGAGGGCCAGTACAT------------
----ACGGCTGGCATTTAGATAGCGCTTGAAGGGCAACAAAGTC------CATTCCTTAT
GTTATTTGGTCCTCAAGACA---ACCTTGTATGTGCAGGTGACATGTAGGG---------
----------------TTGTGTGTTAGTGTGTATAAGAAAATGGAGCAGCAGGGTATGTG
TGTGC--ATGTGCGCATA------------------------------TG-ACCCCTGAA
TGGCCCTGGGGGCCCTGTCTCCTTCCTCCACAC--TAG--AGTCCCCAAACGTCCCGTCA
TCAACACACCA
>Tvu
TGACAAGCGCAGTCTTCG--GTGAGCTCAGCATGGCTCCCGTTGCCCACATTCCTTTCCG
TCTTCTAGTGAAGAGGAACCGGGAAGGGGGAAGGAGCCC-AGGCAGGCGGGGC-TGTCCT
AATAATTGGGG-----TTATGG------CTGTAACAGGGTCAGTAAAC------------
----ATAGCTGGCATTTAGATGGTGCTTTACGGTCGACAAAGTC------CATTCCTTCT
GTTATTCGATCCTCACAACA---GTCTTGTCCGAGTGTGTGCACAGGACGT---------
------GTAGGATTGTGTGTGTGTTAGTGTGTATAAGAAGATGGAGCAGCAGGCTGTGTG
TGCAT--GTGCATGCATGTCCCCGTG----------------------TG-ACCCCTGCG
TGGCCCTGGGGGCCCTGTCTCCCTCCTTCCCAC--TAG--GGTCCCCAAACGTCCTGTCA
TCAACACACCG
>Mro
TGACAAGCGCAGTCTTCG--GTGAGCTCAGTATGGCTTCCATTGCCTACATTGCTTTCTC
TTTTGTGATGAAGAGGAG--------------GGAGCCC-AGGCAGGTGGGGC-CATGCC
CAGG-TTGGGG-----TTATGG------CTGTAACAGAGTCAGTAAAC------------
----ACAGCTGGCATTTAGATAGTGCTTTAAGGTTGACAAAGTC------TGTTCCTTAT
GTTATTTGATGCTCACAACA---ACCCTGTCAGAGTGTGTGCAGGGGATGT---------

------GTAGGATT-----------GTGTGTATAAGAAAAGAAAGCAGCAAGATGTGTG
CATGT--ATGTGTGCATA------------------------------TG-ACCCCTGCA
TGGCCCTGGGAGCCCTGTCTCCTTCCTTCCCAC--TA---GGTCCCCAAACGTCCTGTCA
TCAACACACCA
>Ptr
TGATAAGCGCAGTCTTCG--GTGAGCTCAGTATGGCTTCCATTGCTTACGTTGCTTTCCC
TTTTCTGATGAAGAGGAA-------------GGAGCCC-AGGCAGGTGGGGC-CATGCC
CAGG-TTGGAG-----TTATGG------CTGTAACAGAGTCAGTAAAC------------
----ACAGCTGGCATTTAGATAGTGCTTTAAAGTTGACCAAGTC------TGTTCCTTAT
GTTATTTGATGCTGACAACA---ACCTTGTCAGAATGTGTGCAGGGGATGT---------
------GTAGGATT------------GTGTGTATAAAAAAAGGAAGCAGCAGGATGTGTG
CATT---ATATGTGCATA------------------------------TG-AGCCCTGCA
TGGCCCTGGGGGCCCTGTCTCCTTCCTTCCCAC--TAG--GGTCCCCAAACGTCCTGTCA
TCAACACACCA
>Vur
TGACAAGCGCAGTCTTCG--GTGAGCTCAGCGTGGCTGCCCTGCCCTTAT----------
---TCTGATGAA---TAACTGGGGAGGGGGATGGAGACC-AGGCAGGTAGGGC-CATCCT
AATAATTGGGG-----TTGTGG------CTGTGATAGGGTCAGTTAAC------------
----ATAGCTGGCGTTCAGATAGTGCTTTAAGGTTGACAAAGTG------CGTCCCTTAT
ATCATTCGATCCTCACGACC---ACCCTGTCAGAGTGTGTGCAGGGGATGT---------
------GTAGGGC----TGTGTGTTAGTGTGTATAAGAAAGTGGAGCAGCAGTATGTGTG
TGCAT----ATGTGCGTA------------------------------TG-GCCCCTGCA
TGGCCCTGGGGGCCCTGTCTCCTTCCTCCCCAC--TAG--GGTCCCCAAACGTCCTGTCA
TCAACACACCG
>Human
TGACAAGCGCAGCTTGAG--GTCAGC----------------------------------
--------------------AGCTAGGGGAC---------AGGTGTGGG-----------
-----TAGGGG-----TATGGG-----------TCGGGCGGTGAGTGC------------

------TGA-GGC-------------------ATG-GAGGGGGTCGGGGGCGT-------
-----------CTCCCCA---------------------------------GTCATGTGA
CTGCCGTGGTGACTGTGCACCTGACTGCCCCACATCAG--AGTCCCCAAACGCCCGATCA
TCAACACACCC

MARKER 169

>MIR3_MarsA

------------------------GGGAAGCAGCGTGGTACAGTGGAAAGAGCGCTGGAT
TTGGAGTCAGAGGACCTGGGTTCAAATCC---------------------------CGG-
-------CTC-TGCCACTTACTACCTG-TG--TGACCTTGGGC-AAGTCAC-TTAACCTC
TCTGGGCCTCAG-TTTCCTCATCTGTAAAATGAGGGGGTTGGACTAGATGACCTCTAAGG
TCCCTTCCAGCTCTAAATCTA-TGATCCTAT---GATCCTATGA----------------

>L1-4_ME_3end

----TTTTTATTTAAAATTTTGAGTTCCAAATTTTTCTCCCTCCCTCCCCTCCCCCCTCC
CTAAGAT---------GGTAAGCAATTTGATATAGGTTATACATGTGCAATCATGTAAAA
CATATTTCCATATTAGTCATGTTGTAAAAGAAGACACAGAATAAAAAAAAAA--------
--GTCTGCTTCATCTGCA
TTCMGATTCCACAGTTTTTCTCTGGATGTGGATAGCATTTTGCATCATGAGTCCTTTGG-
----------AAATGTTTTGGATCMTTGCATTGCTGAGAAGAGCTAAGTCTATCAAAAAY
AGTCMTCACACACTGTKGCTGTTACTGTGTACAATGTTCTCCTGGTTCTGCTCACTTCAC
TCAGCATCAGTTCATGTAAGTCTTTCCAGGTTTTTCTGAAGTCTACCTGTTCATCATTTC
TTATAGCACAATAATATTCCATTACATTCATATACCACAATTTGTTCAGCCATTCCCCAA
TTGATGGACATCCCCTCAATTTCCAGTTCTTTGCCACCACAAAAAGAGCTGCTATAAATA
TTTTTGTACATGT---

>Mdo
TTACCAAACAGAAGTTTGATAACCTCTACTGTTGCCGAGAGTCAATCTTGGATGG--GTA
AGTAGAGACTGCAAGTATCAGACCC----------------GTGAGAAA-GGGGAGAGAG
GGGTAGCTTAAAGCTTTCAGC---------------------------------------

----------------C-TTTGATCCTATTGGCACCAGCAGCCTTGCCTCACCTGGACTA
A-TAACTATTGCTTTCCTTTACTTTAAAT-GGGTGCCTGTGAAACCCCTTTCAAGAGACC
CTAGTTCAGTATCCTTTCCCTG----------------------AGGCACCTACCTCTGT
GAAA----------------TCCTCACA---CTAGGATTGCATTTGAATGGCACCTTCAT
CT-TT--TCCCACAG--CCTCAAGAGGACCACAGATG
>Dvi
TTACCAAACAGAAGTTTGATAACCTGTACTGCTGCCGAGAGTCAATCTTGGATGG--GTA
AGTAGAGACTGCAAGTGTCAGACCC----------------GTGAGAAAAAGGGGAGGAG
GGGTAGCTTAAAGGTTTCAGC---------------------------------------

----------------TTTGTGATCCTATTGGCACCAGCAGCCTTGCCTCACCTGGTCTA
A--CTCTATTGCTTTCCTTTACTTTGGAC-GGGTGCCTATGAAACCCCTTTCAAGAGACC
CTAGCTTGGTGTCCTTTCCCTG----------------------AGACACCTACCTCTGT
GAAA----------------TCCGCACA---CCAGGATTGCATTTGAATGGCACCTTCAT
CT-TT--TCCCACAG--CCTCAAGAGGACCACAGATG
>Mnu
TTACCAAACAGAAGTTTGATAACCTGTACTGCTGCCGAGAGTCAATCTTGGATGG--GTA
AGTAGAGACTGCAAGTGTCAGACCC----------------GTGAGAAAAAGGGGAGGAG
GGGTAGCTTAAAGGTTTCAGC---------------------------------------

----------------TTTGTGATCCTATTGGCACCAGCAGCCTTGCCTCACCTGGTGTA
A--CTCTATTGCTTTCCTTTACTTTGGAC-GGGTGCCTATGAAACCCCTTTCAAGAGACT
CTAGCTTGGTGTCCTTTCCCTG----------------------AGACACCTATCTCTGT
GAAA----------------TCCGCACA---CCAGGATTGCATTTGAATGGCACCTTCAT
CT-TT--TCCCACAG--CCTCAAGAGGACCACAGATG
>Rra
TCACCAAGCAGAAGTTTGATAATCTCTACTGCTGCCGGGAGTCCATTTTGGATGG--GTG
AGTAGAGACTGCAAGTCTCAGACCT-----------------GTGAGGA--GGGGAAGAG
AGAAAGTTTTAGCCACGGAA------------------------GCAAAGGAGGTGGATT
TGGGGGTCAGAGAATCAAG-GCC-------------------------------------
-TAAAG-CTA----------CCACCTACTCTCTG--------------------------
------CCTCGG-TTTCCTCACCTATAAAATGAATCATTGTA-CTAGATAGTGTCTGGT-
--CTCTTCTCCC--------------CCCTC-----------------------------

----------------TCAGT----CT-TAGGCAACAATGACCTCTCCTCTCCCAGTTTC
ACTCTTCATTGCCTTCCTTTCCATTGGGC-TGGTGCCTGTGAATTCC-------------
-----------------AGTTCAGGATCCT--GAGCCTGTG---ACCT----CCCTGTAT
GAAA---TCTACATA-----CCAGCATG---AACTCACAGGTTTTTAATGCAACCTTACT
CT-TT--TCCCTCAG--CCTCAAGAGGACCACAGATG
>Cfu
TCACCAAGCAGAAGTTTGATAATCTCTACTGCTGTCGGGAGTCCATTTTGGATGG--GTG
AGTAGTGACTGCAAGTCTCAGACCT-----------------GTGAGGA--GGGGAGGAG
AGAAAGTTTTAGCCACCGCA------------------------GCAAAGGAGGTGGG-T
TTGGGGTCAGAGAATCAAG-GCC-------------------------------------
-TAAAG-CTA----------CCACCTACTCTCTG--------------------------
------CCTCGG-TTTCCTCACTTATAAAATGAATCGTTGTA-CTAGATAGTGTTTGGTC
TCCTCTTCTCCC--------------CTCTC-----------------------------

----------------TCAGT----CT-TAGGCAACAAGGACTTCTCCTCTCCCAGTCTC

ACTCTTCATTGCCTTCCTTTCCATTGGGC-TTGTGCCTGTGAATTCC-------------
-----------------AGTTCAGGATCCTCTGAACCTGTG---AGCT----CCCTGTAT
GAAA---TATACATA-----CCAGCACG---AATTCACAGGTTTTAATGCAACCTTACT
CT-TT--TCCCTCAG--CCTCAAGAGGACCACAGATG
>Dgl
TTACCAAACAGAAGTTTGATAATCTGTACTGCTGCCGAGAGTCCATTTTGGATGG--GTG
AGTAGAGGCTGGAAGTATCAAACTT----------------GTGAGGAA--GGAGAGGAG
AGAGGGCTCGAAGCTGTCATG-----------------------GGAAAGGCTGTGGGCT
TGGG-GT--AGGGATCAGG-CCCAGGTC---------------------------TAGGT
ATGAAGCCTCATGC----CGCCACCTGCTCTGTGAC--------------TTGT-ACCTC
ACTTGACCTCAG-TTTC-TCATCTGTAAAATGGAAGGGTTGGACTAGATAGTGGCCAGTC
TCTTCT--AGCTCACATTCTTGTGATCCTCT-----------------------------

----------------TCACTGGTGCAGGAAGCAACAGCAGCCCTGTGTCTCCCAGTCTG
ACTCTTTTTTGCCTTCCTTTACTTTGGGC-AGGTGTCTGTA--------------AGATA
GCAGTTTGGTGTCTTCTACCTGAGGCACCCCGAGCCTATAAAAAGGGTCTGTCCCCACCA
TCCCCCCTGCACACAAGGATTGTACTTG---AATGAGCAGTGTTTGAAAGGAACCTTCAT
CT-TT--TTCCACAG--CCTCAAGAGGACCACAGATG
>Nty
TTACCAAACAGAAGTTTGATAACCTGTACTGCTGCCGAGAGTCCATTTTGGATGG--GTA
AGTAGAGACTGTAAGGAA--------------------------GGGGA--GGAGAGAAG
AGATTTCTGGAAGCTGTCATG-----------------------GGAAAGGTCATGGA-T
TTGG-GTCAAAGCATGATG-CCCAGGTC-------------------------TCTAGGT
ATGAGGCCTCATGC----TGTCCTCGGCT-------------------CATTTT-ACCTC
ACTTGACCTGAG-CTTCCTTATCTGTAAAATGAAAGGGCTGGACTAGCTGGTGTTTAGTC
TCTTTTCCAAGTCACAGTCTTGTGATCCCAT-----------------------------

----------------TACTTGGTACAAGTGGAAACGGCATACTTTCTTCACCCTGCCTC
ACTCTGTTT--CCTTCCTTTTCTTTGGGC-AGGCATCCATG--ACTTATTTCCAGAGACA
CCAGTTTGGGGTCCTCAATCTGAGGCCCTCCAGGCCTCTAA---GGCT-CCCCACTATGT
GAAA---AACACACAGGGATTGCATTTG---AATGGACAGTATTTGAATGGAACCCTCAT
CT-CTTTTTCCACAG--CCTCAAGAGGACCACAGATG
>Pta
TTACCAAACAGAAGTTTGATAACCTGTACTGCTGCCGAGAGTCCATTTTGGATGG--GTG
AGTAGACACTGCTGGTGTCAAACCT----------------GTGAGAAA--GGGGAGGAG

AGAGAGCTTGAAGTTGTTGTA-----------------------GAAAAAGCCATGAG-T
TTGAGGCCAGAGGATCAGG-CCCAGGTC---------------------------TAGAT
ATGAAGCCTTATGC----TGTCACCTGCTTTGGGACCCACAAGAAAGTCATTTT-ACCTA
ACTTGAGCTCAG-TTTCCTCATCTATAAACTGAAAAGATTGGGCTAGATAGTGTCAAGTC
CCTTTTTCAGCTTACAGTCTTGTGATACTAT-----------------------------

----------------TACTTGGTACCCTAACTGACTAAAGA------------------
-------ATAGCCACTCTTTACTTTGGGC-ATGTGTATGCC-------------------
-------------CTTTACCTGAGGCACCCTA------------GGCC-TTCTCTCATGT
GAAAA-CTGTACACTGGGATTGTATTTG---AATTAGCAGTATTTGGATGGAACCTTCAT
CT-TTT-TTTCACAG--CCTCAAGAGGACCACAGATG
>Dgo
TTACCAAACAGAAGTTTGATAACCTGTACTGCTGTCGAGAGTCCATTTTAGATGG--GTG
AGTAGACACTGCTAGTGTCAAACCT----------------ATGAGGAA--GGGGAGGAG
AGAGAGCTTGAAGTTGTTGTA-----------------------GAAAAAGCCATGAG-T
TTAAGGCCAGAGGATCAGG-CCCAGGTCTAGATATGAAGCCTTATGGCCCAGGTCTAGAT
ATGAAGCCTTATGC----TGCCACCTGCTTTGGGACCCACAAGAAAGTCATTTT-ACCTA
ACTTGACCTCAG-TTTCCTCATCTGTAAACTGAAAAGATTGGGCTAGATAGTGTCAAGTC
CCTTTTTCAGCTTACAGTCTTGTGATACTAT-----------------------------

----------------TACTTGGTACCCTAACTGACTAAAGA------------------
-------ATAGCCACTCTTTACTTTGGGC-ATTTGTGTGTC-------------------
-------------CTCTACCTGAGGCACCCTG------------GGCC-TTCCCTCATGT
GAAAG-CTGTACACTGGGATTGCATTTG---AATTAGCAGTATTTGGATGGAACCTTCAT
CT-TTT-TTTCACAG--CCTCAAGAGGACCACAGATG
>Scr
TTACCAAACAGAAGTTTGATAACCTATACTGCTGCCGAGAGTCCATTTTGGATGG--GTG
AGTAGACACTACTGGTATCAAACCT----------------ATGAGGAA--GGGGAGGAG
AGAGAGCTTGAAGTTGTTGTA-----------------------GAAAAAGCCATGAG-T
TTGAGGCCAGAGGATCAGG-CCCAGGTC----------------------------TGAT
A--AAGCCTTATGC----TGCCACCTGCTTTGGGACCCACAAAAAAGTCATTTT-ACCTA
ACTTGACCTCAG-TTTCCACATCTGTAAACTGAAAAGATTGGGCTAGATAGTGTCAAGTC
CCTTTTTCAGCTCACAATCTTGTAATACTGT-----------------------------

----------------TACTTGGTACCCTAACTGACTAAAGA------------------
-------ATAGCCACTCTTTACTTTGGGC-ATGTGCGTGTG-------------------
-----------TCCTCTAATTGAGGCACCCTG------------GGCC-TTCCCTCATGT
GAAAG-CTGTACACTGGGATTGCATTTG---AACTAGCAGTATTTGGATGGAACCTTCAT
CT-TTT-TTTCACAG--CCTCAAGAGGACCACAGATG
>Bil
TTACCAAACAGAAGTTTGATAATCTGTATTGCTGCCGAGAGTCCATTCTAGATGG--GTG
AGTGGAGACTGCAAGTGTCAAATCCATGAGGAGATGAAAATGTGAGGAA--TGGGAGGAA
AGATAACTTGAAGCTGTCATG-----------------------GGAAAGGCCATGGA-T
TTGAGATAAGGGGATCAGG-CCCAGGA---------------------------------
------------------------CTGCTCTGTGACCCACAAGAAAGTCATTTT-ACTCC
ATTTGGCCTCAG-TTTCCTCCTCTTTAAAATGAAAGGGTTGGACTAGATGGTATCTAGCC
TCTTTTCCAGCTCAATCTTA------TTCTC----------------TTTTTTTAATTTA
TTTTTATTAACAATATTATTTGAGTTTTACAATTCCCCCCCATCTTGCTTCCCTCCCCC
CACCCCACCCCCACGGAAAGCACTCTATCAGTCTTTACTTTGTTTCCATGTTGTACCTTG
ATCCAAATTG--GGTGTGATGAGCTAAATCATATCTTTAAAGAAGAAAAGAGAAGTCCTA
AGAGGTAACAAGATCAGACAATAAGCTATCTGTTTTTTTCTAAATTAAAGGGAATAGTCC
TTGCACTTTGTTCAAACTCCACAGCTCCTTATCTGGATACAGATGGCACTCTCCTTTGCA
GACAGCCCCAAACTGTTCCCGATTGTTGCACTGATGGAATGAGCGAGTCCTTCAAGGTTG
ATCATCACTCCCATGTTGCTGTTAGGGTGTACAG-TGTTTTCTGGTTCTGCTCATCTCAC
TCAGCATCAGTTCATGCAAATCCCTCCAGACTTCTCTGAAATCCTGTCCCTCCTGGTTTC
TAATANAGCAATAGTGTTCCATGACATACATATACCACAGTTTGCTAAGCCATTTCCCAA
TTGAAGGACATTTACTTGATTTCTGATTCTTTACCACCACAAACAGTGCTGCTATAAATC
AATCTTATTCTCT---TACTTGGGACAAATGACAACAGCAGCCTTTTCTCACTCTGTCTG
ACTCTTTATTGCCTTCTTTTACTCTGGCCCAGGTGGATGTGAATCCCATTTCAAGAGATG
CCAGTTTGGAGTCCTTTATTTGAGGCACCCCAAGCCTGGAA---GGC--T-CCCCTATGT
GAAAA-CTGCATACAGGGATTGTCTTTA---AATTAGCAGTATTTGAATAGAACCTTCAT
CTTTTTTT-CCACAG--CCTCAAGAGGACCACAGATG
>Pgu
TTACCAAACAGAAGTTTGATAACCTGTACTGCTGCCGAGAGTCCATTCTGGATGG--GTG
AGTGGAGACTG-------CAAATCCGTGAGGAGATG-AAGTGTGAGGAA--CAGGAGGAG
AGTTAACTTGAAGCTTTCGTG-----------------------GGAAAGGCCATGGT-T
TTGGGATTAGAGGATCAGA-CCCAGG----------------------------------
-----------------------ACTGCTCTGTGACCCACAAGAA-GTCATTTT-ACTTC
ATTTGGCCTCAG-TTTCCTCCGCTGTAAAATGAAAGGGTTGG-CTAGATGGTATCTGGTC
TCTTTTCCAGCTCA--GTCTTATC---CTTT-----------------------------

----------------TACTTGGTATAAATGACAACAGCAGCCTTTTCTCACTCTGTCTG
ACTCTTTATTGCCTTCTTTTACTCTGGCC-AGGTGGATGTGAATCCCATTTCAAGAGATG
CCNGTTTGGAGTCCTTTATTTGAGGCACCCCAAGGCTGGAA---GGC--TCCCTTTACGT
GACAA-CTGCACACAGGGATTGTCTTTG---AATTAGCAGTATTTGAATAGAACCTTCAT
CT-TTTTTTCCACAG--CCTCAAGAGGACCACAGATG
>Iso
TTACCAAACAGAAGTTTGATAACCTGTACTGCTGCCGAGAGTCCATTCTGGATGG--GTG
AGTGGAGACTG-------CAAATCTGTGAGGAGATG-AAGTGTGAGGAA--CAGGAGGAG
AGATAACTTGAAGCTGTCGTG-----------------------GGAAAGGCCATGGT-T
TTGGGATTAGAGGATCAGA-CCCAGGA---------------------------------
-----------------------CTGCTCTGTGACCCACAAGAAAGTCATTTT-ACTTC
ATTCGGCCTCAG-TTTCCTCCGCTGTAAAATGAAAGGGTTGGACTAGATGGTATCTGGTC
TCTTTTCCAGCTCA--GTCTTATGATCCTTT-----------------------------

----------------TACTTGGTATAAATGACCTCAGCAGCCTTTTCTCACTCTGTCTG
ACTCTTTATTGCCTTCTTTTACTCTGGCC-AGGTGGATGTGAATCCCATTTCAAGAGATG
CCAGTTTGGAGTCCTTTATTTGAGGCACCCCAAGGCTGGAA---GGC--TCCTTTTACGT
GAAAA-CTGCACACAGGGATTGTCTTTG---AATTAGCAGTATTTGAATAGAACCTTCAT
CT-TTTTTTCCACAG--CCTCAAGAGGACCACAGATG
>Tro
TTACCAAACAGAAGTTTGATAACTTGTACTGCTGCCGAGAGTCCATTTTGGATGG--GTG
AGTATTAGACCTCTGCGAAA-------------------------------GGGGAGGAG
AGAGAACTTGGAGCTGTCATG-----------------------GGGAA-ACCATAGG-T
TTGGGGTCTGAGAGTCAGG-CCCAGGCCC---------------------------AGG-

--TCTAGAGTCTCACTTC
CCTCTTCCAGCTCACAATCTTTGGATCCTCC-----------------------------

----------------TCCTTGGCACAAG-GGAAAGAGCAG-CTTTCCTCACCCTTTCT-
---CTTTATTGCTTTCTTTTATTTTGGGC-AGGTGTCTGTGAATCCCATTTCAAGGGACG
CCATTTTGGGGTCCTCTACCTAAGACACCCCGAGCCTGTAA---GGC--TCCCCCAGTGT
GAATG-CTGCACGGG-GCATTACATTTG---AATTG---GCATTTGAATGGAGCCTTCAT
CT-TTT-TTCTACAG--CCTCAAGAGGACCACAGATG

```
>Ppe
TCACCAAGCAGAAGTTTGATAACTTGTACTGCTGCCGAGAGTCCATTCTAGATGG--GTG
AGTAAAGTCTGGCAGTATCAAACCT----------------CTGAGGAA--GGGGAGGAG
AGAA--CTCGAAGCTATCATG-----------------------GGAAAGACCATGGG-C
TTGGGGTCCAGGTATGGAG-TCTCATGC--------------------------------
------------------TGCCACCTGCTCTGTGACCCACAAGAAAGTCATTTT-ACCT-
ACTTGGCCTCAG-TTTCCTCATCTGTAAAATGAAAAGGCTGGACTAGATGGTGTCCAGTC
TCTTTTCCAGCTCACAGTCTTGCGATCTTAT-----------------------------
------------------------------------------------------------
------------------------------------------------------------
------------------------------------------------------------
------------------------------------------------------------
------------------------------------------------------------
------------------------------------------------------------
------------------------------------------------------------
------------------------------------------------------------
------------------------------------------------------------
------------------------------------------------------------
----------------CACTTGGTACAAGTGACAACAGCAGCCTTTTCTCATCGTATCT-
---CTTTATTGCTTTCCTTTACTTTGAGC-AGATGTCTGTGAATCCTATTTCAAGGGACA
CCAGTTTGGGGTCCTCTACCTGAGGCACCCTGAGCCTGTAA---GGC--TCCCCCAATGC
GAAAG-CTGCATGCAGGGATTACACTTG---AATTAGCAGCATTTGAATGGAGCCTTCAT
CT-TTT-TTCCACAG--CCTCAAGAGGACCACAGATG
>Tvu
TTACCAAACAGAAGTTTGATAACTTGTACTGCTGCCGAGAGTCCATTTTGGATGG--GTG
AGTAGAGACTGCAGGTGTCAAACCT----------------GTGAGGAA--GGGGAGGAG
AGAGAACTTGAAGGTGTCATG-----------------------GGAAAGACCATGGA-T
TTGGGGTCAGAGGATCAGG-TCCAAGTCC---------------------------AGGT
ATGAAGTCTTGTGC----TGCCACCTGCTTGATGACCTC----------------ACTTG
ACCTCAG------TTTCCTTATCTGTAAAATGAAAGGGCCGGACTATATGGTACCCAGTC
CCTTTTCCAGGTCACAGTCTTGTCATCCTGT-----------------------------
------------------------------------------------------------
------------------------------------------------------------
------------------------------------------------------------
------------------------------------------------------------
------------------------------------------------------------
------------------------------------------------------------
------------------------------------------------------------
------------------------------------------------------------
------------------------------------------------------------
------------------------------------------------------------
----------------TACTTGGCATAAGTGATGATAGCAGCCTTTCCTCACACTGTCTG
ATTCTCTATTGCTTTCCTTCACTTTGGGC-AGGTGTCTGTGAATCCCATTTCAAGAGACA
CCAGTTTGGGGTCTTCTACCTGAGGCACC-TGAGCCTATAA---GGC--TCCCCCAATGG
GAAAG-CTGCACGCAGGGATTGCATTTG---AATTAGCAGTATTTGAATGGAGTCTTCGT
CT-TTT-TTCCACAG--CCTCAAGAGGACCACAGATG
>Mro
TTACCAAGCAGAAGTTTGATAACTTGTACTGCTGCCGAGAATCCATTTTGGATGG--GTA
AGTAGAGACTGCAAGTATCCAACCT----------------GTGAGGAA--GGGGAAGAG
AGAGAACTTGAAGCTGTCATG-----------------------GGAAAGACCATAAA-T
TTGGGGTCAGAGGATCAGG-TTCAGGTCC---------------------------AGGT
ATGAAGTCTCATGC----TGCCACCTGCTC--TGACCCACAAGAAAGTCATTTTTACTTC
ACTTGACCTCTTATTTCCTTATCTGTAAAGTGAAAGGACTGGACTAGATGGTGTCCACTC
```

TCTTTTCCAGCTCA--GTCTTGTGATCCTGT-----------------------------

----------------CACTTCNTACAAATGACAGCAGCAACCTTTCCTCACCATCT--G
ACCCTTTA-----TTACTTTACTTTGGGC-AG--GTTTCTGAATCCCATTTCAGGAGACA
CCAGTTTGGGGTCCCCTACCTAAGGCACCCTGATCCTATAA---GGC--TCCCCCAGTAT
GAAAG-CCGCACACAGG-ATTGCC-TTG---AATTAGCAGTATTTGAATGGAGCCTTCAT
CT-TTT-TTCCACAG--CCTCAAGAGGACCACAGATG
>Vur
TTACCAAACAGAAGTTTGATAACTTGTACTGCTGCCGAGAGTCCATTTTGGATGG--GTG
AGTAGAGACTGCAAGTATCAAACCT----------------ATGAGGAA--GGGGAGGAG
AGAGAGCTTGAAGCTGTCATT-----------------------GGAAAGGCCCTGGG-T
TTGGGGTCAGAGGATCAGG-CCCAGGTCC---------------------------AGGT
GTGAAGCCTTATGC----TGCCACCTGCTCTGTGACCCACAAGAAAGTCATTTT-ACCTC
ACTTGACTTCAC-TTTGCTCATCTGGAGGCTA-AAGGGTTGGACTGCATGGTGTCCAGTC
TCTTTTCCAGCTCA-AGTCTTGTGATCCTGT-----------------------------

----------------CACTTGGTACAAGTGACAAGAGCAACCTTTGTTTACCTTGTCTG
ACTCTTTATTGCTTTTCTTTACTTTGGGC-AGGTGTCTGTGAATCCCATTTAAAGAGACA
GCAGTTTGGGGTCCTCTAGCTGAGGCACCCCGAGCCTGTAA---GAC--TCACCCTATGT
GACAG-GTGCACAGAGTGATTGCATTTG---AATTAGCAGTGTTTGAATGGAGCCTTCAT
CT-TTT-TTCCATAG--CCTCAAGAGGACCACAGATG
>human
TTACCAAACAGAAGTTTGATAACTTGTACTGCTGCCGAGAATCCATTTTGGATGG--GTA
GGTTGAATGTATATATATTCAAACT----------------TCTAGGGG-----------


```
------------------------------------------------------------
------------------------------------------------------------
------------------------------------------------------------
------------------------------------------------------------
----------------------------------------------------------CT
CTGGTCTT-----CCTTTGGCTTTAAAAG-TTT-ATTAGAGGGA-CCATT---TCATACT
GAGCTCAACCAGCTTCCAATTGATATTCTCATGGGCATT-----GGC----CCACACTTT
TAACT-TTGTGAAGAGGGACTGAGTTTGAGCTATTAACG---TTTGAGTTGAGCCCTTGT
CTGTTT---CCACAG--CCTGAAGAGGACCACAGATG
```

MARKER 172

>MIR3_MarsB

-------GAGAAGCAGCATGG-TGTAG---------------------------------

------------------------TGGATAGAGCAC------TGGACTTGGAGTCAGGAA
GACCTGGGTTCAAATCCCGC-CTCAGACACTTACTAGCTGTGTGAC-CCTGGGCAAGTC-
AC--

--TTA---AC
C---

---TCTCTGGGCCT
CAGTTTCCTCATCTGTAAAA-------TGAGGGGGTTG----------------------

---------------GACTAGAT---GGCCTCTAAGGTCCC---TTCCAGCTCTAAAT--

>WSINE1

------------------------------TTTTT--------TTTTTTTTTGAGGCAAT
TGGGGTTAAGTGACTTGCCCAGGGTCACACAGCTAGT-------AAGTGTCTGAGGCTGG
ATTTGAACTCAGGTCCTCCTGACTCCAGGGCTGGTGCTCT-ATCCACTGCACCACCTAGC
TGCCCCTC--

>MAR1b_Mdo

------------------------------------TTGTTGTTGTTGTTCAGTCGTTTT
CAGTCATGTCTGACTCTTCGTGACCCCATTT-----------GGGGTTTTCTTGG-----
-------CAAAGATACTGGAGTGGTTT-GCCATTTCCTTCTCCA------------GCTC
ATTTTACAGATGAGGAAACTGAGGCAAACAGGGT-------------TAAGTGACTTGCC
CAGGGTCACACAGCTAGTAAGTGTCTGAGGCCAGATTTGAACTCAGGTCTTCCTGACT-C
CAGGCCCGGCGCTCTATCCACTGTGCCACCTA-GCTGCCCTC------------------


```
-------------------------------------
>SINE1_Mdo
------------------------------------------------------------
------------------------------------------------------------
------------------------------------------------------------
------------------------------------------------------------
------------------------------------------------------------
------------------------------------------------------------
------------------------------------------------------------
------------------------------------------------------------
------------------------------------------------------------
------------------------------------------------------------
------------------------------------------------------------
------------------------------------------------------------
------------------------------------------------------------
------------------------------------------------------------
------------------------------------------------------------
--------------------------------------------TTTTTTTTTTTTT---
AAACCCTTA-CCTTCCATCTTGGAATCAATACTGTGTATTGGTTCCAAGGCAGAAGAGTG
GTAAGGGCTAGGCAATGGGGGTCAAGTGAC-TTGCCCAGGGTCACACAGCTGGGAAGTGT
CTGAGGCCAGATTTGAACCTAGGACCTCCCATCTCTAGGCCTGGCTCTCAATCCACTGAG
CTACCCAGCTGCCCCC--------------------------------------------
------------------------------------------------------------
------------------------------------------------------------
------------------------------------------------------------
------------------------------------------------------------
------------------------------------------------------------
------------------------------------------------------------
------------------------------------------------------------
------------------------------------------------------------
------------------------------------------------------------
------------------------------------------------------------
------------------------------------------------------------
------------------------------------------------------------
------------------------------------------------------------
-------------------------------------
>Mon1
------------------------------------------------------------
------------------------------------------------------------
------------------------------------------------------------
------------------------------------------------------------
------------------------------------------------------------
------------------------------------------------------------
------------------------------------------------------------
------------------------------------------------------------
------------------------------------------------------------
------------------------------------------------------------
------------------------------------------------------------
------------------------------------------------------------
------------------------------------------------------------
------------------------------------------------------------
------------------------------------------------------------
------------------------------------------------------------
```


-------------GGAAAGAGCACGGGCCTGGGAGTCAGAGGACCTGGGTTCTAATCCCG
GCTCTGCCACTTGCCTGCTGTGTGACCTTGGGCAAGTCACTTAACTTCTCT---GTGCCT
CAGTTTCCTCANCTGTAAAA-------TG-------------------------------

>RTE-2_MD

----------TTGGTTGGTTGTTGTC----CTTCGTACTCGAAGAGGACCAAAATGACAT
CACTATGTCGGGGTCAATGTACAGTGTGTCCGACTGTGGCTGATCAGACCAATATGAGCT
CGGAAGGCTCTACCACAGGTTGGGCACAAATAGCCCGTATGAACATTTGGGA-TGGAGAT
GTCTCTAAATTTGCGCATCTCACGTTTCTTTTG--AGCT---------------------


```
>DNAT_ME
------------------------------------------------------------
------------------------------------------------------------
------------------------------------------------------------
------------------------------------------------------------
------------------------------------------------------------
------------------------------------------------------------
------------------------------------------------------------
------------------------------------------------------------
------------------------------------------------------------
------------------------------------------------------------
------------------------------------------------------------
------------------------------------------------------------
------------------------------------------------------------
------------------------------------------------------------
------------------------------------------------------------
------------------------------------------------------------
------------------------------------------------------------
------------------------------------------------------------
------------------------------------------------------------
------------------------------------------------------------
------------------------------------------------------------
------------------------------------------------------------
------------------------------------------------------------
------------------------------------------------------------
------------------------------------------------------------
------------------------------------------------------------
------------------------------------------------------------
----------------------------------------------------CAGGGGTG
GGGAACCTGCAGCCTCGAGGCCACATGTGGCCCTCTAGGTCCTCAAGTGCGGCCCTTTGA
CTGAATCCAAACTTCACAGAACAAATCCCCTTAATAAAAGGATTTGTTCTGTAAAACTTG
GACTCAGTCAAAAGGCCACACCCAAGGACCTAGAAGGCCACATGTGGCCTTGAGGCTGCA
GGTTCCCCACCCCTG---------------------------------------------
------------------------------------------------------------
-------------------------------------
>Mdo
GGCCATG---GAAACAGCAGTTCTCAGGCAACAATATCAAAGGAATCAGCTTGCCCTTTA
AAC-CAGTCATCTCATCAGCCCTTCTCTTTTCAAAGAGATTAGTGGCAGCAGTCTCAACC
-TACAGGCATTTGGTTGGCCT--AAGGAAAGTCAATTCTTTGATGTGT------------
-------GAG--GCTGCATGGGCATAG----------------------------------
------------------------------------------------------------
------------------------------------------------------------
------------------------TTGACAGAATTC------AAGATTGAGAGTCAGGAA
GACCTGGGTTTAAATCCCC-ATTCTGACACTTACAGACTGTGCGACTACTCATCCTCTCT
GC----------------------------------------------------------
------------------------------------------------------------
------------------------------------------------------------
------------------------------------------------------------
------------------------------------------------------------
---------------------------------------------CA-AGACTC----AT
CTACTCCATTATAGACAGATTACAT----------------------CTGTGTTGGTAAA
GGGAAT---TCCAGGACCAATGAA----TTTGCATTTTGG------TTTTTTTTTTT---
AAACTCTTA-CCTTCCGTCTTAGAATCAATACTACACGTCAGTTCCAAGGCAGAAGAGCA
```

```
TTAAGGGCTAAGCAATAGGGGTTAAATGATTTTGCCCAGTGTCACAAAGCTAGGAAGTGT
CTGAGGCCACATTTGAATCCAGGACCTCC-------AGGCCTGGCCCTCTGT-CACTGTG
CTACCTAGCTGC-------TCCTAAATTCTTGAT---TGTCTCTA---------------
----------------------AATGTCTTATCTTTTCAGTTGTA---AGACCA--TTTC
CCTC-T------------------------------------------------------
------------------------------------------------------------
------------------------------------------------------------
------------------------------------------------------------
--GATTTTTCACAGG-AAGAGCCCTAGGCTAGAATTCAAAAGATCTGAGTTCTAATTCCA
GTTTAGCTGCTCATAGACTTGAT-------------CTTTGTC--TTTGATTTTGCTTCT
CAGTTTCCTTCACTATAAAA-------TGATGAGTTTG----------------------
------------------------------------------------------------
------------------------------------------------------------
------------------------------------------------------------
---------------GACTAGGG---TAGCCTTAAAATCCT---TCAAAACCCCAGCA--
-----------TTCTTGGCATGGTTCTTTGTATGTGACATT-GCTTAAGTGTACATGTTT
GTTCTTAG--GGTTATGAGATACGTATGTATGACAGC
>Dvi
GGCCATG---GAAGCAGCAGTTCTCAAGCAACAATATCAAAGGAATCAGCATGCCCTATA
AAC-CAGTCATCTCATCAGCCCTTCTATTTCCAAAGAGATTAGTGGCAGCAGTCTCAACC
-TACAGGCATTTGGTCAGCCT--AAGGAAAGTCAATTCTTTGATGTGATAACCTTAAAAT
GTGT---GAG--GCTGCATGGGTATAG---------------------------------
------------------------------------------------------------
------------------------------------------------------------
------------------------GGGACAGAATTC------AAGATTGGGAATCAGGAA
GATCTGGGTTTAAATTCC-CATTCTGACACTTACAGGCTGTGCAACTACTCGGCCTCTCT
GC----------------------------------------------------------
------------------------------------------------------------
------------------------------------------------------------
------------------------------------------------------------
------------------------------------------------------------
------------------------------------------------------------
------------------------------------------------------------
-----------CAAGAC----TCA----TTTGCATTTTCTTTC-TTTTTTTTTTTTTAAC
AAACTCTTACCCTTCCATCTTAGAATCAATACTATGCATCAGTTCCAAGACAGAAGAGTA
TTAAGGGCTA-GCAATAGGGGTTAAATGACTTTATGCAGTGTCGCACAGCTAGGAAGTGT
CTGAGGCCACATTTGAACCCAGGATCTTC-TATCTTCAGGCCTGGCCCTCTGTCACTGAG
CCACCTAGCTGC-------TCCTAA------------ATTATATATTCTTGGAATACTGT
TGGAATCCTGTATT-GTATCTTAATGTCTTATCTTTCCAGTTATA---AGACCA--TTTT
CCTCTGA-----------------------------------------------------
------------------------------------------------------------
------------------------------------------------------------
------------------------------------------------------------
--CACTTTTCACAGG-AAGAGCCCTAGGCTAAAAGTCAAAAGATCTGAGTTCTAATTTGA
GCTTAACTGGTCATAGACTTGAT-------------CTTTGTT--TTTGACTTTGCTTCT
CAGTTTCCTTCACTATAAAA-------TGATGGGTTTG----------------------
------------------------------------------------------------
------------------------------------------------------------
------------------------------------------------------------
---------------GACTAGGG---CATCCTTAAAATCCT---TGAAAATCCCAGCA--
-----------TTCTTGGCATAGTTCTTTGTATGTGACATT-GCTTAAGTGTACATGTTT
ATTGGTAG--GGTTATGAGATACGTATGTATGACAGC
>Mnu
```

GGCCATG---GAAGCAGCAGTTCTCAAGCAACAATATCAAAGGAATCAGCATGCCCTATA
AAC-CAGTCATCTCATCAGCCCTTCTATTTCCAAAGAGATTAGTGGCAGCAGTCTCAACC
-TACAGGCATTTGGTCAGCCT--AAGGAAAGTCAATTCTTTGATGTGATAACCTTAAAAT
GTGT---GAG--ACTGCATGGGTATAG---------------------------------

------------------------GGGACAGAATTC------AAGATTGGGAATCAGGAA
GATCTGGGTTTAAATTCC-CATTCTGACACTTACAGGCTGTGCAACTACTCGGCCTCTCT
GC--

-----------CAAGAC----TCA----TTTGAATTTTCTTTCTTTTTTTTTTTTTTAAC
AAACTCTTACCCTTCCATCTTAGAATCAATACTATGCATCAGTTCCAAGGCAGAAGAGTA
TTAAGGGCTA-GCAATAGGGGTTAAATGACTTTATGCAGTGTCACACAGCTAGGAAGTGT
CTGAGGCCACATTTAAACCCAGGATCTTC-TATTTTCAGGCCTGGCCCTCTGTCACTGAG
CCACCTAGCTGC-------TCCTAA------------ATTATATATTCTTGGAGTGCTGT
TGGAATCCTGTATT-GTATCTTAATGTCTTATCTTTCCAGTTATA---AGACCA--TTTT
CCTCTGA---

--CACTTTTCACAGG-AAGAGCCCTAGGATAAAAGTCAAAAGATCTGAGTTCTGATTTGG
GCTTAACTGGTCATAGACTTGAT-------------CTTTGTT--TTTGACTTTGCTTCT
CAGTTTCCTTCACTATAAAA-------TGATGGGTTTG----------------------

---------------GACTAGGG---CATCCTTAAAATCCT---TGAAAACCCCAGCA--
-----------TTCTTGGCATAGTTCTTTGTATGTGACATT-GCTTAAGTGTACATGTTT
ATTGGTAG--GGTTATGAGATACGTATGTATGACAGC
>Rra
AGTCATG---GAAGCTGCTGTCCTTGAGCAGCAATAGCAG-----TCAGCTTGCCTGCTA
CAC-TCATCATCTCAAAGGCCCTTCTCTTCCC-TCAAAATCACTAGCAGTTGTGTGAACT
-GAAAGGCTTTTCCTTGGCCT--GTGGAAAGTCAGTTTTTGGATGTGTTAGCCTTAAAAA
GTAT---GAG--AC-ACTATAGCATAG---------------------------------

------------------------TTGATAGAGTTC------AAGATTTGTAGTCAAAAG
GAC-----------TCTTT-ACTTTAACATTTAGAAGCTATGTGACCATACATCCTCTCT
GAACCTGAGGCAACTAATTCC---CCAAGACCT----TGTTGTT---GTTCAGTCATTTT
-AGAGACATCTGACTCTTTGTGGCCCCTATAGCCTTTTATCTGTGTGTTG------TTTT
TTTTTAACAAGGACATTGGAGTGGTTTTTCTATTTCTTTGTCCTGGGATTCATGAAAGGC
ACTT-ACAGTCATTACTGTATTTGCATTTCAAATACAAGTCCCCTCATAAGTGACTTCTC
CAGGGTCACATAGCTAGTATGTGTCTGAAGCTGGATTTAAACTCAGGTTCTCCTGGCTAA
TAAACCCAGTGATCTGCCCATTGTACCACCTAAGCTGCCCAC---CTGAGACTT----AC
CTTCTGAGTTGTAGACAGATTTTAC----------------------CTGCCACCATAAA
GAGAAT---TCCAGTAAC--

-------------------------------------ACTACATATACTTGACATACCAT
TGTAACCCTATATT-GTATCCTAATGTCTCATCTTTCCAATTATG---AGACCA--ATTT
CCCTAT--

--GACTTTTCA-TGGAAAAACCAAA-GGCTAGAAATTAAGAG-------TTCTAATGCCT
ACTTAGCTGGCTTTGGACTTGGT-------------CTCTGACCAGATAACTGTGCTTCT
TAGTGTCTTTCTCTAT-AAA-------TGATGAGTTTG----------------------

---------------GACGTGTG---TATCTTTAAGGTCCC---TTAAAACTCCCACA--
-----------TTCTTGGTCTGGTCCTTTATGTGTGATATTTCCTTATGTGCACCTTCTT
GCTCTTAG--GGTTATGAGATACGCATGTATGACAGT
>Cfu
AGCCATG---GAAGCTGCTGTCCTTGAGCAGCAGTATCAGAGCAATTAGCTTGCCCACTA
CAC-TCATCGTCTCATCAGCCCTTCTCTTCCC-TCAAAATCACTAGCAGCTGTGTGAACT
-GAAAGGCTTTTCCTTGGCCT--GGGGAAAGTCAGTTCTTGGATGTGTTAGCCTTAAAAA
GTGT---GAG--AC-ATTATAGCATAG----------------------------------
----

------------------------TTGATAGAGTTC------AAGATTTGTAGTCAAAAG
GAC-----------TCCCT-ACTCTAACATTTAGAAGCTGTGTGACCGTACATCCTCTCT
GAAACTCAAGCAACTAACTCC---CCAAGACTT----TGTTGTT---ATTCACTTGTTTT
-AGAGACATCTGACTCTTTGTGGCCCCCATAGCCTTTTGTCTGTGGTTTTTTTTGTTTTT
TTTTTAACAAGGACATTGGAGNGTTTTTGCCATTTCTTTGTCCTGGGATTCATGAAGGGC
ATTT-ACAGTCATTACTGTATTTGCATTTCAAATACAAGTCCCCTC-TAAGTGACTTCTC
CAGGGTCACATAACTAGTATGTGTCTGAAGCTGAATTTAAACTCAGGTTCTCCTGGCTAA
TAAACCCAGTGCTCTGCCCACTGTACCACCTAAGCTGCCCAC---CTGAGACTT----AC
CTTCTGAGTTGTAGACAGATTTTAC----------------------CTGCCACCATAAA
GAGAAT---TCCAGTGAC--

-------------------------------------ACTGCATATACTTGACATACCAT
TGTAAACCTATATT-GTATCCTAATGTCTCATCTTGCCAATTATG---AGACCA--ATTT
CCCTAT--

--GACTTTTCA-TGGAAAAACCAAA-GACTAGAAATT--GAG-------TTCTAATGCTT
ACTTAGCTGGCTGTGGACTTGGT-------------CTCTGACCAGATAACTTTGCTTCT
AAGTGTCCTTCCCTAT-AAA-------TGATGAGTTTG----------------------

---------------GACGTGTG---TATCTTTAAGGTCCC---TTAAAACTCCCACA--
-----------TTCTTGGTCTGGTCCTTTGGGTGTGATATTTCCTTATGTGCACCTTCTT
GCTCTTAG--GGTTATGAGATACGCATGTATGACAGC
>Dgl
GGCCATG---GAAGCAGCTGTCCTCCAGCAATAGCATCAAAGGAATCAGCTCGCCCTATA

AAC-CAGTCATCTCATCAGCCTTTCTCTTTCCAAAGAGATAAGTGGCAGCAATTTGAACT
-TAAAGGCATTTGGTTGGCCT--CAGGAAAATCATCTCTTTGGTGTGGTAACCTTAAAAT
GCTT---GAG--GCAATATGG-CATAG---------------------------------

------------------------TTGATAGACTTT------AAGACATGGAGTCGAAAA
GAA---------AATCCCC-ACTCTAACTCTTAGGGGCTGTGTGACTTCTCACTCTCTTT
-C--

--TAAC-ACTTA---AC
CTGTTCAGT----GATAGATTAGAT----------------------TTGTGTTGGCAAA
GGGAAT---TCCCACACCAGTGAA-----------------------------------

------------------------------------ATTGCATGTTCTTGATGTGTTAT
TGTAA--CTATATT-ATATCCTAATGTCTCCATTTTCTAATCACG---AGACCA--TTTT
CTCTAC-----TGGTTGGTTGTTGTC----CTTTGTTCTCAAAGGGGATCAAAATGACAT
CACTATGTTGGGGTCAAAGG-CAGTGTGTCCCATTGTGTTTGATCAGACCAAGATGAGCT
CCAAAGGCTCTGGCACAGGTGGGACACAAGTAATCCATGTGAACATTTAGAG-TGGAGAT
GTCTCTAAATTGACACATCTCCTGTTTCTTTTG--AGCT---ACT-GTTTTTTCCTTAA-
--TACTTTTCA-TGGAAAGAGTACTAGGCTAGAAGTCAAGAGATCTGAGTTCAA-TCCCA
GCTTAGCTTACCATGCACTTGAT-------CTCTGTCTTTGACCAGATCACTTTGCGCCT
CAGTTTCCTTCACTATAAAA-------TGATGGGTTTG----------------------

---------------GACCAGAA---CATCTTTGAGGTCCC---TTAAAACTTTAACA--
-----------TTCTTGGTATGGTTCTTTGTTCGTGATATTTCCTCAAGTCTACATGTTT
GCTCTTAG--GGCTACGAGATACGCATGTATGACAGT
>Nty
AGTCACA---TAAGCAGCTGGCCTCAAGCAACAATATCAAAGGAATCAGCTTGCCCTATA
AAC-CAGTCATCTCATTAGCCCTTTTCTTTCCATAGAGATAAATGGCAGCAATTTGAACT
-TAAAGGCATTTGGTTGGCCT--AAGAAAAGTCAGTTATTTGAGGTGGTAACCTCAAAAT
CTTT---GAG--GC-AATAGGGCATAG---TTTTTTTTAAAAATTATTTTACAAGGCAAT
AATGGTTAAGTAATTTGCCCAAGGTCACACACCTTGTAAGTATCAAGTGTCAGAGGCTGA
ATTTGAACTCAGGTCCTCCTGACTCCAGGGCTGGTGTTCTTATTTCCTGTTCCATCTAGC
TACCCC---GGTATGACATAG---TTGATAGAGTTC------AAGACTTAGAGTCCAAAA
GGA-TATGTTTAAATTGCC-ACTCTGATACGCAGGGGCTTTGTGACTCCTCAACTATTCT
GC--

--TTAG-ACGT----AC
CTGATCAGTTATAGACAGATTACAT----------------------TTGCATTGGTAAA
GGGAAT---TCCCACGCTAGTTAA-----------------------------------


```
-------------------------------------ATTGCATGTTCTTGATATATTTT
TATAACTTTGTATT-GTTTTCCAATGTCTGCACTTTCTAATTATG---AGACCA--TTTT
TCCTAT-----TGGTTGGTTGTTGTC----CTTTGTTCTTGAAGAGGACCAAAA-----C
CATTAGGTGGGGGTCGAGGTACAGTATGTCTGACTGTGTCTGATCCGACCAATAGGACCT
CGGACATCTCTGCAGAA-TTTGGGCACAAATAGTCCATATAATTATTTAGG--TGGAAAT
GTCTCTAAATTTGGGCGTCTCATGTTTATTTTG--AGCT---ACTACATTTTTCCT-AC-
--TGCTTTTCA-TGGAAAAAGCACTAGGCTAGATATGAAGAGATCTGCGATCTAATCCCA
GTTTTGCTAGCCATGGACTCAAT-------ATGTGTCTTTGACCAGATCACTTTGTTTCT
CAGTTTCCTTCACTATAAAG-------TGATGGGTTTG----------------------
------------------------------------------------------------
------------------------------------------------------------
------------------------------------------------------------
---------------GACCAGGA---TAACTTTGAGGTCCC---TTAAAACTCCAAACA-
-----------TTCTTGCTATGGCTCTTTGTATATGATATTTGCTC--ATCTACATGTTT
GCTCTTAG--GGTTATGAGATACGCATGTATGACAGT
>Pta
A-----------------------------------------------------------
------------------------------------------------------------
------------------------------------------------------------
------------------------------------------------------------
------------------------------------------------------------
------------------------------------------------------------
------------------------------------------------------------
--ACTGGATTTAAATCC---------------TAGGCCTGTGTGAGAACTAAACCTTCCT
GC----------------------------------------------------------
------------------------------------------------------------
------------------------------------------------------------
------------------------------------------------------------
------------------------------------------------------------
---------------------------------------------TAAA-ACTT----AC
CTGTCCAGTTACAGACAGGTTACAT----------------------TTGCATCGGAAAA
GGAAAT---TCTCATGTCAGTTAA------------------------------------
------------------------------------------------------------
------------------------------------------------------------
------------------------------------------------------------
-------------------------------------ACTGCACTTAT-TGATGTGTTAT
TGGTACTCTATATT-GTATTCTCATGTCCCTACTTTCCCGTTATG---AGGTCA--CTCT
CCCTAG-----GGGTTGGGTATTG-C----CATCTTGATTCTCTGAGATCC-AAGGACAT
CACCACGTCGGGGTCTCGGTGCAGTGTGCCCAACTGTGGCTCATCAGACCATTATGAGTT
CCGAGATCTGTACCACAGATTGGGCACAAATAGTCCATATGAATATT-AGG--TGGAAAT
GCCTCTAA-TTTGCATGTTTCATGTTTCTTTTG--AGCT---ATTGCA-TTTCCCCTAA-
--GTCTTTTCA-TGGAAAGAGCACAAGGCTAGAAGA-------TCTGAGTTCTAATCTCA
GCTTAGTTAGCCATAGACTCGAT-------CTATGTCTTTGCCTAGATCATTTTGCTCCT
CA-TTTACTTCACTATAAAA-------TGATGGGTTCG----------------------
------------------------------------------------------------
------------------------------------------------------------
------------------------------------------------------------
---------------CATCAGGA---CATTTTTGAGGTTCC---TTAAAACTCCAACC--
------------TCTTGCTATGGTTCTTTCTTTGTGATATATCCTC--ATCTACATGTTT
GCTCTTAG--GGTTACGAGATCCGTATGTATGACAGT
>Dgo
A-----------------------------------------------------------
------------------------------------------------------------
```


--ACTGGATTTAAATCC---------------TAGGCCTGTGTGAGAACTAAACCTTTCT
GC--

---TAAA-ACTT----AC
CT-TCCAGTTACAGACAGGTCATAT----------------------TTGTATCGGAAAA
GGAAAT---TCTCACATCAGTGAA------------------------------------

-------------------------------------ATTGTACTTACTTGATGTGTTAT
TGGCACTCTGTATT-GTATCCTCATGTCCCTACTTTCCAGTTATG---AGGTCA--TTCT
CCCTAC-----GGGTTGGTTATTGTC-----ATCTTGATCCTCTGAGATC-AAAGGACAT
CCCCATGTCGGGGTCTCGGTGCATTGTGCCCAACTGGGGTTCATCAGACCATTGTGAGCT
CTGAGATCTCTACCATAGTTTGGGTACAAATAGTCCATATGAATATT-ATG--TGGAAAT
GTCTCTAA-TTTTCATGTTTCATGTTTCTTTTG--AGCT---ATTACA-TTTTCCTTAA-
--GTCCTTTCA-TGGAAGGAGCACAAGGCTAGAAGA-------TCTGAGTTTTAATCTTA
GCTTAGCTAGCCATAGACTTGAT-------CTATGTCTTTGCCTAGATCGTTTTGCTCCT
CATTTTCCTTCACTATAAAA-------TGATGGGTTTG----------------------

---------------GATCAGGA---CATTTTTGAGGTTCC---TTAAAACTCCAAC---
-----------TTCTTGATGTGGTTCTTTGTTTGTGATATGTCCTT--GTCTACATGTTT
GCTCTTAG--GGTTACGAGATACGTATGTATGACAGT
>Bil
AGCCGCCATGTAAGCAGCTGTCCTCAAGCAGCTGTGTCAAAGGAATCATTTTGCCCTATA
AA-TTAGTCATCTCATCAACCCTTCTCTTTCCAAAGAGATAATTGGCAGCAATTTGAACT
-TAAAGGTATTTGGTTAGCCTA-AAGGAAAGTCAGTTATTGGATGTGGTAACCTTAAAAT
ATTT---ATG--GAAATATAG-CATAG---------------------------------

------------------------TTAAACTGATAG-AGTTCACAACTTGGAGTCCAAAA
GTCCTGGGTTTGAATCCCTACTCTTGACACTTTGGA-GT---------------CTCTCC
AC--

---TAGG-AGTT----AC
CTGTTCAGTTATAAAGAGATTACATTTGCAGTG-----------------------TAAA
GGGAGG---TCCCACACAAAGGAA------------------------------------

-------------------------------------ATTGCATTTTCTTGATTTTGTTA

TAACTCTTTATT---GTATCCTAACATCTCCACTTTCTAATTACT---TAACCATTTTTT
TCCTAC-----TAGTTGG-TGTTGTC-----TTTGTTCTCAAGGAGGATCAAACTGACAT
CACTATATT-GGGTC-AGGTGTGTCGA---------TTGATCAGACCAATGCAA-GCTCA
GAGG--TCTCTACTACAGATCGGACACAAATAGTCCAAATCTATCTATAGA--TGGATAT
GTCTCTAGATTTGCACTTCTCATTTTTTTTA----AGCT---ACTGCATTTTTCCTTAC-
--TACTTTTCA-TGGAAAGAGCTCTAGGTTAGAAGTCAAGAGATCCGACTTGA-------
-------------------------------TTTCTGTCTGATCAGATCATTTTGTTCTT
CAGTTTTCTTCACTGTAAAA-------TGATGGGTTTG----------------------

---------------GACCAAAA---CATATTTGAGATCCC---TTAAAACTCCAAAAT-
----------TGTCTTGGTATGGGTCTTTGTATGCGATATTTGCTCAGGTCTACATGTTT
GCCCTTAG--GGTTACGAGATACGCATGTATGACAGC
>Pgu
AGCCGCCGTGTAAGCAGCTGTCTTCAAACAGCTGTGTCAAAGGAATCATTTTGCCCTATA
AA-TTAGTCATCTCACCAATCCTTATCTTTCC--------------AGATAATTTGAACT
-TAAAGGTATTTGATAGGCCT--AAGGAAAGTCAGTTATTGGATGTGGTAATCTTAAAAT
ATTT---GTG--GAAATATAA-TACAG---------------------------------

------------------------TTAAAC------------------TGGAGTCTAAAA
GACTTGGGTTTGAATCCCCATCCTT-ACATTTTGGAGGT----------------TCTCT
AC--

---TAGG-ACTT----AC
CTATTCAGTTATAAAGAGATTACATTTGCAGTGGATACTGGTTA----ATATCTAGTAAA
GGAAGG---TCCCACACAAGTGA-------------------------------------

-------------------------------------ATTGCATTTTCTTGATATTATTA
TAACTCTATATT-------------GTATCCACTTTCCAATTACT---TAACCA--TTTT
TCCTAC-----TAGTTGG-TGTTGTCTTTGTTTTGTTTTCAAAAAGGACCAAACTGACAG
CACCATATTGGGGTC-AGGTGTATCGG---------TTGATCAGCCCAATACAA-GCTCA
GAGGTCTCTCTACCACAC-GTGGACACAAATTATCCGAACCTACCT-TAGA--TGGAGAT
GTCTCTAGATTTGCACATCTCATTTTTTTAAAC--TACT------GC-TTTTTCCTTAC-
--TACTTTTCA-TGGTAAGAGCTCTAGGTTAGAAGTCAATCAGACTCAA-----------
-------------------------------TCTCTGTCTGATCAGATCATTTTGTTCCC
CAGTTTTCTTCACTGTAAAA-------TGATGGGTTTG----------------------

---------------GACAAGGA---CGTATTTGAGATCCC---TTAAAATGCCAACA--
-----------TTCTTGGTATGGTTCTTTGTATGTGATATTTGCTC--ATCTACATGTTT
GCTCTTAG--GGTTACGAGATACGCATGTATGACAGT
>Iso
AGCCGCCGTGTAAGCAGCTGTCCTCAAGCAGCTGTGTCAAAGGAATCATTTTGCCCTATA
AA-TTAGTCATCTCACCAATCCTTATCTTTCC--------------AGGTAATTTGAACT
-TAAAGGTATTTGTTTGGCCT--AAGGAAAGTCAGTTATTGGATGTGGTAATCTTAAAAT

```
ATTT---GTG--GAAATATAA-TACAG---------------------------------
------------------------------------------------------------
------------------------------------------------------------
------------------------TTAAACTGATAG---TCACAACTTGGGAGTCTAAAA
GACTTGGGTTTGAATCCCCATCCTT-ACATTTTGGAGGT----------------TCTCT
AC----------------------------------------------------------
------------------------------------------------------------
------------------------------------------------------------
------------------------------------------------------------
------------------------------------------------------------
---------------------------------------------TAGG-ACTT----AC
CTATTCAGTTATAAAGAGATTATATTTGCAGTGGATACTGGTTATTATATATCTAGCAAA
GGGAGG---TCCCACACAAGTAAA------------------------------------
------------------------------------------------------------
------------------------------------------------------------
------------------------------------------------------------
-------------------------------------ATTGCATTTTCTTGATATTATTA
TAACTTTATATT-------------GTATCTACTTTCCAATTACT---TAACCA--TTTT
CCCTAC-----TAGTTGG-TGTTGTCTTTGTTTTGTTTTCAAAAAGGACCAAACTGACAG
CACCATATTGGGGTC-AGGTGTATCGG---------TTGATCAGCCCAATACAA-GCACA
GAGGTCTCTCTACCACACGTTGGACACAAATTATCCAAACCTACCT-TAGA--TGGAGAT
GTCTCTAGATTTGCACATCTCATTTTTTTTTTTTAAGCT---ACTGC-TTTTTCCTTAC-
--TACTTTTCA-TGGTAAGAGCTTTAGGTTAGAAGTCAATCAGACTCAA-----------
-----------------------------------TCTCTGATCAGATCATTTTGTTCCC
CAGTTTTCTTCACTGTAAAA-------TGATGGGTTTG----------------------
------------------------------------------------------------
------------------------------------------------------------
------------------------------------------------------------
---------------GACAAGGA---AATATTTGAGATCCC---TTAAAATACCAACA--
-----------TTCTTGATATGGTTCTTTTTATGTGATATTTGCGC--GTCTACATGTTT
GCTCTTAG--GGTTACGAGATACGCATGTATGACAGT
>Tro
AGCCATG---TAAGCAACTGTCCTCAAGCAGCAGCATCGACGGGATCAGCTTGCCCTATA
AA------CCTCCCAT--CCACCCCTCTTTCC--AGAGAGAAGTGGCAGCGGGTTGAACT
CTAAAGGCATTTGGCCAGCAT--ACGGAAAGTCAGTTATCTGATGTGGTAACCTCGAAAC
GCTT---GAG--GCAGTATTG-CAGAG---------------------------------
------------------------------------------------------------
------------------------------------------------------------
-----------------------------TTGATGGACTTCAGGACTTGGT-GTCCACAT
GACCTGGGTTTAAACTCCT-GCTCTGACCCTTAGGGGCTGTGTGACTCCCCGCCTTGT--
GC----------------------------------------------------------
------------------------------------------------------------
------------------------------------------------------------
------------------------------------------------------------
------------------------------------------------------------
---------------------------------------------CGAG-GCTT----AT
CTGCTCCATT----ACAGATTACAT----------------------CTGCATCAGTAAA
GGGAAC---TCCCACACCTGTGAAG-----------------------------------
------------------------------------------------------------
------------------------------------------------------------
------------------------------------------------------------
-------------------------------------ATACCCTATTTTTCATGTGCTAC
TGTTACTTG--TGCTGAAGCCTAATGTCTTTAGTTTCCATTTACA---AGACCA--TTTT
```

CCCTG------CTGATCAGGGGCGTC----CTTTGTCTTG-AAGAGGACCAAAATGACAG
CACTATGTTGGGGTC-AAGTACAGTGTGCCCAAAGGTGGCTGGTCAGAGCAGTATGAGCT
-GGGAGGGCCTATCACAGATTGAGCACAAACAGTCTGTGTGAACATTTGGGCTTCTAGAG
GTCTCTAAATTTGCACATCTTATTTTTCTTTTG--AGCT-----------CCCCCC-AC-
------TTTAA-TCAAAAGACCACTAGGGGAGAAGTCAAGGGACTTGAATTTTAATCCTA
GCTTAGCTGGCCCTGGACTTCAT-------CTCTATGTCTGATCAGATCACTTTGCTGCC
CAGTGTCCTTTACTACGAAATACTATATGATCAGTTTA---GACCACGA---CAGGGGTA
AGGAACCTGCAGCCTCAAGGCCACACGTGGCCCTCTAGGCGGCCCTTTTTTAAGGGTCAA
------------------------ATCTCCTTAATAAAAGGATTTGTTCTGTAAAACTTG
G-------ACTCAGTCCACACCTGATTACCTACAAGGCCACATGGGGCCTCAAGACCCTG
---------------AACCAGGA---CATGTTTGAGGGCCC---CCAACACTCCAGCA--
-----------TTCTTGGTATGGT-CTGCGTATCTGATATTTGCTCAAGTCTACGTGTTT
GCTCCTAG--GGTTATGAGATACGTATGTATGACAGC
>Ppe
AGCCACA---GAAGCAGCTGTCCTCAAGCAGCAATATCAGCGGAATCAGCTTGCCCTATA
A-GCCAGTCATCTCATCCACCCTTCTCTTTCC--AGAGATAAGTGGCAGCAAACTGAGCT
TTAAAGGCATTTGGTTGGCCT--AAGGAAAGTCAGTTATCTGATGTGGTTACCTTGAAGT
ATTT---GAG--GCAGTGTGG-CATAG---------------------------------

-----------------------------TTGATAGAGTCCAAGACCTGGC-GTCCAAAT
GACCTGGGCTTAAACCCCCTACTCTGACCATTAGGGGCTGTGTGAATCCCCACCTTCT--
GC--

---CAAG-GCTT----AC
CTGCTCAGTT----ACAGATTATGT----------------------TTGCATCGGTAAA
GGGAAT---TCTCACACCAGTGAA------------------------------------

-------------------------------------ACTGC------------CATGAC
TATAACTCTTATGTTTTATCCTCATGTCTCTGCTTTCCAGTTATG---AGGACA--TTTT
CCCTAC-----TCATCCGTTGTTGTC----CTTTCTCTT-AAAGAGGACCAAACTGACAT
CGTTATGTTGGGGTCCAGGTACAGCGTGCCCTACGGTGGCTGGTCAGATCAGCATGAGCT
TAGCAGTCCCTGTCACAGACCGAGCACAGATCATCCGGGTGAACATTTGGAG-TGCAGAT
GTCTCTGAATTTGCACATCTCATATTTCTTTTG--AGTT---ACTGC-ATTTCCCT-AC-
------TTTA--TGGAAAGAACACTAGGGTAGAAGTCAAGAGATCTGAATTCTAATCCCA
GCTTAGCTGGCCCTGGACTTCAT-------CCCTGTGTTTGACTAGATCACTTTGCTCCT
CAGTTTACTTCACTATAAAG-------TGATCGGTTTG---GACCAGGC---CAGGGGTG
AGGAACTGGTGGCCTCAAGGCGGCACATGGCCCTCTAGTTCTTCAAGTGCAGTCCTTTGA
CTGAATCAAGATTTCACAGATCAGATCTCCTTAATAAAAGGATCTGTTCTGTAATGCTTG
GACTCAGTCAGAAGGCCACACCTGAGGACCTAAGAGGCCCCATGTGGCCTCGAGGTCCTG
---------------AACCAGGA---CATCTTTGAGGCCCC---CTAACACTCCAGCA--
-----------TTCTTGGCATGGCACTTTG----TGATATTTGCTCAAGTCTACATGTTT
GCTCTTAG--GGTTACGAGATACGCATGTATGACAGC
>Mro
AGCTACG---TAAA--TAGCTCCTCAAGAAGCACTATCAACGGAATCAACTTGCCCTATA
AAC-CAGTCATCTCATCAGCTCTTCTCTTTCC--AGAGATAAGTGGCAGCAAATTGAACT
TTGAAGGCATTTTTTTT---------GGAAGTCAGTTATTTGATGTAGTAACCTTAAAAT
GTTT---GAG--GA-ACATGG-CACAG---------------------------------

-----------------------------TTGATAGAAGTCAAGACTTGGG-GTCCAAAA
GATCTGAGTTTAAATCCCT-GCTCTGACCCTTAGGGGCTGTGTGACTACTCAACTTCT--
GC--

---TAAG-GTTT----AC
CTGCCCAGTT----ACATATTACAT----------------------TTGCATGGGTAAA
GGGAAT---TCCCCTGCCAGTGAT------------------------------------

-------------------------------------ACTGCATATTCTTGATGTATTAC
TGTAACTCTATATT-GTATCCTAATGTCTCCACTCTCCAATTATG---AGGCTA------
--TTAC------AATCAGTTGTTGTC----CTTTGTTTTGGAAGAGGACCAAAATGGCAT
CACTATGTTGGGGTC--------AACTGTGTCTGACTGGCTGATCAGACCAATATGAGCT
TGGAACATTCTACCCCAGAACGGGAGCAAATAATCCATGTGAACATCTAGGG-TAGAGAT
GTCTCTCAATGTGCCCATCTCATATT------------------------TCCCCT-AC-
------TTTCA-TGGAAAGAGCACTAGG---------------CTAGAATTCTAATCCCA
GCTTAGCTGGCCACGGACTTAAT-------CTCTGTCTTTGACCGGATCACTTTGCTCCT
CAGTTTCCTTCACTATGGAA-------TGATCAGTTGG---GATCAGGA---CAGGGGTA
GGGAACCTGTAGCCTTGAGACCAC---GTGGCCTCTAGGTCTTCAAGTGCGACCCTTTGA
CTGAATCCAAATTTCACAGTCCAAACCCCCTTCATAAAAGGATTTGTTCTGTATAACTTG
GACTCAGTCAAAAAGCCGCACCCAAGGACCCAGAAGGCCACATGTGACCTCAGGATCACA
GGTTCCCCACCCCTGAACTAAGA---CATCTTTGAGGTCCC---TTAACACTCCAGTA--
-----------TTCTTGGTAGGGTTCTTTGTATGTGATATTTGCTCAAGAGTACACGTTT
GCTCTTAG--GGTTACGAGATACGCATGTATGACAGT
>human
TCGCTG----AGGGTGGGACTGTGGCTTCAGTACC----------AGGAGTTGAGCTGCA
CTCTCAGCCTGGCATCTCAGCACTCTCTTCCCAGATAAATTACAGGGAGCAGGAGGCACT
TGAAGATACTGTGCTAACTTATGAAGAACACCTTGATTTTGTTTGGTTTTGCTATTGGTA
GGC---

---C
AATATGGTATTCACAGCAAAAGACAGGGGAAGAGGAGTGAGACCTGGGAGTAGATTGTTT
GTTTTCAG--GGCTATGAGATACACATGTTTGATAGT

MARKER 182

```
>WALLSI1A
------------------------------------------------------------
---------------------TGCATCCTGGGCCATCTCCAGTCATCCTGATCTATATCT
TGCCACTGGACCCAGATGGCTCTGGAGGAGAAAGTGAGGCTGGTGACTTTGCACAGCCCT
CCCTCACTTAAATCCAATTCACTTGCAAGTCATGKCATCACCTCCCTGATGTCATGGTCC
TCTTTGAGAACAAAGGACAAACAA---CAA------------------------------
------------------------------------------------------------
------------------------------------------------------------
------------------------------------------------------------
------------------------------------------------------------
------------------------------------------------------------
------------------------------------------------------------
------------------------------------------------------------
------------------------------------------------------------
------------------------------------------------------------
------------------------------------------------------------
------------------------------------------------------------
------------------------------------------------------------
------------------------------------------------------------
----------------------------
>SPIN_NA_6_Et
------------------------------------------------------------
------------------------------------------------------------
------------------------------------------------------------
------------------------------------------------------------
------------------------------------------------------------
------------------------------------------------------------
------------------------------------------------------------
------------------------------------------------------------
-----------------------------------------CAGTGGTTCTCAACCTTCC
TAATGCCGCGACCCTTTAATACAGTTCCTCATGTTGTGGTGACCCCCCAACCATAAAATT
ATTTTCGTTGCTACTTCATAACTGTAATTTTGCTACTGTTATGAATCGTAATGTAAATAT
CTGATATGCAG-ATGTATTTTCATTGTTACAAA----------TTGAACATAA-------
------------------------------------------------------------
------------------------------------------------------------
------------------------------------------------------------
------------------------------------------------------------
------------------------------------------------------------
------------------------------------------------------------
----------------------------
>MdoRep1
------------------------------------------------------------
------------------------------------------------------------
------------------------------------------------------------
------------------------------------------------------------
------------------------------------------------------------
------------------------------------------------------------
------------------------------------------------------------
------------------------------------------------------------
------------------------------------------------------------
------------------------------------------------------------
```

------------------------------ACCCTAAGGACCATGGGACCTTTCCATCTG
ACTTGCAGCTGAAA--CCTTCCATATGTGTTGTCTCCCCCATTAGAATGTGAGCTCCTTG
AGAGCAGGGACTGTCTTACTTTTCTATTTGTA-TCCCCAGCGCTTAGCACAG-------T
GCTTTGCACATAGTAAGTGCTTAATAAATGCTTTATTCATTCATTCATT-----------

>Mdo
-TATTG-CTCGCCCAG--GTAAGTTGGCCATGGCTGAGGGCGGAATGAGGA-AGGACTTA
AGAGCCATAGTCAAAGC----ATGACTTCCGTGCCATTCCATTTCTCTGTACCAGCTGGC
ACT---

---------------------------CAT-CACCCC---CCAGGCAAATCCT--TTCTT
CTTCCTCTCTTTTCTTTCCCATCCCATCTCTTT---------------------------
--TCTTCCTTTCTCCTTCTCACT---TTCCTCAATCCTC---------------------
CCATTGCCCTTCCCTTCCTTC---------------------------------------

---------TTCCTC-----TCCTTGCATATTGTCC-TCCATTAGAATGTATTCTCCTTG
AGGTCAAAGACTGTTTTGTCTTTGTCTTAGCA-TCAACTGAT------GGAAAA-----T
GGCTCTGAATAA-TTGTTGATTAATTCCTCC--------------CTTTTCTCCTTTCTT
TTCTGCTGATCCTTAATAAGGAATTGGGCAGTGAGTGCCCTCTACAGCTCTCTCTTCCCT
GGGAGTCTGTAAGGTGT-TCTGGATGG-GGCTGGGGATCAGGTGTCTGCTTCTGGAATGG
CCTTTACCTCTTATGGGCAGAGGTGGGG-TCAATAACTCACAATTGGATCCCCTTCTAG-
-GATGAAGATGGGAAGCAGCTGTCAGAT
>Dvi
-TATTG-CTGGCCCAG--GTAAGCCGGCCATAGCTGAGGGCCAGATGAGGA-AGGGCTGA
AGAGTCATGGGCAAAGC----ATGACTTTGTTGTCATTCCCTTTCTCTGTACCAACTGGC
ACT---

---------------------------CAT-CA-CCT---CCAGGCAAATCCT--TTCTT
CTTCCACTCTTTCCTTTCCTAACCCATATCTTT-------------TCTCTCCTTCTCTC
TTCTCTCTCCCTTTCTCCTCACTCTCCTCCTCAATCCTCTTCCCTTCCTTTTTCAGTTTC
CTTCTGCCCATTGTCC------TTTCTCTCCTTT---CTCTCCTTGCATGT---------

-------------------------------TGTCC-TCCATTAGAATGTATGCTCCTTG
AGGTCAAAGATTGTTTTGTCTTTGCCTTAACA-TCAGTTCATTGCCTAG----------T
GG-ATGGCATAA-TTATTGATTGATTTCTCC--------------CTTTCCTCCTTTCTT
TNCTGCTGATCCTCAACTGGAAATTGGGTAGTGAGTGACCTCTACAGCTCTTCCTTCCCT
GGGGGTCTGGAAAGTAT-TCTGGA-GGGGGCTGGGGATCAGGTGTCCGATTCTGGAATGG
CCTTTGCCTGTTATGGGCAGAGGTGGGAGTCAACAACTCACAACTAGATCCCCTTCTAG-
-GATGAAGATGGGAAGCAGCTGTCAGAT
>Mnu
GTATTGCGTGGCCCAG--GTAAGCTGGCCATAGCTGAGGGCCAGATGAGGA-AGGGCTGA
AGAGTCATGGGCAAAGC----ATGACTTTGTTGTCATTCCCTTTCTCTGTACCAACTGGC
ACT---

```
------------------------------------------------------------
---------------------------CAT-CG-CCT---CCAGGCAAATCCT--TTCTT
CTTCCACTCTTTCCTTTCCTAACCCATATCTTT-------------TCTTTCCTTCTCTC
TTCTCTCTCCCTTTCTCCTCACTCTCCTCCTCAATCCTCTTCCCTTCCTTTTTCAGTTTC
CTTCTGCCCATTGTCC------TTTCTCTCCTTTTTCCTCTCCTTGCATGT---------
------------------------------------------------------------
------------------------------------------------------------
------------------------------------------------------------
------------------------------------------------------------
-------------------------------TGTCC-TCCATTAGAATGTATGCTCCTTG
AGGTCAAAGACTGTTTTGTCTTTGCCTTAACA-TCAGTTCATTGCCTAG----------T
GG-ATGGCATAA-TTATTGATTGATTTCTCC--------------CTTTCCTCCTTTCTT
TTCTGCTGATCCTCAACTGGAAATTGGGTAGTGAGTGACCTCTACAGCTCTTCCTTCCCT
GGGGGTCTGGAAGGTGT-TCTGGA-GGGGGCTGGGGATCAGGTGTCCGATTCTGGAATGG
CCTTTGCCTGTTATGGGCAGAGGTGGGGGTCAACAACTCACAACTAGATCCCCTTCTAG-
-GATGAAGATGGGAAGCAGCTGTCAGAT
>Dgo
-TGNTG-CTGGCCAAG--GTGAGCAAAAGGAGGACGAGGGTGGGGTGAGGA-AGGGC-T-
GGAGTCATGGCCAAAGC----CTGACGTTGACTTTATGCCAGTTCTCTGAACCACCCCTC
ACT---------------------------------------------------------
------------------------------------------------------------
---------------------------CAT-CA-CCC---CCTAGCAAATCCT--TTCTC
TTTCTCCT--CATCTCCCTCCGTGCCTCTCTT---------CCCTTTCCCTTCCTCTCGG
TTTTCTTTCCCTTCCCTTTTTCTTTTCTTCCAAATTCCTCTTCTCTTCTCTT-CCCCC--
----TTTTTCCTCTTC------TTCTCCCTCTTCTTGCTGTCCTTCCATGC---------
------------------------------------------------------------
------------------------------------------------------------
------------------------------------------------------------
------------------------------------------------------------
TACTTCTC-------CCTTCCTTGTGTACTATGTCC-TCCATTAAACTCTAATCTGCTTG
AAGTCAGGGGCTGCTTTGCCTTTGTTTTCACA-TCAGCTCACAGCCTGAAACAC-----A
GTGACTGAACAA-TTGCTTGGTGATCTATTC----------CTACTTCTCTTCCTTCC-T
CTCTGCTGTTCCTCAACGTGGCACGGGGCAGCATGTGCCATCTACAACCTTCCCTTCCCT
GGGAGTCTGGAGGGTGA-------------------GCCAGGTGTCTTCTCCTG-----G
CCTTTACCTCTTATAGGTAGAGGTAAGG-TCCATCATTCCCAACTGCAATCCCTTTCAG-
-GATGAAGACGGGAAGCAGCTATCAGAT
>Scr
-TGTTG-CTGGCCAAG--GTGAGCAAGGGA-GGCTGAGGGTGGAGTGAGGA-AGGGC-T-
GGAGTCATGGTCAAAGC----CTGACTTGGACTTTATGCCAGTTCTCTGAACTATCCCTC
TCT---------------------------------------------------------
------------------------------------------------------------
---------------------------CAG-GA-CCC---CCTGGCAAATCCT--TTCTT
TCTCCTCA----TCTCCCTTCTTGCCTCTCTT---------CCCTTTTCCTTTCTTTCTG
TTTTCTTTCCCTTCCCTTTTTCTTTTCTTCCAAATTCCTCTTCTCTTCTCCTTCCCCC--
-TTCTCTTTCCTCTTC------TTCTCCCTTTTCTTGCTCTCCTTCCATGCTACTTCTCC
TTTGTGCCCTT-------------------------------------------------
------------------------------------------------------------
------------------------------------------------------------
------------------------------------------------------------
-------------------------------TGTGC-TCCATTAGAATCTAATCTGCTT-
-AGTCAGAGACTGTTTTGCCTTTGTTTTTGCA-TGAGCTCACAGCCTGAAACAG----CA
GTGACTGAACAA-TTGCTTGGTGATTGATTC----------CTACTTCTCTTCCTTCC-T
CTCTGCTGATCCTCCACATGGCACTGGGTAGCGAGTGCCATCTACCACCTTCCCTTCCCT
```

GGGAGTCTGGAGGGTGA-------------------GCCAGATGTCTTGTCCTG-----G
CCTTTACCTCTTATGGGTAGAGGTAAGC-TCCATCATTCCCAACTGTAATCCCTTCTAG-
-GATGAAGATGGGAGGCAGCTGTCAGAT
>Pgu
-TGTTG-CTGGCCAAG--GTGAGCAAGGGA-GAATGAGGGCAGGGTGAAGG-AGGGCCT-
AGAGTCATGGTCGAAGG----CTGACTAGGACTTAATGCCATTTCTCTGAAGCATTCCTT
GCA---

---------------------------CAT-CA-CTC---CCTGGCAAGTCTTCTTTCTT
CTTCCTC-------CCCTCCAT----------------------TTCCCTTCCTTTCTCT
TTTCTTTTTCTTTCCTTCT-CCCTTTCTTCTTTATTCCTCTTCTCTTCT----CCCCC--
-TTCTTCCTCTTCTTC----TCATTCTTCTTTCCCTGCT-GCCTCTCATC----------

---TTT
TATTTCTGTC-----CTATATATGTGCACATTGCCCCCTCATTAGAATCTAATCTGCTTG
AAGTCAGGGGCTGTTTTGCCTTTGCCTTTGC--TCAGCCCATGGATTGGCACAG-----T
GGCTCTGAACAAATTGCTTGTTGTTTGATTC----------CTACTCCCCTTT----GTT
CTCTGCTCTTCTTCAACTAGACTCTGGGTAGAGGGTTCCCTCTATAGTCTTTCCTTCCCT
GGGAATCTGGAAGGTAATTCTGGA------GAGTGGGACAGGTGTCTCCTCCTGGAATGA
TCTTTGTTTATTATGGGCAGAGGTGGGGGTCAATCACTCACAACTGGAACCCCTTCTAG-
-GATGAAGATGGGAAGCAGCTTTCAGAT
>Iso
-TGTTG-CTGGCCAAG--GTGAGCAAGGGA-GAATGAGGGCAGGGTGAAGG-AGGGCCT-
AGAGTCATGGTCAAAGG----CTGACTTGGACTTAATGCCATTTCTCTGAAGCATTCCTT
GCA---

---------------------------CAT-CA-CTC---CCAGGCAGGTCTTCTTTCTT
CTTCCTC-------CCCTCCAT----------------------TTCCCTTCCTTTCTCT
TTTCTTTCTCTTTCCTTCT-CCCTTTCTTCTTTATTCGTCTTCTCTTCT----CCCCC--
-TTCTTCCTCTTCTTC----TCATTCTTCTTTCCCTGCT-GCCTCTCATC----------

---TTT
TATTTCTGTC-----CTATATATGTGCACATTGCCCCCTCATTAGAATCTAATCTGTTTG
AAGTCAGGGGCTGTTTTGCCTTTGCCTTTGC--TCAGCTCATGGATTGGCACAG-----T
GGCTCTGAACAAATTGCTTGTTGTTTGATTC----------CTACTCCCCTTT----GTT
CTCTGCTCTTCTTCAACTAGACTCTGGGTAGAGGGTTCCCTCTACAGTCTTTCCTTCCCT
GGGAATCTGGAAGGTAATTCTGGA------GAGTGGGTCAGGTGTCTCCTCCTGGAATGA
TCTTTGTTTATTATGGGCAGAGGTGGGGGTCAATCACTCATAACTGGAACCCCTTCTAG-
-GATGAAGATGGGAAGCAGCTTTCAGAT
>Tro
-TGCTG-CTGGCCAAG--GTTGGTGGCCAA-GGGC------AGGGTGAGGA-AGGGCCT-
AGAGCCATGGGCAAAGCCTGCCTGGC-TTTACTTGAAGTCATTTCTCTGAACCACCTTTC
CCT---

---------------------------CAT-CA-TCC---CCTCGCAAGTCCTCTTTCTT
CTTCCTC---TCCTCTCTGCACGCCTCTTCCCATGT---------TCCCTTCCTGTCTCT
CTTCTTTCCTTTCTTCCCCATTCCTCTTTCTCTTCCCTT--------------CCCCC--
-TTCCTCTGCTCTCTCTTC---TTGCTCTCCTTCCAGTTTACTTCTTCCCTTTT------

-ATTTTTGTT-----TGCTGTATGTGGACTTTGTTTCC-CATTAGAATCTAATCTGCTTG
AAGTCAAGGGCTGCTTGGACTTTGTCTTTGCCCGTAGCC--TGG---CACACAG-----A
GGCTCTGAACAA-TTGCTTGTGGAGTGATGC----------CTACCTTTCTTTCCTTCTT
CTCTGCTGTTACTTAACTACACTTTGGGTAGCGAGTGTCCTCTAGAGTCCTCCCTTCCCA
GGGAGTCTGGAGGGTGGTTCTGGA------GGGCAGATCAGGTGTCTCTTCCTCCCGTGG
CCTTTGCCTCTACTGGGCAGAGGTGGGG-TCAATCACTCACACCTGGTACCTCTTCTAG-
-GATGAAGAGGGGAAGCAGCTGTCAGAT
>Ppe
-TGTTG-CTGGCCAAG--GTGAGCAAAGGA-GGCC-----AATGGTGAGAA-AGGGCCT-
AGAGCTGTGGTCAAAGC----CTGGCTTTGACTTGATGCCATTTCTCTGAACCATCCCTC
ACT---

---------------------------CAT-CA-TGC---CCT-----GTTCTCTTTCTT
CCTCTTC---TCCTCCCTGCATGCCTCTTCCCCCTT---------TCCCTTCCTTTCTCT
T---CTTCCTTTCCTCCTCATTTCTCTT-CTCTTCTCTT--------------CCCCG--
-TTCCTCTTCTCCTCCCTCTCCTTGCTCTTCTTCCATGCTACTTCTCCTTCCCTTTTTTT
TTGGTCCTATGTATGTGTACTTTATTTTCCCATTAGAA---CAGTGGTTCTCAATCTTCC
TAATGCCGTGACCCTTTAATACAGTTCCTCATGTTGTGGTAACCCCCCAGCCATAAAATT
ATTTTTGTTGCTACTTCATAACTGTAATTTTGTTACTGTTATGAATCATAATGTAAATAT
CTGATACGCAGGATGTATTTAAATTGAAAATAACCTACAGGTTGAGAACTGCTG------
---------------------------------------CATTAGAATCTAATCTGCTTT
AAATCAAGGGCTATTT-GCCTTTGTCTTTGCTCA-------------CACAGAG------
-GTTCTGAACCA-TTGCTTGTGGACTGATTC----------CTACCTTTCCTTCCTTCTT
CTCTGC----------------ATTGGGTAGCAAGTGCCCTCCAGAGTCCTCC-TTCCCT
GGGAGTCTGGAGGGTGGTTCTGGA------AGGCAGACGAGGTGTCTGCTCCTGGAATGG
CCTTTGCCTCTACTGGGC--AGGTGGGGGGCAATCACTCACAACTGGTACCCCTTCTAG-
-GATGAAGATGGGAAGCAGCTGTCAGAT
>Tvu
-TGTTG-CTGGCC----------------------------AGGGTGAGCCGAGGGCCT-
AAAGTCATGGTCAAAGC----CTGACTTTGACTTTATGCCATTTCTCTGAACCATTCCTC
ACT---

---------------------------CAT-CA-TCC---CCAAGCAAATCCTCTTTCTT
CTTCCTC---TCCTCCCTCCATGCCTCTTCC-CTTT---------TCCCTTCCTTTATCC
TTTCTTTCCCTTCTCTTCTTCCTTTCCTCCTCATTCCTCTTCTCTTCCT----CCCTTC-
-------------TTCTTCTTCTTGCTCTCCTTCCATGCTACTTCTT--CCCCTTTATTT
TTG---

---------T-----CCTATATATGTGCACATTGTCCCCTATTAGAATCTAATCTGCTTG
AAGTCAAGGGCTATTTTGCCCTTATCTTTGCTCATGGCC-----TGACACACAG-----T
GGCTCTGAACAA-TTACTTGTGGATTGATTC----------CTACCTTCCCTTCCTTCTT
CTCTGCTGTTCCTTAACTAGGCATTGGGTAGCAAGTGCCTTCTATAG-------------
-------------CTGGTTCTAGA------GGGCGGGCCAGGCATCTGCTCCTGGGATTG
TCTTTGCTTCTACTAGGCAGAGGTGGGG-CCAATTGCTCACAACTGGTACTCCTTCTAG-
-GATGAAGATGGGAAGCAGCTGTCAGAT
>Mro
-TGTTG-CTGGCCAAG--GTGAGCAAGAAA-GGCCCAGGGTAGGGTGAAGA-AGGGCCT-
AGAGTCATGGTCAAAGC----CTGACTTTGACTTTATGGCATTTCTCTGAACCATCTCTC

ACT---CAATATCT---GGCTCAGGAGAAGAAGGTGAGATTGGTGACCTTGCACAGCCCT
CTCTCACTCAAATCAAAGTCAGCTGCAAGTCACGTCATCATCT---TGATGTCATGGTCC
TCTTCGAGAACAAAGGACAGACA----CAAACAATCC---CCTAGCAAGTCCTCTTCCTT
CTTCCTC---TCCTCCCTTCATGCCTCTTCTCCCCTT-------TTCTTCCTCTCCTCCT
CTGTCTTCCCCTCCCCTCCCCTCCTCTCTCTCTTCTGTT--------------CCCCTTT
CTTC---CTTTTCTCCCACTTCTTGCTCTCCTTTCACACTACTTCCCTTTTATTTTTGT-

---------------CCTATGTATGTACATGTTGTCCCCCATTAGAATCTGATCTGCTTG
AAGTCAAGGGCTATTTTGCCTTTGTTTTTGCTCATGGCC-----TGATACATGG-----T
GGCTCTGAACAA-TTGCTTGTGGGTTGATTC----------CTACCTTCCCTTCCTTCTT
CTCTGCTGTTCCTTGACTAGC-ATTGGGTAGTGAGTGCTCCCTACAGTTCTCCCTTCCC-
TGGGAGTTGGAGGGTGGTTCTGGA-------GGGTGGCCAGGTGTCTGCTCCTGGAATGG
CCTTTGCCTCTCTTGGGTAGAGGTGGGG-CCAATCAGTCACAACTGGTACCCCTTCTAG-
-GATGAAGATGGGAAGCAGCTGTCAGAT
>Ptr
-TGTTG-CTGGCCAAG--GTGAGCAAGAGA-GGCCCAGGGAAGGGTGGAGA-AGA-CCT-
AGAGTGATGGTCAAAGC----CTGATTTTGACTT-ATGCCATTTCTCTGAACCATCTCTC
ACT---CAATATCC---AGCTCAGGAGAAGAAGGTGAGGTTGGTGACCTTGCACAGCCCT
CTCTCACTCAAATCAAAGTCAGCAGCAAGTCATGTCATCATCT---TGATGTCACGGTCT
TCTTTGAGAATGAAGGACAAACA----CAAATAATCC---CCTAGCAAGTCCTCTTTCTT
CTTCCTT---TTCTCCCTTCATGCCTCTTCTCCCCCT-------TTCTTTCTCTACTCCT
TTGTCCTCTCCTTCCTTTCCCTCTTCTCTCTCTTTTCTCTTCCTCTTCCTT---CCTTTT
CTTC------------CACTTCTTGCTCTCCTTTCATGATACTTCTCCTTCCCTTTT---

--ATTTTTAT-----CCTATGTATGTGCATATTGTCCCCCATTAGAATCTGATCTGCTTG
AAGTCAAGGGCTATTCTGCCTTTGTTTTTGCTCATGGCC-----TGACACGCAG-----T
GGCTTTGAACAA-TTGCTTGTGGATTAATTC----------CTACCTTCCCTTCCTTC-T
CTCTGCTGTTCCTTAACTAGC-ATTGGGTAGCGAGTACCTTCTACGGTCCTCCCTTCCC-
TGGGAGTTGGAGGGTGGTTCTGGA-------GGGTGGCCAAGTGTCTGCTCCTGGAATGG
CCTTTGCCTCTC------AGAAGTGGG--CCAATCAGTCACAACTGGTACTCCTTCTAG-
-GATGAAGATGGGAAGCAGCTGTCAGAT
>Vur
-TGTTG-CTGGCCAAG--GCGAGCAAGGGA-GGCC-AAGGAGGGGTGAGGA-AGGGCCT-
AGAGTCATGGTCGAAGT----CGCT------------------TCTCTGAACCATCTCTC
ACT---

---------------------------CAT-CA-TTG---GCTAGCAAATCCTCTTTCTT
CCTCCCC---CTCTCCATNCCTCTCCCCCCTTTCCCTTTGTCTTTTCTTTCCCTTTGTCT
TTTCTTTCCCTTCTCTTCTTCCTTTCTTCCTCATTCCTCTTCTCTTCCCTT--CCCCCCT
CTTCTTCCCCTTCNTCCTTTTCTAGCTATCCNTCGATGCCACTTCTCTTTCCTTTTT---

--ATTTTTGT-----CCTATGTATATGTATGTTGTCCCCCATTAAAATCTAATCTGCTTG
AAGTCAAGGGATGTTTTGCCTTTGTCTTTGCTCGCAGCC-----TGACACACACACTGAC
AGCTCTGAACAA-TTGCTTGTTGATAGATTC----------CTACCTCCCCTTCCTTCT-

--CTGCGGTTCCTCAACAAGGCACTGGGTAGGG--------------TCCTCCCTTCCC-
TGGGGTTTGGAGGGTGGTTCTAGA------GGGCAGGCCAGGTGTCTGCTCCTGGAATGG
CCTTTGCCTCTACTGGGCGGAGGTGGGG-CTAATCACTCACAACTGGAACCCTTTCTAG-
-GATGAAGATGGGAAGCAGCTGTCAGAT

MARKER 194

```
>WSINE1
------------------------------------------------------------
------------------------------------------------------------
------------------------------------------------------------
---------------------------GAGGGGCAGCTAGGTGGTGCAGTGGATAGAGCA
CCAGCCCTGGAGTCAGGAGGACCTGAGTTCAAATCCAGCCTCAGACACTTA-------CT
AGCTGTGTGACCCTGGGCAAGTCACTTAACCCCAATTGCCTC------------------
---AAAAAAAAAAAAAA-------------------------------------------
------------------------------------------------------------
------------------------------------------------------------
------------------------------------------------------------
------------------------------------------------------------
--------------------------
>Mdo
AGTCAGCATTTGAACCCCTCGTGGCAAATGGAGCCCCAGCATCTCTTGTTCCCAA--GTA
AGTG---TTTCCCT-AATAGGATGTTTAGCAAGGAGGCACTTGAGTCCAAGTTATTCTTC
CCAAA--CCTGTACTTATTAAGAACAGA--------------------------------
---CTTCATTAC------------------------------------------------
------------------------------------------------------------
------------------------------------------------------------
--------------------CCAGTAACTG---TTTCTCTTTGCC------TGTTATGTT
AGTTCAAGATAGTATCTGTCACCTTCAGAGACTTAGGCAGGATTAAAATTCAAA-TGTGC
T----------------------------------------TAGCTAAA-TTGTTACCAG
---TAAAATTAGCCTAATTCCTTC-CCCCTCAACCACATGAAGAAAGGAGTTATGGCCAA
GTTAGATGTAGCCTTAAAAAATGTCACTTTTCATGAGATCTCTGACTCCTTTTTTT----
-----CCTTCCATTAG--GCCTGGAT
>Dvi
AGTCAGCATTTGAACCCCTTGTGGCAAATGGAGCCCCAGCATCTCTTGTTCCCAA--GTA
AG-----TTTCCCT-AATAGGATGTTTAGCAAGGAAGCAAACAGA---------------
-------GC---------------CAGA--------------------------------
---CTTCATTAC------------------------------------------------
------------------------------------------------------------
------------------------------------------------------------
--------------------CCAATAACTG---TTTTTCTTTGCTTATT-ATATTACGTT
GGTTCAAAATAGTATCTGTCACCTTCAG-GATTTAGGCAGGATTAAAATTCAAA-TGTGT
AGATCTTTCTCATAGCCCTACTCTCTTTTCATAATCCTTCTTAGCTAAA-TTGTTAACAG
---TAAAATTAGCCTAATTCCTTT-CCCTTCAACCATATGAAGAAAGGAGCTACAACCAA
GTTAAGTGTAGTCTTAAAAAATGTCACTTTTCCTGAGATCTCTGACACCTTTTTTTTTTT
TTTCTCCTTCCATTAG--GCCTGGAT
>Dgo
ACTCGGCATTTGAACCCTTGGTGGCAAATGGGGCCCCAGCGTCTCTTGTTCCCAA--GTA
AGTAATGGCTCCCTAAAT---AAGTTT-ACAAGGAGGTAAACA-----------------
-------GCCTGAATTTAAGTGATTCTGCCCAA-CCTGTACT--GAACATCAGAATCATA
AAAATTAATGAT------------------------------------------------
------------------------------------------------------------
------------------------------------------------------------
--------------------CCGATAATTG--------TTTCTCTATAC-CTGTCATGCT
GGTTCCAGATACAATCTGTTGGCTTCAGAGCTTTTAACAGGATTAAATTTCAAA-TACCT
TGATCTTCATATT---CCCAGCATGTTA--------------------------------
---CTACATTAATATAATT-CTTCTCCTGGGTT--ACATAAAGAAAGAGGCCATGGCTAA
GT--------CGCA-----CACGTCACATCCCAAGAGGTTTCTGACTC---------CTT
```

```
TTGTTCCTTCCATTAG--GCCTGGGT
>Scr
ACTCGGCATTTGAACCCTTGGTGGCAAATGGGGCCCCAGCGTCTCTTGTTCCCAA--GTA
AGTAATGG------------AAAGTTT-AATAGAAACTTAATAGAAAGTTTAACAAGGAG
GTAAACAGCCTGAGTCTAAGTGACTCTGCCCAA-CCTGTACTTGGAACATCAGAACCATA
AAAATTAATGAT------------------------------------------------
------------------------------------------------------------
------------------------------------------------------------
--------------------CCGATAATGG--------TTTCTCTCTGC-CTATCATGCT
-GTTCCAGATACAATCTGTTAGCTGCAGAGCCTTGAGCAGGATTAAATTTCAAA-TACCT
TGATCTTTATATT---CCCACTGTGTTG--------------------------------
---CTACATTAACATAATTCCTTCTCCTGGGTT--ACATAAAGA----GGCCATGGCCAA
GT----------------------CACATCCCAAGATG-------------------CTT
TTGTTCCTTCCATTAG--GCCTGGAT
>Bil
ACTCGGCATTTGAACCCCTGGTGGCAAATGGAGCCCCAGCATCACTTGTTCCCAA--GTA
AGTCAGAGTTCCCTTTATAGAATGTTTAGCAAGGATGGAA--GGA---------------
-------ACCTAAATCTAAGAGATTCTGCCTAGACCTGCTT---GTAAAGAATATCATAT
CAAATTATTTAC------------------------------------------------
------------------------------------------------------------
------------------------------------------------------------
--------------------CCAATAACTC-------TTTTCTCTTTGC-ATGTCATGTT
GGTTCAGGATAGATT----CACTGTCAGAGCCTTATGCAGGGTTAGTTTTTAAA-TACTT
AGATCTTCTTCCTATTCCCACTTGGTTTTCATAAC--TGTCTCACCAAA-TCGTTACCAA
---TAATATTAATATAATTCCTCT-CACGAATT--GGATCAAGAAAGAAGCCTTGTTCAA
GTTAGATGTAGCCTTGAGAAATGACACTTCTCAAGAGATCCCCAATTG---------CCT
TTATTTCTTCCATTAG--ACCTGGAT
>Ppe
ATTCAGCATTTGAACCTCTGGTCGCAAATGGAGCCCCAGCATCTCTTGTTCCGAA--GTA
AGTGATAGTTCCCTTAATAGAATGTTTAACGAGAAGGCAAACAGA---------------
-------GCCTGCGTCTAAGTGATTTTGCCCCAACCTGTACTC-GATAAGAACATCAGAC
CTAATTAATTAC---CCAGTAACT----AGAGGCAGCTAGCTTATGCAGTGAATAGAGCA
CTGGCCCTGGAGTGAGGAGGACCTGAGTTAAAATCCAAATGC---CACTTAA------CT
AGCTGTGTGACCTTGAGCAAGTCACTTGACCCCGAT--------------CCCCGCGAA-
-------------AAAA------AGTAACT-------CTTTCTCTTTGC-TTATCATGTT
GGTTCCAGATAGAATTTGTCACCTTCCAAGCCATAAGCAGTGTTAAATTTCAGA-TATTT
AATTCTTCGTCATGTTCCCAGTTTGTTTTCATAACTCCACCTCGCTAAA-TTGTTAGCAG
---TTACATTAACATAATTCCTTCTCCTGGGTT--GCATAAAGAAAGAAGCCATGGCTAG
GTTAGATGTAGCCTTGAGAAATATCACTTCCCAAGAGATACTTGACTA---------CCT
TTGTTCCTTCCATTAG--GCCTGGAT
>Tvu
ACTCAGCATTTGAACCTCTGGTCGCAAATGGAGCCCCAGCATCTCTTGTTCCCAA--GTA
AGTCATAGTTCCATTAATAGAATGTTTAGCAAGAAGGTAAACAGA---------------
-------------GTCTAAGTGATTCTGCCCAAACCTATACTC-ATCAAGAACATCAGGC
TTAATTAATTAC---CCAGTAACT----AGAGACAGCTAGGTGTAGCAGTGAATAGAGCA
TCTGCCTTGGAGTCAGGAGTT--------CAAAACCAGCCTCAGACACTTGACACTAGCT
A----TGTGACCTTCGGCAAGGCACTTAACCCTGATTGCCTCATCTCCCCCCCAAAGAAA
AAG----------ATTA---CCCAGTAACT-------GTTTCTCTTTGTTTTATCATGTT
GGTTCT-GTTGAATCTGTCACCCTT-AGAGCCTTAAGCAAGCTTAAGTTTCAGA-TACTT
AGATCTTCATCATATTCCCAATTTGTTTTCGTAACTCCACCTAGCTGAA-TTGTTAGCAA
TTATTACATTAACATAATTTCTTCTACTGGGTT--GCATAAAGAAAGAAGCCATGGCCAA
GTTAGATGTAGCCTTGAGAAATGTCACTTCCCAAGAGATACCTGACTA---------CCT
TTGTTCCGTCCATTAG--GCCTGGAT
```

```
>Mro
ACTCAGCTTTTGAACCTCTGGTTGCAAATGGAGCCCCAGCATCTCTTGTTCCCAA--GTA
AGTAAA---------AGTAGAATGTTTAGCAAGAAGGGAAACAGA---------------
-------GCCCGAGTCTAAACGATTCTCCCCAAACCTCTACTC-ACTAAGAACATCAGAC
CTAATTAATCAC--CCCAGTAAT----GAGAGGCAGCTAGGTAGAGCAGTGAGTAGGGTA
CCTGCCCTGGAGTCAGGAGAACCTGAGTTCAAACCCAGCCTCAGACACTTGACACTTACT
AGTTGTGTGACCTTGGACAAGTCACTCAACCCTGATTGCCTCGCCTACCCCTTCCAAAAA
GAGAAAAAAAAAAATTA---CCCAGTAACT-------GTTTCTCTTTGC-TTATCATGTT
GGTTCAGGGTCAAATCTGTCACCCTTGGAGCCTTAAACTGGGTTAAGTTCCAAA-TACTT
AGATCTTCCTCATATTTCCAGTTTATTTTCATAACTCTGCCTAGCTGAA-TTGTTAGCAA
---TCACATTAGCATACTTCCTTCTCCTGGGTT--ACATGAAGAGAGAAGCCATGGCCAT
GTTAGAGGTAGCCTTGAGAAACGTCACTTTCCAAGAGATGCCTGACTG---------CCT
TTG-TCCTCCCACTAG--GCCCGGAT
>Vur
ACTCAGCGTTTGAACCCCTGGTCGCAAATGGAGCCCCAGCATCTCTGGTTCCCAA--GTA
AGTAATAGTTCCCTAAATAGAACGTTTAGCAAAGAGGTAAACAGA---------------
-------GCCTGCGTCTGAGTGATTCTGCCCAGACCTACACTC-ATTAAGAACATCAGAC
CTAATTAGTTG-------------------------------------------------
------------------------------------------------------------
------------------------------------------------------------
--------------------TCCAGTAACT-------GCTACTCTTTGC-TTTTCTTGTT
GGTTCAGGATAGTATCTGTCACCCTCAGAGCCTTAAGCAGGGTTAAATTTCAAAATACTT
AGATCTTCCTCATATCCCCAGTTTGTTTTCATAACT--GCCTAGCTGAAATTGTTACCAA
---TTATGTTAACATAACTCCTTCTCCTGGGTT--ACTTAAAGAGAGAAACCATGGCCAA
GTTAGATGTAGCCCTGAGAAA------------------------------------TCT
TTGTTCCTTCCATTAG--GCCTGGAT
```

MARKER 205

```
>MIR3_MarsA
------------------------------------------------------------
------------------------------------------------------------
---------------GGGAAG-CAGCGTGGTACAGTG---GAAAGAGCG----C-----T
GGATTTGGAGTCAGAGGACCTGGGTTCAAATCCCGGCTCTGCCACTTACTACCTGTGTGA
CCTTGGGCAAGTCACTTAACCTCTCTG----GGCCTCAGTTTCCTCATCTGTAAAATGAG
GGGGTTGGACTAGATGACCTCTAAGGTCCCTTCCAGCTCTAAATCTATGATCCTATGATC
CTATGA------------------------------------------------------
------------------------------------------------------------
------------------------------------------------------------
------------------------------------------------------------
------------------------------------------------------------
---------
>Mdo
CTTTCCATGGGGATGATCTGCTGCATCCGGAAATATATCCCAGAGGG--GTGAGCAGGCC
AAGCCAGGGGCCCGGAGGCTGG-CCTGGGGGGGCAGGGGAGCCTGGG-GG----------
------GGCACTGGA---------------------------------------------
------------------------------------------------------------
------------------------------------------------------------
------------------------------------------------------------
------------GCC-GCTCTG-GCGTCCCC------TTCCTCCTCCTTGCAGAGGGCCC
TACATTTCCGTCCTGA-CACTGTGG--------------TCCAGAA---------GGCTG
GGCTCTGGGTCC----TGTGGT-CCCGCCAGCCCTTGCGGAT-TGGGGAG----CAGACA
GGGGC--CCCC--GGGAAGGCTCCGGAGTCAGCACTGTGTCCC-AGCTCCTCTTTCCGAT
CCCTGGACAG--ATGCTGCCGTTACCTGACCCTGCAGACCCGAACGCTCATCAGCGTGGG
CATCTTCTC
>Tel
CTTTCCATGGGGATGATCTGCTGCATCCGGAAATATATCCCAGAGGG--GTGAGGAGGCT
AAACCAGGGGCCTGGAGGCTGG-CTTGGGGGGACCGGGAGTGGGGTGGGGGGCCGTTCTG
CTGCCGGGCACTGGA---------------------------------------------
------------------------------------------------------------
------------------------------------------------------------
------------------------------------------------------------
------------GCC-GTTCTG-GCATCCCA------TTCCTCCCCTTGCTCCTTGCCGT
CCGG-----CCCTAGACTTCTGTGTCCGTGGCACTGCGGTCTAGAA---------GGCTG
GGCTGTCCGCCCT---GTGGGT-CCCACCAGCCCTTGGGGATTTGGGGAG----AAGACA
GGGGC--CCCGTGAGGAAGGCTCCAGAGTCAGCACTGTGTCCC-AGCTCCTTTTTCCAAT
CCCTGGACAG--ATGCTGCCGTTACCTGACCCTGCAGACCCGAACGCTCATCAGCGTGGG
CATCTTCTC
>Dvi
CTTTCCATGGGGATGATCTGCTGCATCCGGAAATATATCCCAGAGGG--GTGAGGAGACT
AAGGCAGTGGCCTAGAGGCTGG-CCTGGGGGGGTGAGGGTGGGGGGTGCTG---GTTCTG
CTGCCTGGCACCGGA---------------------------------------------
------------------------------------------------------------
------------------------------------------------------------
------------------------------------------------------------
------------GCT-TCCCTG-GCATCCCA------TTCCTCCTCCTGCTCCTTGCCAT
CCTG-----CCCTAGATTTCTGTGG--------------TCTAGAA---------GGCTG
GCTCTTGGTCCTTGGGTCCCACCAGCCCTTGGGATTTGGGGA-G----------AAGACA
GGGGC--CC-GTGGGGAAGGCTCCAGAGTCAGCACTGTGTCCC-AGCTCCTTTTTCCAAT
CCCTGGACAG--ATGCTGCCGTTACCTGACCCTGCAGACCCGAACGCTCATCAGCGTGGG
```

CATCTTCTC
>Rra
ATTTCCATGGAGATGATCTGCTGCATCCGGAAATATATCCCAGAGGG--GTGAGGAGGCC
AAGGCAAAGGCCTGGAGGCTGGACTTGGGG-----------TGGGGTGGG-GG-AGGACG
GGGCAGGGCA-GGGA---------------------------GATGGAGCAGCCAC---
--TGCCC-------------AACTTGAGCTCAGAGGCAGAT---CCCATGTCTGCTCCTC
TTTGTCTGG----GTCACTCCCACACCCTG----GAGGTCCAAGTAGATAACTT------
-----------------------------CCCCA---------AG--------------
------------CCC-TCTGTC-TGTACAT--------------GCCA--------CTAG
GACTGGAGCTCTCCAGG--------CCCTACGGGTCATGC-TA--------GCCCTTGAG
GGTTT-AAGAGGAAAGGGGGATGGGTGGCACCCCTTC---TT-GGCCTAGGCCACAGACA
GGGGG-TCCTGTGGGGAAGGCTGGTGAGTCAGCCCTGTGTCCCCAGCTCCTTTCTCCAAT
CCCCGGACAG--ATGCAGCCGTTATCTGACCCTGCAGACCCGAACGCTCATCANTGTGTG
CATCTTCTC
>Cfu
ATTTCCATGGGGATGATCTGCTGCATCCGGAAATATATCCCAGAGGG--GTGAGGAGGCC
AAGGCAAAGGCCTAGAGGCTGGACTTGGGG-----------TGGGGTGGG-GG-NGAACG
GGGTGGGGCG-GGGA---------------------------GATGGAGCAGCCAC---
--TGCCC-------------AGCTTGAGCTG-GTGGCAGAT---CCCATGTCTGCTCCTC
TTTGTCTGG----GTCACTCCCACACCCTG----GAGGTCCAACTAGATAACTT------
-----------------------------CCCCA---------AG--------------
------------CCC-TCTGTC-TGTACAT--------------TCGT--AAGCCACTAT
GACTGGAGCTCTCCAGG--------CCCTACGGGTCATAC-TA--------GCCCTTGAG
GGTTT-AAGAGGAAAGGGGGATGGGTGGCACCCCTTC---TT-GGCCCAGGCCACAGACA
GGGGG-TCCTGTGGGGAAGGCTGGTGAGTCAGCCCTGTGTCCC-AGCTCCTTTCTCCAAT
CCTCGGACAG--ATGCAGCCGTTATCTGACCCTGCAGACCCGAACGCTCATCAGCGTGGG
CATCTTCTC
>Dgl
ATTTCCATGGGGATGATCTGCTGCATCCGGAAATACATCCCAGAGGG--GTGAGGAGGCC
CAGCTGAG-GCCTGCAGGCTGGGCTTGGGT---------AGGGTTGTGGG-CA-------
----TGGGG---
--CCCC
AGCTCTGGT----ATCA-CTTTACACCCTG----GGCCTCAGTCTGCTCGTGTCTGCAGT
GAGGAGGCTGAG--------------CTGTCCCT---------AGATGTACTTGCCT---
------------GTG-TCACTG-TCCGGAA--------GACTGGTTGGT----CCCTTGG
GGCATCTGGGAA------------------------------A--------GCACTTGTG
GGTTT-AGGGAGGAAAGGGGA----------------------------------AGATG
GGGA-AGACTGTGGGGAAGGCTGCTGAGCTGGCGCTGTGTCCC-----------------
---TGGACAG--ATGCAGCCATTACCTGACCCTGCAGACCCGAACACTCATCAGCGTGGG
CATCTTCTC
>Dgo
ATTTCCATGGGAATGATCTGCCACATCCGGAAATATATCCCAGAGGG--GTGAGGAAGCC
GAGCC-AGCGGCTGGACGCTGGACTTGGGC----------GGGCCGTGGG-CA-------
---TCGGGCA-GGGA---------------------------GTCGGAGCAGCTGC---
--TGCCC------GGGGTCAGCTGCAGGACA-TTGAAG-AGGGCCCCAGCCTTGCTCCTT
TTCTCCTGG----GTCA-CTCTACATCCCC----GCCCTCAGTTTCCCCATCTGTTCAGT
GAGGAGGTTG---------GCGGG-----------------------------------

-------------------------AGCCCATGTGAGCGGTTG--------GCAC---TG
GGTT---GGGAG--------------------CCCTCGATG-CCCGGGGGTCCCCC-TG
G----CGCTTGGTGTGG-GGCCGTTGAGGCGACACCGAGTCCC-AGCCCCGCTTTCCCAT
ACTTGGACAG--GTGCAGCAATTACCTGACCCTGCAGACGCGAACGCTGATCAGTGTGGG
CATCTTCTC

```
>Pgu
ATCTCCTTGGGACTGATCAATGGCATCCGGAAATATATCCCAGAGGG--GTGAGGAGGCC
AAGG--AGGGCATGGAGATAGGTTGTGGGC---------GGGTCCATGGG-CA-----TT
GGGTGTGGAA-GGGA----------------------------GTCCTGGCTCACGCATC
AGATCCC----------------------------------------ATCATTGCTGCTT
ACCATCTGG----ATCA-CCTCACTCCTTG----GGCCTCAGTTTCTTCATCTGTTAAAT
GAGGAGGTTG---------AACTTCTGGGAGCCCTCTGTCCCTAGACCGAAGAGCCA---
------------TTG-TCACTG-TCCAGTT--------AGACTAGTCTCTTGAGACATCGG
GGAGTCTTGGTCTACAGCTCTTGGAGCACTGTGGATCACCCTA--------GGACTTGTG
GGTAT-GGGGAGGAAAGAGGATGGATGAGACCCATTC---TT-GGCCCAGGCCAGAGATG
GAGAAAACCTGTGGCGAAGGCTGTTGAGCCAGCACTGTGTCCC-AGCTCCTCCTTCCAAT
CCCTGGACAG--AAGCAGCCATTACCTGACCCTGCAGACCCGAACGCTCATCAGCCTGGG
CATCTTCTC
>Tro
ATTTCCATGGGACTGATCTGTACTATCCGGAAATACATCCCAGAGGG--GTGAGAAGGCC
AAG-CCCAGGCTTGGAAGCTGGACTTGGGA---------GGGGCCCTGGA-CA-------
----TGGGCAAGGG----------------------------GATG-------------
------------------------------------------------------------
------------------------------------------------------------
------------------------------------------------------------
------------------------------------------------------------
----------------------------------------------------------TG
AGTTTGGGGAGGGGGGAAGGATGGATGAGACCCATTC---TG-GGCTCAGGCCAGAGGT-
GAGAAAGCATGTGGAGAGGGCCAAGGAGTTGGTACTGTGTCCC-AGCTTCTTCTTCCAAT
CCCTGGCTAG--ATGCAGCGATTACCTGAACCTGCAGACCCGAACACTCATCAGTGTGGG
CATCTTCTC
>Mro
ATTTCCATGGGGATGATCTGCTGCATCCGGAAATATATCCCAGAGGG--GTGAGGAGGCC
AAG--CCAGGGCCTGGGGCTGGACTTGGGT---------GGGGCCCTGGG-CA-------
---TTGGGCA-GGGA----------------------------GATGGAGCAGCTGT---
--TGGGC-AT-TGA-AGAGAGCCTGAGTTCT-GGCAC--CAGACCCCACCACTGCTCCTT
ACTATCTGG----GTCA-CTTCACACCCTG----GGCCTCAGTTTCCTCCTCTGTTAAAT
GAGGAAGTTG---------ACCTGCTGAGAGCCCTCTGTCCCTAGATTTATGAGCCT---
------------GCA-TCACTA-TCTAGAA---------GACTGGTCTCTTGGGACACCTG
GGAATCTTAGTCTGGAGCTCTGGGTGCCCTGTGGATCGCATTA--------GCTCTTGTT
GGCTT-GGGGAGGAGGGAGGATGGATGAGACCCATTC---TT-GGCTCATGCCAGAGATG
GGGACAGCCTGGGGAGAAGGC--CAGAGTTGGCACTGTGTCCC-AGCTCCTTTTTCCAAT
CCCTGGACAG--ATGCAGCCATTACCTGACCCTGCAGACCCGAACACTCATCAGTGTGGG
CATCTTCTC
>Vur
ATTTCCATGGGGATGGTCTGCTGCATCCGGAACTATATCCCGGAGGG--GTGAGGAGGCC
-----CCGGGCCTGGAGGCTGGACTTGGGC---------GGGGCCGCGGG-CA-------
---ACGGGCA-GGGA----------------------------GACAGAGCGGCTGC---
--TGGGC-AT-GGAAAGAGAACCTTGCTCTG-GCGCCAGAT---CCCGCTGCTGCTACTT
ACTGTCTGG----GTCG-CTTCACCCCCTG----GGCCTCAGTTTCCTCATCTGTTAAAT
GAGGAAGTTG---------ACCTGCTGAGAGCCCTCCGTCCCTAGATTTGGGAGCCT---
------------CCC-TCCGTG-TCTGGAA----------------GACTGGTGTCTTGGG
GCACCCGCGCATCTCGGCGCGGAGCTCTGTGG-GTCACGC-TA--------GCCCTTGTG
GGTTT-GGGAAGGAA----------------------------GGCGCAGGCCAGAGATG
GGGCGAGCCTCTGGAGAAGGTCGCTGAGTCAGCACCGTGTCCC-AGCTCCTTTTTCCGAT
CCCTGGACAG--ATGCAGACCTCACCTGAGCCTGCAGAGCCGAACGCTCATCAGCGTGGG
CATCTTCTC
```

```
>human
ATCTCCTTGGGGCTGGTCAGTTGCATCCAGAGATGCCTCCCTCAGGG--GTAAGGAGTGA
AACTGGAAGGGC-ACAGGTGCCACCAGGGA---GGGCTGGGCCCAGCTCC-CA-------
-------AGGCT-GA---------------------------------------------
------------------------------------------------------------
------------------------------------------------------------
------------------------------------------------------------
------------GGTTCCTGAGCTGGGCAGATACAGGACAGCAGCCATTGGCAGTCACGG
GGCA-----GCCCTCCCCTATGACAACCATTGTCTTAGCCCTACATCCGCTCATTTGATG
CAGTCAGACATGA--GTGTGCC-CAGGGAGGTTCTTCCCCTTGGTGTCTCCCCTGAGACA
GT-TCACAGCCA-CCCGAGGCT---GGCCTCAAGAGGACCCCC-TGCAGCCCTTGCCCCT
CTCCA-ATAG--GTGTGGCCCCTACCAGACCCCGCAGACCCGGGCACTTCTCAGCATGGC
CATCTTCTC
```

MARKER 206

```
>WSINE1
------------------------------------------------------------
------------------------------------------------------------
-----------------------------TTTTTTTTTTTTTT-----------------
-GAGGCAATTGGGGTTAAGTGACTTGCCCAGGGTCACACAGCTAGTAAGTGTCTGAGGCT
GGATTTGAACTCAGGTCCTCCTGACTCCAGGGCTGGTGCTCTATCCACTGCACCACCTAG
CTGCCCCTC---------------------------------------------------
---------------------------
>Mdo
CCTTAGGAAAACA--CTGTAGGGGAAGGGCAGAAAAAGGCACTA-CTGCTTTAGCTTAGC
---------------------TTTAGTTTAGCTTCCCAAGCC-AGTTCCTCCACTGC-AG
CGGAGCCTCC---TCCCTGTCTAG------------------------------------
------------------------------------------------------------
------------------------------------------------------------
--------------------------TTTTCAACAGAA--TTTCTGCTCACGTCTCCCAG
CACCCTGAATTAC--CTTCTTCTCCTT
>Dvi
CCTTAGGAAAACA--CTGTAGGGGGATTGTAGAACAAGGCGTTA-CTGCTTTAGGTTAGC
CCCATCTAGTC----------TTTAGCTTAGTTTTCCAATCCAAATTCCTTAACCCC-AG
CCATGCTTTA---TCCCTGTCCAG------------------------------------
------------------------------------------------------------
------------------------------------------------------------
---------------------------TTTCAACAGAA--TTTCTACTAACTTCTCCCAG
CACCCTGTATTAC--CTTCTTCTCCTT
>Mnu
CCTTAGGAAAACA--CTGTAGGGGAAATGTAGAACAAGGCATTA-CTGCTTTAGGTTAGC
CCCATCTAGTC----------TTTAGCTTAGTTTTCCAATCAAAATTCCTTAACTCC-AG
CCATGCTTTG---TCCCTGTCCAG------------------------------------
------------------------------------------------------------
------------------------------------------------------------
---------------------------TTTCAACAGAA--TTTCTACTAACTTCTCCCAG
CACCCTGTATTAC--CTTCTTCTCCTT
>Bil
CCTTAGGAAAACA--CTGCAGGGGAGGAATAGAGAAACACATTA-CTACTTTAAGTCGGC
ACCATTCAGTCAGCAGAAA-TCATAGC-----TTTTC-ATCCAAATTCCTTAACACCAAT
TAAAGTGTCC---TACCCCTCTAG------------------------------------
------------------------------------------------------------
------------------------------------------------------------
---------------------------TTTCAACAGAA-ATTTCTATTAACTTCCCCCAG
AACCCTCTATTAC--CTTTTTTTCCTT
>Pgu
CCTTAGGAAAACA--CTGCAGGGGAAGAAAAGAGAAATGCATTA-CTATTTTAAGTTGGC
ATCATCTAGTCGGCAGAAA-TCATAGC-----TTTTCAATCCAAATTCCTTAACACCAAT
TAAAGTGTTC---TAACCCTCTAG------------------------------------
------------------------------------------------------------
------------------------------------------------------------
---------------------------TTTCAACAGAA-GTTTCCACTAACTTCCCCCAG
AACCCTCTATTAC--CTTTTTTTCCTT
>Tro
CCTTAGGAAAACA--CTGCAGA-GAAAGAACAGAAAATGCATTAACTGCTCTAGGTTGGC
ACCACTTAGCCAACAGAAC-----TGC-----TTTTCAGTTCAAATTCCTTAATACC-AG
```

TTAAGCTTCC---TGTTCCT-TAG----------TATTTTTGGGGGGGGGGTGAAGAGGG
GGAGGCAATTAGAGTTAAGTGACCTGCCCAGGGTCACACAGCTAGT----GTATGAAGTG
GGATTTTAACTCAGGTCTTCACC--TCCACAGCCAGTACTCTTTCTACTG-CCCCCT-CA
CTGCCTC-C---TGCCCCTCTAG----TTTCAACAGCA--TTTCTACTAACTTCCCTTGG
CACCTTCTGTTAC--CTTCTTCTCCTT
>Ppe
CCTTAGGAAAACA--CTGCAGGGGAAGAACAGAAAAATGCATTAACTGCTTTAGGTTGGC
ATCATTTAGTCAACAGAAATCGTATGC-----TTTTCAGTTAAAATTCCTTAACACC-AG
-----CTTCC---TGCCCCTCTAG------TTTTTTTGGGGGGTAGGGGGGTGGG--GAG
GGAGGCAATTGAAATTAAGTGACTTGCCCGGGGTCACACATCTAGT----GTCTGAAGCA
GAATTTGATGTCAGGTCCTCAAC--TCCAGGGCCAGTATGCTATCAACTGCACC--TACC
CTGCCCT-C---TGCCCCTCTAG----TTTCAACAGAA--TTTCTACTAACATCCCTCAG
CACCCACTGTTAC--CTTCTTCTCCTT
>Mro
CCTTAGGAAAACA--CTGCAGGGGAAGAATAGAAAAATGCATTAACTACTTTAGGTTGGC
ACCATTTAGTCGATAGAAG-TCATAGC-----TTTTCAGTTCAAATTCCTTAACACC-AG
CTATGCTTCC---TGCTCCTCTAG---TTTCTTTTCTTTTTTTGGGGGGGGGGGGTGAGA
GGAGGCAATTAG--TTAGGTGACTTGCCCAGGGTCACACATCTAGT----GTCTGAAGCA
GGATTTGAACTCGGGTCCTG-AC--TCCAGGGCCAGTGCTCTACCCCCTGCACCACCTAA
CTGCCCCCT---TGCCCCTCTAT----TTTCAACAGAATTTTTCCACTAACTTCCCTGAG
CACCCTTTGTTAC--CTTCTTCTCCTT
>Vur
CCTTAGGAAAACA--CTGCAGGGGAAGAATAGAAAAATGCATTAACTGCT---ATTTGGC
ACCATTTAGTCAACAGAAG-TCATTGC-----TTTTCAGTCCAAATTCCTTAACATC-AA
CTAAGCTCCC---TGCCCCTCTAG-------TTTCTTTCTTTCTTTTTTTGGGGGGGGGA
GGAGGCAATTGGGATTAAGTGACTTGCCCAGGGTCACACAGCTAGT----GTCTGAGGCA
GCATTTGAACTCAGGTCTTCCTGACTTCAGGGCCCATGCTCTATCCACTGCACCACTTAA
CTGCCCC-T---TGCCCCTCTAG----TTTCAACAGAA---TTCTGCTAACTTCCTCCAG
CACCTTTTGTTAC--CTTTTTTTCCTT
