# Supplementary figures and images for: Tracking Marsupial Evolution Using Archaic Genomic Retroposon Insertions
Source: PLoS Biol. 2010 Jul 27;8(7):e1000436. doi: 10.1371/journal.pbio.1000436 (PMC2910653; doi:10.1371/journal.pbio.1000436)

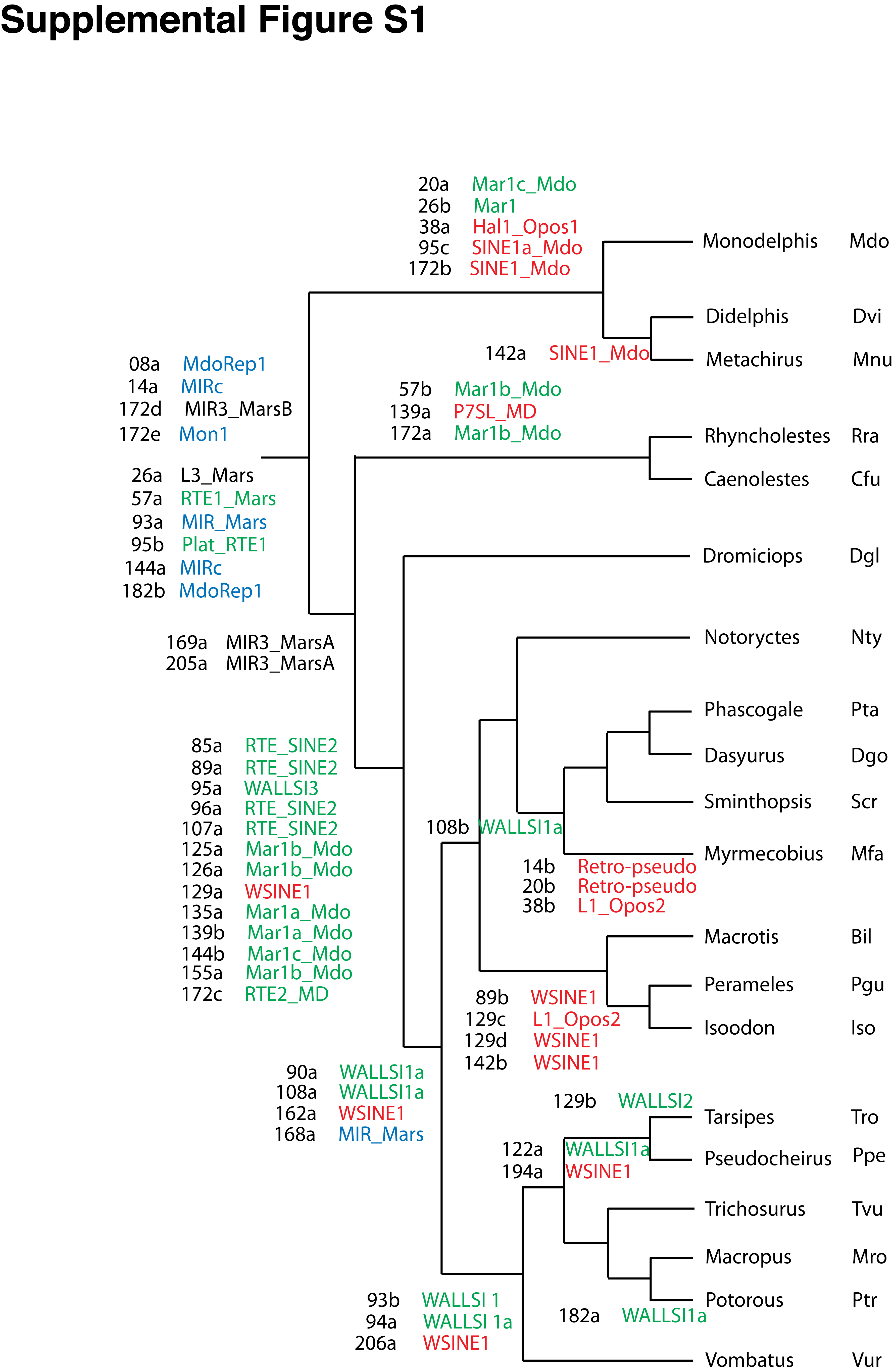

Supplement: Figure S1 — Marker location and SINE retroposon subtypes. Marker location and SINE retroposon subtypes (red = L1-, green = RTE-, blue = L2-, black = L3-mobilized SINEs; see also Figure 1). The numbering of the elements corresponds to Table 1. (3.18 MB JPG) [file pbio.1000436.s002.jpg]

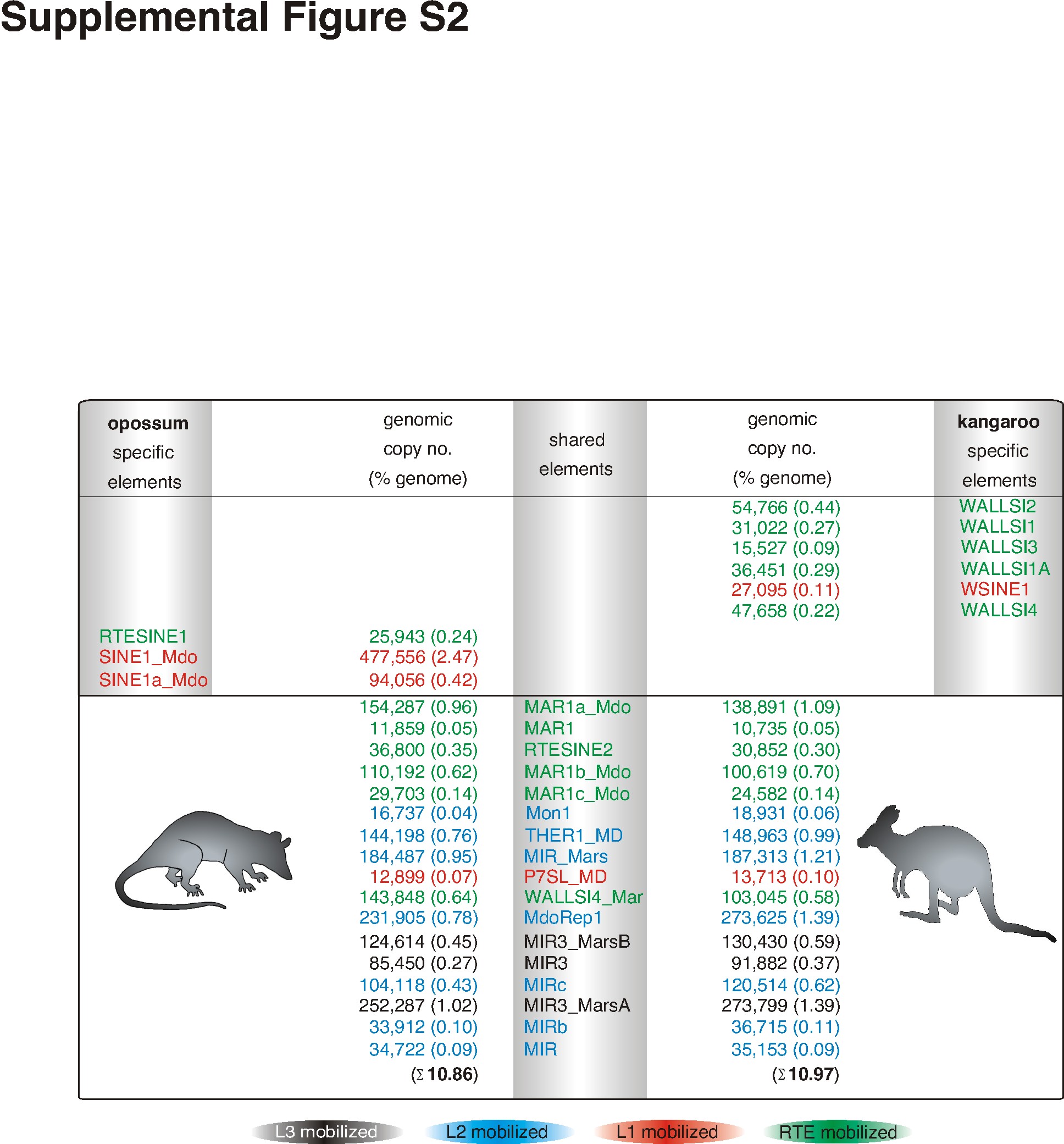

Supplement: Figure S2 — Compilation of genomic copies of SINE elements in opossum and kangaroo. Compilation of genomic copies of SINE elements in opossum and kangaroo (red = L1-, green = RTE-, blue = L2-, black = L3-mobilized SINEs; see also Figure 1). (0.41 MB JPG) [file pbio.1000436.s003.jpg]

**Table S1.** TinT matrices


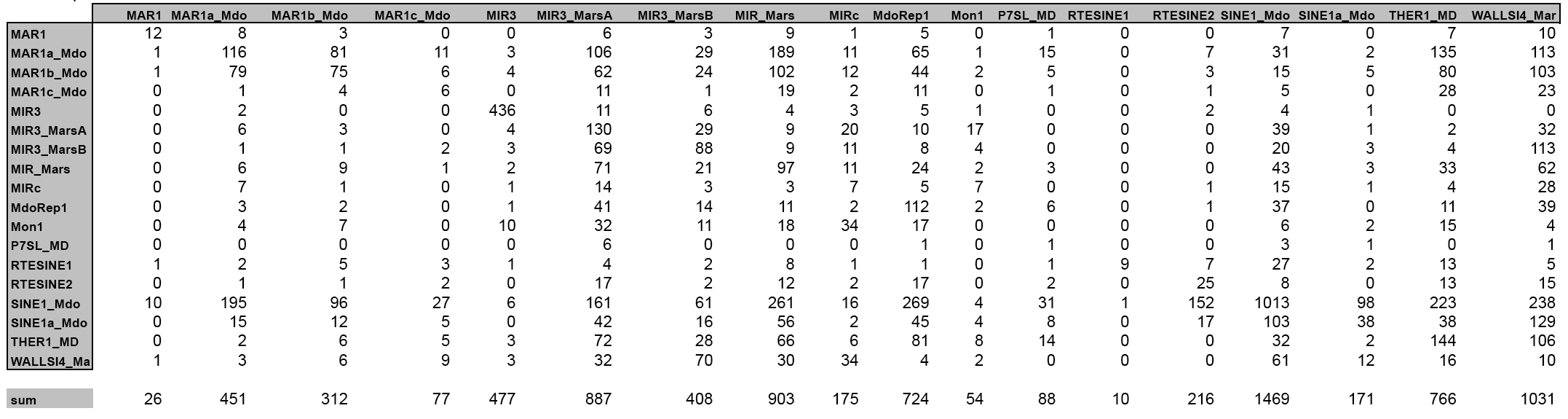


Table S1a


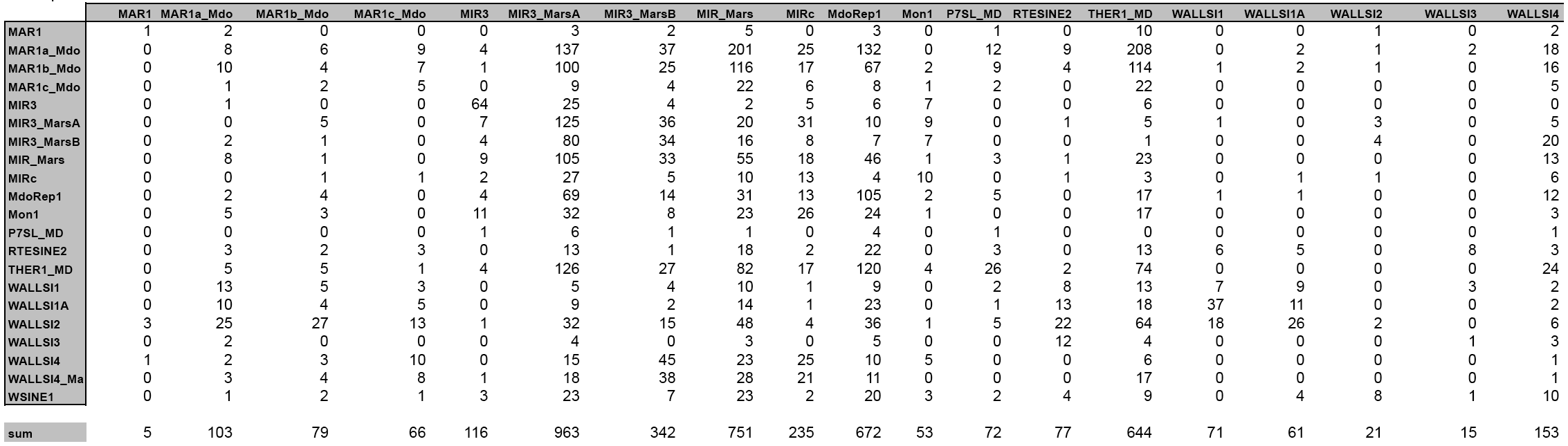


Table S1b

Supplement: Table S1 — TinT matrices. (a) Transpositions in transpositions in opossum. (b) Transpositions in transpositions in kangaroo. (0.29 MB DOC) [file pbio.1000436.s005.doc]
