# Supplementary material for: Tracking Marsupial Evolution Using Archaic Genomic Retroposon Insertions
Source: PLoS Biol. 2010 Jul 27;8(7):e1000436. doi: 10.1371/journal.pbio.1000436 (PMC2910653; doi:10.1371/journal.pbio.1000436)
Supplement: Figure S3 — Presence/absence data matrix and phylogenetic reconstruction. Presence/absence data matrix and phylogenetic reconstruction. (A) presence (1) and absence (0) matrix of orthologous SINE elements. Question marks denote missing data. (B) Strict consensus parsimonious tree from six equally parsimonious trees, using the irrev.up option of character transformation (PAUP* 4.0b10), heuristic search (1,000 random sequence addition), and TBR branch swapping. Human was used as outgroup. Treelength: 53; Consistency Index = 1. (0.83 MB DOC) [file pbio.1000436.s004.doc]

# **Supplemental Figure S3**

## **A**

begin data;

dimensions ntax=21 nchar=53;

format transpose;

taxlabels Mdo Dvi Mnu Rra Cfu Dgl Nty Pta Dgo Scr Mfa Bil Pgu Iso Tro Ppe Tvu Mro Ptr Vur Hsa;

matrix

char1 111?111?1?11??1111110

char2 0000??01111000?0?00?0

char3 1111??11111111?1?11?0

char4 111?000?0000000000000

char5 000?000?1110000000000

char6 111?11?1?????11111110

char7 111?00?0?????00000000

char8 1?10000000000000?0000

char9 0?00000111100000?0000

char10 111111111111111??11?0

char11 000110000000000??00?0

char12 0???01?11111111?11110

char13 000001????11111111110

char14 000000????01110000000

char15 00???01111?1??1111??0

char16 111111?1111???1?11110

char17 000000?0000???1?11110

char18 00000?0?00?0001111?10

char19 0?0?01111?????1??1??0

char20 1?1?00000?????0??0??0

char21 1?1?11111?????1??1??0

char22 0000?111?1111?1111110

char23 000001?111111111?1?10

char24 00?000111111111111110

char25 00?000111111110000000

char26 0??000000000001111100

char27 000001?111111111?1110

char28 0???011111??1111?1?10

char29 00000111?111111111?10

char30 00000000?000001100?00

char31 00000000?001110000?00

char32 00000000?001110000?00

char33 000?011111?1??1111110

char34 00?1100000?000??00000

char35 00?0011111?111??11110

char36 011???0??0?000?0?0?00

char37 000???0??0?111?0?0?00

char38 111111?11?1?1111111?0

char39 000001?11?1?1111111?0

char40 0000011111111111?1?10

char41 000?001?11111111?1??0

char42 000000?1?11?1?1111110

char43 0001111111?1111111?10

char44 000110000??00000?0??0

char45 111000000??00000?0??0

char46 000001111??11111?1??0

char47 111111111??11111?1??0

char48 111111111??11111?1??0

char49 000?????00??000001100

char50 111?????11??111111110

char51 00??????00?0???111?00

char52 00?1111?1???1?1??1?10

char53 000????????00?11?1?10

;

end;

## **B**

**
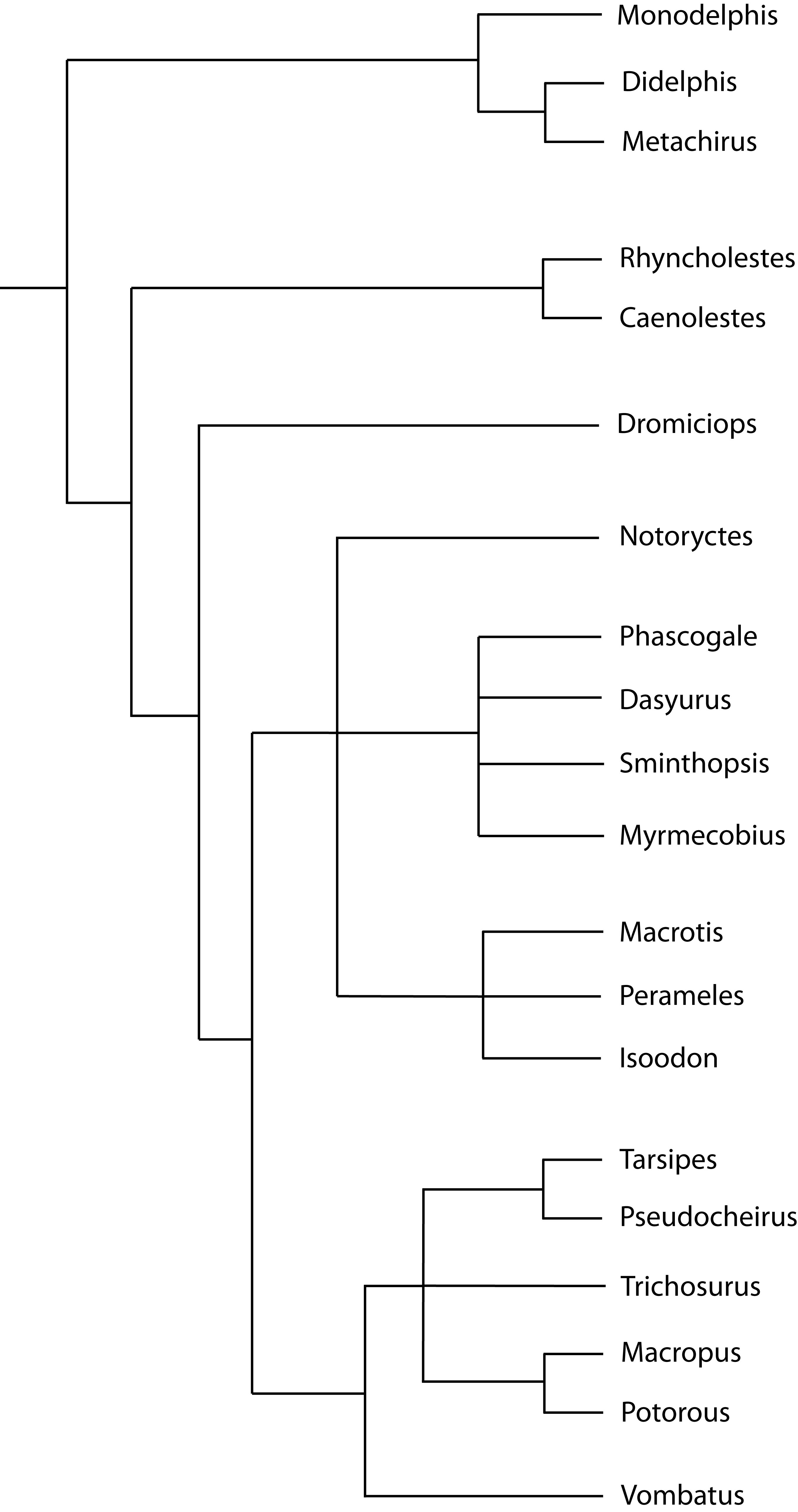
**

**Figure S3:** Presence/absence data matrix and phylogenetic reconstruction. ***A.*** presence (1) and absence (0) matrix of orthologous SINE elements. Question marks denote missing data. ***B.*** Strict consensus parsimonious tree from 6 equally parsimonious trees, using the irrev.up option of character transformation (PAUP* 4.0b10), heuristic search (1000 random sequence addition), and TBR branch swapping. Human were used as outgroup. Treelength: 53; Consistency Index = 1.

**Marker code**

char1 08a

char2 14a

char3 14b

char4 20a

char5 20b

char6 26a

char7 26b

char8 38a

char9 38b

char10 57a

char11 57b

char12 85a

char13 89a

char14 89b

char15 90a

char16 93a

char17 93b

char18 94a

char19 95a

char20 95b

char21 95c

char22 96a

char23 107a

char24 108a

char25 108b

char26 122a

char27 125a

char28 126a

char29 129a

char30 129b

char31 129c

char32 129d

char33 135a

char34 139a

char35 139b

char36 142a

char37 142b

char38 144a

char39 144b

char40 155a

char41 162a

char42 168a

char43 169a

char44 172a

char45 172b

char46 172c

char47 172d

char48 172e

char49 182a

char50 182b

char51 194a

char52 205a

char53 206a
