# Supplementary material for: Tracking Marsupial Evolution Using Archaic Genomic Retroposon Insertions
Source: PLoS Biol. 2010 Jul 27;8(7):e1000436. doi: 10.1371/journal.pbio.1000436 (PMC2910653; doi:10.1371/journal.pbio.1000436)
Supplement: Table S2 — The accession numbers for the established marsupial sequences. (0.06 MB DOC) [file pbio.1000436.s006.doc]

**Table S2**. The accession numbers for the established marsupial sequences.

|  | Accession numbers |
| --- | --- |
| 08 | FN661519-FN661532 |
| 14 | FN661533-FN661546 |
| 20 | FN661547-FN661563 |
| 26 | FN661564-FN661574 |
| 38 | FN661575-FN661591 |
| 57 | FN661592-FN661607 |
| 85 | FN661608-FN661621 |
| 89 | FN661622-FN661636 |
| 90 | FN661637-FN661647 |
| 93 | FN661648-FN661658 |
| 94 | FN661659-FN661673 |
| 95 | FN661674-FN661681 |
| 96 | FN661682-FN661697 |
| 107 | FN661698-FN661713 |
| 108 | FN661714-FN661723 |
| 122 | FN661724-FN661740 |
| 125 | FN661741-FN661757 |
| 126 | FN661758-FN661769 |
| 129 | FN661770-FN661786 |
| 135 | FN661787-FN661801 |
| 139 | FN661802-FN661816 |
| 142 | FN661817-FN661825 |
| 144 | FN661826-FN661840 |
| 155 | FN661841-FN661857 |

| 162 | FN661858-FN661871 |
| --- | --- |
| 168 | FN661872-FN661883 |
| 169 | FN661884-FN661900 |
| 172 | FN661901-FN661914 |
| 182 | FN661915-FN661926 |
| 194 | FN661927-FN661934 |
| 205 | FN661935-FN661943 |
| 206 | FN661944-FN661951 |
