# Supplementary material for: Tracking Marsupial Evolution Using Archaic Genomic Retroposon Insertions
Source: PLoS Biol. 2010 Jul 27;8(7):e1000436. doi: 10.1371/journal.pbio.1000436 (PMC2910653; doi:10.1371/journal.pbio.1000436)
Supplement: Table S3 — The primers used for amplification of single copy marsupial introns containing retroposed elements. The primers used for amplification of single copy marsupial introns containing retroposed elements. The location of each marker on the chromosomes (Chr.) in the Monodelphis genome is listed. (0.07 MB DOC) [file pbio.1000436.s007.doc]

**Table S3**.

|  | Forward Primer | Reverse primer | Chr. | Location | Gene |
| --- | --- | --- | --- | --- | --- |
| 08 | GGC AGG GAT GCA GAT CAG | AGG TTC TGA GGG TAA GAG CC | 1 | 50,489,157-50,489,803 | SEC24C |
| 14 | GTA ACC GAG ACT CGG TCC | GAT GGG ATC CTC ATG TCA C | 1 | 497,689,635-497,690,524 | PREX1 |
| 20 | GTA CAT TTT TGG AGG ATA TG | CGC AAA TTA ACA AAA TAG AG | 1 | 275,833,696-275,834,433 | KLHDC1 |
| 26 | CCT GTT TAG CAC TGA TCA G | GTT CAC AGA ATG TTA GCT TC | 2 | 300,365,534-300,366,612 | PKHD1 |
| 38 | GGG TCC TTT GTC ACT GAA G | CTA TCA TTT ACT TCA TCT GAA G | 2 | 432,480,455-432,481,128 | SYNE1 |
| 57 | CAA TCT CTA TTA AAC GCT GC  CTT TCT CCA CAT CTT TTC C | GAC GAC AAA ATA TGT GGA AC  GGA CAA TCG CGA GGT AAG | 1 | 37,987,932-37,988,461 | ADAM12 |
| 85 | CTG TTT CTG ATC CGC TCC | GCT GCC CAC TTC ATA ATA G | 5 | 23,030,529-23,030,792 | PPEF2 |
| 89 | GCT GCC CAC TCT GTG ATG G | GCC TGG CTG GAA ACT CTC | Un | 148,257,330-148,257,726 | KCP |
| 90 | GAC CCA TAT GAT GTG TAT TG  GGG ACA GAA GGT AGG GAG | GAG GAC TTT GGA GAC TGG  CAC GGT TTG GCA TTC TCA CA | 6 | 155,026,136-155,026,701 | RPUCD1 |
| 93 | GTC TGT GGT ACA CCC TGG  GCC TCC AAA TGG TAA TCC | GAG ATG ATT CTT GAT CAT GTC  GCT ATA CTT CCA ATC TGA AAT GGC | 5 | 240,053,705-240,054,501 | CYB5R2 |
| 94 | GAA AGG TCA GTT TCC TGG | GCC TCC TGA GAT GAT ACA C | 3 | 263,828,834-263,829,303 | GREB1L |
| 95 | CAT CAG TCG TCA GAA TAA ATC TG | CCA CTG GAG AAG GGA GTT TC | 1 | 329,411,635-329,412,698 | KDM3a |
| 96 | CAA AGC TGT GTG GTT ACC  CTG GCC ATT GTT ACT GCA GAC | CAA GAG AAA GTA GAA GGT TGG  CAA TGC ATC AAC TGA GAG GTC | 4 | 352,865,707-352,866,112 | UBR4 |
| 107 | GGA CTG TAA CAA GGA GCA G | GTG CAC TGT GAA CAC CAG | 4 | 370,127,844-370,128,440 | PLCD1 |
| 108 | CAG ATC CCT ATG TGA AGC TGA  CTT ATC CCT GAC CCT AAG AAT G  ACA GTG GAA TGA RTC ATT CAC ATT GT  GTG AAG CTG AAA CTT ATT CCT GAC C | GTT CGA TCC CAG TCC CAG  CGT CTA TCT TTA TCT GTA GG  CTA TCT TTR TCT GTA GGT TTT AAT TTG CT  CCA GTC CCA GAT CTC RAC AGA TAA | 2 | 227,612,146-227,612,671 | PRKCA |
| 122 | GAA GAT GTC TTC TTT GAG C | CAT GAA CTC CCC AAA GTG | 3 | 435,645,702-435,646,043 | HETR7A |
| 125 | CTA TGT CTA GGG AGC TCA TCC | CTC ATC CCA GGA GCT GCC | 1 | 109,299,441-109,299,975 | SFXN2 |
| 126 | CAG TGA GTG TCT TAT GTC AG | CTA CCA CTT TAA GGA AAT C | 1 | 201,611,646-201,612,062 | STRC |
| 129 | CAT GGC CTT TGG CTC ACT C | CCT CTT GTC TAC ATT CTG GTC ATG | 1 | 502,502,165-502,502,561 | OGDH |
| 135 | CAA GTT CAT CAT GTT CAG | CCA GAG GAA TTA GAA TTT AGA C | 1 | 515,946,556-515,946,919 | BIRC6 |
| 139 | GAT CCT GGA ATT TGA GAA CC | CAA TCT CCT TCA GGT GGC | 2 | 525,803,992-525,804,415 | RABL5 |
| 142 | CAT CTC CCT TCA TCA TCC | GAC TAC AGC AAG GAG ATC AC | 2 | 280,701,124-280,701,748 | WRAP53 |
| 144 | GAA CAG ACT TTC CGT TGG | GAA AAT TTC CCA TTC ATC CC | Un | 50,534,576-50,535,140 | GNPTG |
| 155 | GAC TTT ACC TGC ATG TAT GC | CCT AGA GAC CAG CTT CGG | 8 | 116,742,169-116,742,580 | MICAL3 |
| 162 | GCC AAT GGA GTG AAG TTT G | CTC TTA CAT TGG TCC AGC C | 1 | 111,396,504-111,397,239 | PZDZ7 |
| 168 | CTG CCC CAC AAC TAT GAC CC  GTA CAA GCG CAG TCT KCG GT  GGC CAG CGT CAT CAT CTA TCG | GGA CGC TGA TGC TCA CCA C  AGG MCG TTT GGG GAC CCT  CCA ACT CAC AGA ATW GAG TGA TTC CAG | 2 | 478,350,311-478,350,634 | CELSR2 |
| 169 | CAG CCA TGA ATG TCA ATG ACT CTG | CAT CTG TGG TCC TCT TGA GGC | 2 | 479,332,236-479,332,593 | AHCYL1 |
| 172 | CCA CTG AGT TAC ACC TTT CTC AG | CAA GGT ATG CTG TGA TAT TCA TCA C | 4 | 226,939,303-226,940,209 | SORL1 |
| 182 | GAC TCT GGA CTT CAT TGA TGT GC | CTT CTG CTC GGA TGT CCT C | 3 | 473,235,124-473,235,735 | CYP4F22 |
| 194 | GAC AGT AGT GGG AGT GGA C | GTG AGG CTT CTC TTC AGA G | 3 | 529,804,345-529,805,019 | POM121 |
| 205 | CCT CCT ACT GCA ATG TGG AC | GTC ACC CAG ACT CCG GTG | 8 | 15,498,831-15,499,231 | CPT1B |
| 206 | GCC ATT GGC TAT TAT CTC | CAG CCA TCA TGT CAA CTC | 8 | 27,242,153-27,242,360 | OR2T8 |
